# Supplementary material for: Functional role of the type 1 pilus rod structure in mediating host-pathogen interactions
Source: eLife. 2018 Jan 18;7:e31662. doi: 10.7554/eLife.31662 (PMC5798934; doi:10.7554/eLife.31662)
Supplement: Supplementary file 1. [file elife-31662-supp1.docx]

**Validation statistics for FimA model**

| **Phenix Real-Space Refinement** | |
| --- | --- |
| Map CC (around atoms) | 0.68 |
| **MolProbity Score** | **1.90** |
| Ramachandran outliers | 0.00% |
| Ramachandran favored | 92.21% |
| Ramachandran allowed | 7.8% |
| Poor rotamers | 0 |
| RMSD bonds (Å) | 0.00 |
| RMSD bond angles (°) | 0.85 |
| All atom clashscore | 7.75 |
