# Supplementary material for: Functional role of the type 1 pilus rod structure in mediating host-pathogen interactions
Source: eLife. 2018 Jan 18;7:e31662. doi: 10.7554/eLife.31662 (PMC5798934; doi:10.7554/eLife.31662)
Supplement: Supplementary file 2. [file elife-31662-supp2.docx]

**Helical parameters comparison within FimA models**

| Rotation(°)  /Translation(Å) | FimA cryo-EM model | Solid NMR FimA model  PDB code ID:2N7H | Solid NMR FimA model  PDB code ID:2MX3 |
| --- | --- | --- | --- |
| N-N+1 | 115.0/7.7 | 111.5/7.2 | 111.5/8.7 |
| N-N+2 | 130.0/15.4 | 137.0/14.4 | 137.0/17.5 |
| N-N+3 | 15.1/23.0 | 25.5/21.6 | 25.5/26.2 |
| N-N+4 | 99.9/30.7 | 86.0/28.8 | 86.0/34.9 |
