# Supplementary material for: Functional role of the type 1 pilus rod structure in mediating host-pathogen interactions
Source: eLife. 2018 Jan 18;7:e31662. doi: 10.7554/eLife.31662 (PMC5798934; doi:10.7554/eLife.31662)
Supplement: Supplementary file 3. [file elife-31662-supp3.docx]

| Number | GeneID | ENA_ID | Description | Species | Bit Score | E-value | Representative Sequence |
| --- | --- | --- | --- | --- | --- | --- | --- |
| 1 | A1S3_00303 | ELD93555 | type-1 fimbrial protein, A chain | Escherichia coli KTE47 | 369.9 | 9.9E-108 |  |
| 2 | G688_04331 | EQN26608 | type-1 fimbrial protein, A chain | Escherichia coli HVH 9 (4-6942539) | 369.9 | 9.9E-108 |  |
| 3 | WKY_04683 | ELJ47442 | fimbrial protein FimA | Escherichia coli KTE180 | 369.9 | 9.9E-108 |  |
| 4 | G786_04682 | EQR43915 | type-1 fimbrial protein, A chain | Escherichia coli HVH 126 (4-6034225) | 369.9 | 9.9E-108 |  |
| 5 | ECOK1_4812 | ADE92610 | type-1 fimbrial protein | Escherichia coli IHE3034 | 369.9 | 9.9E-108 |  |
| 6 | A1SI_00416 | ELE16036 | type-1 fimbrial protein, A chain | Escherichia coli KTE55 | 369.9 | 9.9E-108 |  |
| 7 | SC80_07005 | KIE82459 | type-1 fimbrial protein subunit A | Escherichia coli RS218 | 369.9 | 9.9E-108 |  |
| 8 | UTI89_C5011 | ABE10414 | type 1 fimbriae major subunit FimA | Escherichia coli UTI89 | 369.9 | 9.9E-108 | Yes |
| 9 | A1YC_00304 | EOW41399 | fimbrial protein FimA | Escherichia coli KTE126 | 369.9 | 9.9E-108 |  |
| 10 | G717_04822 | EQO56927 | type-1 fimbrial protein, A chain | Escherichia coli HVH 42 (4-2100061) | 369.9 | 9.9E-108 |  |
| 11 | NRG857_21765 | ADR29760 | type 1 fimbriae major subunit FimA | Escherichia coli O83:H1 str. NRG 857C | 369.9 | 9.9E-108 |  |
| 12 | WKW_04668 | ELJ46785 | fimbrial protein FimA | Escherichia coli KTE179 | 369.9 | 9.9E-108 |  |
| 13 | G997_04670 | ERA14686 | type-1 fimbrial protein, A chain | Escherichia coli UMEA 3834-1 | 369.9 | 9.9E-108 |  |
| 14 | G961_04746 | ERB28780 | type-1 fimbrial protein, A chain | Escherichia coli UMEA 3298-1 | 369.9 | 9.9E-108 |  |
| 15 | G743_01791 | EQP82363 | type-1 fimbrial protein, A chain | Escherichia coli HVH 80 (4-2428830) | 369.9 | 9.9E-108 |  |
| 16 | AB60_4819 | KDV96356 | type-1 fimbrial protein, A chain | Escherichia coli 2-156-04_S1_C3 | 369.9 | 9.9E-108 | Yes |
| 17 | ECOPMV1_04760 | CDH68041 | Type-1A pilin | Escherichia coli PMV-1 | 369.9 | 9.9E-108 |  |
| 18 | AB747_20735 | KNX99844 | type-1 fimbrial protein subunit A | Escherichia coli (strain UTI89 / UPEC) | 369.9 | 9.9E-108 |  |
| 19 | G981_04505 | EQZ45607 | type-1 fimbrial protein, A chain | Escherichia coli UMEA 3632-1 | 369.9 | 9.9E-108 |  |
| 20 | A17W_03402 | ELH96319 | fimbrial protein FimA | Escherichia coli KTE229 | 369.9 | 9.9E-108 |  |
| 21 | L282_2734 | AJB37696 | type 1 fimbriae major subunit FimA | Escherichia coli APEC IMT5155 | 369.9 | 9.9E-108 |  |
| 22 | G683_04699 | EQN00201 | type-1 fimbrial protein, A chain | Escherichia coli HVH 3 (4-7276001) | 369.9 | 9.9E-108 |  |
| 23 | APECO1_2116 | ABJ03821 | type 1 fimbriae major subunit FimA | Escherichia coli APEC O1 | 369.4 | 1.4E-107 |  |
| 24 | ERS139198_00300 | CUA00170 | type 1 fimbriae major subunit FimA | Escherichia coli (GCA_001286605) | 368.8 | 2.3E-107 | Yes |
| 25 | T638_06370 | KKJ21616 | type-1 fimbrial protein subunit A | Escherichia coli MRSN 10204 | 368.4 | 3E-107 | Yes |
| 26 | ECDEC1E_0064 | EHU32350 | fimbrial subunit type 1 | Escherichia coli DEC1E | 368.2 | 3.4E-107 |  |
| 27 | A1UC_05348 | EOV68212 | fimbrial protein FimA | Escherichia coli KTE70 | 368.2 | 3.4E-107 |  |
| 28 | WGG_04591 | ELF78850 | fimbrial protein FimA | Escherichia coli KTE43 | 368.2 | 3.4E-107 |  |
| 29 | ECDEC2B_0010 | EHU45220 | fimbrial subunit type 1 | Escherichia coli DEC2B | 368.2 | 3.4E-107 |  |
| 30 | ERS085359_04941 | CTT85305 | type 1 fimbriae major subunit FimA | Escherichia coli HVH 87 (4-5977630) | 368.2 | 3.4E-107 | Yes |
| 31 | A1UA_05223 | EOV58202 | fimbrial protein FimA | Escherichia coli KTE69 | 368.2 | 3.4E-107 |  |
| 32 | G962_04127 | EQY78984 | type-1 fimbrial protein, A chain | Escherichia coli UMEA 3304-1 | 368.2 | 3.4E-107 |  |
| 33 | ECDEC2C_5322 | EHU34979 | fimbrial subunit type 1 | Escherichia coli DEC2C | 368.2 | 3.4E-107 |  |
| 34 | ECDEC2A_0092 | EHU33783 | type-1 fimbrial protein, A chain | Escherichia coli DEC2A | 368.2 | 3.4E-107 |  |
| 35 | ECDEC2E_5225 | EHU48711 | fimbrial subunit type 1 | Escherichia coli DEC2E | 368.2 | 3.4E-107 |  |
| 36 | G935_02340 | EQX79709 | type-1 fimbrial protein, A chain | Escherichia coli UMEA 3190-1 | 368.2 | 3.4E-107 |  |
| 37 | ECDEC1B_5215 | EHU04575 | fimbrial subunit type 1 | Escherichia coli DEC1B | 368.2 | 3.4E-107 |  |
| 38 | ECDEC1C_5245 | EHU02448 | fimbrial subunit type 1 | Escherichia coli DEC1C | 368.2 | 3.4E-107 |  |
| 39 | ECDEC1A_5104 | EHU02692 | fimbrial subunit type 1 | Escherichia coli DEC1A | 368.2 | 3.4E-107 |  |
| 40 | G749_05040 | EQP98040 | type-1 fimbrial protein, A chain | Escherichia coli HVH 87 (4-5977630) | 368.2 | 3.4E-107 |  |
| 41 | G694_04553 | EQN58586 | type-1 fimbrial protein, A chain | Escherichia coli HVH 18 (4-8589585) | 368.2 | 3.4E-107 |  |
| 42 | ECDEC2D_0035 | EHU49404 | fimbrial subunit type 1 | Escherichia coli DEC2D | 368.2 | 3.4E-107 |  |
| 43 | EC236275_1844 | EFR17170 | fimbrial subunit type 1 | Escherichia coli 2362-75 | 368.2 | 3.4E-107 | Yes |
| 44 | UM146_22260 | ADN73776 | type 1 fimbriae major subunit FimA | Escherichia coli UM146 | 366.9 | 8.5E-107 | Yes |
| 45 | A1UK_04964 | EOV83209 | fimbrial protein FimA | Escherichia coli KTE74 | 364.9 | 3.4E-106 |  |
| 46 | A1YW_00069 | ELF22954 | type-1 fimbrial protein, A chain | Escherichia coli KTE143 | 364.9 | 3.4E-106 |  |
| 47 | LF82_0658 | CAP78795 | Type-1 fimbrial protein, A chain | Escherichia coli LF82 | 364.1 | 6.2E-106 |  |
| 48 | ECTW07793_4519 | EII95126 | type-1 fimbrial protein, A chain | Escherichia coli TW07793 | 363 | 1.3E-105 | Yes |
| 49 | ERS085395_03359 | CTV08955 | type 1 fimbriae major subunit FimA | Escherichia coli | 361.8 | 3.2E-105 | Yes |
| 50 | A1W3_00475 | ELG39199 | fimbrial protein FimA | Escherichia coli KTE84 | 361.8 | 3.2E-105 |  |
| 51 | G921_02631 | EQX06246 | type-1 fimbrial protein, A chain | Escherichia coli UMEA 3155-1 | 360.2 | 9.6E-105 |  |
| 52 | ECIG_03245 | EGI12987 | type 1 fimbriae major subunit FimA | Escherichia coli M605 | 360.2 | 9.6E-105 |  |
| 53 | G820_04527 | EQS74803 | type-1 fimbrial protein, A chain | Escherichia coli HVH 162 (4-5627982) | 360.2 | 9.6E-105 |  |
| 54 | ECAA86_00017 | EGH38260 | type 1 fimbriae major subunit FimA | Escherichia coli AA86 | 360.2 | 9.6E-105 | Yes |
| 55 | G842_00611 | EQT71815 | type-1 fimbrial protein, A chain | Escherichia coli HVH 190 (4-3255514) | 360.2 | 9.6E-105 |  |
| 56 | G769_04513 | ESP40218 | type-1 fimbrial protein, A chain | Escherichia coli HVH 108 (4-6924867) | 359.6 | 1.5E-104 |  |
| 57 | WEO_04650 | ELC45408 | type-1 fimbrial protein, A chain | Escherichia coli KTE28 | 359.6 | 1.5E-104 | Yes |
| 58 | A1WI_00370 | EOW04855 | fimbrial protein FimA | Escherichia coli KTE98 | 354.7 | 4.9E-103 | Yes |
| 59 | G719_04638 | EQO66418 | type-1 fimbrial protein, A chain | Escherichia coli HVH 44 (4-2298570) | 354.4 | 5.9E-103 |  |
| 60 | G686_04535 | EQN19237 | type-1 fimbrial protein, A chain | Escherichia coli HVH 6 (3-8296502) | 354.4 | 5.9E-103 |  |
| 61 | G931_04474 | EQX50464 | type-1 fimbrial protein, A chain | Escherichia coli UMEA 3176-1 | 354.4 | 5.9E-103 |  |
| 62 | AF43_04044 | KDG61511 | type-1 fimbrial protein, A chain | Escherichia coli MGH 57 | 354.4 | 5.9E-103 |  |
| 63 | G836_04862 | EQT38036 | type-1 fimbrial protein, A chain | Escherichia coli HVH 184 (4-3343286) | 354.4 | 5.9E-103 |  |
| 64 | AD08_4710 | EYD94273 | type-1 fimbrial protein, A chain | Escherichia coli 1-110-08_S4_C2 | 354.4 | 5.9E-103 |  |
| 65 | AC65_3931 | KDW72387 | type-1 fimbrial protein, A chain | Escherichia coli 2-005-03_S4_C1 | 354.4 | 5.9E-103 |  |
| 66 | AB73_4983 | KDZ27091 | type-1 fimbrial protein, A chain | Escherichia coli 3-020-07_S1_C3 | 354.4 | 5.9E-103 |  |
| 67 | AC31_3572 | KDZ63212 | type-1 fimbrial protein, A chain | Escherichia coli 3-073-06_S3_C2 | 354.4 | 5.9E-103 |  |
| 68 | AD02_4539 | KDY64212 | type-1 fimbrial protein, A chain | Escherichia coli 2-460-02_S4_C2 | 354.4 | 5.9E-103 |  |
| 69 | G866_03444 | ETF28066 | type-1 fimbrial protein, A chain | Escherichia coli HVH 214 (4-3062198) | 354.4 | 5.9E-103 |  |
| 70 | ERGG_03311 | EGB55823 | fimbrial protein | Escherichia coli H489 | 354.4 | 5.9E-103 |  |
| 71 | ECLG_03391 | EGI38215 | type 1 fimbriae major subunit FimA | Escherichia coli TA271 | 354.4 | 5.9E-103 |  |
| 72 | AB35_4799 | KDY98598 | type-1 fimbrial protein, A chain | Escherichia coli 2-474-04_S1_C2 | 354.4 | 5.9E-103 |  |
| 73 | G937_04520 | EQX79089 | type-1 fimbrial protein, A chain | Escherichia coli UMEA 3199-1 | 354.4 | 5.9E-103 |  |
| 74 | ECDEC6B_5543 | EHV49579 | fimbrial subunit type 1 | Escherichia coli DEC6B | 354.4 | 5.9E-103 |  |
| 75 | AC53_4779 | KEM96799 | type-1 fimbrial protein, A chain | Escherichia coli 7-233-03_S3_C3 | 354.4 | 5.9E-103 |  |
| 76 | AC50_3938 | KDZ14049 | type-1 fimbrial protein, A chain | Escherichia coli 2-474-04_S3_C3 | 354.4 | 5.9E-103 |  |
| 77 | AD29_4699 | KEO10694 | type-1 fimbrial protein, A chain | Escherichia coli 2-222-05_S4_C3 | 354.4 | 5.9E-103 |  |
| 78 | AD10_4912 | EZJ36502 | type-1 fimbrial protein, A chain | Escherichia coli 1-182-04_S4_C2 | 354.4 | 5.9E-103 |  |
| 79 | AC21_4526 | KDY88118 | type-1 fimbrial protein, A chain | Escherichia coli 2-474-04_S3_C2 | 354.4 | 5.9E-103 |  |
| 80 | AD46_4717 | KEM44896 | type-1 fimbrial protein, A chain | Escherichia coli 6-175-07_S4_C3 | 354.4 | 5.9E-103 |  |
| 81 | AC25_5204 | EYE15526 | type-1 fimbrial protein, A chain | Escherichia coli 1-110-08_S3_C2 | 354.4 | 5.9E-103 |  |
| 82 | AC02_3803 | KDZ36719 | type-1 fimbrial protein, A chain | Escherichia coli 3-020-07_S3_C1 | 354.4 | 5.9E-103 |  |
| 83 | G880_04827 | ERA98246 | type-1 fimbrial protein, A chain | Escherichia coli KOEGE 10 (25a) | 354.4 | 5.9E-103 |  |
| 84 | AC12_5295 | KDA70819 | type-1 fimbrial protein, A chain | Escherichia coli 2-005-03_S3_C2 | 354.4 | 5.9E-103 |  |
| 85 | AD03_3637 | KDZ04736 | type-1 fimbrial protein, A chain | Escherichia coli 2-474-04_S4_C2 | 354.4 | 5.9E-103 |  |
| 86 | AD42_4132 | KDZ79729 | type-1 fimbrial protein, A chain | Escherichia coli 3-073-06_S4_C3 | 354.4 | 5.9E-103 |  |
| 87 | AD00_5473 | KDY08908 | type-1 fimbrial protein, A chain | Escherichia coli 2-316-03_S4_C2 | 354.4 | 5.9E-103 |  |
| 88 | L476_04471 | ESL16953 | type-1 fimbrial protein, A chain | Escherichia coli BIDMC 39 | 354.4 | 5.9E-103 |  |
| 89 | AB98_1822 | EYD90126 | type-1 fimbrial protein, A chain | Escherichia coli 1-176-05_S3_C1 | 354.4 | 5.9E-103 |  |
| 90 | G829_04839 | EQT17397 | type-1 fimbrial protein, A chain | Escherichia coli HVH 175 (4-3405184) | 354.4 | 5.9E-103 |  |
| 91 | AD33_3422 | KDZ11538 | type-1 fimbrial protein, A chain | Escherichia coli 2-474-04_S4_C3 | 354.4 | 5.9E-103 |  |
| 92 | C4390_42460 | EMS03614 | major type 1 subunit fimbrin (pilin) | Escherichia coli O127:H27 str. C43/90 | 354.4 | 5.9E-103 |  |
| 93 | ECRM12761_25690 | AHY68175 | type 1 fimbriae major subunit FimA | Escherichia coli O145:H28 str. RM12761 | 354.4 | 5.9E-103 |  |
| 94 | AC99_2570 | KDX91042 | type-1 fimbrial protein, A chain | Escherichia coli 2-222-05_S4_C2 | 354.4 | 5.9E-103 | Yes |
| 95 | ESBG_03747 | EIG72442 | type-1 fimbrial protein, A chain | Escherichia sp. 4_1_40B | 354.4 | 5.9E-103 |  |
| 96 | WIO_04635 | ELI49035 | fimbrial protein FimA | Escherichia coli KTE125 | 354.4 | 5.9E-103 |  |
| 97 | ECP030229310_4577 | ENE16433 | type-1 fimbrial protein, A chain | Escherichia coli P0302293.10 | 354.4 | 5.9E-103 |  |
| 98 | AD39_4948 | EZJ13419 | type-1 fimbrial protein, A chain | Escherichia coli 1-182-04_S4_C3 | 354.4 | 5.9E-103 |  |
| 99 | G718_04591 | EQO57407 | type-1 fimbrial protein, A chain | Escherichia coli HVH 43 (4-2173468) | 354.4 | 5.9E-103 |  |
| 100 | AC30_4904 | KEJ64133 | type-1 fimbrial protein, A chain | Escherichia coli 3-020-07_S3_C2 | 354.4 | 5.9E-103 |  |
| 101 | AC52_4855 | KEL70456 | type-1 fimbrial protein, A chain | Escherichia coli 5-366-08_S3_C3 | 354.4 | 5.9E-103 |  |
| 102 | WCK_00543 | ELF59700 | fimbrial protein FimA | Escherichia coli KTE9 | 354.4 | 5.9E-103 |  |
| 103 | CFSAN002236_23550 | ERF86995 | type-1 fimbrial protein subunit A | Escherichia coli O104:H21 str. CFSAN002236 | 354.4 | 5.9E-103 |  |
| 104 | ERS085382_04337 | CUA51271 | type 1 fimbriae major subunit FimA | Escherichia coli O45:K1 (strain S88 / ExPEC) | 354.4 | 5.9E-103 |  |
| 105 | G916_04581 | ERB08819 | type-1 fimbrial protein, A chain | Escherichia coli UMEA 3144-1 | 354.4 | 5.9E-103 |  |
| 106 | AB68_4703 | KEN06889 | type-1 fimbrial protein, A chain | Escherichia coli 7-233-03_S1_C3 | 354.4 | 5.9E-103 |  |
| 107 | A1US_00271 | ELG30549 | fimbrial protein FimA | Escherichia coli KTE78 | 354.4 | 5.9E-103 | Yes |
| 108 | ECP03022936_4845 | ENE32373 | type-1 fimbrial protein, A chain | Escherichia coli P0302293.6 | 354.4 | 5.9E-103 |  |
| 109 | BY39_21610 | EZA65548 | type-1 fimbrial protein subunit A | Escherichia coli O104:H21 str. 94-3025 | 354.4 | 5.9E-103 |  |
| 110 | ECDEC6A_5165 | EHV50357 | type-1 fimbrial protein, A chain | Escherichia coli DEC6A | 354.4 | 5.9E-103 |  |
| 111 | ECP03022933_4784 | ENE18960 | type-1 fimbrial protein, A chain | Escherichia coli P0302293.3 | 354.4 | 5.9E-103 |  |
| 112 | AD38_4816 | EZJ15087 | type-1 fimbrial protein, A chain | Escherichia coli 1-176-05_S4_C3 | 354.4 | 5.9E-103 |  |
| 113 | AC87_4625 | KDT89893 | type-1 fimbrial protein, A chain | Escherichia coli 3-105-05_S4_C1 | 354.4 | 5.9E-103 | Yes |
| 114 | A1YU_04044 | ELF07183 | type-1 fimbrial protein, A chain | Escherichia coli KTE142 | 354.4 | 5.9E-103 |  |
| 115 | A1S9_01533 | ELG08171 | fimbrial protein FimA | Escherichia coli KTE50 | 354.4 | 5.9E-103 |  |
| 116 | ECP03022938_4892 | ENE36845 | type-1 fimbrial protein, A chain | Escherichia coli P0302293.8 | 354.4 | 5.9E-103 |  |
| 117 | AD20_4584 | KEL99558 | type-1 fimbrial protein, A chain | Escherichia coli 6-175-07_S4_C2 | 354.4 | 5.9E-103 |  |
| 118 | L960_2487c | AIF62310 | type-1 fimbrial-like protein | Escherichia coli B7A | 354.4 | 5.9E-103 |  |
| 119 | ECKD2_14397 | EIL50000 | major type 1 subunit fimbrin (pilin) | Escherichia coli KD2 | 354.4 | 5.9E-103 |  |
| 120 | AC81_4829 | EZJ65086 | type-1 fimbrial protein, A chain | Escherichia coli 1-176-05_S4_C1 | 354.4 | 5.9E-103 |  |
| 121 | AD32_4450 | KDY73091 | type-1 fimbrial protein, A chain | Escherichia coli 2-460-02_S4_C3 | 354.4 | 5.9E-103 |  |
| 122 | AB83_4913 | KDS94705 | type-1 fimbrial protein, A chain | Escherichia coli 2-011-08_S3_C1 | 354.4 | 5.9E-103 |  |
| 123 | AC28_4932 | KEO24626 | type-1 fimbrial protein, A chain | Escherichia coli 1-250-04_S3_C2 | 354.4 | 5.9E-103 |  |
| 124 | AD09_4734 | KDA87315 | type-1 fimbrial protein, A chain | Escherichia coli 1-176-05_S4_C2 | 354.4 | 5.9E-103 |  |
| 125 | AB95_5492 | KEM80896 | type-1 fimbrial protein, A chain | Escherichia coli 7-233-03_S3_C1 | 354.4 | 5.9E-103 |  |
| 126 | AB94_4540 | KEL89024 | type-1 fimbrial protein, A chain | Escherichia coli 5-366-08_S3_C1 | 354.4 | 5.9E-103 |  |
| 127 | AB15_4929 | KDZ16987 | type-1 fimbrial protein, A chain | Escherichia coli 3-020-07_S1_C1 | 354.4 | 5.9E-103 |  |
| 128 | WC3_00367 | EOU56822 | fimbrial protein FimA | Escherichia coli KTE35 | 354.4 | 5.9E-103 |  |
| 129 | G794_04668 | ESP12241 | type-1 fimbrial protein, A chain | Escherichia coli HVH 136 (4-5970458) | 354.4 | 5.9E-103 |  |
| 130 | AC05_4621 | KDT58479 | type-1 fimbrial protein, A chain | Escherichia coli 3-267-03_S3_C1 | 354.4 | 5.9E-103 |  |
| 131 | AB36_4712 | KEM56773 | type-1 fimbrial protein, A chain | Escherichia coli 7-233-03_S1_C2 | 354.4 | 5.9E-103 |  |
| 132 | G872_04321 | EQV01909 | type-1 fimbrial protein, A chain | Escherichia coli HVH 221 (4-3136817) | 354.4 | 5.9E-103 |  |
| 133 | CFSAN002237_19485 | ERF89646 | type-1 fimbrial protein subunit A | Escherichia coli O104:H21 str. CFSAN002237 | 354.4 | 5.9E-103 |  |
| 134 | AB03_5240 | KEJ20457 | type-1 fimbrial protein, A chain | Escherichia coli 2-316-03_S1_C1 | 354.4 | 5.9E-103 |  |
| 135 | AC00_4896 | EZJ80864 | type-1 fimbrial protein, A chain | Escherichia coli 1-250-04_S3_C1 | 354.4 | 5.9E-103 |  |
| 136 | AC41_4725 | KDA82250 | type-1 fimbrial protein, A chain | Escherichia coli 2-011-08_S3_C3 | 354.4 | 5.9E-103 |  |
| 137 | A1UU_01765 | ELG33919 | fimbrial protein FimA | Escherichia coli KTE79 | 354.4 | 5.9E-103 |  |
| 138 | G986_04483 | EQZ59802 | type-1 fimbrial protein, A chain | Escherichia coli UMEA 3682-1 | 354.4 | 5.9E-103 |  |
| 139 | AD22_4836 | KEN55656 | type-1 fimbrial protein, A chain | Escherichia coli 6-537-08_S4_C2 | 354.4 | 5.9E-103 |  |
| 140 | ECS88_4932 | CAR06076 | major type 1 subunit fimbrin (pilin) | Escherichia coli S88 | 354.4 | 5.9E-103 | Yes |
| 141 | AB43_5001 | KDZ18292 | type-1 fimbrial protein, A chain | Escherichia coli 3-020-07_S1_C2 | 354.4 | 5.9E-103 |  |
| 142 | AC74_5115 | KEJ45465 | type-1 fimbrial protein, A chain | Escherichia coli 2-460-02_S4_C1 | 354.4 | 5.9E-103 |  |
| 143 | G925_04666 | EQX22123 | type-1 fimbrial protein, A chain | Escherichia coli (strain UMEA 3162-1) | 354.4 | 5.9E-103 |  |
| 144 | ECP03022932_4998 | EMX08789 | type-1 fimbrial protein, A chain | Escherichia coli P0302293.2 | 354.4 | 5.9E-103 |  |
| 145 | EC2732_17036 | KRR51301 | major type 1 subunit fimbrin (pilin) | Escherichia coli VL2732 | 354.4 | 5.9E-103 |  |
| 146 | AC04_4512 | KDT32987 | type-1 fimbrial protein, A chain | Escherichia coli 3-105-05_S3_C1 | 354.4 | 5.9E-103 |  |
| 147 | A31G_01924 | ELF28447 | type-1 fimbrial protein, A chain | Escherichia coli KTE161 | 354.4 | 5.9E-103 |  |
| 148 | ESMG_00467 | EIF87160 | type-1 fimbrial protein, A chain | Escherichia coli M919 | 354.4 | 5.9E-103 |  |
| 149 | BX09_24205 | EZD97575 | type-1 fimbrial protein subunit A | Escherichia coli O145:H28 str. 2009C-3292 | 354.4 | 5.9E-103 |  |
| 150 | G917_04571 | ESP39527 | type-1 fimbrial protein, A chain | Escherichia coli UMEA 3148-1 | 354.4 | 5.9E-103 |  |
| 151 | AC92_4724 | KEM87125 | type-1 fimbrial protein, A chain | Escherichia coli 6-537-08_S4_C1 | 354.4 | 5.9E-103 |  |
| 152 | AC86_4262 | KDU34566 | type-1 fimbrial protein, A chain | Escherichia coli 3-073-06_S4_C1 | 354.4 | 5.9E-103 |  |
| 153 | AC23_4945 | KEN17976 | type-1 fimbrial protein, A chain | Escherichia coli 7-233-03_S3_C2 | 354.4 | 5.9E-103 |  |
| 154 | A15E_00391 | ELH63205 | fimbrial protein FimA | Escherichia coli KTE202 | 354.4 | 5.9E-103 |  |
| 155 | G825_04861 | EQS96608 | type-1 fimbrial protein, A chain | Escherichia coli HVH 170 (4-3026949) | 354.4 | 5.9E-103 |  |
| 156 | ECRM13516_5272 | AHG17853 | type 1 fimbriae major subunit FimA | Escherichia coli O145:H28 str. RM13516 | 354.4 | 5.9E-103 | Yes |
| 157 | G896_04546 | EQV96069 | type-1 fimbrial protein, A chain | Escherichia coli KOEGE 118 (317a) | 354.4 | 5.9E-103 |  |
| 158 | AB97_0052 | EYE30784 | type-1 fimbrial protein, A chain | Escherichia coli 1-110-08_S3_C1 | 354.4 | 5.9E-103 |  |
| 159 | BU56_08200 | KDV36607 | type-1 fimbrial protein subunit A | Escherichia coli O145:H25 str. 07-3858 | 354.4 | 5.9E-103 |  |
| 160 | AC03_3859 | KDZ61416 | type-1 fimbrial protein, A chain | Escherichia coli 3-073-06_S3_C1 | 354.4 | 5.9E-103 |  |
| 161 | AB48_3880 | KEK80757 | type-1 fimbrial protein, A chain | Escherichia coli 3-475-03_S1_C2 | 354.4 | 5.9E-103 |  |
| 162 | G998_04268 | ERA13129 | type-1 fimbrial protein, A chain | Escherichia coli UMEA 3889-1 | 354.4 | 5.9E-103 |  |
| 163 | G834_04636 | EQT31754 | type-1 fimbrial protein, A chain | Escherichia coli HVH 182 (4-0985554) | 354.4 | 5.9E-103 |  |
| 164 | G933_04560 | EQX63263 | type-1 fimbrial protein, A chain | Escherichia coli UMEA 3180-1 | 354.4 | 5.9E-103 |  |
| 165 | EC970246_4018 | EIG92904 | type-1 fimbrial protein, A chain | Escherichia coli 97.0246 | 354.4 | 5.9E-103 |  |
| 166 | AC36_4330 | KEL07192 | type-1 fimbrial protein, A chain | Escherichia coli 4-203-08_S3_C2 | 354.4 | 5.9E-103 |  |
| 167 | G843_04679 | EQT72235 | type-1 fimbrial protein, A chain | Escherichia coli HVH 191 (3-9341900) | 354.4 | 5.9E-103 |  |
| 168 | AC82_4861 | EZJ57569 | type-1 fimbrial protein, A chain | Escherichia coli 1-182-04_S4_C1 | 354.4 | 5.9E-103 |  |
| 169 | G849_04767 | EQU07266 | type-1 fimbrial protein, A chain | Escherichia coli HVH 197 (4-4466217) | 354.4 | 5.9E-103 |  |
| 170 | AC13_4744 | KDA76988 | type-1 fimbrial protein, A chain | Escherichia coli 2-011-08_S3_C2 | 354.4 | 5.9E-103 |  |
| 171 | AB32_5448 | KEJ35622 | type-1 fimbrial protein, A chain | Escherichia coli 2-316-03_S1_C2 | 354.4 | 5.9E-103 |  |
| 172 | AC75_4753 | KEN81963 | type-1 fimbrial protein, A chain | Escherichia coli 2-474-04_S4_C1 | 354.4 | 5.9E-103 |  |
| 173 | G869_04793 | EQU87568 | type-1 fimbrial protein, A chain | Escherichia coli HVH 217 (4-1022806) | 354.4 | 5.9E-103 |  |
| 174 | AB92_3661 | KDY85714 | type-1 fimbrial protein, A chain | Escherichia coli 2-474-04_S3_C1 | 354.4 | 5.9E-103 |  |
| 175 | AC91_4607 | KEM00767 | type-1 fimbrial protein, A chain | Escherichia coli 6-175-07_S4_C1 | 354.4 | 5.9E-103 |  |
| 176 | HMPREF1611_03511 | ESD82371 | type-1 fimbrial protein, A chain | Escherichia coli 908573 | 354.4 | 5.9E-103 |  |
| 177 | AC61_4777 | KEK90365 | type-1 fimbrial protein, A chain | Escherichia coli 4-203-08_S3_C3 | 354.4 | 5.9E-103 |  |
| 178 | WEY_00371 | EOV00577 | fimbrial protein FimA | Escherichia coli KTE34 | 354.4 | 5.9E-103 |  |
| 179 | WCS_04858 | EOU53463 | fimbrial protein FimA | Escherichia coli KTE14 | 354.4 | 5.9E-103 |  |
| 180 | G999_04652 | ERA14020 | type-1 fimbrial protein, A chain | Escherichia coli UMEA 3893-1 | 354.4 | 5.9E-103 |  |
| 181 | G753_04353 | EQQ13615 | type-1 fimbrial protein, A chain | Escherichia coli HVH 91 (4-4638751) | 354.4 | 5.9E-103 |  |
| 182 | GECO_04396 | KFB91652 | type 1 fimbriae major subunit | Escherichia coli DSM 30083 = JCM 1649 = ATCC 11775 | 354.4 | 5.9E-103 |  |
| 183 | WCE_04755 | ELC04837 | type-1 fimbrial protein, A chain | Escherichia coli KTE5 | 354.4 | 5.9E-103 |  |
| 184 | WAW_00434 | EOU39654 | fimbrial protein FimA | Escherichia coli KTE7 | 354.4 | 5.9E-103 |  |
| 185 | HMPREF9534_02254 | EFJ81711 | fimbrial protein | Escherichia coli MS 69-1 | 354.4 | 5.9E-103 | Yes |
| 186 | ECP03022937_4839 | END92773 | type-1 fimbrial protein, A chain | Escherichia coli P0302293.7 | 354.4 | 5.9E-103 |  |
| 187 | AC32_4509 | KDT44934 | type-1 fimbrial protein, A chain | Escherichia coli 3-105-05_S3_C2 | 354.4 | 5.9E-103 |  |
| 188 | AC55_0040 | EYE15614 | type-1 fimbrial protein, A chain | Escherichia coli 1-110-08_S3_C3 | 354.4 | 5.9E-103 |  |
| 189 | ECA0157_08069 | EST86874 | major type 1 subunit fimbrin (pilin) | Escherichia coli ECA-0157 | 354.1 | 7.3E-103 | Yes |
| 190 | EcE110019_2185 | EDV86998 | type-1 fimbrial protein homolog | Escherichia coli E110019 | 353.2 | 1.4E-102 |  |
| 191 | ERS085405_02503 | CTV95939 | type 1 fimbriae major subunit FimA | Escherichia coli (GCA_001285485) | 353.2 | 1.4E-102 |  |
| 192 | P411_21165 | EZD87851 | type-1 fimbrial protein subunit A | Escherichia coli O39:NM str. F8704-2 | 353.2 | 1.4E-102 |  |
| 193 | AB44_4700 | KDZ57185 | type-1 fimbrial protein, A chain | Escherichia coli 3-073-06_S1_C2 | 352.9 | 1.8E-102 | Yes |
| 194 | AD34_5041 | KEL56186 | type-1 fimbrial protein, A chain | Escherichia coli 5-172-05_S4_C3 | 352.9 | 1.8E-102 |  |
| 195 | AC76_5098 | KEL33102 | type-1 fimbrial protein, A chain | Escherichia coli 5-172-05_S4_C1 | 352.9 | 1.8E-102 |  |
| 196 | AD04_4700 | KEL22182 | type-1 fimbrial protein, A chain | Escherichia coli 5-172-05_S4_C2 | 352.9 | 1.8E-102 |  |
| 197 | ECP03022939_4794 | ENE44997 | type-1 fimbrial protein, A chain | Escherichia coli P0302293.9 | 352.5 | 2.3E-102 |  |
| 198 | ECP03022934_5016 | ENE25984 | type-1 fimbrial protein, A chain | Escherichia coli P0302293.4 | 352.5 | 2.3E-102 | Yes |
| 199 | WIG_04406 | ELI23391 | fimbrial protein FimA | Escherichia coli KTE117 | 351.7 | 4.2E-102 |  |
| 200 | AB02_4574 | KDX64025 | type-1 fimbrial protein, A chain | Escherichia coli 2-222-05_S1_C1 | 351.7 | 4.2E-102 |  |
| 201 | AB31_4285 | KDX73009 | type-1 fimbrial protein, A chain | Escherichia coli 2-222-05_S1_C2 | 351.7 | 4.2E-102 |  |
| 202 | A153_00609 | ELH45192 | fimbrial protein FimA | Escherichia coli KTE196 | 351.7 | 4.2E-102 |  |
| 203 | XB00_25630 | KLD43776 | type-1 fimbrial protein subunit A | Escherichia coli (GCA_001010165) | 351.7 | 4.2E-102 |  |
| 204 | AB63_4284 | KDX77098 | type-1 fimbrial protein, A chain | Escherichia coli 2-222-05_S1_C3 | 351.7 | 4.2E-102 |  |
| 205 | AC90_4523 | KDT81423 | type-1 fimbrial protein, A chain | Escherichia coli 3-475-03_S4_C1 | 351.5 | 4.7E-102 | Yes |
| 206 | G697_04681 | EQN69711 | type-1 fimbrial protein, A chain | Escherichia coli HVH 21 (4-4517873) | 351.5 | 4.9E-102 | Yes |
| 207 | AB86_4035 | KDW13327 | type-1 fimbrial protein, A chain | Escherichia coli 2-177-06_S3_C1 | 350.5 | 9.7E-102 |  |
| 208 | BX68_04260 | EYZ00078 | type-1 fimbrial protein subunit A | Escherichia coli O177:NM str. 2010C-4558 | 350.5 | 9.7E-102 |  |
| 209 | A17G_00026 | EOV45858 | fimbrial protein FimA | Escherichia coli KTE221 | 350.5 | 9.7E-102 |  |
| 210 | G897_04343 | EQW07885 | type-1 fimbrial protein, A chain | Escherichia coli KOEGE 131 (358a) | 350.5 | 9.7E-102 |  |
| 211 | WKG_04662 | ELJ07778 | fimbrial protein FimA | Escherichia coli KTE163 | 350.5 | 9.7E-102 | Yes |
| 212 | EcHS_A4540 | ABV08693 | type-1 fimbrial protein homolog | Escherichia coli HS | 350.4 | 1E-101 | Yes |
| 213 | ECP02994384_5203 | ENB95394 | type-1 fimbrial protein, A chain | Escherichia coli P0299438.4 | 348.9 | 2.9E-101 |  |
| 214 | A1U1_04594 | EOV52255 | fimbrial protein FimA | Escherichia coli KTE64 | 348.9 | 2.9E-101 |  |
| 215 | ECDEC12E_0025 | EHX54706 | fimbrial subunit type 1 | Escherichia coli DEC12E | 348.9 | 2.9E-101 |  |
| 216 | BY43_15450 | EZA75358 | type-1 fimbrial protein subunit A | Escherichia coli O25:NM str. E2539C1 | 348.9 | 2.9E-101 |  |
| 217 | ECEPECA12_5168 | EIQ57951 | type-1 fimbrial protein, A chain | Escherichia coli EPECa12 | 348.9 | 2.9E-101 |  |
| 218 | ECDEC7D_0091 | EHV96580 | fimbrial subunit type 1 | Escherichia coli DEC7D | 348.9 | 2.9E-101 |  |
| 219 | AB87_4626 | KDX50849 | type-1 fimbrial protein, A chain | Escherichia coli 2-210-07_S3_C1 | 348.9 | 2.9E-101 |  |
| 220 | ECTX1999_5174 | EGX18414 | type-1 fimbrial protein, A chain | Escherichia coli TX1999 | 348.9 | 2.9E-101 |  |
| 221 | A193_00579 | ELD74367 | type-1 fimbrial protein, A chain | Escherichia coli KTE234 | 348.9 | 2.9E-101 |  |
| 222 | ECP029943810_4839 | ENB84226 | type-1 fimbrial protein, A chain | Escherichia coli P0299438.10 | 348.9 | 2.9E-101 |  |
| 223 | EC2016001_0328 | ENA25688 | type-1 fimbrial protein, A chain | Escherichia coli 201600.1 | 348.9 | 2.9E-101 |  |
| 224 | WG3_00192 | EOU96439 | fimbrial protein FimA | Escherichia coli KTE36 | 348.9 | 2.9E-101 |  |
| 225 | ECDEC7A_0001 | EHV81987 | type-1 fimbrial protein, A chain | Escherichia coli DEC7A | 348.9 | 2.9E-101 |  |
| 226 | EC75_04586 | EIL70469 | major type 1 subunit fimbrin (pilin) | Escherichia coli 75 | 348.9 | 2.9E-101 |  |
| 227 | G943_04909 | EQY08751 | type-1 fimbrial protein, A chain | Escherichia coli UMEA 3212-1 | 348.9 | 2.9E-101 | Yes |
| 228 | L474_04412 | ESL31362 | type-1 fimbrial protein, A chain | Escherichia coli BIDMC 37 | 348.9 | 2.9E-101 |  |
| 229 | ECA727_15865 | EST77924 | major type 1 subunit fimbrin (pilin) | Escherichia coli ECA-727 | 348.9 | 2.9E-101 |  |
| 230 | EC180050_0296 | EMW90808 | type-1 fimbrial protein, A chain | Escherichia coli 180050 | 348.9 | 2.9E-101 |  |
| 231 | WGE_00627 | ELF78989 | fimbrial protein FimA | Escherichia coli KTE42 | 348.9 | 2.9E-101 |  |
| 232 | ECDEC12D_5610 | EHX38154 | fimbrial subunit type 1 | Escherichia coli DEC12D | 348.9 | 2.9E-101 |  |
| 233 | ECDEC7C_0026 | EHV91089 | fimbrial subunit type 1 | Escherichia coli DEC7C | 348.9 | 2.9E-101 |  |
| 234 | PCN061_4499 | AKM37930 | major type 1 subunit fimbrin (pilin) | Escherichia coli PCN061 | 348.9 | 2.9E-101 | Yes |
| 235 | ECB41_4811 | EIJ01902 | type-1 fimbrial protein, A chain | Escherichia coli B41 | 348.9 | 2.9E-101 |  |
| 236 | EcB171_2741 | EDX31678 | type-1 fimbrial protein homolog | Escherichia coli B171 | 348.9 | 2.9E-101 |  |
| 237 | ECDEC12C_5341 | EHX24081 | type-1 fimbrial protein, A chain | Escherichia coli DEC12C | 348.9 | 2.9E-101 |  |
| 238 | ECDEC12B_0033 | EHX37420 | fimbrial subunit type 1 | Escherichia coli DEC12B | 348.9 | 2.9E-101 |  |
| 239 | ECG581_0035 | EGX13862 | type-1 fimbrial protein, A chain | Escherichia coli G58-1 | 348.9 | 2.9E-101 |  |
| 240 | EC2762100_5077 | EMW48601 | type-1 fimbrial protein, A chain | Escherichia coli 2762100 | 348.9 | 2.9E-101 | Yes |
| 241 | ECDEC7E_4977 | EHV94338 | type-1 fimbrial protein, A chain | Escherichia coli DEC7E | 348.9 | 2.9E-101 |  |
| 242 | ERDG_03721 | EGB35885 | fimbrial protein | Escherichia coli E482 | 348.9 | 2.9E-101 |  |
| 243 | AC73_5275 | KDY38321 | type-1 fimbrial protein, A chain | Escherichia coli 2-427-07_S4_C1 | 348.9 | 2.9E-101 |  |
| 244 | HMPREF9530_03170 | EFK20224 | fimbrial protein | Escherichia coli MS 21-1 | 348.9 | 2.9E-101 | Yes |
| 245 | ERS085440_03834 | CTS97754 | type 1 fimbriae major subunit FimA | Escherichia coli | 348.9 | 2.9E-101 | Yes |
| 246 | ECDEC12A_0032 | EHX37052 | type-1 fimbrial protein, A chain | Escherichia coli DEC12A | 348.9 | 2.9E-101 |  |
| 247 | A1WK_00577 | EOW12356 | fimbrial protein FimA | Escherichia coli KTE100 | 348.9 | 2.9E-101 |  |
| 248 | EC2731150_4984 | EMW70551 | type-1 fimbrial protein, A chain | Escherichia coli 2731150 | 348.9 | 2.9E-101 |  |
| 249 | ECE128010_0495 | EFZ49142 | type-1 fimbrial protein, A chain | Escherichia coli E128010 | 348.9 | 2.9E-101 |  |
| 250 | ECEPECC34262_0034 | EIQ72989 | fimbrial subunit type 1 | Escherichia coli EPEC C342-62 | 347.3 | 9.6E-101 | Yes |
| 251 | AC87_4627 | KDT89724 | type-1 fimbrial protein, A chain | Escherichia coli 3-105-05_S4_C1 | 341.5 | 5.5E-99 |  |
| 252 | ECKG_03433 | EGI24014 | type 1 fimbriae major subunit FimA | Escherichia coli TA206 | 341.4 | 6.1E-99 | Yes |
| 253 | G846_04696 | EQT91084 | type-1 fimbrial protein, A chain | Escherichia coli HVH 194 (4-2356805) | 341.4 | 6.1E-99 |  |
| 254 | G816_04471 | EQS57099 | type-1 fimbrial protein, A chain | Escherichia coli HVH 158 (4-3224287) | 341.4 | 6.1E-99 |  |
| 255 | EAKF1_ch1539c | AHE59428 | type 1 fimbriae major subunit FimA | Escherichia albertii KF1 | 341.3 | 6.5E-99 | Yes |
| 256 | A1SC_03641 | EOV45332 | fimbrial protein FimA | Escherichia sp. KTE52 | 340.1 | 1.6E-98 | Yes |
| 257 | AB44_5539 | KDZ48586 | type-1 fimbrial protein, A chain | Escherichia coli 3-073-06_S1_C2 | 340 | 1.6E-98 |  |
| 258 | AD04_3749 | KEL24711 | type-1 fimbrial protein, A chain | Escherichia coli 5-172-05_S4_C2 | 340 | 1.6E-98 |  |
| 259 | WCO_03930 | ELC14604 | type-1 fimbrial protein, A chain | Escherichia sp. KTE11 | 339.2 | 3E-98 | Yes |
| 260 | A1WG_02405 | EOV91506 | fimbrial protein FimA | Escherichia sp. KTE96 | 339.2 | 3E-98 |  |
| 261 | A31E_04154 | EOW56825 | fimbrial protein FimA | Escherichia sp. KTE159 | 339.2 | 3E-98 |  |
| 262 | AC41_4722 | KDA82251 | type-1 fimbrial protein, A chain | Escherichia coli 2-011-08_S3_C3 | 338.4 | 4.9E-98 |  |
| 263 | A1UM_00140 | ELE59496 | type-1 fimbrial protein, A chain | Escherichia coli KTE75 | 336.3 | 2.3E-97 | Yes |
| 264 | BW79_12295 | EYZ99696 | type-1 fimbrial protein subunit A | Escherichia coli O119:H4 str. 03-3458 | 335 | 5.8E-97 |  |
| 265 | CDK86539 | CDK86539 | type 1 fimbriae major subunit FimA | Escherichia coli IS29 | 335 | 5.8E-97 |  |
| 266 | BU64_06085 | KDV62136 | type-1 fimbrial protein subunit A | Escherichia coli O128:H2 str. 2011C-3317 | 335 | 5.8E-97 |  |
| 267 | AC98_4741 | KDX54073 | type-1 fimbrial protein, A chain | Escherichia coli 2-210-07_S4_C2 | 335 | 5.8E-97 |  |
| 268 | ECP03048168_5072 | ENF63268 | type-1 fimbrial protein, A chain | Escherichia coli P0304816.8 | 335 | 5.8E-97 |  |
| 269 | BX77_23255 | EYY68574 | type-1 fimbrial protein subunit A | Escherichia coli O111:NM str. 2010C-4818 | 335 | 5.8E-97 |  |
| 270 | A1SW_00443 | ELE36891 | fimbrial subunit type 1 | Escherichia coli KTE62 | 335 | 5.8E-97 |  |
| 271 | i01_06266 | EHF98107 | major type 1 subunit fimbrin (pilin) | Escherichia coli cloneA_i1 | 335 | 5.8E-97 |  |
| 272 | ECP030481613_4830 | ENF35147 | type-1 fimbrial protein, A chain | Escherichia coli P0304816.13 | 335 | 5.8E-97 |  |
| 273 | G800_04593 | EQS11022 | fimbrial subunit type 1 | Escherichia coli HVH 142 (4-5627451) | 335 | 5.8E-97 |  |
| 274 | WKQ_00003 | ELJ45705 | fimbrial protein FimA | Escherichia coli KTE174 | 335 | 5.8E-97 |  |
| 275 | BX60_18900 | EYU74152 | type-1 fimbrial protein subunit A | Escherichia coli O111:NM str. 2010C-4221 | 335 | 5.8E-97 |  |
| 276 | EC01288_4719 | EKJ54222 | type-1 fimbrial protein, A chain | Escherichia coli 0.1288 | 335 | 5.8E-97 |  |
| 277 | AC95_4858 | KDV78000 | type-1 fimbrial protein, A chain | Escherichia coli 2-052-05_S4_C2 | 335 | 5.8E-97 |  |
| 278 | ECAI27_01270 | EIE57781 | Fimbrial protein | Escherichia coli AI27 | 335 | 5.8E-97 |  |
| 279 | L455_09099 | ETX90596 | fimbrial subunit type 1 | Escherichia coli BIDMC 20A | 335 | 5.8E-97 |  |
| 280 | G830_04538 | EQT17807 | fimbrial subunit type 1 | Escherichia coli HVH 176 (4-3428664) | 335 | 5.8E-97 |  |
| 281 | G722_04492 | EQO79515 | fimbrial subunit type 1 | Escherichia coli HVH 48 (4-2658593) | 335 | 5.8E-97 |  |
| 282 | L475_04775 | ESL31875 | fimbrial subunit type 1 | Escherichia coli BIDMC 38 | 335 | 5.8E-97 |  |
| 283 | ECDEC13D_4974 | EHX56679 | fimbrial subunit type 1 | Escherichia coli DEC13D | 335 | 5.8E-97 |  |
| 284 | ESQG_01459 | EHN86481 | fimbrial subunit type 1 | Escherichia coli H494 | 335 | 5.8E-97 |  |
| 285 | AF25_04776 | KDG48315 | fimbrial subunit type 1 | Escherichia coli CHS 69 | 335 | 5.8E-97 |  |
| 286 | WKE_00004 | ELJ18353 | fimbrial protein FimA | Escherichia coli KTE160 | 335 | 5.8E-97 |  |
| 287 | ECP02994833_4741 | END76828 | type-1 fimbrial protein, A chain | Escherichia coli P0299483.3 | 335 | 5.8E-97 |  |
| 288 | A177_00295 | ELD45792 | fimbrial subunit type 1 | Escherichia coli KTE216 | 335 | 5.8E-97 |  |
| 289 | ECP03048164_4859 | ENH27467 | type-1 fimbrial protein, A chain | Escherichia coli P0304816.4 | 335 | 5.8E-97 |  |
| 290 | AB65_5214 | KEJ36638 | type-1 fimbrial protein, A chain | Escherichia coli 2-460-02_S1_C3 | 335 | 5.8E-97 |  |
| 291 | AE39_03810 | KDF87819 | fimbrial subunit type 1 | Escherichia coli BIDMC 64 | 335 | 5.8E-97 |  |
| 292 | EC970259_5201 | EIH46533 | type-1 fimbrial protein, A chain | Escherichia coli 99.0741 | 335 | 5.8E-97 |  |
| 293 | ECP03048165_4870 | ENH33702 | type-1 fimbrial protein, A chain | Escherichia coli P0304816.5 | 335 | 5.8E-97 |  |
| 294 | EC174900_4753 | EMZ59497 | type-1 fimbrial protein, A chain | Escherichia coli 174900 | 335 | 5.8E-97 |  |
| 295 | A1W7_00268 | ELE93782 | fimbrial subunit type 1 | Escherichia coli KTE87 | 335 | 5.8E-97 |  |
| 296 | G940_04726 | EQX94612 | fimbrial subunit type 1 | Escherichia coli UMEA 3203-1 | 335 | 5.8E-97 |  |
| 297 | A1Y5_00863 | ELG57355 | fimbrial protein FimA | Escherichia coli KTE118 | 335 | 5.8E-97 |  |
| 298 | AD12_0001 | EZJ31867 | type-1 fimbrial protein, A chain | Escherichia coli 1-392-07_S4_C2 | 335 | 5.8E-97 |  |
| 299 | V412_07105 | ETE33670 | type 1 fimbrial protein | Escherichia coli LAU-EC7 | 335 | 5.8E-97 |  |
| 300 | G926_04498 | EQX26743 | fimbrial subunit type 1 | Escherichia coli UMEA 3163-1 | 335 | 5.8E-97 |  |
| 301 | BX99_22915 | EZQ39567 | type-1 fimbrial protein subunit A | Escherichia coli O111:H8 str. 2011C-3453 | 335 | 5.8E-97 |  |
| 302 | ECCG_02899 | EFE60472 | major type 1 subunit fimbrin | Escherichia coli B088 | 335 | 5.8E-97 |  |
| 303 | G840_04550 | EQT56808 | fimbrial subunit type 1 | Escherichia coli HVH 188 (4-2356988) | 335 | 5.8E-97 |  |
| 304 | EC2719100_0055 | EMX91818 | type-1 fimbrial protein, A chain | Escherichia coli 2719100 | 335 | 5.8E-97 |  |
| 305 | AB81_5186 | KEN66069 | type-1 fimbrial protein, A chain | Escherichia coli 6-537-08_S1_C3 | 335 | 5.8E-97 |  |
| 306 | P423_24540 | AGY86957 | type-1 fimbrial protein subunit A | Escherichia coli JJ1886 | 335 | 5.8E-97 | Yes |
| 307 | ECMP0209401_0051 | EMX61200 | type-1 fimbrial protein, A chain | Escherichia coli MP020940.1 | 335 | 5.8E-97 |  |
| 308 | ERS139235_01093 | CTX12028 | fimbrial protein | Escherichia coli (strain ATCC 9637 / CCM 2024 / DSM 1116 / NCIMB 8666 / NRRL B-766 / W) | 335 | 5.8E-97 |  |
| 309 | BZ00_10425 | EZD45064 | type-1 fimbrial protein subunit A | Escherichia coli O111:NM str. K6895 | 335 | 5.8E-97 |  |
| 310 | G968_04300 | ESK01923 | fimbrial subunit type 1 | Escherichia coli UMEA 3336-1 | 335 | 5.8E-97 |  |
| 311 | EC30301_5107 | EGW77986 | fimbrial subunit type 1 | Escherichia coli 3030-1 | 335 | 5.8E-97 |  |
| 312 | Q458_26340 | ETD60982 | type-1 fimbrial protein subunit A | Escherichia coli ATCC BAA-2209 | 335 | 5.8E-97 |  |
| 313 | BX37_12050 | EZQ27085 | type-1 fimbrial protein subunit A | Escherichia coli O111:H8 str. 2009EL-2169 | 335 | 5.8E-97 |  |
| 314 | A1S7_00576 | ELE01009 | fimbrial subunit type 1 | Escherichia coli KTE49 | 335 | 5.8E-97 |  |
| 315 | G708_04661 | EQO17947 | fimbrial subunit type 1 | Escherichia coli HVH 32 (4-3773988) | 335 | 5.8E-97 |  |
| 316 | EC90111_5732 | EII21479 | type-1 fimbrial protein, A chain | Escherichia coli 9.0111 | 335 | 5.8E-97 |  |
| 317 | ECOLIN_23995 | AID81487 | type-1 fimbrial protein subunit A | Escherichia coli Nissle 1917 | 335 | 5.8E-97 |  |
| 318 | BW71_19730 | EZA20102 | type-1 fimbrial protein subunit A | Escherichia coli O113:H21 str. 07-4224 | 335 | 5.8E-97 |  |
| 319 | G780_04718 | EQR11762 | fimbrial subunit type 1 | Escherichia coli HVH 118 (4-7345399) | 335 | 5.8E-97 |  |
| 320 | ECSTECO31_4917 | EJK93356 | fimbrial subunit type 1 | Escherichia coli STEC_O31 | 335 | 5.8E-97 |  |
| 321 | AD26_4636 | KDX37693 | type-1 fimbrial protein, A chain | Escherichia coli 2-156-04_S4_C3 | 335 | 5.8E-97 |  |
| 322 | BY46_04550 | EZA87044 | type-1 fimbrial protein subunit A | Escherichia coli O111:H8 str. F6627 | 335 | 5.8E-97 |  |
| 323 | A15G_01126 | ELH55216 | fimbrial protein FimA | Escherichia coli KTE203 | 335 | 5.8E-97 |  |
| 324 | AE38_04459 | KDF79911 | fimbrial subunit type 1 | Escherichia coli BIDMC 63 | 335 | 5.8E-97 |  |
| 325 | BX74_01850 | EYY78258 | type-1 fimbrial protein subunit A | Escherichia coli O111:NM str. 2010C-4746 | 335 | 5.8E-97 |  |
| 326 | A31I_00055 | ELF33247 | fimbrial subunit type 1 | Escherichia coli KTE162 | 335 | 5.8E-97 |  |
| 327 | BX76_08460 | EYY68971 | type-1 fimbrial protein subunit A | Escherichia coli O111:NM str. 2010C-4799 | 335 | 5.8E-97 |  |
| 328 | A1YQ_00360 | ELG76796 | fimbrial protein FimA | Escherichia coli KTE140 | 335 | 5.8E-97 |  |
| 329 | BZ01_24550 | EZD48100 | type-1 fimbrial protein subunit A | Escherichia coli O111:NM str. K6897 | 335 | 5.8E-97 |  |
| 330 | EC958_0045 | CDN85262 | type 1 fimbriae major subunit FimA | Escherichia coli O25b:H4-ST131 | 335 | 5.8E-97 |  |
| 331 | AE45_04380 | KDF95940 | fimbrial subunit type 1 | Escherichia coli BIDMC 70 | 335 | 5.8E-97 |  |
| 332 | EC178900_4787 | ENA54422 | type-1 fimbrial protein, A chain | Escherichia coli 178900 | 335 | 5.8E-97 | Yes |
| 333 | G912_04726 | EQW76248 | fimbrial subunit type 1 | Escherichia coli UMEA 3122-1 | 335 | 5.8E-97 |  |
| 334 | ECP02994832_0101 | END84220 | type-1 fimbrial protein, A chain | Escherichia coli P0299483.2 | 335 | 5.8E-97 |  |
| 335 | ECP030481614_4987 | ENF42431 | type-1 fimbrial protein, A chain | Escherichia coli P0304816.14 | 335 | 5.8E-97 |  |
| 336 | BX58_19470 | EYU91588 | type-1 fimbrial protein subunit A | Escherichia coli O111:NM str. 2010C-3977 | 335 | 5.8E-97 |  |
| 337 | EC33884_4993 | EII55864 | type-1 fimbrial protein, A chain | Escherichia coli 3.3884 | 335 | 5.8E-97 |  |
| 338 | A1U3_04657 | ELG21214 | fimbrial protein FimA | Escherichia coli KTE65 | 335 | 5.8E-97 |  |
| 339 | G851_04733 | EQU06186 | fimbrial subunit type 1 | Escherichia coli HVH 199 (4-5670322) | 335 | 5.8E-97 |  |
| 340 | BX21_19000 | EZQ43151 | type-1 fimbrial protein subunit A | Escherichia coli O111:H8 str. 2009C-4126 | 335 | 5.8E-97 |  |
| 341 | ECOG_02487 | EGI52678 | fimbrial subunit type 1 | Escherichia coli H299 | 335 | 5.8E-97 | Yes |
| 342 | ECoL_02142 | EFW75163 | type 1 fimbriae major subunit FimA | Escherichia coli EC4100B | 335 | 5.8E-97 |  |
| 343 | BZ03_14765 | EZD59560 | type-1 fimbrial protein subunit A | Escherichia coli O111:NM str. K6904 | 335 | 5.8E-97 |  |
| 344 | BY98_14575 | EZD38441 | type-1 fimbrial protein subunit A | Escherichia coli O111:NM str. K6728 | 335 | 5.8E-97 |  |
| 345 | ECO26H__800043 | CEK08507 | major type 1 subunit fimbrin (pilin) | Escherichia coli O26:H11 | 335 | 5.8E-97 |  |
| 346 | AE40_03891 | KDF94831 | fimbrial subunit type 1 | Escherichia coli BIDMC 65 | 335 | 5.8E-97 |  |
| 347 | EC96154_4682 | EIH98196 | type-1 fimbrial protein, A chain | Escherichia coli 96.154 | 335 | 5.8E-97 |  |
| 348 | EschWDRAFT_4535 | EFN35888 | Fimbrial protein | Escherichia coli W (GCA_000258145) | 335 | 5.8E-97 |  |
| 349 | ECP03018678_4731 | ENC85869 | type-1 fimbrial protein, A chain | Escherichia coli P0301867.8 | 335 | 5.8E-97 |  |
| 350 | HMPREF9539_03157 | EFU46268 | fimbrial protein | Escherichia coli MS 110-3 | 335 | 5.8E-97 |  |
| 351 | BX97_26175 | EYY04906 | type-1 fimbrial protein subunit A | Escherichia coli O111:NM str. 2011C-3362 | 335 | 5.8E-97 |  |
| 352 | ECDEC7B_4714 | EHV88108 | fimbrial subunit type 1 | Escherichia coli DEC7B | 335 | 5.8E-97 | Yes |
| 353 | BW80_07845 | EZA05261 | type-1 fimbrial protein subunit A | Escherichia coli O111:NM str. 03-3484 | 335 | 5.8E-97 |  |
| 354 | BY99_00395 | EZD44724 | type-1 fimbrial protein subunit A | Escherichia coli O111:NM str. K6890 | 335 | 5.8E-97 |  |
| 355 | WGY_04660 | ELJ77903 | fimbrial protein FimA | Escherichia coli KTE95 | 335 | 5.8E-97 |  |
| 356 | G706_04683 | EQO09235 | fimbrial subunit type 1 | Escherichia coli HVH 30 (4-2661829) | 335 | 5.8E-97 |  |
| 357 | ECSF_4255 | BAI57795 | type-1 fimbrial major subunit FimA | Escherichia coli SE15 | 335 | 5.8E-97 |  |
| 358 | G685_00707 | EQN23174 | fimbrial subunit type 1 | Escherichia coli HVH 5 (4-7148410) | 335 | 5.8E-97 |  |
| 359 | A1SO_00588 | ELE27249 | fimbrial subunit type 1 | Escherichia coli KTE58 | 335 | 5.8E-97 |  |
| 360 | G795_04494 | EQR82324 | fimbrial subunit type 1 | Escherichia coli HVH 137 (4-2124971) | 335 | 5.8E-97 |  |
| 361 | AB76_1916 | KDT62512 | type-1 fimbrial protein, A chain | Escherichia coli 3-267-03_S1_C3 | 335 | 5.8E-97 |  |
| 362 | G960_04691 | ERB28435 | fimbrial subunit type 1 | Escherichia coli UMEA 3292-1 | 335 | 5.8E-97 |  |
| 363 | CR63_21695 | KKJ99655 | type-1 fimbrial protein subunit A | Escherichia coli NB8 | 335 | 5.8E-97 |  |
| 364 | ECP030481612_4832 | ENF26944 | type-1 fimbrial protein, A chain | Escherichia coli P0304816.12 | 335 | 5.8E-97 |  |
| 365 | ERBG_01514 | EGC12410 | fimbrial protein | Escherichia coli E1167 | 335 | 5.8E-97 |  |
| 366 | WK1_04328 | ELI74402 | fimbrial protein FimA | Escherichia coli KTE138 | 335 | 5.8E-97 |  |
| 367 | AD41_5258 | KDZ42141 | type-1 fimbrial protein, A chain | Escherichia coli 3-020-07_S4_C3 | 335 | 5.8E-97 |  |
| 368 | AB34_5285 | KEO35328 | type-1 fimbrial protein, A chain | Escherichia coli 2-460-02_S1_C2 | 335 | 5.8E-97 |  |
| 369 | AD40_5106 | KEN62212 | type-1 fimbrial protein, A chain | Escherichia coli 1-392-07_S4_C3 | 335 | 5.8E-97 |  |
| 370 | G847_04317 | EQT88551 | fimbrial subunit type 1 | Escherichia coli HVH 195 (3-7155360) | 335 | 5.8E-97 |  |
| 371 | BW88_24065 | EYZ59765 | type-1 fimbrial protein subunit A | Escherichia coli O79:H7 str. 06-3501 | 335 | 5.8E-97 |  |
| 372 | ECDEC13A_0086 | EHX52170 | fimbrial subunit type 1 | Escherichia coli DEC13A | 335 | 5.8E-97 |  |
| 373 | AE37_04310 | KDF81702 | fimbrial subunit type 1 | Escherichia coli BIDMC 62 | 335 | 5.8E-97 |  |
| 374 | ECDEC13B_4645 | EHX55103 | fimbrial subunit type 1 | Escherichia coli DEC13B | 335 | 5.8E-97 |  |
| 375 | HMPREF1615_00166 | ESE12284 | type-1 fimbrial protein, A chain | Escherichia coli 908632 | 335 | 5.8E-97 |  |
| 376 | ECDEC9E_5623 | EHW49046 | fimbrial subunit type 1 | Escherichia coli DEC9E | 335 | 5.8E-97 | Yes |
| 377 | BW70_02170 | EZA37362 | type-1 fimbrial protein subunit A | Escherichia coli O174:H8 str. 04-3038 | 335 | 5.8E-97 |  |
| 378 | ECP03018671_5292 | EMX15066 | type-1 fimbrial protein, A chain | Escherichia coli P0301867.1 | 335 | 5.8E-97 | Yes |
| 379 | AC68_4692 | KDW12845 | type-1 fimbrial protein, A chain | Escherichia coli 2-156-04_S4_C1 | 335 | 5.8E-97 |  |
| 380 | G710_04744 | EQO25391 | fimbrial subunit type 1 | Escherichia coli HVH 35 (4-2962667) | 335 | 5.8E-97 |  |
| 381 | ECHM605_02320 | EIL82355 | major type 1 subunit fimbrin (pilin) FimA | Escherichia coli HM605 | 335 | 5.8E-97 |  |
| 382 | EC2785200_4679 | EMW30260 | type-1 fimbrial protein, A chain | Escherichia coli 2785200 | 335 | 5.8E-97 | Yes |
| 383 | AD28_4479 | KDX61190 | type-1 fimbrial protein, A chain | Escherichia coli 2-210-07_S4_C3 | 335 | 5.8E-97 |  |
| 384 | ECO9574_00310 | EIL27249 | fimbrial protein domain-containing protein | Escherichia coli O111:H8 str. CVM9574 | 335 | 5.8E-97 |  |
| 385 | BX71_12235 | EYY94445 | type-1 fimbrial protein subunit A | Escherichia coli O111:NM str. 2010C-4715 | 335 | 5.8E-97 |  |
| 386 | AE10_04568 | KDG67509 | fimbrial subunit type 1 | Escherichia coli UCI 51 | 335 | 5.8E-97 |  |
| 387 | AD31_5182 | KEJ42790 | type-1 fimbrial protein, A chain | Escherichia coli 2-427-07_S4_C3 | 335 | 5.8E-97 |  |
| 388 | ECNA114_4559 | AEG39408 | Putative type 1 fimbriae major subunit FimA | Escherichia coli NA114 | 335 | 5.8E-97 |  |
| 389 | ECSTEC94C_5153 | EGW77894 | fimbrial subunit type 1 | Escherichia coli STEC_94C | 335 | 5.8E-97 |  |
| 390 | ECP030481610_4877 | ENF19846 | type-1 fimbrial protein, A chain | Escherichia coli P0304816.10 | 335 | 5.8E-97 | Yes |
| 391 | CDL42382 | CDL42382 | type 1 fimbriae major subunit FimA | Escherichia coli ISC41 | 335 | 5.8E-97 |  |
| 392 | BY07_20005 | EYX72193 | type-1 fimbrial protein subunit A | Escherichia coli O111:NM str. 2011C-3679 | 335 | 5.8E-97 |  |
| 393 | CFSAN001632_05187 | EKU01690 | type-1 fimbrial major subunit FimA | Escherichia coli O111:H8 str. CFSAN001632 | 335 | 5.8E-97 |  |
| 394 | A1SY_00605 | ELG19914 | fimbrial protein FimA | Escherichia coli KTE63 | 335 | 5.8E-97 |  |
| 395 | HMPREF1597_03580 | ESD18453 | type-1 fimbrial protein, A chain | Escherichia coli 907701 | 335 | 5.8E-97 |  |
| 396 | HMPREF1590_02962 | ESC97931 | type-1 fimbrial protein, A chain | Escherichia coli 113302 | 335 | 5.8E-97 |  |
| 397 | L668_08200 | ERA59255 | type-1 fimbrial protein subunit A | Escherichia coli 95NR1 | 335 | 5.8E-97 |  |
| 398 | G862_04590 | ERA85081 | fimbrial subunit type 1 | Escherichia coli HVH 210 (4-3042480) | 335 | 5.8E-97 |  |
| 399 | A135_00500 | ELH31326 | fimbrial protein FimA | Escherichia coli KTE175 | 335 | 5.8E-97 |  |
| 400 | G807_04422 | EQS33136 | fimbrial subunit type 1 | Escherichia coli HVH 149 (4-4451880) | 335 | 5.8E-97 |  |
| 401 | G990_04479 | EQZ72252 | fimbrial subunit type 1 | Escherichia coli UMEA 3702-1 | 335 | 5.8E-97 |  |
| 402 | G696_04610 | EQN58012 | fimbrial subunit type 1 | Escherichia coli HVH 20 (4-5865042) | 335 | 5.8E-97 |  |
| 403 | C202_21551 | EMD02560 | type-1 fimbrial major subunit FimA | Escherichia coli O08 | 335 | 5.8E-97 |  |
| 404 | G734_04755 | EQP29545 | fimbrial subunit type 1 | Escherichia coli HVH 68 (4-0888028) | 335 | 5.8E-97 |  |
| 405 | ECIAI1_4530 | CAR01271 | major type 1 subunit fimbrin (pilin) | Escherichia coli IAI1 | 335 | 5.8E-97 |  |
| 406 | ECDEC8E_5334 | EHW21325 | fimbrial subunit type 1 | Escherichia coli DEC8E | 335 | 5.8E-97 |  |
| 407 | BX69_00610 | EYZ00775 | type-1 fimbrial protein subunit A | Escherichia coli O111:NM str. 2010C-4592 | 335 | 5.8E-97 |  |
| 408 | A17Q_04737 | EOX03371 | fimbrial protein FimA | Escherichia coli KTE226 | 335 | 5.8E-97 |  |
| 409 | WAY_04395 | EOU27301 | fimbrial protein FimA | Escherichia coli KTE13 | 335 | 5.8E-97 |  |
| 410 | BX73_05325 | EYY74087 | type-1 fimbrial protein subunit A | Escherichia coli O111:NM str. 2010C-4735 | 335 | 5.8E-97 |  |
| 411 | ECDEC13C_5261 | EHX54310 | fimbrial subunit type 1 | Escherichia coli DEC13C | 335 | 5.8E-97 |  |
| 412 | HMPREF1593_03580 | ESC95017 | type-1 fimbrial protein, A chain | Escherichia coli 907391 | 335 | 5.8E-97 |  |
| 413 | G681_04677 | EQN01354 | fimbrial subunit type 1 | Escherichia coli HVH 1 (4-6876161) | 335 | 5.8E-97 |  |
| 414 | BY96_07035 | EZD34003 | type-1 fimbrial protein subunit A | Escherichia coli O111:NM str. K6722 | 335 | 5.8E-97 |  |
| 415 | HMPREF1621_01431 | ESE36137 | type-1 fimbrial protein, A chain | Escherichia coli A25922R | 335 | 5.8E-97 |  |
| 416 | G863_04695 | EQU65341 | fimbrial subunit type 1 | Escherichia coli HVH 211 (4-3041891) | 335 | 5.8E-97 |  |
| 417 | LY180_22630 | AGW11315 | type-1 fimbrial protein subunit A | Escherichia coli LY180 | 335 | 5.8E-97 |  |
| 418 | G995_04742 | ERA00095 | fimbrial subunit type 1 | Escherichia coli UMEA 3805-1 | 335 | 5.8E-97 |  |
| 419 | BW91_08655 | EYZ42974 | type-1 fimbrial protein subunit A | Escherichia coli O91:H14 str. 06-3691 | 335 | 5.8E-97 |  |
| 420 | HMPREF1614_04604 | ESD94176 | type-1 fimbrial protein, A chain | Escherichia coli 908624 | 335 | 5.8E-97 |  |
| 421 | ECMT8_17701 | EIL75074 | major type 1 subunit fimbrin (pilin) FimA | Escherichia coli CUMT8 | 335 | 5.8E-97 |  |
| 422 | BZ05_12450 | EZD70136 | type-1 fimbrial protein subunit A | Escherichia coli O111:NM str. K6915 | 335 | 5.8E-97 |  |
| 423 | AD13_4602 | KDZ38192 | type-1 fimbrial protein, A chain | Escherichia coli 3-020-07_S4_C2 | 335 | 5.8E-97 |  |
| 424 | ESTG_04075 | EIG42814 | fimbrial subunit type 1 | Escherichia coli B799 | 335 | 5.8E-97 |  |
| 425 | G857_04823 | EQU41944 | fimbrial subunit type 1 | Escherichia coli HVH 205 (4-3094677) | 335 | 5.8E-97 |  |
| 426 | EC179100_4988 | END29354 | type-1 fimbrial protein, A chain | Escherichia coli 179100 | 335 | 5.8E-97 |  |
| 427 | AD24_4589 | KDT12168 | type-1 fimbrial protein, A chain | Escherichia coli 2-011-08_S4_C3 | 335 | 5.8E-97 |  |
| 428 | WK3_04467 | ELI79071 | fimbrial protein FimA | Escherichia coli KTE139 | 335 | 5.8E-97 |  |
| 429 | ERCG_03409 | EGB31599 | fimbrial protein | Escherichia coli E1520 | 335 | 5.8E-97 | Yes |
| 430 | HMPREF1598_03564 | ESD18255 | type-1 fimbrial protein, A chain | Escherichia coli 907710 | 335 | 5.8E-97 |  |
| 431 | G855_04533 | EQU29465 | fimbrial subunit type 1 | Escherichia coli HVH 203 (4-3126218) | 335 | 5.8E-97 |  |
| 432 | WCQ_04447 | ELC17020 | fimbrial subunit type 1 | Escherichia coli KTE12 | 335 | 5.8E-97 |  |
| 433 | ECC1470_03199 | EST87068 | major type 1 subunit fimbrin (pilin) FimA | Escherichia coli ECC-1470 (GCA_000831565) | 335 | 5.8E-97 |  |
| 434 | ECDEC10F_0173 | EHX02330 | fimbrial subunit type 1 | Escherichia coli DEC10F | 335 | 5.8E-97 |  |
| 435 | A17E_04400 | ELD45367 | fimbrial subunit type 1 | Escherichia coli KTE220 | 335 | 5.8E-97 |  |
| 436 | AC84_5475 | KEN92328 | type-1 fimbrial protein, A chain | Escherichia coli 1-392-07_S4_C1 | 335 | 5.8E-97 |  |
| 437 | AE54_03978 | KDG42466 | fimbrial subunit type 1 | Escherichia coli BIDMC 79 | 335 | 5.8E-97 |  |
| 438 | A1YA_02094 | ELG61910 | fimbrial protein FimA | Escherichia coli KTE123 | 335 | 5.8E-97 |  |
| 439 | BX16_16455 | EZE34091 | type-1 fimbrial protein subunit A | Escherichia coli O91:NM str. 2009C-3745 | 335 | 5.8E-97 |  |
| 440 | G702_04600 | EQN87820 | fimbrial subunit type 1 | Escherichia coli HVH 26 (4-5703913) | 335 | 5.8E-97 |  |
| 441 | ECP030186711_4982 | ENC87489 | type-1 fimbrial protein, A chain | Escherichia coli P0301867.11 | 335 | 5.8E-97 |  |
| 442 | ECP03018673_4955 | ENG93185 | type-1 fimbrial protein, A chain | Escherichia coli P0301867.3 | 335 | 5.8E-97 |  |
| 443 | AD01_4597 | KDY41281 | type-1 fimbrial protein, A chain | Escherichia coli 2-427-07_S4_C2 | 335 | 5.8E-97 |  |
| 444 | BW78_20135 | EYZ94307 | type-1 fimbrial protein subunit A | Escherichia coli O174:H21 str. 03-3269 | 335 | 5.8E-97 |  |
| 445 | G853_04725 | EQU18636 | fimbrial subunit type 1 | Escherichia coli HVH 201 (4-4459431) | 335 | 5.8E-97 |  |
| 446 | BX70_24805 | EYZ01494 | type-1 fimbrial protein subunit A | Escherichia coli O111:NM str. 2010C-4622 | 335 | 5.8E-97 |  |
| 447 | BY97_00395 | EZD28881 | type-1 fimbrial protein subunit A | Escherichia coli O111:NM str. K6723 | 335 | 5.8E-97 |  |
| 448 | ECO111_5168 | BAI38885 | major type 1 subunit fimbrin (pilin) FimA | Escherichia coli O111:H- str. 11128 | 335 | 5.8E-97 | Yes |
| 449 | A17K_00420 | ELH94120 | fimbrial protein FimA | Escherichia coli KTE223 | 335 | 5.8E-97 |  |
| 450 | ECDEC9B_5155 | EHW33802 | fimbrial subunit type 1 | Escherichia coli DEC9B | 335 | 5.8E-97 |  |
| 451 | AC71_4562 | KEM85271 | type-1 fimbrial protein, A chain | Escherichia coli 2-222-05_S4_C1 | 335 | 5.8E-97 |  |
| 452 | EC12264_5380 | EIH21468 | type-1 fimbrial protein, A chain | Escherichia coli 1.2264 | 335 | 5.8E-97 |  |
| 453 | G838_04308 | EQT51890 | fimbrial subunit type 1 | Escherichia coli HVH 186 (4-3405044) | 335 | 5.8E-97 |  |
| 454 | DR76_2425 | AIL16371 | type-1 fimbrial protein, A chain | Escherichia coli ATCC 25922 | 335 | 5.8E-97 |  |
| 455 | ECO9570_12993 | EIL26775 | fimbrial protein domain-containing protein | Escherichia coli O111:H8 str. CVM9570 | 335 | 5.8E-97 |  |
| 456 | G692_04615 | EQN42726 | fimbrial subunit type 1 | Escherichia coli HVH 16 (4-7649002) | 335 | 5.8E-97 |  |
| 457 | A171_04107 | ELD28729 | fimbrial subunit type 1 | Escherichia coli KTE213 | 335 | 5.8E-97 |  |
| 458 | ECP03018677_5002 | ENH04209 | type-1 fimbrial protein, A chain | Escherichia coli P0301867.7 | 335 | 5.8E-97 |  |
| 459 | ECP03048167_4877 | ENF58802 | type-1 fimbrial protein, A chain | Escherichia coli P0304816.7 | 335 | 5.8E-97 |  |
| 460 | BX18_22300 | EZE38046 | type-1 fimbrial protein subunit A | Escherichia coli O111:NM str. 2009C-4006 | 335 | 5.8E-97 |  |
| 461 | AB18_1739 | KDU19996 | type-1 fimbrial protein, A chain | Escherichia coli 3-267-03_S1_C1 | 335 | 5.8E-97 |  |
| 462 | L411_05108 | ERO93601 | fimbrial subunit type 1 | Escherichia coli BWH 24 | 335 | 5.8E-97 |  |
| 463 | ECOK1180_4893 | EFZ61794 | fimbrial subunit type 1 | Escherichia coli OK1180 | 335 | 5.8E-97 |  |
| 464 | ECP03048166_4874 | ENF44660 | type-1 fimbrial protein, A chain | Escherichia coli P0304816.6 | 335 | 5.8E-97 |  |
| 465 | BX03_00020 | EYW94423 | type-1 fimbrial protein subunit A | Escherichia coli O111:NM str. 08-4487 | 335 | 5.8E-97 |  |
| 466 | ECDEC9D_5245 | EHW47660 | fimbrial subunit type 1 | Escherichia coli DEC9D | 335 | 5.8E-97 |  |
| 467 | HMPREF1601_01786 | ESA90739 | type-1 fimbrial protein, A chain | Escherichia coli 907779 | 335 | 5.8E-97 |  |
| 468 | L667_09980 | ERE04306 | type-1 fimbrial protein subunit A | Escherichia coli 95JB1 | 335 | 5.8E-97 |  |
| 469 | HMPREF1592_04026 | ESA74051 | type-1 fimbrial protein, A chain | Escherichia coli 907357 | 335 | 5.8E-97 |  |
| 470 | ESPG_02754 | EHN96215 | fimbrial subunit type 1 | Escherichia coli H397 | 335 | 5.8E-97 |  |
| 471 | AC88_5107 | KEJ55381 | type-1 fimbrial protein, A chain | Escherichia coli 3-267-03_S4_C1 | 335 | 5.8E-97 |  |
| 472 | WK7_04597 | ELI90000 | fimbrial protein FimA | Escherichia coli KTE148 | 335 | 5.8E-97 |  |
| 473 | ECDEC8A_5252 | EHW03141 | type-1 fimbrial protein, A chain | Escherichia coli DEC8A | 335 | 5.8E-97 |  |
| 474 | AE24_04576 | KDG86403 | fimbrial subunit type 1 | Escherichia coli UCI 65 | 335 | 5.8E-97 |  |
| 475 | AB24_5049 | KEM36345 | type-1 fimbrial protein, A chain | Escherichia coli 6-537-08_S1_C1 | 335 | 5.8E-97 |  |
| 476 | L456_04788 | ETX87701 | fimbrial subunit type 1 | Escherichia coli BIDMC 20B | 335 | 5.8E-97 |  |
| 477 | G952_04890 | EQY50234 | fimbrial subunit type 1 | Escherichia coli UMEA 3240-1 | 335 | 5.8E-97 |  |
| 478 | AC58_3301 | KDU09479 | type-1 fimbrial protein, A chain | Escherichia coli 3-105-05_S3_C3 | 335 | 5.8E-97 |  |
| 479 | ECDEC9A_5607 | EHW28643 | fimbrial subunit type 1 | Escherichia coli DEC9A | 335 | 5.8E-97 |  |
| 480 | ECP030186713_5105 | END86267 | type-1 fimbrial protein, A chain | Escherichia coli P0301867.13 | 335 | 5.8E-97 |  |
| 481 | G941_04668 | EQX95572 | fimbrial subunit type 1 | Escherichia coli UMEA 3206-1 | 335 | 5.8E-97 |  |
| 482 | G763_00438 | EQQ43924 | fimbrial subunit type 1 | Escherichia coli HVH 102 (4-6906788) | 335 | 5.8E-97 |  |
| 483 | WEM_01814 | EOU79643 | fimbrial protein FimA | Escherichia coli KTE27 | 335 | 5.8E-97 |  |
| 484 | ECK71_07028 | KRR59682 | type-1 fimbrial major subunit FimA | Escherichia coli K71 | 335 | 5.8E-97 |  |
| 485 | BY03_08470 | EYX95759 | type-1 fimbrial protein subunit A | Escherichia coli O111:NM str. 2011C-3573 | 335 | 5.8E-97 |  |
| 486 | G945_04477 | EQY17066 | fimbrial subunit type 1 | Escherichia coli UMEA 3216-1 | 335 | 5.8E-97 |  |
| 487 | HMPREF9542_04432 | EGB86151 | fimbrial protein | Escherichia coli MS 117-3 | 335 | 5.8E-97 | Yes |
| 488 | A1SQ_00455 | ELG19615 | fimbrial protein FimA | Escherichia coli KTE59 | 335 | 5.8E-97 |  |
| 489 | AE50_04500 | KDG21482 | fimbrial subunit type 1 | Escherichia coli BIDMC 75 | 335 | 5.8E-97 |  |
| 490 | AE33_04522 | KDF70476 | fimbrial subunit type 1 | Escherichia coli BIDMC 58 | 335 | 5.8E-97 |  |
| 491 | AB05_5041 | KEO24758 | type-1 fimbrial protein, A chain | Escherichia coli 2-460-02_S1_C1 | 335 | 5.8E-97 |  |
| 492 | BX20_15820 | EZE49752 | type-1 fimbrial protein subunit A | Escherichia coli O111:NM str. 2009C-4052 | 335 | 5.8E-97 |  |
| 493 | ECDEC8B_5519 | EHW02962 | fimbrial subunit type 1 | Escherichia coli DEC8B | 335 | 5.8E-97 | Yes |
| 494 | AC44_0024 | KEO17095 | type-1 fimbrial protein, A chain | Escherichia coli 2-177-06_S3_C3 | 335 | 5.8E-97 |  |
| 495 | AC69_5111 | KDX42650 | type-1 fimbrial protein, A chain | Escherichia coli 2-177-06_S4_C1 | 335 | 5.8E-97 |  |
| 496 | WKM_04407 | ELJ20332 | fimbrial protein FimA | Escherichia coli KTE167 | 335 | 5.8E-97 |  |
| 497 | BX39_17135 | EZQ24496 | type-1 fimbrial protein subunit A | Escherichia coli O111:NM str. 2010C-3053 | 335 | 5.8E-97 |  |
| 498 | G739_04731 | EQP57044 | fimbrial subunit type 1 | Escherichia coli HVH 76 (4-2538717) | 335 | 5.8E-97 |  |
| 499 | ECSE_4587 | BAG80111 | type-1 fimbrial major subunit FimA | Escherichia coli (strain SE11) | 335 | 5.8E-97 |  |
| 500 | HMPREF1618_04151 | ESE15044 | type-1 fimbrial protein, A chain | Escherichia coli 908691 | 335 | 5.8E-97 |  |
| 501 | BX07_07440 | EZD94719 | type-1 fimbrial protein subunit A | Escherichia coli O91:H14 str. 2009C-3227 | 335 | 5.8E-97 |  |
| 502 | CDK60031 | CDK60031 | type 1 fimbriae major subunit FimA | Escherichia coli IS9 | 335 | 5.8E-97 |  |
| 503 | ECDEC13E_4947 | EHX67656 | fimbrial subunit type 1 | Escherichia coli DEC13E | 335 | 5.8E-97 |  |
| 504 | A1SM_00306 | ELE26409 | fimbrial subunit type 1 | Escherichia coli KTE57 | 335 | 5.8E-97 |  |
| 505 | BW82_15190 | EYZ89914 | type-1 fimbrial protein subunit A | Escherichia coli O111:NM str. 04-3211 | 335 | 5.8E-97 |  |
| 506 | ECP03048163_4903 | ENH27332 | type-1 fimbrial protein, A chain | Escherichia coli P0304816.3 | 335 | 5.8E-97 |  |
| 507 | ECDEC9C_5166 | EHW39333 | fimbrial subunit type 1 | Escherichia coli DEC9C | 335 | 5.8E-97 |  |
| 508 | AC35_2646 | KEK87153 | type-1 fimbrial protein, A chain | Escherichia coli 3-475-03_S3_C2 | 335 | 5.8E-97 |  |
| 509 | A133_00460 | ELH30417 | fimbrial protein FimA | Escherichia coli KTE173 | 335 | 5.8E-97 |  |
| 510 | ECJB195_5462 | EIH87987 | type-1 fimbrial protein, A chain | Escherichia coli JB1-95 | 335 | 5.8E-97 |  |
| 511 | ECO9634_18976 | EJE75074 | fimbrial protein domain-containing protein | Escherichia coli O111:H8 str. CVM9634 | 335 | 5.8E-97 |  |
| 512 | ECDEC5E_5461 | EHV41949 | type-1 fimbrial protein, A chain | Escherichia coli DEC5E | 335 | 5.8E-97 |  |
| 513 | CDL05889 | CDL05889 | type 1 fimbriae major subunit FimA | Escherichia coli IS35 | 335 | 5.8E-97 |  |
| 514 | BZ02_23725 | EZD49902 | type-1 fimbrial protein subunit A | Escherichia coli O111:NM str. K6898 | 335 | 5.8E-97 |  |
| 515 | HMPREF1607_02899 | ESD56769 | type-1 fimbrial protein, A chain | Escherichia coli 908524 | 335 | 5.8E-97 |  |
| 516 | ESAG_03938 | EEH88226 | fimbrial subunit type 1 | Escherichia sp. 3_2_53FAA | 335 | 5.8E-97 |  |
| 517 | ECP03018675_5028 | ENG96461 | type-1 fimbrial protein, A chain | Escherichia coli P0301867.5 | 335 | 5.8E-97 |  |
| 518 | AF24_03970 | KDG47793 | fimbrial subunit type 1 | Escherichia coli CHS 68 | 335 | 5.8E-97 |  |
| 519 | G927_04625 | EQX28037 | fimbrial subunit type 1 | Escherichia coli UMEA 3172-1 | 335 | 5.8E-97 |  |
| 520 | L404_04791 | ETY47959 | fimbrial subunit type 1 | Escherichia coli BWH 34 | 335 | 5.8E-97 |  |
| 521 | G742_04540 | EQP66787 | fimbrial subunit type 1 | Escherichia coli HVH 79 (4-2512823) | 335 | 5.8E-97 |  |
| 522 | BX59_03330 | EYU86274 | type-1 fimbrial protein subunit A | Escherichia coli O111:NM str. 2010C-4086 | 335 | 5.8E-97 |  |
| 523 | AC97_5038 | KDW38134 | type-1 fimbrial protein, A chain | Escherichia coli 2-177-06_S4_C2 | 335 | 5.8E-97 |  |
| 524 | G984_04773 | EQZ48774 | fimbrial subunit type 1 | Escherichia coli UMEA 3662-1 | 335 | 5.8E-97 |  |
| 525 | AB52_4904 | KEN41173 | type-1 fimbrial protein, A chain | Escherichia coli 6-537-08_S1_C2 | 335 | 5.8E-97 |  |
| 526 | G844_04749 | EQT77252 | fimbrial subunit type 1 | Escherichia coli HVH 192 (4-3054470) | 335 | 5.8E-97 |  |
| 527 | AC16_3142 | KDX44284 | type-1 fimbrial protein, A chain | Escherichia coli 2-177-06_S3_C2 | 335 | 5.8E-97 |  |
| 528 | G746_04710 | EQP84598 | fimbrial subunit type 1 | Escherichia coli HVH 84 (4-1021478) | 335 | 5.8E-97 |  |
| 529 | ECO9602_05480 | EJE67479 | fimbrial protein domain-containing protein | Escherichia coli O111:H8 str. CVM9602 | 335 | 5.8E-97 |  |
| 530 | KO11_23195 | AFH19595 | major type 1 subunit fimbrin (pilin) FimA | Escherichia coli KO11 | 335 | 5.8E-97 | Yes |
| 531 | HMPREF1596_02535 | ESD11596 | type-1 fimbrial protein, A chain | Escherichia coli 907700 | 335 | 5.8E-97 |  |
| 532 | ECP03048162_4809 | ENF44280 | type-1 fimbrial protein, A chain | Escherichia coli P0304816.2 | 335 | 5.8E-97 |  |
| 533 | ECP03048169_4819 | ENF66260 | type-1 fimbrial protein, A chain | Escherichia coli P0304816.9 | 335 | 5.8E-97 |  |
| 534 | ECP03018674_4978 | ENA27974 | type-1 fimbrial protein, A chain | Escherichia coli P0301867.4 | 335 | 5.8E-97 |  |
| 535 | A15U_00340 | ELD25454 | fimbrial subunit type 1 | Escherichia coli KTE210 | 335 | 5.8E-97 |  |
| 536 | ECP03018672_4950 | ENA40926 | type-1 fimbrial protein, A chain | Escherichia coli P0301867.2 | 335 | 5.8E-97 |  |
| 537 | BY05_09775 | EYX78483 | type-1 fimbrial protein subunit A | Escherichia coli O111:NM str. 2011C-3632 | 335 | 5.8E-97 |  |
| 538 | HMPREF1609_05023 | ESD66402 | type-1 fimbrial protein, A chain | Escherichia coli 908541 | 335 | 5.8E-97 |  |
| 539 | V411_26250 | ETE09727 | type 1 fimbrial protein | Escherichia coli LAU-EC6 | 335 | 5.8E-97 |  |
| 540 | ECP02994831_0098 | END74257 | type-1 fimbrial protein, A chain | Escherichia coli P0299483.1 | 335 | 5.8E-97 |  |
| 541 | AE53_02593 | KDG37313 | fimbrial subunit type 1 | Escherichia coli BIDMC 78 | 335 | 5.8E-97 |  |
| 542 | ECP030481611_4777 | ENF16178 | type-1 fimbrial protein, A chain | Escherichia coli P0304816.11 | 335 | 5.8E-97 |  |
| 543 | ERLG_03377 | EGB51155 | fimbrial protein | Escherichia coli H263 | 335 | 5.8E-97 |  |
| 544 | G805_04502 | EQS28701 | fimbrial subunit type 1 | Escherichia coli HVH 147 (4-5893887) | 335 | 5.8E-97 |  |
| 545 | A1Y1_04526 | ELG45754 | fimbrial protein FimA | Escherichia coli KTE115 | 335 | 5.8E-97 | Yes |
| 546 | EC253486_0090 | EGW78208 | fimbrial subunit type 1 | Escherichia coli 2534-86 | 335 | 5.8E-97 |  |
| 547 | A15W_00404 | ELH78501 | fimbrial protein FimA | Escherichia coli KTE211 | 335 | 5.8E-97 |  |
| 548 | AC07_2915 | KEK76819 | type-1 fimbrial protein, A chain | Escherichia coli 3-475-03_S3_C1 | 335 | 5.8E-97 |  |
| 549 | BW73_21785 | KDV16238 | type-1 fimbrial protein subunit A | Escherichia coli O111:NM str. 01-3076 | 335 | 5.8E-97 |  |
| 550 | AC66_3043 | KDT04850 | type-1 fimbrial protein, A chain | Escherichia coli 2-011-08_S4_C1 | 335 | 5.8E-97 |  |
| 551 | BZ04_15305 | EZD66922 | type-1 fimbrial protein subunit A | Escherichia coli O111:NM str. K6908 | 335 | 5.8E-97 |  |
| 552 | G778_04647 | EQR01022 | fimbrial subunit type 1 | Escherichia coli HVH 116 (4-6879942) | 335 | 5.8E-97 |  |
| 553 | BX93_03155 | EYY07757 | type-1 fimbrial protein subunit A | Escherichia coli O111:NM str. 2011C-3170 | 335 | 5.8E-97 |  |
| 554 | EREG_02531 | EGB41875 | fimbrial protein | Escherichia coli H120 | 335 | 5.8E-97 |  |
| 555 | AE51_04128 | KDG24971 | fimbrial subunit type 1 | Escherichia coli BIDMC 76 | 335 | 5.8E-97 |  |
| 556 | HMPREF1594_01822 | ESC98959 | type-1 fimbrial protein, A chain | Escherichia coli 907446 | 335 | 5.8E-97 |  |
| 557 | WEA_04455 | ELF87804 | fimbrial protein FimA | Escherichia coli KTE22 | 335 | 5.8E-97 |  |
| 558 | LI75_01950 | AIT33114 | type-1 fimbrial protein subunit A | Escherichia coli FAP1 | 335 | 5.8E-97 |  |
| 559 | ECP03048161_2215 | EMZ97820 | type-1 fimbrial protein, A chain | Escherichia coli P0304816.1 | 335 | 5.8E-97 |  |
| 560 | PU62_20705 | KHH26076 | type-1 fimbrial protein subunit A | Escherichia coli (GCA_000797585) | 334.8 | 6.5E-97 | Yes |
| 561 | AC15_4764 | KDW27064 | type-1 fimbrial protein, A chain | Escherichia coli 2-156-04_S3_C2 | 334.2 | 1E-96 | Yes |
| 562 | ECP030529312_4707 | ENG39269 | type-1 fimbrial protein, A chain | Escherichia coli p0305293.12 | 333.5 | 1.6E-96 | Yes |
| 563 | ABE89_08835 | KML98774 | type-1 fimbrial protein subunit A | Escherichia coli (GCA_001039205) | 333.4 | 1.7E-96 | Yes |
| 564 | PU10_25455 | KHJ05288 | type-1 fimbrial protein subunit A | Escherichia coli (GCA_000798315) | 333.4 | 1.7E-96 | Yes |
| 565 | AM272_14640 | KQJ40305 | type-1 fimbrial protein subunit A | Escherichia coli (GCA_001420135) | 333.4 | 1.7E-96 | Yes |
| 566 | SSJG_02831 | EGJ06782 | type-1 fimbrial protein, A chain | Escherichia coli D9 | 333.4 | 1.7E-96 | Yes |
| 567 | PPECC33_04814 | AKK51302 | type-1 fimbrial major subunit FimA | Escherichia coli PCN033 | 333.2 | 2E-96 | Yes |
| 568 | M13_15251 | EST67445 | major type 1 subunit fimbrin (pilin) FimA | Escherichia coli P4-96 | 333.2 | 2E-96 |  |
| 569 | MOI_17839 | EST67522 | major type 1 subunit fimbrin (pilin) FimA | Escherichia coli P4-NR | 333.2 | 2E-96 |  |
| 570 | ERFG_03556 | EGB70717 | fimbrial protein | Escherichia coli TW10509 | 333.2 | 2E-96 |  |
| 571 | EC2730350_4614 | ENA73803 | type-1 fimbrial protein, A chain | Escherichia coli 2730350 | 333.2 | 2E-96 |  |
| 572 | AE34_00420 | KDF73171 | type-1 fimbrial protein, A chain | Escherichia coli BIDMC 59 | 333.2 | 2.1E-96 | Yes |
| 573 | HMPREF9548_04477 | EFK00878 | fimbrial protein | Escherichia coli MS 182-1 | 332.9 | 2.6E-96 | Yes |
| 574 | AGA22_01995 | KNY66905 | type-1 fimbrial protein subunit A | Escherichia coli (GCA_001262805) | 332.8 | 2.8E-96 |  |
| 575 | EC50588_4790 | EIH00946 | type-1 fimbrial protein, A chain | Escherichia coli 5.0588 | 332.8 | 2.8E-96 | Yes |
| 576 | PU36_12320 | KHI15601 | type-1 fimbrial protein subunit A | Escherichia coli | 332.2 | 4.1E-96 | Yes |
| 577 | A364_00205 | EMD15419 | type-1 fimbrial major subunit FimA | Escherichia coli SEPT362 | 332.2 | 4.1E-96 |  |
| 578 | WQ89_04490 | KNF81720 | type-1 fimbrial protein subunit A | Escherichia coli | 332.2 | 4.1E-96 | Yes |
| 579 | AM260_11160 | KQI85933 | type-1 fimbrial protein subunit A | Escherichia coli (GCA_001419845) | 332.1 | 4.5E-96 | Yes |
| 580 | ECAD30_15130 | EKJ83607 | Fimbrial protein | Escherichia coli AD30 | 332 | 4.7E-96 | Yes |
| 581 | BU54_25055 | KDV42706 | type-1 fimbrial protein subunit A | Escherichia coli O45:H2 str. 2010C-4211 | 332 | 4.9E-96 |  |
| 582 | L342_2270 | ESS94099 | type 1 fimbriae major subunit FimA | Escherichia coli CE516 | 332 | 4.9E-96 |  |
| 583 | BW84_24120 | EYZ85335 | type-1 fimbrial protein subunit A | Escherichia coli O118:H16 str. 06-3256 | 332 | 4.9E-96 |  |
| 584 | ECP03052939_4584 | ENG74312 | type-1 fimbrial protein, A chain | Escherichia coli p0305293.9 | 332 | 4.9E-96 |  |
| 585 | EC2851500_4843 | EMV96308 | type-1 fimbrial protein, A chain | Escherichia coli 2851500 | 332 | 4.9E-96 |  |
| 586 | ECP03052938_4729 | ENG67741 | type-1 fimbrial protein, A chain | Escherichia coli p0305293.8 | 332 | 4.9E-96 |  |
| 587 | A1SU_00020 | EOV57279 | fimbrial protein FimA | Escherichia coli KTE61 | 332 | 4.9E-96 |  |
| 588 | AC70_1383 | KDW89167 | type-1 fimbrial protein, A chain | Escherichia coli 2-210-07_S4_C1 | 332 | 4.9E-96 | Yes |
| 589 | ECDEC10E_5185 | EHW84100 | fimbrial subunit type 1 | Escherichia coli DEC10E | 332 | 4.9E-96 | Yes |
| 590 | CFSAN001629_05885 | EKU02269 | major type 1 subunit fimbrin (pilin) FimA | Escherichia coli O26:H11 str. CFSAN001629 | 332 | 4.9E-96 |  |
| 591 | BX00_00700 | EYX06771 | type-1 fimbrial protein subunit A | Escherichia coli O118:H16 str. 08-3651 | 332 | 4.9E-96 |  |
| 592 | EC2850750_4915 | EMW00350 | type-1 fimbrial protein, A chain | Escherichia coli 2850750 | 332 | 4.9E-96 |  |
| 593 | EC179550_4667 | ENA57793 | type-1 fimbrial protein, A chain | Escherichia coli 179550 | 332 | 4.9E-96 |  |
| 594 | ECJURUA2010_4785 | EMX45212 | type-1 fimbrial protein, A chain | Escherichia coli Jurua 20/10 | 332 | 4.9E-96 |  |
| 595 | EC2729250_4766 | ENA47438 | type-1 fimbrial protein, A chain | Escherichia coli 2729250 | 332 | 4.9E-96 |  |
| 596 | A1WY_00576 | ELF02992 | type-1 fimbrial protein, A chain | Escherichia coli KTE111 | 332 | 4.9E-96 |  |
| 597 | ECDEC11B_5026 | EHW98401 | fimbrial subunit type 1 | Escherichia coli DEC11B | 332 | 4.9E-96 |  |
| 598 | EC930624_5289 | EIH67661 | type-1 fimbrial protein, A chain | Escherichia coli 93.0624 | 332 | 4.9E-96 |  |
| 599 | BW90_07285 | EYZ69609 | type-1 fimbrial protein subunit A | Escherichia coli O118:H16 str. 06-3612 | 332 | 4.9E-96 |  |
| 600 | BW86_17875 | EZG48600 | type-1 fimbrial protein subunit A | Escherichia coli O26:H11 str. 06-3464 | 332 | 4.9E-96 |  |
| 601 | A1YS_00312 | ELG82368 | fimbrial protein FimA | Escherichia coli KTE141 | 332 | 4.9E-96 |  |
| 602 | ECP03052932_4661 | ENG51731 | type-1 fimbrial protein, A chain | Escherichia coli p0305293.2 | 332 | 4.9E-96 |  |
| 603 | ECDEC14D_5076 | EHX84443 | fimbrial subunit type 1 | Escherichia coli DEC14D | 332 | 4.9E-96 | Yes |
| 604 | G918_04486 | ERB12095 | type-1 fimbrial protein, A chain | Escherichia coli UMEA 3150-1 | 332 | 4.9E-96 |  |
| 605 | EC178850_4558 | ENG83831 | type-1 fimbrial protein, A chain | Escherichia coli 178850 | 332 | 4.9E-96 |  |
| 606 | EC2850400_4933 | EMW13082 | type-1 fimbrial protein, A chain | Escherichia coli 2850400 | 332 | 4.9E-96 |  |
| 607 | BX56_22435 | EYU87668 | type-1 fimbrial protein subunit A | Escherichia coli O45:H2 str. 2010C-3876 | 332 | 4.9E-96 |  |
| 608 | Q456_0220005 | ETJ57253 | type-1 fimbrial protein subunit A | Escherichia coli ATCC BAA-2193 | 332 | 4.9E-96 |  |
| 609 | AB80_4970 | KEM21291 | type-1 fimbrial protein, A chain | Escherichia coli 6-319-05_S1_C3 | 332 | 4.9E-96 |  |
| 610 | BX85_15680 | EZG75449 | type-1 fimbrial protein subunit A | Escherichia coli O26:H11 str. 2010C-5028 | 332 | 4.9E-96 |  |
| 611 | BX24_08655 | EZE65813 | type-1 fimbrial protein subunit A | Escherichia coli O91:H21 str. 2009C-4646 | 332 | 4.9E-96 |  |
| 612 | EC2872800_4867 | EMV42138 | type-1 fimbrial protein, A chain | Escherichia coli 2872800 | 332 | 4.9E-96 |  |
| 613 | ECP03052931_5088 | EMZ79613 | type-1 fimbrial protein, A chain | Escherichia coli p0305293.1 | 332 | 4.9E-96 |  |
| 614 | ECC69171_18600 | KIG25320 | type-1 fimbrial protein subunit A | Escherichia coli C691-71 (14b) | 332 | 4.9E-96 |  |
| 615 | EC174750_4753 | EMW90148 | type-1 fimbrial protein, A chain | Escherichia coli 174750 | 332 | 4.9E-96 | Yes |
| 616 | ECMP0215527_4999 | EMU56332 | type-1 fimbrial protein, A chain | Escherichia coli MP021552.7 | 332 | 4.9E-96 |  |
| 617 | ECO26_5511 | BAI28633 | major type 1 subunit fimbrin (pilin) FimA | Escherichia coli O26:H11 str. 11368 | 332 | 4.9E-96 |  |
| 618 | AB23_4764 | KEM96534 | type-1 fimbrial protein, A chain | Escherichia coli 6-319-05_S1_C1 | 332 | 4.9E-96 |  |
| 619 | ECDEC10C_0026 | EHW81821 | fimbrial subunit type 1 | Escherichia coli DEC10C | 332 | 4.9E-96 |  |
| 620 | AF33_04549 | KDG50630 | type-1 fimbrial protein, A chain | Escherichia coli CHS 77 | 332 | 4.9E-96 |  |
| 621 | BX26_16145 | EZQ32857 | type-1 fimbrial protein subunit A | Escherichia coli O26:H1 str. 2009C-4747 | 332 | 4.9E-96 |  |
| 622 | EC23916_5644 | EII45186 | type-1 fimbrial protein, A chain | Escherichia coli 2.3916 | 332 | 4.9E-96 |  |
| 623 | EC900105_5890 | EIJ16464 | type-1 fimbrial protein, A chain | Escherichia coli 900105 (10e) | 332 | 4.9E-96 |  |
| 624 | EC80569_4589 | EKK39132 | type-1 fimbrial protein, A chain | Escherichia coli 8.0569 | 332 | 4.9E-96 |  |
| 625 | BY06_21860 | EZH08784 | type-1 fimbrial protein subunit A | Escherichia coli O26:H11 str. 2011C-3655 | 332 | 4.9E-96 |  |
| 626 | ECMP02155211_4660 | EMU56359 | type-1 fimbrial protein, A chain | Escherichia coli MP021552.11 | 332 | 4.9E-96 |  |
| 627 | BX95_19110 | EZG86391 | type-1 fimbrial protein subunit A | Escherichia coli O26:H11 str. 2011C-3270 | 332 | 4.9E-96 |  |
| 628 | ECTW15901_4874 | EKI14219 | type-1 fimbrial protein, A chain | Escherichia coli TW15901 | 332 | 4.9E-96 |  |
| 629 | ECDEC11A_4996 | EHW84758 | fimbrial subunit type 1 | Escherichia coli DEC11A | 332 | 4.9E-96 |  |
| 630 | BX65_19300 | EYZ12839 | type-1 fimbrial protein subunit A | Escherichia coli O103:H2 str. 2010C-4433 | 332 | 4.9E-96 |  |
| 631 | Q457_24080 | ETI72928 | type-1 fimbrial protein subunit A | Escherichia coli ATCC BAA-2196 | 332 | 4.9E-96 |  |
| 632 | AB78_2314 | KEK99689 | type-1 fimbrial protein, A chain | Escherichia coli 4-203-08_S1_C3 | 332 | 4.9E-96 |  |
| 633 | AB70_4983 | EZK04538 | type-1 fimbrial protein, A chain | Escherichia coli 1-176-05_S1_C3 | 332 | 4.9E-96 |  |
| 634 | BX75_23375 | EYY76122 | type-1 fimbrial protein subunit A | Escherichia coli O26:NM str. 2010C-4788 | 332 | 4.9E-96 |  |
| 635 | BX23_02230 | EZE49148 | type-1 fimbrial protein subunit A | Escherichia coli O118:H16 str. 2009C-4446 | 332 | 4.9E-96 |  |
| 636 | EC1303_c45060 | AJF59119 | major type 1 subunit fimbrin (pilin) | Escherichia coli 1303 | 332 | 4.9E-96 |  |
| 637 | EC2747800_4719 | EMW70281 | type-1 fimbrial protein, A chain | Escherichia coli 2747800 | 332 | 4.9E-96 |  |
| 638 | ECDEC6E_5032 | EHV68521 | fimbrial subunit type 1 | Escherichia coli DEC6E | 332 | 4.9E-96 |  |
| 639 | BY40_21450 | EZA68180 | type-1 fimbrial protein subunit A | Escherichia coli O157:H16 str. 98-3133 | 332 | 4.9E-96 |  |
| 640 | ECP030529313_4578 | END30665 | type-1 fimbrial protein, A chain | Escherichia coli p0305293.13 | 332 | 4.9E-96 |  |
| 641 | G965_04422 | EQY93498 | type-1 fimbrial protein, A chain | Escherichia coli UMEA 3318-1 | 332 | 4.9E-96 |  |
| 642 | EC960497_4780 | EIH32668 | type-1 fimbrial protein, A chain | Escherichia coli 96.0497 | 332 | 4.9E-96 |  |
| 643 | AE12_04071 | KDG73955 | type-1 fimbrial protein, A chain | Escherichia coli UCI 53 | 332 | 4.9E-96 |  |
| 644 | ECP03052936_4605 | ENH50960 | type-1 fimbrial protein, A chain | Escherichia coli p0305293.6 | 332 | 4.9E-96 |  |
| 645 | BU55_12150 | KDV39757 | type-1 fimbrial protein subunit A | Escherichia coli O146:H21 str. 2010C-3325 | 332 | 4.9E-96 |  |
| 646 | EC2749250_4763 | EMW64842 | type-1 fimbrial protein, A chain | Escherichia coli 2749250 | 332 | 4.9E-96 |  |
| 647 | AC22_0087 | KEL87435 | type-1 fimbrial protein, A chain | Escherichia coli 5-366-08_S3_C2 | 332 | 4.9E-96 |  |
| 648 | BU59_05365 | KDV32505 | type-1 fimbrial protein subunit A | Escherichia coli O69:H11 str. 07-3763 | 332 | 4.9E-96 |  |
| 649 | BX80_26560 | EZG69492 | type-1 fimbrial protein subunit A | Escherichia coli O26:H11 str. 2010C-4834 | 332 | 4.9E-96 |  |
| 650 | ECP030529311_4815 | ENG35149 | type-1 fimbrial protein, A chain | Escherichia coli p0305293.11 | 332 | 4.9E-96 |  |
| 651 | BX61_17490 | EZH56965 | type-1 fimbrial protein subunit A | Escherichia coli O26:H11 str. 2010C-4244 | 332 | 4.9E-96 |  |
| 652 | BY01_21045 | EZG97585 | type-1 fimbrial protein subunit A | Escherichia coli O26:H11 str. 2011C-3506 | 332 | 4.9E-96 |  |
| 653 | L339_00650 | ESV05724 | type 1 fimbriae major subunit FimA | Escherichia coli E1777 | 332 | 4.9E-96 |  |
| 654 | L340_3493 | EPH48412 | type 1 fimbriae major subunit FimA | Escherichia coli E2265 | 332 | 4.9E-96 |  |
| 655 | BX57_18755 | EZH53160 | type-1 fimbrial protein subunit A | Escherichia coli O26:H11 str. 2010C-3902 | 332 | 4.9E-96 |  |
| 656 | BX14_11935 | EZE22336 | type-1 fimbrial protein subunit A | Escherichia coli O45:H2 str. 2009C-3686 | 332 | 4.9E-96 |  |
| 657 | EC32303_4952 | EII75274 | type-1 fimbrial protein, A chain | Escherichia coli 3.2303 | 332 | 4.9E-96 |  |
| 658 | ECP030529315_4771 | ENG48118 | type-1 fimbrial protein, A chain | Escherichia coli p0305293.15 | 332 | 4.9E-96 |  |
| 659 | ECP03052935_4633 | ENH41239 | type-1 fimbrial protein, A chain | Escherichia coli p0305293.5 | 332 | 4.9E-96 |  |
| 660 | ECP03052933_4691 | ENG57226 | type-1 fimbrial protein, A chain | Escherichia coli p0305293.3 | 332 | 4.9E-96 |  |
| 661 | G845_04397 | EQT84572 | type-1 fimbrial protein, A chain | Escherichia coli HVH 193 (4-3331423) | 332 | 4.9E-96 |  |
| 662 | ECO10030_24284 | EJF01933 | major type 1 subunit fimbrin (pilin) FimA | Escherichia coli O26:H11 str. CVM10030 | 332 | 4.9E-96 |  |
| 663 | AB39_4827 | EZK13508 | type-1 fimbrial protein, A chain | Escherichia coli 1-176-05_S1_C2 | 332 | 4.9E-96 |  |
| 664 | BX86_16260 | EYY39718 | type-1 fimbrial protein subunit A | Escherichia coli O153:H2 str. 2010C-5034 | 332 | 4.9E-96 |  |
| 665 | ECTW00353_4781 | EKI21418 | type-1 fimbrial protein, A chain | Escherichia coli TW00353 | 332 | 4.9E-96 |  |
| 666 | BX63_09995 | EYU85231 | type-1 fimbrial protein subunit A | Escherichia coli O26:NM str. 2010C-4347 | 332 | 4.9E-96 |  |
| 667 | BW85_22985 | EYZ76905 | type-1 fimbrial protein subunit A | Escherichia coli O69:H11 str. 06-3325 | 332 | 4.9E-96 |  |
| 668 | BX96_24030 | EZG95719 | type-1 fimbrial protein subunit A | Escherichia coli O26:H11 str. 2011C-3282 | 332 | 4.9E-96 |  |
| 669 | AC20_4672 | KDY56893 | type-1 fimbrial protein, A chain | Escherichia coli 2-460-02_S3_C2 | 332 | 4.9E-96 |  |
| 670 | EC2756500_4972 | EMW55266 | type-1 fimbrial protein, A chain | Escherichia coli 2756500 | 332 | 4.9E-96 |  |
| 671 | ECDEC10D_5641 | EHW71887 | fimbrial subunit type 1 | Escherichia coli DEC10D | 332 | 4.9E-96 |  |
| 672 | WIA_00001 | ELI21484 | fimbrial protein FimA | Escherichia coli KTE109 | 332 | 4.9E-96 |  |
| 673 | A13U_00398 | ELH13128 | fimbrial protein FimA | Escherichia coli KTE192 | 332 | 4.9E-96 |  |
| 674 | BU58_07455 | KDV67600 | type-1 fimbrial protein subunit A | Escherichia coli O26:H11 str. 2011C-3274 | 332 | 4.9E-96 |  |
| 675 | AB51_4793 | KEM08009 | type-1 fimbrial protein, A chain | Escherichia coli 6-319-05_S1_C2 | 332 | 4.9E-96 |  |
| 676 | BU63_12685 | KDV73214 | type-1 fimbrial protein subunit A | Escherichia coli O118:H16 str. 07-4255 | 332 | 4.9E-96 |  |
| 677 | ECDEC11D_5083 | EHX04064 | type-1 fimbrial protein, A chain | Escherichia coli DEC11D | 332 | 4.9E-96 |  |
| 678 | ECP030529314_4855 | ENE04569 | type-1 fimbrial protein, A chain | Escherichia coli p0305293.14 | 332 | 4.9E-96 |  |
| 679 | BX12_03910 | EZE30434 | type-1 fimbrial protein subunit A | Escherichia coli O69:H11 str. 2009C-3601 | 332 | 4.9E-96 |  |
| 680 | BX40_01940 | EYV52386 | type-1 fimbrial protein subunit A | Escherichia coli O103:H11 str. 2010C-3214 | 332 | 4.9E-96 |  |
| 681 | ECENVIRA811_0030 | EMX78080 | type-1 fimbrial protein, A chain | Escherichia coli Envira 8/11 | 332 | 4.9E-96 |  |
| 682 | EC2770900_4686 | EMW46554 | type-1 fimbrial protein, A chain | Escherichia coli 2770900 | 332 | 4.9E-96 |  |
| 683 | BX28_02905 | EZH22405 | type-1 fimbrial protein subunit A | Escherichia coli O26:H11 str. 2009C-4760 | 332 | 4.9E-96 |  |
| 684 | BW74_09290 | EZA29638 | type-1 fimbrial protein subunit A | Escherichia coli O45:H2 str. 01-3147 | 332 | 4.9E-96 |  |
| 685 | BX17_14840 | EZH25748 | type-1 fimbrial protein subunit A | Escherichia coli O26:H11 str. 2009C-3996 | 332 | 4.9E-96 |  |
| 686 | ECO9942_09775 | EIL30300 | major type 1 subunit fimbrin (pilin) FimA | Escherichia coli O26:H11 str. CVM9942 | 332 | 4.9E-96 |  |
| 687 | BY08_12110 | EYX87044 | type-1 fimbrial protein subunit A | Escherichia coli O103:H2 str. 2011C-3750 | 332 | 4.9E-96 |  |
| 688 | BX08_06500 | EZE07535 | type-1 fimbrial protein subunit A | Escherichia coli O103:H2 str. 2009C-3279 | 332 | 4.9E-96 |  |
| 689 | ECP030529310_4631 | ENG25615 | type-1 fimbrial protein, A chain | Escherichia coli p0305293.10 | 332 | 4.9E-96 | Yes |
| 690 | G964_04338 | EQY81870 | type-1 fimbrial protein, A chain | Escherichia coli UMEA 3317-1 | 332 | 4.9E-96 |  |
| 691 | AB91_4670 | KDY50235 | type-1 fimbrial protein, A chain | Escherichia coli 2-460-02_S3_C1 | 332 | 4.9E-96 |  |
| 692 | ECDEC10A_0022 | EHW70237 | fimbrial subunit type 1 | Escherichia coli DEC10A | 332 | 4.9E-96 |  |
| 693 | BW81_21065 | EZG49255 | type-1 fimbrial protein subunit A | Escherichia coli O26:H11 str. 03-3500 | 332 | 4.9E-96 |  |
| 694 | DA88_22225 | KDM71056 | type-1 fimbrial protein subunit A | Escherichia coli O26:H11 (strain 11368 / EHEC) | 332 | 4.9E-96 |  |
| 695 | ECENVIRA101_0024 | EMX76334 | type-1 fimbrial protein, A chain | Escherichia coli Envira 10/1 | 332 | 4.9E-96 |  |
| 696 | ECO9450_23094 | EIL00500 | major type 1 subunit fimbrin (pilin) FimA | Escherichia coli O103:H2 str. CVM9450 | 332 | 4.9E-96 |  |
| 697 | EC2866350_4687 | ENB04001 | type-1 fimbrial protein, A chain | Escherichia coli 2866350 | 332 | 4.9E-96 |  |
| 698 | ECO10021_11227 | EJE78926 | major type 1 subunit fimbrin (pilin) FimA | Escherichia coli O26:H11 str. CVM10021 | 332 | 4.9E-96 |  |
| 699 | ECDEC8D_0002 | EHW31958 | fimbrial subunit type 1 | Escherichia coli DEC8D | 332 | 4.9E-96 |  |
| 700 | BW72_33420 | KDV18472 | type-1 fimbrial protein subunit A | Escherichia coli O78:H12 str. 00-3279 | 332 | 4.9E-96 |  |
| 701 | EC2720900_0034 | EMX96607 | type-1 fimbrial protein, A chain | Escherichia coli 2720900 | 332 | 4.9E-96 |  |
| 702 | BX64_14795 | EZG59608 | type-1 fimbrial protein subunit A | Escherichia coli O26:H11 str. 2010C-4430 | 332 | 4.9E-96 |  |
| 703 | ECO9534_17237 | EIL17770 | major type 1 subunit fimbrin (pilin) FimA | Escherichia coli O111:H11 str. CVM9534 | 332 | 4.9E-96 |  |
| 704 | ECEPECA14_0515 | EFZ43767 | fimbrial subunit type 1 | Escherichia coli EPECa14 | 332 | 4.9E-96 |  |
| 705 | BX38_24825 | EZH36775 | type-1 fimbrial protein subunit A | Escherichia coli O26:H11 str. 2010C-3051 | 332 | 4.9E-96 |  |
| 706 | EC2866550_4959 | EMV66030 | type-1 fimbrial protein, A chain | Escherichia coli 2866550 | 332 | 4.9E-96 |  |
| 707 | BX15_07570 | EZH15603 | type-1 fimbrial protein subunit A | Escherichia coli O26:H11 str. 2009C-3689 | 332 | 4.9E-96 |  |
| 708 | BW68_15625 | EZA44177 | type-1 fimbrial protein subunit A | Escherichia coli O26:H11 str. 05-3646 | 332 | 4.9E-96 |  |
| 709 | BW97_06240 | EYX08969 | type-1 fimbrial protein subunit A | Escherichia coli O69:H11 str. 07-4281 | 332 | 4.9E-96 |  |
| 710 | BX11_20545 | EZE27639 | type-1 fimbrial protein subunit A | Escherichia coli O123:H11 str. 2009C-3307 | 332 | 4.9E-96 |  |
| 711 | BX78_10725 | EZG64777 | type-1 fimbrial protein subunit A | Escherichia coli O26:H11 str. 2010C-4819 | 332 | 4.9E-96 |  |
| 712 | HMPREF9545_02692 | EFU57541 | fimbrial protein | Escherichia coli MS 16-3 | 332 | 4.9E-96 | Yes |
| 713 | ECSTECB2F1_4712 | EGW64659 | fimbrial subunit type 1 | Escherichia coli STEC_B2F1 | 332 | 4.9E-96 | Yes |
| 714 | EC32608_5490 | EIH56910 | type-1 fimbrial protein, A chain | Escherichia coli 3.2608 | 332 | 4.9E-96 |  |
| 715 | ECBCE034MS14_5192 | EMV15247 | type-1 fimbrial protein, A chain | Escherichia coli BCE034_MS-14 | 332 | 4.9E-96 |  |
| 716 | G711_04696 | ESP06154 | type-1 fimbrial protein, A chain | Escherichia coli HVH 36 (4-5675286) | 332 | 4.9E-96 |  |
| 717 | BX88_12120 | EZG83656 | type-1 fimbrial protein subunit A | Escherichia coli O26:H11 str. 2010EL-1699 | 332 | 4.9E-96 |  |
| 718 | ECDEC11E_4987 | EHX14660 | type-1 fimbrial protein, A chain | Escherichia coli DEC11E | 332 | 4.9E-96 |  |
| 719 | BX30_16775 | EZH30896 | type-1 fimbrial protein subunit A | Escherichia coli O26:H11 str. 2009C-4826 | 332 | 4.9E-96 |  |
| 720 | Q459_03850 | ETD59155 | type-1 fimbrial protein subunit A | Escherichia coli ATCC BAA-2215 | 332 | 4.9E-96 |  |
| 721 | EC2867750_4976 | EMV53826 | type-1 fimbrial protein, A chain | Escherichia coli 2867750 | 332 | 4.9E-96 |  |
| 722 | EC80566_4595 | EKK38123 | type-1 fimbrial major subunit | Escherichia coli 8.0566 | 332 | 4.9E-96 |  |
| 723 | ECEC1865_5753 | EKJ10633 | type-1 fimbrial protein, A chain | Escherichia coli EC1865 | 332 | 4.9E-96 |  |
| 724 | HMPREF1610_03973 | ESD66053 | type-1 fimbrial protein, A chain | Escherichia coli 908555 | 332 | 4.9E-96 |  |
| 725 | EC2853500_4877 | EMV96219 | type-1 fimbrial protein, A chain | Escherichia coli 2853500 | 332 | 4.9E-96 |  |
| 726 | AB49_4573 | KEK90913 | type-1 fimbrial protein, A chain | Escherichia coli 4-203-08_S1_C2 | 332 | 4.9E-96 |  |
| 727 | ECSTECS1191_0256 | EGX21680 | fimbrial subunit type 1 | Escherichia coli STEC_S1191 | 332 | 4.9E-96 |  |
| 728 | H003_04420 | ERA40511 | type-1 fimbrial protein, A chain | Escherichia coli UMEA 4076-1 | 332 | 4.9E-96 | Yes |
| 729 | WI1_04344 | ELJ90240 | fimbrial protein FimA | Escherichia coli KTE97 | 332 | 4.9E-96 |  |
| 730 | EC2875000_4920 | EMV33035 | type-1 fimbrial protein, A chain | Escherichia coli 2875000 | 332 | 4.9E-96 |  |
| 731 | BX98_04760 | EZG90712 | type-1 fimbrial protein subunit A | Escherichia coli O26:H11 str. 2011C-3387 | 332 | 4.9E-96 |  |
| 732 | ECDEC14C_5174 | EHX82511 | fimbrial subunit type 1 | Escherichia coli DEC14C | 332 | 4.9E-96 |  |
| 733 | AB21_4540 | KDU59967 | type-1 fimbrial protein, A chain | Escherichia coli 4-203-08_S1_C1 | 332 | 4.9E-96 |  |
| 734 | A13K_00350 | ELC76551 | type-1 fimbrial protein, A chain | Escherichia coli KTE187 | 332 | 4.9E-96 |  |
| 735 | BX55_20200 | EZH45600 | type-1 fimbrial protein subunit A | Escherichia coli O26:H11 str. 2010C-3871 | 332 | 4.9E-96 |  |
| 736 | EC40967_5579 | EII33915 | type-1 fimbrial protein, A chain | Escherichia coli 4.0967 | 332 | 4.9E-96 |  |
| 737 | ECO9455_01970 | EJE96834 | major type 1 subunit fimbrin (pilin) FimA | Escherichia coli O111:H11 str. CVM9455 | 332 | 4.9E-96 |  |
| 738 | ECO9545_16436 | EIL20787 | major type 1 subunit fimbrin (pilin) FimA | Escherichia coli O111:H11 str. CVM9545 | 332 | 4.9E-96 |  |
| 739 | BX29_09750 | EZE68855 | type-1 fimbrial protein subunit A | Escherichia coli O45:H2 str. 2009C-4780 | 332 | 4.9E-96 |  |
| 740 | ECO103_5095 | BAI33774 | major type 1 subunit fimbrin (pilin) FimA | Escherichia coli O103:H2 str. 12009 | 332 | 4.9E-96 | Yes |
| 741 | EC2735000_4827 | EMZ62170 | type-1 fimbrial protein, A chain | Escherichia coli 2735000 | 332 | 4.9E-96 |  |
| 742 | CFSAN001630_09015 | EKU05478 | major type 1 subunit fimbrin (pilin) FimA | Escherichia coli O111:H11 str. CFSAN001630 | 332 | 4.9E-96 |  |
| 743 | EC2860050_4809 | EMV87461 | type-1 fimbrial protein, A chain | Escherichia coli 2860050 | 332 | 4.9E-96 |  |
| 744 | AB11_5139 | EYD92537 | type-1 fimbrial protein, A chain | Escherichia coli 1-176-05_S1_C1 | 332 | 4.9E-96 |  |
| 745 | ECSTECH18_5453 | EGX01118 | fimbrial subunit type 1 | Escherichia coli STEC_H.1.8 | 332 | 4.9E-96 |  |
| 746 | ECO10224_00769 | EJE69706 | major type 1 subunit fimbrin (pilin) FimA | Escherichia coli O26:H11 str. CVM10224 | 332 | 4.9E-96 |  |
| 747 | ECDEC11C_5351 | EHX05904 | type-1 fimbrial protein, A chain | Escherichia coli DEC11C | 332 | 4.9E-96 |  |
| 748 | EC2874_20575 | KRR54439 | major type 1 subunit fimbrin (pilin) FimA | Escherichia coli VL2874 | 332 | 4.9E-96 | Yes |
| 749 | BX06_02275 | EZE12867 | type-1 fimbrial protein subunit A | Escherichia coli O69:H11 str. 08-4661 | 332 | 4.9E-96 |  |
| 750 | UWO_18500 | EIF16572 | major type 1 subunit fimbrin (pilin) FimA | Escherichia coli O32:H37 str. P4 | 332 | 4.9E-96 |  |
| 751 | ECC34666_5076 | EMV14129 | type-1 fimbrial protein, A chain | Escherichia coli C-34666 | 332 | 4.9E-96 |  |
| 752 | ECMP02155212_0265 | EMU72101 | type-1 fimbrial protein, A chain | Escherichia coli MP021552.12 | 332 | 4.9E-96 |  |
| 753 | AD16_0309 | KDU27784 | type-1 fimbrial protein, A chain | Escherichia coli 3-267-03_S4_C2 | 332 | 4.9E-96 |  |
| 754 | ECO9553_13124 | EJE88228 | major type 1 subunit fimbrin (pilin) FimA | Escherichia coli O111:H11 str. CVM9553 | 332 | 4.9E-96 |  |
| 755 | ECDEC14B_5244 | EHX73031 | fimbrial subunit type 1 | Escherichia coli DEC14B | 332 | 4.9E-96 |  |
| 756 | A1YO_00353 | ELG73923 | fimbrial protein FimA | Escherichia coli KTE136 | 332 | 4.9E-96 |  |
| 757 | AC49_4698 | KDY57203 | type-1 fimbrial protein, A chain | Escherichia coli 2-460-02_S3_C3 | 332 | 4.9E-96 |  |
| 758 | ECP03052937_4747 | ENH48663 | type-1 fimbrial protein, A chain | Escherichia coli p0305293.7 | 332 | 4.9E-96 |  |
| 759 | ECMP0215528_5211 | EMX32750 | type-1 fimbrial protein, A chain | Escherichia coli MP021552.8 | 332 | 4.9E-96 |  |
| 760 | BX13_23230 | EZH10147 | type-1 fimbrial protein subunit A | Escherichia coli O26:H11 str. 2009C-3612 | 332 | 4.9E-96 |  |
| 761 | BU53_15845 | KDV46141 | type-1 fimbrial protein subunit A | Escherichia coli O91:H21 str. 2009C-3740 | 332 | 4.9E-96 |  |
| 762 | ECDEC10B_0023 | EHW79985 | fimbrial subunit type 1 | Escherichia coli DEC10B | 332 | 4.9E-96 |  |
| 763 | EC180200_4703 | ENA60803 | type-1 fimbrial protein, A chain | Escherichia coli 180200 | 332 | 4.9E-96 |  |
| 764 | EcE22_2408 | EDV83264 | type-1 fimbrial protein homolog | Escherichia coli E22 | 332 | 4.9E-96 |  |
| 765 | ECDEC8C_0020 | EHW26527 | fimbrial subunit type 1 | Escherichia coli DEC8C | 332 | 4.9E-96 |  |
| 766 | ECO10026_29394 | EIL44113 | hypothetical protein | Escherichia coli O26:H11 str. CVM10026 | 332 | 4.9E-96 |  |
| 767 | UMNF18_5378 | AEJ59806 | fimbrial subunit type 1 | Escherichia coli UMNF18 | 332 | 4.9E-96 | Yes |
| 768 | BX41_24865 | EZH47251 | type-1 fimbrial protein subunit A | Escherichia coli O26:H11 str. 2010C-3472 | 332 | 4.9E-96 |  |
| 769 | ECDEC6D_5111 | EHV65429 | type-1 fimbrial protein, A chain | Escherichia coli DEC6D | 332 | 4.9E-96 |  |
| 770 | EC2866750_4932 | EMV69652 | type-1 fimbrial protein, A chain | Escherichia coli 2866750 | 332 | 4.9E-96 |  |
| 771 | EC2860650_4712 | ENA88532 | type-1 fimbrial protein, A chain | Escherichia coli 2860650 | 332 | 4.9E-96 |  |
| 772 | ECDEC6C_5135 | EHV52515 | type-1 fimbrial protein, A chain | Escherichia coli DEC6C | 332 | 4.9E-96 |  |
| 773 | ECP03052934_4737 | ENG57691 | type-1 fimbrial protein, A chain | Escherichia coli p0305293.4 | 332 | 4.9E-96 |  |
| 774 | ECO9952_02419 | EJF04536 | major type 1 subunit fimbrin (pilin) FimA | Escherichia coli O26:H11 str. CVM9952 | 332 | 4.9E-96 |  |
| 775 | ECP030481615_4787 | ENF42042 | type-1 fimbrial protein, A chain | Escherichia coli P0304816.15 | 331.9 | 5.2E-96 | Yes |
| 776 | SF274771_5018 | EGJ80848 | fimbrial subunit type 1 | Shigella flexneri 2747-71 | 331.7 | 6.1E-96 |  |
| 777 | SFK671_3941 | EGJ82496 | fimbrial subunit type 1 | Shigella flexneri K-671 | 331.7 | 6.1E-96 |  |
| 778 | SF293071_3871 | EGJ94926 | fimbrial subunit type 1 | Shigella flexneri 2930-71 | 331.7 | 6.1E-96 |  |
| 779 | SFK404_5377 | EIQ19704 | type-1 fimbrial protein, A chain | Shigella flexneri K-404 | 331.7 | 6.1E-96 |  |
| 780 | SFJ1713_5010 | EGM58768 | fimbrial subunit type 1 | Shigella flexneri J1713 | 331.7 | 6.1E-96 |  |
| 781 | SFV_4213 | ABF06196 | major type 1 subunit fimbrin (pilin) | Shigella flexneri 5 str. 8401 | 331.7 | 6.1E-96 | Yes |
| 782 | SFyv_6113 | AIL43532 | FimA | Shigella flexneri Shi06HN006 | 331.7 | 6.1E-96 |  |
| 783 | SFK218_5449 | EGK16143 | fimbrial subunit type 1 | Shigella flexneri K-218 | 331.7 | 6.1E-96 |  |
| 784 | SF660363_4846 | EJL09933 | fimbrial subunit type 1 | Shigella flexneri 6603-63 | 331.7 | 6.1E-96 |  |
| 785 | SF2A_22830 | AKK56605 | type-1 fimbrial protein subunit A | Shigella flexneri G1663 | 331.7 | 6.1E-96 |  |
| 786 | SFK304_5240 | EGK31444 | fimbrial subunit type 1 | Shigella flexneri K-304 | 331.7 | 6.1E-96 |  |
| 787 | SF4208 | AAN45628 | major type 1 subunit fimbrin (pilin) | Shigella flexneri 2a str. 301 | 331.7 | 6.1E-96 | Yes |
| 788 | SFK272_0234 | EGK30693 | fimbrial subunit type 1 | Shigella flexneri K-272 | 331.7 | 6.1E-96 |  |
| 789 | ERS574920_03325 | CEP58464 | major type 1 subunit fimbrin | Shigella flexneri 2a | 331.7 | 6.1E-96 |  |
| 790 | S4465 | AAP19413 | major type 1 subunit fimbrin (pilin) | Shigella flexneri 2a str. 2457T (GCA_000183785) | 331.7 | 6.1E-96 |  |
| 791 | SFxv_4591 | ADA76504 | Type-1 fimbrial major subunit | Shigella flexneri 2002017 | 331.7 | 6.1E-96 |  |
| 792 | SFy_6044 | AIL38595 | FimA | Shigella flexneri 2003036 | 331.7 | 6.1E-96 |  |
| 793 | SF434370_4562 | EGJ79816 | fimbrial subunit type 1 | Shigella flexneri 4343-70 | 331.7 | 6.1E-96 |  |
| 794 | SF285071_4086 | EIQ05195 | type-1 fimbrial protein, A chain | Shigella flexneri 2850-71 | 331.7 | 6.1E-96 |  |
| 795 | SF5M90T_4121 | EID64423 | major type 1 subunit fimbrin (pilin) | Shigella flexneri 5a str. M90T | 331.7 | 6.1E-96 |  |
| 796 | NCTC1_04574 | CDX09637 | major type 1 subunit fimbrin,Type-1A pilin,type-1 fimbrial protein subunit A,P pilus assembly protein, pilin FimA,Fimbrial protein | Shigella flexneri | 331.7 | 6.1E-96 |  |
| 797 | WQ71_08215 | KLH05460 | type-1 fimbrial protein subunit A | Escherichia coli (GCA_001012355) | 331.3 | 7.9E-96 | Yes |
| 798 | AC067_06225 | KOA35877 | type-1 fimbrial protein subunit A | Escherichia coli | 330.8 | 1.1E-95 |  |
| 799 | EC12741_5427 | EIG79635 | type-1 fimbrial protein, A chain | Escherichia coli 1.2741 | 330.8 | 1.1E-95 |  |
| 800 | ERJG_03163 | EGB60878 | fimbrial protein | Escherichia coli M863 | 330.8 | 1.1E-95 | Yes |
| 801 | ECSTEC7V_5027 | EGE61941 | fimbrial subunit type 1 | Escherichia coli STEC_7v | 330.8 | 1.1E-95 |  |
| 802 | G777_00482 | EQR06838 | fimbrial subunit type 1 | Escherichia coli HVH 115 (4-4465989) | 330.7 | 1.2E-95 |  |
| 803 | G776_04656 | EQQ96811 | fimbrial subunit type 1 | Escherichia coli HVH 115 (4-4465997) | 330.7 | 1.2E-95 | Yes |
| 804 | SFK227_5269 | EGK31557 | fimbrial subunit type 1 | Shigella flexneri K-227 | 330.5 | 1.4E-95 | Yes |
| 805 | ECP02994383_4881 | ENB93657 | type-1 fimbrial protein, A chain | Escherichia coli P0299438.3 | 330.4 | 1.5E-95 |  |
| 806 | ECP02994386_4889 | ENC09028 | type-1 fimbrial protein, A chain | Escherichia coli P0299438.6 | 330.4 | 1.5E-95 |  |
| 807 | ECP02994387_4949 | ENC09261 | type-1 fimbrial protein, A chain | Escherichia coli P0299438.7 | 330.4 | 1.5E-95 |  |
| 808 | ECP029943811_4899 | ENB91450 | type-1 fimbrial protein, A chain | Escherichia coli P0299438.11 | 330.4 | 1.5E-95 |  |
| 809 | ECP02994388_4883 | ENC18707 | type-1 fimbrial protein, A chain | Escherichia coli P0299438.8 | 330.4 | 1.5E-95 |  |
| 810 | ECP02994385_4975 | ENC05261 | type-1 fimbrial protein, A chain | Escherichia coli P0299438.5 | 330.4 | 1.5E-95 |  |
| 811 | ECMP0215612_0297 | EMX33697 | type-1 fimbrial protein, A chain | Escherichia coli MP021561.2 | 330.4 | 1.5E-95 | Yes |
| 812 | G861_04233 | EQU61546 | type-1 fimbrial protein, A chain | Escherichia coli HVH 209 (4-3062651) | 330.4 | 1.5E-95 |  |
| 813 | HMPREF9535_02846 | EFK73240 | fimbrial protein | Escherichia coli MS 78-1 | 330.4 | 1.5E-95 | Yes |
| 814 | EC2726950_4843 | ENA46046 | type-1 fimbrial protein, A chain | Escherichia coli 2726950 | 330.4 | 1.5E-95 | Yes |
| 815 | ERS085342_03211 | CTT76148 | fimbrial protein | Escherichia coli (GCA_001283725) | 330.1 | 1.8E-95 | Yes |
| 816 | G796_04494 | EQR88647 | type-1 fimbrial protein, A chain | Escherichia coli HVH 138 (4-6066704) | 330.1 | 1.8E-95 | Yes |
| 817 | A311_00409 | ELG92198 | fimbrial protein FimA | Escherichia coli KTE146 | 329.8 | 2.3E-95 | Yes |
| 818 | AD25_4672 | KDV76737 | type-1 fimbrial protein, A chain | Escherichia coli 2-052-05_S4_C3 | 329.7 | 2.4E-95 | Yes |
| 819 | HMPREF9349_04251 | EGU95824 | type-1 fimbrial protein, A chain | Escherichia coli MS 79-10 | 329.3 | 3.3E-95 |  |
| 820 | EC1999001_4732 | EMZ73467 | type-1 fimbrial protein, A chain | Escherichia coli 199900.1 | 329.3 | 3.3E-95 |  |
| 821 | ECP03047778_4620 | ENF04704 | type-1 fimbrial protein, A chain | Escherichia coli P0304777.8 | 329.3 | 3.3E-95 |  |
| 822 | ECP030477713_4752 | ENE60382 | type-1 fimbrial protein, A chain | Escherichia coli P0304777.13 | 329.3 | 3.3E-95 |  |
| 823 | ECP030477715_4759 | ENE71587 | type-1 fimbrial protein, A chain | Escherichia coli P0304777.15 | 329.3 | 3.3E-95 |  |
| 824 | AC33_4842 | KDT91192 | type-1 fimbrial protein, A chain | Escherichia coli 3-267-03_S3_C2 | 329.3 | 3.3E-95 |  |
| 825 | AE25_04216 | KDG93218 | type-1 fimbrial protein, A chain | Escherichia coli UCI 66 | 329.3 | 3.3E-95 |  |
| 826 | G759_04769 | ESJ98605 | type-1 fimbrial protein, A chain | Escherichia coli HVH 98 (4-5799287) | 329.3 | 3.3E-95 |  |
| 827 | WIC_04865 | ELI18351 | fimbrial protein FimA | Escherichia coli KTE112 | 329.3 | 3.3E-95 |  |
| 828 | HMPREF9350_02463 | EFU35807 | fimbrial protein | Escherichia coli MS 85-1 | 329.3 | 3.3E-95 |  |
| 829 | EC178200_4296 | ENG78418 | type-1 fimbrial protein, A chain | Escherichia coli 178200 | 329.3 | 3.3E-95 |  |
| 830 | WGU_00014 | ELJ87464 | fimbrial protein FimA | Escherichia coli KTE90 | 329.3 | 3.3E-95 |  |
| 831 | ECDG_02698 | EFF03495 | major type 1 subunit fimbrin | Escherichia coli B185 | 329.3 | 3.3E-95 | Yes |
| 832 | ECJURUA1811_4869 | EMX58829 | type-1 fimbrial protein, A chain | Escherichia coli Jurua 18/11 | 329.3 | 3.3E-95 |  |
| 833 | HMPREF9536_03307 | EFJ86407 | fimbrial protein | Escherichia coli MS 84-1 | 329.3 | 3.3E-95 |  |
| 834 | A1WA_04477 | ELG38550 | fimbrial protein FimA | Escherichia coli KTE91 | 329.3 | 3.3E-95 |  |
| 835 | EcSMS35_4840 | ACB18236 | type-1 fimbrial protein homolog | Escherichia coli SMS-3-5 | 329.3 | 3.3E-95 | Yes |
| 836 | ECP030477714_4791 | ENE64846 | type-1 fimbrial protein, A chain | Escherichia coli P0304777.14 | 329.3 | 3.3E-95 |  |
| 837 | ECP030477712_4786 | ENE59539 | type-1 fimbrial protein, A chain | Escherichia coli P0304777.12 | 329.3 | 3.3E-95 |  |
| 838 | A13E_01198 | ELH37517 | fimbrial protein FimA | Escherichia coli KTE184 | 329.3 | 3.3E-95 |  |
| 839 | ECP030477711_4737 | ENE51894 | type-1 fimbrial protein, A chain | Escherichia coli P0304777.11 | 329.3 | 3.3E-95 |  |
| 840 | G783_04758 | EQR30146 | type-1 fimbrial protein, A chain | Escherichia coli HVH 121 (4-6877826) | 329.3 | 3.3E-95 |  |
| 841 | G709_00499 | EQO36897 | type-1 fimbrial protein, A chain | Escherichia coli HVH 33 (4-2174936) | 329.3 | 3.3E-95 |  |
| 842 | ECP03047777_4730 | ENE96906 | type-1 fimbrial protein, A chain | Escherichia coli P0304777.7 | 329.3 | 3.3E-95 |  |
| 843 | APT99_17415 | KSY18426 | type-1 fimbrial protein subunit A | Escherichia coli (strain SMS-3-5 / SECEC) | 329.3 | 3.3E-95 |  |
| 844 | ECP03047773_4767 | ENE82455 | type-1 fimbrial protein, A chain | Escherichia coli P0304777.3 | 329.3 | 3.3E-95 |  |
| 845 | ECP03047774_5152 | ENE89101 | type-1 fimbrial protein, A chain | Escherichia coli P0304777.4 | 329.3 | 3.3E-95 |  |
| 846 | ECP03047775_4541 | ENE91958 | type-1 fimbrial protein, A chain | Escherichia coli P0304777.5 | 329.3 | 3.3E-95 | Yes |
| 847 | ECP03047779_4731 | ENF07495 | type-1 fimbrial protein, A chain | Escherichia coli P0304777.9 | 329.3 | 3.3E-95 |  |
| 848 | HMPREF1608_03717 | ESD66974 | type-1 fimbrial protein, A chain | Escherichia coli 908525 | 329.3 | 3.3E-95 |  |
| 849 | ECP030477710_4697 | ENE40686 | type-1 fimbrial protein, A chain | Escherichia coli P0304777.10 | 329.3 | 3.3E-95 |  |
| 850 | HMPREF1595_03990 | ESD04895 | type-1 fimbrial protein, A chain | Escherichia coli 907672 | 329.3 | 3.3E-95 |  |
| 851 | ECP03047772_4714 | ENE74106 | type-1 fimbrial protein, A chain | Escherichia coli P0304777.2 | 329.3 | 3.3E-95 |  |
| 852 | HMPREF9347_03724 | EFK67374 | fimbrial protein | Escherichia coli MS 124-1 | 329.3 | 3.3E-95 |  |
| 853 | A1S5_00741 | ELG02224 | fimbrial protein FimA | Escherichia coli KTE48 | 329.3 | 3.3E-95 |  |
| 854 | HMPREF9345_03567 | EFK49955 | fimbrial protein | Escherichia coli MS 107-1 | 329.3 | 3.3E-95 |  |
| 855 | ECP03047771_4667 | EMW95229 | type-1 fimbrial protein, A chain | Escherichia coli P0304777.1 | 329.3 | 3.3E-95 |  |
| 856 | A13W_03650 | ELC92562 | type-1 fimbrial protein, A chain | Escherichia coli KTE193 | 329.3 | 3.3E-95 |  |
| 857 | G725_04892 | EQO90974 | type-1 fimbrial protein, A chain | Escherichia coli HVH 53 (4-0631051) | 328.8 | 4.7E-95 |  |
| 858 | H000_04159 | ERA29482 | type-1 fimbrial protein, A chain | Escherichia coli UMEA 3899-1 | 328.8 | 4.7E-95 |  |
| 859 | G839_03864 | EQT55713 | type-1 fimbrial protein, A chain | Escherichia coli HVH 187 (4-4471660) | 328.8 | 4.7E-95 |  |
| 860 | ECIAI39_4787 | CAR20884 | major type 1 subunit fimbrin (pilin) | Escherichia coli IAI39 | 328.8 | 4.7E-95 |  |
| 861 | CE10_5058 | AEQ15720 | major type 1 subunit fimbrin (pilin) | Escherichia coli O7:K1 str. CE10 | 328.8 | 4.7E-95 | Yes |
| 862 | SFVA6_0197 | EGK29837 | fimbrial subunit type 1 | Shigella flexneri VA-6 | 328 | 8.4E-95 | Yes |
| 863 | SFK1770_0194 | EIQ20882 | type-1 fimbrial protein, A chain | Shigella flexneri K-1770 | 328 | 8.4E-95 |  |
| 864 | G721_04518 | EQO77490 | type-1 fimbrial protein, A chain | Escherichia coli HVH 46 (4-2758776) | 327.9 | 8.9E-95 | Yes |
| 865 | G811_04673 | EQS44718 | type-1 fimbrial protein, A chain | Escherichia coli HVH 153 (3-9344314) | 327.9 | 8.9E-95 |  |
| 866 | G923_04698 | EQX13146 | type-1 fimbrial protein, A chain | Escherichia coli UMEA 3160-1 | 327.9 | 8.9E-95 |  |
| 867 | ERYG_00371 | KNA43562 | fimbrial protein | Escherichia coli M114 | 327.8 | 9.3E-95 | Yes |
| 868 | G852_04867 | EQU18338 | type-1 fimbrial protein, A chain | Escherichia coli HVH 200 (4-4449924) | 327.6 | 1.1E-94 | Yes |
| 869 | SF123566_7378 | EIQ52878 | type-1 fimbrial protein, A chain | Shigella flexneri 1235-66 | 327.5 | 1.2E-94 | Yes |
| 870 | EC180600_4725 | EMW73178 | type-1 fimbrial protein, A chain | Escherichia coli 180600 | 327.4 | 1.2E-94 | Yes |
| 871 | AM273_02115 | KQJ50894 | type-1 fimbrial protein subunit A | Escherichia coli (GCA_001420165) | 327.3 | 1.3E-94 | Yes |
| 872 | AB67_5011 | KEJ70454 | type-1 fimbrial protein, A chain | Escherichia coli 5-366-08_S1_C3 | 327.2 | 1.5E-94 |  |
| 873 | AB08_5594 | KEL57133 | type-1 fimbrial protein, A chain | Escherichia coli 5-366-08_S1_C1 | 327.2 | 1.5E-94 |  |
| 874 | G654_03772 | EWC57542 | fimbrial protein | Escherichia coli EC096/10 | 327.2 | 1.5E-94 |  |
| 875 | A313_03074 | ELG89861 | fimbrial protein FimA | Escherichia coli KTE147 | 327.2 | 1.5E-94 |  |
| 876 | A1YY_04150 | ELG79325 | fimbrial protein FimA | Escherichia coli KTE144 | 327.2 | 1.5E-94 |  |
| 877 | A1U5_00241 | ELE46150 | type-1 fimbrial protein, A chain | Escherichia coli KTE66 | 327.2 | 1.5E-94 | Yes |
| 878 | ECNG_01383 | EGI42629 | type-1 fimbrial protein, A chain | Escherichia coli TA280 | 326.6 | 2.1E-94 | Yes |
| 879 | CDL30121 | CDL30121 | type 1 fimbriae major subunit FimA | Escherichia coli ISC7 | 326.4 | 2.5E-94 | Yes |
| 880 | AD12_4987 | EZJ32456 | type-1 fimbrial protein, A chain | Escherichia coli 1-392-07_S4_C2 | 326.4 | 2.6E-94 |  |
| 881 | PU31_20260 | KHI41588 | type-1 fimbrial protein subunit A | Escherichia coli (GCA_000797775) | 326.3 | 2.6E-94 | Yes |
| 882 | G903_04598 | EQW36494 | type-1 fimbrial protein, A chain | Escherichia coli UMEA 3053-1 | 326 | 3.3E-94 |  |
| 883 | G727_04797 | EQO93564 | type-1 fimbrial protein, A chain | Escherichia coli HVH 55 (4-2646161) | 326 | 3.3E-94 | Yes |
| 884 | BX46_11020 | EYV44989 | type-1 fimbrial protein subunit A | Escherichia coli O145:NM str. 2010C-3511 | 325.8 | 3.8E-94 |  |
| 885 | BX50_13340 | EYV16915 | type-1 fimbrial protein subunit A | Escherichia coli O145:NM str. 2010C-3521 | 325.8 | 3.8E-94 |  |
| 886 | BW87_04370 | EYZ76651 | type-1 fimbrial protein subunit A | Escherichia coli O145:NM str. 06-3484 | 325.8 | 3.8E-94 |  |
| 887 | ECRM13514_5557 | AHG12173 | type 1 fimbriae major subunit FimA | Escherichia coli O145:H28 str. RM13514 | 325.8 | 3.8E-94 | Yes |
| 888 | BX48_09440 | EYV22672 | type-1 fimbrial protein subunit A | Escherichia coli O145:NM str. 2010C-3517 | 325.8 | 3.8E-94 |  |
| 889 | BX02_26900 | EYW91335 | type-1 fimbrial protein subunit A | Escherichia coli O145:NM str. 08-4270 | 325.8 | 3.8E-94 |  |
| 890 | BX42_05835 | EYV57492 | type-1 fimbrial protein subunit A | Escherichia coli O145:NM str. 2010C-3507 | 325.8 | 3.8E-94 |  |
| 891 | BX43_26700 | EZE91450 | type-1 fimbrial protein subunit A | Escherichia coli O145:NM str. 2010C-3508 | 325.8 | 3.8E-94 |  |
| 892 | BX44_01770 | EYV38378 | type-1 fimbrial protein subunit A | Escherichia coli O145:NM str. 2010C-3509 | 325.8 | 3.8E-94 |  |
| 893 | BX49_04010 | EYV18154 | type-1 fimbrial protein subunit A | Escherichia coli O145:NM str. 2010C-3518 | 325.8 | 3.8E-94 |  |
| 894 | ECRM12581_27310 | AHY73992 | type 1 fimbriae major subunit FimA | Escherichia coli O145:H28 str. RM12581 | 325.8 | 3.8E-94 |  |
| 895 | BX51_00380 | EYV20065 | type-1 fimbrial protein subunit A | Escherichia coli O145:NM str. 2010C-3526 | 325.8 | 3.8E-94 |  |
| 896 | DC23_14395 | KDM77231 | type-1 fimbrial protein subunit A | Escherichia coli O145:H28 str. 4865/96 | 325.8 | 3.8E-94 |  |
| 897 | BX45_00390 | EYV46004 | type-1 fimbrial protein subunit A | Escherichia coli O145:NM str. 2010C-3510 | 325.8 | 3.8E-94 |  |
| 898 | O199_0202260 | ETJ70869 | type-1 fimbrial protein subunit A | Escherichia coli ATCC 35150 | 325.8 | 3.8E-94 |  |
| 899 | WR02_17525 | KNF47131 | type-1 fimbrial protein subunit A | Escherichia coli | 325.8 | 3.8E-94 |  |
| 900 | BX47_26880 | EYV24888 | type-1 fimbrial protein subunit A | Escherichia coli O145:NM str. 2010C-3516 | 325.8 | 3.8E-94 |  |
| 901 | BX67_25105 | EYZ21902 | type-1 fimbrial protein subunit A | Escherichia coli O145:NM str. 2010C-4557C2 | 325.8 | 3.8E-94 |  |
| 902 | PU13_21255 | KIH19917 | type-1 fimbrial protein subunit A | Escherichia coli (GCA_000819365) | 325.8 | 3.8E-94 |  |
| 903 | AU10_11095 | EZG31898 | type-1 fimbrial protein subunit A | Escherichia coli E1728 | 325.8 | 3.8E-94 | Yes |
| 904 | ESRG_01613 | EHN87315 | type-1 fimbrial protein, A chain | Escherichia coli TA124 | 325.6 | 4.4E-94 | Yes |
| 905 | G959_04570 | ESK11753 | type-1 fimbrial protein, A chain | Escherichia coli UMEA 3290-1 | 325.5 | 4.8E-94 |  |
| 906 | G977_04844 | EQZ23809 | type-1 fimbrial protein, A chain | Escherichia coli UMEA 3585-1 | 325.5 | 4.8E-94 |  |
| 907 | AD23_4865 | EZJ33439 | type-1 fimbrial protein, A chain | Escherichia coli 2-005-03_S4_C3 | 325.5 | 4.8E-94 |  |
| 908 | ECP_4649 | ABG72585 | type-1 fimbrial major subunit | Escherichia coli 536 | 325.5 | 4.8E-94 | Yes |
| 909 | G907_04446 | EQW50319 | type-1 fimbrial protein, A chain | Escherichia coli UMEA 3097-1 | 325.5 | 4.8E-94 |  |
| 910 | G975_03699 | ETF33541 | type-1 fimbrial protein, A chain | Escherichia coli UMEA 3489-1 | 325.5 | 4.8E-94 |  |
| 911 | G909_04626 | EQW60038 | type-1 fimbrial protein, A chain | Escherichia coli UMEA 3113-1 | 325.5 | 4.8E-94 |  |
| 912 | AC67_4940 | KDT24186 | type-1 fimbrial protein, A chain | Escherichia coli 2-052-05_S4_C1 | 325.5 | 4.8E-94 |  |
| 913 | ECED1_5199 | CAR11152 | major type 1 subunit fimbrin (pilin) | Escherichia coli ED1a | 325.5 | 4.8E-94 |  |
| 914 | HMPREF9553_04096 | EFJ59822 | type-1 fimbrial protein, A chain | Escherichia coli MS 200-1 | 325.5 | 4.8E-94 |  |
| 915 | EcF11_0640 | EDV68690 | type-1 fimbrial protein homolog | Escherichia coli F11 | 325.5 | 4.8E-94 |  |
| 916 | A13I_02520 | EOX26464 | fimbrial protein FimA | Escherichia coli KTE186 | 325.5 | 4.8E-94 |  |
| 917 | A13G_00244 | EOX26800 | fimbrial protein FimA | Escherichia coli KTE185 | 325.5 | 4.8E-94 |  |
| 918 | G704_04771 | EQO01212 | type-1 fimbrial protein, A chain | Escherichia coli HVH 28 (4-0907367) | 325.5 | 4.8E-94 |  |
| 919 | DO98_14900 | KDO87768 | type-1 fimbrial protein subunit A | Escherichia coli O81 (strain ED1a) | 325.5 | 4.8E-94 |  |
| 920 | AC93_4709 | EZJ47425 | type-1 fimbrial protein, A chain | Escherichia coli 2-005-03_S4_C2 | 325.5 | 4.8E-94 |  |
| 921 | G888_04546 | EQV60904 | type-1 fimbrial protein, A chain | Escherichia coli KOEGE 58 (171a) | 325.5 | 4.8E-94 |  |
| 922 | BW75_08270 | EZA15053 | type-1 fimbrial protein subunit A | Escherichia coli O81:NM str. 02-3012 | 325.5 | 4.8E-94 |  |
| 923 | G892_04591 | EQV75843 | type-1 fimbrial protein, A chain | Escherichia coli KOEGE 70 (185a) | 325.5 | 4.8E-94 |  |
| 924 | G881_04712 | EQV27991 | type-1 fimbrial protein, A chain | Escherichia coli KOEGE 30 (63a) | 325.5 | 4.8E-94 |  |
| 925 | G773_04502 | EQQ80591 | type-1 fimbrial protein, A chain | Escherichia coli HVH 112 (4-5987253) | 325.5 | 4.8E-94 |  |
| 926 | G761_01032 | EQQ43247 | type-1 fimbrial protein, A chain | Escherichia coli HVH 100 (4-2850729) | 325.5 | 4.8E-94 |  |
| 927 | WIU_04544 | ELI61780 | fimbrial protein FimA | Escherichia coli KTE131 | 325.5 | 4.8E-94 |  |
| 928 | G946_04361 | EQY23631 | type-1 fimbrial protein, A chain | Escherichia coli UMEA 3217-1 | 325.5 | 4.8E-94 |  |
| 929 | G980_04557 | EQZ33058 | type-1 fimbrial protein, A chain | Escherichia coli UMEA 3617-1 | 325.5 | 4.8E-94 |  |
| 930 | HMPREF1622_05062 | ESE27389 | type-1 fimbrial protein, A chain | Escherichia coli A35218R | 325.5 | 4.8E-94 |  |
| 931 | WGW_00010 | ELJ90791 | fimbrial protein FimA | Escherichia coli KTE94 | 325.5 | 4.8E-94 |  |
| 932 | WCI_04698 | ELF43356 | fimbrial protein FimA | Escherichia coli KTE8 | 325.5 | 4.8E-94 |  |
| 933 | G974_00407 | ESK21404 | type-1 fimbrial protein, A chain | Escherichia coli UMEA 3426-1 | 325.5 | 4.8E-94 |  |
| 934 | A15M_00061 | ELD17298 | type-1 fimbrial protein, A chain | Escherichia coli KTE206 | 325.5 | 4.8E-94 |  |
| 935 | G837_04722 | EQT43955 | type-1 fimbrial protein, A chain | Escherichia coli HVH 185 (4-2876639) | 325.5 | 4.8E-94 |  |
| 936 | G728_04396 | EQP02767 | type-1 fimbrial protein, A chain | Escherichia coli HVH 56 (4-2153033) | 325.5 | 4.8E-94 |  |
| 937 | G899_04631 | EQW12604 | type-1 fimbrial protein, A chain | Escherichia coli UMEA 3022-1 | 325.5 | 4.8E-94 |  |
| 938 | HMPREF9533_03087 | EGB82102 | type-1 fimbrial protein, A chain | Escherichia coli MS 60-1 | 325.5 | 4.8E-94 |  |
| 939 | ECEG_02927 | EFF14732 | hypothetical protein | Escherichia coli B354 | 324.8 | 8E-94 | Yes |
| 940 | AE16_04220 | KDG81430 | fimbrial subunit type 1 | Escherichia coli UCI 57 | 324.6 | 8.9E-94 | Yes |
| 941 | AE17_03594 | KDG84293 | fimbrial subunit type 1 | Escherichia coli UCI 58 | 324.6 | 8.9E-94 |  |
| 942 | AD47_4830 | KEM55079 | type-1 fimbrial protein, A chain | Escherichia coli 6-319-05_S4_C3 | 324.5 | 9.5E-94 |  |
| 943 | C4893_41760 | EMR92139 | major type 1 subunit fimbrin (pilin) | Escherichia coli ONT:H33 str. C48/93 | 324.5 | 1E-93 | Yes |
| 944 | A1UQ_00295 | ELE66674 | type-1 fimbrial protein, A chain | Escherichia coli KTE77 | 324.3 | 1.1E-93 | Yes |
| 945 | ACU58_24685 | KPO16423 | type-1 fimbrial protein subunit A | Escherichia coli (GCA_001309535) | 324.3 | 1.1E-93 |  |
| 946 | AB86_4908 | KDW11729 | type-1 fimbrial protein, A chain | Escherichia coli 2-177-06_S3_C1 | 324 | 1.4E-93 |  |
| 947 | WQ72_07890 | KLH18415 | type-1 fimbrial protein subunit A | Escherichia coli | 323.9 | 1.5E-93 |  |
| 948 | AB85_4855 | KDV97287 | type-1 fimbrial protein, A chain | Escherichia coli 2-156-04_S3_C1 | 323.9 | 1.5E-93 |  |
| 949 | BX10_01835 | EZE16030 | type-1 fimbrial protein subunit A | Escherichia coli O121:H7 str. 2009C-3299 | 323.9 | 1.5E-93 | Yes |
| 950 | ECBCE002MS12_4672 | EMV29153 | type-1 fimbrial protein, A chain | Escherichia coli BCE002_MS12 | 323.9 | 1.5E-93 | Yes |
| 951 | ECBCE001MS16_4689 | EMX83911 | type-1 fimbrial protein, A chain | Escherichia coli BCE001_MS16 | 323.9 | 1.5E-93 |  |
| 952 | MC63_22765 | KGY83486 | type-1 fimbrial protein subunit A | Shigella flexneri (GCA_000783735) | 323.8 | 1.6E-93 | Yes |
| 953 | G716_04680 | EQO53407 | type-1 fimbrial protein, A chain | Escherichia coli HVH 41 (4-2677849) | 323.7 | 1.8E-93 |  |
| 954 | WK5_00001 | ELI92888 | fimbrial protein FimA | Escherichia coli KTE145 | 323.7 | 1.8E-93 |  |
| 955 | G772_04508 | EQQ70520 | type-1 fimbrial protein, A chain | Escherichia coli HVH 111 (4-7039018) | 323.7 | 1.8E-93 |  |
| 956 | AC80_5196 | EYD96373 | type-1 fimbrial protein, A chain | Escherichia coli 1-110-08_S4_C1 | 323.7 | 1.8E-93 |  |
| 957 | G936_04815 | EQX71417 | type-1 fimbrial protein, A chain | Escherichia coli UMEA 3193-1 | 323.7 | 1.8E-93 |  |
| 958 | G988_04400 | ESK23466 | type-1 fimbrial protein, A chain | Escherichia coli UMEA 3693-1 | 323.7 | 1.8E-93 |  |
| 959 | CDK52690 | CDK52690 | type 1 fimbriae major subunit FimA | Escherichia coli IS5 | 323.7 | 1.8E-93 |  |
| 960 | CDK45226 | CDK45226 | type 1 fimbriae major subunit FimA | Escherichia coli IS1 | 323.7 | 1.8E-93 |  |
| 961 | A13S_00398 | ELC93703 | type-1 fimbrial protein, A chain | Escherichia coli KTE191 | 323.7 | 1.8E-93 |  |
| 962 | E2348C_4621 | CAS12169 | major type 1 subunit fimbrin (pilin) | Escherichia coli O127:H6 str. E2348/69 | 323.7 | 1.8E-93 | Yes |
| 963 | A13C_03586 | ELH36553 | fimbrial protein FimA | Escherichia coli KTE183 | 323.7 | 1.8E-93 |  |
| 964 | WCU_04647 | ELC23588 | type-1 fimbrial protein, A chain | Escherichia coli KTE15 | 323.7 | 1.8E-93 | Yes |
| 965 | WI9_04456 | ELI04428 | fimbrial protein FimA | Escherichia coli KTE106 | 323.7 | 1.8E-93 |  |
| 966 | G877_04634 | ERA85982 | type-1 fimbrial protein, A chain | Escherichia coli HVH 228 (4-7787030) | 323.7 | 1.8E-93 |  |
| 967 | A1UO_04712 | ELE54289 | type-1 fimbrial protein, A chain | Escherichia coli KTE76 | 323.7 | 1.8E-93 |  |
| 968 | AF56_04385 | EZQ55258 | type-1 fimbrial protein, A chain | Escherichia coli BIDMC 83 | 323.7 | 1.8E-93 |  |
| 969 | H001_04564 | ERA28700 | type-1 fimbrial protein, A chain | Escherichia coli UMEA 3955-1 | 323.7 | 1.8E-93 |  |
| 970 | A1UG_04867 | ELE46755 | type-1 fimbrial protein, A chain | Escherichia coli KTE72 | 323.7 | 1.8E-93 |  |
| 971 | G973_04623 | EQZ11168 | type-1 fimbrial protein, A chain | Escherichia coli UMEA 3391-1 | 323.7 | 1.8E-93 |  |
| 972 | G991_04526 | EQZ84863 | type-1 fimbrial protein, A chain | Escherichia coli UMEA 3703-1 | 323.7 | 1.8E-93 |  |
| 973 | WI5_04532 | ELI01597 | fimbrial protein FimA | Escherichia coli KTE104 | 323.7 | 1.8E-93 |  |
| 974 | APU06_19695 | KSY57322 | type-1 fimbrial protein subunit A | Escherichia coli HVH 41 (4-2677849) | 323.7 | 1.8E-93 |  |
| 975 | AB14_4661 | KDW70397 | type-1 fimbrial protein, A chain | Escherichia coli 1-392-07_S1_C1 | 323.7 | 1.8E-93 |  |
| 976 | A1W9_04483 | EOV85032 | fimbrial protein FimA | Escherichia coli KTE89 | 323.7 | 1.8E-93 |  |
| 977 | G900_03785 | EQW24842 | type-1 fimbrial protein, A chain | Escherichia coli UMEA 3033-1 | 323.7 | 1.8E-93 |  |
| 978 | WE5_03968 | EOU59196 | fimbrial protein FimA | Escherichia coli KTE19 | 323.7 | 1.8E-93 |  |
| 979 | A13O_00074 | ELC87049 | type-1 fimbrial protein, A chain | Escherichia coli KTE189 | 323.7 | 1.8E-93 |  |
| 980 | G983_04256 | EQZ47986 | type-1 fimbrial protein, A chain | Escherichia coli UMEA 3656-1 | 323.7 | 1.8E-93 |  |
| 981 | H002_04791 | ERA28384 | type-1 fimbrial protein, A chain | Escherichia coli UMEA 4075-1 | 323.7 | 1.8E-93 |  |
| 982 | V415_14915 | ETE21308 | type 1 fimbrial protein | Escherichia coli LAU-EC10 | 323.7 | 1.8E-93 |  |
| 983 | AB42_4423 | KDW79813 | type-1 fimbrial protein, A chain | Escherichia coli 1-392-07_S1_C2 | 323.7 | 1.8E-93 |  |
| 984 | G953_04594 | EQY50158 | type-1 fimbrial protein, A chain | Escherichia coli UMEA 3244-1 | 323.7 | 1.8E-93 |  |
| 985 | T654_04028 | EYT05003 | type-1 fimbrial protein, A chain | Escherichia coli K02 | 323.7 | 1.8E-93 |  |
| 986 | A1W5_00057 | ELE85055 | type-1 fimbrial protein, A chain | Escherichia coli KTE86 | 323.7 | 1.8E-93 |  |
| 987 | G920_04329 | EQW97660 | type-1 fimbrial protein, A chain | Escherichia coli UMEA 3152-1 | 323.7 | 1.8E-93 |  |
| 988 | WGS_04328 | ELJ64309 | fimbrial protein FimA | Escherichia coli KTE88 | 323.7 | 1.8E-93 |  |
| 989 | L913_3478 | ESA25201 | type 1 fimbriae major subunit FimA | Escherichia coli SCD2 | 323.7 | 1.8E-93 |  |
| 990 | AB50_4952 | KEJ04605 | type-1 fimbrial protein, A chain | Escherichia coli 6-175-07_S1_C2 | 323.7 | 1.8E-93 |  |
| 991 | EcoM_02393 | EFW69885 | type 1 fimbriae major subunit FimA | Escherichia coli WV_060327 | 323.7 | 1.8E-93 |  |
| 992 | A1WE_00058 | ELE94949 | type-1 fimbrial protein, A chain | Escherichia coli KTE93 | 323.7 | 1.8E-93 |  |
| 993 | AB79_5081 | KEM48570 | type-1 fimbrial protein, A chain | Escherichia coli 6-175-07_S1_C3 | 323.7 | 1.8E-93 |  |
| 994 | A175_00077 | ELH86317 | fimbrial protein FimA | Escherichia coli KTE215 | 323.7 | 1.8E-93 |  |
| 995 | K427_27075 | EWY51439 | type-1 fimbrial protein subunit A | Escherichia coli MP1 | 323.7 | 1.8E-93 |  |
| 996 | G826_04531 | EQS94728 | type-1 fimbrial protein, A chain | Escherichia coli HVH 171 (4-3191958) | 323.7 | 1.8E-93 |  |
| 997 | HQ24_22200 | AIF39558 | type-1 fimbrial protein subunit A | Escherichia coli KLY | 323.1 | 2.5E-93 |  |
| 998 | G858_04769 | EQU46076 | type-1 fimbrial protein, A chain | Escherichia coli HVH 206 (4-3128229) | 323.1 | 2.5E-93 |  |
| 999 | ECGG_03264 | EFF02900 | type 1 fimbriae major subunit FimA | Escherichia coli FVEC1412 | 323.1 | 2.5E-93 |  |
| 1000 | A139_04424 | ELC68643 | type-1 fimbrial protein, A chain | Escherichia coli KTE181 | 323.1 | 2.5E-93 |  |
| 1001 | IAE_10064 | EGU27029 | major type 1 subunit fimbrin (pilin) | Escherichia coli XH140A | 323.1 | 2.5E-93 |  |
| 1002 | G894_04451 | EQV93955 | type-1 fimbrial protein, A chain | Escherichia coli KOEGE 73 (195a) | 323.1 | 2.5E-93 |  |
| 1003 | G689_04587 | EQN36950 | type-1 fimbrial protein, A chain | Escherichia coli HVH 10 (4-6832164) | 323.1 | 2.5E-93 |  |
| 1004 | AB69_4871 | EYE31516 | type-1 fimbrial protein, A chain | Escherichia coli 1-110-08_S1_C3 | 323.1 | 2.5E-93 |  |
| 1005 | ECCZ_01870 | EST65320 | major type 1 subunit fimbrin (pilin) | Escherichia coli ECC-Z | 323.1 | 2.5E-93 |  |
| 1006 | A1SK_02379 | ELE13874 | type-1 fimbrial protein, A chain | Escherichia coli KTE56 | 323.1 | 2.5E-93 |  |
| 1007 | EC54115_03957 | EIL58003 | major type 1 subunit fimbrin (pilin) | Escherichia coli 541-15 | 323.1 | 2.5E-93 |  |
| 1008 | G967_04576 | EQY93834 | type-1 fimbrial protein, A chain | Escherichia coli UMEA 3329-1 | 323.1 | 2.5E-93 |  |
| 1009 | HMPREF1599_04106 | ESA83518 | type-1 fimbrial protein, A chain | Escherichia coli 907713 | 323.1 | 2.5E-93 |  |
| 1010 | WII_04737 | ELI33515 | fimbrial protein FimA | Escherichia coli KTE120 | 323.1 | 2.5E-93 |  |
| 1011 | L444_09134 | ETY20381 | type-1 fimbrial protein, A chain | Escherichia coli BIDMC 15 | 323.1 | 2.5E-93 |  |
| 1012 | PGC_10080 | CDP75627 | Putative uncharacterized protein | Escherichia coli D6-117.29 | 323.1 | 2.5E-93 |  |
| 1013 | G828_04747 | EQT06488 | type-1 fimbrial protein, A chain | Escherichia coli HVH 173 (3-9175482) | 323.1 | 2.5E-93 |  |
| 1014 | IAM_13814 | EGV47111 | major type 1 subunit fimbrin (pilin) | Escherichia coli XH001 | 323.1 | 2.5E-93 |  |
| 1015 | WCA_00519 | ELC04740 | type-1 fimbrial protein, A chain | Escherichia coli KTE2 | 323.1 | 2.5E-93 |  |
| 1016 | AF55_03220 | EZQ66574 | type-1 fimbrial protein, A chain | Escherichia coli BIDMC 82 | 323.1 | 2.5E-93 |  |
| 1017 | G781_04794 | EQR15620 | type-1 fimbrial protein, A chain | Escherichia coli HVH 119 (4-6879578) | 323.1 | 2.5E-93 |  |
| 1018 | G434_03625 | EOW71231 | fimbrial protein FimA | Escherichia sp. KTE172 | 323.1 | 2.5E-93 |  |
| 1019 | b4314 | AAC77270 | major type 1 subunit fimbrin (pilin) | Escherichia coli str. K-12 substr. MG1655 (GCA_000801205) | 323.1 | 2.5E-93 | Yes |
| 1020 | G867_04846 | EQU82036 | type-1 fimbrial protein, A chain | Escherichia coli HVH 215 (4-3008371) | 323.1 | 2.5E-93 |  |
| 1021 | HMPREF1589_00471 | ESA76443 | type-1 fimbrial protein, A chain | Escherichia coli 113290 | 323.1 | 2.5E-93 |  |
| 1022 | WAS_00523 | EOX00053 | fimbrial protein FimA | Escherichia coli KTE1 | 323.1 | 2.5E-93 |  |
| 1023 | A1YI_00407 | EOW53765 | fimbrial protein FimA | Escherichia coli KTE132 | 323.1 | 2.5E-93 |  |
| 1024 | G803_04917 | EQS19590 | type-1 fimbrial protein, A chain | Escherichia coli HVH 145 (4-5672112) | 323.1 | 2.5E-93 |  |
| 1025 | G771_04838 | EQQ61861 | type-1 fimbrial protein, A chain | Escherichia coli HVH 110 (4-6978754) | 323.1 | 2.5E-93 |  |
| 1026 | G767_04789 | EQQ54008 | type-1 fimbrial protein, A chain | Escherichia coli HVH 106 (4-6881831) | 323.1 | 2.5E-93 |  |
| 1027 | ECB_04183 | ACT41809 | major type 1 subunit fimbrin (pilin) | Escherichia coli B str. REL606 | 323.1 | 2.5E-93 |  |
| 1028 | AC72_4741 | KDY08109 | type-1 fimbrial protein, A chain | Escherichia coli 2-316-03_S4_C1 | 323.1 | 2.5E-93 |  |
| 1029 | N840_4397 | AGX36208 | major type 1 subunit fimbrin (pilin) | synthetic Escherichia coli C321.deltaA | 323.1 | 2.5E-93 |  |
| 1030 | BW25113_4314 | AIN34588 | major type 1 subunit fimbrin (pilin) | Escherichia coli BW25113 | 323.1 | 2.5E-93 |  |
| 1031 | G747_04448 | EQP87837 | type-1 fimbrial protein, A chain | Escherichia coli HVH 85 (4-0792144) | 323.1 | 2.5E-93 |  |
| 1032 | G823_04742 | EQS83503 | type-1 fimbrial protein, A chain | Escherichia coli HVH 167 (4-6073565) | 323.1 | 2.5E-93 |  |
| 1033 | G789_04710 | EQR56312 | type-1 fimbrial protein, A chain | Escherichia coli HVH 130 (4-7036876) | 323.1 | 2.5E-93 |  |
| 1034 | L454_04734 | ERO99454 | type-1 fimbrial protein, A chain | Escherichia coli BIDMC 19C | 323.1 | 2.5E-93 |  |
| 1035 | A31O_00462 | EOW72644 | fimbrial protein FimA | Escherichia coli KTE170 | 323.1 | 2.5E-93 |  |
| 1036 | BN896_4012 | CDJ74531 | major type 1 subunit fimbrin (pilin) | Escherichia coli str. K-12 substr. MC4100 | 323.1 | 2.5E-93 |  |
| 1037 | ECVR50_4792 | AKA93484 | type-1 fimbrial protein, A chain | Escherichia coli VR50 | 323.1 | 2.5E-93 |  |
| 1038 | G791_04732 | EQR69081 | type-1 fimbrial protein, A chain | Escherichia coli HVH 133 (4-4466519) | 323.1 | 2.5E-93 |  |
| 1039 | A31C_00495 | ELH03244 | fimbrial protein FimA | Escherichia coli KTE158 | 323.1 | 2.5E-93 |  |
| 1040 | ECJG_03528 | EGI23291 | type-1 fimbrial protein, A chain (Type-1A pilin) | Escherichia coli M718 | 323.1 | 2.5E-93 |  |
| 1041 | AB10_4748 | EYE31736 | type-1 fimbrial protein, A chain | Escherichia coli 1-110-08_S1_C1 | 323.1 | 2.5E-93 |  |
| 1042 | B185_015001 | ELL41149 | type 1 fimbriae major subunit FimA | Escherichia coli J96 | 323.1 | 2.5E-93 |  |
| 1043 | PGA_01106 | CDU33093 | Fimbrial protein | Escherichia coli D6-113.11 | 323.1 | 2.5E-93 |  |
| 1044 | A1Y3_00656 | ELF04238 | type-1 fimbrial protein, A chain | Escherichia coli KTE116 | 323.1 | 2.5E-93 |  |
| 1045 | L453_09021 | ETX96657 | type-1 fimbrial protein, A chain | Escherichia coli BIDMC 19B | 323.1 | 2.5E-93 |  |
| 1046 | L428_08940 | ETY35372 | type-1 fimbrial protein, A chain | Escherichia coli BIDMC 2B | 323.1 | 2.5E-93 |  |
| 1047 | CS35_4369 | AKF74576 | major type 1 subunit fimbrin (pilin) | Escherichia coli O17:K52:H18 (strain UMN026 / ExPEC) | 323.1 | 2.5E-93 |  |
| 1048 | OQE_35070 | EIE35467 | fimbrial protein | Escherichia coli J53 | 323.1 | 2.5E-93 |  |
| 1049 | G792_04759 | EQR70501 | type-1 fimbrial protein, A chain | Escherichia coli HVH 134 (4-6073441) | 323.1 | 2.5E-93 |  |
| 1050 | G939_03667 | EQX93236 | type-1 fimbrial protein, A chain | Escherichia coli UMEA 3201-1 | 323.1 | 2.5E-93 |  |
| 1051 | ECP02999171_0044 | ENA10861 | type-1 fimbrial protein, A chain | Escherichia coli P0299917.1 | 323.1 | 2.5E-93 |  |
| 1052 | G874_04773 | EQV16304 | type-1 fimbrial protein, A chain | Escherichia coli HVH 223 (4-2976528) | 323.1 | 2.5E-93 |  |
| 1053 | G929_04685 | EQX46313 | type-1 fimbrial protein, A chain | Escherichia coli UMEA 3174-1 | 323.1 | 2.5E-93 |  |
| 1054 | AF44_03470 | KDG67733 | type-1 fimbrial protein, A chain | Escherichia coli MGH 58 | 323.1 | 2.5E-93 |  |
| 1055 | ESSG_00230 | EIG50740 | type-1 fimbrial protein, A chain | Escherichia coli H730 | 323.1 | 2.5E-93 |  |
| 1056 | L432_08810 | ETY61290 | type-1 fimbrial protein, A chain | Escherichia coli BIDMC 6 | 323.1 | 2.5E-93 |  |
| 1057 | G798_04753 | EQR98235 | type-1 fimbrial protein, A chain | Escherichia coli HVH 140 (4-5894387) | 323.1 | 2.5E-93 |  |
| 1058 | HMPREF1612_01606 | ESD92067 | type-1 fimbrial protein, A chain | Escherichia coli 908585 | 323.1 | 2.5E-93 |  |
| 1059 | G808_04463 | EQS44523 | type-1 fimbrial protein, A chain | Escherichia coli HVH 150 (4-3258106) | 323.1 | 2.5E-93 |  |
| 1060 | ECUMN_4921 | CAR16032 | major type 1 subunit fimbrin (pilin) | Escherichia coli UMN026 | 323.1 | 2.5E-93 |  |
| 1061 | G809_04590 | EQS43758 | type-1 fimbrial protein, A chain | Escherichia coli HVH 151 (4-5755573) | 323.1 | 2.5E-93 |  |
| 1062 | G978_04648 | EQZ28302 | type-1 fimbrial protein, A chain | Escherichia coli UMEA 3592-1 | 323.1 | 2.5E-93 |  |
| 1063 | HMPREF9552_00201 | EFJ76117 | type-1 fimbrial protein, A chain | Escherichia coli MS 198-1 | 323.1 | 2.5E-93 |  |
| 1064 | A1YK_00102 | EOW60896 | fimbrial protein FimA | Escherichia coli KTE134 | 323.1 | 2.5E-93 |  |
| 1065 | ECD_04183 | ACT45964 | major type 1 subunit fimbrin (pilin) | Escherichia coli BL21(DE3) | 323.1 | 2.5E-93 |  |
| 1066 | P804_03282 | ETX76946 | type-1 fimbrial protein, A chain | Escherichia coli BIDMC 43b | 323.1 | 2.5E-93 |  |
| 1067 | A195_04376 | ELD74179 | type-1 fimbrial protein, A chain | Escherichia coli KTE235 | 323.1 | 2.5E-93 |  |
| 1068 | G705_04736 | EQO01882 | type-1 fimbrial protein, A chain | Escherichia coli HVH 29 (4-3418073) | 323.1 | 2.5E-93 |  |
| 1069 | A15Q_04922 | ELD16697 | type-1 fimbrial protein, A chain | Escherichia coli KTE208 | 323.1 | 2.5E-93 |  |
| 1070 | G732_04721 | EQP20024 | type-1 fimbrial protein, A chain | Escherichia coli HVH 63 (4-2542528) | 323.1 | 2.5E-93 |  |
| 1071 | HMPREF1600_00675 | ESD30374 | type-1 fimbrial protein, A chain | Escherichia coli 907715 | 323.1 | 2.5E-93 |  |
| 1072 | L670_08809 | KGL70389 | type 1 fimbriae major subunit FimA | Escherichia coli NCTC 50110 | 323.1 | 2.5E-93 |  |
| 1073 | A1W1_04837 | ELE76382 | type-1 fimbrial protein, A chain | Escherichia coli KTE83 | 323.1 | 2.5E-93 |  |
| 1074 | ECP029970676_5106 | ENC26695 | type-1 fimbrial protein, A chain | Escherichia coli P02997067.6 | 323.1 | 2.5E-93 | Yes |
| 1075 | WIK_04758 | ELI36502 | fimbrial protein FimA | Escherichia coli KTE122 | 323.1 | 2.5E-93 |  |
| 1076 | L446_09022 | ETY13459 | type-1 fimbrial protein, A chain | Escherichia coli BIDMC 17A | 323.1 | 2.5E-93 |  |
| 1077 | WIQ_04629 | ELI49492 | fimbrial protein FimA | Escherichia coli KTE128 | 323.1 | 2.5E-93 |  |
| 1078 | L452_05246 | ETY08712 | type-1 fimbrial protein, A chain | Escherichia coli BIDMC 19A | 323.1 | 2.5E-93 |  |
| 1079 | G860_04853 | EQU56241 | type-1 fimbrial protein, A chain | Escherichia coli HVH 208 (4-3112292) | 323.1 | 2.5E-93 |  |
| 1080 | A17I_01810 | EOV39912 | fimbrial protein FimA | Escherichia coli KTE222 | 323.1 | 2.5E-93 |  |
| 1081 | A1UW_04759 | ELE66179 | type-1 fimbrial protein, A chain | Escherichia coli KTE80 | 323.1 | 2.5E-93 |  |
| 1082 | C201_20559 | EMD02923 | type 1 fimbriae major subunit FimA | Escherichia coli S17 | 323.1 | 2.5E-93 |  |
| 1083 | ECFG_03456 | EFI22334 | type-1 fimbrial protein | Escherichia coli FVEC1302 | 323.1 | 2.5E-93 |  |
| 1084 | A1YE_00634 | EOW43062 | fimbrial protein FimA | Escherichia coli KTE127 | 323.1 | 2.5E-93 |  |
| 1085 | G972_04694 | EQZ08502 | type-1 fimbrial protein, A chain | Escherichia coli UMEA 3355-1 | 323.1 | 2.5E-93 |  |
| 1086 | P803_04621 | ETX82096 | type-1 fimbrial protein, A chain | Escherichia coli BIDMC 43a | 323.1 | 2.5E-93 |  |
| 1087 | G744_00122 | EQP94288 | type-1 fimbrial protein, A chain | Escherichia coli HVH 82 (4-2209276) | 323.1 | 2.5E-93 |  |
| 1088 | WEK_00141 | ELC51172 | type-1 fimbrial protein, A chain | Escherichia coli KTE26 | 323.1 | 2.5E-93 |  |
| 1089 | HMPREF9551_00617 | EFI90323 | type-1 fimbrial protein, A chain | Escherichia coli MS 196-1 | 323.1 | 2.5E-93 |  |
| 1090 | A1YG_00369 | EOW53818 | fimbrial protein FimA | Escherichia coli KTE130 | 323.1 | 2.5E-93 |  |
| 1091 | G813_04738 | ERA63171 | type-1 fimbrial protein, A chain | Escherichia coli HVH 155 (4-4509048) | 323.1 | 2.5E-93 |  |
| 1092 | AB88_5622 | KEN94133 | type-1 fimbrial protein, A chain | Escherichia coli 2-222-05_S3_C1 | 323.1 | 2.5E-93 |  |
| 1093 | G698_04633 | EQN74776 | type-1 fimbrial protein, A chain | Escherichia coli HVH 22 (4-2258986) | 323.1 | 2.5E-93 |  |
| 1094 | EC3006_4954 | EKI32767 | type-1 fimbrial protein, A chain | Escherichia coli 3006 | 323.1 | 2.5E-93 |  |
| 1095 | L436_08815 | ETY23985 | type-1 fimbrial protein, A chain | Escherichia coli BIDMC 9 | 323.1 | 2.5E-93 |  |
| 1096 | WGM_04853 | ELJ61285 | fimbrial protein FimA | Escherichia coli KTE82 | 323.1 | 2.5E-93 |  |
| 1097 | G835_04891 | EQT33061 | type-1 fimbrial protein, A chain | Escherichia coli HVH 183 (4-3205932) | 323.1 | 2.5E-93 |  |
| 1098 | BWG_4012 | ACR63705 | major type 1 subunit fimbrin (pilin) | Escherichia coli BW2952 | 323.1 | 2.5E-93 |  |
| 1099 | G784_04612 | EQR29804 | type-1 fimbrial protein, A chain | Escherichia coli HVH 122 (4-6851606) | 323.1 | 2.5E-93 |  |
| 1100 | AC46_3698 | KDX84859 | type-1 fimbrial protein, A chain | Escherichia coli 2-222-05_S3_C3 | 323.1 | 2.5E-93 |  |
| 1101 | G720_04958 | EQO74520 | type-1 fimbrial protein, A chain | Escherichia coli HVH 45 (4-3129918) | 323.1 | 2.5E-93 |  |
| 1102 | G979_04818 | EQZ33239 | type-1 fimbrial protein, A chain | Escherichia coli UMEA 3609-1 | 323.1 | 2.5E-93 |  |
| 1103 | EC5761_17139 | EIL65026 | major type 1 subunit fimbrin (pilin) | Escherichia coli 576-1 | 323.1 | 2.5E-93 |  |
| 1104 | A17O_01128 | EOX13529 | fimbrial protein FimA | Escherichia coli KTE225 | 323.1 | 2.5E-93 |  |
| 1105 | HMPREF1606_00952 | ESD61047 | type-1 fimbrial protein, A chain | Escherichia coli 908522 | 323.1 | 2.5E-93 |  |
| 1106 | HMPREF9543_03375 | EFK89795 | type-1 fimbrial protein, A chain | Escherichia coli MS 146-1 | 323.1 | 2.5E-93 |  |
| 1107 | A1U9_04988 | EOV54780 | fimbrial protein FimA | Escherichia coli KTE68 | 323.1 | 2.5E-93 |  |
| 1108 | HMPREF1605_05328 | ESD45676 | type-1 fimbrial protein, A chain | Escherichia coli 908521 | 323.1 | 2.5E-93 |  |
| 1109 | G774_04771 | EQQ82415 | type-1 fimbrial protein, A chain | Escherichia coli HVH 113 (4-7535473) | 323.1 | 2.5E-93 |  |
| 1110 | G793_04685 | EQR73358 | type-1 fimbrial protein, A chain | Escherichia coli HVH 135 (4-4449320) | 323.1 | 2.5E-93 |  |
| 1111 | ECDH1ME8569_4172 | BAJ46028 | type 1 fimbriae major subunit FimA | Escherichia coli DH1 | 323.1 | 2.5E-93 |  |
| 1112 | A15A_00189 | EOV27213 | fimbrial protein FimA | Escherichia coli KTE200 | 323.1 | 2.5E-93 |  |
| 1113 | AC18_4880 | KEN93665 | type-1 fimbrial protein, A chain | Escherichia coli 2-222-05_S3_C2 | 323.1 | 2.5E-93 |  |
| 1114 | ECBD_3721 | ACT30714 | Fimbrial protein | Escherichia coli 'BL21-Gold(DE3)pLysS AG' | 323.1 | 2.5E-93 |  |
| 1115 | AD06_4796 | KEN17297 | type-1 fimbrial protein, A chain | Escherichia coli 7-233-03_S4_C2 | 323.1 | 2.5E-93 |  |
| 1116 | A317_02443 | ELG97929 | fimbrial protein FimA | Escherichia coli KTE154 | 323.1 | 2.5E-93 |  |
| 1117 | A15I_04648 | ELD00672 | type-1 fimbrial protein, A chain | Escherichia coli KTE204 | 323.1 | 2.5E-93 |  |
| 1118 | L447_04580 | ETY13678 | type-1 fimbrial protein, A chain | Escherichia coli BIDMC 17B | 323.1 | 2.5E-93 |  |
| 1119 | A157_00357 | EOV31520 | fimbrial protein FimA | Escherichia coli KTE198 | 323.1 | 2.5E-93 |  |
| 1120 | SH05_03230 | AKD59511 | type-1 fimbrial protein subunit A | Escherichia coli (strain K12) | 323.1 | 2.5E-93 |  |
| 1121 | BAE78307 | BAE78307 | major type 1 subunit fimbrin | Escherichia coli str. K-12 substr. W3110 | 323.1 | 2.5E-93 |  |
| 1122 | G841_04594 | EQT69915 | type-1 fimbrial protein, A chain | Escherichia coli HVH 189 (4-3220125) | 323.1 | 2.5E-93 |  |
| 1123 | G890_04969 | EQV77924 | type-1 fimbrial protein, A chain | Escherichia coli KOEGE 62 (175a) | 323.1 | 2.5E-93 |  |
| 1124 | HMPREF1613_01499 | ESD92718 | type-1 fimbrial protein, A chain | Escherichia coli 908616 | 323.1 | 2.5E-93 |  |
| 1125 | G735_04622 | EQP30896 | type-1 fimbrial protein, A chain | Escherichia coli HVH 69 (4-2837072) | 323.1 | 2.5E-93 |  |
| 1126 | G883_04504 | EQV39954 | type-1 fimbrial protein, A chain | Escherichia coli KOEGE 33 (68a) | 323.1 | 2.5E-93 |  |
| 1127 | L429_09125 | ETY29996 | type-1 fimbrial protein, A chain | Escherichia coli BIDMC 3 | 323.1 | 2.5E-93 |  |
| 1128 | WKI_00026 | ELJ30937 | fimbrial protein FimA | Escherichia coli KTE166 | 323.1 | 2.5E-93 |  |
| 1129 | G821_04860 | EQS73504 | type-1 fimbrial protein, A chain | Escherichia coli HVH 163 (4-4697553) | 323.1 | 2.5E-93 |  |
| 1130 | ECK5_20300 | CCP96082 | type 1 fimbriae major subunit FimA | Escherichia coli O10:K5(L):H4 str. ATCC 23506 | 323.1 | 2.5E-93 |  |
| 1131 | G891_04590 | EQV75288 | type-1 fimbrial protein, A chain | Escherichia coli KOEGE 68 (182a) | 323.1 | 2.5E-93 |  |
| 1132 | PU51_03960 | KHH84709 | type-1 fimbrial protein subunit A | Escherichia coli (GCA_000797655) | 322.8 | 3.1E-93 | Yes |
| 1133 | A151_00051 | EOV12093 | fimbrial protein FimA | Escherichia coli KTE195 | 322.8 | 3.2E-93 | Yes |
| 1134 | AD15_5362 | KDT40716 | type-1 fimbrial protein, A chain | Escherichia coli 3-105-05_S4_C2 | 322.8 | 3.2E-93 |  |
| 1135 | APT93_26500 | KSX88201 | type-1 fimbrial protein subunit A | Escherichia coli (GCA_001463205) | 322.8 | 3.2E-93 |  |
| 1136 | G741_04599 | EQP63487 | type-1 fimbrial protein, A chain | Escherichia coli HVH 78 (4-2735946) | 322.8 | 3.2E-93 |  |
| 1137 | G801_04495 | EQS09434 | type-1 fimbrial protein, A chain | Escherichia coli HVH 143 (4-5674999) | 322.8 | 3.2E-93 |  |
| 1138 | G684_04790 | EQN13461 | type-1 fimbrial protein, A chain | Escherichia coli HVH 4 (4-7276109) | 322.8 | 3.2E-93 |  |
| 1139 | ECKD1_07364 | EIL52300 | Major type 1 subunit fimbrin | Escherichia coli KD1 | 322.3 | 4.6E-93 | Yes |
| 1140 | AM265_19265 | KQJ05320 | type-1 fimbrial protein subunit A | Escherichia coli (GCA_001419945) | 322.3 | 4.8E-93 |  |
| 1141 | AB46_4339 | KDU00178 | type-1 fimbrial protein, A chain | Escherichia coli 3-267-03_S1_C2 | 322 | 5.6E-93 | Yes |
| 1142 | G934_04677 | EQX61864 | type-1 fimbrial protein, A chain | Escherichia coli UMEA 3185-1 | 321.7 | 6.9E-93 |  |
| 1143 | HMPREF0986_00669 | EHP67405 | type-1 fimbrial protein, A chain | Escherichia coli 4_1_47FAA | 321.7 | 7.3E-93 | Yes |
| 1144 | G994_04612 | EQZ94021 | type-1 fimbrial protein, A chain | Escherichia coli UMEA 3718-1 | 321.7 | 7.3E-93 | Yes |
| 1145 | GT42_21965 | KIE73415 | type-1 fimbrial protein subunit A | Escherichia coli | 321.7 | 7.3E-93 |  |
| 1146 | G914_04742 | EQW85484 | type-1 fimbrial protein, A chain | Escherichia coli UMEA 3139-1 | 321.7 | 7.3E-93 | Yes |
| 1147 | HMPREF1617_00762 | ESE21358 | type-1 fimbrial protein, A chain | Escherichia coli 908675 | 321.6 | 7.6E-93 |  |
| 1148 | ERKG_02924 | EGB46474 | fimbrial protein | Escherichia coli H252 | 321.6 | 7.6E-93 |  |
| 1149 | G875_04709 | EQV20524 | type-1 fimbrial protein, A chain | Escherichia coli HVH 225 (4-1273116) | 321.6 | 7.6E-93 |  |
| 1150 | A13A_04990 | EOW90156 | fimbrial protein FimA | Escherichia coli KTE182 | 321.6 | 7.6E-93 |  |
| 1151 | G765_05042 | EQQ45648 | type-1 fimbrial protein, A chain | Escherichia coli HVH 104 (4-6977960) | 321.6 | 7.6E-93 |  |
| 1152 | A15K_04819 | ELD04931 | type-1 fimbrial protein, A chain | Escherichia coli KTE205 | 321.6 | 7.6E-93 |  |
| 1153 | G788_04807 | EQR53843 | type-1 fimbrial protein, A chain | Escherichia coli HVH 128 (4-7030436) | 321.6 | 7.6E-93 |  |
| 1154 | G695_04682 | EQN65035 | type-1 fimbrial protein, A chain | Escherichia coli HVH 19 (4-7154984) | 321.6 | 7.6E-93 |  |
| 1155 | G737_04814 | EQP45587 | type-1 fimbrial protein, A chain | Escherichia coli HVH 73 (4-2393174) | 321.6 | 7.6E-93 |  |
| 1156 | G730_04640 | EQP09548 | type-1 fimbrial protein, A chain | Escherichia coli HVH 59 (4-1119338) | 321.6 | 7.6E-93 |  |
| 1157 | G690_04425 | ESP12976 | type-1 fimbrial protein, A chain | Escherichia coli HVH 12 (4-7653042) | 321.6 | 7.6E-93 |  |
| 1158 | G882_04584 | EQV36552 | type-1 fimbrial protein, A chain | Escherichia coli KOEGE 32 (66a) | 321.6 | 7.6E-93 |  |
| 1159 | A19A_00280 | EOX16867 | fimbrial protein FimA | Escherichia coli KTE240 | 321.6 | 7.6E-93 |  |
| 1160 | WIS_04718 | ELI51548 | fimbrial protein FimA | Escherichia coli KTE129 | 321.6 | 7.6E-93 |  |
| 1161 | G736_04969 | EQP42913 | type-1 fimbrial protein, A chain | Escherichia coli HVH 70 (4-2963531) | 321.6 | 7.6E-93 |  |
| 1162 | AC85_5369 | KEJ53303 | type-1 fimbrial protein, A chain | Escherichia coli 3-020-07_S4_C1 | 321.6 | 7.6E-93 |  |
| 1163 | WCC_00155 | ELC03551 | type-1 fimbrial protein, A chain | Escherichia coli KTE4 | 321.6 | 7.6E-93 |  |
| 1164 | AD30_5214 | KDY13735 | type-1 fimbrial protein, A chain | Escherichia coli 2-316-03_S4_C3 | 321.6 | 7.6E-93 |  |
| 1165 | G865_04973 | EQU75555 | type-1 fimbrial protein, A chain | Escherichia coli HVH 213 (4-3042928) | 321.6 | 7.6E-93 |  |
| 1166 | WAU_00553 | EOU41305 | fimbrial protein FimA | Escherichia coli KTE3 | 321.6 | 7.6E-93 |  |
| 1167 | G787_04666 | EQR49486 | type-1 fimbrial protein, A chain | Escherichia coli HVH 127 (4-7303629) | 321.6 | 7.6E-93 |  |
| 1168 | G873_04538 | EQV07674 | type-1 fimbrial protein, A chain | Escherichia coli HVH 222 (4-2977443) | 321.6 | 7.6E-93 |  |
| 1169 | P810_03280 | ETY55486 | type-1 fimbrial protein, A chain | Escherichia coli BIDMC 49a | 321.6 | 7.6E-93 |  |
| 1170 | G806_01559 | ESP34412 | type-1 fimbrial protein, A chain | Escherichia coli HVH 148 (4-3192490) | 321.6 | 7.6E-93 |  |
| 1171 | G833_04628 | EQT22132 | type-1 fimbrial protein, A chain | Escherichia coli HVH 180 (4-3051617) | 321.6 | 7.6E-93 |  |
| 1172 | WGO_04699 | ELJ63705 | fimbrial protein FimA | Escherichia coli KTE85 | 321.6 | 7.6E-93 |  |
| 1173 | APU16_13290 | KSZ09636 | type-1 fimbrial protein subunit A | Escherichia coli HVH 70 (4-2963531) | 321.6 | 7.6E-93 |  |
| 1174 | G901_04564 | EQW26670 | type-1 fimbrial protein, A chain | Escherichia coli UMEA 3041-1 | 321.6 | 7.6E-93 |  |
| 1175 | G915_04338 | EQW93087 | type-1 fimbrial protein, A chain | Escherichia coli UMEA 3140-1 | 321.6 | 7.6E-93 |  |
| 1176 | G768_04914 | EQQ62541 | type-1 fimbrial protein, A chain | Escherichia coli HVH 107 (4-5860571) | 321.6 | 7.6E-93 |  |
| 1177 | G906_05082 | EQW54025 | type-1 fimbrial protein, A chain | Escherichia coli UMEA 3088-1 | 321.6 | 7.6E-93 |  |
| 1178 | G832_04603 | ESP27348 | type-1 fimbrial protein, A chain | Escherichia coli HVH 178 (4-3189163) | 321.6 | 7.6E-93 |  |
| 1179 | G944_04803 | EQY08473 | type-1 fimbrial protein, A chain | Escherichia coli UMEA 3215-1 | 321.6 | 7.6E-93 |  |
| 1180 | P811_03271 | ETY52637 | type-1 fimbrial protein, A chain | Escherichia coli BIDMC 49b | 321.6 | 7.6E-93 |  |
| 1181 | WEE_00334 | ELF77866 | fimbrial protein FimA | Escherichia coli KTE23 | 321.2 | 1E-92 |  |
| 1182 | WEI_00582 | ELC40124 | type-1 fimbrial protein, A chain | Escherichia coli KTE25 | 321.2 | 1E-92 | Yes |
| 1183 | WK9_04505 | ELI91230 | fimbrial protein FimA | Escherichia coli KTE150 | 321.2 | 1E-92 |  |
| 1184 | WE1_00511 | ELF60572 | fimbrial protein FimA | Escherichia coli KTE17 | 321.2 | 1E-92 |  |
| 1185 | UC40_06790 | KJG98284 | type-1 fimbrial protein subunit A | Escherichia coli | 321.2 | 1E-92 | Yes |
| 1186 | WE3_00332 | ELF68609 | fimbrial protein FimA | Escherichia coli KTE18 | 321.2 | 1E-92 |  |
| 1187 | A179_00604 | ELH78759 | fimbrial protein FimA | Escherichia coli KTE217 | 321.2 | 1E-92 |  |
| 1188 | AC59_4540 | KDT71121 | type-1 fimbrial protein, A chain | Escherichia coli 3-373-03_S3_C3 | 321.1 | 1.1E-92 |  |
| 1189 | A319_00328 | EOW64287 | fimbrial protein FimA | Escherichia coli KTE155 | 321.1 | 1.1E-92 |  |
| 1190 | A31A_00658 | ELF23249 | type-1 fimbrial protein, A chain | Escherichia coli KTE156 | 321.1 | 1.1E-92 | Yes |
| 1191 | AC34_4560 | KDU07641 | type-1 fimbrial protein, A chain | Escherichia coli 3-373-03_S3_C2 | 321.1 | 1.1E-92 | Yes |
| 1192 | A155_00583 | ELH54107 | fimbrial protein FimA | Escherichia coli KTE197 | 321.1 | 1.1E-92 |  |
| 1193 | A1YM_01689 | ELG65693 | fimbrial protein FimA | Escherichia coli KTE135 | 320.9 | 1.2E-92 |  |
| 1194 | HMPREF9346_03624 | EFK44737 | type-1 fimbrial protein, A chain | Escherichia coli MS 119-7 | 320.9 | 1.2E-92 | Yes |
| 1195 | ECPG_01543 | EGI43216 | type-1 fimbrial protein, A chain (Type-1A pilin) | Escherichia coli H591 | 320.9 | 1.2E-92 |  |
| 1196 | ECDEC15E_0002 | EHY21320 | fimbrial subunit type 1 | Escherichia coli DEC15E | 320.9 | 1.2E-92 |  |
| 1197 | AB71_0113 | EZK00367 | type-1 fimbrial protein, A chain | Escherichia coli 1-182-04_S1_C3 | 320.9 | 1.2E-92 |  |
| 1198 | EC2788150_4859 | EMW37565 | type-1 fimbrial protein, A chain | Escherichia coli 2788150 | 320.9 | 1.2E-92 |  |
| 1199 | AC64_4800 | KEM79681 | type-1 fimbrial protein, A chain | Escherichia coli 6-537-08_S3_C3 | 320.9 | 1.2E-92 |  |
| 1200 | ECDEC15B_5181 | EHX98948 | fimbrial subunit type 1 | Escherichia coli DEC15B | 320.9 | 1.2E-92 |  |
| 1201 | EC2865200_4920 | EMV84891 | type-1 fimbrial protein, A chain | Escherichia coli 2865200 | 320.9 | 1.2E-92 |  |
| 1202 | HMPREF9348_02794 | EFO57967 | type-1 fimbrial protein, A chain | Escherichia coli MS 145-7 | 320.9 | 1.2E-92 |  |
| 1203 | ECMP0209801_0001 | END56532 | type-1 fimbrial protein, A chain | Escherichia coli MP020980.1 | 320.9 | 1.2E-92 |  |
| 1204 | EC2780750_4865 | EMW44903 | type-1 fimbrial protein, A chain | Escherichia coli 2780750 | 320.9 | 1.2E-92 |  |
| 1205 | VP69_08590 | KKB18851 | type-1 fimbrial protein subunit A | Escherichia coli | 320.9 | 1.2E-92 |  |
| 1206 | AB64_2973 | KDY91232 | type-1 fimbrial protein, A chain | Escherichia coli 2-427-07_S1_C3 | 320.9 | 1.2E-92 |  |
| 1207 | HMPREF1591_03540 | ESA62900 | type-1 fimbrial protein, A chain | Escherichia coli 113303 | 320.9 | 1.2E-92 |  |
| 1208 | AC39_4987 | KEN12677 | type-1 fimbrial protein, A chain | Escherichia coli 6-537-08_S3_C2 | 320.9 | 1.2E-92 |  |
| 1209 | AC48_3478 | KDY30468 | type-1 fimbrial protein, A chain | Escherichia coli 2-427-07_S3_C3 | 320.9 | 1.2E-92 |  |
| 1210 | ECDEC15A_5300 | EHX92016 | fimbrial subunit type 1 | Escherichia coli DEC15A | 320.9 | 1.2E-92 |  |
| 1211 | ECOT7509_4664 | ERC51128 | type-1 fimbrial protein, A chain | Escherichia coli TW07509 | 320.9 | 1.2E-92 |  |
| 1212 | ECO9340_01865 | EIL09200 | major type 1 subunit fimbrin (pilin) | Escherichia coli O103:H25 str. CVM9340 | 320.9 | 1.2E-92 |  |
| 1213 | AB38_4978 | EYE31194 | type-1 fimbrial protein, A chain | Escherichia coli 1-110-08_S1_C2 | 320.9 | 1.2E-92 |  |
| 1214 | ECDEC15D_4998 | EHY09583 | fimbrial subunit type 1 | Escherichia coli DEC15D | 320.9 | 1.2E-92 |  |
| 1215 | ECP03047993_4945 | END98332 | type-1 fimbrial protein, A chain | Escherichia coli P0304799.3 | 320.9 | 1.2E-92 |  |
| 1216 | G797_04416 | EQR89488 | type-1 fimbrial protein, A chain | Escherichia coli HVH 139 (4-3192644) | 320.9 | 1.2E-92 |  |
| 1217 | SU67_18565 | KIO39377 | type-1 fimbrial protein subunit A | Escherichia coli O139:H28 str. E24377A (GCA_000832005) | 320.9 | 1.2E-92 |  |
| 1218 | AB40_4846 | KDA65775 | type-1 fimbrial protein, A chain | Escherichia coli 1-182-04_S1_C2 | 320.9 | 1.2E-92 |  |
| 1219 | T22_006302 | ENO10389 | type 1 fimbriae major subunit FimA | Escherichia coli O157:H43 str. T22 | 320.9 | 1.2E-92 |  |
| 1220 | BY04_19115 | EYX86421 | type-1 fimbrial protein subunit A | Escherichia coli O156:H25 str. 2011C-3602 | 320.9 | 1.2E-92 |  |
| 1221 | AB04_4528 | KDZ90580 | type-1 fimbrial protein, A chain | Escherichia coli 2-427-07_S1_C1 | 320.9 | 1.2E-92 |  |
| 1222 | BX66_13200 | EYZ24230 | type-1 fimbrial protein subunit A | Escherichia coli O103:H25 str. 2010C-4529 | 320.9 | 1.2E-92 |  |
| 1223 | AB33_4628 | KDY17857 | type-1 fimbrial protein, A chain | Escherichia coli 2-427-07_S1_C2 | 320.9 | 1.2E-92 |  |
| 1224 | ECOK1357_4709 | EFZ67440 | type-1 fimbrial protein, A chain | Escherichia coli OK1357 | 320.9 | 1.2E-92 |  |
| 1225 | ECW26_02960 | EID69038 | fimbrial protein | Escherichia coli W26 | 320.9 | 1.2E-92 |  |
| 1226 | EC2864350_4660 | ENA89895 | type-1 fimbrial protein, A chain | Escherichia coli 2864350 | 320.9 | 1.2E-92 |  |
| 1227 | AE49_03671 | KDG17545 | type-1 fimbrial protein, A chain | Escherichia coli BIDMC 74 | 320.9 | 1.2E-92 |  |
| 1228 | AC17_2107 | KDW99693 | type-1 fimbrial protein, A chain | Escherichia coli 2-210-07_S3_C2 | 320.9 | 1.2E-92 |  |
| 1229 | AB12_5247 | EZK34629 | type-1 fimbrial protein, A chain | Escherichia coli 1-182-04_S1_C1 | 320.9 | 1.2E-92 |  |
| 1230 | AD14_4809 | KDZ71916 | type-1 fimbrial protein, A chain | Escherichia coli 3-073-06_S4_C2 | 320.9 | 1.2E-92 |  |
| 1231 | AC11_4755 | KEM71256 | type-1 fimbrial protein, A chain | Escherichia coli 6-537-08_S3_C1 | 320.9 | 1.2E-92 |  |
| 1232 | ECMP0209802_0275 | EMX57009 | type-1 fimbrial protein, A chain | Escherichia coli MP020980.2 | 320.9 | 1.2E-92 |  |
| 1233 | AB90_5128 | KDY26605 | type-1 fimbrial protein, A chain | Escherichia coli 2-427-07_S3_C1 | 320.9 | 1.2E-92 |  |
| 1234 | AC26_3404 | EYD82615 | type-1 fimbrial protein, A chain | Escherichia coli 1-176-05_S3_C2 | 320.9 | 1.2E-92 | Yes |
| 1235 | G887_04639 | EQV61276 | type-1 fimbrial protein, A chain | Escherichia coli KOEGE 56 (169a) | 320.6 | 1.5E-92 |  |
| 1236 | G700_04354 | EQN79976 | type-1 fimbrial protein, A chain | Escherichia coli HVH 24 (4-5985145) | 320.6 | 1.5E-92 |  |
| 1237 | WE9_00119 | ELC44840 | type-1 fimbrial protein, A chain | Escherichia coli KTE21 | 320.6 | 1.5E-92 | Yes |
| 1238 | G950_04655 | EQY37713 | type-1 fimbrial protein, A chain | Escherichia coli UMEA 3230-1 | 320.6 | 1.5E-92 |  |
| 1239 | G822_04297 | EQS77296 | type-1 fimbrial protein, A chain | Escherichia coli HVH 164 (4-5953081) | 320.6 | 1.5E-92 |  |
| 1240 | ERS139229_02179 | CTW91689 | fimbrial protein | Escherichia coli HVH 25 (4-5851939) | 320.6 | 1.5E-92 | Yes |
| 1241 | G966_05068 | ESK25391 | type-1 fimbrial protein, A chain | Escherichia coli UMEA 3323-1 | 320.6 | 1.5E-92 |  |
| 1242 | WE7_00089 | EOU74393 | fimbrial protein FimA | Escherichia coli KTE20 | 320.6 | 1.5E-92 |  |
| 1243 | G948_04667 | EQY34874 | type-1 fimbrial protein, A chain | Escherichia coli UMEA 3221-1 | 320.6 | 1.5E-92 |  |
| 1244 | HMPREF1602_05060 | ESD31663 | type-1 fimbrial protein, A chain | Escherichia coli 907889 | 320.6 | 1.5E-92 |  |
| 1245 | ECSTECMHI813_4800 | EGW99974 | type-1 fimbrial protein, A chain | Escherichia coli STEC_MHI813 | 320.6 | 1.5E-92 | Yes |
| 1246 | ESNG_01161 | EHO01969 | type-1 fimbrial protein | Escherichia coli B093 | 320.6 | 1.5E-92 | Yes |
| 1247 | G701_04772 | EQN87712 | type-1 fimbrial protein, A chain | Escherichia coli HVH 25 (4-5851939) | 320.6 | 1.5E-92 |  |
| 1248 | G785_04654 | EQR38175 | type-1 fimbrial protein, A chain | Escherichia coli HVH 125 (4-2634716) | 320.6 | 1.5E-92 |  |
| 1249 | EC970264_4949 | KIO83567 | type-1 fimbrial protein, A chain | Escherichia coli 97.0264 | 320.6 | 1.6E-92 |  |
| 1250 | G938_04723 | EQX81938 | type-1 fimbrial protein, A chain | Escherichia coli UMEA 3200-1 | 320.6 | 1.6E-92 |  |
| 1251 | A1SG_01140 | ELG10035 | fimbrial protein FimA | Escherichia coli KTE54 | 320.6 | 1.6E-92 | Yes |
| 1252 | WES_00525 | EOU82573 | fimbrial protein FimA | Escherichia sp. KTE31 | 320.6 | 1.6E-92 |  |
| 1253 | WC5_01838 | EOU45983 | fimbrial protein FimA | Escherichia sp. KTE114 | 320.6 | 1.6E-92 |  |
| 1254 | WEW_02710 | EOQ54956 | fimbrial protein FimA | Escherichia coli KTE33 | 320.6 | 1.6E-92 | Yes |
| 1255 | WEQ_04190 | ELF82189 | fimbrial protein FimA | Escherichia coli KTE29 | 320.4 | 1.7E-92 | Yes |
| 1256 | ECSTECC16502_0298 | EGW75272 | type-1 fimbrial protein, A chain | Escherichia coli STEC_C165-02 | 320.3 | 1.9E-92 | Yes |
| 1257 | AE47_03889 | KDG05526 | type-1 fimbrial protein, A chain | Escherichia coli BIDMC 72 | 320 | 2.3E-92 |  |
| 1258 | A17C_04559 | EOV29740 | fimbrial protein FimA | Escherichia coli KTE219 | 320 | 2.3E-92 |  |
| 1259 | WKC_04505 | ELJ04637 | fimbrial protein FimA | Escherichia coli KTE157 | 320 | 2.3E-92 | Yes |
| 1260 | AE48_04581 | KDG10000 | type-1 fimbrial protein, A chain | Escherichia coli BIDMC 73 | 320 | 2.3E-92 |  |
| 1261 | SK82_03417 | KLX80319 | type-1 fimbrial protein, A chain | Escherichia coli (GCA_001030585) | 320 | 2.3E-92 |  |
| 1262 | A17U_03570 | ELD56439 | type-1 fimbrial protein, A chain | Escherichia coli KTE228 | 319.9 | 2.5E-92 |  |
| 1263 | G750_04674 | EQP98727 | type-1 fimbrial protein, A chain | Escherichia coli HVH 88 (4-5854636) | 319.9 | 2.5E-92 |  |
| 1264 | APT88_04505 | KSX54621 | type-1 fimbrial protein subunit A | Escherichia coli | 319.9 | 2.5E-92 |  |
| 1265 | L408_00427 | ETY51030 | type-1 fimbrial protein, A chain | Escherichia coli BWH 40 | 319.9 | 2.5E-92 |  |
| 1266 | WKU_04618 | ELJ37073 | fimbrial protein FimA | Escherichia coli KTE177 | 319.9 | 2.5E-92 |  |
| 1267 | WI7_04511 | ELI02044 | fimbrial protein FimA | Escherichia coli KTE105 | 319.9 | 2.5E-92 |  |
| 1268 | A13Q_00333 | ELH29750 | fimbrial protein FimA | Escherichia coli KTE190 | 319.9 | 2.5E-92 |  |
| 1269 | HMPREF1588_04974 | ESA64415 | type-1 fimbrial protein, A chain | Escherichia coli 110957 | 319.9 | 2.5E-92 |  |
| 1270 | G733_04579 | EQP27805 | type-1 fimbrial protein, A chain | Escherichia coli HVH 65 (4-2262045) | 319.9 | 2.5E-92 |  |
| 1271 | WC9_04680 | EOU44753 | fimbrial protein FimA | Escherichia coli KTE231 | 319.9 | 2.5E-92 |  |
| 1272 | G895_04774 | EQV95296 | type-1 fimbrial protein, A chain | Escherichia coli KOEGE 77 (202a) | 319.9 | 2.5E-92 |  |
| 1273 | AE52_03240 | KDG33744 | type-1 fimbrial protein, A chain | Escherichia coli BIDMC 77 | 319.9 | 2.5E-92 |  |
| 1274 | EC990741_4987 | EIH11394 | type-1 fimbrial protein, A chain | Escherichia coli 97.0259 | 319.9 | 2.5E-92 | Yes |
| 1275 | ECMG_03841 | EGI29009 | type-1 fimbrial protein, A chain (Type-1A pilin) | Escherichia coli TA143 | 319.4 | 3.5E-92 | Yes |
| 1276 | ERS085411_00308 | CTU56225 | fimbrial protein | Escherichia coli | 319.3 | 3.9E-92 | Yes |
| 1277 | A31M_04706 | ELF33605 | type-1 fimbrial protein, A chain | Escherichia coli KTE169 | 319.2 | 4.1E-92 |  |
| 1278 | G713_04622 | EQO38357 | type-1 fimbrial protein, A chain | Escherichia coli HVH 38 (4-2774682) | 319.2 | 4.1E-92 |  |
| 1279 | WKO_04575 | ELJ21559 | fimbrial protein FimA | Escherichia coli KTE168 | 319.2 | 4.1E-92 |  |
| 1280 | ECNC101_02838 | EFM51619 | major type 1 subunit fimbrin (pilin) FimA | Escherichia coli NC101 | 319.2 | 4.1E-92 | Yes |
| 1281 | WIE_00169 | ELI30707 | fimbrial protein FimA | Escherichia coli KTE113 | 319.2 | 4.1E-92 |  |
| 1282 | WIW_04593 | ELI65829 | fimbrial protein FimA | Escherichia coli KTE133 | 319.2 | 4.1E-92 |  |
| 1283 | A13Y_00290 | ELH19703 | fimbrial protein FimA | Escherichia coli KTE194 | 319.2 | 4.1E-92 |  |
| 1284 | A17M_04695 | ELD47219 | type-1 fimbrial protein, A chain | Escherichia coli KTE224 | 319.2 | 4.1E-92 |  |
| 1285 | A15O_00583 | ELH67498 | fimbrial protein FimA | Escherichia coli KTE207 | 319.2 | 4.1E-92 |  |
| 1286 | EC91649_4795 | KKA62175 | type-1 fimbrial protein, A chain | Escherichia coli 9.1649 | 319.2 | 4.1E-92 |  |
| 1287 | XB01_16960 | KLD45829 | type-1 fimbrial protein subunit A | Escherichia coli | 319.2 | 4.1E-92 |  |
| 1288 | WCY_00459 | ELC32634 | type-1 fimbrial protein, A chain | Escherichia coli KTE16 | 319.2 | 4.1E-92 |  |
| 1289 | AA99_5327 | KDA60607 | type-1 fimbrial protein, A chain | Escherichia coli 2-052-05_S1_C1 | 319.2 | 4.3E-92 |  |
| 1290 | AB25_4924 | EZK26040 | type-1 fimbrial protein, A chain | Escherichia coli 2-005-03_S1_C2 | 319.2 | 4.3E-92 |  |
| 1291 | AB53_4966 | EZK10061 | type-1 fimbrial protein, A chain | Escherichia coli 2-005-03_S1_C3 | 319.2 | 4.3E-92 | Yes |
| 1292 | AB55_5043 | KDT23372 | type-1 fimbrial protein, A chain | Escherichia coli 2-052-05_S1_C3 | 319.2 | 4.3E-92 |  |
| 1293 | AB29_4950 | KDW27279 | type-1 fimbrial protein, A chain | Escherichia coli 2-177-06_S1_C2 | 319.2 | 4.3E-92 |  |
| 1294 | AA97_5002 | EZK35649 | type-1 fimbrial protein, A chain | Escherichia coli 2-005-03_S1_C1 | 319.2 | 4.3E-92 |  |
| 1295 | AB01_5012 | KDW13405 | type-1 fimbrial protein, A chain | Escherichia coli 2-177-06_S1_C1 | 319.2 | 4.3E-92 |  |
| 1296 | AB61_4937 | KDW36413 | type-1 fimbrial protein, A chain | Escherichia coli 2-177-06_S1_C3 | 319.2 | 4.3E-92 |  |
| 1297 | EC07798_0002 | EKI46121 | type-1 fimbrial protein, A chain | Escherichia coli 07798 | 319.1 | 4.4E-92 |  |
| 1298 | ERS139252_00944 | CTX20498 | fimbrial protein | Escherichia coli (GCA_001286345) | 319.1 | 4.4E-92 | Yes |
| 1299 | AB16_1143 | KDZ53187 | type-1 fimbrial protein, A chain | Escherichia coli 3-073-06_S1_C1 | 319.1 | 4.4E-92 |  |
| 1300 | AB80_5572 | KEM15898 | type-1 fimbrial protein, A chain | Escherichia coli 6-319-05_S1_C3 | 319 | 4.9E-92 |  |
| 1301 | G893_00520 | EQV94158 | type-1 fimbrial protein, A chain | Escherichia coli KOEGE 71 (186a) | 318.9 | 5.2E-92 | Yes |
| 1302 | AC789_1c47700 | AJE58919 | type 1 fimbriae major, subunit FimA | Escherichia coli | 318.7 | 5.9E-92 | Yes |
| 1303 | ERS085366_03480 | CTT42074 | fimbrial protein | Escherichia coli | 318.5 | 6.8E-92 | Yes |
| 1304 | PU48_07995 | KHI04086 | type-1 fimbrial protein subunit A | Escherichia coli (GCA_000798115) | 318.5 | 6.9E-92 | Yes |
| 1305 | PU04_13810 | KHJ24840 | type-1 fimbrial protein subunit A | Escherichia coli (GCA_000797735) | 318.5 | 7.1E-92 |  |
| 1306 | BX81_02385 | EYY46066 | type-1 fimbrial protein subunit A | Escherichia coli O165:H25 str. 2010C-4874 | 318.5 | 7.1E-92 | Yes |
| 1307 | EL76_4118 | KGM58315 | Type-1 fimbrial protein, A chain | Escherichia coli G3/10 | 318.3 | 7.7E-92 |  |
| 1308 | AB26_4842 | EZK15589 | type-1 fimbrial protein, A chain | Escherichia coli 2-011-08_S1_C2 | 318.3 | 7.7E-92 |  |
| 1309 | EC2845650_5144 | EMW27648 | type-1 fimbrial protein, A chain | Escherichia coli 2845650 | 318.3 | 7.7E-92 |  |
| 1310 | L403_04591 | ESM26279 | type-1 fimbrial protein, A chain | Escherichia coli BWH 32 | 318.3 | 7.7E-92 |  |
| 1311 | G913_04369 | EQW81436 | type-1 fimbrial protein, A chain | Escherichia coli UMEA 3124-1 | 318.3 | 7.7E-92 | Yes |
| 1312 | PU38_14075 | KIG80492 | type-1 fimbrial protein subunit A | Escherichia coli | 318.3 | 7.7E-92 | Yes |
| 1313 | SD22575_0051 | EIQ65491 | type-1 fimbrial protein, A chain | Shigella dysenteriae 225-75 | 318.3 | 7.7E-92 | Yes |
| 1314 | APU18_05995 | KSZ22665 | type-1 fimbrial protein subunit A | Escherichia coli | 318.3 | 7.9E-92 | Yes |
| 1315 | ERS139208_00959 | CTU76201 | fimbrial protein | Escherichia coli (GCA_001286025) | 318 | 9.5E-92 | Yes |
| 1316 | SB521682_5165 | EGI88516 | type-1 fimbrial protein, A chain | Shigella boydii 5216-82 | 317.7 | 1.2E-91 | Yes |
| 1317 | C827_04198 | EMZ38877 | type-1 fimbrial protein, A chain | Escherichia coli SWW33 | 317.7 | 1.2E-91 | Yes |
| 1318 | AD25_4892 | KDV76258 | type-1 fimbrial protein, A chain | Escherichia coli 2-052-05_S4_C3 | 316.8 | 2.3E-91 |  |
| 1319 | J444_4739 | AKP87389 | type 1 fimbriae major subunit FimA | Escherichia coli ACN001 | 316.7 | 2.4E-91 |  |
| 1320 | APECO78_02710 | AGC85281 | type 1 fimbriae major subunit FimA | Escherichia coli APEC O78 | 316.7 | 2.4E-91 | Yes |
| 1321 | BN16_03781 | CCK49694 | major type 1 subunit fimbrin (pilin) | Escherichia coli chi7122 | 316.7 | 2.4E-91 |  |
| 1322 | G799_04633 | EQR98459 | type-1 fimbrial protein, A chain | Escherichia coli HVH 141 (4-5995973) | 316.4 | 3.1E-91 |  |
| 1323 | A31K_01751 | ELH09232 | fimbrial protein FimA | Escherichia coli KTE165 | 316.4 | 3.1E-91 |  |
| 1324 | G831_04201 | ETF14362 | type-1 fimbrial protein, A chain | Escherichia coli HVH 177 (4-2876612) | 316.4 | 3.1E-91 |  |
| 1325 | G854_04905 | EQU28354 | type-1 fimbrial protein, A chain | Escherichia coli HVH 202 (4-3163997) | 316.4 | 3.1E-91 |  |
| 1326 | G908_04265 | EQW62035 | type-1 fimbrial protein, A chain | Escherichia coli UMEA 3108-1 | 316.4 | 3.1E-91 |  |
| 1327 | WCG_02038 | ELF46851 | fimbrial protein FimA | Escherichia coli KTE6 | 316.4 | 3.1E-91 |  |
| 1328 | G693_04558 | EQN48774 | type-1 fimbrial protein, A chain | Escherichia coli HVH 17 (4-7473087) | 316.4 | 3.1E-91 |  |
| 1329 | G804_04799 | EQS28804 | type-1 fimbrial protein, A chain | Escherichia coli HVH 146 (4-3189767) | 316.4 | 3.1E-91 |  |
| 1330 | G812_04519 | EQS59381 | type-1 fimbrial protein, A chain | Escherichia coli HVH 154 (4-5636698) | 316.4 | 3.1E-91 |  |
| 1331 | G752_04700 | EQQ09822 | type-1 fimbrial protein, A chain | Escherichia coli HVH 90 (4-3191362) | 316.4 | 3.1E-91 |  |
| 1332 | A137_00357 | ELC68312 | type-1 fimbrial protein, A chain | Escherichia coli KTE178 | 316.4 | 3.1E-91 | Yes |
| 1333 | ECDEC14A_4841 | EHX71034 | type-1 fimbrial protein, A chain | Escherichia coli DEC14A | 316.2 | 3.5E-91 | Yes |
| 1334 | SGF_01649 | EFW60914 | type 1 fimbriae major subunit FimA | Shigella flexneri CDC 796-83 | 316.1 | 3.7E-91 |  |
| 1335 | SB444474_5019 | EIQ29296 | type-1 fimbrial protein, A chain | Shigella boydii 4444-74 | 316.1 | 3.7E-91 |  |
| 1336 | ABE81_24285 | AKI69309 | type-1 fimbrial protein subunit A | Shigella boydii serotype 4 (strain Sb227) | 316.1 | 3.7E-91 |  |
| 1337 | SBO_4364 | ABB68780 | major type 1 subunit fimbrin | Shigella boydii Sb227 | 316.1 | 3.7E-91 | Yes |
| 1338 | UH31_18770 | KIZ61415 | type-1 fimbrial protein subunit A | Escherichia coli (GCA_000935475) | 316.1 | 3.7E-91 |  |
| 1339 | SF148580_4850 | EJZ61176 | fimbrial subunit type 1 | Shigella flexneri 1485-80 | 316.1 | 3.7E-91 |  |
| 1340 | SFCCH060_5009 | EIQ02284 | type-1 fimbrial protein, A chain | Shigella flexneri CCH060 | 316.1 | 3.7E-91 |  |
| 1341 | SB359474_5020 | EGI92087 | type-1 fimbrial protein, A chain | Shigella boydii 3594-74 | 316.1 | 3.7E-91 |  |
| 1342 | HW42_03150 | AIZ80943 | type-1 fimbrial protein subunit A | Escherichia coli | 315.8 | 4.8E-91 | Yes |
| 1343 | ECEC1734_0031 | EIP71506 | type-1 fimbrial protein, A chain | Escherichia coli EC1734 | 315.4 | 6.1E-91 |  |
| 1344 | SS17_5467 | AIF96949 | type 1 fimbriae major subunit FimA | Escherichia coli O157:H7 str. SS17 | 315.4 | 6.1E-91 |  |
| 1345 | ECT184097_4996 | ERC65838 | type-1 fimbrial protein, A chain | Escherichia coli T1840_97 | 315.4 | 6.1E-91 |  |
| 1346 | ECPA7_0401 | EKH07696 | type-1 fimbrial protein, A chain | Escherichia coli PA7 | 315.4 | 6.1E-91 |  |
| 1347 | EC970003_0030 | EKW68028 | type-1 fimbrial protein, A chain | Escherichia coli 97.0003 | 315.4 | 6.1E-91 |  |
| 1348 | BW94_20860 | EYZ38295 | type-1 fimbrial protein subunit A | Escherichia coli O157:H7 str. 06-4039 | 315.4 | 6.1E-91 |  |
| 1349 | S3E_0030 | ERD22965 | type-1 fimbrial protein, A chain | Escherichia coli B106 | 315.4 | 6.1E-91 |  |
| 1350 | BY29_17745 | EYW39174 | type-1 fimbrial protein subunit A | Escherichia coli O157:H7 str. 2011EL-2112 | 315.4 | 6.1E-91 |  |
| 1351 | ECDEC3A_5318 | EHU51820 | fimbrial subunit type 1 | Escherichia coli DEC3A | 315.4 | 6.1E-91 |  |
| 1352 | ECEC1737_5618 | EKI59039 | type-1 fimbrial protein, A chain | Escherichia coli EC1737 | 315.4 | 6.1E-91 |  |
| 1353 | ECPA38_5445 | EKI33209 | type-1 fimbrial protein, A chain | Escherichia coli PA38 | 315.4 | 6.1E-91 |  |
| 1354 | EC82524_5564 | EKK52348 | type-1 fimbrial protein, A chain | Escherichia coli 8.2524 | 315.4 | 6.1E-91 |  |
| 1355 | BY69_11235 | EZB98881 | type-1 fimbrial protein subunit A | Escherichia coli O157:H7 str. K2191 | 315.4 | 6.1E-91 |  |
| 1356 | BY85_22415 | EZC64783 | type-1 fimbrial protein subunit A | Escherichia coli O157:H7 str. K5453 | 315.4 | 6.1E-91 |  |
| 1357 | EC60172_0030 | EKK37849 | type-1 fimbrial major subunit | Escherichia coli 6.0172 | 315.4 | 6.1E-91 |  |
| 1358 | ECOSU61_14691 | EFX32603 | Major type 1 subunit fimbrin | Escherichia coli O157:H7 str. LSU-61 | 315.4 | 6.1E-91 |  |
| 1359 | EC990713_0030 | EKW96275 | type-1 fimbrial protein, A chain | Escherichia coli 99.0713 | 315.4 | 6.1E-91 |  |
| 1360 | EC990839_5074 | ELV31206 | type-1 fimbrial protein, A chain | Escherichia coli 99.0839 | 315.4 | 6.1E-91 |  |
| 1361 | S1C_5442 | ERC33949 | type-1 fimbrial protein, A chain | Escherichia coli B93 | 315.4 | 6.1E-91 |  |
| 1362 | ECPA2_0030 | ELV98820 | type-1 fimbrial protein, A chain | Escherichia coli PA2 | 315.4 | 6.1E-91 |  |
| 1363 | ECTW14301_5462 | EIP05618 | type-1 fimbrial protein, A chain | Escherichia coli TW14301 | 315.4 | 6.1E-91 |  |
| 1364 | G2583_5115 | ADD59529 | Major type 1 subunit fimbrin | Escherichia coli O55:H7 str. CB9615 | 315.4 | 6.1E-91 | Yes |
| 1365 | BY63_06790 | EZB71564 | type-1 fimbrial protein subunit A | Escherichia coli O157:H7 str. K1795 | 315.4 | 6.1E-91 |  |
| 1366 | EC940618_5450 | EKW08502 | type-1 fimbrial protein, A chain | Escherichia coli 94.0618 | 315.4 | 6.1E-91 |  |
| 1367 | BY75_13305 | EZC30149 | type-1 fimbrial protein subunit A | Escherichia coli O157:H7 str. K2854 | 315.4 | 6.1E-91 |  |
| 1368 | ECTW10119_0284 | EIO86382 | type-1 fimbrial protein, A chain | Escherichia coli TW10119 | 315.4 | 6.1E-91 |  |
| 1369 | ECTW14313_5567 | EIP03320 | type-1 fimbrial protein, A chain | Escherichia coli TW14313 | 315.4 | 6.1E-91 |  |
| 1370 | BY89_20740 | EZC86965 | type-1 fimbrial protein subunit A | Escherichia coli O157:H7 str. K5607 | 315.4 | 6.1E-91 |  |
| 1371 | ECEC1738_0096 | EIP63333 | type-1 fimbrial protein, A chain | Escherichia coli EC1738 | 315.4 | 6.1E-91 |  |
| 1372 | ECO55CA74_24640 | AEZ43460 | major type 1 subunit fimbrin | Escherichia coli O55:H7 str. RM12579 | 315.4 | 6.1E-91 |  |
| 1373 | ECPA35_0082 | ELW36464 | type-1 fimbrial protein, A chain | Escherichia coli PA35 | 315.4 | 6.1E-91 |  |
| 1374 | ECEC1736_5564 | EKI57102 | type-1 fimbrial protein, A chain | Escherichia coli EC1736 | 315.4 | 6.1E-91 |  |
| 1375 | ECEC4013_0093 | EIP38868 | type-1 fimbrial protein, A chain | Escherichia coli EC4013 | 315.4 | 6.1E-91 |  |
| 1376 | ECDEC4B_5653 | EHU89522 | fimbrial subunit type 1 | Escherichia coli DEC4B | 315.4 | 6.1E-91 |  |
| 1377 | ECPA31_5530 | EIO07001 | type-1 fimbrial protein, A chain | Escherichia coli PA31 | 315.4 | 6.1E-91 |  |
| 1378 | BX83_07525 | EYY46566 | type-1 fimbrial protein subunit A | Escherichia coli O157:H7 str. 2010C-4979C1 | 315.4 | 6.1E-91 |  |
| 1379 | S11_0096 | ERB91821 | type-1 fimbrial protein, A chain | Escherichia coli B26-1 | 315.4 | 6.1E-91 |  |
| 1380 | BY15_00140 | EYX44005 | type-1 fimbrial protein subunit A | Escherichia coli O157:H7 str. 2011EL-2094 | 315.4 | 6.1E-91 |  |
| 1381 | ECoD_01986 | EFW65222 | type 1 fimbriae major subunit FimA | Escherichia coli O157:H7 str. EC1212 | 315.4 | 6.1E-91 |  |
| 1382 | BY73_25965 | EZC08764 | type-1 fimbrial protein subunit A | Escherichia coli O157:H7 str. K2622 | 315.4 | 6.1E-91 |  |
| 1383 | BX01_14845 | EYX02216 | type-1 fimbrial protein subunit A | Escherichia coli O157:H7 str. 08-4169 | 315.4 | 6.1E-91 |  |
| 1384 | ECPA8_0029 | ELW12439 | type-1 fimbrial protein, A chain | Escherichia coli PA8 | 315.4 | 6.1E-91 |  |
| 1385 | ESCCO14588_1208 | EEC30531 | type-1 fimbrial protein homolog | Escherichia coli O157:H7 str. TW14588 | 315.4 | 6.1E-91 |  |
| 1386 | EC08BKT77219_5181 | ERE00897 | type-1 fimbrial protein, A chain | Escherichia coli 08BKT77219 | 315.4 | 6.1E-91 |  |
| 1387 | ECH7EC4206_A4033 | EDZ76748 | type-1 fimbrial protein homolog | Escherichia coli O157:H7 str. EC4206 | 315.4 | 6.1E-91 |  |
| 1388 | S1G_5289 | ERC48275 | type-1 fimbrial protein, A chain | Escherichia coli B95 | 315.4 | 6.1E-91 |  |
| 1389 | BW96_01450 | EYZ36205 | type-1 fimbrial protein subunit A | Escherichia coli O157:H7 str. 07-3391 | 315.4 | 6.1E-91 |  |
| 1390 | ECDEC3C_0108 | EHU79278 | fimbrial subunit type 1 | Escherichia coli DEC3C | 315.4 | 6.1E-91 |  |
| 1391 | ECF_01262 | EGD69117 | type 1 fimbriae major subunit FimA | Escherichia coli O157:H7 str. 1125 | 315.4 | 6.1E-91 |  |
| 1392 | EC970010_5666 | EKY34636 | type-1 fimbrial protein, A chain | Escherichia coli 97.0010 | 315.4 | 6.1E-91 |  |
| 1393 | ECH7EC4045_A0398 | EDZ84515 | type-1 fimbrial protein homolog | Escherichia coli O157:H7 str. EC4045 | 315.4 | 6.1E-91 |  |
| 1394 | BY37_09425 | EYW07445 | type-1 fimbrial protein subunit A | Escherichia coli O157:H7 str. 2011EL-2312 | 315.4 | 6.1E-91 |  |
| 1395 | BY78_01280 | EZC43995 | type-1 fimbrial protein subunit A | Escherichia coli O157:H7 str. K4406 | 315.4 | 6.1E-91 |  |
| 1396 | BY11_12755 | EYX57431 | type-1 fimbrial protein subunit A | Escherichia coli O157:H7 str. 2011EL-2090 | 315.4 | 6.1E-91 |  |
| 1397 | BY92_10570 | EZD08956 | type-1 fimbrial protein subunit A | Escherichia coli O157:H7 str. K5852 | 315.4 | 6.1E-91 |  |
| 1398 | BY76_10745 | EZC37633 | type-1 fimbrial protein subunit A | Escherichia coli O157:H7 str. K4396 | 315.4 | 6.1E-91 |  |
| 1399 | EC01304_5752 | EKJ55012 | type-1 fimbrial protein, A chain | Escherichia coli 0.1304 | 315.4 | 6.1E-91 |  |
| 1400 | ECTW09109_0096 | EIO79116 | type-1 fimbrial protein, A chain | Escherichia coli TW09109 | 315.4 | 6.1E-91 |  |
| 1401 | EC950183_0017 | EKW36782 | type-1 fimbrial protein, A chain | Escherichia coli 95.0183 | 315.4 | 6.1E-91 |  |
| 1402 | ECFDA504_0030 | EKH36331 | type-1 fimbrial protein, A chain | Escherichia coli FDA504 | 315.4 | 6.1E-91 |  |
| 1403 | ECTW10246_0275 | EIO63382 | type-1 fimbrial protein, A chain | Escherichia coli TW10246 | 315.4 | 6.1E-91 |  |
| 1404 | ECDEC3B_5544 | EHU53043 | fimbrial subunit type 1 | Escherichia coli DEC3B | 315.4 | 6.1E-91 |  |
| 1405 | BY84_03640 | EZC77674 | type-1 fimbrial protein subunit A | Escherichia coli O157:H7 str. K5449 | 315.4 | 6.1E-91 |  |
| 1406 | BY24_25840 | EYW64036 | type-1 fimbrial protein subunit A | Escherichia coli O157:H7 str. 2011EL-2106 | 315.4 | 6.1E-91 |  |
| 1407 | BY22_04620 | EYW71163 | type-1 fimbrial protein subunit A | Escherichia coli O157:H7 str. 2011EL-2104 | 315.4 | 6.1E-91 |  |
| 1408 | EC09BKT76207_5482 | ERB68156 | type-1 fimbrial protein, A chain | Escherichia coli 09BKT076207 | 315.4 | 6.1E-91 |  |
| 1409 | ECEC4437_5756 | EIP48983 | type-1 fimbrial protein, A chain | Escherichia coli EC4437 | 315.4 | 6.1E-91 |  |
| 1410 | BY70_17600 | EZC05161 | type-1 fimbrial protein subunit A | Escherichia coli O157:H7 str. K2192 | 315.4 | 6.1E-91 |  |
| 1411 | BY32_03990 | EYW27194 | type-1 fimbrial protein subunit A | Escherichia coli O157:H7 str. 2011EL-2286 | 315.4 | 6.1E-91 |  |
| 1412 | B232_0150 | ERE42130 | type-1 fimbrial protein, A chain | Escherichia coli Tx1686 | 315.4 | 6.1E-91 |  |
| 1413 | B230_0102 | ERC91713 | type-1 fimbrial protein, A chain | Escherichia coli 14A | 315.4 | 6.1E-91 |  |
| 1414 | S1U_0029 | ERD88005 | type-1 fimbrial protein, A chain | Escherichia coli B83 | 315.4 | 6.1E-91 |  |
| 1415 | BY86_08085 | EZC83426 | type-1 fimbrial protein subunit A | Escherichia coli O157:H7 str. K5460 | 315.4 | 6.1E-91 |  |
| 1416 | EC5412_5615 | EKI06075 | type-1 fimbrial protein, A chain | Escherichia coli 5412 | 315.4 | 6.1E-91 |  |
| 1417 | BY87_00145 | EZC90345 | type-1 fimbrial protein subunit A | Escherichia coli O157:H7 str. K5467 | 315.4 | 6.1E-91 |  |
| 1418 | BY51_15810 | EYV89273 | type-1 fimbrial protein subunit A | Escherichia coli O157:H7 str. F7350 | 315.4 | 6.1E-91 |  |
| 1419 | ECEC4196_5737 | EIO92426 | type-1 fimbrial protein, A chain | Escherichia coli EC4196 | 315.4 | 6.1E-91 |  |
| 1420 | ECPA32_5612 | EIO07276 | type-1 fimbrial protein, A chain | Escherichia coli PA32 | 315.4 | 6.1E-91 |  |
| 1421 | EC930055_5498 | EKW04321 | type-1 fimbrial protein, A chain | Escherichia coli 93.0055 | 315.4 | 6.1E-91 |  |
| 1422 | S1K_0032 | ERE36127 | type-1 fimbrial protein, A chain | Escherichia coli B89 | 315.4 | 6.1E-91 |  |
| 1423 | BY48_01455 | EZB05962 | type-1 fimbrial protein subunit A | Escherichia coli O157:H7 str. F6749 | 315.4 | 6.1E-91 |  |
| 1424 | ECEC4436_5588 | EIP40237 | type-1 fimbrial protein, A chain | Escherichia coli EC4436 | 315.4 | 6.1E-91 |  |
| 1425 | BY13_13745 | EYX48122 | type-1 fimbrial protein subunit A | Escherichia coli O157:H7 str. 2011EL-2092 | 315.4 | 6.1E-91 |  |
| 1426 | EC34870_0029 | EKK37409 | type-1 fimbrial protein, A chain | Escherichia coli 3.4870 | 315.4 | 6.1E-91 |  |
| 1427 | ECTT12B_0031 | EKH95582 | type-1 fimbrial protein, A chain | Escherichia coli TT12B | 315.4 | 6.1E-91 |  |
| 1428 | BY30_04740 | EYW34623 | type-1 fimbrial protein subunit A | Escherichia coli O157:H7 str. 2011EL-2113 | 315.4 | 6.1E-91 |  |
| 1429 | EC09BKT24447_0098 | ERE21812 | type-1 fimbrial protein, A chain | Escherichia coli 09BKT024447 | 315.4 | 6.1E-91 |  |
| 1430 | ECEC4448_5645 | EIP49943 | type-1 fimbrial protein, A chain | Escherichia coli EC4448 | 315.4 | 6.1E-91 |  |
| 1431 | EC990816_5166 | ELV30849 | type-1 fimbrial protein, A chain | Escherichia coli 99.0816 | 315.4 | 6.1E-91 |  |
| 1432 | BX05_07285 | EZD90135 | type-1 fimbrial protein subunit A | Escherichia coli O157:NM str. 08-4540 | 315.4 | 6.1E-91 |  |
| 1433 | EC08BKT55439_5133 | ERC53532 | type-1 fimbrial protein, A chain | Escherichia coli 08BKT055439 | 315.4 | 6.1E-91 |  |
| 1434 | ECSP_5398 | ACT75077 | major type 1 subunit fimbrin (pilin) | Escherichia coli O157:H7 str. TW14359 | 315.4 | 6.1E-91 |  |
| 1435 | ECFRIK920_5839 | EKG95799 | type-1 fimbrial protein, A chain | Escherichia coli FRIK920 | 315.4 | 6.1E-91 |  |
| 1436 | BY26_11410 | EYW49154 | type-1 fimbrial protein subunit A | Escherichia coli O157:H7 str. 2011EL-2108 | 315.4 | 6.1E-91 |  |
| 1437 | ECO7815_09073 | EFX22745 | Major type 1 subunit fimbrin | Escherichia coli O55:H7 str. 3256-97 | 315.4 | 6.1E-91 |  |
| 1438 | EC960939_5680 | EKW43922 | type-1 fimbrial protein, A chain | Escherichia coli 96.0939 | 315.4 | 6.1E-91 |  |
| 1439 | ECEC1870_5604 | EKJ36514 | type-1 fimbrial protein, A chain | Escherichia coli EC1870 | 315.4 | 6.1E-91 |  |
| 1440 | Z5912 | AAG59496 | major type 1 subunit fimbrin (pilin) | Escherichia coli O157:H7 str. EDL933 (GCA_000732965) | 315.4 | 6.1E-91 | Yes |
| 1441 | EC881467_0025 | EKV88781 | type-1 fimbrial protein, A chain | Escherichia coli 88.1467 | 315.4 | 6.1E-91 |  |
| 1442 | BY49_01640 | EZB04391 | type-1 fimbrial protein subunit A | Escherichia coli O157:H7 str. F6750 | 315.4 | 6.1E-91 |  |
| 1443 | ECDEC3F_0089 | EHU97586 | fimbrial subunit type 1 | Escherichia coli DEC3F | 315.4 | 6.1E-91 |  |
| 1444 | EC950083_5037 | ELW29966 | type-1 fimbrial protein, A chain | Escherichia coli 95.0083 | 315.4 | 6.1E-91 |  |
| 1445 | BY95_02015 | EZD19476 | type-1 fimbrial protein subunit A | Escherichia coli O157:H7 str. K6687 | 315.4 | 6.1E-91 |  |
| 1446 | BW92_08250 | EYZ52246 | type-1 fimbrial protein subunit A | Escherichia coli O157:H7 str. 06-3745 | 315.4 | 6.1E-91 |  |
| 1447 | ECTW09098_5713 | EIO65773 | type-1 fimbrial protein, A chain | Escherichia coli TW09098 | 315.4 | 6.1E-91 |  |
| 1448 | S1M_0017 | ERE39712 | type-1 fimbrial protein, A chain | Escherichia coli B90 | 315.4 | 6.1E-91 |  |
| 1449 | EC990848_5089 | ELV35371 | type-1 fimbrial protein, A chain | Escherichia coli 99.0848 | 315.4 | 6.1E-91 |  |
| 1450 | EC991775_4975 | ELV47060 | type-1 fimbrial protein, A chain | Escherichia coli 99.1775 | 315.4 | 6.1E-91 |  |
| 1451 | EC93001_5702 | EIN33852 | type-1 fimbrial protein, A chain | Escherichia coli 93-001 | 315.4 | 6.1E-91 |  |
| 1452 | ECPA45_0030 | EKH87916 | type-1 fimbrial protein, A chain | Escherichia coli PA45 | 315.4 | 6.1E-91 |  |
| 1453 | ECT92401_0021 | ERC92384 | type-1 fimbrial protein, A chain | Escherichia coli T924_01 | 315.4 | 6.1E-91 |  |
| 1454 | ECPA28_0031 | EIO09641 | type-1 fimbrial protein, A chain | Escherichia coli PA28 | 315.4 | 6.1E-91 |  |
| 1455 | BY74_13495 | EZC25631 | type-1 fimbrial protein subunit A | Escherichia coli O157:H7 str. K2845 | 315.4 | 6.1E-91 |  |
| 1456 | ECH7EC4401_4305 | EDU76302 | type-1 fimbrial protein homolog | Escherichia coli O157:H7 str. EC4401 | 315.4 | 6.1E-91 |  |
| 1457 | ECNE037_0032 | EKH61352 | type-1 fimbrial protein, A chain | Escherichia coli NE037 | 315.4 | 6.1E-91 |  |
| 1458 | BY54_16075 | EZB30337 | type-1 fimbrial protein subunit A | Escherichia coli O157:H7 str. F7410 | 315.4 | 6.1E-91 |  |
| 1459 | BX33_22515 | EZE88818 | type-1 fimbrial protein subunit A | Escherichia coli O157:H7 str. 2009EL1449 | 315.4 | 6.1E-91 |  |
| 1460 | SS52_5680 | AJA29451 | type 1 fimbriae major subunit FimA | Escherichia coli O157:H7 str. SS52 | 315.4 | 6.1E-91 |  |
| 1461 | ECDEC4D_5334 | EHU99400 | fimbrial subunit type 1 | Escherichia coli DEC4D | 315.4 | 6.1E-91 |  |
| 1462 | ECPA41_5698 | EIO29677 | type-1 fimbrial protein, A chain | Escherichia coli PA41 | 315.4 | 6.1E-91 |  |
| 1463 | ECEC1847_5632 | EKI72720 | type-1 fimbrial protein, A chain | Escherichia coli EC1847 | 315.4 | 6.1E-91 |  |
| 1464 | ECPA9_0029 | EIN68661 | type-1 fimbrial protein, A chain | Escherichia coli PA9 | 315.4 | 6.1E-91 |  |
| 1465 | ECEC1868_0032 | EKJ35934 | type-1 fimbrial protein, A chain | Escherichia coli EC1868 | 315.4 | 6.1E-91 |  |
| 1466 | EC991781_0017 | ELW23883 | type-1 fimbrial protein, A chain | Escherichia coli 99.1781 | 315.4 | 6.1E-91 |  |
| 1467 | ACP61_05335 | KOZ32824 | type-1 fimbrial protein subunit A | Escherichia coli O55:H7 (strain CB9615 / EPEC) | 315.4 | 6.1E-91 |  |
| 1468 | ECO9389_14658 | EFX13475 | Major type 1 subunit fimbrin | Escherichia coli O157:H- str. 493-89 | 315.4 | 6.1E-91 |  |
| 1469 | BY72_11030 | EZC17273 | type-1 fimbrial protein subunit A | Escherichia coli O157:H7 str. K2581 | 315.4 | 6.1E-91 |  |
| 1470 | EC34880_5145 | ELW28332 | type-1 fimbrial protein, A chain | Escherichia coli 3.4880 | 315.4 | 6.1E-91 |  |
| 1471 | ECO2687_22254 | EFX18252 | Major type 1 subunit fimbrin | Escherichia coli O157:H- str. H 2687 | 315.4 | 6.1E-91 |  |
| 1472 | BY67_04755 | EZB80123 | type-1 fimbrial protein subunit A | Escherichia coli O157:H7 str. K1927 | 315.4 | 6.1E-91 |  |
| 1473 | ECFRIK1990_5779 | EIN35125 | type-1 fimbrial protein, A chain | Escherichia coli FRIK1990 | 315.4 | 6.1E-91 |  |
| 1474 | ECEC4439_5634 | EIP35304 | type-1 fimbrial protein, A chain | Escherichia coli EC4439 | 315.4 | 6.1E-91 |  |
| 1475 | ECPA10_5909 | EIN65429 | type-1 fimbrial protein, A chain | Escherichia coli PA10 | 315.4 | 6.1E-91 |  |
| 1476 | CDCO157_4958 | AFJ32006 | FimA | Escherichia coli Xuzhou21 | 315.4 | 6.1E-91 |  |
| 1477 | S1I_0031 | ERD08486 | type-1 fimbrial protein, A chain | Escherichia coli B103 | 315.4 | 6.1E-91 |  |
| 1478 | ECEC1864_0030 | EKJ19840 | type-1 fimbrial protein, A chain | Escherichia coli EC1864 | 315.4 | 6.1E-91 |  |
| 1479 | EC900039_5460 | EKV91500 | type-1 fimbrial protein, A chain | Escherichia coli 90.0039 | 315.4 | 6.1E-91 |  |
| 1480 | S35_0029 | ERD05035 | type-1 fimbrial protein, A chain | Escherichia coli B104 | 315.4 | 6.1E-91 |  |
| 1481 | Q455_0213270 | ETJ79067 | type-1 fimbrial protein subunit A | Escherichia coli ATCC BAA-2192 | 315.4 | 6.1E-91 |  |
| 1482 | S1O_5044 | ERD40247 | type-1 fimbrial protein, A chain | Escherichia coli B15 | 315.4 | 6.1E-91 |  |
| 1483 | ECEC1862_5614 | EKJ00657 | type-1 fimbrial protein, A chain | Escherichia coli EC1862 | 315.4 | 6.1E-91 |  |
| 1484 | BZ06_06580 | EZD75280 | type-1 fimbrial protein subunit A | Escherichia coli O157:H7 str. K7140 | 315.4 | 6.1E-91 |  |
| 1485 | ECEC1850_0046 | EKJ05420 | type-1 fimbrial protein, A chain | Escherichia coli EC1850 | 315.4 | 6.1E-91 |  |
| 1486 | BW98_00995 | EYX13640 | type-1 fimbrial protein subunit A | Escherichia coli O157:H7 str. 08-3037 | 315.4 | 6.1E-91 |  |
| 1487 | BY88_08890 | EZC92783 | type-1 fimbrial protein subunit A | Escherichia coli O157:H7 str. K5602 | 315.4 | 6.1E-91 |  |
| 1488 | ECPA25_5446 | EIN89788 | type-1 fimbrial protein, A chain | Escherichia coli PA25 | 315.4 | 6.1E-91 |  |
| 1489 | BY57_07555 | EZB41536 | type-1 fimbrial protein subunit A | Escherichia coli O157:H7 str. H2495 | 315.4 | 6.1E-91 |  |
| 1490 | EC990678_0094 | EKW96671 | type-1 fimbrial major subunit | Escherichia coli 99.0678 | 315.4 | 6.1E-91 |  |
| 1491 | S3A_0030 | ERD73413 | type-1 fimbrial protein, A chain | Escherichia coli B49-2 | 315.4 | 6.1E-91 |  |
| 1492 | EC990815_5059 | ELV23608 | type-1 fimbrial protein, A chain | Escherichia coli 99.0815 | 315.4 | 6.1E-91 |  |
| 1493 | EC900091_0097 | EKW05787 | type-1 fimbrial protein, A chain | Escherichia coli 90.0091 | 315.4 | 6.1E-91 |  |
| 1494 | ECFDA507_5679 | EKH13368 | type-1 fimbrial protein, A chain | Escherichia coli FDA507 | 315.4 | 6.1E-91 |  |
| 1495 | EC890511_5600 | EKV70668 | type-1 fimbrial protein, A chain | Escherichia coli 89.0511 | 315.4 | 6.1E-91 |  |
| 1496 | ECDEC4F_5255 | EHV16593 | fimbrial subunit type 1 | Escherichia coli DEC4F | 315.4 | 6.1E-91 |  |
| 1497 | BY77_00615 | EZC28536 | type-1 fimbrial protein subunit A | Escherichia coli O157:H7 str. K4405 | 315.4 | 6.1E-91 |  |
| 1498 | S33_0031 | ERD24006 | type-1 fimbrial protein, A chain | Escherichia coli B108 | 315.4 | 6.1E-91 |  |
| 1499 | BY59_23300 | EZB48650 | type-1 fimbrial protein subunit A | Escherichia coli O157:H7 str. K1420 | 315.4 | 6.1E-91 |  |
| 1500 | ECEC1846_5610 | EKI64790 | type-1 fimbrial protein, A chain | Escherichia coli EC1846 | 315.4 | 6.1E-91 |  |
| 1501 | ECEC4421_5507 | EIP10314 | type-1 fimbrial protein, A chain | Escherichia coli EC4421 | 315.4 | 6.1E-91 |  |
| 1502 | EC100833_0098 | EKK67956 | type-1 fimbrial major subunit | Escherichia coli 10.0833 | 315.4 | 6.1E-91 |  |
| 1503 | ECDEC5B_0019 | EHV37787 | fimbrial subunit type 1 | Escherichia coli DEC5B | 315.4 | 6.1E-91 |  |
| 1504 | ECDEC4A_5329 | EHU86077 | fimbrial subunit type 1 | Escherichia coli DEC4A | 315.4 | 6.1E-91 |  |
| 1505 | EC960932_0092 | EKW70471 | type-1 fimbrial protein, A chain | Escherichia coli 96.0932 | 315.4 | 6.1E-91 |  |
| 1506 | BY61_02760 | EZB59822 | type-1 fimbrial protein subunit A | Escherichia coli O157:H7 str. K1792 | 315.4 | 6.1E-91 |  |
| 1507 | S17_0031 | ERD68392 | type-1 fimbrial protein, A chain | Escherichia coli B40-2 | 315.4 | 6.1E-91 |  |
| 1508 | BY52_09025 | EZB20608 | type-1 fimbrial protein subunit A | Escherichia coli O157:H7 str. F7377 | 315.4 | 6.1E-91 |  |
| 1509 | EC951288_5377 | EKW22394 | type-1 fimbrial protein, A chain | Escherichia coli 95.1288 | 315.4 | 6.1E-91 |  |
| 1510 | BY25_21460 | EYW57164 | type-1 fimbrial protein subunit A | Escherichia coli O157:H7 str. 2011EL-2107 | 315.4 | 6.1E-91 |  |
| 1511 | S1E_5455 | ERC40356 | type-1 fimbrial protein, A chain | Escherichia coli B94 | 315.4 | 6.1E-91 |  |
| 1512 | ECPA47_5046 | ELV91657 | type-1 fimbrial protein, A chain | Escherichia coli PA47 | 315.4 | 6.1E-91 |  |
| 1513 | EC52239_0030 | EKK36735 | type-1 fimbrial protein, A chain | Escherichia coli 5.2239 | 315.4 | 6.1E-91 |  |
| 1514 | QYO_0031 | ERC15201 | type-1 fimbrial protein, A chain | Escherichia coli B29-1 | 315.4 | 6.1E-91 |  |
| 1515 | ECDEC5C_5260 | EHV31655 | fimbrial subunit type 1 | Escherichia coli DEC5C | 315.4 | 6.1E-91 |  |
| 1516 | BY68_02615 | EZB81707 | type-1 fimbrial protein subunit A | Escherichia coli O157:H7 str. K2188 | 315.4 | 6.1E-91 |  |
| 1517 | B231_0099 | ERE45425 | type-1 fimbrial protein, A chain | Escherichia coli Tx3800 | 315.4 | 6.1E-91 |  |
| 1518 | ECPA19_4664 | ELV78215 | type-1 fimbrial protein, A chain | Escherichia coli PA19 | 315.4 | 6.1E-91 |  |
| 1519 | ECPA15_0031 | EIN85413 | type-1 fimbrial protein, A chain | Escherichia coli PA15 | 315.4 | 6.1E-91 |  |
| 1520 | EC960109_5699 | EKY34549 | type-1 fimbrial protein, A chain | Escherichia coli 96.0109 | 315.4 | 6.1E-91 |  |
| 1521 | B233_0030 | ERD09279 | type-1 fimbrial protein, A chain | Escherichia coli 2886-75 | 315.4 | 6.1E-91 |  |
| 1522 | ECH7EC4113_5504 | EDU54326 | type-1 fimbrial protein homolog | Escherichia coli O157:H7 str. EC4113 | 315.4 | 6.1E-91 |  |
| 1523 | ECPA22_0094 | EIN94420 | type-1 fimbrial protein, A chain | Escherichia coli PA22 | 315.4 | 6.1E-91 |  |
| 1524 | EC80416_5199 | EKK71782 | type-1 fimbrial major subunit | Escherichia coli 8.0416 | 315.4 | 6.1E-91 |  |
| 1525 | BW99_13295 | EYX12315 | type-1 fimbrial protein subunit A | Escherichia coli O157:H7 str. 08-3527 | 315.4 | 6.1E-91 |  |
| 1526 | BY12_10560 | EYX54536 | type-1 fimbrial protein subunit A | Escherichia coli O157:H7 str. 2011EL-2091 | 315.4 | 6.1E-91 |  |
| 1527 | BX04_24435 | EZD85342 | type-1 fimbrial protein subunit A | Escherichia coli O157:H7 str. 08-4529 | 315.4 | 6.1E-91 |  |
| 1528 | ECPA33_5592 | EIO11132 | type-1 fimbrial protein, A chain | Escherichia coli PA33 | 315.4 | 6.1E-91 |  |
| 1529 | ECFDA505_5583 | EIN15790 | type-1 fimbrial protein, A chain | Escherichia coli FDA505 | 315.4 | 6.1E-91 |  |
| 1530 | EC930056_0029 | EKW19039 | type-1 fimbrial protein, A chain | Escherichia coli 93.0056 | 315.4 | 6.1E-91 |  |
| 1531 | BY62_06940 | EZB55869 | type-1 fimbrial protein subunit A | Escherichia coli O157:H7 str. K1793 | 315.4 | 6.1E-91 |  |
| 1532 | EC71982_0039 | ELW21820 | type-1 fimbrial protein, A chain | Escherichia coli 7.1982 | 315.4 | 6.1E-91 |  |
| 1533 | BY71_01725 | EZB95430 | type-1 fimbrial protein subunit A | Escherichia coli O157:H7 str. K2324 | 315.4 | 6.1E-91 |  |
| 1534 | EC950943_0029 | EKW36592 | type-1 fimbrial protein, A chain | Escherichia coli 95.0943 | 315.4 | 6.1E-91 |  |
| 1535 | S1W_0031 | ERD91791 | type-1 fimbrial protein, A chain | Escherichia coli B84 | 315.4 | 6.1E-91 |  |
| 1536 | BY19_06045 | EYW85320 | type-1 fimbrial protein subunit A | Escherichia coli O157:H7 str. 2011EL-2099 | 315.4 | 6.1E-91 |  |
| 1537 | ECMA6_0144 | EKI01279 | type-1 fimbrial protein, A chain | Escherichia coli MA6 | 315.4 | 6.1E-91 |  |
| 1538 | ECTW09195_5725 | EIO86194 | type-1 fimbrial protein, A chain | Escherichia coli TW09195 | 315.4 | 6.1E-91 |  |
| 1539 | EC880221_0096 | EKK87682 | type-1 fimbrial protein, A chain | Escherichia coli 88.0221 | 315.4 | 6.1E-91 |  |
| 1540 | BY94_01455 | EZD15745 | type-1 fimbrial protein subunit A | Escherichia coli O157:H7 str. K6676 | 315.4 | 6.1E-91 |  |
| 1541 | ECPA40_0103 | EIO32411 | type-1 fimbrial protein, A chain | Escherichia coli PA40 | 315.4 | 6.1E-91 |  |
| 1542 | ECFRIK2001_0277 | EKH63182 | type-1 fimbrial protein, A chain | Escherichia coli FRIK2001 | 315.4 | 6.1E-91 |  |
| 1543 | ECEC1848_0094 | EKI91885 | type-1 fimbrial protein, A chain | Escherichia coli EC1848 | 315.4 | 6.1E-91 |  |
| 1544 | ECEC1869_0030 | EKJ47678 | type-1 fimbrial protein, A chain | Escherichia coli EC1869 | 315.4 | 6.1E-91 |  |
| 1545 | ECPA3_5718 | EIN49127 | type-1 fimbrial protein, A chain | Escherichia coli PA3 | 315.4 | 6.1E-91 |  |
| 1546 | BX89_02475 | EZQ47406 | type-1 fimbrial protein subunit A | Escherichia coli O157: str. 2010EL-2044 | 315.4 | 6.1E-91 |  |
| 1547 | ECPA5_5564 | EIN52282 | type-1 fimbrial protein, A chain | Escherichia coli PA5 | 315.4 | 6.1E-91 |  |
| 1548 | EC960107_0029 | EKW74724 | type-1 fimbrial protein, A chain | Escherichia coli 96.0107 | 315.4 | 6.1E-91 |  |
| 1549 | ECBD561099_5212 | ERC62108 | type-1 fimbrial protein, A chain | Escherichia coli Bd5610_99 | 315.4 | 6.1E-91 |  |
| 1550 | S3G_0031 | ERD37199 | type-1 fimbrial protein, A chain | Escherichia coli B112 | 315.4 | 6.1E-91 |  |
| 1551 | EC990670_0031 | ELW46386 | type-1 fimbrial protein, A chain | Escherichia coli 99.0670 | 315.4 | 6.1E-91 |  |
| 1552 | ECH7EC4076_2473 | EDU68758 | type-1 fimbrial protein homolog | Escherichia coli O157:H7 str. EC4076 | 315.4 | 6.1E-91 |  |
| 1553 | ECH7EC4196_1025 | EDU34025 | type-1 fimbrial protein homolog | Escherichia coli O157:H7 str. EC4196 | 315.4 | 6.1E-91 |  |
| 1554 | BX34_16685 | EYV70468 | type-1 fimbrial protein subunit A | Escherichia coli O157:H7 str. 2009EL1705 | 315.4 | 6.1E-91 |  |
| 1555 | ECTW06591_5268 | EIO45841 | type-1 fimbrial protein, A chain | Escherichia coli TW06591 | 315.4 | 6.1E-91 |  |
| 1556 | EC80586_0030 | EKK61104 | type-1 fimbrial protein, A chain | Escherichia coli 8.0586 | 315.4 | 6.1E-91 |  |
| 1557 | QYS_4920 | ERC10675 | type-1 fimbrial protein, A chain | Escherichia coli B36-1 | 315.4 | 6.1E-91 |  |
| 1558 | BX36_01485 | EYV67660 | type-1 fimbrial protein subunit A | Escherichia coli O157:H7 str. 2009EL2109 | 315.4 | 6.1E-91 |  |
| 1559 | BY16_15970 | EYX32992 | type-1 fimbrial protein subunit A | Escherichia coli O157:H7 str. 2011EL-2096 | 315.4 | 6.1E-91 |  |
| 1560 | BY93_18435 | EZD17507 | type-1 fimbrial protein subunit A | Escherichia coli O157:H7 str. K6590 | 315.4 | 6.1E-91 |  |
| 1561 | ECFRIK1997_0032 | EKH48846 | type-1 fimbrial protein, A chain | Escherichia coli FRIK1997 | 315.4 | 6.1E-91 |  |
| 1562 | BY58_11985 | EZB46997 | type-1 fimbrial protein subunit A | Escherichia coli O157:H7 str. H2498 | 315.4 | 6.1E-91 |  |
| 1563 | BY35_00430 | EYW13514 | type-1 fimbrial protein subunit A | Escherichia coli O157:H7 str. 2011EL-2289 | 315.4 | 6.1E-91 |  |
| 1564 | BY28_12320 | EYW43036 | type-1 fimbrial protein subunit A | Escherichia coli O157:H7 str. 2011EL-2111 | 315.4 | 6.1E-91 |  |
| 1565 | S3K_0031 | ERD52945 | type-1 fimbrial protein, A chain | Escherichia coli B114 | 315.4 | 6.1E-91 |  |
| 1566 | BX35_01270 | EZE89461 | type-1 fimbrial protein subunit A | Escherichia coli O157:H7 str. 2009EL1913 | 315.4 | 6.1E-91 |  |
| 1567 | ECFRIK1996_5648 | EIN15663 | type-1 fimbrial protein, A chain | Escherichia coli FRIK1996 | 315.4 | 6.1E-91 |  |
| 1568 | ECEC1849_5553 | EKI82690 | type-1 fimbrial protein, A chain | Escherichia coli EC1849 | 315.4 | 6.1E-91 |  |
| 1569 | S1Y_0031 | ERD98698 | type-1 fimbrial protein, A chain | Escherichia coli B85 | 315.4 | 6.1E-91 |  |
| 1570 | EC881042_0030 | EKV85114 | type-1 fimbrial protein, A chain | Escherichia coli 88.1042 | 315.4 | 6.1E-91 |  |
| 1571 | BY64_13430 | EZB74702 | type-1 fimbrial protein subunit A | Escherichia coli O157:H7 str. K1796 | 315.4 | 6.1E-91 |  |
| 1572 | ECPA49_0030 | EKH83353 | type-1 fimbrial protein, A chain | Escherichia coli PA49 | 315.4 | 6.1E-91 |  |
| 1573 | ECPA34_0030 | EKH20932 | type-1 fimbrial protein, A chain | Escherichia coli PA34 | 315.4 | 6.1E-91 |  |
| 1574 | ECEC4422_0028 | EIP33957 | type-1 fimbrial protein, A chain | Escherichia coli EC4422 | 315.4 | 6.1E-91 |  |
| 1575 | QYK_0029 | ERC08326 | type-1 fimbrial protein, A chain | Escherichia coli B28-1 | 315.4 | 6.1E-91 |  |
| 1576 | ECT23400_5278 | ERC74202 | type-1 fimbrial protein, A chain | Escherichia coli T234_00 | 315.4 | 6.1E-91 |  |
| 1577 | ECPA42_0031 | EIO49136 | type-1 fimbrial protein, A chain | Escherichia coli PA42 | 315.4 | 6.1E-91 |  |
| 1578 | ECPA13_4915 | ELV76711 | type-1 fimbrial protein, A chain | Escherichia coli PA13 | 315.4 | 6.1E-91 |  |
| 1579 | ECFDA506_0354 | EKH25301 | type-1 fimbrial protein, A chain | Escherichia coli FDA506 | 315.4 | 6.1E-91 |  |
| 1580 | BW89_03570 | EYZ63317 | type-1 fimbrial protein subunit A | Escherichia coli O55:H7 str. 06-3555 | 315.4 | 6.1E-91 |  |
| 1581 | BY33_00430 | EYW24523 | type-1 fimbrial protein subunit A | Escherichia coli O157:H7 str. 2011EL-2287 | 315.4 | 6.1E-91 |  |
| 1582 | EC902281_0029 | EKW03209 | type-1 fimbrial protein, A chain | Escherichia coli 90.2281 | 315.4 | 6.1E-91 |  |
| 1583 | QYC_0030 | ERB88989 | type-1 fimbrial protein, A chain | Escherichia coli B102 | 315.4 | 6.1E-91 |  |
| 1584 | BY38_12470 | EZF05469 | type-1 fimbrial protein subunit A | Escherichia coli O157:H7 str. 2011EL-2313 | 315.4 | 6.1E-91 |  |
| 1585 | BY66_06810 | EZB87923 | type-1 fimbrial protein subunit A | Escherichia coli O157:H7 str. K1921 | 315.4 | 6.1E-91 |  |
| 1586 | QYE_0098 | ERB79231 | type-1 fimbrial protein, A chain | Escherichia coli B107 | 315.4 | 6.1E-91 |  |
| 1587 | ECPA24_5477 | EIN88109 | type-1 fimbrial protein, A chain | Escherichia coli PA24 | 315.4 | 6.1E-91 |  |
| 1588 | BY34_23140 | EYW09893 | type-1 fimbrial protein subunit A | Escherichia coli O157:H7 str. 2011EL-2288 | 315.4 | 6.1E-91 |  |
| 1589 | BX22_21815 | EZE61432 | type-1 fimbrial protein subunit A | Escherichia coli O157:H7 str. 2009C-4258 | 315.4 | 6.1E-91 |  |
| 1590 | BY91_01465 | EYV84711 | type-1 fimbrial protein subunit A | Escherichia coli O157:H7 str. K5806 | 315.4 | 6.1E-91 |  |
| 1591 | BY65_09965 | EZB77236 | type-1 fimbrial protein subunit A | Escherichia coli O157:H7 str. K1845 | 315.4 | 6.1E-91 |  |
| 1592 | ECDEC4E_5297 | EHV06157 | fimbrial subunit type 1 | Escherichia coli DEC4E | 315.4 | 6.1E-91 |  |
| 1593 | QYQ_0015 | ERC23460 | type-1 fimbrial protein, A chain | Escherichia coli B29-2 | 315.4 | 6.1E-91 |  |
| 1594 | BY14_02855 | EYX44869 | type-1 fimbrial protein subunit A | Escherichia coli O157:H7 str. 2011EL-2093 | 315.4 | 6.1E-91 |  |
| 1595 | EC09BKT78844_5715 | ELV15324 | type-1 fimbrial protein, A chain | Escherichia coli 09BKT078844 | 315.4 | 6.1E-91 |  |
| 1596 | BY17_20295 | EYX30684 | type-1 fimbrial protein subunit A | Escherichia coli O157:H7 str. 2011EL-2097 | 315.4 | 6.1E-91 |  |
| 1597 | QYI_5233 | ERC30076 | type-1 fimbrial protein, A chain | Escherichia coli B7-2 | 315.4 | 6.1E-91 |  |
| 1598 | BY27_03910 | EYW49467 | type-1 fimbrial protein subunit A | Escherichia coli O157:H7 str. 2011EL-2109 | 315.4 | 6.1E-91 |  |
| 1599 | ECEC1735_5663 | EKI46633 | type-1 fimbrial protein, A chain | Escherichia coli EC1735 | 315.4 | 6.1E-91 |  |
| 1600 | BY45_26425 | EZA89739 | type-1 fimbrial protein subunit A | Escherichia coli O157:H7 str. F6142 | 315.4 | 6.1E-91 |  |
| 1601 | S3I_0017 | ERD44616 | type-1 fimbrial protein, A chain | Escherichia coli B113 | 315.4 | 6.1E-91 |  |
| 1602 | EC991753_5089 | ELV44351 | type-1 fimbrial protein, A chain | Escherichia coli 99.1753 | 315.4 | 6.1E-91 |  |
| 1603 | BY56_10140 | EZB32027 | type-1 fimbrial protein subunit A | Escherichia coli O157:H7 str. G5303 | 315.4 | 6.1E-91 |  |
| 1604 | BW95_22280 | EYZ37428 | type-1 fimbrial protein subunit A | Escherichia coli O157:H7 str. 07-3091 | 315.4 | 6.1E-91 |  |
| 1605 | BY36_24470 | EZF08548 | type-1 fimbrial protein subunit A | Escherichia coli O157:H7 str. 2011EL-2290 | 315.4 | 6.1E-91 |  |
| 1606 | BY18_15095 | EYX27773 | type-1 fimbrial protein subunit A | Escherichia coli O157:H7 str. 2011EL-2098 | 315.4 | 6.1E-91 |  |
| 1607 | ECFRIK523_5604 | EKJ48145 | type-1 fimbrial protein, A chain | Escherichia coli FRIK523 | 315.4 | 6.1E-91 |  |
| 1608 | S37_0031 | ERD35063 | type-1 fimbrial protein, A chain | Escherichia coli B109 | 315.4 | 6.1E-91 |  |
| 1609 | BY21_12420 | EYW75533 | type-1 fimbrial protein subunit A | Escherichia coli O157:H7 str. 2011EL-2103 | 315.4 | 6.1E-91 |  |
| 1610 | ECDEC3E_0017 | EHU85632 | fimbrial subunit type 1 | Escherichia coli DEC3E | 315.4 | 6.1E-91 |  |
| 1611 | BY23_05540 | EYW67167 | type-1 fimbrial protein subunit A | Escherichia coli O157:H7 str. 2011EL-2105 | 315.4 | 6.1E-91 |  |
| 1612 | ECFDA517_5910 | EIN16647 | type-1 fimbrial protein, A chain | Escherichia coli FDA517 | 315.4 | 6.1E-91 |  |
| 1613 | ECEC4402_5668 | EIP27265 | type-1 fimbrial protein, A chain | Escherichia coli EC4402 | 315.4 | 6.1E-91 |  |
| 1614 | BY09_01260 | EYX70072 | type-1 fimbrial protein subunit A | Escherichia coli O157:H7 str. 2011EL-1107 | 315.4 | 6.1E-91 |  |
| 1615 | BY82_00170 | EZC62943 | type-1 fimbrial protein subunit A | Escherichia coli O157:H7 str. K5418 | 315.4 | 6.1E-91 |  |
| 1616 | EC960427_5622 | EKW38076 | type-1 fimbrial protein, A chain | Escherichia coli 96.0427 | 315.4 | 6.1E-91 |  |
| 1617 | ECTW11039_0032 | EIO69673 | type-1 fimbrial protein, A chain | Escherichia coli TW11039 | 315.4 | 6.1E-91 |  |
| 1618 | S1Q_4936 | ERD45143 | type-1 fimbrial protein, A chain | Escherichia coli B17 | 315.4 | 6.1E-91 |  |
| 1619 | ECFRIK1985_5876 | EIN33004 | type-1 fimbrial protein, A chain | Escherichia coli FRIK1985 | 315.4 | 6.1E-91 |  |
| 1620 | EC990672_5675 | EKW76233 | type-1 fimbrial protein, A chain | Escherichia coli 99.0672 | 315.4 | 6.1E-91 |  |
| 1621 | S13_0099 | ERC00118 | type-1 fimbrial protein, A chain | Escherichia coli B26-2 | 315.4 | 6.1E-91 |  |
| 1622 | BX90_01455 | EZQ50270 | type-1 fimbrial protein subunit A | Escherichia coli O157: str. 2010EL-2045 | 315.4 | 6.1E-91 |  |
| 1623 | BY83_24020 | EZC70434 | type-1 fimbrial protein subunit A | Escherichia coli O157:H7 str. K5448 | 315.4 | 6.1E-91 |  |
| 1624 | BY31_07810 | EYW25800 | type-1 fimbrial protein subunit A | Escherichia coli O157:H7 str. 2011EL-2114 | 315.4 | 6.1E-91 |  |
| 1625 | S15_0031 | ERD72613 | type-1 fimbrial protein, A chain | Escherichia coli B40-1 | 315.4 | 6.1E-91 |  |
| 1626 | EC991793_0017 | ELV62475 | type-1 fimbrial protein, A chain | Escherichia coli 99.1793 | 315.4 | 6.1E-91 |  |
| 1627 | BY50_12650 | EZB11553 | type-1 fimbrial protein subunit A | Escherichia coli O157:H7 str. F6751 | 315.4 | 6.1E-91 |  |
| 1628 | EC990814_5082 | ELV14500 | type-1 fimbrial protein, A chain | Escherichia coli 99.0814 | 315.4 | 6.1E-91 |  |
| 1629 | QYY_0031 | ERD83203 | type-1 fimbrial protein, A chain | Escherichia coli B5-2 | 315.4 | 6.1E-91 |  |
| 1630 | XF37_26800 | KKF80412 | type-1 fimbrial protein subunit A | Escherichia coli O157:H7 | 315.4 | 6.1E-91 |  |
| 1631 | S3C_0032 | ERD20762 | type-1 fimbrial protein, A chain | Escherichia coli B105 | 315.4 | 6.1E-91 |  |
| 1632 | ECEC4203_5686 | EIO87462 | type-1 fimbrial protein, A chain | Escherichia coli EC4203 | 315.4 | 6.1E-91 |  |
| 1633 | ECT128201_5099 | ERE14343 | type-1 fimbrial protein, A chain | Escherichia coli T1282_01 | 315.4 | 6.1E-91 |  |
| 1634 | ECH7EC869_5302 | EDU93255 | type-1 fimbrial protein homolog | Escherichia coli O157:H7 str. EC869 | 315.4 | 6.1E-91 |  |
| 1635 | ECH7EC4501_2828 | EDU87410 | type-1 fimbrial protein homolog | Escherichia coli O157:H7 str. EC4501 | 315.4 | 6.1E-91 |  |
| 1636 | ECTW07945_0024 | EIO79473 | type-1 fimbrial protein, A chain | Escherichia coli TW07945 | 315.4 | 6.1E-91 |  |
| 1637 | ECNE1487_0148 | EKH53695 | type-1 fimbrial protein, A chain | Escherichia coli NE1487 | 315.4 | 6.1E-91 |  |
| 1638 | ECDEC5D_0188 | EHV46610 | fimbrial subunit type 1 | Escherichia coli DEC5D | 315.4 | 6.1E-91 |  |
| 1639 | ECATCC700728_5056 | ELV63274 | type-1 fimbrial protein, A chain | Escherichia coli ATCC 700728 | 315.4 | 6.1E-91 |  |
| 1640 | EC100821_0030 | EKK91198 | type-1 fimbrial protein, A chain | Escherichia coli 10.0821 | 315.4 | 6.1E-91 |  |
| 1641 | QYU_0029 | ERC32320 | type-1 fimbrial protein, A chain | Escherichia coli B36-2 | 315.4 | 6.1E-91 |  |
| 1642 | S31_4991 | ERD88445 | type-1 fimbrial protein, A chain | Escherichia coli B86 | 315.4 | 6.1E-91 |  |
| 1643 | EC971742_5127 | EKW68837 | type-1 fimbrial protein, A chain | Escherichia coli 97.1742 | 315.4 | 6.1E-91 |  |
| 1644 | ECPA23_5620 | EKH63864 | type-1 fimbrial protein, A chain | Escherichia coli PA23 | 315.4 | 6.1E-91 |  |
| 1645 | BY79_01460 | EZC48736 | type-1 fimbrial protein subunit A | Escherichia coli O157:H7 str. K4527 | 315.4 | 6.1E-91 |  |
| 1646 | ECEC1856_5605 | EKI92968 | type-1 fimbrial protein, A chain | Escherichia coli EC1856 | 315.4 | 6.1E-91 |  |
| 1647 | EC970007_5013 | EKW71386 | type-1 fimbrial protein, A chain | Escherichia coli 97.0007 | 315.4 | 6.1E-91 |  |
| 1648 | ECPA11_0029 | ELV75982 | type-1 fimbrial protein, A chain | Escherichia coli PA11 | 315.4 | 6.1E-91 |  |
| 1649 | ECNE098_0030 | EKJ53081 | type-1 fimbrial protein, A chain | Escherichia coli NE098 | 315.4 | 6.1E-91 |  |
| 1650 | ECDEC3D_5453 | EHU68122 | fimbrial subunit type 1 | Escherichia coli DEC3D | 315.4 | 6.1E-91 |  |
| 1651 | QYG_5327 | ERC25325 | type-1 fimbrial protein, A chain | Escherichia coli B7-1 | 315.4 | 6.1E-91 |  |
| 1652 | ECEC1863_5353 | EIP72941 | type-1 fimbrial protein, A chain | Escherichia coli EC1863 | 315.4 | 6.1E-91 |  |
| 1653 | EC960428_0031 | EKW51263 | type-1 fimbrial protein, A chain | Escherichia coli 96.0428 | 315.4 | 6.1E-91 |  |
| 1654 | BY20_01455 | EYW83353 | type-1 fimbrial protein subunit A | Escherichia coli O157:H7 str. 2011EL-2101 | 315.4 | 6.1E-91 |  |
| 1655 | QYM_0031 | ERC07696 | type-1 fimbrial protein, A chain | Escherichia coli B28-2 | 315.4 | 6.1E-91 |  |
| 1656 | ECEC1866_5502 | EKJ20879 | type-1 fimbrial protein, A chain | Escherichia coli EC1866 | 315.4 | 6.1E-91 |  |
| 1657 | ECEC1845_5595 | EIP72267 | type-1 fimbrial protein, A chain | Escherichia coli EC1845 | 315.4 | 6.1E-91 |  |
| 1658 | EC991805_5002 | ELV61602 | type-1 fimbrial protein, A chain | Escherichia coli 99.1805 | 315.4 | 6.1E-91 |  |
| 1659 | ECCB7326_5676 | EKH96698 | type-1 fimbrial protein, A chain | Escherichia coli CB7326 | 315.4 | 6.1E-91 |  |
| 1660 | ECH74042_A0954 | EDZ86250 | type-1 fimbrial protein homolog | Escherichia coli O157:H7 str. EC4042 | 315.4 | 6.1E-91 |  |
| 1661 | ECH7EC508_2466 | EDU97843 | type-1 fimbrial protein homolog | Escherichia coli O157:H7 str. EC508 | 315.4 | 6.1E-91 |  |
| 1662 | ECPA14_5737 | EIN69773 | type-1 fimbrial protein, A chain | Escherichia coli PA14 | 315.4 | 6.1E-91 |  |
| 1663 | ECDEC4C_5592 | EHV00436 | fimbrial subunit type 1 | Escherichia coli DEC4C | 315.4 | 6.1E-91 |  |
| 1664 | ECDEC5A_5150 | EHV18515 | fimbrial subunit type 1 | Escherichia coli DEC5A | 315.4 | 6.1E-91 |  |
| 1665 | ECH7EC4486_2032 | EDU82689 | type-1 fimbrial protein homolog | Escherichia coli O157:H7 str. EC4486 | 315.4 | 6.1E-91 |  |
| 1666 | ECH74115_5820 | ACI34852 | type-1 fimbrial protein homolog | Escherichia coli O157:H7 str. EC4115 | 315.4 | 6.1E-91 | Yes |
| 1667 | ECPA39_5717 | EIO24319 | type-1 fimbrial protein, A chain | Escherichia coli PA39 | 315.4 | 6.1E-91 |  |
| 1668 | BY53_05115 | EZB18155 | type-1 fimbrial protein subunit A | Escherichia coli O157:H7 str. F7384 | 315.4 | 6.1E-91 |  |
| 1669 | BY90_06615 | EZD00255 | type-1 fimbrial protein subunit A | Escherichia coli O157:H7 str. K5609 | 315.4 | 6.1E-91 |  |
| 1670 | WQ88_01530 | KLH11532 | type-1 fimbrial protein subunit A | Escherichia coli (GCA_001012395) | 315.4 | 6.3E-91 | Yes |
| 1671 | AB64_5240 | KDY83843 | type-1 fimbrial protein, A chain | Escherichia coli 2-427-07_S1_C3 | 314.7 | 1E-90 |  |
| 1672 | AC48_4791 | KDY25564 | type-1 fimbrial protein, A chain | Escherichia coli 2-427-07_S3_C3 | 314.7 | 1E-90 |  |
| 1673 | ECoA_01154 | EGD70505 | type 1 fimbriae major subunit FimA | Escherichia coli O157:H7 str. 1044 | 313.8 | 1.9E-90 |  |
| 1674 | BAB38696 | BAB38696 | major type 1 subunit fimbrin | Escherichia coli O157:H7 str. Sakai | 313.8 | 1.9E-90 | Yes |
| 1675 | G905_04654 | EQW45347 | type-1 fimbrial protein, A chain | Escherichia coli UMEA 3087-1 | 313.8 | 2E-90 | Yes |
| 1676 | ECO5905_15018 | EFX28090 | Major type 1 subunit fimbrin | Escherichia coli O55:H7 str. USDA 5905 | 313.7 | 2.1E-90 | Yes |
| 1677 | EC5905_0087 | EKI02747 | type-1 fimbrial protein, A chain | Escherichia coli 5905 | 313.7 | 2.1E-90 |  |
| 1678 | BY41_13775 | EYV93461 | type-1 fimbrial protein subunit A | Escherichia coli O86:H34 str. 99-3124 | 313.5 | 2.4E-90 |  |
| 1679 | ERS085358_04326 | CTT02039 | fimbrial protein | Escherichia coli (GCA_001283825) | 313.5 | 2.4E-90 | Yes |
| 1680 | G824_04712 | EQS91268 | type-1 fimbrial protein, A chain | Escherichia coli HVH 169 (4-1075578) | 313.5 | 2.4E-90 |  |
| 1681 | A13M_00158 | ELC85867 | type-1 fimbrial protein, A chain | Escherichia coli KTE188 | 313.5 | 2.4E-90 |  |
| 1682 | G715_04533 | EQO48517 | type-1 fimbrial protein, A chain | Escherichia coli HVH 40 (4-1219782) | 313.5 | 2.4E-90 |  |
| 1683 | G957_04747 | EQY71507 | type-1 fimbrial protein, A chain | Escherichia coli UMEA 3268-1 | 313.5 | 2.4E-90 |  |
| 1684 | A1SS_00313 | ELE36058 | type-1 fimbrial protein, A chain | Escherichia coli KTE60 | 313.5 | 2.4E-90 |  |
| 1685 | A1U7_00763 | ELE44674 | type-1 fimbrial protein, A chain | Escherichia coli KTE67 | 313.5 | 2.4E-90 |  |
| 1686 | G703_04520 | EQN89179 | type-1 fimbrial protein, A chain | Escherichia coli HVH 27 (4-7449267) | 313.5 | 2.4E-90 |  |
| 1687 | G992_04404 | EQZ85476 | type-1 fimbrial protein, A chain | Escherichia coli UMEA 3705-1 | 313.5 | 2.4E-90 |  |
| 1688 | G699_03929 | ETF18705 | type-1 fimbrial protein, A chain | Escherichia coli HVH 23 (4-6066488) | 313.5 | 2.4E-90 |  |
| 1689 | G687_04685 | EQN26430 | type-1 fimbrial protein, A chain | Escherichia coli HVH 7 (4-7315031) | 313.5 | 2.4E-90 |  |
| 1690 | G754_04620 | EQQ19309 | type-1 fimbrial protein, A chain | Escherichia coli HVH 92 (4-5930790) | 313.5 | 2.4E-90 |  |
| 1691 | HMPREF9549_02310 | EFJ56279 | fimbrial protein | Escherichia coli MS 185-1 | 313.5 | 2.4E-90 |  |
| 1692 | G924_04719 | EQX14141 | type-1 fimbrial protein, A chain | Escherichia coli UMEA 3161-1 | 313.5 | 2.4E-90 |  |
| 1693 | A1S1_04325 | ELF92163 | fimbrial protein FimA | Escherichia coli KTE46 | 313.5 | 2.4E-90 |  |
| 1694 | G714_04548 | EQO38159 | type-1 fimbrial protein, A chain | Escherichia coli HVH 39 (4-2679949) | 313.5 | 2.4E-90 |  |
| 1695 | G738_04684 | EQP44601 | type-1 fimbrial protein, A chain | Escherichia coli HVH 74 (4-1034782) | 313.5 | 2.4E-90 |  |
| 1696 | HMPREF1604_02610 | ESD40676 | type-1 fimbrial protein, A chain | Escherichia coli 908519 | 313.5 | 2.4E-90 |  |
| 1697 | G956_04750 | EQY63442 | type-1 fimbrial protein, A chain | Escherichia coli UMEA 3264-1 | 313.5 | 2.4E-90 |  |
| 1698 | G886_04552 | EQV52125 | type-1 fimbrial protein, A chain | Escherichia coli KOEGE 44 (106a) | 313.5 | 2.4E-90 | Yes |
| 1699 | G751_04748 | EQP98054 | type-1 fimbrial protein, A chain | Escherichia coli HVH 89 (4-5885604) | 313.5 | 2.4E-90 |  |
| 1700 | HMPREF9532_05234 | EGB74375 | fimbrial protein | Escherichia coli MS 57-2 | 313.5 | 2.4E-90 |  |
| 1701 | G996_04796 | ERA02703 | type-1 fimbrial protein, A chain | Escherichia coli UMEA 3821-1 | 313.5 | 2.4E-90 |  |
| 1702 | G790_04598 | EQR60827 | type-1 fimbrial protein, A chain | Escherichia coli HVH 132 (4-6876862) | 313.5 | 2.4E-90 |  |
| 1703 | HMPREF1603_02694 | ESD37382 | type-1 fimbrial protein, A chain | Escherichia coli 907892 | 313.5 | 2.4E-90 |  |
| 1704 | EH65_22655 | KEP01856 | type-1 fimbrial protein subunit A | Escherichia coli (GCA_001285825) | 313.5 | 2.4E-90 |  |
| 1705 | G949_04821 | EQY35939 | type-1 fimbrial protein, A chain | Escherichia coli UMEA 3222-1 | 313.5 | 2.4E-90 |  |
| 1706 | G682_04734 | EQM98466 | type-1 fimbrial protein, A chain | Escherichia coli HVH 2 (4-6943160) | 313.5 | 2.4E-90 |  |
| 1707 | G955_04661 | EQY63787 | type-1 fimbrial protein, A chain | Escherichia coli UMEA 3257-1 | 313.5 | 2.4E-90 |  |
| 1708 | G770_04968 | EQQ62233 | type-1 fimbrial protein, A chain | Escherichia coli HVH 109 (4-6977162) | 313.5 | 2.4E-90 |  |
| 1709 | G819_04946 | EQS60395 | type-1 fimbrial protein, A chain | Escherichia coli HVH 161 (4-3119890) | 313.5 | 2.4E-90 |  |
| 1710 | OQA_21598 | EIA33919 | major type 1 subunit fimbrin (pilin) | Escherichia coli SCI-07 | 313.5 | 2.4E-90 |  |
| 1711 | G764_04660 | EQQ45239 | type-1 fimbrial protein, A chain | Escherichia coli HVH 103 (4-5904188) | 313.5 | 2.4E-90 |  |
| 1712 | H004_04667 | ERA41263 | type-1 fimbrial protein, A chain | Escherichia coli UMEA 4207-1 | 313.5 | 2.4E-90 |  |
| 1713 | A15S_02507 | ELH63261 | fimbrial protein FimA | Escherichia coli KTE209 | 313.5 | 2.4E-90 |  |
| 1714 | G911_04802 | EQW72692 | type-1 fimbrial protein, A chain | Escherichia coli UMEA 3121-1 | 313.5 | 2.4E-90 | Yes |
| 1715 | G756_04700 | EQQ20719 | type-1 fimbrial protein, A chain | Escherichia coli HVH 95 (4-6074464) | 313.5 | 2.4E-90 |  |
| 1716 | AC54_0001 | KEN35978 | type-1 fimbrial protein, A chain | Escherichia coli 8-415-05_S3_C3 | 313.5 | 2.4E-90 |  |
| 1717 | G748_04783 | ESP14110 | type-1 fimbrial protein, A chain | Escherichia coli HVH 86 (4-7026218) | 313.5 | 2.4E-90 |  |
| 1718 | WKA_04528 | ELI96092 | fimbrial protein FimA | Escherichia coli KTE153 | 313.5 | 2.4E-90 |  |
| 1719 | A1SE_00350 | ELE08293 | type-1 fimbrial protein, A chain | Escherichia coli KTE53 | 313.5 | 2.4E-90 |  |
| 1720 | AC79_4755 | KEJ05049 | type-1 fimbrial protein, A chain | Escherichia coli 8-415-05_S4_C1 | 313.5 | 2.4E-90 |  |
| 1721 | AB96_0001 | KEN45777 | type-1 fimbrial protein, A chain | Escherichia coli 8-415-05_S3_C1 | 313.5 | 2.4E-90 |  |
| 1722 | G885_04648 | EQV47259 | type-1 fimbrial protein, A chain | Escherichia coli KOEGE 43 (105a) | 313.5 | 2.4E-90 |  |
| 1723 | G932_04707 | EQX51227 | type-1 fimbrial protein, A chain | Escherichia coli UMEA 3178-1 | 313.5 | 2.4E-90 |  |
| 1724 | G691_04683 | EQN40433 | type-1 fimbrial protein, A chain | Escherichia coli HVH 13 (4-7634056) | 313.5 | 2.4E-90 |  |
| 1725 | G864_04658 | EQU66581 | type-1 fimbrial protein, A chain | Escherichia coli HVH 212 (3-9305343) | 313.5 | 2.4E-90 |  |
| 1726 | A17Y_00156 | ELD65673 | type-1 fimbrial protein, A chain | Escherichia coli KTE230 | 313.5 | 2.4E-90 |  |
| 1727 | G889_04778 | EQV61612 | type-1 fimbrial protein, A chain | Escherichia coli KOEGE 61 (174a) | 313.5 | 2.4E-90 |  |
| 1728 | G993_04633 | EQZ84775 | type-1 fimbrial protein, A chain | Escherichia coli UMEA 3707-1 | 313.5 | 2.4E-90 |  |
| 1729 | G827_04750 | EQT05648 | type-1 fimbrial protein, A chain | Escherichia coli HVH 172 (4-3248542) | 313.5 | 2.4E-90 |  |
| 1730 | G707_04582 | EQO10952 | type-1 fimbrial protein, A chain | Escherichia coli HVH 31 (4-2602156) | 313.5 | 2.4E-90 |  |
| 1731 | G930_04734 | EQX39401 | type-1 fimbrial protein, A chain | Escherichia coli UMEA 3175-1 | 313.5 | 2.4E-90 |  |
| 1732 | G815_04570 | ERA65092 | type-1 fimbrial protein, A chain | Escherichia coli HVH 157 (4-3406229) | 313.5 | 2.4E-90 |  |
| 1733 | G987_04599 | EQZ63550 | type-1 fimbrial protein, A chain | Escherichia coli UMEA 3687-1 | 313.5 | 2.4E-90 |  |
| 1734 | G802_04863 | EQS12121 | type-1 fimbrial protein, A chain | Escherichia coli HVH 144 (4-4451937) | 313.5 | 2.4E-90 |  |
| 1735 | G898_04596 | EQW12211 | type-1 fimbrial protein, A chain | Escherichia coli UMEA 3014-1 | 313.5 | 2.4E-90 |  |
| 1736 | G850_04498 | EQU05500 | type-1 fimbrial protein, A chain | Escherichia coli HVH 198 (4-3206106) | 313.5 | 2.4E-90 |  |
| 1737 | HMPREF9544_02313 | EFU52584 | fimbrial protein | Escherichia coli MS 153-1 | 313.5 | 2.4E-90 |  |
| 1738 | G856_04400 | EQU34067 | type-1 fimbrial protein, A chain | Escherichia coli HVH 204 (4-3112802) | 313.5 | 2.4E-90 |  |
| 1739 | G876_04712 | EQV20938 | type-1 fimbrial protein, A chain | Escherichia coli HVH 227 (4-2277670) | 313.5 | 2.4E-90 |  |
| 1740 | AB22_1081 | KEL52050 | type-1 fimbrial protein, A chain | Escherichia coli 6-175-07_S1_C1 | 313.5 | 2.4E-90 |  |
| 1741 | G989_04757 | EQZ71472 | type-1 fimbrial protein, A chain | Escherichia coli UMEA 3694-1 | 313.5 | 2.4E-90 |  |
| 1742 | HMPREF9531_01590 | EFJ93315 | fimbrial protein | Escherichia coli MS 45-1 | 313.5 | 2.4E-90 |  |
| 1743 | G729_04807 | EQP06012 | type-1 fimbrial protein, A chain | Escherichia coli HVH 58 (4-2839709) | 313.5 | 2.4E-90 |  |
| 1744 | G731_04477 | EQP15090 | type-1 fimbrial protein, A chain | Escherichia coli HVH 61 (4-2736020) | 313.5 | 2.4E-90 |  |
| 1745 | G817_04750 | ERA71835 | type-1 fimbrial protein, A chain | Escherichia coli HVH 159 (4-5818141) | 313.5 | 2.4E-90 |  |
| 1746 | G818_04692 | ERA80031 | type-1 fimbrial protein, A chain | Escherichia coli HVH 160 (4-5695937) | 313.5 | 2.4E-90 |  |
| 1747 | G982_04756 | ERF50441 | type-1 fimbrial protein, A chain | Escherichia coli UMEA 3652-1 | 313.5 | 2.4E-90 |  |
| 1748 | G870_04559 | EQU95727 | type-1 fimbrial protein, A chain | Escherichia coli HVH 218 (4-4500903) | 313.5 | 2.4E-90 |  |
| 1749 | G868_04576 | EQU88617 | type-1 fimbrial protein, A chain | Escherichia coli HVH 216 (4-3042952) | 313.5 | 2.4E-90 |  |
| 1750 | G782_04536 | EQR23650 | type-1 fimbrial protein, A chain | Escherichia coli HVH 120 (4-6978681) | 313.5 | 2.4E-90 |  |
| 1751 | G871_04501 | EQV03583 | type-1 fimbrial protein, A chain | Escherichia coli HVH 220 (4-5876842) | 313.5 | 2.4E-90 |  |
| 1752 | WI3_00001 | ELJ99862 | fimbrial protein FimA | Escherichia coli KTE99 | 313.5 | 2.4E-90 |  |
| 1753 | G712_04770 | EQO31692 | type-1 fimbrial protein, A chain | Escherichia coli HVH 37 (4-2773848) | 313.5 | 2.4E-90 |  |
| 1754 | AD07_0001 | KEJ16734 | type-1 fimbrial protein, A chain | Escherichia coli 8-415-05_S4_C2 | 313.5 | 2.4E-90 |  |
| 1755 | AC24_4831 | KEN68257 | type-1 fimbrial protein, A chain | Escherichia coli 8-415-05_S3_C2 | 313.5 | 2.4E-90 |  |
| 1756 | G775_04552 | EQQ82675 | type-1 fimbrial protein, A chain | Escherichia coli HVH 114 (4-7037740) | 313.5 | 2.4E-90 |  |
| 1757 | G971_04691 | ESK23905 | type-1 fimbrial protein, A chain | Escherichia coli UMEA 3342-1 | 313.5 | 2.4E-90 |  |
| 1758 | G922_04593 | EQX05409 | type-1 fimbrial protein, A chain | Escherichia coli UMEA 3159-1 | 313.5 | 2.4E-90 |  |
| 1759 | G757_04765 | EQQ30955 | type-1 fimbrial protein, A chain | Escherichia coli HVH 96 (4-5934869) | 313.5 | 2.4E-90 |  |
| 1760 | G859_04709 | EQU51485 | type-1 fimbrial protein, A chain | Escherichia coli HVH 207 (4-3113221) | 313.5 | 2.4E-90 |  |
| 1761 | G745_02963 | ETF19735 | type-1 fimbrial protein, A chain | Escherichia coli HVH 83 (4-2051087) | 313.5 | 2.4E-90 |  |
| 1762 | WIY_00004 | ELI80465 | fimbrial protein FimA | Escherichia coli KTE137 | 313.5 | 2.4E-90 |  |
| 1763 | A17A_00740 | ELH90462 | fimbrial protein FimA | Escherichia coli KTE218 | 313.5 | 2.4E-90 |  |
| 1764 | G910_04286 | EQW69322 | type-1 fimbrial protein, A chain | Escherichia coli UMEA 3117-1 | 313.5 | 2.4E-90 |  |
| 1765 | AD36_0001 | KEJ35767 | type-1 fimbrial protein, A chain | Escherichia coli 8-415-05_S4_C3 | 313.5 | 2.4E-90 |  |
| 1766 | G779_04911 | EQR10060 | type-1 fimbrial protein, A chain | Escherichia coli HVH 117 (4-6857191) | 313.5 | 2.4E-90 |  |
| 1767 | WGK_00467 | ELF70106 | fimbrial protein FimA | Escherichia coli KTE45 | 313.5 | 2.4E-90 |  |
| 1768 | G724_04658 | EQO85493 | type-1 fimbrial protein, A chain | Escherichia coli HVH 51 (4-2172526) | 313.5 | 2.4E-90 |  |
| 1769 | G985_04555 | EQZ59252 | type-1 fimbrial protein, A chain | Escherichia coli UMEA 3671-1 | 313.4 | 2.6E-90 | Yes |
| 1770 | EC991762_0030 | ELW27722 | type-1 fimbrial protein, A chain | Escherichia coli 99.1762 | 313.2 | 3E-90 | Yes |
| 1771 | PU77_21860 | KHG71626 | type-1 fimbrial protein subunit A | Escherichia coli | 313 | 3.4E-90 | Yes |
| 1772 | c5393 | AAN83815 | Type-1 fimbrial protein, A chain precursor | Escherichia coli CFT073 | 313 | 3.4E-90 | Yes |
| 1773 | i02_4911 | AER87426 | Type-1 fimbrial protein, A chain precursor | Escherichia coli str. 'clone D i2' | 313 | 3.4E-90 |  |
| 1774 | i14_4911 | AER92345 | Type-1 fimbrial protein, A chain precursor | Escherichia coli str. 'clone D i14' | 313 | 3.4E-90 |  |
| 1775 | BY81_11685 | EZC56794 | type-1 fimbrial protein subunit A | Escherichia coli O121:H19 str. K5269 | 312.7 | 4.2E-90 |  |
| 1776 | ECP03052602_4708 | ENE03243 | type-1 fimbrial protein, A chain | Escherichia coli P0305260.2 | 312.7 | 4.2E-90 |  |
| 1777 | BX32_22695 | EYV80095 | type-1 fimbrial protein subunit A | Escherichia coli O121:H19 str. 2009EL1412 | 312.7 | 4.2E-90 |  |
| 1778 | BX94_04790 | EYY14695 | type-1 fimbrial protein subunit A | Escherichia coli O121:H19 str. 2011C-3216 | 312.7 | 4.2E-90 |  |
| 1779 | ECP03052601_4490 | EMZ89738 | type-1 fimbrial protein, A chain | Escherichia coli P0305260.1 | 312.7 | 4.2E-90 |  |
| 1780 | BX72_10275 | EYY89865 | type-1 fimbrial protein subunit A | Escherichia coli O121:H19 str. 2010C-4732 | 312.7 | 4.2E-90 |  |
| 1781 | ECP02994389_4607 | ENC26268 | type-1 fimbrial protein, A chain | Escherichia coli P0299438.9 | 312.7 | 4.2E-90 |  |
| 1782 | Q460_23935 | ETI71935 | type-1 fimbrial protein subunit A | Escherichia coli ATCC BAA-2219 | 312.7 | 4.2E-90 |  |
| 1783 | ECP030526011_4680 | ENF78614 | type-1 fimbrial protein, A chain | Escherichia coli P0305260.11 | 312.7 | 4.2E-90 |  |
| 1784 | ECP03052604_4695 | ENF98081 | type-1 fimbrial protein, A chain | Escherichia coli P0305260.4 | 312.7 | 4.2E-90 |  |
| 1785 | BW69_19265 | EZA33086 | type-1 fimbrial protein subunit A | Escherichia coli O103:H11 str. 04-3023 | 312.7 | 4.2E-90 |  |
| 1786 | ECP030526013_4819 | ENF83226 | type-1 fimbrial protein, A chain | Escherichia coli P0305260.13 | 312.7 | 4.2E-90 |  |
| 1787 | BX79_06255 | EYY63251 | type-1 fimbrial protein subunit A | Escherichia coli O121:H19 str. 2010C-4824 | 312.7 | 4.2E-90 |  |
| 1788 | BY00_02680 | EYY03497 | type-1 fimbrial protein subunit A | Escherichia coli O121:H19 str. 2011C-3500 | 312.7 | 4.2E-90 |  |
| 1789 | HMPREF9550_01113 | EFK26735 | fimbrial protein | Escherichia coli MS 187-1 | 312.7 | 4.2E-90 |  |
| 1790 | ECP03052607_4890 | ENG09735 | type-1 fimbrial protein, A chain | Escherichia coli P0305260.7 | 312.7 | 4.2E-90 |  |
| 1791 | ECP030526012_4645 | ENF80718 | type-1 fimbrial protein, A chain | Escherichia coli P0305260.12 | 312.7 | 4.2E-90 |  |
| 1792 | ECP030526015_4643 | ENF93182 | type-1 fimbrial protein, A chain | Escherichia coli P0305260.15 | 312.7 | 4.2E-90 |  |
| 1793 | BX84_16390 | EYY37874 | type-1 fimbrial protein subunit A | Escherichia coli O121:H19 str. 2010C-4989 | 312.7 | 4.2E-90 |  |
| 1794 | AC42_4714 | KDV77055 | type-1 fimbrial protein, A chain | Escherichia coli 2-052-05_S3_C3 | 312.7 | 4.2E-90 |  |
| 1795 | ECP03052605_4606 | ENG08608 | type-1 fimbrial protein, A chain | Escherichia coli P0305260.5 | 312.7 | 4.2E-90 |  |
| 1796 | EC5411_15332 | EIL63549 | FimA | Escherichia coli 541-1 | 312.7 | 4.2E-90 |  |
| 1797 | AC14_4974 | KEN80910 | type-1 fimbrial protein, A chain | Escherichia coli 2-052-05_S3_C2 | 312.7 | 4.2E-90 |  |
| 1798 | ECP03052608_4690 | ENG20636 | type-1 fimbrial protein, A chain | Escherichia coli P0305260.8 | 312.7 | 4.2E-90 | Yes |
| 1799 | BW93_13965 | EYZ46482 | type-1 fimbrial protein subunit A | Escherichia coli O121:H19 str. 06-3822 | 312.7 | 4.2E-90 |  |
| 1800 | A1UY_00574 | ELE75345 | type-1 fimbrial protein, A chain | Escherichia coli KTE81 | 312.7 | 4.2E-90 |  |
| 1801 | EC50959_5376 | EII09903 | type-1 fimbrial protein, A chain | Escherichia coli 5.0959 | 312.7 | 4.2E-90 | Yes |
| 1802 | BX52_15280 | EYV08536 | type-1 fimbrial protein subunit A | Escherichia coli O121:H19 str. 2010C-3609 | 312.7 | 4.2E-90 |  |
| 1803 | AC06_4053 | KDT63325 | type-1 fimbrial protein, A chain | Escherichia coli 3-373-03_S3_C1 | 312.7 | 4.2E-90 |  |
| 1804 | BX53_00475 | EZE99951 | type-1 fimbrial protein subunit A | Escherichia coli O121:H19 str. 2010C-3794 | 312.7 | 4.2E-90 |  |
| 1805 | ETEC_4626 | CBJ04135 | fimbrial protein | Escherichia coli ETEC H10407 | 312.7 | 4.2E-90 | Yes |
| 1806 | BX87_04770 | EYY33132 | type-1 fimbrial protein subunit A | Escherichia coli O121:H19 str. 2010EL1058 | 312.7 | 4.2E-90 |  |
| 1807 | AB09_4775 | KEN30532 | type-1 fimbrial protein, A chain | Escherichia coli 8-415-05_S1_C1 | 312.7 | 4.2E-90 |  |
| 1808 | BX27_21945 | EZE76677 | type-1 fimbrial protein subunit A | Escherichia coli O121:H19 str. 2009C-4750 | 312.7 | 4.2E-90 |  |
| 1809 | ECP030526010_4661 | ENF69300 | type-1 fimbrial protein, A chain | Escherichia coli P0305260.10 | 312.7 | 4.2E-90 |  |
| 1810 | BW83_02280 | EYZ86845 | type-1 fimbrial protein subunit A | Escherichia coli O121:H19 str. 06-3003 | 312.7 | 4.2E-90 |  |
| 1811 | BY80_11920 | EZC53186 | type-1 fimbrial protein subunit A | Escherichia coli O121:H19 str. K5198 | 312.7 | 4.2E-90 |  |
| 1812 | BX54_06915 | EYV01812 | type-1 fimbrial protein subunit A | Escherichia coli O121:H19 str. 2010C-3840 | 312.7 | 4.2E-90 |  |
| 1813 | ECP03052606_4642 | ENG10363 | type-1 fimbrial protein, A chain | Escherichia coli P0305260.6 | 312.7 | 4.2E-90 |  |
| 1814 | AB84_4592 | KDT14092 | type-1 fimbrial protein, A chain | Escherichia coli 2-052-05_S3_C1 | 312.7 | 4.2E-90 |  |
| 1815 | BX31_00795 | EZE90224 | type-1 fimbrial protein subunit A | Escherichia coli O121:H19 str. 2009EL1302 | 312.7 | 4.2E-90 |  |
| 1816 | BX25_24910 | EYV82737 | type-1 fimbrial protein subunit A | Escherichia coli O121:H19 str. 2009C-4659 | 312.7 | 4.2E-90 |  |
| 1817 | BY47_07740 | EZA94057 | type-1 fimbrial protein subunit A | Escherichia coli O121:H19 str. F6714 | 312.7 | 4.2E-90 |  |
| 1818 | BW77_17785 | EZA12184 | type-1 fimbrial protein subunit A | Escherichia coli O121:H19 str. 03-3227 | 312.7 | 4.2E-90 |  |
| 1819 | BX19_15140 | EZE42779 | type-1 fimbrial protein subunit A | Escherichia coli O121:H19 str. 2009C-4050 | 312.7 | 4.2E-90 |  |
| 1820 | ECP03052603_4697 | ENF97393 | type-1 fimbrial protein, A chain | Escherichia coli P0305260.3 | 312.7 | 4.2E-90 |  |
| 1821 | AB37_4810 | KEO19332 | type-1 fimbrial protein, A chain | Escherichia coli 8-415-05_S1_C2 | 312.7 | 4.2E-90 |  |
| 1822 | BX91_04195 | EYY27553 | type-1 fimbrial protein subunit A | Escherichia coli O121:H19 str. 2011C-3072 | 312.7 | 4.2E-90 |  |
| 1823 | BX82_16180 | EYY51275 | type-1 fimbrial protein subunit A | Escherichia coli O121:H19 str. 2010C-4966 | 312.7 | 4.2E-90 |  |
| 1824 | BY02_03355 | EYY00191 | type-1 fimbrial protein subunit A | Escherichia coli O121:H19 str. 2011C-3537 | 312.7 | 4.2E-90 |  |
| 1825 | UC21_09135 | KJY11861 | type-1 fimbrial protein subunit A | Escherichia coli O78:H11 (strain H10407 / ETEC) | 312.7 | 4.2E-90 |  |
| 1826 | ECP02994382_4566 | ENA00244 | type-1 fimbrial protein, A chain | Escherichia coli P0299438.2 | 312.7 | 4.2E-90 |  |
| 1827 | BX62_24915 | EYU80177 | type-1 fimbrial protein subunit A | Escherichia coli O121:H19 str. 2010C-4254 | 312.7 | 4.2E-90 |  |
| 1828 | ESOG_00112 | EHO03074 | type-1 fimbrial protein | Escherichia coli E101 | 312.7 | 4.3E-90 | Yes |
| 1829 | ERS139237_03412 | CUA13397 | fimbrial protein | Escherichia coli (GCA_001286485) | 311 | 1.4E-89 | Yes |
| 1830 | ECP03052609_4670 | ENG27748 | type-1 fimbrial protein, A chain | Escherichia coli P0305260.9 | 311 | 1.4E-89 | Yes |
| 1831 | ECARS42123_4864 | EKI21463 | type-1 fimbrial protein, A chain | Escherichia coli ARS4.2123 | 310.8 | 1.6E-89 |  |
| 1832 | EC3003_4853 | EII88287 | type-1 fimbrial protein, A chain | Escherichia coli 3003 | 310.8 | 1.6E-89 |  |
| 1833 | ECRN5871_1621 | EFZ75114 | type-1 fimbrial protein, A chain | Escherichia coli RN587/1 | 310.8 | 1.6E-89 | Yes |
| 1834 | AD35_4417 | KEN49900 | type-1 fimbrial protein, A chain | Escherichia coli 7-233-03_S4_C3 | 310.8 | 1.6E-89 |  |
| 1835 | AC78_4465 | KEN37367 | type-1 fimbrial protein, A chain | Escherichia coli 7-233-03_S4_C1 | 310.8 | 1.6E-89 | Yes |
| 1836 | AB85_5090 | KDV96068 | type-1 fimbrial protein, A chain | Escherichia coli 2-156-04_S3_C1 | 310.7 | 1.7E-89 |  |
| 1837 | WG7_00030 | EOV16050 | fimbrial protein FimA | Escherichia coli KTE38 | 310.7 | 1.8E-89 |  |
| 1838 | WG5_00020 | EOU99240 | fimbrial protein FimA | Escherichia coli KTE37 | 310.7 | 1.8E-89 |  |
| 1839 | G740_04501 | EQP64004 | type-1 fimbrial protein, A chain | Escherichia coli HVH 77 (4-2605759) | 310.5 | 1.9E-89 |  |
| 1840 | WIM_00016 | ELI48262 | fimbrial protein FimA | Escherichia coli KTE124 | 310.5 | 1.9E-89 | Yes |
| 1841 | A15C_00476 | ELD03186 | type-1 fimbrial protein, A chain | Escherichia coli KTE201 | 310.5 | 1.9E-89 |  |
| 1842 | AB25_5433 | EZK20322 | type-1 fimbrial protein, A chain | Escherichia coli 2-005-03_S1_C2 | 310.5 | 1.9E-89 |  |
| 1843 | AB53_5353 | EZK07801 | type-1 fimbrial protein, A chain | Escherichia coli 2-005-03_S1_C3 | 310.5 | 1.9E-89 |  |
| 1844 | AC22_5671 | KEL68157 | type-1 fimbrial protein, A chain | Escherichia coli 5-366-08_S3_C2 | 310.5 | 1.9E-89 |  |
| 1845 | AC46_5695 | KDX73661 | type-1 fimbrial protein, A chain | Escherichia coli 2-222-05_S3_C3 | 310 | 2.9E-89 |  |
| 1846 | AB69_4699 | EYE16472 | type-1 fimbrial protein, A chain | Escherichia coli 1-110-08_S1_C3 | 310 | 2.9E-89 |  |
| 1847 | EH64_09805 | KEP03983 | type-1 fimbrial protein subunit A | Escherichia coli str. UCD_JA23 | 309.7 | 3.5E-89 | Yes |
| 1848 | AL530_23805 | KTK77809 | type-1 fimbrial protein subunit A | Escherichia fergusonii | 309.2 | 5.2E-89 | Yes |
| 1849 | AC85_3604 | KEJ56460 | type-1 fimbrial protein, A chain | Escherichia coli 3-020-07_S4_C1 | 308.4 | 8.5E-89 |  |
| 1850 | AD30_5556 | KDY09650 | type-1 fimbrial protein, A chain | Escherichia coli 2-316-03_S4_C3 | 308.4 | 8.5E-89 |  |
| 1851 | AC17_5212 | KDW91287 | type-1 fimbrial protein, A chain | Escherichia coli 2-210-07_S3_C2 | 307.8 | 1.4E-88 |  |
| 1852 | AB71_5158 | EZK01045 | type-1 fimbrial protein, A chain | Escherichia coli 1-182-04_S1_C3 | 307.8 | 1.4E-88 |  |
| 1853 | EC2866450_4929 | EMV66490 | type-1 fimbrial protein, A chain | Escherichia coli 2866450 | 304 | 2E-87 |  |
| 1854 | AC24_5045 | KEN80912 | type-1 fimbrial protein, A chain | Escherichia coli 8-415-05_S3_C2 | 303.2 | 3.6E-87 |  |
| 1855 | G947_04726 | EQY26003 | type-1 fimbrial protein, A chain | Escherichia coli UMEA 3220-1 | 302.9 | 4.3E-87 |  |
| 1856 | EC2846750_4874 | EMZ59777 | type-1 fimbrial protein, A chain | Escherichia coli 2846750 | 302.5 | 5.6E-87 |  |
| 1857 | AB20_4734 | KDT77251 | type-1 fimbrial protein, A chain | Escherichia coli 3-475-03_S1_C1 | 302.5 | 5.6E-87 |  |
| 1858 | L343_4510 | ESS88469 | type 1 fimbriae major subunit FimA | Escherichia coli CE549 | 302.5 | 5.6E-87 |  |
| 1859 | EC2872000_5039 | EMV50865 | type-1 fimbrial protein, A chain | Escherichia coli 2872000 | 302.5 | 5.6E-87 |  |
| 1860 | BY42_02810 | EYV85391 | type-1 fimbrial protein subunit A | Escherichia coli O6:H16 str. 99-3165 | 302.5 | 5.6E-87 |  |
| 1861 | EC40522_5693 | EIH80233 | type-1 fimbrial protein, A chain | Escherichia coli 4.0522 | 302.5 | 5.6E-87 |  |
| 1862 | ECP03019043_4912 | END87044 | type-1 fimbrial protein, A chain | Escherichia coli P0301904.3 | 302.5 | 5.6E-87 |  |
| 1863 | BY44_02195 | EZA68922 | type-1 fimbrial protein subunit A | Escherichia coli O6:H16 str. F5656C1 | 302.5 | 5.6E-87 |  |
| 1864 | JO86_22710 | KGT27533 | type-1 fimbrial protein subunit A | Escherichia coli | 302.5 | 5.6E-87 |  |
| 1865 | N444_14020 | ETS26501 | type-1 fimbrial protein subunit A | Escherichia coli O6:H16:CFA/II str. B2C | 302.5 | 5.6E-87 |  |
| 1866 | AB45_4356 | KDZ78668 | type-1 fimbrial protein, A chain | Escherichia coli 3-105-05_S1_C2 | 302.5 | 5.6E-87 |  |
| 1867 | EC2871950_5013 | EMV50636 | type-1 fimbrial protein, A chain | Escherichia coli 2871950 | 302.5 | 5.6E-87 |  |
| 1868 | AD36_4932 | KEJ24698 | type-1 fimbrial protein, A chain | Escherichia coli 8-415-05_S4_C3 | 300.5 | 2.4E-86 |  |
| 1869 | AB96_5247 | KEN34204 | type-1 fimbrial protein, A chain | Escherichia coli 8-415-05_S3_C1 | 300.5 | 2.4E-86 |  |
| 1870 | AC54_4862 | KEN30313 | type-1 fimbrial protein, A chain | Escherichia coli 8-415-05_S3_C3 | 300.5 | 2.4E-86 |  |
| 1871 | AB88_5070 | KEN84370 | type-1 fimbrial protein, A chain | Escherichia coli 2-222-05_S3_C1 | 299.8 | 4E-86 |  |
| 1872 | AD06_0001 | KEN16704 | type-1 fimbrial protein, A chain | Escherichia coli 7-233-03_S4_C2 | 299.8 | 4E-86 |  |
