# Supplementary material for: Functional role of the type 1 pilus rod structure in mediating host-pathogen interactions
Source: eLife. 2018 Jan 18;7:e31662. doi: 10.7554/eLife.31662 (PMC5798934; doi:10.7554/eLife.31662)
Supplement: Supplementary file 4. [file elife-31662-supp4.docx]

| Protein | Number | GeneID | ENA_ID | Description | Species | Bit Score | E-value |
| --- | --- | --- | --- | --- | --- | --- | --- |
| FimC | 1 | G817_04752 | ERA71837 | chaperone fimC | Escherichia coli HVH 159 (4-5818141) | 665.6 | 1.80E-196 |
| FimC | 2 | G740_04503 | EQP64006 | chaperone fimC | Escherichia coli HVH 77 (4-2605759) | 665.6 | 1.80E-196 |
| FimC | 3 | G775_04554 | EQQ82677 | chaperone fimC | Escherichia coli HVH 114 (4-7037740) | 665.6 | 1.80E-196 |
| FimC | 4 | WI3_00003 | ELJ99864 | fimbrial chaperone FimC | Escherichia coli KTE99 | 665.6 | 1.80E-196 |
| FimC | 5 | G688_04333 | EQN26610 | chaperone fimC | Escherichia coli HVH 9 (4-6942539) | 665.6 | 1.80E-196 |
| FimC | 6 | G802_04865 | EQS12123 | chaperone fimC | Escherichia coli HVH 144 (4-4451937) | 665.6 | 1.80E-196 |
| FimC | 7 | AD07_0003 | KEJ16736 | chaperone protein fimC | Escherichia coli 8-415-05_S4_C2 | 665.6 | 1.80E-196 |
| FimC | 8 | G870_04561 | EQU95729 | chaperone fimC | Escherichia coli HVH 218 (4-4500903) | 665.6 | 1.80E-196 |
| FimC | 9 | EcoM_02395 | EFW69887 | chaperone FimC | Escherichia coli WV_060327 | 665.6 | 1.80E-196 |
| FimC | 10 | G920_04331 | EQW97662 | chaperone fimC | Escherichia coli UMEA 3152-1 | 665.6 | 1.80E-196 |
| FimC | 11 | G956_04752 | EQY63444 | chaperone fimC | Escherichia coli UMEA 3264-1 | 665.6 | 1.80E-196 |
| FimC | 12 | G745_02965 | ETF19737 | chaperone fimC | Escherichia coli HVH 83 (4-2051087) | 665.6 | 1.80E-196 |
| FimC | 13 | G947_04728 | EQY26005 | chaperone fimC | Escherichia coli UMEA 3220-1 | 665.6 | 1.80E-196 |
| FimC | 14 | A1SS_00315 | ELE36060 | chaperone fimC | Escherichia coli KTE60 | 665.6 | 1.80E-196 |
| FimC | 15 | G932_04709 | EQX51229 | chaperone fimC | Escherichia coli UMEA 3178-1 | 665.6 | 1.80E-196 |
| FimC | 16 | NRG857_21775 | ADR29762 | type I fimbrial chaperone | Escherichia coli O83:H1 str. NRG 857C | 665.6 | 1.80E-196 |
| FimC | 17 | A1SE_00352 | ELE08295 | chaperone fimC | Escherichia coli KTE53 | 665.6 | 1.80E-196 |
| FimC | 18 | G856_04402 | EQU34069 | chaperone fimC | Escherichia coli HVH 204 (4-3112802) | 665.6 | 1.80E-196 |
| FimC | 19 | G982_04758 | ERF50443 | chaperone fimC | Escherichia coli UMEA 3652-1 | 665.6 | 1.80E-196 |
| FimC | 20 | G683_04701 | EQN00203 | chaperone fimC | Escherichia coli HVH 3 (4-7276001) | 665.6 | 1.80E-196 |
| FimC | 21 | G989_04759 | EQZ71474 | chaperone fimC | Escherichia coli UMEA 3694-1 | 665.6 | 1.80E-196 |
| FimC | 22 | A1S3_00305 | ELD93557 | chaperone fimC | Escherichia coli KTE47 | 665.6 | 1.80E-196 |
| FimC | 23 | G715_04535 | EQO48519 | chaperone fimC | Escherichia coli HVH 40 (4-1219782) | 665.6 | 1.80E-196 |
| FimC | 24 | UTI89_C5013 | ABE10416 | periplasmic chaperone protein FimC precursor | Escherichia coli UTI89 | 665.6 | 1.80E-196 |
| FimC | 25 | G756_04702 | EQQ20721 | chaperone fimC | Escherichia coli HVH 95 (4-6074464) | 665.6 | 1.80E-196 |
| FimC | 26 | HMPREF1603_02696 | ESD37384 | gram-negative pili assembly chaperone domain protein | Escherichia coli 907892 | 665.6 | 1.80E-196 |
| FimC | 27 | G703_04522 | EQN89181 | chaperone fimC | Escherichia coli HVH 27 (4-7449267) | 665.6 | 1.80E-196 |
| FimC | 28 | G717_04824 | EQO56929 | chaperone fimC | Escherichia coli HVH 42 (4-2100061) | 665.6 | 1.80E-196 |
| FimC | 29 | i02_4913 | AER87428 | chaperone protein fimC precursor | Escherichia coli str. 'clone D i2' | 665.6 | 1.80E-196 |
| FimC | 30 | HMPREF9531_01592 | EFJ93317 | gram-negative pili assembly chaperone domain protein | Escherichia coli MS 45-1 | 665.6 | 1.80E-196 |
| FimC | 31 | G770_04970 | EQQ62235 | chaperone fimC | Escherichia coli HVH 109 (4-6977162) | 665.6 | 1.80E-196 |
| FimC | 32 | ECAA86_00019 | EGH38262 | chaperone FimC | Escherichia coli AA86 | 665.6 | 1.80E-196 |
| FimC | 33 | G930_04736 | EQX39403 | chaperone fimC | Escherichia coli UMEA 3175-1 | 665.6 | 1.80E-196 |
| FimC | 34 | G820_04529 | EQS74805 | chaperone fimC | Escherichia coli HVH 162 (4-5627982) | 665.6 | 1.80E-196 |
| FimC | 35 | G699_03931 | ETF18707 | chaperone fimC | Escherichia coli HVH 23 (4-6066488) | 665.6 | 1.80E-196 |
| FimC | 36 | G729_04809 | EQP06014 | chaperone fimC | Escherichia coli HVH 58 (4-2839709) | 665.6 | 1.80E-196 |
| FimC | 37 | ECOPMV1_04762 | CDH68043 | Chaperone protein fimC precursor | Escherichia coli PMV-1 | 665.6 | 1.80E-196 |
| FimC | 38 | G996_04798 | ERA02705 | chaperone fimC | Escherichia coli UMEA 3821-1 | 665.6 | 1.80E-196 |
| FimC | 39 | G868_04578 | EQU88619 | chaperone fimC | Escherichia coli HVH 216 (4-3042952) | 665.6 | 1.80E-196 |
| FimC | 40 | G748_04785 | ESP14112 | chaperone fimC | Escherichia coli HVH 86 (4-7026218) | 665.6 | 1.80E-196 |
| FimC | 41 | AC24_4833 | KEN68259 | chaperone protein fimC | Escherichia coli 8-415-05_S3_C2 | 665.6 | 1.80E-196 |
| FimC | 42 | A1U7_00765 | ELE44676 | chaperone fimC | Escherichia coli KTE67 | 665.6 | 1.80E-196 |
| FimC | 43 | A17Y_00158 | ELD65675 | chaperone fimC | Escherichia coli KTE230 | 665.6 | 1.80E-196 |
| FimC | 44 | G898_04598 | EQW12213 | chaperone fimC | Escherichia coli UMEA 3014-1 | 665.6 | 1.80E-196 |
| FimC | 45 | T638_06380 | KKJ21618 | molecular chaperone FimC | Escherichia coli MRSN 10204 | 665.6 | 1.80E-196 |
| FimC | 46 | G731_04479 | EQP15092 | chaperone fimC | Escherichia coli HVH 61 (4-2736020) | 665.6 | 1.80E-196 |
| FimC | 47 | G707_04584 | EQO10954 | chaperone fimC | Escherichia coli HVH 31 (4-2602156) | 665.6 | 1.80E-196 |
| FimC | 48 | G987_04601 | EQZ63552 | chaperone fimC | Escherichia coli UMEA 3687-1 | 665.6 | 1.80E-196 |
| FimC | 49 | WEE_00336 | ELF77868 | fimbrial chaperone FimC | Escherichia coli KTE23 | 665.6 | 1.80E-196 |
| FimC | 50 | G992_04406 | EQZ85478 | chaperone fimC | Escherichia coli UMEA 3705-1 | 665.6 | 1.80E-196 |
| FimC | 51 | G815_04572 | ERA65094 | chaperone fimC | Escherichia coli HVH 157 (4-3406229) | 665.6 | 1.80E-196 |
| FimC | 52 | G738_04686 | EQP44603 | chaperone fimC | Escherichia coli HVH 74 (4-1034782) | 665.6 | 1.80E-196 |
| FimC | 53 | G782_04538 | EQR23652 | chaperone fimC | Escherichia coli HVH 120 (4-6978681) | 665.6 | 1.80E-196 |
| FimC | 54 | A13M_00160 | ELC85869 | chaperone fimC | Escherichia coli KTE188 | 665.6 | 1.80E-196 |
| FimC | 55 | A179_00606 | ELH78761 | fimbrial chaperone FimC | Escherichia coli KTE217 | 665.6 | 1.80E-196 |
| FimC | 56 | G871_04503 | EQV03585 | chaperone fimC | Escherichia coli HVH 220 (4-5876842) | 665.6 | 1.80E-196 |
| FimC | 57 | G885_04650 | EQV47261 | chaperone fimC | Escherichia coli KOEGE 43 (105a) | 665.6 | 1.80E-196 |
| FimC | 58 | UM146_22270 | ADN73778 | type I fimbrial chaperone | Escherichia coli UM146 | 665.6 | 1.80E-196 |
| FimC | 59 | WKA_04530 | ELI96094 | fimbrial chaperone FimC | Escherichia coli KTE153 | 665.6 | 1.80E-196 |
| FimC | 60 | G876_04714 | EQV20940 | chaperone fimC | Escherichia coli HVH 227 (4-2277670) | 665.6 | 1.80E-196 |
| FimC | 61 | HMPREF9549_02312 | EFJ56281 | gram-negative pili assembly chaperone domain protein | Escherichia coli MS 185-1 | 665.6 | 1.80E-196 |
| FimC | 62 | WE1_00513 | ELF60574 | fimbrial chaperone FimC | Escherichia coli KTE17 | 665.6 | 1.80E-196 |
| FimC | 63 | G691_04685 | EQN40435 | chaperone fimC | Escherichia coli HVH 13 (4-7634056) | 665.6 | 1.80E-196 |
| FimC | 64 | G864_04660 | EQU66583 | chaperone fimC | Escherichia coli HVH 212 (3-9305343) | 665.6 | 1.80E-196 |
| FimC | 65 | G754_04622 | EQQ19311 | chaperone fimC | Escherichia coli HVH 92 (4-5930790) | 665.6 | 1.80E-196 |
| FimC | 66 | H004_04669 | ERA41265 | chaperone fimC | Escherichia coli UMEA 4207-1 | 665.6 | 1.80E-196 |
| FimC | 67 | G993_04635 | EQZ84777 | chaperone fimC | Escherichia coli UMEA 3707-1 | 665.6 | 1.80E-196 |
| FimC | 68 | G955_04663 | EQY63789 | chaperone fimC | Escherichia coli UMEA 3257-1 | 665.6 | 1.80E-196 |
| FimC | 69 | A15C_00478 | ELD03188 | chaperone fimC | Escherichia coli KTE201 | 665.6 | 1.80E-196 |
| FimC | 70 | LF82_0660 | CAP78797 | chaperone protein fimC | Escherichia coli LF82 | 665.6 | 1.80E-196 |
| FimC | 71 | G911_04804 | EQW72694 | chaperone fimC | Escherichia coli UMEA 3121-1 | 665.6 | 1.80E-196 |
| FimC | 72 | EH65_22665 | KEP01858 | molecular chaperone FimC | Escherichia coli (strain UTI89 / UPEC) | 665.6 | 1.80E-196 |
| FimC | 73 | A15S_02509 | ELH63263 | fimbrial chaperone FimC | Escherichia coli KTE209 | 665.6 | 1.80E-196 |
| FimC | 74 | G751_04750 | EQP98056 | chaperone fimC | Escherichia coli HVH 89 (4-5885604) | 665.6 | 1.80E-196 |
| FimC | 75 | G924_04721 | EQX14143 | chaperone fimC | Escherichia coli UMEA 3161-1 | 665.6 | 1.80E-196 |
| FimC | 76 | G824_04714 | EQS91270 | chaperone fimC | Escherichia coli HVH 169 (4-1075578) | 665.6 | 1.80E-196 |
| FimC | 77 | G687_04687 | EQN26432 | chaperone fimC | Escherichia coli HVH 7 (4-7315031) | 665.6 | 1.80E-196 |
| FimC | 78 | AD36_0003 | KEJ35769 | chaperone protein fimC | Escherichia coli 8-415-05_S4_C3 | 665.6 | 1.80E-196 |
| FimC | 79 | ECIG_03247 | EGI12989 | chaperone protein FimC | Escherichia coli M605 | 665.6 | 1.80E-196 |
| FimC | 80 | G997_04672 | ERA14688 | chaperone fimC | Escherichia coli UMEA 3834-1 | 665.6 | 1.80E-196 |
| FimC | 81 | G949_04823 | EQY35941 | chaperone fimC | Escherichia coli UMEA 3222-1 | 665.6 | 1.80E-196 |
| FimC | 82 | G889_04780 | EQV61614 | chaperone fimC | Escherichia coli KOEGE 61 (174a) | 665.6 | 1.80E-196 |
| FimC | 83 | BY41_13785 | EYV93463 | molecular chaperone FimC | Escherichia coli O86:H34 str. 99-3124 | 665.6 | 1.80E-196 |
| FimC | 84 | A17A_00742 | ELH90464 | fimbrial chaperone FimC | Escherichia coli KTE218 | 665.6 | 1.80E-196 |
| FimC | 85 | SC80_07015 | KIE82461 | molecular chaperone FimC | Escherichia coli RS218 | 665.6 | 1.80E-196 |
| FimC | 86 | G682_04736 | EQM98468 | chaperone fimC | Escherichia coli HVH 2 (4-6943160) | 665.6 | 1.80E-196 |
| FimC | 87 | G975_03701 | ETF33543 | chaperone fimC | Escherichia coli UMEA 3489-1 | 665.6 | 1.80E-196 |
| FimC | 88 | G827_04752 | EQT05650 | chaperone fimC | Escherichia coli HVH 172 (4-3248542) | 665.6 | 1.80E-196 |
| FimC | 89 | WIM_00018 | ELI48264 | fimbrial chaperone FimC | Escherichia coli KTE124 | 665.6 | 1.80E-196 |
| FimC | 90 | G981_04507 | EQZ45609 | chaperone fimC | Escherichia coli UMEA 3632-1 | 665.6 | 1.80E-196 |
| FimC | 91 | ECOK1_4815 | ADE89910 | chaperone protein FimC | Escherichia coli IHE3034 | 665.6 | 1.80E-196 |
| FimC | 92 | G922_04595 | EQX05411 | chaperone fimC | Escherichia coli UMEA 3159-1 | 665.6 | 1.80E-196 |
| FimC | 93 | G819_04944 | EQS60393 | chaperone fimC | Escherichia coli HVH 161 (4-3119890) | 665.6 | 1.80E-196 |
| FimC | 94 | HMPREF9544_02311 | EFU52582 | gram-negative pili assembly chaperone domain protein | Escherichia coli MS 153-1 | 665.6 | 1.80E-196 |
| FimC | 95 | c5395 | AAN83817 | Chaperone protein fimC precursor | Escherichia coli CFT073 | 665.6 | 1.80E-196 |
| FimC | 96 | G757_04767 | EQQ30957 | chaperone fimC | Escherichia coli HVH 96 (4-5934869) | 665.6 | 1.80E-196 |
| FimC | 97 | G743_01793 | EQP82365 | chaperone fimC | Escherichia coli HVH 80 (4-2428830) | 665.6 | 1.80E-196 |
| FimC | 98 | WE3_00334 | ELF68611 | fimbrial chaperone FimC | Escherichia coli KTE18 | 665.6 | 1.80E-196 |
| FimC | 99 | G818_04694 | ERA80033 | chaperone fimC | Escherichia coli HVH 160 (4-5695937) | 665.6 | 1.80E-196 |
| FimC | 100 | G786_04684 | EQR43917 | chaperone fimC | Escherichia coli HVH 126 (4-6034225) | 665.6 | 1.80E-196 |
| FimC | 101 | G712_04772 | EQO31694 | chaperone fimC | Escherichia coli HVH 37 (4-2773848) | 665.6 | 1.80E-196 |
| FimC | 102 | i14_4913 | AER92347 | chaperone protein fimC precursor | Escherichia coli str. 'clone D i14' | 665.6 | 1.80E-196 |
| FimC | 103 | G779_04913 | EQR10062 | chaperone fimC | Escherichia coli HVH 117 (4-6857191) | 665.6 | 1.80E-196 |
| FimC | 104 | APECO1_2114 | ABJ03823 | type I fimbrial chaperone | Escherichia coli O1:K1 / APEC | 665.6 | 1.80E-196 |
| FimC | 105 | G905_04656 | EQW45349 | chaperone fimC | Escherichia coli UMEA 3087-1 | 665.6 | 1.80E-196 |
| FimC | 106 | AL530_23795 | KTK77807 | molecular chaperone FimC | Escherichia fergusonii | 665.6 | 1.80E-196 |
| FimC | 107 | G961_04748 | ERB28782 | chaperone fimC | Escherichia coli UMEA 3298-1 | 665.6 | 1.80E-196 |
| FimC | 108 | WKY_04685 | ELJ47444 | fimbrial chaperone FimC | Escherichia coli KTE180 | 665.6 | 1.80E-196 |
| FimC | 109 | G790_04600 | EQR60829 | chaperone fimC | Escherichia coli HVH 132 (4-6876862) | 665.6 | 1.80E-196 |
| FimC | 110 | A1YC_00306 | EOW41401 | fimbrial chaperone FimC | Escherichia coli KTE126 | 665.6 | 1.80E-196 |
| FimC | 111 | G886_04554 | EQV52127 | chaperone fimC | Escherichia coli KOEGE 44 (106a) | 665.6 | 1.80E-196 |
| FimC | 112 | AC54_0003 | KEN35980 | chaperone protein fimC | Escherichia coli 8-415-05_S3_C3 | 665.6 | 1.80E-196 |
| FimC | 113 | G971_04693 | ESK23907 | chaperone fimC | Escherichia coli UMEA 3342-1 | 665.6 | 1.80E-196 |
| FimC | 114 | G714_04550 | EQO38161 | chaperone fimC | Escherichia coli HVH 39 (4-2679949) | 665.6 | 1.80E-196 |
| FimC | 115 | WEI_00580 | ELC40122 | chaperone fimC | Escherichia coli KTE25 | 665.6 | 1.80E-196 |
| FimC | 116 | G859_04711 | EQU51487 | chaperone fimC | Escherichia coli HVH 207 (4-3113221) | 665.6 | 1.80E-196 |
| FimC | 117 | G957_04749 | EQY71509 | chaperone fimC | Escherichia coli UMEA 3268-1 | 665.6 | 1.80E-196 |
| FimC | 118 | WIY_00006 | ELI80467 | fimbrial chaperone FimC | Escherichia coli KTE137 | 665.6 | 1.80E-196 |
| FimC | 119 | G764_04662 | EQQ45241 | chaperone fimC | Escherichia coli HVH 103 (4-5904188) | 665.6 | 1.80E-196 |
| FimC | 120 | G724_04660 | EQO85495 | chaperone fimC | Escherichia coli HVH 51 (4-2172526) | 665.6 | 1.80E-196 |
| FimC | 121 | A1SI_00418 | ELE16038 | chaperone fimC | Escherichia coli KTE55 | 665.6 | 1.80E-196 |
| FimC | 122 | WK9_04507 | ELI91232 | fimbrial chaperone FimC | Escherichia coli KTE150 | 665.6 | 1.80E-196 |
| FimC | 123 | HMPREF1595_03992 | ESD04897 | gram-negative pili assembly chaperone domain protein | Escherichia coli 907672 | 663.1 | 1.00E-195 |
| FimC | 124 | HMPREF1608_03719 | ESD66976 | gram-negative pili assembly chaperone domain protein | Escherichia coli 908525 | 663.1 | 1.00E-195 |
| FimC | 125 | ECDEC2C_5324 | EHU34981 | gram-negative pili assembly chaperone, N-terminal domain protein | Escherichia coli DEC2C | 663 | 1.10E-195 |
| FimC | 126 | G713_04624 | EQO38359 | chaperone fimC | Escherichia coli HVH 38 (4-2774682) | 663 | 1.10E-195 |
| FimC | 127 | G761_01034 | EQQ43249 | chaperone fimC | Escherichia coli HVH 100 (4-2850729) | 663 | 1.10E-195 |
| FimC | 128 | G977_04846 | EQZ23811 | chaperone fimC | Escherichia coli UMEA 3585-1 | 663 | 1.10E-195 |
| FimC | 129 | G837_04724 | EQT43957 | chaperone fimC | Escherichia coli HVH 185 (4-2876639) | 663 | 1.10E-195 |
| FimC | 130 | G909_04628 | EQW60040 | chaperone fimC | Escherichia coli UMEA 3113-1 | 663 | 1.10E-195 |
| FimC | 131 | G845_04399 | EQT84574 | chaperone fimC | Escherichia coli HVH 193 (4-3331423) | 663 | 1.10E-195 |
| FimC | 132 | G881_04714 | EQV27993 | chaperone fimC | Escherichia coli KOEGE 30 (63a) | 663 | 1.10E-195 |
| FimC | 133 | T654_04030 | EYT05005 | chaperone fimC | Escherichia coli K02 | 663 | 1.10E-195 |
| FimC | 134 | ECDEC2B_0012 | EHU45222 | gram-negative pili assembly chaperone, N-terminal domain protein | Escherichia coli DEC2B | 663 | 1.10E-195 |
| FimC | 135 | G831_04203 | ETF14364 | chaperone fimC | Escherichia coli HVH 177 (4-2876612) | 663 | 1.10E-195 |
| FimC | 136 | G772_04510 | EQQ70522 | chaperone fimC | Escherichia coli HVH 111 (4-7039018) | 663 | 1.10E-195 |
| FimC | 137 | A31M_04708 | ELF33607 | chaperone fimC | Escherichia coli KTE169 | 663 | 1.10E-195 |
| FimC | 138 | A13S_00400 | ELC93705 | chaperone fimC | Escherichia coli KTE191 | 663 | 1.10E-195 |
| FimC | 139 | AF56_04387 | EZQ55260 | chaperone fimC | Escherichia coli BIDMC 83 | 663 | 1.10E-195 |
| FimC | 140 | G700_04356 | EQN79978 | chaperone fimC | Escherichia coli HVH 24 (4-5985145) | 663 | 1.10E-195 |
| FimC | 141 | G983_04258 | EQZ47988 | chaperone fimC | Escherichia coli UMEA 3656-1 | 663 | 1.10E-195 |
| FimC | 142 | A13K_00352 | ELC76553 | chaperone fimC | Escherichia coli KTE187 | 663 | 1.10E-195 |
| FimC | 143 | A1WE_00060 | ELE94951 | chaperone fimC | Escherichia coli KTE93 | 663 | 1.10E-195 |
| FimC | 144 | WI9_04458 | ELI04430 | fimbrial chaperone FimC | Escherichia coli KTE106 | 663 | 1.10E-195 |
| FimC | 145 | A13O_00076 | ELC87051 | chaperone fimC | Escherichia coli KTE189 | 663 | 1.10E-195 |
| FimC | 146 | HMPREF9553_04098 | EFJ59824 | gram-negative pili assembly chaperone domain protein | Escherichia coli MS 200-1 | 663 | 1.10E-195 |
| FimC | 147 | G693_04560 | EQN48776 | chaperone fimC | Escherichia coli HVH 17 (4-7473087) | 663 | 1.10E-195 |
| FimC | 148 | G892_04593 | EQV75845 | chaperone fimC | Escherichia coli KOEGE 70 (185a) | 663 | 1.10E-195 |
| FimC | 149 | G826_04533 | EQS94730 | chaperone fimC | Escherichia coli HVH 171 (4-3191958) | 663 | 1.10E-195 |
| FimC | 150 | WGG_04593 | ELF78852 | fimbrial chaperone FimC | Escherichia coli KTE43 | 663 | 1.10E-195 |
| FimC | 151 | WE5_03970 | EOU59198 | fimbrial chaperone FimC | Escherichia coli KTE19 | 663 | 1.10E-195 |
| FimC | 152 | A1UG_04869 | ELE46757 | chaperone fimC | Escherichia coli KTE72 | 663 | 1.10E-195 |
| FimC | 153 | ECDEC1A_5106 | EHU02582 | gram-negative pili assembly chaperone, N-terminal domain protein | Escherichia coli DEC1A | 663 | 1.10E-195 |
| FimC | 154 | A13Y_00292 | ELH19705 | fimbrial chaperone FimC | Escherichia coli KTE194 | 663 | 1.10E-195 |
| FimC | 155 | G684_04792 | EQN13463 | chaperone fimC | Escherichia coli HVH 4 (4-7276109) | 663 | 1.10E-195 |
| FimC | 156 | G773_04504 | EQQ80593 | chaperone fimC | Escherichia coli HVH 112 (4-5987253) | 663 | 1.10E-195 |
| FimC | 157 | A1UC_05350 | EOV68214 | fimbrial chaperone FimC | Escherichia coli KTE70 | 663 | 1.10E-195 |
| FimC | 158 | WCI_04700 | ELF43358 | fimbrial chaperone FimC | Escherichia coli KTE8 | 663 | 1.10E-195 |
| FimC | 159 | G934_04679 | EQX61866 | chaperone fimC | Escherichia coli UMEA 3185-1 | 663 | 1.10E-195 |
| FimC | 160 | ECKG_03435 | EGI24016 | chaperone protein FimC | Escherichia coli TA206 | 663 | 1.10E-195 |
| FimC | 161 | G974_00409 | ESK21406 | chaperone fimC | Escherichia coli UMEA 3426-1 | 663 | 1.10E-195 |
| FimC | 162 | G804_04801 | EQS28806 | chaperone fimC | Escherichia coli HVH 146 (4-3189767) | 663 | 1.10E-195 |
| FimC | 163 | G980_04559 | EQZ33060 | chaperone fimC | Escherichia coli UMEA 3617-1 | 663 | 1.10E-195 |
| FimC | 164 | WGS_04330 | ELJ64311 | fimbrial chaperone FimC | Escherichia coli KTE88 | 663 | 1.10E-195 |
| FimC | 165 | AB42_4425 | KDW79815 | chaperone protein fimC | Escherichia coli 1-392-07_S1_C2 | 663 | 1.10E-195 |
| FimC | 166 | ERS139229_02181 | CTW91708 | type I fimbrial chaperone | Escherichia coli HVH 87 (4-5977630) | 663 | 1.10E-195 |
| FimC | 167 | A1YS_00314 | ELG82370 | fimbrial chaperone FimC | Escherichia coli KTE141 | 663 | 1.10E-195 |
| FimC | 168 | G991_04528 | EQZ84865 | chaperone fimC | Escherichia coli UMEA 3703-1 | 663 | 1.10E-195 |
| FimC | 169 | WCY_00461 | ELC32636 | chaperone fimC | Escherichia coli KTE16 | 663 | 1.10E-195 |
| FimC | 170 | ECDEC1E_0066 | EHU32352 | gram-negative pili assembly chaperone, N-terminal domain protein | Escherichia coli DEC1E | 663 | 1.10E-195 |
| FimC | 171 | A1W9_04485 | EOV85034 | fimbrial chaperone FimC | Escherichia coli KTE89 | 663 | 1.10E-195 |
| FimC | 172 | ECDEC2E_5227 | EHU48713 | gram-negative pili assembly chaperone, N-terminal domain protein | Escherichia coli DEC2E | 663 | 1.10E-195 |
| FimC | 173 | A17M_04697 | ELD47221 | chaperone fimC | Escherichia coli KTE224 | 663 | 1.10E-195 |
| FimC | 174 | G887_04641 | EQV61278 | chaperone fimC | Escherichia coli KOEGE 56 (169a) | 663 | 1.10E-195 |
| FimC | 175 | G935_02342 | EQX79711 | chaperone fimC | Escherichia coli UMEA 3190-1 | 663 | 1.10E-195 |
| FimC | 176 | G953_04596 | EQY50160 | chaperone fimC | Escherichia coli UMEA 3244-1 | 663 | 1.10E-195 |
| FimC | 177 | G801_04497 | EQS09436 | chaperone fimC | Escherichia coli HVH 143 (4-5674999) | 663 | 1.10E-195 |
| FimC | 178 | WIW_04595 | ELI65831 | fimbrial chaperone FimC | Escherichia coli KTE133 | 663 | 1.10E-195 |
| FimC | 179 | WIE_00171 | ELI30709 | fimbrial chaperone FimC | Escherichia coli KTE113 | 663 | 1.10E-195 |
| FimC | 180 | G716_04678 | EQO53405 | chaperone fimC | Escherichia coli HVH 41 (4-2677849) | 663 | 1.10E-195 |
| FimC | 181 | WCG_02040 | ELF46853 | fimbrial chaperone FimC | Escherichia coli KTE6 | 663 | 1.10E-195 |
| FimC | 182 | BW75_08260 | EZA15051 | molecular chaperone FimC | Escherichia coli O81:NM str. 02-3012 | 663 | 1.10E-195 |
| FimC | 183 | ECNC101_02828 | EFM51617 | type I fimbrial chaperone | Escherichia coli NC101 | 663 | 1.10E-195 |
| FimC | 184 | G785_04656 | EQR38177 | chaperone fimC | Escherichia coli HVH 125 (4-2634716) | 663 | 1.10E-195 |
| FimC | 185 | ECKD1_07354 | EIL52298 | type I fimbrial chaperone | Escherichia coli KD1 | 663 | 1.10E-195 |
| FimC | 186 | HMPREF9545_02690 | EFU57539 | gram-negative pili assembly chaperone domain protein | Escherichia coli MS 16-3 | 663 | 1.10E-195 |
| FimC | 187 | G888_04548 | EQV60906 | chaperone fimC | Escherichia coli KOEGE 58 (171a) | 663 | 1.10E-195 |
| FimC | 188 | WKO_04577 | ELJ21561 | fimbrial chaperone FimC | Escherichia coli KTE168 | 663 | 1.10E-195 |
| FimC | 189 | A31K_01753 | ELH09234 | fimbrial chaperone FimC | Escherichia coli KTE165 | 663 | 1.10E-195 |
| FimC | 190 | AC93_4711 | EZJ47427 | chaperone protein fimC | Escherichia coli 2-005-03_S4_C2 | 663 | 1.10E-195 |
| FimC | 191 | G704_04773 | EQO01214 | chaperone fimC | Escherichia coli HVH 28 (4-0907367) | 663 | 1.10E-195 |
| FimC | 192 | G728_04398 | EQP02769 | chaperone fimC | Escherichia coli HVH 56 (4-2153033) | 663 | 1.10E-195 |
| FimC | 193 | G973_04625 | EQZ11170 | chaperone fimC | Escherichia coli UMEA 3391-1 | 663 | 1.10E-195 |
| FimC | 194 | G694_04555 | EQN58588 | chaperone fimC | Escherichia coli HVH 18 (4-8589585) | 663 | 1.10E-195 |
| FimC | 195 | WI1_04346 | ELJ90242 | fimbrial chaperone FimC | Escherichia coli KTE97 | 663 | 1.10E-195 |
| FimC | 196 | G962_04129 | EQY78986 | chaperone fimC | Escherichia coli UMEA 3304-1 | 663 | 1.10E-195 |
| FimC | 197 | WK5_00003 | ELI92890 | fimbrial chaperone FimC | Escherichia coli KTE145 | 663 | 1.10E-195 |
| FimC | 198 | G741_04601 | EQP63489 | chaperone fimC | Escherichia coli HVH 78 (4-2735946) | 663 | 1.10E-195 |
| FimC | 199 | G946_04363 | EQY23633 | chaperone fimC | Escherichia coli UMEA 3217-1 | 663 | 1.10E-195 |
| FimC | 200 | G812_04521 | EQS59383 | chaperone fimC | Escherichia coli HVH 154 (4-5636698) | 663 | 1.10E-195 |
| FimC | 201 | A137_00359 | ELC68314 | chaperone fimC | Escherichia coli KTE178 | 663 | 1.10E-195 |
| FimC | 202 | A13C_03588 | ELH36555 | fimbrial chaperone FimC | Escherichia coli KTE183 | 663 | 1.10E-195 |
| FimC | 203 | G988_04402 | ESK23468 | chaperone fimC | Escherichia coli UMEA 3693-1 | 663 | 1.10E-195 |
| FimC | 204 | G908_04267 | EQW62037 | chaperone fimC | Escherichia coli UMEA 3108-1 | 663 | 1.10E-195 |
| FimC | 205 | V415_14905 | ETE21306 | molecular chaperone FimC | Escherichia coli LAU-EC10 | 663 | 1.10E-195 |
| FimC | 206 | A1W5_00059 | ELE85057 | chaperone fimC | Escherichia coli KTE86 | 663 | 1.10E-195 |
| FimC | 207 | G877_04636 | ERA85984 | chaperone fimC | Escherichia coli HVH 228 (4-7787030) | 663 | 1.10E-195 |
| FimC | 208 | AB16_1140 | KDZ53184 | chaperone protein fimC | Escherichia coli 3-073-06_S1_C1 | 663 | 1.10E-195 |
| FimC | 209 | A15M_00063 | ELD17300 | chaperone fimC | Escherichia coli KTE206 | 663 | 1.10E-195 |
| FimC | 210 | G727_04799 | EQO93566 | chaperone fimC | Escherichia coli HVH 55 (4-2646161) | 663 | 1.10E-195 |
| FimC | 211 | A1W3_00478 | ELG38949 | fimbrial chaperone FimC | Escherichia coli KTE84 | 663 | 1.10E-195 |
| FimC | 212 | A15O_00585 | ELH67500 | fimbrial chaperone FimC | Escherichia coli KTE207 | 663 | 1.10E-195 |
| FimC | 213 | G749_05042 | EQP98042 | chaperone fimC | Escherichia coli HVH 87 (4-5977630) | 663 | 1.10E-195 |
| FimC | 214 | A13I_02522 | EOX26466 | fimbrial chaperone FimC | Escherichia coli KTE186 | 663 | 1.10E-195 |
| FimC | 215 | A151_00053 | EOV12095 | fimbrial chaperone FimC | Escherichia coli KTE195 | 663 | 1.10E-195 |
| FimC | 216 | G752_04702 | EQQ09824 | chaperone fimC | Escherichia coli HVH 90 (4-3191362) | 663 | 1.10E-195 |
| FimC | 217 | ECDEC1B_5217 | EHU04577 | gram-negative pili assembly chaperone, N-terminal domain protein | Escherichia coli DEC1B | 663 | 1.10E-195 |
| FimC | 218 | G899_04633 | EQW12606 | chaperone fimC | Escherichia coli UMEA 3022-1 | 663 | 1.10E-195 |
| FimC | 219 | CDK52692 | CDK52692 | chaperone FimC | Escherichia coli IS5 | 663 | 1.10E-195 |
| FimC | 220 | HMPREF1622_05060 | ESE27387 | gram-negative pili assembly chaperone domain protein | Escherichia coli A35218R | 663 | 1.10E-195 |
| FimC | 221 | A13U_00400 | ELH13130 | fimbrial chaperone FimC | Escherichia coli KTE192 | 663 | 1.10E-195 |
| FimC | 222 | H001_04566 | ERA28702 | chaperone fimC | Escherichia coli UMEA 3955-1 | 663 | 1.10E-195 |
| FimC | 223 | G903_04600 | EQW36496 | chaperone fimC | Escherichia coli UMEA 3053-1 | 663 | 1.10E-195 |
| FimC | 224 | G959_04572 | ESK11755 | chaperone fimC | Escherichia coli UMEA 3290-1 | 663 | 1.10E-195 |
| FimC | 225 | H003_04422 | ERA40513 | chaperone fimC | Escherichia coli UMEA 4076-1 | 663 | 1.10E-195 |
| FimC | 226 | A1YW_00071 | ELF22956 | chaperone fimC | Escherichia coli KTE143 | 663 | 1.10E-195 |
| FimC | 227 | G936_04817 | EQX71419 | chaperone fimC | Escherichia coli UMEA 3193-1 | 663 | 1.10E-195 |
| FimC | 228 | WIA_00003 | ELI21486 | fimbrial chaperone FimC | Escherichia coli KTE109 | 663 | 1.10E-195 |
| FimC | 229 | G854_04907 | EQU28356 | chaperone fimC | Escherichia coli HVH 202 (4-3163997) | 663 | 1.10E-195 |
| FimC | 230 | WCU_04649 | ELC23590 | chaperone fimC | Escherichia coli KTE15 | 663 | 1.10E-195 |
| FimC | 231 | G950_04657 | EQY37715 | chaperone fimC | Escherichia coli UMEA 3230-1 | 663 | 1.10E-195 |
| FimC | 232 | WG5_00022 | EOU99242 | fimbrial chaperone FimC | Escherichia coli KTE37 | 663 | 1.10E-195 |
| FimC | 233 | ECDEC2D_0037 | EHU49241 | gram-negative pili assembly chaperone, N-terminal domain protein | Escherichia coli DEC2D | 663 | 1.10E-195 |
| FimC | 234 | A1UO_04714 | ELE54291 | chaperone fimC | Escherichia coli KTE76 | 663 | 1.10E-195 |
| FimC | 235 | A175_00079 | ELH86319 | fimbrial chaperone FimC | Escherichia coli KTE215 | 663 | 1.10E-195 |
| FimC | 236 | WIU_04546 | ELI61782 | fimbrial chaperone FimC | Escherichia coli KTE131 | 663 | 1.10E-195 |
| FimC | 237 | WI5_04534 | ELI01599 | fimbrial chaperone FimC | Escherichia coli KTE104 | 663 | 1.10E-195 |
| FimC | 238 | G799_04635 | EQR98461 | chaperone fimC | Escherichia coli HVH 141 (4-5995973) | 663 | 1.10E-195 |
| FimC | 239 | G948_04669 | EQY34876 | chaperone fimC | Escherichia coli UMEA 3221-1 | 663 | 1.10E-195 |
| FimC | 240 | ECDEC1C_5247 | EHU02450 | gram-negative pili assembly chaperone, N-terminal domain protein | Escherichia coli DEC1C | 663 | 1.10E-195 |
| FimC | 241 | ECDEC2A_0094 | EHU33785 | chaperone protein fimC | Escherichia coli DEC2A | 663 | 1.10E-195 |
| FimC | 242 | H002_04793 | ERA28386 | chaperone fimC | Escherichia coli UMEA 4075-1 | 663 | 1.10E-195 |
| FimC | 243 | CDK45228 | CDK45228 | chaperone FimC | Escherichia coli IS1 | 663 | 1.10E-195 |
| FimC | 244 | G900_03783 | EQW24840 | chaperone fimC | Escherichia coli UMEA 3033-1 | 663 | 1.10E-195 |
| FimC | 245 | A1UA_05225 | EOV58204 | fimbrial chaperone FimC | Escherichia coli KTE69 | 663 | 1.10E-195 |
| FimC | 246 | A1UK_04966 | EOV83211 | fimbrial chaperone FimC | Escherichia coli KTE74 | 663 | 1.10E-195 |
| FimC | 247 | WG7_00032 | EOV16052 | fimbrial chaperone FimC | Escherichia coli KTE38 | 663 | 1.10E-195 |
| FimC | 248 | G970_04591 | EQZ06491 | chaperone fimC | Escherichia coli UMEA 3341-1 | 663 | 1.10E-195 |
| FimC | 249 | G907_04448 | EQW50321 | chaperone fimC | Escherichia coli UMEA 3097-1 | 663 | 1.10E-195 |
| FimC | 250 | EC236275_1846 | EFR17172 | chaperone protein fimC | Escherichia coli 2362-75 | 663 | 1.10E-195 |
| FimC | 251 | ERS139198_00303 | CUA00177 | type I fimbrial chaperone | Escherichia coli (GCA_001286605) | 662.5 | 1.60E-195 |
| FimC | 252 | A1S1_04327 | ELF92165 | fimbrial chaperone FimC | Escherichia coli KTE46 | 661.3 | 3.60E-195 |
| FimC | 253 | G811_04675 | EQS44720 | chaperone fimC | Escherichia coli HVH 153 (3-9344314) | 661.3 | 3.60E-195 |
| FimC | 254 | ERS085393_00508 | CTT95197 | type I fimbrial chaperone | Escherichia coli O78:H11 (strain H10407 / ETEC) | 661.3 | 3.60E-195 |
| FimC | 255 | ECP03052605_4608 | ENG08606 | chaperone protein fimC | Escherichia coli P0305260.5 | 661.3 | 3.60E-195 |
| FimC | 256 | BW69_19275 | EZA33088 | molecular chaperone FimC | Escherichia coli O103:H11 str. 04-3023 | 661.3 | 3.60E-195 |
| FimC | 257 | WGW_00012 | ELJ90793 | fimbrial chaperone FimC | Escherichia coli KTE94 | 661.3 | 3.60E-195 |
| FimC | 258 | AC14_4774 | KEN70505 | chaperone protein fimC | Escherichia coli 2-052-05_S3_C2 | 661.3 | 3.60E-195 |
| FimC | 259 | ECP03052607_4694 | ENG10879 | chaperone protein fimC | Escherichia coli P0305260.7 | 661.3 | 3.60E-195 |
| FimC | 260 | HMPREF9550_01115 | EFK26737 | gram-negative pili assembly chaperone domain protein | Escherichia coli MS 187-1 | 661.3 | 3.60E-195 |
| FimC | 261 | G850_04500 | EQU05502 | chaperone fimC | Escherichia coli HVH 198 (4-3206106) | 661.3 | 3.60E-195 |
| FimC | 262 | AE17_03596 | KDG84295 | chaperone fimC | Escherichia coli UCI 58 | 661.3 | 3.60E-195 |
| FimC | 263 | ECP030526015_4645 | ENF93184 | chaperone protein fimC | Escherichia coli P0305260.15 | 661.3 | 3.60E-195 |
| FimC | 264 | ECP030526011_4682 | ENF78616 | chaperone protein fimC | Escherichia coli P0305260.11 | 661.3 | 3.60E-195 |
| FimC | 265 | ECP03052602_4710 | ENE03241 | chaperone protein fimC | Escherichia coli P0305260.2 | 661.3 | 3.60E-195 |
| FimC | 266 | A13G_00246 | EOX26802 | fimbrial chaperone FimC | Escherichia coli KTE185 | 661.3 | 3.60E-195 |
| FimC | 267 | G910_04288 | EQW69324 | chaperone fimC | Escherichia coli UMEA 3117-1 | 661.3 | 3.60E-195 |
| FimC | 268 | AC42_4716 | KDV77019 | chaperone protein fimC | Escherichia coli 2-052-05_S3_C3 | 661.3 | 3.60E-195 |
| FimC | 269 | EC5411_15322 | EIL63547 | type-1 fimbrial chaperone FimC | Escherichia coli 541-1 | 661.3 | 3.60E-195 |
| FimC | 270 | ECP030526010_4663 | ENF69302 | chaperone protein fimC | Escherichia coli P0305260.10 | 661.3 | 3.60E-195 |
| FimC | 271 | AB22_1079 | KEL52268 | chaperone protein fimC | Escherichia coli 6-175-07_S1_C1 | 661.3 | 3.60E-195 |
| FimC | 272 | G721_04520 | EQO77492 | chaperone fimC | Escherichia coli HVH 46 (4-2758776) | 661.3 | 3.60E-195 |
| FimC | 273 | HMPREF1604_02608 | ESD40674 | gram-negative pili assembly chaperone domain protein | Escherichia coli 908519 | 661.3 | 3.60E-195 |
| FimC | 274 | AE16_04222 | KDG81432 | chaperone fimC | Escherichia coli UCI 57 | 661.3 | 3.60E-195 |
| FimC | 275 | ECP02994389_4609 | ENC26270 | chaperone protein fimC | Escherichia coli P0299438.9 | 661.3 | 3.60E-195 |
| FimC | 276 | AB84_4594 | KDT14122 | chaperone protein fimC | Escherichia coli 2-052-05_S3_C1 | 661.3 | 3.60E-195 |
| FimC | 277 | ETEC_4628 | CBJ04137 | fimbrial chaperone | Escherichia coli ETEC H10407 | 661.3 | 3.60E-195 |
| FimC | 278 | G923_04700 | EQX13148 | chaperone fimC | Escherichia coli UMEA 3160-1 | 661.3 | 3.60E-195 |
| FimC | 279 | A1UY_00576 | ELE75347 | chaperone fimC | Escherichia coli KTE81 | 661.3 | 3.60E-195 |
| FimC | 280 | OQA_21608 | EIA33921 | type-1 fimbrial chaperone FimC | Escherichia coli SCI-07 | 661.3 | 3.60E-195 |
| FimC | 281 | WGK_00469 | ELF70108 | fimbrial chaperone FimC | Escherichia coli KTE45 | 661.3 | 3.60E-195 |
| FimC | 282 | ECP03052609_4672 | ENG27743 | chaperone protein fimC | Escherichia coli P0305260.9 | 661.3 | 3.60E-195 |
| FimC | 283 | HMPREF9532_05236 | EGB74377 | gram-negative pili assembly chaperone domain protein | Escherichia coli MS 57-2 | 661.3 | 3.60E-195 |
| FimC | 284 | ECP03052601_4492 | EMZ89919 | chaperone protein fimC | Escherichia coli P0305260.1 | 661.3 | 3.60E-195 |
| FimC | 285 | ECTW07793_4521 | EII94627 | gram-negative pili assembly chaperone, N-terminal domain protein | Escherichia coli TW07793 | 660.9 | 5.10E-195 |
| FimC | 286 | ERS085358_04328 | CTT02114 | type I fimbrial chaperone | Escherichia coli O127:H6 (strain E2348/69 / EPEC) | 660.6 | 6.10E-195 |
| FimC | 287 | E2348C_4623 | CAS12171 | chaperone, periplasmic | Escherichia coli O127:H6 str. E2348/69 | 660.6 | 6.10E-195 |
| FimC | 288 | G690_04427 | ESP12978 | chaperone fimC | Escherichia coli HVH 12 (4-7653042) | 660.5 | 6.50E-195 |
| FimC | 289 | EC2731150_4986 | EMW70553 | chaperone protein fimC | Escherichia coli 2731150 | 660.5 | 6.50E-195 |
| FimC | 290 | P811_03273 | ETY52639 | chaperone fimC | Escherichia coli BIDMC 49b | 660.5 | 6.50E-195 |
| FimC | 291 | WC9_04682 | EOU44755 | fimbrial chaperone FimC | Escherichia coli KTE231 | 660.5 | 6.50E-195 |
| FimC | 292 | WIG_04408 | ELI23393 | fimbrial chaperone FimC | Escherichia coli KTE117 | 660.5 | 6.50E-195 |
| FimC | 293 | G774_04773 | EQQ82417 | chaperone fimC | Escherichia coli HVH 113 (4-7535473) | 660.5 | 6.50E-195 |
| FimC | 294 | G921_02633 | EQX06248 | chaperone fimC | Escherichia coli UMEA 3155-1 | 660.5 | 6.50E-195 |
| FimC | 295 | AB69_4869 | EYE31514 | chaperone protein fimC | Escherichia coli 1-110-08_S1_C3 | 660.5 | 6.50E-195 |
| FimC | 296 | AE52_03242 | KDG33746 | chaperone fimC | Escherichia coli BIDMC 77 | 660.5 | 6.50E-195 |
| FimC | 297 | AB29_4953 | KDW27097 | chaperone protein fimC | Escherichia coli 2-177-06_S1_C2 | 660.5 | 6.50E-195 |
| FimC | 298 | HMPREF1594_01820 | ESC98957 | gram-negative pili assembly chaperone domain protein | Escherichia coli 907446 | 660.5 | 6.50E-195 |
| FimC | 299 | EC2872000_5043 | EMV50829 | chaperone protein fimC | Escherichia coli 2872000 | 660.5 | 6.50E-195 |
| FimC | 300 | L454_04736 | ERO99456 | chaperone fimC | Escherichia coli BIDMC 19C | 660.5 | 6.50E-195 |
| FimC | 301 | WKU_04620 | ELJ37075 | fimbrial chaperone FimC | Escherichia coli KTE177 | 660.5 | 6.50E-195 |
| FimC | 302 | AC72_4739 | KDY08107 | chaperone protein fimC | Escherichia coli 2-316-03_S4_C1 | 660.5 | 6.50E-195 |
| FimC | 303 | A135_00502 | ELH31328 | fimbrial chaperone FimC | Escherichia coli KTE175 | 660.5 | 6.50E-195 |
| FimC | 304 | A15I_04650 | ELD00674 | chaperone fimC | Escherichia coli KTE204 | 660.5 | 6.50E-195 |
| FimC | 305 | ECO10030_24274 | EJF01931 | chaperone FimC, periplasmic | Escherichia coli O26:H11 str. CVM10030 | 660.5 | 6.50E-195 |
| FimC | 306 | A17W_03404 | ELH96321 | fimbrial chaperone FimC | Escherichia coli KTE229 | 660.5 | 6.50E-195 |
| FimC | 307 | AC68_4695 | KDW12848 | chaperone protein fimC | Escherichia coli 2-156-04_S4_C1 | 660.5 | 6.50E-195 |
| FimC | 308 | G821_04862 | EQS73506 | chaperone fimC | Escherichia coli HVH 163 (4-4697553) | 660.5 | 6.50E-195 |
| FimC | 309 | AE38_04461 | KDF79913 | chaperone fimC | Escherichia coli BIDMC 63 | 660.5 | 6.50E-195 |
| FimC | 310 | ECEC1865_5755 | EKJ10635 | type I fimbrial chaperone | Escherichia coli EC1865 | 660.5 | 6.50E-195 |
| FimC | 311 | G739_04733 | EQP57046 | chaperone fimC | Escherichia coli HVH 76 (4-2538717) | 660.5 | 6.50E-195 |
| FimC | 312 | A1U9_04990 | EOV54782 | fimbrial chaperone FimC | Escherichia coli KTE68 | 660.5 | 6.50E-195 |
| FimC | 313 | G880_04829 | ERA98248 | chaperone fimC | Escherichia coli KOEGE 10 (25a) | 660.5 | 6.50E-195 |
| FimC | 314 | ECK5_20320 | CCP96084 | chaperone FimC | Escherichia coli O10:K5(L):H4 str. ATCC 23506 | 660.5 | 6.50E-195 |
| FimC | 315 | AE24_04578 | KDG86405 | chaperone fimC | Escherichia coli UCI 65 | 660.5 | 6.50E-195 |
| FimC | 316 | AC71_4564 | KEM85263 | chaperone protein fimC | Escherichia coli 2-222-05_S4_C1 | 660.5 | 6.50E-195 |
| FimC | 317 | AD04_4698 | KEL22180 | chaperone protein fimC | Escherichia coli 5-172-05_S4_C2 | 660.5 | 6.50E-195 |
| FimC | 318 | G964_04340 | EQY81872 | chaperone fimC | Escherichia coli UMEA 3317-1 | 660.5 | 6.50E-195 |
| FimC | 319 | HMPREF1614_04606 | ESD94178 | gram-negative pili assembly chaperone domain protein | Escherichia coli 908624 | 660.5 | 6.50E-195 |
| FimC | 320 | ECDEC7E_4979 | EHV94340 | chaperone protein fimC | Escherichia coli DEC7E | 660.5 | 6.50E-195 |
| FimC | 321 | AC76_0694 | KEL40644 | chaperone protein fimC | Escherichia coli 5-172-05_S4_C1 | 660.5 | 6.50E-195 |
| FimC | 322 | WKC_04507 | ELJ04639 | fimbrial chaperone FimC | Escherichia coli KTE157 | 660.5 | 6.50E-195 |
| FimC | 323 | G795_04496 | EQR82326 | chaperone fimC | Escherichia coli HVH 137 (4-2124971) | 660.5 | 6.50E-195 |
| FimC | 324 | G737_04816 | EQP45589 | chaperone fimC | Escherichia coli HVH 73 (4-2393174) | 660.5 | 6.50E-195 |
| FimC | 325 | L411_05110 | ERO93603 | chaperone fimC | Escherichia coli BWH 24 | 660.5 | 6.50E-195 |
| FimC | 326 | A1SK_02381 | ELE13876 | chaperone fimC | Escherichia coli KTE56 | 660.5 | 6.50E-195 |
| FimC | 327 | AC20_4674 | KDY56895 | chaperone protein fimC | Escherichia coli 2-460-02_S3_C2 | 660.5 | 6.50E-195 |
| FimC | 328 | G803_04919 | EQS19592 | chaperone fimC | Escherichia coli HVH 145 (4-5672112) | 660.5 | 6.50E-195 |
| FimC | 329 | G695_04684 | EQN65037 | chaperone fimC | Escherichia coli HVH 19 (4-7154984) | 660.5 | 6.50E-195 |
| FimC | 330 | ECFG_03458 | EFI22336 | chaperone fimC | Escherichia coli FVEC1302 | 660.5 | 6.50E-195 |
| FimC | 331 | AC90_4525 | KDT81435 | chaperone protein fimC | Escherichia coli 3-475-03_S4_C1 | 660.5 | 6.50E-195 |
| FimC | 332 | L429_09123 | ETY29994 | chaperone fimC | Escherichia coli BIDMC 3 | 660.5 | 6.50E-195 |
| FimC | 333 | ECDEC10A_0024 | EHW70239 | gram-negative pili assembly chaperone, N-terminal domain protein | Escherichia coli DEC10A | 660.5 | 6.50E-195 |
| FimC | 334 | AD01_4595 | KDY41279 | chaperone protein fimC | Escherichia coli 2-427-07_S4_C2 | 660.5 | 6.50E-195 |
| FimC | 335 | HMPREF1610_03975 | ESD66055 | gram-negative pili assembly chaperone domain protein | Escherichia coli 908555 | 660.5 | 6.50E-195 |
| FimC | 336 | M13_15241 | EST67443 | chaperone FimC, periplasmic | Escherichia coli P4-96 | 660.5 | 6.50E-195 |
| FimC | 337 | ECGG_03266 | EFF02902 | chaperone fimC | Escherichia coli FVEC1412 | 660.5 | 6.50E-195 |
| FimC | 338 | G742_04542 | EQP66789 | chaperone fimC | Escherichia coli HVH 79 (4-2512823) | 660.5 | 6.50E-195 |
| FimC | 339 | G434_03627 | EOW71233 | fimbrial chaperone FimC | Escherichia sp. KTE172 | 660.5 | 6.50E-195 |
| FimC | 340 | L453_09023 | ETX96659 | chaperone fimC | Escherichia coli BIDMC 19B | 660.5 | 6.50E-195 |
| FimC | 341 | G883_04506 | EQV39956 | chaperone fimC | Escherichia coli KOEGE 33 (68a) | 660.5 | 6.50E-195 |
| FimC | 342 | AB92_3659 | KDY85712 | chaperone protein fimC | Escherichia coli 2-474-04_S3_C1 | 660.5 | 6.50E-195 |
| FimC | 343 | ECO9952_02429 | EJF04538 | chaperone FimC, periplasmic | Escherichia coli O26:H11 str. CVM9952 | 660.5 | 6.50E-195 |
| FimC | 344 | A1WY_00578 | ELF02994 | chaperone fimC | Escherichia coli KTE111 | 660.5 | 6.50E-195 |
| FimC | 345 | ECP03018674_4980 | ENA27976 | chaperone protein fimC | Escherichia coli P0301867.4 | 660.5 | 6.50E-195 |
| FimC | 346 | EC3006_4957 | EKI32770 | type I fimbrial chaperone | Escherichia coli 3006 | 660.5 | 6.50E-195 |
| FimC | 347 | WKG_04664 | ELJ07780 | fimbrial chaperone FimC | Escherichia coli KTE163 | 660.5 | 6.50E-195 |
| FimC | 348 | ESSG_00228 | EIG50738 | chaperone fimC | Escherichia coli H730 | 660.5 | 6.50E-195 |
| FimC | 349 | ECTW00353_4783 | EKI21420 | type I fimbrial chaperone | Escherichia coli TW00353 | 660.5 | 6.50E-195 |
| FimC | 350 | G807_04424 | EQS33138 | chaperone fimC | Escherichia coli HVH 149 (4-4451880) | 660.5 | 6.50E-195 |
| FimC | 351 | CFSAN001630_09025 | EKU05480 | type I fimbrial chaperone | Escherichia coli O111:H11 str. CFSAN001630 | 660.5 | 6.50E-195 |
| FimC | 352 | G681_04679 | EQN01356 | chaperone fimC | Escherichia coli HVH 1 (4-6876161) | 660.5 | 6.50E-195 |
| FimC | 353 | G702_04602 | EQN87822 | chaperone fimC | Escherichia coli HVH 26 (4-5703913) | 660.5 | 6.50E-195 |
| FimC | 354 | BX56_22445 | EYU87670 | molecular chaperone FimC | Escherichia coli O45:H2 str. 2010C-3876 | 660.5 | 6.50E-195 |
| FimC | 355 | HMPREF1596_02537 | ESD11598 | gram-negative pili assembly chaperone domain protein | Escherichia coli 907700 | 660.5 | 6.50E-195 |
| FimC | 356 | G841_04596 | EQT69917 | chaperone fimC | Escherichia coli HVH 189 (4-3220125) | 660.5 | 6.50E-195 |
| FimC | 357 | BY42_11160 | EYV92419 | molecular chaperone FimC | Escherichia coli O6:H16 str. 99-3165 | 660.5 | 6.50E-195 |
| FimC | 358 | G836_04864 | EQT38038 | chaperone fimC | Escherichia coli HVH 184 (4-3343286) | 660.5 | 6.50E-195 |
| FimC | 359 | A1YE_00636 | EOW43064 | fimbrial chaperone FimC | Escherichia coli KTE127 | 660.5 | 6.50E-195 |
| FimC | 360 | WEQ_04192 | ELF82191 | fimbrial chaperone FimC | Escherichia coli KTE29 | 660.5 | 6.50E-195 |
| FimC | 361 | HMPREF9539_03155 | EFU46266 | gram-negative pili assembly chaperone domain protein | Escherichia coli MS 110-3 | 660.5 | 6.50E-195 |
| FimC | 362 | AE40_03893 | KDF94833 | chaperone fimC | Escherichia coli BIDMC 65 | 660.5 | 6.50E-195 |
| FimC | 363 | ECP03019043_4924 | END86737 | chaperone protein fimC | Escherichia coli P0301904.3 | 660.5 | 6.50E-195 |
| FimC | 364 | ECB41_4813 | EIJ01572 | gram-negative pili assembly chaperone, N-terminal domain protein | Escherichia coli B41 | 660.5 | 6.50E-195 |
| FimC | 365 | AD25_4675 | KDV76740 | chaperone protein fimC | Escherichia coli 2-052-05_S4_C3 | 660.5 | 6.50E-195 |
| FimC | 366 | AC46_3696 | KDX84857 | chaperone protein fimC | Escherichia coli 2-222-05_S3_C3 | 660.5 | 6.50E-195 |
| FimC | 367 | B185_026117 | ELL38984 | type I fimbrial chaperone | Escherichia coli J96 | 660.5 | 6.50E-195 |
| FimC | 368 | AC04_4514 | KDT32989 | chaperone protein fimC | Escherichia coli 3-105-05_S3_C1 | 660.5 | 6.50E-195 |
| FimC | 369 | ECP03018677_5004 | ENH04211 | chaperone protein fimC | Escherichia coli P0301867.7 | 660.5 | 6.50E-195 |
| FimC | 370 | P423_24550 | AGY86959 | molecular chaperone FimC | Escherichia coli JJ1886 | 660.5 | 6.50E-195 |
| FimC | 371 | G771_04836 | EQQ61859 | chaperone fimC | Escherichia coli HVH 110 (4-6978754) | 660.5 | 6.50E-195 |
| FimC | 372 | EcB171_2739 | EDX31637 | chaperone protein FimC | Escherichia coli B171 | 660.5 | 6.50E-195 |
| FimC | 373 | A1UW_04761 | ELE66181 | chaperone fimC | Escherichia coli KTE80 | 660.5 | 6.50E-195 |
| FimC | 374 | WG3_00194 | EOU96441 | fimbrial chaperone FimC | Escherichia coli KTE36 | 660.5 | 6.50E-195 |
| FimC | 375 | G708_04663 | EQO17949 | chaperone fimC | Escherichia coli HVH 32 (4-3773988) | 660.5 | 6.50E-195 |
| FimC | 376 | G787_04668 | EQR49488 | chaperone fimC | Escherichia coli HVH 127 (4-7303629) | 660.5 | 6.50E-195 |
| FimC | 377 | EC2874_20585 | KRR54441 | type I fimbrial chaperone | Escherichia coli VL2874 | 660.5 | 6.50E-195 |
| FimC | 378 | G769_04515 | ESP40220 | chaperone fimC | Escherichia coli HVH 108 (4-6924867) | 660.5 | 6.50E-195 |
| FimC | 379 | EC178850_4560 | ENG83830 | chaperone protein fimC | Escherichia coli 178850 | 660.5 | 6.50E-195 |
| FimC | 380 | CR63_21705 | KKJ99657 | molecular chaperone FimC | Escherichia coli NB8 | 660.5 | 6.50E-195 |
| FimC | 381 | ECLG_03393 | EGI38217 | periplasmic chaperone | Escherichia coli TA271 | 660.5 | 6.50E-195 |
| FimC | 382 | AE50_04502 | KDG21484 | chaperone fimC | Escherichia coli BIDMC 75 | 660.5 | 6.50E-195 |
| FimC | 383 | ECSTECC16502_0300 | EGW75274 | chaperone protein fimC | Escherichia coli STEC_C165-02 | 660.5 | 6.50E-195 |
| FimC | 384 | AC95_4860 | KDV78002 | chaperone protein fimC | Escherichia coli 2-052-05_S4_C2 | 660.5 | 6.50E-195 |
| FimC | 385 | ECO26_5513 | BAI28635 | chaperone FimC, periplasmic | Escherichia coli O26:H11 str. 11368 | 660.5 | 6.50E-195 |
| FimC | 386 | G861_04235 | EQU61548 | chaperone fimC | Escherichia coli HVH 209 (4-3062651) | 660.5 | 6.50E-195 |
| FimC | 387 | ERDG_03719 | EGB35883 | gram-negative pili assembly chaperone domain-containing protein | Escherichia coli E482 | 660.5 | 6.50E-195 |
| FimC | 388 | ECO9340_01855 | EIL09198 | chaperone FimC, periplasmic | Escherichia coli O103:H25 str. CVM9340 | 660.5 | 6.50E-195 |
| FimC | 389 | L428_08942 | ETY35374 | chaperone fimC | Escherichia coli BIDMC 2B | 660.5 | 6.50E-195 |
| FimC | 390 | A1WI_00372 | EOW04857 | fimbrial chaperone FimC | Escherichia coli KTE98 | 660.5 | 6.50E-195 |
| FimC | 391 | N444_08260 | ETS27567 | molecular chaperone FimC | Escherichia coli O6:H16:CFA/II str. B2C | 660.5 | 6.50E-195 |
| FimC | 392 | ECSTECS1191_0258 | EGX21682 | chaperone protein fimC | Escherichia coli STEC_S1191 | 660.5 | 6.50E-195 |
| FimC | 393 | WK3_04469 | ELI79073 | fimbrial chaperone FimC | Escherichia coli KTE139 | 660.5 | 6.50E-195 |
| FimC | 394 | ECC34666_5078 | EMV14117 | chaperone protein fimC | Escherichia coli C-34666 | 660.5 | 6.50E-195 |
| FimC | 395 | A1YQ_00362 | ELG76798 | fimbrial chaperone FimC | Escherichia coli KTE140 | 660.5 | 6.50E-195 |
| FimC | 396 | HMPREF1621_01429 | ESE36135 | gram-negative pili assembly chaperone domain protein | Escherichia coli A25922R | 660.5 | 6.50E-195 |
| FimC | 397 | ECDEC11D_5085 | EHX04066 | chaperone protein fimC | Escherichia coli DEC11D | 660.5 | 6.50E-195 |
| FimC | 398 | G789_04712 | EQR56314 | chaperone fimC | Escherichia coli HVH 130 (4-7036876) | 660.5 | 6.50E-195 |
| FimC | 399 | ECD_04185 | ACT45966 | chaperone, periplasmic | Escherichia coli BL21(DE3) | 660.5 | 6.50E-195 |
| FimC | 400 | HMPREF1599_04104 | ESA83516 | gram-negative pili assembly chaperone domain protein | Escherichia coli 907713 | 660.5 | 6.50E-195 |
| FimC | 401 | L408_00429 | ETY51032 | chaperone fimC | Escherichia coli BWH 40 | 660.5 | 6.50E-195 |
| FimC | 402 | WGM_04855 | ELJ61287 | fimbrial chaperone FimC | Escherichia coli KTE82 | 660.5 | 6.50E-195 |
| FimC | 403 | AB93_4332 | KEL47610 | chaperone protein fimC | Escherichia coli 5-172-05_S3_C1 | 660.5 | 6.50E-195 |
| FimC | 404 | HMPREF1605_05326 | ESD45674 | gram-negative pili assembly chaperone domain protein | Escherichia coli 908521 | 660.5 | 6.50E-195 |
| FimC | 405 | A15Q_04924 | ELD16699 | chaperone fimC | Escherichia coli KTE208 | 660.5 | 6.50E-195 |
| FimC | 406 | EC5761_17129 | EIL65024 | chaperone FimC, periplasmic | Escherichia coli 576-1 | 660.5 | 6.50E-195 |
| FimC | 407 | ECO9942_09765 | EIL30298 | chaperone FimC, periplasmic | Escherichia coli O26:H11 str. CVM9942 | 660.5 | 6.50E-195 |
| FimC | 408 | G897_04345 | EQW07887 | chaperone fimC | Escherichia coli KOEGE 131 (358a) | 660.5 | 6.50E-195 |
| FimC | 409 | ECAD30_15110 | EKJ83605 | gram-negative pili assembly chaperone protein | Escherichia coli AD30 | 660.5 | 6.50E-195 |
| FimC | 410 | G895_04776 | EQV95298 | chaperone fimC | Escherichia coli KOEGE 77 (202a) | 660.5 | 6.50E-195 |
| FimC | 411 | EcE22_2410 | EDV83330 | chaperone protein FimC | Escherichia coli E22 | 660.5 | 6.50E-195 |
| FimC | 412 | BU63_12670 | KDV73212 | molecular chaperone FimC | Escherichia coli O118:H16 str. 07-4255 | 660.5 | 6.50E-195 |
| FimC | 413 | ESMG_00469 | EIF87162 | chaperone fimC | Escherichia coli M919 | 660.5 | 6.50E-195 |
| FimC | 414 | G750_04676 | EQP98729 | chaperone fimC | Escherichia coli HVH 88 (4-5854636) | 660.5 | 6.50E-195 |
| FimC | 415 | AD03_3639 | KDZ04738 | chaperone protein fimC | Escherichia coli 2-474-04_S4_C2 | 660.5 | 6.50E-195 |
| FimC | 416 | BY40_21440 | EZA68178 | molecular chaperone FimC | Escherichia coli O157:H16 str. 98-3133 | 660.5 | 6.50E-195 |
| FimC | 417 | ECP03022934_5018 | ENE25986 | chaperone protein fimC | Escherichia coli P0302293.4 | 660.5 | 6.50E-195 |
| FimC | 418 | ECP03022939_4796 | ENE44999 | chaperone protein fimC | Escherichia coli P0302293.9 | 660.5 | 6.50E-195 |
| FimC | 419 | AC91_4609 | KEM00769 | chaperone protein fimC | Escherichia coli 6-175-07_S4_C1 | 660.5 | 6.50E-195 |
| FimC | 420 | BX40_01930 | EYV52384 | molecular chaperone FimC | Escherichia coli O103:H11 str. 2010C-3214 | 660.5 | 6.50E-195 |
| FimC | 421 | ERLG_03379 | EGB51157 | gram-negative pili assembly chaperone domain-containing protein | Escherichia coli H263 | 660.5 | 6.50E-195 |
| FimC | 422 | ECO9553_13134 | EJE88230 | chaperone FimC, periplasmic | Escherichia coli O111:H11 str. CVM9553 | 660.5 | 6.50E-195 |
| FimC | 423 | WAS_00525 | EOX00055 | fimbrial chaperone FimC | Escherichia coli KTE1 | 660.5 | 6.50E-195 |
| FimC | 424 | EC990741_4989 | EIH11076 | gram-negative pili assembly chaperone, N-terminal domain protein | Escherichia coli 97.0259 | 660.5 | 6.50E-195 |
| FimC | 425 | AB34_5287 | KEO35347 | chaperone protein fimC | Escherichia coli 2-460-02_S1_C2 | 660.5 | 6.50E-195 |
| FimC | 426 | WEM_01816 | EOU79645 | fimbrial chaperone FimC | Escherichia coli KTE27 | 660.5 | 6.50E-195 |
| FimC | 427 | ECO10021_11217 | EJE78924 | chaperone FimC, periplasmic | Escherichia coli O26:H11 str. CVM10021 | 660.5 | 6.50E-195 |
| FimC | 428 | G697_04683 | EQN69713 | chaperone fimC | Escherichia coli HVH 21 (4-4517873) | 660.5 | 6.50E-195 |
| FimC | 429 | G933_04562 | EQX63265 | chaperone fimC | Escherichia coli UMEA 3180-1 | 660.5 | 6.50E-195 |
| FimC | 430 | ECDEC10E_5187 | EHW84102 | gram-negative pili assembly chaperone, N-terminal domain protein | Escherichia coli DEC10E | 660.5 | 6.50E-195 |
| FimC | 431 | ECDEC7C_0028 | EHV91091 | gram-negative pili assembly chaperone, N-terminal domain protein | Escherichia coli DEC7C | 660.5 | 6.50E-195 |
| FimC | 432 | A13A_04992 | EOW90158 | fimbrial chaperone FimC | Escherichia coli KTE182 | 660.5 | 6.50E-195 |
| FimC | 433 | WC3_00369 | EOU56824 | fimbrial chaperone FimC | Escherichia coli KTE35 | 660.5 | 6.50E-195 |
| FimC | 434 | G732_04723 | EQP20026 | chaperone fimC | Escherichia coli HVH 63 (4-2542528) | 660.5 | 6.50E-195 |
| FimC | 435 | WKW_04670 | ELJ46787 | fimbrial chaperone FimC | Escherichia coli KTE179 | 660.5 | 6.50E-195 |
| FimC | 436 | G901_04566 | EQW26672 | chaperone fimC | Escherichia coli UMEA 3041-1 | 660.5 | 6.50E-195 |
| FimC | 437 | A1SU_00022 | EOV57281 | fimbrial chaperone FimC | Escherichia coli KTE61 | 660.5 | 6.50E-195 |
| FimC | 438 | AC23_4947 | KEN17978 | chaperone protein fimC | Escherichia coli 7-233-03_S3_C2 | 660.5 | 6.50E-195 |
| FimC | 439 | A317_02445 | ELG97931 | fimbrial chaperone FimC | Escherichia coli KTE154 | 660.5 | 6.50E-195 |
| FimC | 440 | AD26_4638 | KDX37695 | chaperone protein fimC | Escherichia coli 2-156-04_S4_C3 | 660.5 | 6.50E-195 |
| FimC | 441 | AB11_4749 | EYD78652 | chaperone protein fimC | Escherichia coli 1-176-05_S1_C1 | 660.5 | 6.50E-195 |
| FimC | 442 | AC41_4728 | KDA82231 | chaperone protein fimC | Escherichia coli 2-011-08_S3_C3 | 660.5 | 6.50E-195 |
| FimC | 443 | BX12_03920 | EZE30436 | molecular chaperone FimC | Escherichia coli O69:H11 str. 2009C-3601 | 660.5 | 6.50E-195 |
| FimC | 444 | HMPREF1588_04976 | ESA64417 | gram-negative pili assembly chaperone domain protein | Escherichia coli 110957 | 660.5 | 6.50E-195 |
| FimC | 445 | AD02_4541 | KDY64214 | chaperone protein fimC | Escherichia coli 2-460-02_S4_C2 | 660.5 | 6.50E-195 |
| FimC | 446 | A15A_00191 | EOV27215 | fimbrial chaperone FimC | Escherichia coli KTE200 | 660.5 | 6.50E-195 |
| FimC | 447 | AB49_4571 | KEK90911 | chaperone protein fimC | Escherichia coli 4-203-08_S1_C2 | 660.5 | 6.50E-195 |
| FimC | 448 | G941_04670 | EQX95574 | chaperone fimC | Escherichia coli UMEA 3206-1 | 660.5 | 6.50E-195 |
| FimC | 449 | WIS_04720 | ELI51550 | fimbrial chaperone FimC | Escherichia coli KTE129 | 660.5 | 6.50E-195 |
| FimC | 450 | AB98_5063 | EYD78766 | chaperone protein fimC | Escherichia coli 1-176-05_S3_C1 | 660.5 | 6.50E-195 |
| FimC | 451 | AC22_0089 | KEL87437 | chaperone protein fimC | Escherichia coli 5-366-08_S3_C2 | 660.5 | 6.50E-195 |
| FimC | 452 | G979_04820 | EQZ33241 | chaperone fimC | Escherichia coli UMEA 3609-1 | 660.5 | 6.50E-195 |
| FimC | 453 | PGC_10100 | CDP75629 | Putative uncharacterized protein | Escherichia coli D6-117.29 | 660.5 | 6.50E-195 |
| FimC | 454 | A171_04109 | ELD28731 | chaperone fimC | Escherichia coli KTE213 | 660.5 | 6.50E-195 |
| FimC | 455 | G890_04971 | EQV77926 | chaperone fimC | Escherichia coli KOEGE 62 (175a) | 660.5 | 6.50E-195 |
| FimC | 456 | EC970264_4951 | KIO83574 | gram-negative pili assembly chaperone, N-terminal domain protein | Escherichia coli 97.0264 | 660.5 | 6.50E-195 |
| FimC | 457 | AB26_4845 | EZK15592 | chaperone protein fimC | Escherichia coli 2-011-08_S1_C2 | 660.5 | 6.50E-195 |
| FimC | 458 | A1YK_00100 | EOW60894 | fimbrial chaperone FimC | Escherichia coli KTE134 | 660.5 | 6.50E-195 |
| FimC | 459 | L343_1236 | ESS97637 | chaperone FimC | Escherichia coli CE549 | 660.5 | 6.50E-195 |
| FimC | 460 | G965_04424 | EQY93500 | chaperone fimC | Escherichia coli UMEA 3318-1 | 660.5 | 6.50E-195 |
| FimC | 461 | ECRM12761_25700 | AHY68177 | chaperone FimC | Escherichia coli O145:H28 str. RM12761 | 660.5 | 6.50E-195 |
| FimC | 462 | EC930624_5291 | EIH67784 | gram-negative pili assembly chaperone, N-terminal domain protein | Escherichia coli 93.0624 | 660.5 | 6.50E-195 |
| FimC | 463 | WKQ_00005 | ELJ45707 | fimbrial chaperone FimC | Escherichia coli KTE174 | 660.5 | 6.50E-195 |
| FimC | 464 | ECDEC11B_5028 | EHW98403 | gram-negative pili assembly chaperone, N-terminal domain protein | Escherichia coli DEC11B | 660.5 | 6.50E-195 |
| FimC | 465 | ECMP02155211_4662 | EMU56361 | chaperone protein fimC | Escherichia coli MP021552.11 | 660.5 | 6.50E-195 |
| FimC | 466 | G990_04481 | EQZ72254 | chaperone fimC | Escherichia coli UMEA 3702-1 | 660.5 | 6.50E-195 |
| FimC | 467 | G706_04685 | EQO09237 | chaperone fimC | Escherichia coli HVH 30 (4-2661829) | 660.5 | 6.50E-195 |
| FimC | 468 | AC70_1385 | KDW89247 | chaperone protein fimC | Escherichia coli 2-210-07_S4_C1 | 660.5 | 6.50E-195 |
| FimC | 469 | EC2785200_4681 | EMW30262 | chaperone protein fimC | Escherichia coli 2785200 | 660.5 | 6.50E-195 |
| FimC | 470 | EC900105_5892 | EIJ16482 | gram-negative pili assembly chaperone, N-terminal domain protein | Escherichia coli 900105 (10e) | 660.5 | 6.50E-195 |
| FimC | 471 | G874_04775 | EQV16306 | chaperone fimC | Escherichia coli HVH 223 (4-2976528) | 660.5 | 6.50E-195 |
| FimC | 472 | ECUMN_4923 | CAR16034 | chaperone, periplasmic | Escherichia coli UMN026 | 660.5 | 6.50E-195 |
| FimC | 473 | AC21_4524 | KDY88116 | chaperone protein fimC | Escherichia coli 2-474-04_S3_C2 | 660.5 | 6.50E-195 |
| FimC | 474 | ESBG_03749 | EIG72444 | chaperone fimC | Escherichia sp. 4_1_40B | 660.5 | 6.50E-195 |
| FimC | 475 | G833_04630 | EQT22134 | chaperone fimC | Escherichia coli HVH 180 (4-3051617) | 660.5 | 6.50E-195 |
| FimC | 476 | WIK_04760 | ELI36504 | fimbrial chaperone FimC | Escherichia coli KTE122 | 660.5 | 6.50E-195 |
| FimC | 477 | EC2762100_5079 | EMW48603 | chaperone protein fimC | Escherichia coli 2762100 | 660.5 | 6.50E-195 |
| FimC | 478 | G698_04635 | EQN74778 | chaperone fimC | Escherichia coli HVH 22 (4-2258986) | 660.5 | 6.50E-195 |
| FimC | 479 | HMPREF9552_00199 | EFJ76115 | gram-negative pili assembly chaperone domain protein | Escherichia coli MS 198-1 | 660.5 | 6.50E-195 |
| FimC | 480 | EH66_19690 | KEO95254 | molecular chaperone FimC | Escherichia coli HVH 70 (4-2963531) | 660.5 | 6.50E-195 |
| FimC | 481 | CFSAN001629_05895 | EKU02271 | type I fimbrial chaperone | Escherichia coli O26:H11 str. CFSAN001629 | 660.5 | 6.50E-195 |
| FimC | 482 | AB25_4926 | EZK26042 | chaperone protein fimC | Escherichia coli 2-005-03_S1_C2 | 660.5 | 6.50E-195 |
| FimC | 483 | G692_04617 | EQN42728 | chaperone fimC | Escherichia coli HVH 16 (4-7649002) | 660.5 | 6.50E-195 |
| FimC | 484 | UWO_18510 | EIF16574 | chaperone FimC, periplasmic | Escherichia coli O32:H37 str. P4 | 660.5 | 6.50E-195 |
| FimC | 485 | A17G_00028 | EOV45860 | fimbrial chaperone FimC | Escherichia coli KTE221 | 660.5 | 6.50E-195 |
| FimC | 486 | AB48_3878 | KEK80755 | chaperone protein fimC | Escherichia coli 3-475-03_S1_C2 | 660.5 | 6.50E-195 |
| FimC | 487 | AB63_4286 | KDX77102 | chaperone protein fimC | Escherichia coli 2-222-05_S1_C3 | 660.5 | 6.50E-195 |
| FimC | 488 | PPECC33_04816 | AKK51304 | chaperone, periplasmic | Escherichia coli PCN033 | 660.5 | 6.50E-195 |
| FimC | 489 | AD22_4683 | KEN57254 | chaperone protein fimC | Escherichia coli 6-537-08_S4_C2 | 660.5 | 6.50E-195 |
| FimC | 490 | G855_04535 | EQU29467 | chaperone fimC | Escherichia coli HVH 203 (4-3126218) | 660.5 | 6.50E-195 |
| FimC | 491 | AA97_5004 | EZK35651 | chaperone protein fimC | Escherichia coli 2-005-03_S1_C1 | 660.5 | 6.50E-195 |
| FimC | 492 | G763_00440 | EQQ43926 | chaperone fimC | Escherichia coli HVH 102 (4-6906788) | 660.5 | 6.50E-195 |
| FimC | 493 | G851_04735 | EQU06188 | chaperone fimC | Escherichia coli HVH 199 (4-5670322) | 660.5 | 6.50E-195 |
| FimC | 494 | G918_04488 | ERB12097 | chaperone fimC | Escherichia coli UMEA 3150-1 | 660.5 | 6.50E-195 |
| FimC | 495 | WEK_00143 | ELC51174 | chaperone fimC | Escherichia coli KTE26 | 660.5 | 6.50E-195 |
| FimC | 496 | ECDEC7B_4716 | EHV88190 | gram-negative pili assembly chaperone, N-terminal domain protein | Escherichia coli DEC7B | 660.5 | 6.50E-195 |
| FimC | 497 | G720_04960 | EQO74522 | chaperone fimC | Escherichia coli HVH 45 (4-3129918) | 660.5 | 6.50E-195 |
| FimC | 498 | ECP029970676_5108 | ENC26697 | chaperone protein fimC | Escherichia coli P02997067.6 | 660.5 | 6.50E-195 |
| FimC | 499 | G853_04727 | EQU18638 | chaperone fimC | Escherichia coli HVH 201 (4-4459431) | 660.5 | 6.50E-195 |
| FimC | 500 | HMPREF1618_04153 | ESE15046 | gram-negative pili assembly chaperone domain protein | Escherichia coli 908691 | 660.5 | 6.50E-195 |
| FimC | 501 | WKM_04409 | ELJ20334 | fimbrial chaperone FimC | Escherichia coli KTE167 | 660.5 | 6.50E-195 |
| FimC | 502 | G984_04775 | EQZ48776 | chaperone fimC | Escherichia coli UMEA 3662-1 | 660.5 | 6.50E-195 |
| FimC | 503 | AD42_4134 | KDZ79731 | chaperone protein fimC | Escherichia coli 3-073-06_S4_C3 | 660.5 | 6.50E-195 |
| FimC | 504 | L404_04793 | ETY47961 | chaperone fimC | Escherichia coli BWH 34 | 660.5 | 6.50E-195 |
| FimC | 505 | BY44_19130 | EZA74592 | molecular chaperone FimC | Escherichia coli O6:H16 str. F5656C1 | 660.5 | 6.50E-195 |
| FimC | 506 | G765_05044 | EQQ45650 | chaperone fimC | Escherichia coli HVH 104 (4-6977960) | 660.5 | 6.50E-195 |
| FimC | 507 | A15W_00406 | ELH78503 | fimbrial chaperone FimC | Escherichia coli KTE211 | 660.5 | 6.50E-195 |
| FimC | 508 | AE51_04130 | KDG24973 | chaperone fimC | Escherichia coli BIDMC 76 | 660.5 | 6.50E-195 |
| FimC | 509 | AA99_4993 | KDA60912 | chaperone protein fimC | Escherichia coli 2-052-05_S1_C1 | 660.5 | 6.50E-195 |
| FimC | 510 | AD19_4388 | KEL06088 | chaperone protein fimC | Escherichia coli 4-203-08_S4_C2 | 660.5 | 6.50E-195 |
| FimC | 511 | EC40967_5581 | EII33548 | gram-negative pili assembly chaperone, N-terminal domain protein | Escherichia coli 4.0967 | 660.5 | 6.50E-195 |
| FimC | 512 | BX11_20555 | EZE27641 | molecular chaperone FimC | Escherichia coli O123:H11 str. 2009C-3307 | 660.5 | 6.50E-195 |
| FimC | 513 | AD13_4996 | KDZ36766 | chaperone protein fimC | Escherichia coli 3-020-07_S4_C2 | 660.5 | 6.50E-195 |
| FimC | 514 | ESAG_03940 | EEH88228 | chaperone fimC | Escherichia sp. 3_2_53FAA | 660.5 | 6.50E-195 |
| FimC | 515 | AB99_4813 | EZJ86792 | chaperone protein fimC | Escherichia coli 1-182-04_S3_C1 | 660.5 | 6.50E-195 |
| FimC | 516 | G891_04592 | EQV75290 | chaperone fimC | Escherichia coli KOEGE 68 (182a) | 660.5 | 6.50E-195 |
| FimC | 517 | G906_05084 | EQW54027 | chaperone fimC | Escherichia coli UMEA 3088-1 | 660.5 | 6.50E-195 |
| FimC | 518 | AE39_03812 | KDF87821 | chaperone fimC | Escherichia coli BIDMC 64 | 660.5 | 6.50E-195 |
| FimC | 519 | WGY_04662 | ELJ77905 | fimbrial chaperone FimC | Escherichia coli KTE95 | 660.5 | 6.50E-195 |
| FimC | 520 | AC49_4700 | KDY57205 | chaperone protein fimC | Escherichia coli 2-460-02_S3_C3 | 660.5 | 6.50E-195 |
| FimC | 521 | A1U3_04659 | ELG21216 | fimbrial chaperone FimC | Escherichia coli KTE65 | 660.5 | 6.50E-195 |
| FimC | 522 | A1YA_02096 | ELG61912 | fimbrial chaperone FimC | Escherichia coli KTE123 | 660.5 | 6.50E-195 |
| FimC | 523 | G926_04500 | EQX26745 | chaperone fimC | Escherichia coli UMEA 3163-1 | 660.5 | 6.50E-195 |
| FimC | 524 | ECCZ_01860 | EST65318 | chaperone FimC, periplasmic | Escherichia coli ECC-Z | 660.5 | 6.50E-195 |
| FimC | 525 | L446_09024 | ETY13461 | chaperone fimC | Escherichia coli BIDMC 17A | 660.5 | 6.50E-195 |
| FimC | 526 | BW79_12305 | EYZ99698 | molecular chaperone FimC | Escherichia coli O119:H4 str. 03-3458 | 660.5 | 6.50E-195 |
| FimC | 527 | AC64_4802 | KEM79683 | chaperone protein fimC | Escherichia coli 6-537-08_S3_C3 | 660.5 | 6.50E-195 |
| FimC | 528 | A1SY_00607 | ELG19916 | fimbrial chaperone FimC | Escherichia coli KTE63 | 660.5 | 6.50E-195 |
| FimC | 529 | P803_04623 | ETX82098 | chaperone fimC | Escherichia coli BIDMC 43a | 660.5 | 6.50E-195 |
| FimC | 530 | EC23916_5646 | EII45227 | gram-negative pili assembly chaperone, N-terminal domain protein | Escherichia coli 2.3916 | 660.5 | 6.50E-195 |
| FimC | 531 | WII_04739 | ELI33517 | fimbrial chaperone FimC | Escherichia coli KTE120 | 660.5 | 6.50E-195 |
| FimC | 532 | G800_04595 | EQS11024 | chaperone fimC | Escherichia coli HVH 142 (4-5627451) | 660.5 | 6.50E-195 |
| FimC | 533 | ECEPECA14_0513 | EFZ43765 | chaperone protein fimC | Escherichia coli EPECa14 | 660.5 | 6.50E-195 |
| FimC | 534 | AC44_0026 | KEO17097 | chaperone protein fimC | Escherichia coli 2-177-06_S3_C3 | 660.5 | 6.50E-195 |
| FimC | 535 | A15K_04821 | ELD04933 | chaperone fimC | Escherichia coli KTE205 | 660.5 | 6.50E-195 |
| FimC | 536 | ESRG_01611 | EHN87313 | chaperone fimC | Escherichia coli TA124 | 660.5 | 6.50E-195 |
| FimC | 537 | G733_04581 | EQP27807 | chaperone fimC | Escherichia coli HVH 65 (4-2262045) | 660.5 | 6.50E-195 |
| FimC | 538 | G939_03665 | EQX93234 | chaperone fimC | Escherichia coli UMEA 3201-1 | 660.5 | 6.50E-195 |
| FimC | 539 | AC86_4264 | KDU34568 | chaperone protein fimC | Escherichia coli 3-073-06_S4_C1 | 660.5 | 6.50E-195 |
| FimC | 540 | AE47_03891 | KDG05528 | chaperone fimC | Escherichia coli BIDMC 72 | 660.5 | 6.50E-195 |
| FimC | 541 | BX75_23385 | EYY76124 | molecular chaperone FimC | Escherichia coli O26:NM str. 2010C-4788 | 660.5 | 6.50E-195 |
| FimC | 542 | ECP03022932_5000 | EMX08791 | chaperone protein fimC | Escherichia coli P0302293.2 | 660.5 | 6.50E-195 |
| FimC | 543 | G696_04612 | EQN58014 | chaperone fimC | Escherichia coli HVH 20 (4-5865042) | 660.5 | 6.50E-195 |
| FimC | 544 | BW97_06250 | EYX08971 | molecular chaperone FimC | Escherichia coli O69:H11 str. 07-4281 | 660.5 | 6.50E-195 |
| FimC | 545 | HMPREF9530_03168 | EFK20222 | gram-negative pili assembly chaperone domain protein | Escherichia coli MS 21-1 | 660.5 | 6.50E-195 |
| FimC | 546 | A1Y3_00658 | ELF04240 | chaperone fimC | Escherichia coli KTE116 | 660.5 | 6.50E-195 |
| FimC | 547 | AC65_3933 | KDW72389 | chaperone protein fimC | Escherichia coli 2-005-03_S4_C1 | 660.5 | 6.50E-195 |
| FimC | 548 | A139_04426 | ELC68645 | chaperone fimC | Escherichia coli KTE181 | 660.5 | 6.50E-195 |
| FimC | 549 | ECDEC10D_5643 | EHW71889 | gram-negative pili assembly chaperone, N-terminal domain protein | Escherichia coli DEC10D | 660.5 | 6.50E-195 |
| FimC | 550 | ESPG_02752 | EHN96213 | chaperone fimC | Escherichia coli H397 | 660.5 | 6.50E-195 |
| FimC | 551 | AD20_4586 | KEL99592 | chaperone protein fimC | Escherichia coli 6-175-07_S4_C2 | 660.5 | 6.50E-195 |
| FimC | 552 | AC69_4763 | KDX45981 | chaperone protein fimC | Escherichia coli 2-177-06_S4_C1 | 660.5 | 6.50E-195 |
| FimC | 553 | L436_08813 | ETY23983 | chaperone fimC | Escherichia coli BIDMC 9 | 660.5 | 6.50E-195 |
| FimC | 554 | ECC69171_18590 | KIG25318 | molecular chaperone FimC | Escherichia coli C691-71 (14b) | 660.5 | 6.50E-195 |
| FimC | 555 | AE33_04524 | KDF70478 | chaperone fimC | Escherichia coli BIDMC 58 | 660.5 | 6.50E-195 |
| FimC | 556 | G945_04479 | EQY17068 | chaperone fimC | Escherichia coli UMEA 3216-1 | 660.5 | 6.50E-195 |
| FimC | 557 | G736_04971 | EQP42915 | chaperone fimC | Escherichia coli HVH 70 (4-2963531) | 660.5 | 6.50E-195 |
| FimC | 558 | ECDEC5E_5463 | EHV41951 | chaperone protein fimC | Escherichia coli DEC5E | 660.5 | 6.50E-195 |
| FimC | 559 | ECE128010_0498 | EFZ49145 | chaperone protein fimC | Escherichia coli E128010 | 660.5 | 6.50E-195 |
| FimC | 560 | WI7_04513 | ELI02046 | fimbrial chaperone FimC | Escherichia coli KTE105 | 660.5 | 6.50E-195 |
| FimC | 561 | G722_04494 | EQO79517 | chaperone fimC | Escherichia coli HVH 48 (4-2658593) | 660.5 | 6.50E-195 |
| FimC | 562 | ECO10026_29384 | EIL44111 | hypothetical protein | Escherichia coli O26:H11 str. CVM10026 | 660.5 | 6.50E-195 |
| FimC | 563 | G882_04586 | EQV36554 | chaperone fimC | Escherichia coli KOEGE 32 (66a) | 660.5 | 6.50E-195 |
| FimC | 564 | AC31_3574 | KDZ63214 | chaperone protein fimC | Escherichia coli 3-073-06_S3_C2 | 660.5 | 6.50E-195 |
| FimC | 565 | G914_04744 | EQW85486 | chaperone fimC | Escherichia coli UMEA 3139-1 | 660.5 | 6.50E-195 |
| FimC | 566 | ECMP02155212_0267 | EMU72103 | chaperone protein fimC | Escherichia coli MP021552.12 | 660.5 | 6.50E-195 |
| FimC | 567 | A17E_04402 | ELD45369 | chaperone fimC | Escherichia coli KTE220 | 660.5 | 6.50E-195 |
| FimC | 568 | WCC_00157 | ELC03553 | chaperone fimC | Escherichia coli KTE4 | 660.5 | 6.50E-195 |
| FimC | 569 | UMNF18_5380 | AEJ59808 | chaperone protein fimC | Escherichia coli UMNF18 | 660.5 | 6.50E-195 |
| FimC | 570 | AC28_4934 | KEO24623 | chaperone protein fimC | Escherichia coli 1-250-04_S3_C2 | 660.5 | 6.50E-195 |
| FimC | 571 | AF55_03222 | EZQ66576 | chaperone fimC | Escherichia coli BIDMC 82 | 660.5 | 6.50E-195 |
| FimC | 572 | AD45_4270 | KDU65134 | chaperone protein fimC | Escherichia coli 4-203-08_S4_C3 | 660.5 | 6.50E-195 |
| FimC | 573 | AB70_5091 | EZK04050 | chaperone protein fimC | Escherichia coli 1-176-05_S1_C3 | 660.5 | 6.50E-195 |
| FimC | 574 | A17I_01812 | EOV39914 | fimbrial chaperone FimC | Escherichia coli KTE222 | 660.5 | 6.50E-195 |
| FimC | 575 | BX65_19290 | EYZ12837 | molecular chaperone FimC | Escherichia coli O103:H2 str. 2010C-4433 | 660.5 | 6.50E-195 |
| FimC | 576 | A157_00355 | EOV31518 | fimbrial chaperone FimC | Escherichia coli KTE198 | 660.5 | 6.50E-195 |
| FimC | 577 | AC92_4728 | KEM87054 | chaperone protein fimC | Escherichia coli 6-537-08_S4_C1 | 660.5 | 6.50E-195 |
| FimC | 578 | G735_04624 | EQP30898 | chaperone fimC | Escherichia coli HVH 69 (4-2837072) | 660.5 | 6.50E-195 |
| FimC | 579 | AC99_2572 | KDX91044 | chaperone protein fimC | Escherichia coli 2-222-05_S4_C2 | 660.5 | 6.50E-195 |
| FimC | 580 | V412_07095 | ETE33668 | molecular chaperone FimC | Escherichia coli LAU-EC7 | 660.5 | 6.50E-195 |
| FimC | 581 | ECBD_3719 | ACT30712 | Pili assembly chaperone, N-terminal | Escherichia coli 'BL21-Gold(DE3)pLysS AG' | 660.5 | 6.50E-195 |
| FimC | 582 | G846_04698 | EQT91086 | chaperone fimC | Escherichia coli HVH 194 (4-2356805) | 660.5 | 6.50E-195 |
| FimC | 583 | WAU_00555 | EOU41307 | fimbrial chaperone FimC | Escherichia coli KTE3 | 660.5 | 6.50E-195 |
| FimC | 584 | G791_04734 | EQR69083 | chaperone fimC | Escherichia coli HVH 133 (4-4466519) | 660.5 | 6.50E-195 |
| FimC | 585 | A1Y5_00865 | ELG57357 | fimbrial chaperone FimC | Escherichia coli KTE118 | 660.5 | 6.50E-195 |
| FimC | 586 | L455_09101 | ETX90598 | chaperone fimC | Escherichia coli BIDMC 20A | 660.5 | 6.50E-195 |
| FimC | 587 | AE54_03980 | KDG42468 | chaperone fimC | Escherichia coli BIDMC 79 | 660.5 | 6.50E-195 |
| FimC | 588 | AF24_03972 | KDG47795 | chaperone fimC | Escherichia coli CHS 68 | 660.5 | 6.50E-195 |
| FimC | 589 | AC53_4781 | KEM96801 | chaperone protein fimC | Escherichia coli 7-233-03_S3_C3 | 660.5 | 6.50E-195 |
| FimC | 590 | G943_04911 | EQY08753 | chaperone fimC | Escherichia coli UMEA 3212-1 | 660.5 | 6.50E-195 |
| FimC | 591 | WCA_00521 | ELC04742 | chaperone fimC | Escherichia coli KTE2 | 660.5 | 6.50E-195 |
| FimC | 592 | AC08_4299 | KEL11547 | chaperone protein fimC | Escherichia coli 4-203-08_S3_C1 | 660.5 | 6.50E-195 |
| FimC | 593 | ECVR50_4790 | AKA93482 | chaperone protein FimC | Escherichia coli VR50 | 660.5 | 6.50E-195 |
| FimC | 594 | AB55_4898 | KDT09454 | chaperone protein fimC | Escherichia coli 2-052-05_S1_C3 | 660.5 | 6.50E-195 |
| FimC | 595 | G838_04310 | EQT51892 | chaperone fimC | Escherichia coli HVH 186 (4-3405044) | 660.5 | 6.50E-195 |
| FimC | 596 | ECDEC6E_5030 | EHV68519 | gram-negative pili assembly chaperone, N-terminal domain protein | Escherichia coli DEC6E | 660.5 | 6.50E-195 |
| FimC | 597 | P810_03282 | ETY55488 | chaperone fimC | Escherichia coli BIDMC 49a | 660.5 | 6.50E-195 |
| FimC | 598 | EC958_0047 | CDN85264 | type 1 fimbriae periplasmic chaperone protein FimC | Escherichia coli O25b:H4-ST131 | 660.5 | 6.50E-195 |
| FimC | 599 | ECTW15901_4876 | EKI14221 | type I fimbrial chaperone | Escherichia coli TW15901 | 660.5 | 6.50E-195 |
| FimC | 600 | AC50_3936 | KDZ14047 | chaperone protein fimC | Escherichia coli 2-474-04_S3_C3 | 660.5 | 6.50E-195 |
| FimC | 601 | EC80569_4587 | EKK39130 | chaperone protein fimC | Escherichia coli 8.0569 | 660.5 | 6.50E-195 |
| FimC | 602 | WCS_04860 | EOU53465 | fimbrial chaperone FimC | Escherichia coli KTE14 | 660.5 | 6.50E-195 |
| FimC | 603 | A1SG_01142 | ELG10037 | fimbrial chaperone FimC | Escherichia coli KTE54 | 660.5 | 6.50E-195 |
| FimC | 604 | A1YG_00371 | EOW53820 | fimbrial chaperone FimC | Escherichia coli KTE130 | 660.5 | 6.50E-195 |
| FimC | 605 | AC61_4371 | KEK93164 | chaperone protein fimC | Escherichia coli 4-203-08_S3_C3 | 660.5 | 6.50E-195 |
| FimC | 606 | G710_04746 | EQO25393 | chaperone fimC | Escherichia coli HVH 35 (4-2962667) | 660.5 | 6.50E-195 |
| FimC | 607 | G830_04540 | EQT17809 | chaperone fimC | Escherichia coli HVH 176 (4-3428664) | 660.5 | 6.50E-195 |
| FimC | 608 | G705_04738 | EQO01884 | chaperone fimC | Escherichia coli HVH 29 (4-3418073) | 660.5 | 6.50E-195 |
| FimC | 609 | EC80566_4597 | EKK38125 | type I fimbrial chaperone | Escherichia coli 8.0566 | 660.5 | 6.50E-195 |
| FimC | 610 | ECDEC8D_0004 | EHW31960 | gram-negative pili assembly chaperone, N-terminal domain protein | Escherichia coli DEC8D | 660.5 | 6.50E-195 |
| FimC | 611 | A1SQ_00457 | ELG19617 | fimbrial chaperone FimC | Escherichia coli KTE59 | 660.5 | 6.50E-195 |
| FimC | 612 | BX66_13210 | EYZ24232 | molecular chaperone FimC | Escherichia coli O103:H25 str. 2010C-4529 | 660.5 | 6.50E-195 |
| FimC | 613 | HMPREF1589_00469 | ESA76441 | gram-negative pili assembly chaperone domain protein | Escherichia coli 113290 | 660.5 | 6.50E-195 |
| FimC | 614 | A17C_04561 | EOV29742 | fimbrial chaperone FimC | Escherichia coli KTE219 | 660.5 | 6.50E-195 |
| FimC | 615 | HMPREF1597_03582 | ESD18455 | gram-negative pili assembly chaperone domain protein | Escherichia coli 907701 | 660.5 | 6.50E-195 |
| FimC | 616 | G873_04540 | EQV07676 | chaperone fimC | Escherichia coli HVH 222 (4-2977443) | 660.5 | 6.50E-195 |
| FimC | 617 | G835_04893 | EQT33063 | chaperone fimC | Escherichia coli HVH 183 (4-3205932) | 660.5 | 6.50E-195 |
| FimC | 618 | EC2846750_4878 | EMZ59735 | chaperone protein fimC | Escherichia coli 2846750 | 660.5 | 6.50E-195 |
| FimC | 619 | AD28_4476 | KDX61197 | chaperone protein fimC | Escherichia coli 2-210-07_S4_C3 | 660.5 | 6.50E-195 |
| FimC | 620 | AB39_4829 | EZK13510 | chaperone protein fimC | Escherichia coli 1-176-05_S1_C2 | 660.5 | 6.50E-195 |
| FimC | 621 | ECP03018673_4957 | ENG93187 | chaperone protein fimC | Escherichia coli P0301867.3 | 660.5 | 6.50E-195 |
| FimC | 622 | DR76_2427 | AIL16079 | chaperone protein fimC | Escherichia coli ATCC 25922 | 660.5 | 6.50E-195 |
| FimC | 623 | G865_04975 | EQU75557 | chaperone fimC | Escherichia coli HVH 213 (4-3042928) | 660.5 | 6.50E-195 |
| FimC | 624 | A1SA_00491 | ELE06259 | chaperone fimC | Escherichia coli KTE51 | 660.5 | 6.50E-195 |
| FimC | 625 | G730_04642 | EQP09550 | chaperone fimC | Escherichia coli HVH 59 (4-1119338) | 660.5 | 6.50E-195 |
| FimC | 626 | G829_04841 | EQT17399 | chaperone fimC | Escherichia coli HVH 175 (4-3405184) | 660.5 | 6.50E-195 |
| FimC | 627 | A31O_00464 | EOW72646 | fimbrial chaperone FimC | Escherichia coli KTE170 | 660.5 | 6.50E-195 |
| FimC | 628 | L447_04578 | ETY13676 | chaperone fimC | Escherichia coli BIDMC 17B | 660.5 | 6.50E-195 |
| FimC | 629 | G784_04614 | EQR29806 | chaperone fimC | Escherichia coli HVH 122 (4-6851606) | 660.5 | 6.50E-195 |
| FimC | 630 | ECDEC14C_5176 | EHX82513 | gram-negative pili assembly chaperone, N-terminal domain protein | Escherichia coli DEC14C | 660.5 | 6.50E-195 |
| FimC | 631 | AB86_4910 | KDW11731 | chaperone protein fimC | Escherichia coli 2-177-06_S3_C1 | 660.5 | 6.50E-195 |
| FimC | 632 | AF44_03472 | KDG67735 | chaperone fimC | Escherichia coli MGH 58 | 660.5 | 6.50E-195 |
| FimC | 633 | WEO_04652 | ELC45410 | chaperone fimC | Escherichia coli KTE28 | 660.5 | 6.50E-195 |
| FimC | 634 | AC36_4328 | KEL07182 | chaperone protein fimC | Escherichia coli 4-203-08_S3_C2 | 660.5 | 6.50E-195 |
| FimC | 635 | ECHM605_02310 | EIL82353 | chaperone FimC, periplasmic | Escherichia coli HM605 | 660.5 | 6.50E-195 |
| FimC | 636 | AC82_4866 | EZJ57390 | chaperone protein fimC | Escherichia coli 1-182-04_S4_C1 | 660.5 | 6.50E-195 |
| FimC | 637 | AD39_4950 | EZJ13421 | chaperone protein fimC | Escherichia coli 1-182-04_S4_C3 | 660.5 | 6.50E-195 |
| FimC | 638 | AC02_3801 | KDZ36717 | chaperone protein fimC | Escherichia coli 3-020-07_S3_C1 | 660.5 | 6.50E-195 |
| FimC | 639 | ECO9450_23104 | EIL00502 | chaperone FimC, periplasmic | Escherichia coli O103:H2 str. CVM9450 | 660.5 | 6.50E-195 |
| FimC | 640 | G797_04418 | EQR89490 | chaperone fimC | Escherichia coli HVH 139 (4-3192644) | 660.5 | 6.50E-195 |
| FimC | 641 | ECOLIN_24005 | AID81489 | molecular chaperone FimC | Escherichia coli Nissle 1917 | 660.5 | 6.50E-195 |
| FimC | 642 | G978_04650 | EQZ28304 | chaperone fimC | Escherichia coli UMEA 3592-1 | 660.5 | 6.50E-195 |
| FimC | 643 | AC11_4757 | KEM71258 | chaperone protein fimC | Escherichia coli 6-537-08_S3_C1 | 660.5 | 6.50E-195 |
| FimC | 644 | AE45_04382 | KDF95942 | chaperone fimC | Escherichia coli BIDMC 70 | 660.5 | 6.50E-195 |
| FimC | 645 | CFSAN002237_19495 | ERF89648 | molecular chaperone FimC | Escherichia coli O104:H21 str. CFSAN002237 | 660.5 | 6.50E-195 |
| FimC | 646 | ECDEC7A_0003 | EHV81989 | chaperone protein fimC | Escherichia coli DEC7A | 660.5 | 6.50E-195 |
| FimC | 647 | G994_04614 | EQZ94023 | chaperone fimC | Escherichia coli UMEA 3718-1 | 660.5 | 6.50E-195 |
| FimC | 648 | G768_04916 | EQQ62543 | chaperone fimC | Escherichia coli HVH 107 (4-5860571) | 660.5 | 6.50E-195 |
| FimC | 649 | A1W1_04839 | ELE76384 | chaperone fimC | Escherichia coli KTE83 | 660.5 | 6.50E-195 |
| FimC | 650 | G798_04755 | EQR98237 | chaperone fimC | Escherichia coli HVH 140 (4-5894387) | 660.5 | 6.50E-195 |
| FimC | 651 | ECDG_02700 | EFF03497 | fimbrial chaperone | Escherichia coli B185 | 660.5 | 6.50E-195 |
| FimC | 652 | BW85_22975 | EYZ76903 | molecular chaperone FimC | Escherichia coli O69:H11 str. 06-3325 | 660.5 | 6.50E-195 |
| FimC | 653 | G927_04627 | EQX28039 | chaperone fimC | Escherichia coli UMEA 3172-1 | 660.5 | 6.50E-195 |
| FimC | 654 | A1S7_00578 | ELE01011 | chaperone fimC | Escherichia coli KTE49 | 660.5 | 6.50E-195 |
| FimC | 655 | CDL05891 | CDL05891 | chaperone FimC | Escherichia coli IS35 | 660.5 | 6.50E-195 |
| FimC | 656 | AC32_4511 | KDT44940 | chaperone protein fimC | Escherichia coli 3-105-05_S3_C2 | 660.5 | 6.50E-195 |
| FimC | 657 | G744_00124 | EQP94290 | chaperone fimC | Escherichia coli HVH 82 (4-2209276) | 660.5 | 6.50E-195 |
| FimC | 658 | BY39_21620 | EZA65550 | molecular chaperone FimC | Escherichia coli O104:H21 str. 94-3025 | 660.5 | 6.50E-195 |
| FimC | 659 | G915_04340 | EQW93089 | chaperone fimC | Escherichia coli UMEA 3140-1 | 660.5 | 6.50E-195 |
| FimC | 660 | BW84_24110 | EYZ85333 | molecular chaperone FimC | Escherichia coli O118:H16 str. 06-3256 | 660.5 | 6.50E-195 |
| FimC | 661 | AC39_4863 | KEN13307 | chaperone protein fimC | Escherichia coli 6-537-08_S3_C2 | 660.5 | 6.50E-195 |
| FimC | 662 | ECRM13516_5274 | AHG17855 | chaperone FimC | Escherichia coli O145:H28 str. RM13516 | 660.5 | 6.50E-195 |
| FimC | 663 | HMPREF1593_03578 | ESC95015 | gram-negative pili assembly chaperone domain protein | Escherichia coli 907391 | 660.5 | 6.50E-195 |
| FimC | 664 | G863_04697 | EQU65343 | chaperone fimC | Escherichia coli HVH 211 (4-3041891) | 660.5 | 6.50E-195 |
| FimC | 665 | ECDEC12A_0035 | EHX37055 | chaperone protein fimC | Escherichia coli DEC12A | 660.5 | 6.50E-195 |
| FimC | 666 | ECEPECA12_5171 | EIQ57954 | chaperone protein fimC | Escherichia coli EPECa12 | 660.5 | 6.50E-195 |
| FimC | 667 | BY04_19105 | EYX86419 | molecular chaperone FimC | Escherichia coli O156:H25 str. 2011C-3602 | 660.5 | 6.50E-195 |
| FimC | 668 | ERFG_03554 | EGB70715 | gram-negative pili assembly chaperone domain-containing protein | Escherichia coli TW10509 | 660.5 | 6.50E-195 |
| FimC | 669 | ECMP0215528_5213 | EMX32752 | chaperone protein fimC | Escherichia coli MP021552.8 | 660.5 | 6.50E-195 |
| FimC | 670 | AD33_3420 | KDZ11605 | chaperone protein fimC | Escherichia coli 2-474-04_S4_C3 | 660.5 | 6.50E-195 |
| FimC | 671 | G823_04744 | EQS83505 | chaperone fimC | Escherichia coli HVH 167 (4-6073565) | 660.5 | 6.50E-195 |
| FimC | 672 | ECEG_02929 | EFF14734 | chaperone FimC | Escherichia coli B354 | 660.5 | 6.50E-195 |
| FimC | 673 | ECDEC11C_5353 | EHX05906 | chaperone protein fimC | Escherichia coli DEC11C | 660.5 | 6.50E-195 |
| FimC | 674 | G828_04750 | EQT06491 | chaperone fimC | Escherichia coli HVH 173 (3-9175482) | 660.5 | 6.50E-195 |
| FimC | 675 | ECMP0215527_5001 | EMU56334 | chaperone protein fimC | Escherichia coli MP021552.7 | 660.5 | 6.50E-195 |
| FimC | 676 | A0259_03280 | AMR21720 | molecular chaperone FimC | Shigella sp. PAMC 28760 | 660.5 | 6.50E-195 |
| FimC | 677 | AD34_3935 | KEL59038 | chaperone protein fimC | Escherichia coli 5-172-05_S4_C3 | 660.5 | 6.50E-195 |
| FimC | 678 | ECB_04185 | ACT41811 | chaperone, periplasmic | Escherichia coli B str. REL606 | 660.5 | 6.50E-195 |
| FimC | 679 | WGO_04701 | ELJ63707 | fimbrial chaperone FimC | Escherichia coli KTE85 | 660.5 | 6.50E-195 |
| FimC | 680 | AE12_04073 | KDG73957 | chaperone fimC | Escherichia coli UCI 53 | 660.5 | 6.50E-195 |
| FimC | 681 | AD29_4701 | KEO10696 | chaperone protein fimC | Escherichia coli 2-222-05_S4_C3 | 660.5 | 6.50E-195 |
| FimC | 682 | ECDEC12E_0027 | EHX54708 | gram-negative pili assembly chaperone, N-terminal domain protein | Escherichia coli DEC12E | 660.5 | 6.50E-195 |
| FimC | 683 | BX00_00690 | EYX06769 | molecular chaperone FimC | Escherichia coli O118:H16 str. 08-3651 | 660.5 | 6.50E-195 |
| FimC | 684 | Q456_0219995 | ETJ57251 | molecular chaperone FimC | Escherichia coli ATCC BAA-2193 | 660.5 | 6.50E-195 |
| FimC | 685 | MOI_17829 | EST67520 | chaperone FimC, periplasmic | Escherichia coli P4-NR | 660.5 | 6.50E-195 |
| FimC | 686 | AD32_4452 | KDY73093 | chaperone protein fimC | Escherichia coli 2-460-02_S4_C3 | 660.5 | 6.50E-195 |
| FimC | 687 | G844_04751 | EQT77254 | chaperone fimC | Escherichia coli HVH 192 (4-3054470) | 660.5 | 6.50E-195 |
| FimC | 688 | PGA_01104 | CDU33091 | Chaperone FimC, periplasmic | Escherichia coli D6-113.11 | 660.5 | 6.50E-195 |
| FimC | 689 | AB78_2316 | KEK99691 | chaperone protein fimC | Escherichia coli 4-203-08_S1_C3 | 660.5 | 6.50E-195 |
| FimC | 690 | ECO103_5097 | BAI33776 | chaperone FimC, periplasmic | Escherichia coli O103:H2 str. 12009 | 660.5 | 6.50E-195 |
| FimC | 691 | G862_04592 | ERA85083 | chaperone fimC | Escherichia coli HVH 210 (4-3042480) | 660.5 | 6.50E-195 |
| FimC | 692 | G689_04589 | EQN36952 | chaperone fimC | Escherichia coli HVH 10 (4-6832164) | 660.5 | 6.50E-195 |
| FimC | 693 | EC1303_c45080 | AJF59121 | periplasmic chaperone | Escherichia coli 1303 | 660.5 | 6.50E-195 |
| FimC | 694 | AC55_0042 | EYE15616 | chaperone protein fimC | Escherichia coli 1-110-08_S3_C3 | 660.5 | 6.50E-195 |
| FimC | 695 | G793_04687 | EQR73360 | chaperone fimC | Escherichia coli HVH 135 (4-4449320) | 660.5 | 6.50E-195 |
| FimC | 696 | ECP030186711_4984 | ENC87491 | chaperone protein fimC | Escherichia coli P0301867.11 | 660.5 | 6.50E-195 |
| FimC | 697 | G985_04557 | EQZ59254 | chaperone fimC | Escherichia coli UMEA 3671-1 | 660.5 | 6.50E-195 |
| FimC | 698 | G912_04728 | EQW76250 | chaperone fimC | Escherichia coli UMEA 3122-1 | 660.5 | 6.50E-195 |
| FimC | 699 | AC58_3299 | KDU09485 | chaperone protein fimC | Escherichia coli 3-105-05_S3_C3 | 660.5 | 6.50E-195 |
| FimC | 700 | ECSTECMHI813_4803 | EGW99977 | chaperone protein fimC | Escherichia coli STEC_MHI813 | 660.5 | 6.50E-195 |
| FimC | 701 | AD06_0004 | KEN16707 | chaperone protein fimC | Escherichia coli 7-233-03_S4_C2 | 660.5 | 6.50E-195 |
| FimC | 702 | ECDEC11E_4989 | EHX14662 | chaperone protein fimC | Escherichia coli DEC11E | 660.5 | 6.50E-195 |
| FimC | 703 | BY08_12100 | EYX87042 | molecular chaperone FimC | Escherichia coli O103:H2 str. 2011C-3750 | 660.5 | 6.50E-195 |
| FimC | 704 | A17K_00422 | ELH94122 | fimbrial chaperone FimC | Escherichia coli KTE223 | 660.5 | 6.50E-195 |
| FimC | 705 | BY43_15440 | EZA75356 | molecular chaperone FimC | Escherichia coli O25:NM str. E2539C1 | 660.5 | 6.50E-195 |
| FimC | 706 | BX14_11925 | EZE22334 | molecular chaperone FimC | Escherichia coli O45:H2 str. 2009C-3686 | 660.5 | 6.50E-195 |
| FimC | 707 | WGE_00629 | ELF78991 | fimbrial chaperone FimC | Escherichia coli KTE42 | 660.5 | 6.50E-195 |
| FimC | 708 | A17O_01130 | EOX13531 | fimbrial chaperone FimC | Escherichia coli KTE225 | 660.5 | 6.50E-195 |
| FimC | 709 | L456_04790 | ETX87703 | chaperone fimC | Escherichia coli BIDMC 20B | 660.5 | 6.50E-195 |
| FimC | 710 | ECP03018675_5030 | ENG96463 | chaperone protein fimC | Escherichia coli P0301867.5 | 660.5 | 6.50E-195 |
| FimC | 711 | G938_04725 | EQX81940 | chaperone fimC | Escherichia coli UMEA 3200-1 | 660.5 | 6.50E-195 |
| FimC | 712 | BX23_02240 | EZE49150 | molecular chaperone FimC | Escherichia coli O118:H16 str. 2009C-4446 | 660.5 | 6.50E-195 |
| FimC | 713 | A153_00611 | ELH45194 | fimbrial chaperone FimC | Escherichia coli KTE196 | 660.5 | 6.50E-195 |
| FimC | 714 | A364_00215 | EMD15421 | type I fimbrial chaperone | Escherichia coli SEPT362 | 660.5 | 6.50E-195 |
| FimC | 715 | G840_04552 | EQT56810 | chaperone fimC | Escherichia coli HVH 188 (4-2356988) | 660.5 | 6.50E-195 |
| FimC | 716 | EC75_04596 | EIL70471 | chaperone FimC, periplasmic | Escherichia coli 75 | 660.5 | 6.50E-195 |
| FimC | 717 | BW68_15635 | EZA44179 | molecular chaperone FimC | Escherichia coli O26:H11 str. 05-3646 | 660.5 | 6.50E-195 |
| FimC | 718 | AC56_4852 | EZJ65528 | chaperone protein fimC | Escherichia coli 1-182-04_S3_C3 | 660.5 | 6.50E-195 |
| FimC | 719 | HMPREF1607_02897 | ESD56767 | gram-negative pili assembly chaperone domain protein | Escherichia coli 908524 | 660.5 | 6.50E-195 |
| FimC | 720 | AE10_04570 | KDG67511 | chaperone fimC | Escherichia coli UCI 51 | 660.5 | 6.50E-195 |
| FimC | 721 | A17U_03572 | ELD56441 | chaperone fimC | Escherichia coli KTE228 | 660.5 | 6.50E-195 |
| FimC | 722 | L475_04777 | ESL31877 | chaperone fimC | Escherichia coli BIDMC 38 | 660.5 | 6.50E-195 |
| FimC | 723 | A1W7_00270 | ELE93784 | chaperone fimC | Escherichia coli KTE87 | 660.5 | 6.50E-195 |
| FimC | 724 | BW74_09300 | EZA29640 | molecular chaperone FimC | Escherichia coli O45:H2 str. 01-3147 | 660.5 | 6.50E-195 |
| FimC | 725 | HMPREF1612_01608 | ESD92069 | gram-negative pili assembly chaperone domain protein | Escherichia coli 908585 | 660.5 | 6.50E-195 |
| FimC | 726 | G806_01561 | ESP34414 | chaperone fimC | Escherichia coli HVH 148 (4-3192490) | 660.5 | 6.50E-195 |
| FimC | 727 | AD41_2613 | KDZ50079 | chaperone protein fimC | Escherichia coli 3-020-07_S4_C3 | 660.5 | 6.50E-195 |
| FimC | 728 | EC32303_4954 | EII75233 | gram-negative pili assembly chaperone, N-terminal domain protein | Escherichia coli 3.2303 | 660.5 | 6.50E-195 |
| FimC | 729 | ECMG_03843 | EGI29011 | chaperone protein FimC | Escherichia coli TA143 | 660.5 | 6.50E-195 |
| FimC | 730 | EC32608_5492 | EIH54462 | gram-negative pili assembly chaperone, N-terminal domain protein | Escherichia coli 3.2608 | 660.5 | 6.50E-195 |
| FimC | 731 | G809_04592 | EQS43760 | chaperone fimC | Escherichia coli HVH 151 (4-5755573) | 660.5 | 6.50E-195 |
| FimC | 732 | G813_04740 | ERA63173 | chaperone fimC | Escherichia coli HVH 155 (4-4509048) | 660.5 | 6.50E-195 |
| FimC | 733 | A31C_00497 | ELH03246 | fimbrial chaperone FimC | Escherichia coli KTE158 | 660.5 | 6.50E-195 |
| FimC | 734 | AB31_4283 | KDX73007 | chaperone protein fimC | Escherichia coli 2-222-05_S1_C2 | 660.5 | 6.50E-195 |
| FimC | 735 | HMPREF1615_00168 | ESE12286 | gram-negative pili assembly chaperone domain protein | Escherichia coli 908632 | 660.5 | 6.50E-195 |
| FimC | 736 | C201_20569 | EMD02925 | type I fimbrial chaperone | Escherichia coli S17 | 660.5 | 6.50E-195 |
| FimC | 737 | G972_04696 | EQZ08504 | chaperone fimC | Escherichia coli UMEA 3355-1 | 660.5 | 6.50E-195 |
| FimC | 738 | AE37_04312 | KDF81704 | chaperone fimC | Escherichia coli BIDMC 62 | 660.5 | 6.50E-195 |
| FimC | 739 | AD16_0307 | KDU27782 | chaperone protein fimC | Escherichia coli 3-267-03_S4_C2 | 660.5 | 6.50E-195 |
| FimC | 740 | Q457_24070 | ETI72926 | molecular chaperone FimC | Escherichia coli ATCC BAA-2196 | 660.5 | 6.50E-195 |
| FimC | 741 | AE53_02595 | KDG37315 | chaperone fimC | Escherichia coli BIDMC 78 | 660.5 | 6.50E-195 |
| FimC | 742 | AC73_3875 | KDY40587 | chaperone protein fimC | Escherichia coli 2-427-07_S4_C1 | 660.5 | 6.50E-195 |
| FimC | 743 | A195_04378 | ELD74181 | chaperone fimC | Escherichia coli KTE235 | 660.5 | 6.50E-195 |
| FimC | 744 | A13Q_00335 | ELH29752 | fimbrial chaperone FimC | Escherichia coli KTE190 | 660.5 | 6.50E-195 |
| FimC | 745 | ECO9534_17247 | EIL17772 | chaperone FimC, periplasmic | Escherichia coli O111:H11 str. CVM9534 | 660.5 | 6.50E-195 |
| FimC | 746 | G967_04578 | EQY93836 | chaperone fimC | Escherichia coli UMEA 3329-1 | 660.5 | 6.50E-195 |
| FimC | 747 | AC98_4739 | KDX54071 | chaperone protein fimC | Escherichia coli 2-210-07_S4_C2 | 660.5 | 6.50E-195 |
| FimC | 748 | HMPREF9543_03377 | EFK89797 | gram-negative pili assembly chaperone domain protein | Escherichia coli MS 146-1 | 660.5 | 6.50E-195 |
| FimC | 749 | WEA_04457 | ELF87806 | fimbrial chaperone FimC | Escherichia coli KTE22 | 660.5 | 6.50E-195 |
| FimC | 750 | G685_00709 | EQN23176 | chaperone fimC | Escherichia coli HVH 5 (4-7148410) | 660.5 | 6.50E-195 |
| FimC | 751 | AB88_5072 | KEN84372 | chaperone protein fimC | Escherichia coli 2-222-05_S3_C1 | 660.5 | 6.50E-195 |
| FimC | 752 | CDK86541 | CDK86541 | chaperone FimC | Escherichia coli IS29 | 660.5 | 6.50E-195 |
| FimC | 753 | ERKG_02922 | EGB46472 | gram-negative pili assembly chaperone domain-containing protein | Escherichia coli H252 | 660.5 | 6.50E-195 |
| FimC | 754 | BX08_06510 | EZE07537 | molecular chaperone FimC | Escherichia coli O103:H2 str. 2009C-3279 | 660.5 | 6.50E-195 |
| FimC | 755 | WEY_00373 | EOV00579 | fimbrial chaperone FimC | Escherichia coli KTE34 | 660.5 | 6.50E-195 |
| FimC | 756 | AF25_04778 | KDG48317 | chaperone fimC | Escherichia coli CHS 69 | 660.5 | 6.50E-195 |
| FimC | 757 | G929_04683 | EQX46311 | chaperone fimC | Escherichia coli UMEA 3174-1 | 660.5 | 6.50E-195 |
| FimC | 758 | G788_04809 | EQR53845 | chaperone fimC | Escherichia coli HVH 128 (4-7030436) | 660.5 | 6.50E-195 |
| FimC | 759 | ERGG_03313 | EGB55825 | gram-negative pili assembly chaperone domain-containing protein | Escherichia coli H489 | 660.5 | 6.50E-195 |
| FimC | 760 | A31I_00057 | ELF33249 | chaperone fimC | Escherichia coli KTE162 | 660.5 | 6.50E-195 |
| FimC | 761 | BX63_10005 | EYU85233 | molecular chaperone FimC | Escherichia coli O26:NM str. 2010C-4347 | 660.5 | 6.50E-195 |
| FimC | 762 | BU54_25040 | KDV42704 | molecular chaperone FimC | Escherichia coli O45:H2 str. 2010C-4211 | 660.5 | 6.50E-195 |
| FimC | 763 | L342_2268 | ESS94097 | chaperone FimC | Escherichia coli CE516 | 660.5 | 6.50E-195 |
| FimC | 764 | G931_04476 | EQX50466 | chaperone fimC | Escherichia coli UMEA 3176-1 | 660.5 | 6.50E-195 |
| FimC | 765 | HMPREF1617_00764 | ESE21360 | gram-negative pili assembly chaperone domain protein | Escherichia coli 908675 | 660.5 | 6.50E-195 |
| FimC | 766 | G711_04698 | ESP06156 | chaperone fimC | Escherichia coli HVH 36 (4-5675286) | 660.5 | 6.50E-195 |
| FimC | 767 | BX26_16155 | EZQ32859 | molecular chaperone FimC | Escherichia coli O26:H1 str. 2009C-4747 | 660.5 | 6.50E-195 |
| FimC | 768 | AD24_4591 | KDT12170 | chaperone protein fimC | Escherichia coli 2-011-08_S4_C3 | 660.5 | 6.50E-195 |
| FimC | 769 | AB91_4668 | KDY50233 | chaperone protein fimC | Escherichia coli 2-460-02_S3_C1 | 660.5 | 6.50E-195 |
| FimC | 770 | A1SM_00308 | ELE26411 | chaperone fimC | Escherichia coli KTE57 | 660.5 | 6.50E-195 |
| FimC | 771 | ECDEC10C_0028 | EHW81643 | gram-negative pili assembly chaperone, N-terminal domain protein | Escherichia coli DEC10C | 660.5 | 6.50E-195 |
| FimC | 772 | ECO10224_00779 | EJE69708 | type I fimbrial chaperone | Escherichia coli O26:H11 str. CVM10224 | 660.5 | 6.50E-195 |
| FimC | 773 | WKE_00006 | ELJ18355 | fimbrial chaperone FimC | Escherichia coli KTE160 | 660.5 | 6.50E-195 |
| FimC | 774 | G767_04791 | EQQ54010 | chaperone fimC | Escherichia coli HVH 106 (4-6881831) | 660.5 | 6.50E-195 |
| FimC | 775 | EcB7A_1821 | EDV62513 | chaperone protein FimC | Escherichia coli B7A | 660.5 | 6.50E-195 |
| FimC | 776 | L452_05244 | ETY08710 | chaperone fimC | Escherichia coli BIDMC 19A | 660.5 | 6.50E-195 |
| FimC | 777 | G734_04757 | EQP29547 | chaperone fimC | Escherichia coli HVH 68 (4-0888028) | 660.5 | 6.50E-195 |
| FimC | 778 | A31G_01926 | ELF28449 | chaperone fimC | Escherichia coli KTE161 | 660.5 | 6.50E-195 |
| FimC | 779 | ECP03022937_4841 | END92775 | chaperone protein fimC | Escherichia coli P0302293.7 | 660.5 | 6.50E-195 |
| FimC | 780 | ECSF_4257 | BAI57797 | type I fimbrial chaperone | Escherichia coli SE15 | 660.5 | 6.50E-195 |
| FimC | 781 | CFSAN002236_23555 | ERF86996 | molecular chaperone FimC | Escherichia coli O104:H21 str. CFSAN002236 | 660.5 | 6.50E-195 |
| FimC | 782 | AC03_3857 | KDZ61414 | chaperone protein fimC | Escherichia coli 3-073-06_S3_C1 | 660.5 | 6.50E-195 |
| FimC | 783 | AE48_04579 | KDG09998 | chaperone fimC | Escherichia coli BIDMC 73 | 660.5 | 6.50E-195 |
| FimC | 784 | G792_04761 | EQR70503 | chaperone fimC | Escherichia coli HVH 134 (4-6073441) | 660.5 | 6.50E-195 |
| FimC | 785 | HMPREF1613_01497 | ESD92716 | gram-negative pili assembly chaperone domain protein | Escherichia coli 908616 | 660.5 | 6.50E-195 |
| FimC | 786 | G940_04728 | EQX94614 | chaperone fimC | Escherichia coli UMEA 3203-1 | 660.5 | 6.50E-195 |
| FimC | 787 | Q459_03860 | ETD59157 | molecular chaperone FimC | Escherichia coli ATCC BAA-2215 | 660.5 | 6.50E-195 |
| FimC | 788 | AB35_4114 | KDZ00012 | chaperone protein fimC | Escherichia coli 2-474-04_S1_C2 | 660.5 | 6.50E-195 |
| FimC | 789 | G986_04485 | EQZ59804 | chaperone fimC | Escherichia coli UMEA 3682-1 | 660.5 | 6.50E-195 |
| FimC | 790 | ECA727_15855 | EST77922 | chaperone FimC, periplasmic | Escherichia coli ECA-727 | 660.5 | 6.50E-195 |
| FimC | 791 | AC66_3045 | KDT04852 | chaperone protein fimC | Escherichia coli 2-011-08_S4_C1 | 660.5 | 6.50E-195 |
| FimC | 792 | AB61_4939 | KDW36415 | chaperone protein fimC | Escherichia coli 2-177-06_S1_C3 | 660.5 | 6.50E-195 |
| FimC | 793 | A177_00297 | ELD45794 | chaperone fimC | Escherichia coli KTE216 | 660.5 | 6.50E-195 |
| FimC | 794 | G808_04465 | EQS44525 | chaperone fimC | Escherichia coli HVH 150 (4-3258106) | 660.5 | 6.50E-195 |
| FimC | 795 | AB21_4538 | KDU59965 | chaperone protein fimC | Escherichia coli 4-203-08_S1_C1 | 660.5 | 6.50E-195 |
| FimC | 796 | WKI_00028 | ELJ30939 | fimbrial chaperone FimC | Escherichia coli KTE166 | 660.5 | 6.50E-195 |
| FimC | 797 | ECDEC12B_0035 | EHX37422 | gram-negative pili assembly chaperone, N-terminal domain protein | Escherichia coli DEC12B | 660.5 | 6.50E-195 |
| FimC | 798 | EC2726950_4845 | ENA46048 | chaperone protein fimC | Escherichia coli 2726950 | 660.5 | 6.50E-195 |
| FimC | 799 | G875_04711 | EQV20526 | chaperone fimC | Escherichia coli HVH 225 (4-1273116) | 660.5 | 6.50E-195 |
| FimC | 800 | ECDEC7D_0093 | EHV96582 | gram-negative pili assembly chaperone, N-terminal domain protein | Escherichia coli DEC7D | 660.5 | 6.50E-195 |
| FimC | 801 | AB44_4698 | KDZ57183 | chaperone protein fimC | Escherichia coli 3-073-06_S1_C2 | 660.5 | 6.50E-195 |
| FimC | 802 | AD46_4719 | KEM44881 | chaperone protein fimC | Escherichia coli 6-175-07_S4_C3 | 660.5 | 6.50E-195 |
| FimC | 803 | G781_04796 | EQR15622 | chaperone fimC | Escherichia coli HVH 119 (4-6879578) | 660.5 | 6.50E-195 |
| FimC | 804 | C4390_42480 | EMS03616 | chaperone, periplasmic | Escherichia coli O127:H27 str. C43/90 | 660.5 | 6.50E-195 |
| FimC | 805 | A1SW_00445 | ELE36893 | chaperone fimC | Escherichia coli KTE62 | 660.5 | 6.50E-195 |
| FimC | 806 | HMPREF1601_01784 | ESA90737 | gram-negative pili assembly chaperone domain protein | Escherichia coli 907779 | 660.5 | 6.50E-195 |
| FimC | 807 | P804_03284 | ETX76948 | chaperone fimC | Escherichia coli BIDMC 43b | 660.5 | 6.50E-195 |
| FimC | 808 | ECDEC12D_5612 | EHX38156 | gram-negative pili assembly chaperone, N-terminal domain protein | Escherichia coli DEC12D | 660.5 | 6.50E-195 |
| FimC | 809 | BFF50_22095 | ODG79012 | molecular chaperone FimC | Shigella sp. FC2928 | 660.5 | 6.50E-195 |
| FimC | 810 | V411_26240 | ETE09725 | molecular chaperone FimC | Escherichia coli LAU-EC6 | 660.5 | 6.50E-195 |
| FimC | 811 | AB83_4915 | KDS94707 | chaperone protein fimC | Escherichia coli 2-011-08_S3_C1 | 660.5 | 6.50E-195 |
| FimC | 812 | WK7_04599 | ELI90002 | fimbrial chaperone FimC | Escherichia coli KTE148 | 660.5 | 6.50E-195 |
| FimC | 813 | G747_04450 | EQP87839 | chaperone fimC | Escherichia coli HVH 85 (4-0792144) | 660.5 | 6.50E-195 |
| FimC | 814 | ECOG_02489 | EGI52680 | chaperone protein FimC | Escherichia coli H299 | 660.5 | 6.50E-195 |
| FimC | 815 | ECDEC10B_0025 | EHW79987 | gram-negative pili assembly chaperone, N-terminal domain protein | Escherichia coli DEC10B | 660.5 | 6.50E-195 |
| FimC | 816 | WIQ_04631 | ELI49494 | fimbrial chaperone FimC | Escherichia coli KTE128 | 660.5 | 6.50E-195 |
| FimC | 817 | ECO9545_16446 | EIL20789 | chaperone FimC, periplasmic | Escherichia coli O111:H11 str. CVM9545 | 660.5 | 6.50E-195 |
| FimC | 818 | ECDEC11A_4998 | EHW84760 | gram-negative pili assembly chaperone, N-terminal domain protein | Escherichia coli DEC11A | 660.5 | 6.50E-195 |
| FimC | 819 | G816_04473 | EQS57101 | chaperone fimC | Escherichia coli HVH 158 (4-3224287) | 660.5 | 6.50E-195 |
| FimC | 820 | AB02_4572 | KDX64023 | chaperone protein fimC | Escherichia coli 2-222-05_S1_C1 | 660.5 | 6.50E-195 |
| FimC | 821 | AB87_4325 | KDX53009 | chaperone protein fimC | Escherichia coli 2-210-07_S3_C1 | 660.5 | 6.50E-195 |
| FimC | 822 | BX68_04270 | EYZ00080 | molecular chaperone FimC | Escherichia coli O177:NM str. 2010C-4558 | 660.5 | 6.50E-195 |
| FimC | 823 | BX86_16270 | EYY39720 | molecular chaperone FimC | Escherichia coli O153:H2 str. 2010C-5034 | 660.5 | 6.50E-195 |
| FimC | 824 | BW90_07295 | EYZ69611 | molecular chaperone FimC | Escherichia coli O118:H16 str. 06-3612 | 660.5 | 6.50E-195 |
| FimC | 825 | AB53_4968 | EZK10062 | chaperone protein fimC | Escherichia coli 2-005-03_S1_C3 | 660.5 | 6.50E-195 |
| FimC | 826 | ECJG_03530 | EGI23293 | chaperone protein FimC | Escherichia coli M718 | 660.5 | 6.50E-195 |
| FimC | 827 | HMPREF1606_00954 | ESD61049 | gram-negative pili assembly chaperone domain protein | Escherichia coli 908522 | 660.5 | 6.50E-195 |
| FimC | 828 | G780_04720 | EQR11764 | chaperone fimC | Escherichia coli HVH 118 (4-7345399) | 660.5 | 6.50E-195 |
| FimC | 829 | G858_04771 | EQU46078 | chaperone fimC | Escherichia coli HVH 206 (4-3128229) | 660.5 | 6.50E-195 |
| FimC | 830 | G944_04805 | EQY08475 | chaperone fimC | Escherichia coli UMEA 3215-1 | 660.5 | 6.50E-195 |
| FimC | 831 | G867_04848 | EQU82038 | chaperone fimC | Escherichia coli HVH 215 (4-3008371) | 660.5 | 6.50E-195 |
| FimC | 832 | G894_04453 | EQV93957 | chaperone fimC | Escherichia coli KOEGE 73 (195a) | 660.5 | 6.50E-195 |
| FimC | 833 | ECNA114_4561 | AEG39410 | Periplasmic chaperone FimC | Escherichia coli NA114 | 660.5 | 6.50E-195 |
| FimC | 834 | A1SO_00590 | ELE27251 | chaperone fimC | Escherichia coli KTE58 | 660.5 | 6.50E-195 |
| FimC | 835 | G860_04855 | EQU56243 | chaperone fimC | Escherichia coli HVH 208 (4-3112292) | 660.5 | 6.50E-195 |
| FimC | 836 | A1Y1_04528 | ELG45756 | fimbrial chaperone FimC | Escherichia coli KTE115 | 660.5 | 6.50E-195 |
| FimC | 837 | A133_00462 | ELH30419 | fimbrial chaperone FimC | Escherichia coli KTE173 | 660.5 | 6.50E-195 |
| FimC | 838 | ECO9455_01960 | EJE96832 | chaperone FimC, periplasmic | Escherichia coli O111:H11 str. CVM9455 | 660.5 | 6.50E-195 |
| FimC | 839 | BX06_02285 | EZE12869 | molecular chaperone FimC | Escherichia coli O69:H11 str. 08-4661 | 660.5 | 6.50E-195 |
| FimC | 840 | A1YI_00409 | EOW53767 | fimbrial chaperone FimC | Escherichia coli KTE132 | 660.5 | 6.50E-195 |
| FimC | 841 | A19A_00282 | EOX16869 | fimbrial chaperone FimC | Escherichia coli KTE240 | 660.5 | 6.50E-195 |
| FimC | 842 | A17Q_04739 | EOX03373 | fimbrial chaperone FimC | Escherichia coli KTE226 | 660.5 | 6.50E-195 |
| FimC | 843 | A1YO_00355 | ELG73925 | fimbrial chaperone FimC | Escherichia coli KTE136 | 660.5 | 6.50E-195 |
| FimC | 844 | EC3003_4855 | EII86800 | gram-negative pili assembly chaperone, N-terminal domain protein | Escherichia coli 3003 | 659.5 | 1.30E-194 |
| FimC | 845 | ECARS42123_4866 | EKI21482 | type I fimbrial chaperone | Escherichia coli ARS4.2123 | 659.5 | 1.30E-194 |
| FimC | 846 | ECRN5871_1619 | EFZ75112 | chaperone protein fimC | Escherichia coli RN587/1 | 659.5 | 1.30E-194 |
| FimC | 847 | ECED1_5201 | CAR11154 | chaperone, periplasmic | Escherichia coli ED1a | 659.1 | 1.70E-194 |
| FimC | 848 | ECSTECDG1313_0204 | EGW98763 | chaperone protein fimC | Escherichia coli STEC_DG131-3 | 658.7 | 2.30E-194 |
| FimC | 849 | ECMP0209401_0053 | EMX61202 | chaperone protein fimC | Escherichia coli MP020940.1 | 658.7 | 2.30E-194 |
| FimC | 850 | EC970259_5203 | EIH46694 | gram-negative pili assembly chaperone, N-terminal domain protein | Escherichia coli 99.0741 | 658.7 | 2.30E-194 |
| FimC | 851 | WR19_20260 | KLH77133 | molecular chaperone FimC | Escherichia coli (strain ATCC 9637 / CCM 2024 / DSM 1116 / NCIMB 8666 / NRRL B-766 / W) | 658.7 | 2.30E-194 |
| FimC | 852 | ECMT8_17711 | EIL75076 | type-1 fimbrial chaperone FimC | Escherichia coli CUMT8 | 658.7 | 2.30E-194 |
| FimC | 853 | AB90_4017 | KDY30866 | chaperone protein fimC | Escherichia coli 2-427-07_S3_C1 | 658.7 | 2.30E-194 |
| FimC | 854 | A1U5_00243 | ELE46152 | chaperone fimC | Escherichia coli KTE66 | 658.7 | 2.30E-194 |
| FimC | 855 | EC179100_4991 | END29357 | chaperone protein fimC | Escherichia coli 179100 | 658.7 | 2.30E-194 |
| FimC | 856 | BW70_02160 | EZA37360 | molecular chaperone FimC | Escherichia coli O174:H8 str. 04-3038 | 658.7 | 2.30E-194 |
| FimC | 857 | AD40_5108 | KEN62228 | chaperone protein fimC | Escherichia coli 1-392-07_S4_C3 | 658.7 | 2.30E-194 |
| FimC | 858 | AE34_00422 | KDF73173 | chaperone fimC | Escherichia coli BIDMC 59 | 658.7 | 2.30E-194 |
| FimC | 859 | ECP03048165_4872 | ENH33704 | chaperone protein fimC | Escherichia coli P0304816.5 | 658.7 | 2.30E-194 |
| FimC | 860 | ECP030481613_4832 | ENF35149 | chaperone protein fimC | Escherichia coli P0304816.13 | 658.7 | 2.30E-194 |
| FimC | 861 | G896_04548 | EQV96071 | chaperone fimC | Escherichia coli KOEGE 118 (317a) | 658.7 | 2.30E-194 |
| FimC | 862 | BX24_08665 | EZE65815 | molecular chaperone FimC | Escherichia coli O91:H21 str. 2009C-4646 | 658.7 | 2.30E-194 |
| FimC | 863 | ECP030481614_4749 | ENF29358 | chaperone protein fimC | Escherichia coli P0304816.14 | 658.7 | 2.30E-194 |
| FimC | 864 | ECP030481611_4779 | ENF16180 | chaperone protein fimC | Escherichia coli P0304816.11 | 658.7 | 2.30E-194 |
| FimC | 865 | A1US_00273 | ELG30551 | fimbrial chaperone FimC | Escherichia coli KTE78 | 658.7 | 2.30E-194 |
| FimC | 866 | BX16_16445 | EZE34089 | molecular chaperone FimC | Escherichia coli O91:NM str. 2009C-3745 | 658.7 | 2.30E-194 |
| FimC | 867 | WCK_00545 | ELF59702 | fimbrial chaperone FimC | Escherichia coli KTE9 | 658.7 | 2.30E-194 |
| FimC | 868 | ECCG_02901 | EFE60474 | fimbrial chaperone | Escherichia coli B088 | 658.7 | 2.30E-194 |
| FimC | 869 | ECDEC13C_5263 | EHX54312 | gram-negative pili assembly chaperone, N-terminal domain protein | Escherichia coli DEC13C | 658.7 | 2.30E-194 |
| FimC | 870 | EREG_02533 | EGB41877 | gram-negative pili assembly chaperone domain-containing protein | Escherichia coli H120 | 658.7 | 2.30E-194 |
| FimC | 871 | AF33_04551 | KDG50632 | chaperone fimC | Escherichia coli CHS 77 | 658.7 | 2.30E-194 |
| FimC | 872 | ECDEC13A_0088 | EHX52172 | gram-negative pili assembly chaperone, N-terminal domain protein | Escherichia coli DEC13A | 658.7 | 2.30E-194 |
| FimC | 873 | AC97_3722 | KDW42118 | chaperone protein fimC | Escherichia coli 2-177-06_S4_C2 | 658.7 | 2.30E-194 |
| FimC | 874 | HMPREF1590_02964 | ESC97933 | gram-negative pili assembly chaperone domain protein | Escherichia coli 113302 | 658.7 | 2.30E-194 |
| FimC | 875 | C202_21561 | EMD02562 | type-1 fimbrial chaperone FimC | Escherichia coli O08 | 658.7 | 2.30E-194 |
| FimC | 876 | ECSTECO31_4919 | EJK93358 | chaperone protein fimC | Escherichia coli STEC_O31 | 658.7 | 2.30E-194 |
| FimC | 877 | G805_04504 | EQS28703 | chaperone fimC | Escherichia coli HVH 147 (4-5893887) | 658.7 | 2.30E-194 |
| FimC | 878 | G777_00484 | EQR06840 | chaperone fimC | Escherichia coli HVH 115 (4-4465989) | 658.7 | 2.30E-194 |
| FimC | 879 | A15G_01128 | ELH55218 | fimbrial chaperone FimC | Escherichia coli KTE203 | 658.7 | 2.30E-194 |
| FimC | 880 | AC84_5018 | KEN94695 | chaperone protein fimC | Escherichia coli 1-392-07_S4_C1 | 658.7 | 2.30E-194 |
| FimC | 881 | AB05_5044 | KEO24643 | chaperone protein fimC | Escherichia coli 2-460-02_S1_C1 | 658.7 | 2.30E-194 |
| FimC | 882 | G776_04658 | EQQ96813 | chaperone fimC | Escherichia coli HVH 115 (4-4465997) | 658.7 | 2.30E-194 |
| FimC | 883 | ECP03048168_4798 | ENF64183 | chaperone protein fimC | Escherichia coli P0304816.8 | 658.7 | 2.30E-194 |
| FimC | 884 | BX07_07430 | EZD94717 | molecular chaperone FimC | Escherichia coli O91:H14 str. 2009C-3227 | 658.7 | 2.30E-194 |
| FimC | 885 | A1UU_01767 | ELG33921 | fimbrial chaperone FimC | Escherichia coli KTE79 | 658.7 | 2.30E-194 |
| FimC | 886 | A15U_00342 | ELD25456 | chaperone fimC | Escherichia coli KTE210 | 658.7 | 2.30E-194 |
| FimC | 887 | HMPREF9542_04434 | EGB86153 | gram-negative pili assembly chaperone domain protein | Escherichia coli MS 117-3 | 658.7 | 2.30E-194 |
| FimC | 888 | ECDEC13D_4976 | EHX56681 | gram-negative pili assembly chaperone, N-terminal domain protein | Escherichia coli DEC13D | 658.7 | 2.30E-194 |
| FimC | 889 | EC54115_03947 | EIL58001 | type-1 fimbrial chaperone FimC | Escherichia coli 541-15 | 658.7 | 2.30E-194 |
| FimC | 890 | LY180_22640 | AGW11317 | molecular chaperone FimC | Escherichia coli LY180 | 658.7 | 2.30E-194 |
| FimC | 891 | AC88_5109 | KEJ55383 | chaperone protein fimC | Escherichia coli 3-267-03_S4_C1 | 658.7 | 2.30E-194 |
| FimC | 892 | EC90111_5734 | EII21311 | gram-negative pili assembly chaperone, N-terminal domain protein | Escherichia coli 9.0111 | 658.7 | 2.30E-194 |
| FimC | 893 | ECNG_01385 | EGI42631 | putative gram-negative pili assembly chaperone, N-domain protein | Escherichia coli TA280 | 658.7 | 2.30E-194 |
| FimC | 894 | G686_04537 | EQN19239 | chaperone fimC | Escherichia coli HVH 6 (3-8296502) | 658.7 | 2.30E-194 |
| FimC | 895 | KO11_23205 | AFH19597 | type-1 fimbrial chaperone FimC | Escherichia coli KO11 | 658.7 | 2.30E-194 |
| FimC | 896 | EschWDRAFT_4533 | EFN35886 | Pili assembly chaperone | Escherichia coli W (GCA_000258145) | 658.7 | 2.30E-194 |
| FimC | 897 | ECP03048167_4879 | ENF58804 | chaperone protein fimC | Escherichia coli P0304816.7 | 658.7 | 2.30E-194 |
| FimC | 898 | LI75_01960 | AIT33116 | molecular chaperone FimC | Escherichia coli FAP1 | 658.7 | 2.30E-194 |
| FimC | 899 | A1UM_00142 | ELE59498 | chaperone fimC | Escherichia coli KTE75 | 658.7 | 2.30E-194 |
| FimC | 900 | AC48_3480 | KDY30412 | chaperone protein fimC | Escherichia coli 2-427-07_S3_C3 | 658.7 | 2.30E-194 |
| FimC | 901 | BX81_02395 | EYY46068 | molecular chaperone FimC | Escherichia coli O165:H25 str. 2010C-4874 | 658.7 | 2.30E-194 |
| FimC | 902 | ESQG_01457 | EHN86479 | chaperone fimC | Escherichia coli H494 | 658.7 | 2.30E-194 |
| FimC | 903 | A1YM_01691 | ELG65695 | fimbrial chaperone FimC | Escherichia coli KTE135 | 658.7 | 2.30E-194 |
| FimC | 904 | ECP02994383_4884 | ENB93569 | chaperone protein fimC | Escherichia coli P0299438.3 | 658.7 | 2.30E-194 |
| FimC | 905 | AD31_5184 | KEJ42813 | chaperone protein fimC | Escherichia coli 2-427-07_S4_C3 | 658.7 | 2.30E-194 |
| FimC | 906 | G995_04744 | ERA00097 | chaperone fimC | Escherichia coli UMEA 3805-1 | 658.7 | 2.30E-194 |
| FimC | 907 | ECoL_02140 | EFW75161 | chaperone FimC | Escherichia coli EC4100B | 658.7 | 2.30E-194 |
| FimC | 908 | BW91_08665 | EYZ42976 | molecular chaperone FimC | Escherichia coli O91:H14 str. 06-3691 | 658.7 | 2.30E-194 |
| FimC | 909 | ECP03048161_2217 | EMZ97822 | chaperone protein fimC | Escherichia coli P0304816.1 | 658.7 | 2.30E-194 |
| FimC | 910 | ECP029943811_4901 | ENB91452 | chaperone protein fimC | Escherichia coli P0299438.11 | 658.7 | 2.30E-194 |
| FimC | 911 | EC174900_4755 | EMZ59499 | chaperone protein fimC | Escherichia coli 174900 | 658.7 | 2.30E-194 |
| FimC | 912 | HMPREF9548_04475 | EFK00876 | gram-negative pili assembly chaperone domain protein | Escherichia coli MS 182-1 | 658.7 | 2.30E-194 |
| FimC | 913 | AC07_2913 | KEK76817 | chaperone protein fimC | Escherichia coli 3-475-03_S3_C1 | 658.7 | 2.30E-194 |
| FimC | 914 | WR24_00615 | KLG75139 | molecular chaperone FimC | Escherichia coli (GCA_001012495) | 658.5 | 2.60E-194 |
| FimC | 915 | L476_04473 | ESL16955 | chaperone fimC | Escherichia coli BIDMC 39 | 658.5 | 2.70E-194 |
| FimC | 916 | G913_04371 | EQW81438 | chaperone fimC | Escherichia coli UMEA 3124-1 | 658.5 | 2.70E-194 |
| FimC | 917 | AC78_4467 | KEN37375 | chaperone protein fimC | Escherichia coli 7-233-03_S4_C1 | 658.5 | 2.80E-194 |
| FimC | 918 | AD35_4419 | KEN49902 | chaperone protein fimC | Escherichia coli 7-233-03_S4_C3 | 658.5 | 2.80E-194 |
| FimC | 919 | HMPREF9533_03085 | EGB82100 | gram-negative pili assembly chaperone domain protein | Escherichia coli MS 60-1 | 658.4 | 2.80E-194 |
| FimC | 920 | WQ87_07745 | KNG26062 | molecular chaperone FimC | Escherichia coli (GCA_001191345) | 658.4 | 2.90E-194 |
| FimC | 921 | UN94_19680 | KJW62905 | molecular chaperone FimC | Escherichia coli (GCA_000965715) | 658 | 3.70E-194 |
| FimC | 922 | BX09_24195 | EZD97573 | molecular chaperone FimC | Escherichia coli O145:H28 str. 2009C-3292 | 658 | 3.70E-194 |
| FimC | 923 | EC2732_17046 | KRR51303 | type I fimbrial chaperone | Escherichia coli VL2732 | 658 | 3.70E-194 |
| FimC | 924 | AB43_3429 | KDZ24774 | chaperone protein fimC | Escherichia coli 3-020-07_S1_C2 | 658 | 3.70E-194 |
| FimC | 925 | G849_04769 | EQU07268 | chaperone fimC | Escherichia coli HVH 197 (4-4466217) | 658 | 3.70E-194 |
| FimC | 926 | AB36_4561 | KEM57341 | chaperone protein fimC | Escherichia coli 7-233-03_S1_C2 | 658 | 3.70E-194 |
| FimC | 927 | AF43_04046 | KDG61513 | chaperone fimC | Escherichia coli MGH 57 | 658 | 3.70E-194 |
| FimC | 928 | AB15_3517 | KDZ21349 | chaperone protein fimC | Escherichia coli 3-020-07_S1_C1 | 658 | 3.70E-194 |
| FimC | 929 | AB68_4559 | KEM95949 | chaperone protein fimC | Escherichia coli 7-233-03_S1_C3 | 658 | 3.70E-194 |
| FimC | 930 | G998_04270 | ERA13131 | chaperone fimC | Escherichia coli UMEA 3889-1 | 658 | 3.70E-194 |
| FimC | 931 | G654_03762 | EWC57540 | type I fimbrial chaperone | Escherichia coli EC096/10 | 658 | 3.70E-194 |
| FimC | 932 | A313_03076 | ELG89863 | fimbrial chaperone FimC | Escherichia coli KTE147 | 658 | 3.70E-194 |
| FimC | 933 | ECIAI39_4789 | CAR20886 | chaperone, periplasmic | Escherichia coli IAI39 | 657.9 | 4.20E-194 |
| FimC | 934 | CE10_5060 | AEQ15722 | chaperone, periplasmic | Escherichia coli O7:K1 str. CE10 | 657.9 | 4.20E-194 |
| FimC | 935 | G725_04894 | EQO90976 | chaperone fimC | Escherichia coli HVH 53 (4-0631051) | 657.9 | 4.20E-194 |
| FimC | 936 | G952_04892 | EQY50236 | chaperone fimC | Escherichia coli UMEA 3240-1 | 657.9 | 4.20E-194 |
| FimC | 937 | H000_04157 | ERA29480 | chaperone fimC | Escherichia coli UMEA 3899-1 | 657.9 | 4.20E-194 |
| FimC | 938 | G839_03862 | EQT55711 | chaperone fimC | Escherichia coli HVH 187 (4-4471660) | 657.9 | 4.20E-194 |
| FimC | 939 | WQ89_04500 | KNF81722 | molecular chaperone FimC | Escherichia coli O7:K1 (strain IAI39 / ExPEC) | 657.9 | 4.20E-194 |
| FimC | 940 | AB51_4719 | KEM08630 | chaperone protein fimC | Escherichia coli 6-319-05_S1_C2 | 657.9 | 4.20E-194 |
| FimC | 941 | ECP030529313_4580 | END30667 | chaperone protein fimC | Escherichia coli p0305293.13 | 657.9 | 4.20E-194 |
| FimC | 942 | ECENVIRA811_0032 | EMX78082 | chaperone protein fimC | Escherichia coli Envira 8/11 | 657.9 | 4.20E-194 |
| FimC | 943 | ECP03052936_4607 | ENH50962 | chaperone protein fimC | Escherichia coli p0305293.6 | 657.9 | 4.20E-194 |
| FimC | 944 | EC2866350_4685 | ENB03999 | chaperone protein fimC | Escherichia coli 2866350 | 657.9 | 4.20E-194 |
| FimC | 945 | EC2860650_4710 | ENA88530 | chaperone protein fimC | Escherichia coli 2860650 | 657.9 | 4.20E-194 |
| FimC | 946 | AB23_4762 | KEM96532 | chaperone protein fimC | Escherichia coli 6-319-05_S1_C1 | 657.9 | 4.20E-194 |
| FimC | 947 | ECP03052931_5086 | EMZ79611 | chaperone protein fimC | Escherichia coli p0305293.1 | 657.9 | 4.20E-194 |
| FimC | 948 | ECP030529312_4705 | ENG39267 | chaperone protein fimC | Escherichia coli p0305293.12 | 657.9 | 4.20E-194 |
| FimC | 949 | ECP030529311_4817 | ENG35151 | chaperone protein fimC | Escherichia coli p0305293.11 | 657.9 | 4.20E-194 |
| FimC | 950 | ECP03052933_4689 | ENG57224 | chaperone protein fimC | Escherichia coli p0305293.3 | 657.9 | 4.20E-194 |
| FimC | 951 | ECP03052939_4586 | ENG74314 | chaperone protein fimC | Escherichia coli p0305293.9 | 657.9 | 4.20E-194 |
| FimC | 952 | ECP030529314_4857 | ENE04571 | chaperone protein fimC | Escherichia coli p0305293.14 | 657.9 | 4.20E-194 |
| FimC | 953 | L339_00648 | ESV05722 | chaperone FimC | Escherichia coli E1777 | 657.9 | 4.20E-194 |
| FimC | 954 | ECP03052937_4745 | ENH48661 | chaperone protein fimC | Escherichia coli p0305293.7 | 657.9 | 4.20E-194 |
| FimC | 955 | EC2747800_4717 | EMW70279 | chaperone protein fimC | Escherichia coli 2747800 | 657.9 | 4.20E-194 |
| FimC | 956 | ECENVIRA101_0026 | EMX76336 | chaperone protein fimC | Escherichia coli Envira 10/1 | 657.9 | 4.20E-194 |
| FimC | 957 | ECP03052935_4631 | ENH41237 | chaperone protein fimC | Escherichia coli p0305293.5 | 657.9 | 4.20E-194 |
| FimC | 958 | EC2860050_4807 | EMV87470 | chaperone protein fimC | Escherichia coli 2860050 | 657.9 | 4.20E-194 |
| FimC | 959 | ECP03052932_4659 | ENG51729 | chaperone protein fimC | Escherichia coli p0305293.2 | 657.9 | 4.20E-194 |
| FimC | 960 | ECP030529310_4629 | ENG25613 | chaperone protein fimC | Escherichia coli p0305293.10 | 657.9 | 4.20E-194 |
| FimC | 961 | EC2729250_4764 | ENA47455 | chaperone protein fimC | Escherichia coli 2729250 | 657.9 | 4.20E-194 |
| FimC | 962 | AB80_4972 | KEM21293 | chaperone protein fimC | Escherichia coli 6-319-05_S1_C3 | 657.9 | 4.20E-194 |
| FimC | 963 | ECP03052938_4727 | ENG67739 | chaperone protein fimC | Escherichia coli p0305293.8 | 657.9 | 4.20E-194 |
| FimC | 964 | ECP030529315_4769 | ENG48116 | chaperone protein fimC | Escherichia coli p0305293.15 | 657.9 | 4.20E-194 |
| FimC | 965 | L340_3491 | EPH48410 | chaperone FimC | Escherichia coli E2265 | 657.9 | 4.20E-194 |
| FimC | 966 | ECP03052934_4739 | ENG57693 | chaperone protein fimC | Escherichia coli p0305293.4 | 657.9 | 4.20E-194 |
| FimC | 967 | EC2770900_4684 | EMW46552 | chaperone protein fimC | Escherichia coli 2770900 | 657.9 | 4.20E-194 |
| FimC | 968 | BY02_03345 | EYY00189 | molecular chaperone FimC | Escherichia coli O121:H19 str. 2011C-3537 | 657.8 | 4.30E-194 |
| FimC | 969 | BX52_15290 | EYV08538 | molecular chaperone FimC | Escherichia coli O121:H19 str. 2010C-3609 | 657.8 | 4.30E-194 |
| FimC | 970 | BX87_04760 | EYY33130 | molecular chaperone FimC | Escherichia coli O121:H19 str. 2010EL1058 | 657.8 | 4.30E-194 |
| FimC | 971 | BY00_02670 | EYY03495 | molecular chaperone FimC | Escherichia coli O121:H19 str. 2011C-3500 | 657.8 | 4.30E-194 |
| FimC | 972 | BX94_04800 | EYY14697 | molecular chaperone FimC | Escherichia coli O121:H19 str. 2011C-3216 | 657.8 | 4.30E-194 |
| FimC | 973 | BX19_15130 | EZE42777 | molecular chaperone FimC | Escherichia coli O121:H19 str. 2009C-4050 | 657.8 | 4.30E-194 |
| FimC | 974 | BX32_22705 | EYV80097 | molecular chaperone FimC | Escherichia coli O121:H19 str. 2009EL1412 | 657.8 | 4.30E-194 |
| FimC | 975 | BX31_00805 | EZE90226 | molecular chaperone FimC | Escherichia coli O121:H19 str. 2009EL1302 | 657.8 | 4.30E-194 |
| FimC | 976 | BU57_33895 | KDV49384 | molecular chaperone FimC | Escherichia coli O121:H19 str. 2011C-3609 | 657.8 | 4.30E-194 |
| FimC | 977 | BW83_02290 | EYZ86847 | molecular chaperone FimC | Escherichia coli O121:H19 str. 06-3003 | 657.8 | 4.30E-194 |
| FimC | 978 | UC21_09125 | KJY11859 | molecular chaperone FimC | Escherichia coli (GCA_000965635) | 657.8 | 4.30E-194 |
| FimC | 979 | BX92_16420 | EYY20979 | molecular chaperone FimC | Escherichia coli O121:H19 str. 2011C-3108 | 657.8 | 4.30E-194 |
| FimC | 980 | BY81_11695 | EZC56796 | molecular chaperone FimC | Escherichia coli O121:H19 str. K5269 | 657.8 | 4.30E-194 |
| FimC | 981 | BX27_21955 | EZE76679 | molecular chaperone FimC | Escherichia coli O121:H19 str. 2009C-4750 | 657.8 | 4.30E-194 |
| FimC | 982 | BX91_04185 | EYY27551 | molecular chaperone FimC | Escherichia coli O121:H19 str. 2011C-3072 | 657.8 | 4.30E-194 |
| FimC | 983 | BX53_00465 | EZE99949 | molecular chaperone FimC | Escherichia coli O121:H19 str. 2010C-3794 | 657.8 | 4.30E-194 |
| FimC | 984 | A311_00411 | ELG92200 | fimbrial chaperone FimC | Escherichia coli KTE146 | 657.8 | 4.30E-194 |
| FimC | 985 | BX62_24905 | EYU80175 | molecular chaperone FimC | Escherichia coli O121:H19 str. 2010C-4254 | 657.8 | 4.30E-194 |
| FimC | 986 | BX25_24900 | EYV82735 | molecular chaperone FimC | Escherichia coli O121:H19 str. 2009C-4659 | 657.8 | 4.30E-194 |
| FimC | 987 | BX79_06245 | EYY63249 | molecular chaperone FimC | Escherichia coli O121:H19 str. 2010C-4824 | 657.8 | 4.30E-194 |
| FimC | 988 | BY47_07750 | EZA94059 | molecular chaperone FimC | Escherichia coli O121:H19 str. F6714 | 657.8 | 4.30E-194 |
| FimC | 989 | BX72_10265 | EYY89863 | molecular chaperone FimC | Escherichia coli O121:H19 str. 2010C-4732 | 657.8 | 4.30E-194 |
| FimC | 990 | BW77_17775 | EZA12182 | molecular chaperone FimC | Escherichia coli O121:H19 str. 03-3227 | 657.8 | 4.30E-194 |
| FimC | 991 | BX84_16400 | EYY37876 | molecular chaperone FimC | Escherichia coli O121:H19 str. 2010C-4989 | 657.8 | 4.30E-194 |
| FimC | 992 | BW93_13955 | EYZ46480 | molecular chaperone FimC | Escherichia coli O121:H19 str. 06-3822 | 657.8 | 4.30E-194 |
| FimC | 993 | BX54_06925 | EYV01814 | molecular chaperone FimC | Escherichia coli O121:H19 str. 2010C-3840 | 657.8 | 4.30E-194 |
| FimC | 994 | BX82_16190 | EYY51277 | molecular chaperone FimC | Escherichia coli O121:H19 str. 2010C-4966 | 657.8 | 4.30E-194 |
| FimC | 995 | Q460_23945 | ETI71937 | molecular chaperone FimC | Escherichia coli ATCC BAA-2219 | 657.8 | 4.30E-194 |
| FimC | 996 | BY80_11930 | EZC53188 | molecular chaperone FimC | Escherichia coli O121:H19 str. K5198 | 657.8 | 4.30E-194 |
| FimC | 997 | PU08_17705 | KHJ06398 | molecular chaperone FimC | Escherichia coli (GCA_000798595) | 657.8 | 4.40E-194 |
| FimC | 998 | ECAI27_01290 | EIE57783 | type-1 fimbrial chaperone FimC | Escherichia coli AI27 | 657.8 | 4.40E-194 |
| FimC | 999 | ECSE_4589 | BAG80113 | type-1 fimbrial chaperone FimC | Escherichia coli (strain SE11) | 657.8 | 4.40E-194 |
| FimC | 1000 | G842_00613 | EQT71817 | chaperone fimC | Escherichia coli HVH 190 (4-3255514) | 657.8 | 4.50E-194 |
| FimC | 1001 | A1WK_00579 | EOW12358 | fimbrial chaperone FimC | Escherichia coli KTE100 | 657.8 | 4.50E-194 |
| FimC | 1002 | A1U1_04596 | EOV52257 | fimbrial chaperone FimC | Escherichia coli KTE64 | 657.8 | 4.50E-194 |
| FimC | 1003 | JQ59_22320 | KGP37506 | molecular chaperone FimC | Escherichia coli (GCA_000773435) | 657.8 | 4.50E-194 |
| FimC | 1004 | HMPREF9540_00353 | EFJ99530 | gram-negative pili assembly chaperone domain protein | Escherichia coli MS 115-1 | 657.8 | 4.50E-194 |
| FimC | 1005 | A193_00581 | ELD74369 | chaperone fimC | Escherichia coli KTE234 | 657.8 | 4.50E-194 |
| FimC | 1006 | L474_04414 | ESL31364 | chaperone fimC | Escherichia coli BIDMC 37 | 657.8 | 4.50E-194 |
| FimC | 1007 | ACU82_19270 | KPO47984 | molecular chaperone FimC | Escherichia coli (GCA_001309595) | 657.7 | 4.60E-194 |
| FimC | 1008 | AC17_2105 | KDW99707 | chaperone protein fimC | Escherichia coli 2-210-07_S3_C2 | 657.7 | 4.70E-194 |
| FimC | 1009 | ECK71_07018 | KRR59680 | chaperone FimC, periplasmic | Escherichia coli K71 | 657.7 | 4.70E-194 |
| FimC | 1010 | ECO9634_18966 | EJE75072 | chaperone FimC, periplasmic | Escherichia coli O111:H8 str. CVM9634 | 657.7 | 4.70E-194 |
| FimC | 1011 | AE49_03673 | KDG17547 | chaperone fimC | Escherichia coli BIDMC 74 | 657.7 | 4.70E-194 |
| FimC | 1012 | ECIAI1_4532 | CAR01273 | chaperone, periplasmic | Escherichia coli IAI1 | 657.7 | 4.70E-194 |
| FimC | 1013 | ECO111_5170 | BAI38887 | chaperone FimC, periplasmic | Escherichia coli O111:H- str. 11128 | 657.7 | 4.70E-194 |
| FimC | 1014 | AD14_3404 | KDZ73633 | chaperone protein fimC | Escherichia coli 3-073-06_S4_C2 | 657.7 | 4.70E-194 |
| FimC | 1015 | BX44_01780 | EYV38380 | molecular chaperone FimC | Escherichia coli O145:NM str. 2010C-3509 | 657.7 | 4.70E-194 |
| FimC | 1016 | ECDEC8E_5336 | EHW21327 | gram-negative pili assembly chaperone, N-terminal domain protein | Escherichia coli DEC8E | 657.7 | 4.70E-194 |
| FimC | 1017 | WGU_00016 | ELJ87466 | fimbrial chaperone FimC | Escherichia coli KTE90 | 657.7 | 4.70E-194 |
| FimC | 1018 | BX42_05825 | EYV57490 | molecular chaperone FimC | Escherichia coli O145:NM str. 2010C-3507 | 657.7 | 4.70E-194 |
| FimC | 1019 | ECDEC15E_0004 | EHY21322 | gram-negative pili assembly chaperone, N-terminal domain protein | Escherichia coli DEC15E | 657.7 | 4.70E-194 |
| FimC | 1020 | O199_0202250 | ETJ70867 | molecular chaperone FimC | Escherichia coli ATCC 35150 | 657.7 | 4.70E-194 |
| FimC | 1021 | DC23_14405 | KDM77233 | molecular chaperone FimC | Escherichia coli O145:H28 str. 4865/96 | 657.7 | 4.70E-194 |
| FimC | 1022 | EC33884_4995 | EII55939 | gram-negative pili assembly chaperone, N-terminal domain protein | Escherichia coli 3.3884 | 657.7 | 4.70E-194 |
| FimC | 1023 | G968_04302 | ESK01925 | chaperone fimC | Escherichia coli UMEA 3336-1 | 657.7 | 4.70E-194 |
| FimC | 1024 | HMPREF1591_03542 | ESA62902 | gram-negative pili assembly chaperone domain protein | Escherichia coli 113303 | 657.7 | 4.70E-194 |
| FimC | 1025 | EC2780750_4863 | EMW44901 | chaperone protein fimC | Escherichia coli 2780750 | 657.7 | 4.70E-194 |
| FimC | 1026 | AB38_4598 | EYE32309 | chaperone protein fimC | Escherichia coli 1-110-08_S1_C2 | 657.7 | 4.70E-194 |
| FimC | 1027 | G960_04693 | ERB28437 | chaperone fimC | Escherichia coli UMEA 3292-1 | 657.7 | 4.70E-194 |
| FimC | 1028 | ECW26_02940 | EID69036 | gram-negative pili assembly chaperone protein | Escherichia coli W26 | 657.7 | 4.70E-194 |
| FimC | 1029 | BX51_00370 | EYV20063 | molecular chaperone FimC | Escherichia coli O145:NM str. 2010C-3526 | 657.7 | 4.70E-194 |
| FimC | 1030 | AB18_1742 | KDU20001 | chaperone protein fimC | Escherichia coli 3-267-03_S1_C1 | 657.7 | 4.70E-194 |
| FimC | 1031 | A13E_01200 | ELH37519 | fimbrial chaperone FimC | Escherichia coli KTE184 | 657.7 | 4.70E-194 |
| FimC | 1032 | BX46_11030 | EYV44991 | molecular chaperone FimC | Escherichia coli O145:NM str. 2010C-3511 | 657.7 | 4.70E-194 |
| FimC | 1033 | EcHS_A4542 | ABV08695 | chaperone protein FimC | Escherichia coli HS | 657.7 | 4.70E-194 |
| FimC | 1034 | BW87_04360 | EYZ76649 | molecular chaperone FimC | Escherichia coli O145:NM str. 06-3484 | 657.7 | 4.70E-194 |
| FimC | 1035 | BX47_26890 | EYV24890 | molecular chaperone FimC | Escherichia coli O145:NM str. 2010C-3516 | 657.7 | 4.70E-194 |
| FimC | 1036 | BX69_00600 | EYZ00773 | molecular chaperone FimC | Escherichia coli O111:NM str. 2010C-4592 | 657.7 | 4.70E-194 |
| FimC | 1037 | ECOT7509_4666 | ERC51130 | chaperone protein fimC | Escherichia coli TW07509 | 657.7 | 4.70E-194 |
| FimC | 1038 | ECMP0209801_0003 | END56534 | chaperone protein fimC | Escherichia coli MP020980.1 | 657.7 | 4.70E-194 |
| FimC | 1039 | BX59_03340 | EYU86276 | molecular chaperone FimC | Escherichia coli O111:NM str. 2010C-4086 | 657.7 | 4.70E-194 |
| FimC | 1040 | AB85_4857 | KDV97215 | chaperone protein fimC | Escherichia coli 2-156-04_S3_C1 | 657.7 | 4.70E-194 |
| FimC | 1041 | ECJB195_5464 | EIH87844 | gram-negative pili assembly chaperone, N-terminal domain protein | Escherichia coli JB1-95 | 657.7 | 4.70E-194 |
| FimC | 1042 | BX93_03145 | EYY07755 | molecular chaperone FimC | Escherichia coli O111:NM str. 2011C-3170 | 657.7 | 4.70E-194 |
| FimC | 1043 | HMPREF1592_04024 | ESA74049 | gram-negative pili assembly chaperone domain protein | Escherichia coli 907357 | 657.7 | 4.70E-194 |
| FimC | 1044 | G759_04771 | ESJ98607 | chaperone fimC | Escherichia coli HVH 98 (4-5799287) | 657.7 | 4.70E-194 |
| FimC | 1045 | BX67_25095 | EYZ21900 | molecular chaperone FimC | Escherichia coli O145:NM str. 2010C-4557C2 | 657.7 | 4.70E-194 |
| FimC | 1046 | ECDEC15D_5000 | EHY09585 | gram-negative pili assembly chaperone, N-terminal domain protein | Escherichia coli DEC15D | 657.7 | 4.70E-194 |
| FimC | 1047 | CDK60033 | CDK60033 | chaperone FimC | Escherichia coli IS9 | 657.7 | 4.70E-194 |
| FimC | 1048 | BY05_09765 | EYX78481 | molecular chaperone FimC | Escherichia coli O111:NM str. 2011C-3632 | 657.7 | 4.70E-194 |
| FimC | 1049 | AC33_0310 | KDU01964 | chaperone protein fimC | Escherichia coli 3-267-03_S3_C2 | 657.7 | 4.70E-194 |
| FimC | 1050 | EC50588_4792 | EIH01564 | gram-negative pili assembly chaperone, N-terminal domain protein | Escherichia coli 5.0588 | 657.7 | 4.70E-194 |
| FimC | 1051 | ECP02994832_0104 | END84223 | chaperone protein fimC | Escherichia coli P0299483.2 | 657.7 | 4.70E-194 |
| FimC | 1052 | ESOG_00110 | EHO03072 | chaperone fimC | Escherichia coli E101 | 657.7 | 4.70E-194 |
| FimC | 1053 | WAY_04397 | EOU27303 | fimbrial chaperone FimC | Escherichia coli KTE13 | 657.7 | 4.70E-194 |
| FimC | 1054 | L667_09990 | ERE04308 | molecular chaperone FimC | Escherichia coli 95JB1 | 657.7 | 4.70E-194 |
| FimC | 1055 | ECC1470_03189 | EST87066 | chaperone FimC, periplasmic | Escherichia coli ECC-1470 (GCA_000831565) | 657.7 | 4.70E-194 |
| FimC | 1056 | T22_006292 | ENO10387 | chaperone FimC, periplasmic | Escherichia coli O157:H43 str. T22 | 657.7 | 4.70E-194 |
| FimC | 1057 | BX49_04000 | EYV18152 | molecular chaperone FimC | Escherichia coli O145:NM str. 2010C-3518 | 657.7 | 4.70E-194 |
| FimC | 1058 | ECDEC15A_5302 | EHX92018 | gram-negative pili assembly chaperone, N-terminal domain protein | Escherichia coli DEC15A | 657.7 | 4.70E-194 |
| FimC | 1059 | ECDEC9A_5609 | EHW28645 | gram-negative pili assembly chaperone, N-terminal domain protein | Escherichia coli DEC9A | 657.7 | 4.70E-194 |
| FimC | 1060 | ERBG_01516 | EGC12412 | gram-negative pili assembly chaperone domain-containing protein | Escherichia coli E1167 | 657.7 | 4.70E-194 |
| FimC | 1061 | AB76_1913 | KDT62509 | chaperone protein fimC | Escherichia coli 3-267-03_S1_C3 | 657.7 | 4.70E-194 |
| FimC | 1062 | BX10_01845 | EZE16032 | molecular chaperone FimC | Escherichia coli O121:H7 str. 2009C-3299 | 657.7 | 4.70E-194 |
| FimC | 1063 | BX45_00380 | EYV46002 | molecular chaperone FimC | Escherichia coli O145:NM str. 2010C-3510 | 657.7 | 4.70E-194 |
| FimC | 1064 | BX48_09430 | EYV22670 | molecular chaperone FimC | Escherichia coli O145:NM str. 2010C-3517 | 657.7 | 4.70E-194 |
| FimC | 1065 | ECMP0209802_0277 | EMX57011 | chaperone protein fimC | Escherichia coli MP020980.2 | 657.7 | 4.70E-194 |
| FimC | 1066 | ECPG_01545 | EGI43218 | periplasmic chaperone, required for type 1 fimbriae | Escherichia coli H591 | 657.7 | 4.70E-194 |
| FimC | 1067 | WCQ_04449 | ELC17022 | chaperone fimC | Escherichia coli KTE12 | 657.7 | 4.70E-194 |
| FimC | 1068 | BW88_24055 | EYZ59763 | molecular chaperone FimC | Escherichia coli O79:H7 str. 06-3501 | 657.7 | 4.70E-194 |
| FimC | 1069 | WK1_04330 | ELI74404 | fimbrial chaperone FimC | Escherichia coli KTE138 | 657.7 | 4.70E-194 |
| FimC | 1070 | ECBCE002MS12_4674 | EMV29130 | chaperone protein fimC | Escherichia coli BCE002_MS12 | 657.7 | 4.70E-194 |
| FimC | 1071 | AB33_3673 | KDY24739 | chaperone protein fimC | Escherichia coli 2-427-07_S1_C2 | 657.7 | 4.70E-194 |
| FimC | 1072 | SSJG_02833 | EGJ06784 | gram-negative pili assembly chaperone domain protein | Escherichia coli D9 | 657.7 | 4.70E-194 |
| FimC | 1073 | HMPREF1609_05021 | ESD66400 | gram-negative pili assembly chaperone domain protein | Escherichia coli 908541 | 657.7 | 4.70E-194 |
| FimC | 1074 | ECDEC15C_5064 | EHY01773 | gram-negative pili assembly chaperone, N-terminal domain protein | Escherichia coli DEC15C | 657.7 | 4.70E-194 |
| FimC | 1075 | A1WA_04479 | ELG38552 | fimbrial chaperone FimC | Escherichia coli KTE91 | 657.7 | 4.70E-194 |
| FimC | 1076 | A1UQ_00297 | ELE66676 | chaperone fimC | Escherichia coli KTE77 | 657.7 | 4.70E-194 |
| FimC | 1077 | EC2864350_4662 | ENA89897 | chaperone protein fimC | Escherichia coli 2864350 | 657.7 | 4.70E-194 |
| FimC | 1078 | HMPREF9346_03626 | EFK44739 | gram-negative pili assembly chaperone domain protein | Escherichia coli MS 119-7 | 657.7 | 4.70E-194 |
| FimC | 1079 | BX50_13350 | EYV16917 | molecular chaperone FimC | Escherichia coli O145:NM str. 2010C-3521 | 657.7 | 4.70E-194 |
| FimC | 1080 | ESTG_04077 | EIG42816 | chaperone fimC | Escherichia coli B799 | 657.7 | 4.70E-194 |
| FimC | 1081 | AB04_3854 | KDZ93249 | chaperone protein fimC | Escherichia coli 2-427-07_S1_C1 | 657.7 | 4.70E-194 |
| FimC | 1082 | BX02_26890 | EYW91333 | molecular chaperone FimC | Escherichia coli O145:NM str. 08-4270 | 657.7 | 4.70E-194 |
| FimC | 1083 | A1S5_00743 | ELG02226 | fimbrial chaperone FimC | Escherichia coli KTE48 | 657.7 | 4.70E-194 |
| FimC | 1084 | L668_08210 | ERA59257 | molecular chaperone FimC | Escherichia coli 95NR1 | 657.7 | 4.70E-194 |
| FimC | 1085 | HMPREF1598_03566 | ESD18257 | gram-negative pili assembly chaperone domain protein | Escherichia coli 907710 | 657.7 | 4.70E-194 |
| FimC | 1086 | ECDEC15B_5183 | EHX98950 | gram-negative pili assembly chaperone, N-terminal domain protein | Escherichia coli DEC15B | 657.7 | 4.70E-194 |
| FimC | 1087 | BX43_26710 | EZE91452 | molecular chaperone FimC | Escherichia coli O145:NM str. 2010C-3508 | 657.7 | 4.70E-194 |
| FimC | 1088 | AB64_2975 | KDY91234 | chaperone protein fimC | Escherichia coli 2-427-07_S1_C3 | 657.7 | 4.70E-194 |
| FimC | 1089 | HMPREF9345_03565 | EFK49953 | gram-negative pili assembly chaperone domain protein | Escherichia coli MS 107-1 | 657.7 | 4.70E-194 |
| FimC | 1090 | ERS139235_01095 | CTX12065 | type I fimbrial chaperone | Escherichia coli O111:H- (strain 11128 / EHEC) | 657.7 | 4.70E-194 |
| FimC | 1091 | G794_04670 | ESP12243 | chaperone fimC | Escherichia coli HVH 136 (4-5970458) | 657.6 | 4.90E-194 |
| FimC | 1092 | WIO_04637 | ELI49037 | fimbrial chaperone FimC | Escherichia coli KTE125 | 657.6 | 4.90E-194 |
| FimC | 1093 | AB32_5197 | KEJ21014 | chaperone protein fimC | Escherichia coli 2-316-03_S1_C2 | 657.6 | 4.90E-194 |
| FimC | 1094 | AC12_5133 | KDA70941 | chaperone protein fimC | Escherichia coli 2-005-03_S3_C2 | 657.6 | 4.90E-194 |
| FimC | 1095 | A15E_00393 | ELH63207 | fimbrial chaperone FimC | Escherichia coli KTE202 | 657.6 | 4.90E-194 |
| FimC | 1096 | A1S9_01535 | ELG08173 | fimbrial chaperone FimC | Escherichia coli KTE50 | 657.6 | 4.90E-194 |
| FimC | 1097 | G916_04583 | ERB08821 | chaperone fimC | Escherichia coli UMEA 3144-1 | 657.6 | 4.90E-194 |
| FimC | 1098 | G718_04593 | EQO57409 | chaperone fimC | Escherichia coli HVH 43 (4-2173468) | 657.6 | 4.90E-194 |
| FimC | 1099 | G719_04640 | EQO66420 | chaperone fimC | Escherichia coli HVH 44 (4-2298570) | 657.6 | 4.90E-194 |
| FimC | 1100 | G834_04638 | EQT31756 | chaperone fimC | Escherichia coli HVH 182 (4-0985554) | 657.6 | 4.90E-194 |
| FimC | 1101 | AO733_23580 | KRV65188 | molecular chaperone FimC | Escherichia coli | 657.6 | 5.10E-194 |
| FimC | 1102 | A1Y9_04271 | EOW25281 | fimbrial chaperone FimC | Escherichia coli KTE121 | 657.5 | 5.40E-194 |
| FimC | 1103 | G857_04825 | EQU41946 | chaperone fimC | Escherichia coli HVH 205 (4-3094677) | 657.4 | 5.80E-194 |
| FimC | 1104 | G746_04712 | EQP84600 | chaperone fimC | Escherichia coli HVH 84 (4-1021478) | 657.4 | 5.80E-194 |
| FimC | 1105 | OO96_18485 | OAO67080 | molecular chaperone FimC | Escherichia coli (GCA_001651605) | 657.3 | 6.10E-194 |
| FimC | 1106 | ESNG_01159 | EHO01967 | chaperone fimC | Escherichia coli B093 | 657.1 | 6.90E-194 |
| FimC | 1107 | AXK33_12285 | OAF42350 | molecular chaperone FimC | Escherichia coli | 657.1 | 6.90E-194 |
| FimC | 1108 | AD871_23995 | AMN65286 | molecular chaperone FimC | Shigella flexneri 4c | 657.1 | 6.90E-194 |
| FimC | 1109 | SFyv_6110 | AIL43530 | periplasmic chaperone | Shigella flexneri Shi06HN006 | 657.1 | 6.90E-194 |
| FimC | 1110 | S4463 | AAP19411 | periplasmic chaperone | Shigella flexneri 2a str. 2457T (GCA_000183785) | 657.1 | 6.90E-194 |
| FimC | 1111 | SFK304_5238 | EGK31452 | chaperone protein fimC | Shigella flexneri K-304 | 657.1 | 6.90E-194 |
| FimC | 1112 | SFy_6041 | AIL38593 | periplasmic chaperone | Shigella flexneri 2003036 | 657.1 | 6.90E-194 |
| FimC | 1113 | ERS574920_03327 | CEP58466 | periplasmic chaperone | Shigella flexneri 2a | 657.1 | 6.90E-194 |
| FimC | 1114 | SF660363_4844 | EJL09931 | gram-negative pili assembly chaperone, N-terminal domain protein | Shigella flexneri 6603-63 | 657.1 | 6.90E-194 |
| FimC | 1115 | SF4206 | AAN45626 | periplasmic chaperone | Shigella flexneri 2a str. 301 | 657.1 | 6.90E-194 |
| FimC | 1116 | SF274771_5016 | EGJ80856 | chaperone protein fimC | Shigella flexneri 2747-71 | 657.1 | 6.90E-194 |
| FimC | 1117 | NCTC1_04572 | CDX09635 | periplasmic chaperone,Chaperone protein fimC precursor,fimbrial chaperone protein FimC,Gram-negative pili assembly chaperone, N-terminal domain | Shigella flexneri | 657.1 | 6.90E-194 |
| FimC | 1118 | SFxv_4589 | ADA76502 | Type I fimbrial chaperone | Shigella flexneri 2002017 | 657.1 | 6.90E-194 |
| FimC | 1119 | SFK404_5375 | EIQ19702 | chaperone protein fimC | Shigella flexneri K-404 | 657.1 | 6.90E-194 |
| FimC | 1120 | SFK671_3943 | EGJ82498 | chaperone protein fimC | Shigella flexneri K-671 | 657.1 | 6.90E-194 |
| FimC | 1121 | ECMP0215612_0299 | EMX33699 | chaperone protein fimC | Escherichia coli MP021561.2 | 657 | 7.50E-194 |
| FimC | 1122 | EC2866550_4961 | EMV66032 | chaperone protein fimC | Escherichia coli 2866550 | 657 | 7.70E-194 |
| FimC | 1123 | EC2875000_4922 | EMV33037 | chaperone protein fimC | Escherichia coli 2875000 | 657 | 7.70E-194 |
| FimC | 1124 | EC2851500_4845 | EMV96310 | chaperone protein fimC | Escherichia coli 2851500 | 657 | 7.70E-194 |
| FimC | 1125 | EC2850750_4917 | EMW00352 | chaperone protein fimC | Escherichia coli 2850750 | 657 | 7.70E-194 |
| FimC | 1126 | EC180200_4705 | ENA60805 | chaperone protein fimC | Escherichia coli 180200 | 657 | 7.70E-194 |
| FimC | 1127 | EC2850400_4935 | EMW13084 | chaperone protein fimC | Escherichia coli 2850400 | 657 | 7.70E-194 |
| FimC | 1128 | EC2853500_4879 | EMV96221 | chaperone protein fimC | Escherichia coli 2853500 | 657 | 7.70E-194 |
| FimC | 1129 | G872_04323 | EQV01911 | chaperone fimC | Escherichia coli HVH 221 (4-3136817) | 656.8 | 8.60E-194 |
| FimC | 1130 | ECS88_4934 | CAR06078 | chaperone, periplasmic | Escherichia coli S88 | 656.8 | 8.60E-194 |
| FimC | 1131 | ERS085405_02505 | CTV96031 | type I fimbrial chaperone | Escherichia coli O45:K1 (strain S88 / ExPEC) | 656.8 | 8.60E-194 |
| FimC | 1132 | G843_04681 | EQT72237 | chaperone fimC | Escherichia coli HVH 191 (3-9341900) | 656.8 | 8.60E-194 |
| FimC | 1133 | ERS372666_04540 | CUK16590 | Chaperone protein fimC precursor | Achromobacter sp. ATCC35328 | 656.8 | 8.60E-194 |
| FimC | 1134 | G937_04522 | EQX79091 | chaperone fimC | Escherichia coli UMEA 3199-1 | 656.8 | 8.60E-194 |
| FimC | 1135 | G825_04863 | EQS96610 | chaperone fimC | Escherichia coli HVH 170 (4-3026949) | 656.8 | 8.60E-194 |
| FimC | 1136 | G753_04355 | EQQ13617 | chaperone fimC | Escherichia coli HVH 91 (4-4638751) | 656.8 | 8.60E-194 |
| FimC | 1137 | G999_04654 | ERA14022 | chaperone fimC | Escherichia coli UMEA 3893-1 | 656.8 | 8.60E-194 |
| FimC | 1138 | G917_04573 | ESP39529 | chaperone fimC | Escherichia coli UMEA 3148-1 | 656.8 | 8.60E-194 |
| FimC | 1139 | G925_04668 | EQX22125 | chaperone fimC | Escherichia coli (strain UMEA 3162-1) | 656.8 | 8.60E-194 |
| FimC | 1140 | WAW_00436 | EOU39656 | fimbrial chaperone FimC | Escherichia coli KTE7 | 656.8 | 8.60E-194 |
| FimC | 1141 | GECO_04394 | KFB91650 | FimC family chaperone | Escherichia coli DSM 30083 = JCM 1649 = ATCC 11775 | 656.8 | 8.60E-194 |
| FimC | 1142 | P411_21155 | EZD87849 | molecular chaperone FimC | Escherichia coli O39:NM str. F8704-2 | 656.8 | 8.60E-194 |
| FimC | 1143 | EcE110019_2183 | EDV87007 | chaperone protein FimC | Escherichia coli E110019 | 656.8 | 8.60E-194 |
| FimC | 1144 | WCE_04757 | ELC04839 | chaperone fimC | Escherichia coli KTE5 | 656.8 | 8.60E-194 |
| FimC | 1145 | G866_03446 | ETF28068 | chaperone fimC | Escherichia coli HVH 214 (4-3062198) | 656.8 | 8.60E-194 |
| FimC | 1146 | G869_04795 | EQU87570 | chaperone fimC | Escherichia coli HVH 217 (4-1022806) | 656.8 | 8.60E-194 |
| FimC | 1147 | N840_4399 | AGX36210 | periplasmic chaperone | synthetic Escherichia coli C321.deltaA | 656.8 | 8.80E-194 |
| FimC | 1148 | BAE78309 | BAE78309 | chaperone, periplasmic | Escherichia coli str. K-12 substr. W3110 | 656.8 | 8.80E-194 |
| FimC | 1149 | CS35_4371 | AKF74578 | periplasmic chaperone | Escherichia coli | 656.8 | 8.80E-194 |
| FimC | 1150 | b4316 | AAC77272 | periplasmic chaperone | Escherichia coli str. K-12 substr. MG1655 (GCA_000801205) | 656.8 | 8.80E-194 |
| FimC | 1151 | BN896_4014 | CDJ74533 | chaperone, periplasmic | Escherichia coli str. K-12 substr. MC4100 | 656.8 | 8.80E-194 |
| FimC | 1152 | IAE_10074 | EGU27031 | chaperone, periplasmic | Escherichia coli XH140A | 656.8 | 8.80E-194 |
| FimC | 1153 | OQE_35090 | EIE35469 | chaperone, periplasmic | Escherichia coli J53 | 656.8 | 8.80E-194 |
| FimC | 1154 | SH05_03240 | AKD59513 | molecular chaperone FimC | Escherichia coli (strain K12) | 656.8 | 8.80E-194 |
| FimC | 1155 | ECDH1ME8569_4174 | BAJ46030 | chaperone, periplasmic | Escherichia coli DH1 | 656.8 | 8.80E-194 |
| FimC | 1156 | L670_08819 | KGL70391 | chaperone, periplasmic | Escherichia coli NCTC 50110 | 656.8 | 8.80E-194 |
| FimC | 1157 | BW25113_4316 | AIN34590 | periplasmic chaperone | Escherichia coli BW25113 | 656.8 | 8.80E-194 |
| FimC | 1158 | HQ24_22210 | AIF39560 | molecular chaperone FimC | Escherichia coli KLY | 656.8 | 8.80E-194 |
| FimC | 1159 | BWG_4014 | ACR62139 | chaperone, periplasmic | Escherichia coli BW2952 | 656.8 | 8.80E-194 |
| FimC | 1160 | IAM_13804 | EGV47109 | chaperone, periplasmic | Escherichia coli XH001 | 656.8 | 8.80E-194 |
| FimC | 1161 | ECP030526012_4647 | ENF80714 | chaperone protein fimC | Escherichia coli P0305260.12 | 656.8 | 9.00E-194 |
| FimC | 1162 | ECP03052608_4692 | ENG20638 | chaperone protein fimC | Escherichia coli P0305260.8 | 656.8 | 9.00E-194 |
| FimC | 1163 | BY29_17755 | EYW39176 | molecular chaperone FimC | Escherichia coli O157:H7 str. 2011EL-2112 | 656.8 | 9.00E-194 |
| FimC | 1164 | BW92_08240 | EYZ52244 | molecular chaperone FimC | Escherichia coli O157:H7 str. 06-3745 | 656.8 | 9.00E-194 |
| FimC | 1165 | EC950943_0031 | EKW36594 | chaperone protein fimC | Escherichia coli 95.0943 | 656.8 | 9.00E-194 |
| FimC | 1166 | ECO5101_17246 | EFX08689 | Chaperone protein FimC | Escherichia coli O157:H7 str. G5101 | 656.8 | 9.00E-194 |
| FimC | 1167 | BY66_06820 | EZB87925 | molecular chaperone FimC | Escherichia coli O157:H7 str. K1921 | 656.8 | 9.00E-194 |
| FimC | 1168 | SS17_5469 | AIF96951 | chaperone FimC | Escherichia coli O157:H7 str. SS17 | 656.8 | 9.00E-194 |
| FimC | 1169 | BY32_04000 | EYW27196 | molecular chaperone FimC | Escherichia coli O157:H7 str. 2011EL-2286 | 656.8 | 9.00E-194 |
| FimC | 1170 | EC990672_5677 | EKW76235 | chaperone protein fimC | Escherichia coli 99.0672 | 656.8 | 9.00E-194 |
| FimC | 1171 | ECDEC3F_0091 | EHU97557 | gram-negative pili assembly chaperone, N-terminal domain protein | Escherichia coli DEC3F | 656.8 | 9.00E-194 |
| FimC | 1172 | ECH74115_5822 | ACI38431 | chaperone protein FimC | Escherichia coli O157:H7 str. EC4115 | 656.8 | 9.00E-194 |
| FimC | 1173 | BX22_21825 | EZE61434 | molecular chaperone FimC | Escherichia coli O157:H7 str. 2009C-4258 | 656.8 | 9.00E-194 |
| FimC | 1174 | BY88_08900 | EZC92785 | molecular chaperone FimC | Escherichia coli O157:H7 str. K5602 | 656.8 | 9.00E-194 |
| FimC | 1175 | BX90_01445 | EZQ50268 | molecular chaperone FimC | Escherichia coli O157: str. 2010EL-2045 | 656.8 | 9.00E-194 |
| FimC | 1176 | BY75_13295 | EZC30147 | molecular chaperone FimC | Escherichia coli O157:H7 str. K2854 | 656.8 | 9.00E-194 |
| FimC | 1177 | BY58_11995 | EZB46999 | molecular chaperone FimC | Escherichia coli O157:H7 str. H2498 | 656.8 | 9.00E-194 |
| FimC | 1178 | EC960939_5682 | EKW43924 | chaperone protein fimC | Escherichia coli 96.0939 | 656.8 | 9.00E-194 |
| FimC | 1179 | ECDEC4C_5594 | EHV00339 | gram-negative pili assembly chaperone, N-terminal domain protein | Escherichia coli DEC4C | 656.8 | 9.00E-194 |
| FimC | 1180 | BW99_13285 | EYX12313 | molecular chaperone FimC | Escherichia coli O157:H7 str. 08-3527 | 656.8 | 9.00E-194 |
| FimC | 1181 | XF37_26810 | KKF80414 | molecular chaperone FimC | Escherichia coli O157:H7 | 656.8 | 9.00E-194 |
| FimC | 1182 | ECDEC5A_5152 | EHV18517 | gram-negative pili assembly chaperone, N-terminal domain protein | Escherichia coli DEC5A | 656.8 | 9.00E-194 |
| FimC | 1183 | S13_0101 | ERC00120 | chaperone protein fimC | Escherichia coli B26-2 | 656.8 | 9.00E-194 |
| FimC | 1184 | ECoD_01984 | EFW65220 | chaperone FimC | Escherichia coli O157:H7 str. EC1212 | 656.8 | 9.00E-194 |
| FimC | 1185 | BY21_12430 | EYW75535 | molecular chaperone FimC | Escherichia coli O157:H7 str. 2011EL-2103 | 656.8 | 9.00E-194 |
| FimC | 1186 | EC950183_0019 | EKW36784 | chaperone protein fimC | Escherichia coli 95.0183 | 656.8 | 9.00E-194 |
| FimC | 1187 | ECPA7_0403 | EKH07698 | periplasmic chaperone required for type 1 fimbriae | Escherichia coli PA7 | 656.8 | 9.00E-194 |
| FimC | 1188 | ECFDA507_5681 | EKH13370 | periplasmic chaperone required for type 1 fimbriae | Escherichia coli FDA507 | 656.8 | 9.00E-194 |
| FimC | 1189 | ECTT12B_0033 | EKH95584 | periplasmic chaperone required for type 1 fimbriae | Escherichia coli TT12B | 656.8 | 9.00E-194 |
| FimC | 1190 | EC71982_0041 | ELW21822 | chaperone protein fimC | Escherichia coli 7.1982 | 656.8 | 9.00E-194 |
| FimC | 1191 | EC960109_5701 | EKY34551 | chaperone protein fimC | Escherichia coli 96.0109 | 656.8 | 9.00E-194 |
| FimC | 1192 | BY14_02845 | EYX44867 | molecular chaperone FimC | Escherichia coli O157:H7 str. 2011EL-2093 | 656.8 | 9.00E-194 |
| FimC | 1193 | ECEC96038_5570 | EKI03004 | periplasmic chaperone required for type 1 fimbriae | Escherichia coli EC96038 | 656.8 | 9.00E-194 |
| FimC | 1194 | BY45_26415 | EZA89737 | molecular chaperone FimC | Escherichia coli O157:H7 str. F6142 | 656.8 | 9.00E-194 |
| FimC | 1195 | ECEC4203_5688 | EIO87452 | periplasmic chaperone required for type 1 fimbriae | Escherichia coli EC4203 | 656.8 | 9.00E-194 |
| FimC | 1196 | ECTW14313_5569 | EIP03324 | periplasmic chaperone required for type 1 fimbriae | Escherichia coli O157:H7 str. TW14313 | 656.8 | 9.00E-194 |
| FimC | 1197 | BX05_07295 | EZD90137 | molecular chaperone FimC | Escherichia coli O157:NM str. 08-4540 | 656.8 | 9.00E-194 |
| FimC | 1198 | ECEC4196_5739 | EIO92435 | periplasmic chaperone required for type 1 fimbriae | Escherichia coli EC4196 | 656.8 | 9.00E-194 |
| FimC | 1199 | ECOSU61_14701 | EFX32605 | Chaperone protein FimC | Escherichia coli O157:H7 str. LSU-61 | 656.8 | 9.00E-194 |
| FimC | 1200 | QYE_0100 | ERB79233 | chaperone protein fimC | Escherichia coli B107 | 656.8 | 9.00E-194 |
| FimC | 1201 | EC900091_0099 | EKW05789 | chaperone protein fimC | Escherichia coli 90.0091 | 656.8 | 9.00E-194 |
| FimC | 1202 | BX36_01495 | EYV67662 | molecular chaperone FimC | Escherichia coli O157:H7 str. 2009EL2109 | 656.8 | 9.00E-194 |
| FimC | 1203 | BX83_07515 | EYY46564 | molecular chaperone FimC | Escherichia coli O157:H7 str. 2010C-4979C1 | 656.8 | 9.00E-194 |
| FimC | 1204 | BY85_22405 | EZC64781 | molecular chaperone FimC | Escherichia coli O157:H7 str. K5453 | 656.8 | 9.00E-194 |
| FimC | 1205 | ECDEC4B_5655 | EHU89524 | gram-negative pili assembly chaperone, N-terminal domain protein | Escherichia coli DEC4B | 656.8 | 9.00E-194 |
| FimC | 1206 | ECPA34_0032 | EKH20934 | periplasmic chaperone required for type 1 fimbriae | Escherichia coli PA34 | 656.8 | 9.00E-194 |
| FimC | 1207 | EC990814_5084 | ELV14502 | chaperone protein fimC | Escherichia coli 99.0814 | 656.8 | 9.00E-194 |
| FimC | 1208 | ECPA15_0033 | EIN85481 | periplasmic chaperone required for type 1 fimbriae | Escherichia coli PA15 | 656.8 | 9.00E-194 |
| FimC | 1209 | EC930055_5500 | EKW04250 | chaperone protein fimC | Escherichia coli 93.0055 | 656.8 | 9.00E-194 |
| FimC | 1210 | S35_0031 | ERD05037 | chaperone protein fimC | Escherichia coli B104 | 656.8 | 9.00E-194 |
| FimC | 1211 | BY83_24030 | EZC70436 | molecular chaperone FimC | Escherichia coli O157:H7 str. K5448 | 656.8 | 9.00E-194 |
| FimC | 1212 | EC990816_5168 | ELV30851 | chaperone protein fimC | Escherichia coli 99.0816 | 656.8 | 9.00E-194 |
| FimC | 1213 | ECEC1868_0034 | EKJ36043 | periplasmic chaperone required for type 1 fimbriae | Escherichia coli EC1868 | 656.8 | 9.00E-194 |
| FimC | 1214 | EC991753_5091 | ELV44353 | chaperone protein fimC | Escherichia coli 99.1753 | 656.8 | 9.00E-194 |
| FimC | 1215 | Z5914 | AAG59498 | periplasmic chaperone, required for type 1 fimbriae | Escherichia coli O157:H7 str. EDL933 (GCA_000732965) | 656.8 | 9.00E-194 |
| FimC | 1216 | EC34880_5147 | ELW28334 | chaperone protein fimC | Escherichia coli 3.4880 | 656.8 | 9.00E-194 |
| FimC | 1217 | BY09_01270 | EYX70074 | molecular chaperone FimC | Escherichia coli O157:H7 str. 2011EL-1107 | 656.8 | 9.00E-194 |
| FimC | 1218 | EC950083_5039 | ELW29968 | chaperone protein fimC | Escherichia coli 95.0083 | 656.8 | 9.00E-194 |
| FimC | 1219 | ESCCO14588_1210 | EEC29755 | chaperone protein FimC | Escherichia coli O157:H7 str. TW14588 | 656.8 | 9.00E-194 |
| FimC | 1220 | ECSP_5400 | ACT75079 | periplasmic chaperone, required for type 1 fimbriae | Escherichia coli O157:H7 str. TW14359 | 656.8 | 9.00E-194 |
| FimC | 1221 | ECFRIK523_5606 | EKJ48071 | periplasmic chaperone required for type 1 fimbriae | Escherichia coli FRIK523 | 656.8 | 9.00E-194 |
| FimC | 1222 | ECEC4439_5636 | EIP35309 | periplasmic chaperone required for type 1 fimbriae | Escherichia coli EC4439 | 656.8 | 9.00E-194 |
| FimC | 1223 | BY92_10560 | EZD08954 | molecular chaperone FimC | Escherichia coli O157:H7 str. K5852 | 656.8 | 9.00E-194 |
| FimC | 1224 | ECTW09195_5727 | EIO86196 | periplasmic chaperone required for type 1 fimbriae | Escherichia coli TW09195 | 656.8 | 9.00E-194 |
| FimC | 1225 | ECEC1737_5620 | EKI59041 | periplasmic chaperone required for type 1 fimbriae | Escherichia coli EC1737 | 656.8 | 9.00E-194 |
| FimC | 1226 | BY87_00155 | EZC90347 | molecular chaperone FimC | Escherichia coli O157:H7 str. K5467 | 656.8 | 9.00E-194 |
| FimC | 1227 | BY24_25830 | EYW64034 | molecular chaperone FimC | Escherichia coli O157:H7 str. 2011EL-2106 | 656.8 | 9.00E-194 |
| FimC | 1228 | ECEC1864_0032 | EKJ19842 | periplasmic chaperone required for type 1 fimbriae | Escherichia coli EC1864 | 656.8 | 9.00E-194 |
| FimC | 1229 | ECH7EC4076_2475 | EDU68733 | chaperone protein FimC | Escherichia coli O157:H7 str. EC4076 | 656.8 | 9.00E-194 |
| FimC | 1230 | B232_0152 | ERE42132 | chaperone protein fimC | Escherichia coli Tx1686 | 656.8 | 9.00E-194 |
| FimC | 1231 | ECEC1849_5555 | EKI82692 | periplasmic chaperone required for type 1 fimbriae | Escherichia coli EC1849 | 656.8 | 9.00E-194 |
| FimC | 1232 | ECTW07945_0026 | EIO79420 | periplasmic chaperone required for type 1 fimbriae | Escherichia coli TW07945 | 656.8 | 9.00E-194 |
| FimC | 1233 | ECT23400_5280 | ERC74204 | chaperone protein fimC | Escherichia coli T234_00 | 656.8 | 9.00E-194 |
| FimC | 1234 | ECPA38_5447 | EKI33211 | periplasmic chaperone required for type 1 fimbriae | Escherichia coli PA38 | 656.8 | 9.00E-194 |
| FimC | 1235 | BY77_00625 | EZC28538 | molecular chaperone FimC | Escherichia coli O157:H7 str. K4405 | 656.8 | 9.00E-194 |
| FimC | 1236 | BY18_15105 | EYX27775 | molecular chaperone FimC | Escherichia coli O157:H7 str. 2011EL-2098 | 656.8 | 9.00E-194 |
| FimC | 1237 | ECEC1738_0098 | EIP63548 | periplasmic chaperone required for type 1 fimbriae | Escherichia coli EC1738 | 656.8 | 9.00E-194 |
| FimC | 1238 | ECDEC5B_0022 | EHV37790 | gram-negative pili assembly chaperone, N-terminal domain protein | Escherichia coli DEC5B | 656.8 | 9.00E-194 |
| FimC | 1239 | BX34_16695 | EYV70470 | molecular chaperone FimC | Escherichia coli O157:H7 str. 2009EL1705 | 656.8 | 9.00E-194 |
| FimC | 1240 | EC930056_0031 | EKW19041 | chaperone protein fimC | Escherichia coli 93.0056 | 656.8 | 9.00E-194 |
| FimC | 1241 | BY34_23130 | EYW09891 | molecular chaperone FimC | Escherichia coli O157:H7 str. 2011EL-2288 | 656.8 | 9.00E-194 |
| FimC | 1242 | BY30_04750 | EYW34625 | molecular chaperone FimC | Escherichia coli O157:H7 str. 2011EL-2113 | 656.8 | 9.00E-194 |
| FimC | 1243 | ACP62_09070 | KOZ23175 | molecular chaperone FimC | Escherichia coli O55:H7 (strain CB9615 / EPEC) | 656.8 | 9.00E-194 |
| FimC | 1244 | ECATCC700728_5058 | ELV63276 | chaperone protein fimC | Escherichia coli ATCC 700728 | 656.8 | 9.00E-194 |
| FimC | 1245 | QYI_5235 | ERC30078 | chaperone protein fimC | Escherichia coli B7-2 | 656.8 | 9.00E-194 |
| FimC | 1246 | BY61_02750 | EZB59820 | molecular chaperone FimC | Escherichia coli O157:H7 str. K1792 | 656.8 | 9.00E-194 |
| FimC | 1247 | BY57_07565 | EZB41538 | molecular chaperone FimC | Escherichia coli O157:H7 str. H2495 | 656.8 | 9.00E-194 |
| FimC | 1248 | BY11_12745 | EYX57429 | molecular chaperone FimC | Escherichia coli O157:H7 str. 2011EL-2090 | 656.8 | 9.00E-194 |
| FimC | 1249 | ECPA5_5566 | EIN52284 | periplasmic chaperone required for type 1 fimbriae | Escherichia coli PA5 | 656.8 | 9.00E-194 |
| FimC | 1250 | ECEC1850_0048 | EKJ05449 | periplasmic chaperone required for type 1 fimbriae | Escherichia coli EC1850 | 656.8 | 9.00E-194 |
| FimC | 1251 | BY17_20285 | EYX30682 | molecular chaperone FimC | Escherichia coli O157:H7 str. 2011EL-2097 | 656.8 | 9.00E-194 |
| FimC | 1252 | ECEC4437_5758 | EIP49008 | periplasmic chaperone required for type 1 fimbriae | Escherichia coli EC4437 | 656.8 | 9.00E-194 |
| FimC | 1253 | BY90_06605 | EZD00253 | molecular chaperone FimC | Escherichia coli O157:H7 str. K5609 | 656.8 | 9.00E-194 |
| FimC | 1254 | S3C_0034 | ERD20764 | chaperone protein fimC | Escherichia coli B105 | 656.8 | 9.00E-194 |
| FimC | 1255 | BY48_01445 | EZB05960 | molecular chaperone FimC | Escherichia coli O157:H7 str. F6749 | 656.8 | 9.00E-194 |
| FimC | 1256 | ECFDA506_0356 | EKH25303 | periplasmic chaperone required for type 1 fimbriae | Escherichia coli FDA506 | 656.8 | 9.00E-194 |
| FimC | 1257 | ECPA32_5614 | EIO07283 | periplasmic chaperone required for type 1 fimbriae | Escherichia coli PA32 | 656.8 | 9.00E-194 |
| FimC | 1258 | B231_0101 | ERE45427 | chaperone protein fimC | Escherichia coli Tx3800 | 656.8 | 9.00E-194 |
| FimC | 1259 | ECT128201_5101 | ERE14345 | chaperone protein fimC | Escherichia coli T1282_01 | 656.8 | 9.00E-194 |
| FimC | 1260 | BY70_17590 | EZC05159 | molecular chaperone FimC | Escherichia coli O157:H7 str. K2192 | 656.8 | 9.00E-194 |
| FimC | 1261 | G2583_5117 | ADD59531 | Chaperone protein FimC | Escherichia coli O55:H7 str. CB9615 | 656.8 | 9.00E-194 |
| FimC | 1262 | BY84_03650 | EZC77676 | molecular chaperone FimC | Escherichia coli O157:H7 str. K5449 | 656.8 | 9.00E-194 |
| FimC | 1263 | BY68_02625 | EZB81709 | molecular chaperone FimC | Escherichia coli O157:H7 str. K2188 | 656.8 | 9.00E-194 |
| FimC | 1264 | EC881042_0032 | EKV85116 | chaperone protein fimC | Escherichia coli 88.1042 | 656.8 | 9.00E-194 |
| FimC | 1265 | ECEC4422_0030 | EIP33959 | periplasmic chaperone required for type 1 fimbriae | Escherichia coli EC4422 | 656.8 | 9.00E-194 |
| FimC | 1266 | EC991781_0019 | ELW23885 | chaperone protein fimC | Escherichia coli 99.1781 | 656.8 | 9.00E-194 |
| FimC | 1267 | EC5905_0089 | EKI02749 | periplasmic chaperone required for type 1 fimbriae | Escherichia coli 5905 | 656.8 | 9.00E-194 |
| FimC | 1268 | ECH7EC4113_5502 | EDU54273 | chaperone protein FimC | Escherichia coli O157:H7 str. EC4113 | 656.8 | 9.00E-194 |
| FimC | 1269 | QYS_4922 | ERC10677 | chaperone protein fimC | Escherichia coli B36-1 | 656.8 | 9.00E-194 |
| FimC | 1270 | BY65_09955 | EZB77234 | molecular chaperone FimC | Escherichia coli O157:H7 str. K1845 | 656.8 | 9.00E-194 |
| FimC | 1271 | QYC_0032 | ERB88991 | chaperone protein fimC | Escherichia coli B102 | 656.8 | 9.00E-194 |
| FimC | 1272 | ECDEC3C_0110 | EHU79280 | gram-negative pili assembly chaperone, N-terminal domain protein | Escherichia coli DEC3C | 656.8 | 9.00E-194 |
| FimC | 1273 | EC960428_0033 | EKW51274 | chaperone protein fimC | Escherichia coli 96.0428 | 656.8 | 9.00E-194 |
| FimC | 1274 | ECoA_01156 | EGD70507 | chaperone FimC | Escherichia coli O157:H7 str. 1044 | 656.8 | 9.00E-194 |
| FimC | 1275 | S3G_0033 | ERD37201 | chaperone protein fimC | Escherichia coli B112 | 656.8 | 9.00E-194 |
| FimC | 1276 | BAB38698 | BAB38698 | periplasmic chaperone required for type 1 fimbriae | Escherichia coli O157:H7 str. Sakai | 656.8 | 9.00E-194 |
| FimC | 1277 | ECPA23_5622 | EKH63866 | periplasmic chaperone required for type 1 fimbriae | Escherichia coli PA23 | 656.8 | 9.00E-194 |
| FimC | 1278 | ECDEC3D_5455 | EHU68124 | gram-negative pili assembly chaperone, N-terminal domain protein | Escherichia coli DEC3D | 656.8 | 9.00E-194 |
| FimC | 1279 | BY28_12310 | EYW43034 | molecular chaperone FimC | Escherichia coli O157:H7 str. 2011EL-2111 | 656.8 | 9.00E-194 |
| FimC | 1280 | ECPA25_5448 | EIN89869 | periplasmic chaperone required for type 1 fimbriae | Escherichia coli PA25 | 656.8 | 9.00E-194 |
| FimC | 1281 | EC970007_5015 | EKW71388 | chaperone protein fimC | Escherichia coli 97.0007 | 656.8 | 9.00E-194 |
| FimC | 1282 | BY36_24460 | EZF08546 | molecular chaperone FimC | Escherichia coli O157:H7 str. 2011EL-2290 | 656.8 | 9.00E-194 |
| FimC | 1283 | BY37_09435 | EYW07447 | molecular chaperone FimC | Escherichia coli O157:H7 str. 2011EL-2312 | 656.8 | 9.00E-194 |
| FimC | 1284 | ECO2687_22264 | EFX18254 | Chaperone protein FimC | Escherichia coli O157:H- str. H 2687 | 656.8 | 9.00E-194 |
| FimC | 1285 | BY94_01445 | EZD15743 | molecular chaperone FimC | Escherichia coli O157:H7 str. K6676 | 656.8 | 9.00E-194 |
| FimC | 1286 | ECF_01260 | EGD69115 | chaperone FimC | Escherichia coli O157:H7 str. 1125 | 656.8 | 9.00E-194 |
| FimC | 1287 | ECPA42_0033 | EIO49133 | periplasmic chaperone required for type 1 fimbriae | Escherichia coli PA42 | 656.8 | 9.00E-194 |
| FimC | 1288 | BY31_07800 | EYW25798 | molecular chaperone FimC | Escherichia coli O157:H7 str. 2011EL-2114 | 656.8 | 9.00E-194 |
| FimC | 1289 | ECH7EC4206_A4035 | EDZ77048 | chaperone protein FimC | Escherichia coli O157:H7 str. EC4206 | 656.8 | 9.00E-194 |
| FimC | 1290 | ECPA40_0105 | EIO32413 | periplasmic chaperone required for type 1 fimbriae | Escherichia coli PA40 | 656.8 | 9.00E-194 |
| FimC | 1291 | BY54_16065 | EZB30335 | molecular chaperone FimC | Escherichia coli O157:H7 str. F7410 | 656.8 | 9.00E-194 |
| FimC | 1292 | BY35_00440 | EYW13516 | molecular chaperone FimC | Escherichia coli O157:H7 str. 2011EL-2289 | 656.8 | 9.00E-194 |
| FimC | 1293 | BY91_01455 | EYV84709 | molecular chaperone FimC | Escherichia coli O157:H7 str. K5806 | 656.8 | 9.00E-194 |
| FimC | 1294 | ECEC1863_5355 | EIP72943 | periplasmic chaperone required for type 1 fimbriae | Escherichia coli EC1863 | 656.8 | 9.00E-194 |
| FimC | 1295 | BY50_12640 | EZB11551 | molecular chaperone FimC | Escherichia coli O157:H7 str. F6751 | 656.8 | 9.00E-194 |
| FimC | 1296 | BY76_10735 | EZC37631 | molecular chaperone FimC | Escherichia coli O157:H7 str. K4396 | 656.8 | 9.00E-194 |
| FimC | 1297 | ECTW10246_0277 | EIO63290 | periplasmic chaperone required for type 1 fimbriae | Escherichia coli TW10246 | 656.8 | 9.00E-194 |
| FimC | 1298 | BY49_01630 | EZB04389 | molecular chaperone FimC | Escherichia coli O157:H7 str. F6750 | 656.8 | 9.00E-194 |
| FimC | 1299 | ECEC1734_0033 | EIP71295 | periplasmic chaperone required for type 1 fimbriae | Escherichia coli EC1734 | 656.8 | 9.00E-194 |
| FimC | 1300 | QYG_5329 | ERC25327 | chaperone protein fimC | Escherichia coli B7-1 | 656.8 | 9.00E-194 |
| FimC | 1301 | BY33_00440 | EYW24525 | molecular chaperone FimC | Escherichia coli O157:H7 str. 2011EL-2287 | 656.8 | 9.00E-194 |
| FimC | 1302 | S1M_0019 | ERE39714 | chaperone protein fimC | Escherichia coli B90 | 656.8 | 9.00E-194 |
| FimC | 1303 | S37_0033 | ERD35175 | chaperone protein fimC | Escherichia coli B109 | 656.8 | 9.00E-194 |
| FimC | 1304 | S11_0098 | ERB91934 | chaperone protein fimC | Escherichia coli B26-1 | 656.8 | 9.00E-194 |
| FimC | 1305 | ECEC1862_5616 | EKJ00659 | periplasmic chaperone required for type 1 fimbriae | Escherichia coli EC1862 | 656.8 | 9.00E-194 |
| FimC | 1306 | BY74_13505 | EZC25633 | molecular chaperone FimC | Escherichia coli O157:H7 str. K2845 | 656.8 | 9.00E-194 |
| FimC | 1307 | EC991762_0032 | ELW27724 | chaperone protein fimC | Escherichia coli 99.1762 | 656.8 | 9.00E-194 |
| FimC | 1308 | ECH7EC869_5304 | EDU93190 | chaperone protein FimC | Escherichia coli O157:H7 str. EC869 | 656.8 | 9.00E-194 |
| FimC | 1309 | BY27_03920 | EYW49469 | molecular chaperone FimC | Escherichia coli O157:H7 str. 2011EL-2109 | 656.8 | 9.00E-194 |
| FimC | 1310 | ECO5905_15028 | EFX28092 | Chaperone protein FimC | Escherichia coli O55:H7 str. USDA 5905 | 656.8 | 9.00E-194 |
| FimC | 1311 | EC881467_0027 | EKV88783 | chaperone protein fimC | Escherichia coli 88.1467 | 656.8 | 9.00E-194 |
| FimC | 1312 | ECT92401_0023 | ERC92386 | chaperone protein fimC | Escherichia coli T924_01 | 656.8 | 9.00E-194 |
| FimC | 1313 | BY51_15800 | EYV89271 | molecular chaperone FimC | Escherichia coli O157:H7 str. F7350 | 656.8 | 9.00E-194 |
| FimC | 1314 | BY79_01450 | EZC48734 | molecular chaperone FimC | Escherichia coli O157:H7 str. K4527 | 656.8 | 9.00E-194 |
| FimC | 1315 | ECH7EC4486_2034 | EDU82739 | chaperone protein FimC | Escherichia coli O157:H7 str. EC4486 | 656.8 | 9.00E-194 |
| FimC | 1316 | EC940618_5452 | EKW08504 | chaperone protein fimC | Escherichia coli 94.0618 | 656.8 | 9.00E-194 |
| FimC | 1317 | BY26_11420 | EYW49156 | molecular chaperone FimC | Escherichia coli O157:H7 str. 2011EL-2108 | 656.8 | 9.00E-194 |
| FimC | 1318 | BW96_01440 | EYZ36203 | molecular chaperone FimC | Escherichia coli O157:H7 str. 07-3391 | 656.8 | 9.00E-194 |
| FimC | 1319 | ECPA48_4933 | ELV93057 | chaperone protein fimC | Escherichia coli PA48 | 656.8 | 9.00E-194 |
| FimC | 1320 | ECH7EC508_2468 | EDU97899 | chaperone protein FimC | Escherichia coli O157:H7 str. EC508 | 656.8 | 9.00E-194 |
| FimC | 1321 | ECCB7326_5678 | EKH96700 | periplasmic chaperone required for type 1 fimbriae | Escherichia coli CB7326 | 656.8 | 9.00E-194 |
| FimC | 1322 | BW98_01005 | EYX13642 | molecular chaperone FimC | Escherichia coli O157:H7 str. 08-3037 | 656.8 | 9.00E-194 |
| FimC | 1323 | S3A_0032 | ERD73339 | chaperone protein fimC | Escherichia coli B49-2 | 656.8 | 9.00E-194 |
| FimC | 1324 | BY72_11020 | EZC17271 | molecular chaperone FimC | Escherichia coli O157:H7 str. K2581 | 656.8 | 9.00E-194 |
| FimC | 1325 | ECH7EC4045_A0396 | EDZ81894 | chaperone protein FimC | Escherichia coli O157:H7 str. EC4045 | 656.8 | 9.00E-194 |
| FimC | 1326 | ECPA24_5479 | EIN88106 | periplasmic chaperone required for type 1 fimbriae | Escherichia coli PA24 | 656.8 | 9.00E-194 |
| FimC | 1327 | BX04_24445 | EZD85344 | molecular chaperone FimC | Escherichia coli O157:H7 str. 08-4529 | 656.8 | 9.00E-194 |
| FimC | 1328 | ECPA45_0032 | EKH87918 | periplasmic chaperone required for type 1 fimbriae | Escherichia coli PA45 | 656.8 | 9.00E-194 |
| FimC | 1329 | ECDEC4E_5299 | EHV06159 | gram-negative pili assembly chaperone, N-terminal domain protein | Escherichia coli DEC4E | 656.8 | 9.00E-194 |
| FimC | 1330 | BY89_20750 | EZC86967 | molecular chaperone FimC | Escherichia coli O157:H7 str. K5607 | 656.8 | 9.00E-194 |
| FimC | 1331 | B230_0104 | ERC91715 | chaperone protein fimC | Escherichia coli 14A | 656.8 | 9.00E-194 |
| FimC | 1332 | BY16_15960 | EYX32990 | molecular chaperone FimC | Escherichia coli O157:H7 str. 2011EL-2096 | 656.8 | 9.00E-194 |
| FimC | 1333 | S1E_5457 | ERC40358 | chaperone protein fimC | Escherichia coli B94 | 656.8 | 9.00E-194 |
| FimC | 1334 | BZ06_06590 | EZD75282 | molecular chaperone FimC | Escherichia coli O157:H7 str. K7140 | 656.8 | 9.00E-194 |
| FimC | 1335 | EC990815_5061 | ELV23610 | chaperone protein fimC | Escherichia coli 99.0815 | 656.8 | 9.00E-194 |
| FimC | 1336 | ECPA11_0031 | ELV75984 | chaperone protein fimC | Escherichia coli PA11 | 656.8 | 9.00E-194 |
| FimC | 1337 | EC960107_0031 | EKW74852 | chaperone protein fimC | Escherichia coli 96.0107 | 656.8 | 9.00E-194 |
| FimC | 1338 | ECO7815_09083 | EFX22747 | Chaperone protein FimC | Escherichia coli O55:H7 str. 3256-97 | 656.8 | 9.00E-194 |
| FimC | 1339 | S1O_5046 | ERD40249 | chaperone protein fimC | Escherichia coli B15 | 656.8 | 9.00E-194 |
| FimC | 1340 | BY67_04745 | EZB80121 | molecular chaperone FimC | Escherichia coli O157:H7 str. K1927 | 656.8 | 9.00E-194 |
| FimC | 1341 | BY95_02005 | EZD19474 | molecular chaperone FimC | Escherichia coli O157:H7 str. K6687 | 656.8 | 9.00E-194 |
| FimC | 1342 | ECFRIK920_5841 | EKG95801 | periplasmic chaperone required for type 1 fimbriae | Escherichia coli FRIK920 | 656.8 | 9.00E-194 |
| FimC | 1343 | BY22_04630 | EYW71165 | molecular chaperone FimC | Escherichia coli O157:H7 str. 2011EL-2104 | 656.8 | 9.00E-194 |
| FimC | 1344 | EC880221_0098 | EKK87684 | chaperone protein fimC | Escherichia coli 88.0221 | 656.8 | 9.00E-194 |
| FimC | 1345 | ECPA13_4917 | ELV76713 | chaperone protein fimC | Escherichia coli PA13 | 656.8 | 9.00E-194 |
| FimC | 1346 | ECH7EC4401_4307 | EDU76338 | chaperone protein FimC | Escherichia coli O157:H7 str. EC4401 | 656.8 | 9.00E-194 |
| FimC | 1347 | BY93_18425 | EZD17505 | molecular chaperone FimC | Escherichia coli O157:H7 str. K6590 | 656.8 | 9.00E-194 |
| FimC | 1348 | BY73_25955 | EZC08762 | molecular chaperone FimC | Escherichia coli O157:H7 str. K2622 | 656.8 | 9.00E-194 |
| FimC | 1349 | ECDEC4D_5336 | EHU99658 | gram-negative pili assembly chaperone, N-terminal domain protein | Escherichia coli DEC4D | 656.8 | 9.00E-194 |
| FimC | 1350 | BX33_22525 | EZE88820 | molecular chaperone FimC | Escherichia coli O157:H7 str. 2009EL1449 | 656.8 | 9.00E-194 |
| FimC | 1351 | S1U_0031 | ERD88007 | chaperone protein fimC | Escherichia coli B83 | 656.8 | 9.00E-194 |
| FimC | 1352 | Q455_0213280 | ETJ79069 | molecular chaperone FimC | Escherichia coli ATCC BAA-2192 | 656.8 | 9.00E-194 |
| FimC | 1353 | EC52239_0032 | EKK36933 | chaperone protein fimC | Escherichia coli 5.2239 | 656.8 | 9.00E-194 |
| FimC | 1354 | S1K_0034 | ERE36129 | chaperone protein fimC | Escherichia coli B89 | 656.8 | 9.00E-194 |
| FimC | 1355 | ECO9389_14668 | EFX13477 | Chaperone protein FimC | Escherichia coli O157:H- str. 493-89 | 656.8 | 9.00E-194 |
| FimC | 1356 | ECPA47_5048 | ELV91659 | chaperone protein fimC | Escherichia coli PA47 | 656.8 | 9.00E-194 |
| FimC | 1357 | ECEC1847_5634 | EKI72722 | periplasmic chaperone required for type 1 fimbriae | Escherichia coli EC1847 | 656.8 | 9.00E-194 |
| FimC | 1358 | EC09BKT76207_5484 | ERB68158 | chaperone protein fimC | Escherichia coli 09BKT076207 | 656.8 | 9.00E-194 |
| FimC | 1359 | BY82_00160 | EZC62941 | molecular chaperone FimC | Escherichia coli O157:H7 str. K5418 | 656.8 | 9.00E-194 |
| FimC | 1360 | BX89_02485 | EZQ47408 | molecular chaperone FimC | Escherichia coli O157: str. 2010EL-2044 | 656.8 | 9.00E-194 |
| FimC | 1361 | ECTW11039_0034 | EIO69675 | periplasmic chaperone required for type 1 fimbriae | Escherichia coli TW11039 | 656.8 | 9.00E-194 |
| FimC | 1362 | BW95_22290 | EYZ37430 | molecular chaperone FimC | Escherichia coli O157:H7 str. 07-3091 | 656.8 | 9.00E-194 |
| FimC | 1363 | ECEC4013_0095 | EIP38870 | periplasmic chaperone required for type 1 fimbriae | Escherichia coli EC4013 | 656.8 | 9.00E-194 |
| FimC | 1364 | BY56_10150 | EZB32029 | molecular chaperone FimC | Escherichia coli O157:H7 str. G5303 | 656.8 | 9.00E-194 |
| FimC | 1365 | BY12_10550 | EYX54534 | molecular chaperone FimC | Escherichia coli O157:H7 str. 2011EL-2091 | 656.8 | 9.00E-194 |
| FimC | 1366 | ECPA19_4662 | ELV78213 | chaperone protein fimC | Escherichia coli PA19 | 656.8 | 9.00E-194 |
| FimC | 1367 | B233_0032 | ERD09281 | chaperone protein fimC | Escherichia coli 2886-75 | 656.8 | 9.00E-194 |
| FimC | 1368 | BY64_13440 | EZB74704 | molecular chaperone FimC | Escherichia coli O157:H7 str. K1796 | 656.8 | 9.00E-194 |
| FimC | 1369 | BW94_20850 | EYZ38293 | molecular chaperone FimC | Escherichia coli O157:H7 str. 06-4039 | 656.8 | 9.00E-194 |
| FimC | 1370 | BY15_00150 | EYX44007 | molecular chaperone FimC | Escherichia coli O157:H7 str. 2011EL-2094 | 656.8 | 9.00E-194 |
| FimC | 1371 | EC82524_5566 | EKK52350 | chaperone protein fimC | Escherichia coli 8.2524 | 656.8 | 9.00E-194 |
| FimC | 1372 | BY59_23310 | EZB48652 | molecular chaperone FimC | Escherichia coli O157:H7 str. K1420 | 656.8 | 9.00E-194 |
| FimC | 1373 | ECFRIK2001_0279 | EKH63184 | periplasmic chaperone required for type 1 fimbriae | Escherichia coli FRIK2001 | 656.8 | 9.00E-194 |
| FimC | 1374 | ECNE1487_0150 | EKH53697 | periplasmic chaperone required for type 1 fimbriae | Escherichia coli NE1487 | 656.8 | 9.00E-194 |
| FimC | 1375 | EC990670_0033 | ELW46388 | chaperone protein fimC | Escherichia coli 99.0670 | 656.8 | 9.00E-194 |
| FimC | 1376 | QYO_0033 | ERC15171 | chaperone protein fimC | Escherichia coli B29-1 | 656.8 | 9.00E-194 |
| FimC | 1377 | ECT184097_4998 | ERC65840 | chaperone protein fimC | Escherichia coli T1840_97 | 656.8 | 9.00E-194 |
| FimC | 1378 | EC902281_0031 | EKW03186 | chaperone protein fimC | Escherichia coli 90.2281 | 656.8 | 9.00E-194 |
| FimC | 1379 | ECPA2_0032 | ELV98822 | chaperone protein fimC | Escherichia coli PA2 | 656.8 | 9.00E-194 |
| FimC | 1380 | BY71_01715 | EZB95428 | molecular chaperone FimC | Escherichia coli O157:H7 str. K2324 | 656.8 | 9.00E-194 |
| FimC | 1381 | QYQ_0017 | ERC23462 | chaperone protein fimC | Escherichia coli B29-2 | 656.8 | 9.00E-194 |
| FimC | 1382 | EC970003_0032 | EKW68030 | chaperone protein fimC | Escherichia coli 97.0003 | 656.8 | 9.00E-194 |
| FimC | 1383 | BY69_11245 | EZB98883 | molecular chaperone FimC | Escherichia coli O157:H7 str. K2191 | 656.8 | 9.00E-194 |
| FimC | 1384 | S3K_0033 | ERD52947 | chaperone protein fimC | Escherichia coli B114 | 656.8 | 9.00E-194 |
| FimC | 1385 | QYU_0031 | ERC32322 | chaperone protein fimC | Escherichia coli B36-2 | 656.8 | 9.00E-194 |
| FimC | 1386 | ECH74042_A0952 | EDZ88541 | chaperone protein FimC | Escherichia coli O157:H7 str. EC4042 | 656.8 | 9.00E-194 |
| FimC | 1387 | ECTW06591_5271 | EIO46235 | periplasmic chaperone required for type 1 fimbriae | Escherichia coli TW06591 | 656.8 | 9.00E-194 |
| FimC | 1388 | BY53_05125 | EZB18157 | molecular chaperone FimC | Escherichia coli O157:H7 str. F7384 | 656.8 | 9.00E-194 |
| FimC | 1389 | S3I_0019 | ERD44452 | chaperone protein fimC | Escherichia coli B113 | 656.8 | 9.00E-194 |
| FimC | 1390 | BX01_14855 | EYX02218 | molecular chaperone FimC | Escherichia coli O157:H7 str. 08-4169 | 656.8 | 9.00E-194 |
| FimC | 1391 | BY62_06930 | EZB55867 | molecular chaperone FimC | Escherichia coli O157:H7 str. K1793 | 656.8 | 9.00E-194 |
| FimC | 1392 | BY20_01445 | EYW83351 | molecular chaperone FimC | Escherichia coli O157:H7 str. 2011EL-2101 | 656.8 | 9.00E-194 |
| FimC | 1393 | BY63_06780 | EZB71562 | molecular chaperone FimC | Escherichia coli O157:H7 str. K1795 | 656.8 | 9.00E-194 |
| FimC | 1394 | BY25_21450 | EYW57162 | molecular chaperone FimC | Escherichia coli O157:H7 str. 2011EL-2107 | 656.8 | 9.00E-194 |
| FimC | 1395 | S1C_5444 | ERC33951 | chaperone protein fimC | Escherichia coli B93 | 656.8 | 9.00E-194 |
| FimC | 1396 | EC01304_5754 | EKJ55014 | periplasmic chaperone required for type 1 fimbriae | Escherichia coli 0.1304 | 656.8 | 9.00E-194 |
| FimC | 1397 | EC5412_5618 | EKI06078 | periplasmic chaperone required for type 1 fimbriae | Escherichia coli 5412 | 656.8 | 9.00E-194 |
| FimC | 1398 | ECPA8_0031 | ELW12441 | chaperone protein fimC | Escherichia coli PA8 | 656.8 | 9.00E-194 |
| FimC | 1399 | S1Q_4938 | ERD45145 | chaperone protein fimC | Escherichia coli B17 | 656.8 | 9.00E-194 |
| FimC | 1400 | BY38_12460 | EZF05467 | molecular chaperone FimC | Escherichia coli O157:H7 str. 2011EL-2313 | 656.8 | 9.00E-194 |
| FimC | 1401 | SS52_5682 | AJA29453 | chaperone FimC | Escherichia coli O157:H7 str. SS52 | 656.8 | 9.00E-194 |
| FimC | 1402 | S1I_0033 | ERD08429 | chaperone protein fimC | Escherichia coli B103 | 656.8 | 9.00E-194 |
| FimC | 1403 | BY52_09015 | EZB20606 | molecular chaperone FimC | Escherichia coli O157:H7 str. F7377 | 656.8 | 9.00E-194 |
| FimC | 1404 | ECH7EC4501_2830 | EDU87345 | chaperone protein FimC | Escherichia coli O157:H7 str. EC4501 | 656.8 | 9.00E-194 |
| FimC | 1405 | ECPA9_0031 | EIN68663 | periplasmic chaperone required for type 1 fimbriae | Escherichia coli PA9 | 656.8 | 9.00E-194 |
| FimC | 1406 | BY23_05530 | EYW67165 | molecular chaperone FimC | Escherichia coli O157:H7 str. 2011EL-2105 | 656.8 | 9.00E-194 |
| FimC | 1407 | ECEC1856_5607 | EKI92970 | periplasmic chaperone required for type 1 fimbriae | Escherichia coli EC1856 | 656.8 | 9.00E-194 |
| FimC | 1408 | BY78_01290 | EZC43997 | molecular chaperone FimC | Escherichia coli O157:H7 str. K4406 | 656.8 | 9.00E-194 |
| FimC | 1409 | BY13_13735 | EYX48120 | molecular chaperone FimC | Escherichia coli O157:H7 str. 2011EL-2092 | 656.8 | 9.00E-194 |
| FimC | 1410 | EC100833_0100 | EKK67958 | periplasmic chaperone required for type 1 fimbriae | Escherichia coli 10.0833 | 656.8 | 9.00E-194 |
| FimC | 1411 | EC991775_4977 | ELV47044 | chaperone protein fimC | Escherichia coli 99.1775 | 656.8 | 9.00E-194 |
| FimC | 1412 | ECDEC3B_5546 | EHU53045 | gram-negative pili assembly chaperone, N-terminal domain protein | Escherichia coli DEC3B | 656.8 | 9.00E-194 |
| FimC | 1413 | S1W_0033 | ERD91793 | chaperone protein fimC | Escherichia coli B84 | 656.8 | 9.00E-194 |
| FimC | 1414 | QYY_0033 | ERD83205 | chaperone protein fimC | Escherichia coli B5-2 | 656.8 | 9.00E-194 |
| FimC | 1415 | ECPA28_0033 | EIO09711 | periplasmic chaperone required for type 1 fimbriae | Escherichia coli PA28 | 656.8 | 9.00E-194 |
| FimC | 1416 | BY86_08095 | EZC83428 | molecular chaperone FimC | Escherichia coli O157:H7 str. K5460 | 656.8 | 9.00E-194 |
| FimC | 1417 | ECDEC4A_5331 | EHU86079 | gram-negative pili assembly chaperone, N-terminal domain protein | Escherichia coli DEC4A | 656.8 | 9.00E-194 |
| FimC | 1418 | BW89_03560 | EYZ63315 | molecular chaperone FimC | Escherichia coli O55:H7 str. 06-3555 | 656.8 | 9.00E-194 |
| FimC | 1419 | ECDEC3A_5320 | EHU52018 | gram-negative pili assembly chaperone, N-terminal domain protein | Escherichia coli DEC3A | 656.8 | 9.00E-194 |
| FimC | 1420 | ECFRIK1990_5781 | EIN35127 | periplasmic chaperone required for type 1 fimbriae | Escherichia coli FRIK1990 | 656.8 | 9.00E-194 |
| FimC | 1421 | ECEC1735_5665 | EKI46632 | periplasmic chaperone required for type 1 fimbriae | Escherichia coli EC1735 | 656.8 | 9.00E-194 |
| FimC | 1422 | ECPA49_0032 | EKH83471 | periplasmic chaperone required for type 1 fimbriae | Escherichia coli PA49 | 656.8 | 9.00E-194 |
| FimC | 1423 | BY19_06035 | EYW85318 | molecular chaperone FimC | Escherichia coli O157:H7 str. 2011EL-2099 | 656.8 | 9.00E-194 |
| FimC | 1424 | ECDEC3E_0019 | EHU85634 | gram-negative pili assembly chaperone, N-terminal domain protein | Escherichia coli DEC3E | 656.8 | 9.00E-194 |
| FimC | 1425 | ECTW10119_0286 | EIO86262 | periplasmic chaperone required for type 1 fimbriae | Escherichia coli TW10119 | 656.8 | 9.00E-194 |
| FimC | 1426 | ECNE098_0032 | EKJ53022 | periplasmic chaperone required for type 1 fimbriae | Escherichia coli NE098 | 656.8 | 9.00E-194 |
| FimC | 1427 | ECPA39_5719 | EIO24320 | periplasmic chaperone required for type 1 fimbriae | Escherichia coli PA39 | 656.8 | 9.00E-194 |
| FimC | 1428 | ECPA33_5594 | EIO11134 | periplasmic chaperone required for type 1 fimbriae | Escherichia coli PA33 | 656.8 | 9.00E-194 |
| FimC | 1429 | ECH7EC4196_1023 | EDU34080 | chaperone protein FimC | Escherichia coli O157:H7 str. EC4196 | 656.8 | 9.00E-194 |
| FimC | 1430 | BX35_01280 | EZE89463 | molecular chaperone FimC | Escherichia coli O157:H7 str. 2009EL1913 | 656.8 | 9.00E-194 |
| FimC | 1431 | S1Y_0033 | ERD98700 | chaperone protein fimC | Escherichia coli B85 | 656.8 | 9.00E-194 |
| FimC | 1432 | GS39_23920 | KFV35475 | molecular chaperone FimC | Escherichia coli (GCA_000749565) | 656.8 | 9.10E-194 |
| FimC | 1433 | EC960497_4782 | EIH33306 | gram-negative pili assembly chaperone, N-terminal domain protein | Escherichia coli 96.0497 | 656.8 | 9.10E-194 |
| FimC | 1434 | ECP02994384_5055 | ENB97740 | chaperone protein fimC | Escherichia coli P0299438.4 | 656.6 | 9.80E-194 |
| FimC | 1435 | ECP029943810_4841 | ENB84228 | chaperone protein fimC | Escherichia coli P0299438.10 | 656.6 | 9.80E-194 |
| FimC | 1436 | EC2016001_0330 | ENA25653 | chaperone protein fimC | Escherichia coli 201600.1 | 656.6 | 9.80E-194 |
| FimC | 1437 | ERS085382_04339 | CUA51274 | type I fimbrial chaperone | Escherichia coli | 656.6 | 9.90E-194 |
| FimC | 1438 | AM260_11150 | KQI85931 | molecular chaperone FimC | Escherichia coli (GCA_001419845) | 656.4 | 1.20E-193 |
| FimC | 1439 | G847_04319 | EQT88553 | chaperone fimC | Escherichia coli HVH 195 (3-7155360) | 656.2 | 1.30E-193 |
| FimC | 1440 | G778_04649 | EQR01024 | chaperone fimC | Escherichia coli HVH 116 (4-6879942) | 656.1 | 1.50E-193 |
| FimC | 1441 | PU51_03970 | KHH84711 | molecular chaperone FimC | Escherichia coli (GCA_000797655) | 656.1 | 1.50E-193 |
| FimC | 1442 | SFK272_0232 | EGK30691 | chaperone protein fimC | Shigella flexneri K-272 | 656 | 1.50E-193 |
| FimC | 1443 | SFK227_5267 | EGK31555 | chaperone protein fimC | Shigella flexneri K-227 | 656 | 1.50E-193 |
| FimC | 1444 | ERS139207_02565 | CTW10813 | type I fimbrial chaperone | Escherichia coli (GCA_001285965) | 656 | 1.60E-193 |
| FimC | 1445 | EC2845650_5146 | EMW27650 | chaperone protein fimC | Escherichia coli 2845650 | 655.9 | 1.60E-193 |
| FimC | 1446 | ECP03022936_4847 | ENE32382 | chaperone protein fimC | Escherichia coli P0302293.6 | 655.9 | 1.60E-193 |
| FimC | 1447 | ERYG_00373 | KNA43564 | gram-negative pili assembly chaperone domain-containing protein | Escherichia coli M114 | 655.9 | 1.60E-193 |
| FimC | 1448 | ECP03022933_4786 | ENE18962 | chaperone protein fimC | Escherichia coli P0302293.3 | 655.9 | 1.60E-193 |
| FimC | 1449 | ECDEC14A_4843 | EHX71036 | chaperone protein fimC | Escherichia coli DEC14A | 655.9 | 1.60E-193 |
| FimC | 1450 | ECSTEC94C_5155 | EGW77896 | chaperone protein fimC | Escherichia coli STEC_94C | 655.9 | 1.60E-193 |
| FimC | 1451 | BW71_19740 | EZA20104 | molecular chaperone FimC | Escherichia coli O113:H21 str. 07-4224 | 655.9 | 1.60E-193 |
| FimC | 1452 | EC96154_4684 | EIH99413 | gram-negative pili assembly chaperone, N-terminal domain protein | Escherichia coli 96.154 | 655.9 | 1.60E-193 |
| FimC | 1453 | AGA24_12020 | KNY55662 | molecular chaperone FimC | Escherichia coli | 655.9 | 1.60E-193 |
| FimC | 1454 | C827_04196 | EMZ38875 | chaperone fimC | Escherichia coli SWW33 | 655.9 | 1.60E-193 |
| FimC | 1455 | A1YY_04152 | ELG79327 | fimbrial chaperone FimC | Escherichia coli KTE144 | 655.8 | 1.70E-193 |
| FimC | 1456 | AC34_4562 | KDU07640 | chaperone protein fimC | Escherichia coli 3-373-03_S3_C2 | 655.7 | 1.90E-193 |
| FimC | 1457 | A319_00330 | EOW64289 | fimbrial chaperone FimC | Escherichia coli KTE155 | 655.7 | 1.90E-193 |
| FimC | 1458 | A31A_00660 | ELF23251 | chaperone fimC | Escherichia coli KTE156 | 655.7 | 1.90E-193 |
| FimC | 1459 | AC59_4542 | KDT71104 | chaperone protein fimC | Escherichia coli 3-373-03_S3_C3 | 655.7 | 1.90E-193 |
| FimC | 1460 | A155_00585 | ELH54109 | fimbrial chaperone FimC | Escherichia coli KTE197 | 655.7 | 1.90E-193 |
| FimC | 1461 | L444_09136 | ETY20383 | chaperone fimC | Escherichia coli BIDMC 15 | 655.7 | 1.90E-193 |
| FimC | 1462 | L432_08808 | ETY61288 | chaperone fimC | Escherichia coli BIDMC 6 | 655.7 | 1.90E-193 |
| FimC | 1463 | G822_04299 | EQS77298 | chaperone fimC | Escherichia coli HVH 164 (4-5953081) | 655.7 | 2.00E-193 |
| FimC | 1464 | WE7_00091 | EOU74395 | fimbrial chaperone FimC | Escherichia coli KTE20 | 655.7 | 2.00E-193 |
| FimC | 1465 | HMPREF1602_05058 | ESD31661 | gram-negative pili assembly chaperone domain protein | Escherichia coli 907889 | 655.7 | 2.00E-193 |
| FimC | 1466 | G966_05070 | ESK25393 | chaperone fimC | Escherichia coli UMEA 3323-1 | 655.7 | 2.00E-193 |
| FimC | 1467 | G701_04774 | EQN87714 | chaperone fimC | Escherichia coli HVH 25 (4-5851939) | 655.7 | 2.00E-193 |
| FimC | 1468 | SM09_00662 | KME75882 | chaperone FimC | Escherichia coli HVH 25 (4-5851939) | 655.7 | 2.00E-193 |
| FimC | 1469 | WE9_00121 | ELC44842 | chaperone fimC | Escherichia coli KTE21 | 655.7 | 2.00E-193 |
| FimC | 1470 | HMPREF1611_03513 | ESD82373 | gram-negative pili assembly chaperone domain protein | Escherichia coli 908573 | 655.6 | 2.00E-193 |
| FimC | 1471 | HMPREF9534_02256 | EFJ81713 | gram-negative pili assembly chaperone domain protein | Escherichia coli MS 69-1 | 655.6 | 2.00E-193 |
| FimC | 1472 | ECKD2_14387 | EIL49998 | type-1 fimbrial chaperone FimC | Escherichia coli KD2 | 655.6 | 2.00E-193 |
| FimC | 1473 | ERS085439_02136 | CTR92134 | type I fimbrial chaperone | Escherichia coli (GCA_001283745) | 655.2 | 2.60E-193 |
| FimC | 1474 | BW78_20145 | EYZ94309 | molecular chaperone FimC | Escherichia coli O174:H21 str. 03-3269 | 655.1 | 2.80E-193 |
| FimC | 1475 | HMPREF9350_02461 | EFU35805 | gram-negative pili assembly chaperone domain protein | Escherichia coli MS 85-1 | 655 | 3.00E-193 |
| FimC | 1476 | G783_04760 | EQR30148 | chaperone fimC | Escherichia coli HVH 121 (4-6877826) | 655 | 3.00E-193 |
| FimC | 1477 | G852_04869 | EQU18340 | chaperone fimC | Escherichia coli HVH 200 (4-4449924) | 655 | 3.00E-193 |
| FimC | 1478 | ECP03047774_4804 | ENE90359 | chaperone protein fimC | Escherichia coli P0304777.4 | 655 | 3.00E-193 |
| FimC | 1479 | ABW50_22695 | KOQ97696 | molecular chaperone FimC | Escherichia coli (GCA_001276265) | 655 | 3.00E-193 |
| FimC | 1480 | HMPREF9347_03726 | EFK67376 | gram-negative pili assembly chaperone domain protein | Escherichia coli MS 124-1 | 655 | 3.00E-193 |
| FimC | 1481 | G709_00501 | EQO36899 | chaperone fimC | Escherichia coli HVH 33 (4-2174936) | 655 | 3.00E-193 |
| FimC | 1482 | HMPREF9536_03309 | EFJ86409 | gram-negative pili assembly chaperone domain protein | Escherichia coli MS 84-1 | 655 | 3.00E-193 |
| FimC | 1483 | AE25_04218 | KDG93220 | chaperone fimC | Escherichia coli UCI 66 | 655 | 3.00E-193 |
| FimC | 1484 | JD73_14440 | KFD77449 | molecular chaperone FimC | Escherichia coli | 655 | 3.10E-193 |
| FimC | 1485 | HMPREF9535_02848 | EFK73242 | gram-negative pili assembly chaperone domain protein | Escherichia coli MS 78-1 | 654.9 | 3.20E-193 |
| FimC | 1486 | PCN061_4501 | AKM37932 | chaperone, periplasmic | Escherichia coli PCN061 | 654.9 | 3.20E-193 |
| FimC | 1487 | ECP02994387_4952 | ENC09264 | chaperone protein fimC | Escherichia coli P0299438.7 | 654.9 | 3.30E-193 |
| FimC | 1488 | G796_04496 | EQR88649 | chaperone fimC | Escherichia coli HVH 138 (4-6066704) | 654.8 | 3.40E-193 |
| FimC | 1489 | ECSTECH18_5455 | EGX01120 | chaperone protein fimC | Escherichia coli STEC_H.1.8 | 654.8 | 3.50E-193 |
| FimC | 1490 | ECDEC8C_0022 | EHW26529 | gram-negative pili assembly chaperone, N-terminal domain protein | Escherichia coli DEC8C | 654.8 | 3.50E-193 |
| FimC | 1491 | ECDEC9B_5157 | EHW33742 | gram-negative pili assembly chaperone, N-terminal domain protein | Escherichia coli DEC9B | 654.7 | 3.70E-193 |
| FimC | 1492 | ABE89_08825 | KML98772 | molecular chaperone FimC | Escherichia coli (GCA_001039155) | 654.7 | 3.70E-193 |
| FimC | 1493 | ECDEC9D_5247 | EHW47662 | gram-negative pili assembly chaperone, N-terminal domain protein | Escherichia coli DEC9D | 654.7 | 3.70E-193 |
| FimC | 1494 | ACU58_24695 | KPO16425 | molecular chaperone FimC | Escherichia coli (GCA_001309535) | 654.6 | 4.20E-193 |
| FimC | 1495 | AC81_4831 | EZJ65088 | chaperone protein fimC | Escherichia coli 1-176-05_S4_C1 | 654.5 | 4.30E-193 |
| FimC | 1496 | AD09_4736 | KDA87317 | chaperone protein fimC | Escherichia coli 1-176-05_S4_C2 | 654.5 | 4.30E-193 |
| FimC | 1497 | AD08_4712 | EYD94275 | chaperone protein fimC | Escherichia coli 1-110-08_S4_C2 | 654.5 | 4.30E-193 |
| FimC | 1498 | AC05_4619 | KDT58463 | chaperone protein fimC | Escherichia coli 3-267-03_S3_C1 | 654.5 | 4.30E-193 |
| FimC | 1499 | AD38_4818 | EZJ15089 | chaperone protein fimC | Escherichia coli 1-176-05_S4_C3 | 654.5 | 4.30E-193 |
| FimC | 1500 | BX29_09740 | EZE68853 | molecular chaperone FimC | Escherichia coli O45:H2 str. 2009C-4780 | 654.5 | 4.40E-193 |
| FimC | 1501 | ECP030481615_4789 | ENF42044 | chaperone protein fimC | Escherichia coli P0304816.15 | 654.2 | 5.60E-193 |
| FimC | 1502 | ECP030481612_4834 | ENF26946 | chaperone protein fimC | Escherichia coli P0304816.12 | 654.2 | 5.60E-193 |
| FimC | 1503 | ECP03048163_4905 | ENH27334 | chaperone protein fimC | Escherichia coli P0304816.3 | 654.2 | 5.60E-193 |
| FimC | 1504 | ECP030481610_4879 | ENF19848 | chaperone protein fimC | Escherichia coli P0304816.10 | 654.2 | 5.60E-193 |
| FimC | 1505 | ECP03048164_4861 | ENH27469 | chaperone protein fimC | Escherichia coli P0304816.4 | 654.2 | 5.60E-193 |
| FimC | 1506 | WIC_04867 | ELI18353 | fimbrial chaperone FimC | Escherichia coli KTE112 | 654 | 6.10E-193 |
| FimC | 1507 | A13W_03652 | ELC92564 | chaperone fimC | Escherichia coli KTE193 | 654 | 6.10E-193 |
| FimC | 1508 | BU69_15085 | EYB44912 | molecular chaperone FimC | Escherichia coli str. UCD_JA65_pb | 654 | 6.20E-193 |
| FimC | 1509 | ERCG_03411 | EGB31601 | gram-negative pili assembly chaperone domain-containing protein | Escherichia coli E1520 | 653.9 | 6.40E-193 |
| FimC | 1510 | BY98_14585 | EZD38443 | molecular chaperone FimC | Escherichia coli O111:NM str. K6728 | 653.8 | 7.10E-193 |
| FimC | 1511 | BX18_22290 | EZE38044 | molecular chaperone FimC | Escherichia coli O111:NM str. 2009C-4006 | 653.8 | 7.10E-193 |
| FimC | 1512 | BZ04_15315 | EZD66924 | molecular chaperone FimC | Escherichia coli O111:NM str. K6908 | 653.8 | 7.10E-193 |
| FimC | 1513 | ECO9574_00320 | EIL27251 | chaperone FimC, periplasmic | Escherichia coli O111:H8 str. CVM9574 | 653.8 | 7.10E-193 |
| FimC | 1514 | BW73_21770 | KDV16236 | molecular chaperone FimC | Escherichia coli O111:NM str. 01-3076 | 653.8 | 7.10E-193 |
| FimC | 1515 | BZ03_14775 | EZD59562 | molecular chaperone FimC | Escherichia coli O111:NM str. K6904 | 653.8 | 7.10E-193 |
| FimC | 1516 | BZ02_23735 | EZD49904 | molecular chaperone FimC | Escherichia coli O111:NM str. K6898 | 653.8 | 7.10E-193 |
| FimC | 1517 | BY99_00405 | EZD44726 | molecular chaperone FimC | Escherichia coli O111:NM str. K6890 | 653.8 | 7.10E-193 |
| FimC | 1518 | BZ01_24560 | EZD48102 | molecular chaperone FimC | Escherichia coli O111:NM str. K6897 | 653.8 | 7.10E-193 |
| FimC | 1519 | EC40522_5696 | EIH80139 | gram-negative pili assembly chaperone, N-terminal domain protein | Escherichia coli 4.0522 | 653.8 | 7.10E-193 |
| FimC | 1520 | BX73_05335 | EYY74089 | molecular chaperone FimC | Escherichia coli O111:NM str. 2010C-4735 | 653.8 | 7.10E-193 |
| FimC | 1521 | Q458_26330 | ETD60980 | molecular chaperone FimC | Escherichia coli ATCC BAA-2209 | 653.8 | 7.10E-193 |
| FimC | 1522 | BZ05_12460 | EZD70138 | molecular chaperone FimC | Escherichia coli O111:NM str. K6915 | 653.8 | 7.10E-193 |
| FimC | 1523 | BX77_23265 | EYY68576 | molecular chaperone FimC | Escherichia coli O111:NM str. 2010C-4818 | 653.8 | 7.10E-193 |
| FimC | 1524 | BY46_04540 | EZA87042 | molecular chaperone FimC | Escherichia coli O111:H8 str. F6627 | 653.8 | 7.10E-193 |
| FimC | 1525 | BX03_00010 | EYW94421 | molecular chaperone FimC | Escherichia coli O111:NM str. 08-4487 | 653.8 | 7.10E-193 |
| FimC | 1526 | BX20_15830 | EZE49754 | molecular chaperone FimC | Escherichia coli O111:NM str. 2009C-4052 | 653.8 | 7.10E-193 |
| FimC | 1527 | BX37_12040 | EZQ27083 | molecular chaperone FimC | Escherichia coli O111:H8 str. 2009EL-2169 | 653.8 | 7.10E-193 |
| FimC | 1528 | BX99_22925 | EZQ39569 | molecular chaperone FimC | Escherichia coli O111:H8 str. 2011C-3453 | 653.8 | 7.10E-193 |
| FimC | 1529 | BX58_19460 | EYU91586 | molecular chaperone FimC | Escherichia coli O111:NM str. 2010C-3977 | 653.8 | 7.10E-193 |
| FimC | 1530 | BX60_18890 | EYU74150 | molecular chaperone FimC | Escherichia coli O111:NM str. 2010C-4221 | 653.8 | 7.10E-193 |
| FimC | 1531 | BX74_01840 | EYY78256 | molecular chaperone FimC | Escherichia coli O111:NM str. 2010C-4746 | 653.8 | 7.10E-193 |
| FimC | 1532 | BX39_17125 | EZQ24494 | molecular chaperone FimC | Escherichia coli O111:NM str. 2010C-3053 | 653.8 | 7.10E-193 |
| FimC | 1533 | BX71_12225 | EYY94443 | molecular chaperone FimC | Escherichia coli O111:NM str. 2010C-4715 | 653.8 | 7.10E-193 |
| FimC | 1534 | WQ79_27195 | KLH56231 | molecular chaperone FimC | Escherichia coli (GCA_001012275) | 653.8 | 7.10E-193 |
| FimC | 1535 | ECO9602_05490 | EJE67481 | chaperone FimC, periplasmic | Escherichia coli O111:H8 str. CVM9602 | 653.8 | 7.10E-193 |
| FimC | 1536 | ECO9570_13003 | EIL26777 | chaperone FimC, periplasmic | Escherichia coli O111:H8 str. CVM9570 | 653.8 | 7.10E-193 |
| FimC | 1537 | BW82_15200 | EYZ89916 | molecular chaperone FimC | Escherichia coli O111:NM str. 04-3211 | 653.8 | 7.10E-193 |
| FimC | 1538 | BW80_07855 | EZA05263 | molecular chaperone FimC | Escherichia coli O111:NM str. 03-3484 | 653.8 | 7.10E-193 |
| FimC | 1539 | EC253486_0092 | EGW78210 | chaperone protein fimC | Escherichia coli 2534-86 | 653.8 | 7.10E-193 |
| FimC | 1540 | BX70_24795 | EYZ01492 | molecular chaperone FimC | Escherichia coli O111:NM str. 2010C-4622 | 653.8 | 7.10E-193 |
| FimC | 1541 | BZ00_10415 | EZD45062 | molecular chaperone FimC | Escherichia coli O111:NM str. K6895 | 653.8 | 7.10E-193 |
| FimC | 1542 | BX97_26185 | EYY04908 | molecular chaperone FimC | Escherichia coli O111:NM str. 2011C-3362 | 653.8 | 7.10E-193 |
| FimC | 1543 | CFSAN001632_05177 | EKU01688 | chaperone FimC, periplasmic | Escherichia coli O111:H8 str. CFSAN001632 | 653.8 | 7.10E-193 |
| FimC | 1544 | BY96_07045 | EZD34005 | molecular chaperone FimC | Escherichia coli O111:NM str. K6722 | 653.8 | 7.10E-193 |
| FimC | 1545 | BY07_20015 | EYX72195 | molecular chaperone FimC | Escherichia coli O111:NM str. 2011C-3679 | 653.8 | 7.10E-193 |
| FimC | 1546 | BY03_08480 | EYX95761 | molecular chaperone FimC | Escherichia coli O111:NM str. 2011C-3573 | 653.8 | 7.10E-193 |
| FimC | 1547 | BY97_00405 | EZD28883 | molecular chaperone FimC | Escherichia coli O111:NM str. K6723 | 653.8 | 7.10E-193 |
| FimC | 1548 | BX21_18990 | EZQ43149 | molecular chaperone FimC | Escherichia coli O111:H8 str. 2009C-4126 | 653.8 | 7.10E-193 |
| FimC | 1549 | BX76_08450 | EYY68969 | molecular chaperone FimC | Escherichia coli O111:NM str. 2010C-4799 | 653.8 | 7.10E-193 |
| FimC | 1550 | GR03_05740 | KFH86531 | molecular chaperone FimC | Escherichia coli str. CS02 | 653.7 | 7.70E-193 |
| FimC | 1551 | G832_04604 | ESP27349 | chaperone fimC | Escherichia coli HVH 178 (4-3189163) | 653.5 | 8.80E-193 |
| FimC | 1552 | LJ08_2851 | KGI48511 | chaperone FimC | Escherichia coli str. VKPM_B-10182 | 653.3 | 1.00E-192 |
| FimC | 1553 | A8V37_22545 | ANM85126 | molecular chaperone FimC | Escherichia coli | 653.2 | 1.10E-192 |
| FimC | 1554 | SU67_18575 | KIO39379 | molecular chaperone FimC | Escherichia coli O139:H28 str. E24377A (GCA_000832005) | 653.2 | 1.10E-192 |
| FimC | 1555 | BN16_03801 | CCK49696 | periplasmic chaperone, required for type 1 fimbriae | Escherichia coli chi7122 | 653.2 | 1.10E-192 |
| FimC | 1556 | APECO78_02720 | AGC85283 | chaperone FimC, periplasmic | Escherichia coli APEC O78 | 653.2 | 1.10E-192 |
| FimC | 1557 | HMPREF9348_02796 | EFO57969 | gram-negative pili assembly chaperone domain protein | Escherichia coli MS 145-7 | 653.2 | 1.10E-192 |
| FimC | 1558 | AD00_4499 | KDY14310 | chaperone protein fimC | Escherichia coli 2-316-03_S4_C2 | 653.2 | 1.10E-192 |
| FimC | 1559 | AB20_4316 | KDT80113 | chaperone protein fimC | Escherichia coli 3-475-03_S1_C1 | 652.9 | 1.30E-192 |
| FimC | 1560 | UH31_18760 | KIZ61413 | molecular chaperone FimC | Escherichia coli (GCA_000935475) | 652.9 | 1.40E-192 |
| FimC | 1561 | ABE81_24295 | AKI69311 | molecular chaperone FimC | Shigella boydii serotype 4 (strain Sb227) | 652.9 | 1.40E-192 |
| FimC | 1562 | SFCCH060_5011 | EIQ02286 | chaperone protein fimC | Shigella flexneri CCH060 | 652.9 | 1.40E-192 |
| FimC | 1563 | BHQ38_15210 | OEG48006 | molecular chaperone FimC | Shigella sp. FC2710 | 652.9 | 1.40E-192 |
| FimC | 1564 | SGF_01647 | EFW60912 | chaperone FimC | Shigella flexneri CDC 796-83 | 652.9 | 1.40E-192 |
| FimC | 1565 | SBO_4366 | ABB68782 | periplasmic chaperone | Shigella boydii Sb227 | 652.9 | 1.40E-192 |
| FimC | 1566 | L403_04593 | ESM26281 | chaperone fimC | Escherichia coli BWH 32 | 652.3 | 2.00E-192 |
| FimC | 1567 | AB45_3860 | KDZ79911 | chaperone protein fimC | Escherichia coli 3-105-05_S1_C2 | 652.3 | 2.10E-192 |
| FimC | 1568 | SF2A_22820 | AKK56603 | molecular chaperone FimC | Shigella flexneri G1663 | 651.4 | 3.80E-192 |
| FimC | 1569 | ECDEC5C_5262 | EHV31843 | gram-negative pili assembly chaperone, N-terminal domain protein | Escherichia coli DEC5C | 651.1 | 4.90E-192 |
| FimC | 1570 | ECO55CA74_24650 | AEZ43462 | chaperone protein FimC | Escherichia coli O55:H7 str. RM12579 | 651.1 | 4.90E-192 |
| FimC | 1571 | GS40_22105 | KFV24396 | molecular chaperone FimC | Escherichia coli (GCA_000749545) | 650.8 | 6.00E-192 |
| FimC | 1572 | BC97_0207365 | KFF39519 | molecular chaperone FimC | Escherichia coli str. E455L | 650.3 | 8.00E-192 |
| FimC | 1573 | A1YU_04046 | ELF07185 | chaperone fimC | Escherichia coli KTE142 | 650.2 | 8.80E-192 |
| FimC | 1574 | AUS26_13510 | KXL61320 | molecular chaperone FimC | Escherichia coli | 650.1 | 9.80E-192 |
| FimC | 1575 | AC15_4768 | KDW27001 | chaperone protein fimC | Escherichia coli 2-156-04_S3_C2 | 649.3 | 1.70E-191 |
| FimC | 1576 | C4893_41780 | EMR92141 | chaperone, periplasmic | Escherichia coli ONT:H33 str. C48/93 | 649.3 | 1.70E-191 |
| FimC | 1577 | AC067_06235 | KOA35879 | molecular chaperone FimC | Escherichia coli | 646.6 | 1.10E-190 |
| FimC | 1578 | ERJG_03165 | EGB60880 | gram-negative pili assembly chaperone domain-containing protein | Escherichia coli M863 | 646.6 | 1.10E-190 |
| FimC | 1579 | EC12741_5429 | EIG79748 | gram-negative pili assembly chaperone, N-terminal domain protein | Escherichia coli 1.2741 | 646.6 | 1.10E-190 |
| FimC | 1580 | G893_00522 | EQV94160 | chaperone fimC | Escherichia coli KOEGE 71 (186a) | 643.8 | 7.90E-190 |
| FimC | 1581 | WC5_01840 | EOU45985 | fimbrial chaperone FimC | Escherichia sp. KTE114 | 642 | 2.80E-189 |
| FimC | 1582 | WES_00527 | EOU82575 | fimbrial chaperone FimC | Escherichia sp. KTE31 | 640.6 | 7.10E-189 |
| FimC | 1583 | WEW_02708 | EOQ54954 | fimbrial chaperone FimC | Escherichia coli KTE33 | 640.6 | 7.10E-189 |
| FimC | 1584 | A1SC_03644 | EOV45335 | fimbrial chaperone FimC | Escherichia sp. KTE52 | 632.9 | 1.50E-186 |
| FimC | 1585 | A1WG_02408 | EOV91509 | fimbrial chaperone FimC | Escherichia sp. KTE96 | 632.9 | 1.50E-186 |
| FimC | 1586 | A31E_04157 | EOW56828 | fimbrial chaperone FimC | Escherichia sp. KTE159 | 632.9 | 1.50E-186 |
| FimC | 1587 | WCO_03933 | ELC14607 | chaperone fimC | Escherichia sp. KTE11 | 632.9 | 1.50E-186 |
| FimC | 1588 | AC41_4723 | KDA82248 | chaperone protein fimC | Escherichia coli 2-011-08_S3_C3 | 623.1 | 1.50E-183 |
| FimC | 1589 | K427_27055 | EWY51436 | molecular chaperone FimC | Escherichia coli MP1 | 611.1 | 6.60E-180 |
| FimC | 1590 | ECP030526013_4710 | ENF84119 | chaperone protein fimC | Escherichia coli P0305260.13 | 609.5 | 2.10E-179 |
| FimC | 1591 | CDL42375 | CDL42375 | chaperone FimC | Escherichia coli ISC41 | 608.6 | 3.70E-179 |
| FimC | 1592 | ECDEC6C_5137 | EHV52517 | chaperone protein fimC | Escherichia coli DEC6C | 608.6 | 3.70E-179 |
| FimC | 1593 | EAE6F_4201 | BAT46349 | chaperone, periplasmic | Escherichia albertii | 606.9 | 1.20E-178 |
| FimC | 1594 | ERS085411_00306 | CTU56173 | type I fimbrial chaperone | Escherichia coli | 606.9 | 1.20E-178 |
| FimC | 1595 | EAKF1_ch1537c | AHE59426 | chaperone FimC | Escherichia albertii KF1 | 606.9 | 1.20E-178 |
| FimC | 1596 | ECDEC13E_4949 | EHX67621 | gram-negative pili assembly chaperone, N-terminal domain protein | Escherichia coli DEC13E | 606.8 | 1.30E-178 |
| FimC | 1597 | ECP03052604_4697 | ENF98082 | chaperone protein fimC | Escherichia coli P0305260.4 | 606.8 | 1.30E-178 |
| FimC | 1598 | SFJ1713_5007 | EGM58765 | gram-negative pili assembly chaperone, N-terminal domain protein | Shigella flexneri SFJ17B | 606.5 | 1.70E-178 |
| FimC | 1599 | EC2749250_4761 | EMW64840 | chaperone protein fimC | Escherichia coli 2749250 | 606 | 2.40E-178 |
| FimC | 1600 | CDL30124 | CDL30124 | chaperone FimC | Escherichia coli ISC7 | 605.8 | 2.70E-178 |
| FimC | 1601 | ECP02994831_0100 | END74259 | chaperone protein fimC | Escherichia coli P0299483.1 | 605.8 | 2.70E-178 |
| FimC | 1602 | ECFRIK1996_5650 | EIN15637 | periplasmic chaperone required for type 1 fimbriae | Escherichia coli FRIK1996 | 604.8 | 5.20E-178 |
| FimC | 1603 | EC990713_0032 | EKW96277 | chaperone protein fimC | Escherichia coli 99.0713 | 604.8 | 5.20E-178 |
| FimC | 1604 | CDCO157_4960 | AFJ32008 | FimC | Escherichia coli Xuzhou21 | 604.8 | 5.20E-178 |
| FimC | 1605 | SB444474_5017 | EIQ29294 | chaperone protein fimC | Shigella boydii 4444-74 | 604.1 | 8.80E-178 |
| FimC | 1606 | AC82_5107 | EZJ56015 | chaperone protein fimC | Escherichia coli 1-182-04_S4_C1 | 602.4 | 2.90E-177 |
| FimC | 1607 | AB73_4981 | KDZ27089 | chaperone protein fimC | Escherichia coli 3-020-07_S1_C3 | 600 | 1.50E-176 |
| FimC | 1608 | ECSTEC7V_5029 | EGE61943 | chaperone protein fimC | Escherichia coli STEC_7v | 598.7 | 3.70E-176 |
| FimC | 1609 | A159_04285 | EOV19077 | fimbrial chaperone FimC | Escherichia coli KTE199 | 597.6 | 8.10E-176 |
| FimC | 1610 | ECP_4651 | ABG72587 | chaperone protein FimC precursor | Escherichia coli 536 | 597.6 | 8.10E-176 |
| FimC | 1611 | A199_00096 | ELD92819 | chaperone fimC | Escherichia coli KTE237 | 597.6 | 8.10E-176 |
| FimC | 1612 | A197_04842 | ELD76886 | chaperone fimC | Escherichia coli KTE236 | 597.6 | 8.10E-176 |
| FimC | 1613 | BX95_19100 | EZG86389 | molecular chaperone FimC | Escherichia coli O26:H11 str. 2011C-3270 | 595.1 | 4.60E-175 |
| FimC | 1614 | BY06_21870 | EZH08786 | molecular chaperone FimC | Escherichia coli O26:H11 str. 2011C-3655 | 595.1 | 4.60E-175 |
| FimC | 1615 | BX28_02915 | EZH22407 | molecular chaperone FimC | Escherichia coli O26:H11 str. 2009C-4760 | 595.1 | 4.60E-175 |
| FimC | 1616 | BW86_17885 | EZG48602 | molecular chaperone FimC | Escherichia coli O26:H11 str. 06-3464 | 595.1 | 4.60E-175 |
| FimC | 1617 | BX78_10735 | EZG64779 | molecular chaperone FimC | Escherichia coli O26:H11 str. 2010C-4819 | 595.1 | 4.60E-175 |
| FimC | 1618 | BX88_12130 | EZG83658 | molecular chaperone FimC | Escherichia coli O26:H11 str. 2010EL-1699 | 595.1 | 4.60E-175 |
| FimC | 1619 | BX57_18745 | EZH53158 | molecular chaperone FimC | Escherichia coli O26:H11 str. 2010C-3902 | 595.1 | 4.60E-175 |
| FimC | 1620 | BX85_15690 | EZG75451 | molecular chaperone FimC | Escherichia coli O26:H11 str. 2010C-5028 | 595.1 | 4.60E-175 |
| FimC | 1621 | BW81_21055 | EZG49253 | molecular chaperone FimC | Escherichia coli O26:H11 str. 03-3500 | 595.1 | 4.60E-175 |
| FimC | 1622 | HMPREF9551_00615 | EFI90321 | gram-negative pili assembly chaperone domain protein | Escherichia coli MS 196-1 | 595.1 | 4.60E-175 |
| FimC | 1623 | BX64_14805 | EZG59610 | molecular chaperone FimC | Escherichia coli O26:H11 str. 2010C-4430 | 595.1 | 4.60E-175 |
| FimC | 1624 | BX98_04770 | EZG90714 | molecular chaperone FimC | Escherichia coli O26:H11 str. 2011C-3387 | 595.1 | 4.60E-175 |
| FimC | 1625 | BX61_17480 | EZH56963 | molecular chaperone FimC | Escherichia coli O26:H11 str. 2010C-4244 | 595.1 | 4.60E-175 |
| FimC | 1626 | BX17_14830 | EZH25746 | molecular chaperone FimC | Escherichia coli O26:H11 str. 2009C-3996 | 595.1 | 4.60E-175 |
| FimC | 1627 | BX80_26550 | EZG69490 | molecular chaperone FimC | Escherichia coli O26:H11 str. 2010C-4834 | 595.1 | 4.60E-175 |
| FimC | 1628 | AC789_1c47720 | AJE58921 | periplasmic chaperone protein FimC precursor | Escherichia coli | 595.1 | 4.60E-175 |
| FimC | 1629 | BX96_24040 | EZG95721 | molecular chaperone FimC | Escherichia coli O26:H11 str. 2011C-3282 | 595.1 | 4.60E-175 |
| FimC | 1630 | BX30_16785 | EZH30898 | molecular chaperone FimC | Escherichia coli O26:H11 str. 2009C-4826 | 595.1 | 4.60E-175 |
| FimC | 1631 | BX38_24815 | EZH36773 | molecular chaperone FimC | Escherichia coli O26:H11 str. 2010C-3051 | 595.1 | 4.60E-175 |
| FimC | 1632 | BX55_20190 | EZH45598 | molecular chaperone FimC | Escherichia coli O26:H11 str. 2010C-3871 | 595.1 | 4.60E-175 |
| FimC | 1633 | BY01_21055 | EZG97587 | molecular chaperone FimC | Escherichia coli O26:H11 str. 2011C-3506 | 595.1 | 4.60E-175 |
| FimC | 1634 | BX41_24875 | EZH47253 | molecular chaperone FimC | Escherichia coli O26:H11 str. 2010C-3472 | 595.1 | 4.60E-175 |
| FimC | 1635 | EL76_4116 | KGM58313 | Chaperone protein FimC | Escherichia coli G3/10 | 593.3 | 1.60E-174 |
| FimC | 1636 | AC79_4758 | KEJ05052 | chaperone protein fimC | Escherichia coli 8-415-05_S4_C1 | 593.1 | 1.90E-174 |
| FimC | 1637 | L282_2738 | AJB37700 | type I fimbrial chaperone | Escherichia coli APEC IMT5155 | 593.1 | 1.90E-174 |
| FimC | 1638 | AB96_0004 | KEN45780 | chaperone protein fimC | Escherichia coli 8-415-05_S3_C1 | 593.1 | 1.90E-174 |
| FimC | 1639 | ECDEC8A_5256 | EHW03145 | chaperone protein fimC | Escherichia coli DEC8A | 592.3 | 3.40E-174 |
| FimC | 1640 | AB51_4795 | KEM08011 | chaperone protein fimC | Escherichia coli 6-319-05_S1_C2 | 592 | 4.20E-174 |
| FimC | 1641 | BX15_07580 | EZH15605 | molecular chaperone FimC | Escherichia coli O26:H11 str. 2009C-3689 | 591.6 | 5.40E-174 |
| FimC | 1642 | BX13_23220 | EZH10145 | molecular chaperone FimC | Escherichia coli O26:H11 str. 2009C-3612 | 591.6 | 5.40E-174 |
| FimC | 1643 | EL80_3904 | KGM77368 | Chaperone protein FimC | Escherichia coli | 591.5 | 5.80E-174 |
| FimC | 1644 | ECPA3_5721 | EIN49130 | periplasmic chaperone required for type 1 fimbriae | Escherichia coli PA3 | 591.4 | 6.40E-174 |
| FimC | 1645 | ECTW09098_5716 | EIO65763 | periplasmic chaperone required for type 1 fimbriae | Escherichia coli TW09098 | 591.4 | 6.40E-174 |
| FimC | 1646 | ECEC4402_5671 | EIP27255 | periplasmic chaperone required for type 1 fimbriae | Escherichia coli EC4402 | 591.4 | 6.40E-174 |
| FimC | 1647 | ECEC4421_5510 | EIP10326 | periplasmic chaperone required for type 1 fimbriae | Escherichia coli EC4421 | 591.4 | 6.40E-174 |
| FimC | 1648 | ECEC4436_5591 | EIP40238 | periplasmic chaperone required for type 1 fimbriae | Escherichia coli EC4436 | 591.4 | 6.40E-174 |
| FimC | 1649 | ECPA31_5533 | EIO07002 | periplasmic chaperone required for type 1 fimbriae | Escherichia coli PA31 | 591.4 | 6.40E-174 |
| FimC | 1650 | ECFDA517_5913 | EIN16650 | periplasmic chaperone required for type 1 fimbriae | Escherichia coli FDA517 | 591.4 | 6.40E-174 |
| FimC | 1651 | ECMA6_0147 | EKI01282 | periplasmic chaperone required for type 1 fimbriae | Escherichia coli MA6 | 591.4 | 6.40E-174 |
| FimC | 1652 | ECEC1869_0033 | EKJ47681 | periplasmic chaperone required for type 1 fimbriae | Escherichia coli EC1869 | 591.4 | 6.40E-174 |
| FimC | 1653 | ECSTECB2F1_4715 | EGW64662 | chaperone protein fimC | Escherichia coli STEC_B2F1 | 591.3 | 6.50E-174 |
| FimC | 1654 | EcSMS35_4843 | ACB19299 | chaperone protein FimC | Escherichia coli SMS-3-5 | 590.6 | 1.10E-173 |
| FimC | 1655 | AD23_4868 | EZJ33442 | chaperone protein fimC | Escherichia coli 2-005-03_S4_C3 | 590.5 | 1.10E-173 |
| FimC | 1656 | AB50_4958 | KEJ04517 | chaperone protein fimC | Escherichia coli 6-175-07_S1_C2 | 590.5 | 1.10E-173 |
| FimC | 1657 | AC80_5199 | EYD96376 | chaperone protein fimC | Escherichia coli 1-110-08_S4_C1 | 590.5 | 1.10E-173 |
| FimC | 1658 | EC91649_4798 | KKA62077 | gram-negative pili assembly chaperone, N-terminal domain protein | Escherichia coli 9.1649 | 590.5 | 1.10E-173 |
| FimC | 1659 | AC67_4943 | KDT24189 | chaperone protein fimC | Escherichia coli 2-052-05_S4_C1 | 590.5 | 1.10E-173 |
| FimC | 1660 | AB60_4822 | KDV96359 | chaperone protein fimC | Escherichia coli 2-156-04_S1_C3 | 590.5 | 1.10E-173 |
| FimC | 1661 | AB14_4664 | KDW70400 | chaperone protein fimC | Escherichia coli 1-392-07_S1_C1 | 590.5 | 1.10E-173 |
| FimC | 1662 | AD15_4357 | KDT42019 | chaperone protein fimC | Escherichia coli 3-105-05_S4_C2 | 590.5 | 1.10E-173 |
| FimC | 1663 | L913_3475 | ESA25198 | chaperone FimC | Escherichia coli SCD2 | 590.5 | 1.10E-173 |
| FimC | 1664 | AB79_5084 | KEM48573 | chaperone protein fimC | Escherichia coli 6-175-07_S1_C3 | 590.5 | 1.10E-173 |
| FimC | 1665 | SF5M90T_4118 | EID64421 | periplasmic chaperone | Shigella flexneri 5a str. M90T | 589.3 | 2.70E-173 |
| FimC | 1666 | SFV_4210 | ABF06194 | Chaperone protein fimC precursor | Shigella flexneri 5 str. 8401 | 589.3 | 2.70E-173 |
| FimC | 1667 | AB09_4603 | KEN18966 | chaperone protein fimC | Escherichia coli 8-415-05_S1_C1 | 588.8 | 3.80E-173 |
| FimC | 1668 | AB37_4679 | KEO04666 | chaperone protein fimC | Escherichia coli 8-415-05_S1_C2 | 588.8 | 3.80E-173 |
| FimC | 1669 | AC06_4050 | KDT63322 | chaperone protein fimC | Escherichia coli 3-373-03_S3_C1 | 588.8 | 3.80E-173 |
| FimC | 1670 | AB95_4994 | KEM67835 | chaperone protein fimC | Escherichia coli 7-233-03_S3_C1 | 588.1 | 6.40E-173 |
| FimC | 1671 | ECTX1999_5178 | EGX18418 | chaperone protein fimC | Escherichia coli TX1999 | 588.1 | 6.40E-173 |
| FimC | 1672 | AB81_4982 | KEN49308 | chaperone protein fimC | Escherichia coli 6-537-08_S1_C3 | 588.1 | 6.40E-173 |
| FimC | 1673 | ECOK1357_4712 | EFZ67443 | chaperone protein fimC | Escherichia coli OK1357 | 588.1 | 6.40E-173 |
| FimC | 1674 | AC18_4885 | KEN93629 | chaperone protein fimC | Escherichia coli 2-222-05_S3_C2 | 588.1 | 6.40E-173 |
| FimC | 1675 | AB01_5015 | KDW13408 | chaperone protein fimC | Escherichia coli 2-177-06_S1_C1 | 588.1 | 6.40E-173 |
| FimC | 1676 | AD30_5211 | KDY13732 | chaperone protein fimC | Escherichia coli 2-316-03_S4_C3 | 588.1 | 6.40E-173 |
| FimC | 1677 | AB97_0055 | EYE30787 | chaperone protein fimC | Escherichia coli 1-110-08_S3_C1 | 588.1 | 6.40E-173 |
| FimC | 1678 | AC30_4907 | KEJ64136 | chaperone protein fimC | Escherichia coli 3-020-07_S3_C2 | 588.1 | 6.40E-173 |
| FimC | 1679 | AC13_4747 | KDA76991 | chaperone protein fimC | Escherichia coli 2-011-08_S3_C2 | 588.1 | 6.40E-173 |
| FimC | 1680 | AB52_4907 | KEN41176 | chaperone protein fimC | Escherichia coli 6-537-08_S1_C2 | 588.1 | 6.40E-173 |
| FimC | 1681 | AC85_5372 | KEJ53306 | chaperone protein fimC | Escherichia coli 3-020-07_S4_C1 | 588.1 | 6.40E-173 |
| FimC | 1682 | AC16_3145 | KDX44287 | chaperone protein fimC | Escherichia coli 2-177-06_S3_C2 | 588.1 | 6.40E-173 |
| FimC | 1683 | ECDEC14D_5079 | EHX84446 | gram-negative pili assembly chaperone, N-terminal domain protein | Escherichia coli DEC14D | 588.1 | 6.40E-173 |
| FimC | 1684 | i01_06270 | EHF98111 | type 1 fimbrial chaperone protein precursor | Escherichia coli cloneA_i1 | 588.1 | 6.40E-173 |
| FimC | 1685 | BU58_07435 | KDV67598 | molecular chaperone FimC | Escherichia coli O26:H11 str. 2011C-3274 | 588.1 | 6.40E-173 |
| FimC | 1686 | AD10_4915 | EZJ36457 | chaperone protein fimC | Escherichia coli 1-182-04_S4_C2 | 588.1 | 6.40E-173 |
| FimC | 1687 | ECDEC14B_5247 | EHX73034 | gram-negative pili assembly chaperone, N-terminal domain protein | Escherichia coli DEC14B | 588.1 | 6.40E-173 |
| FimC | 1688 | AB24_5052 | KEM36348 | chaperone protein fimC | Escherichia coli 6-537-08_S1_C1 | 588.1 | 6.40E-173 |
| FimC | 1689 | AC52_4858 | KEL70459 | chaperone protein fimC | Escherichia coli 5-366-08_S3_C3 | 588.1 | 6.40E-173 |
| FimC | 1690 | ECLT68_3968 | EFZ56975 | chaperone protein fimC | Escherichia coli LT-68 | 588.1 | 6.40E-173 |
| FimC | 1691 | AC25_5207 | EYE15529 | chaperone protein fimC | Escherichia coli 1-110-08_S3_C2 | 588.1 | 6.40E-173 |
| FimC | 1692 | ECDEC6B_5546 | EHV49595 | gram-negative pili assembly chaperone, N-terminal domain protein | Escherichia coli DEC6B | 588.1 | 6.40E-173 |
| FimC | 1693 | AC74_5118 | KEJ45468 | chaperone protein fimC | Escherichia coli 2-460-02_S4_C1 | 588.1 | 6.40E-173 |
| FimC | 1694 | AC87_4622 | KDT89890 | chaperone protein fimC | Escherichia coli 3-105-05_S4_C1 | 588.1 | 6.40E-173 |
| FimC | 1695 | AB65_5217 | KEJ36641 | chaperone protein fimC | Escherichia coli 2-460-02_S1_C3 | 588.1 | 6.40E-173 |
| FimC | 1696 | BU59_05345 | KDV32503 | molecular chaperone FimC | Escherichia coli O69:H11 str. 07-3763 | 588.1 | 6.40E-173 |
| FimC | 1697 | AC75_4756 | KEN81966 | chaperone protein fimC | Escherichia coli 2-474-04_S4_C1 | 588.1 | 6.40E-173 |
| FimC | 1698 | AB94_4543 | KEL89027 | chaperone protein fimC | Escherichia coli 5-366-08_S3_C1 | 588.1 | 6.40E-173 |
| FimC | 1699 | AC00_4899 | EZJ80848 | chaperone protein fimC | Escherichia coli 1-250-04_S3_C1 | 588.1 | 6.40E-173 |
| FimC | 1700 | AB10_4751 | EYE31739 | chaperone protein fimC | Escherichia coli 1-110-08_S1_C1 | 588.1 | 6.40E-173 |
| FimC | 1701 | L960_2485c | AIF62308 | type 1 fimbrial chaperone protein precursor | Escherichia coli B7A (GCA_000725265) | 588.1 | 6.40E-173 |
| FimC | 1702 | SB521682_5168 | EGI88519 | chaperone protein fimC | Shigella boydii 5216-82 | 588.1 | 6.40E-173 |
| FimC | 1703 | ECDEC13B_4648 | EHX55106 | gram-negative pili assembly chaperone, N-terminal domain protein | Escherichia coli DEC13B | 586.2 | 2.30E-172 |
| FimC | 1704 | EC30301_5110 | EGW77989 | chaperone protein fimC | Escherichia coli 3030-1 | 586.2 | 2.30E-172 |
| FimC | 1705 | EC12264_5384 | EIH21356 | gram-negative pili assembly chaperone, N-terminal domain protein | Escherichia coli 1.2264 | 586.2 | 2.30E-172 |
| FimC | 1706 | AC96_4806 | KDX22357 | chaperone protein fimC | Escherichia coli 2-156-04_S4_C2 | 586.2 | 2.30E-172 |
| FimC | 1707 | BU53_15865 | KDV46144 | molecular chaperone FimC | Escherichia coli O91:H21 str. 2009C-3740 | 586.2 | 2.30E-172 |
| FimC | 1708 | BU55_12170 | KDV39760 | molecular chaperone FimC | Escherichia coli O146:H21 str. 2010C-3325 | 586.2 | 2.30E-172 |
| FimC | 1709 | CF58_03240 | AHM42057 | molecular chaperone FimC | Escherichia coli str. ST540 (GCA_000599705) | 586.2 | 2.30E-172 |
| FimC | 1710 | BU64_06065 | KDV62133 | molecular chaperone FimC | Escherichia coli O128:H2 str. 2011C-3317 | 586.2 | 2.30E-172 |
| FimC | 1711 | AB46_4342 | KDU00162 | chaperone protein fimC | Escherichia coli 3-267-03_S1_C2 | 586.2 | 2.30E-172 |
| FimC | 1712 | EcF11_0637 | EDV68456 | chaperone protein FimC | Escherichia coli F11 | 586.1 | 2.60E-172 |
| FimC | 1713 | AB67_5014 | KEJ70457 | chaperone protein fimC | Escherichia coli 5-366-08_S1_C3 | 585.6 | 3.70E-172 |
| FimC | 1714 | AB08_0273 | KEL72194 | chaperone protein fimC | Escherichia coli 5-366-08_S1_C1 | 585.6 | 3.70E-172 |
| FimC | 1715 | BU56_08180 | KDV36604 | molecular chaperone FimC | Escherichia coli O145:H25 str. 07-3858 | 585.6 | 3.70E-172 |
| FimC | 1716 | EC970246_4015 | EIG92838 | gram-negative pili assembly chaperone, N-terminal domain protein | Escherichia coli 97.0246 | 585.6 | 3.70E-172 |
| FimC | 1717 | EC50959_5373 | EII10041 | gram-negative pili assembly chaperone, N-terminal domain protein | Escherichia coli 5.0959 | 585.3 | 4.40E-172 |
| FimC | 1718 | ECDEC9E_5626 | EHW49049 | gram-negative pili assembly chaperone, N-terminal domain protein | Escherichia coli DEC9E | 585.2 | 4.70E-172 |
| FimC | 1719 | ECRM13514_5560 | AHG12176 | chaperone FimC | Escherichia coli O145:H28 str. RM13514 | 585.2 | 4.70E-172 |
| FimC | 1720 | ECDEC8B_5522 | EHW02958 | gram-negative pili assembly chaperone, N-terminal domain protein | Escherichia coli DEC8B | 585.2 | 4.70E-172 |
| FimC | 1721 | ExPEC_4154 | KQL75869 | type 1 fimbrial chaperone protein precursor | Escherichia coli (GCA_001421045) | 585.2 | 4.70E-172 |
| FimC | 1722 | EC01288_4722 | EKJ54225 | type I fimbrial chaperone | Escherichia coli 0.1288 | 585.2 | 4.70E-172 |
| FimC | 1723 | ECRM12581_27325 | AHY73995 | chaperone FimC | Escherichia coli O145:H28 str. RM12581 | 585.2 | 4.70E-172 |
| FimC | 1724 | AB03_5251 | KEJ20371 | chaperone protein fimC | Escherichia coli 2-316-03_S1_C1 | 585.1 | 4.90E-172 |
| FimC | 1725 | BW72_33400 | KDV18469 | molecular chaperone FimC | Escherichia coli O78:H12 str. 00-3279 | 584.6 | 7.40E-172 |
| FimC | 1726 | ECPA14_5740 | EIN69776 | periplasmic chaperone required for type 1 fimbriae | Escherichia coli PA14 | 584.3 | 8.80E-172 |
| FimC | 1727 | ECFDA504_0033 | EKH36334 | periplasmic chaperone required for type 1 fimbriae | Escherichia coli FDA504 | 584.3 | 8.80E-172 |
| FimC | 1728 | ECEC1848_0097 | EKI91888 | periplasmic chaperone required for type 1 fimbriae | Escherichia coli EC1848 | 584.3 | 8.80E-172 |
| FimC | 1729 | EC93001_5705 | EIN33855 | periplasmic chaperone required for type 1 fimbriae | Escherichia coli 93-001 | 584.3 | 8.80E-172 |
| FimC | 1730 | ECTW09109_0099 | EIO79119 | periplasmic chaperone required for type 1 fimbriae | Escherichia coli TW09109 | 584.3 | 8.80E-172 |
| FimC | 1731 | EC08BKT77219_5184 | ERE00900 | chaperone protein fimC | Escherichia coli 08BKT77219 | 584.3 | 8.80E-172 |
| FimC | 1732 | ECBD561099_5215 | ERC62111 | chaperone protein fimC | Escherichia coli Bd5610_99 | 584.3 | 8.80E-172 |
| FimC | 1733 | ECEC1736_5567 | EKI57101 | periplasmic chaperone required for type 1 fimbriae | Escherichia coli EC1736 | 584.3 | 8.80E-172 |
| FimC | 1734 | ECFRIK1985_5879 | EIN33006 | periplasmic chaperone required for type 1 fimbriae | Escherichia coli FRIK1985 | 584.3 | 8.80E-172 |
| FimC | 1735 | ECEC1846_5613 | EKI64793 | periplasmic chaperone required for type 1 fimbriae | Escherichia coli EC1846 | 584.3 | 8.80E-172 |
| FimC | 1736 | ECTW14301_5465 | EIP05616 | periplasmic chaperone required for type 1 fimbriae | Escherichia coli TW14301 | 584.3 | 8.80E-172 |
| FimC | 1737 | ECEC1870_5607 | EKJ36517 | periplasmic chaperone required for type 1 fimbriae | Escherichia coli EC1870 | 584.3 | 8.80E-172 |
| FimC | 1738 | ECFDA505_5586 | EIN15793 | periplasmic chaperone required for type 1 fimbriae | Escherichia coli FDA505 | 584.3 | 8.80E-172 |
| FimC | 1739 | ECEC1845_5598 | EIP72270 | periplasmic chaperone required for type 1 fimbriae | Escherichia coli EC1845 | 584.3 | 8.80E-172 |
| FimC | 1740 | ECEC1866_5505 | EKJ20882 | periplasmic chaperone required for type 1 fimbriae | Escherichia coli EC1866 | 584.3 | 8.80E-172 |
| FimC | 1741 | EC08BKT55439_5136 | ERC53535 | chaperone protein fimC | Escherichia coli 08BKT055439 | 584.3 | 8.80E-172 |
| FimC | 1742 | ECDEC4F_5258 | EHV16596 | gram-negative pili assembly chaperone, N-terminal domain protein | Escherichia coli DEC4F | 584.3 | 8.80E-172 |
| FimC | 1743 | ECNE037_0035 | EKH61335 | periplasmic chaperone required for type 1 fimbriae | Escherichia coli NE037 | 584.3 | 8.80E-172 |
| FimC | 1744 | ECFRIK1999_0017 | EKH43261 | periplasmic chaperone required for type 1 fimbriae | Escherichia coli FRIK1999 | 584.3 | 8.80E-172 |
| FimC | 1745 | BN1008_721 | CEE04065 | chaperone protein fimC | Escherichia coli (GCA_000751315) | 584.3 | 9.00E-172 |
| FimC | 1746 | SF148580_4847 | EJZ61173 | gram-negative pili assembly chaperone, N-terminal domain protein | Shigella flexneri 1485-80 | 583.6 | 1.40E-171 |
| FimC | 1747 | AC35_2642 | KEK87149 | chaperone protein fimC | Escherichia coli 3-475-03_S3_C2 | 583.5 | 1.50E-171 |
| FimC | 1748 | J444_4741 | AKP87391 | type I fimbrial chaperone | Escherichia coli ACN001 | 583.4 | 1.70E-171 |
| FimC | 1749 | ECDEC9C_5169 | EHW39336 | gram-negative pili assembly chaperone, N-terminal domain protein | Escherichia coli DEC9C | 582.2 | 3.90E-171 |
| FimC | 1750 | AD37_4809 | EYD93629 | chaperone protein fimC | Escherichia coli 1-110-08_S4_C3 | 582.1 | 4.10E-171 |
| FimC | 1751 | ECOK1180_4897 | EFZ61798 | chaperone protein fimC | Escherichia coli OK1180 | 581.3 | 7.20E-171 |
| FimC | 1752 | AB12_4959 | EZK25496 | chaperone protein fimC | Escherichia coli 1-182-04_S1_C1 | 580.9 | 9.40E-171 |
| FimC | 1753 | AB71_0116 | EZK00370 | chaperone protein fimC | Escherichia coli 1-182-04_S1_C3 | 580.9 | 9.40E-171 |
| FimC | 1754 | EC07798_0005 | EKI46124 | type I fimbrial chaperone | Escherichia coli 07798 | 580.6 | 1.20E-170 |
| FimC | 1755 | ECPA10_5912 | EIN65432 | periplasmic chaperone required for type 1 fimbriae | Escherichia coli PA10 | 579.3 | 2.90E-170 |
| FimC | 1756 | ECFRIK1997_0035 | EKH48849 | periplasmic chaperone required for type 1 fimbriae | Escherichia coli FRIK1997 | 579.3 | 2.90E-170 |
| FimC | 1757 | ECPA41_5701 | EIO29680 | periplasmic chaperone required for type 1 fimbriae | Escherichia coli PA41 | 579.3 | 2.90E-170 |
| FimC | 1758 | AD12_0004 | EZJ31870 | chaperone protein fimC | Escherichia coli 1-392-07_S4_C2 | 579 | 3.60E-170 |
| FimC | 1759 | ECDEC5D_0191 | EHV46613 | gram-negative pili assembly chaperone, N-terminal domain protein | Escherichia coli DEC5D | 578.7 | 4.30E-170 |
| FimC | 1760 | AB73_4127 | KDZ30481 | chaperone protein fimC | Escherichia coli 3-020-07_S1_C3 | 578.3 | 5.80E-170 |
| FimC | 1761 | ECEC4448_5648 | EIP49946 | periplasmic chaperone required for type 1 fimbriae | Escherichia coli EC4448 | 577.1 | 1.40E-169 |
| FimC | 1762 | ECPA22_0097 | EIN94359 | periplasmic chaperone required for type 1 fimbriae | Escherichia coli PA22 | 577.1 | 1.40E-169 |
| FimC | 1763 | AC26_3401 | EYD82548 | chaperone protein fimC | Escherichia coli 1-176-05_S3_C2 | 575.8 | 3.40E-169 |
| FimC | 1764 | AB40_4849 | KDA65778 | chaperone protein fimC | 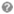  | Escherichia coli 1-182-04_S1_C2 | | --- | | 573.7 | 1.50E-168 |
| FimH | 1 | AB747_20765 | KNX99849 | fimbrial protein | Escherichia coli (strain UTI89 / UPEC) | 817.4 | 2.90E-242 |
| FimH | 2 | G997_04676 | ERA14692 | protein fimH | Escherichia coli UMEA 3834-1 | 817.4 | 2.90E-242 |
| FimH | 3 | UTI89_C5017 | ABE10420 | type 1 fimbiral adhesin FimH | Escherichia coli UTI89 | 817.4 | 2.90E-242 |
| FimH | 4 | A1SI_00422 | ELE16042 | protein fimH | Escherichia coli KTE55 | 817.4 | 2.90E-242 |
| FimH | 5 | SC80_07035 | KIE82465 | fimbrial protein | Escherichia coli RS218 | 817.4 | 2.90E-242 |
| FimH | 6 | ECOK1_4819 | ADE90235 | protein FimH | Escherichia coli IHE3034 | 817.4 | 2.90E-242 |
| FimH | 7 | UM146_22290 | ADN73782 | type 1 fimbiral adhesin FimH | Escherichia coli UM146 | 817.4 | 2.90E-242 |
| FimH | 8 | G961_04752 | ERB28786 | protein fimH | Escherichia coli UMEA 3298-1 | 817.4 | 2.90E-242 |
| FimH | 9 | V415_14885 | ETE21302 | fimbrial protein FimH | Escherichia coli LAU-EC10 | 815.5 | 1.10E-241 |
| FimH | 10 | A1Y9_04267 | EOW25277 | fimbrial protein FimH | Escherichia coli KTE121 | 815.5 | 1.10E-241 |
| FimH | 11 | WE5_03974 | EOU59202 | fimbrial protein FimH | Escherichia coli KTE19 | 815.5 | 1.10E-241 |
| FimH | 12 | G899_04637 | EQW12610 | protein fimH | Escherichia coli UMEA 3022-1 | 815.5 | 1.10E-241 |
| FimH | 13 | AB42_4429 | KDW79819 | protein fimH | Escherichia coli 1-392-07_S1_C2 | 815.5 | 1.10E-241 |
| FimH | 14 | A13C_03592 | ELH36559 | fimbrial protein FimH | Escherichia coli KTE183 | 815.5 | 1.10E-241 |
| FimH | 15 | AB50_4962 | KEJ04521 | protein fimH | Escherichia coli 6-175-07_S1_C2 | 815.5 | 1.10E-241 |
| FimH | 16 | A13M_00164 | ELC85873 | protein fimH | Escherichia coli KTE188 | 815.5 | 1.10E-241 |
| FimH | 17 | G786_04688 | EQR43921 | protein fimH | Escherichia coli HVH 126 (4-6034225) | 815.5 | 1.10E-241 |
| FimH | 18 | G688_04337 | EQN26614 | protein fimH | Escherichia coli HVH 9 (4-6942539) | 815.5 | 1.10E-241 |
| FimH | 19 | K427_27035 | EWY51432 | fimbrial protein | Escherichia coli MP1 | 815.5 | 1.10E-241 |
| FimH | 20 | T654_04034 | EYT05009 | protein fimH | Escherichia coli K02 | 815.5 | 1.10E-241 |
| FimH | 21 | G700_04360 | EQN79982 | protein fimH | Escherichia coli HVH 24 (4-5985145) | 815.5 | 1.10E-241 |
| FimH | 22 | A1WE_00064 | ELE94955 | protein fimH | Escherichia coli KTE93 | 815.5 | 1.10E-241 |
| FimH | 23 | A17Y_00162 | ELD65679 | protein fimH | Escherichia coli KTE230 | 815.5 | 1.10E-241 |
| FimH | 24 | A159_04281 | EOV19073 | fimbrial protein FimH | Escherichia coli KTE199 | 815.5 | 1.10E-241 |
| FimH | 25 | G909_04632 | EQW60044 | protein fimH | Escherichia coli UMEA 3113-1 | 815.5 | 1.10E-241 |
| FimH | 26 | A1S1_04331 | ELF92169 | fimbrial protein FimH | Escherichia coli KTE46 | 815.5 | 1.10E-241 |
| FimH | 27 | G845_04403 | EQT84578 | protein fimH | Escherichia coli HVH 193 (4-3331423) | 815.5 | 1.10E-241 |
| FimH | 28 | G946_04367 | EQY23637 | protein fimH | Escherichia coli UMEA 3217-1 | 815.5 | 1.10E-241 |
| FimH | 29 | G716_04674 | EQO53401 | protein fimH | Escherichia coli HVH 41 (4-2677849) | 815.5 | 1.10E-241 |
| FimH | 30 | G923_04704 | EQX13152 | protein fimH | Escherichia coli UMEA 3160-1 | 815.5 | 1.10E-241 |
| FimH | 31 | L282_2742 | AJB37704 | type 1 fimbiral adhesin FimH | Escherichia coli APEC IMT5155 | 815.5 | 1.10E-241 |
| FimH | 32 | G761_01038 | EQQ43253 | protein fimH | Escherichia coli HVH 100 (4-2850729) | 815.5 | 1.10E-241 |
| FimH | 33 | WI3_00007 | ELJ99868 | fimbrial protein FimH | Escherichia coli KTE99 | 815.5 | 1.10E-241 |
| FimH | 34 | A197_04838 | ELD76882 | protein fimH | Escherichia coli KTE236 | 815.5 | 1.10E-241 |
| FimH | 35 | G717_04828 | EQO56933 | protein fimH | Escherichia coli HVH 42 (4-2100061) | 815.5 | 1.10E-241 |
| FimH | 36 | WGS_04334 | ELJ64315 | fimbrial protein FimH | Escherichia coli KTE88 | 815.5 | 1.10E-241 |
| FimH | 37 | CDK45232 | CDK45232 | mannose-specific adhesin FimH | Escherichia coli IS1 | 815.5 | 1.10E-241 |
| FimH | 38 | H001_04570 | ERA28706 | protein fimH | Escherichia coli UMEA 3955-1 | 815.5 | 1.10E-241 |
| FimH | 39 | G683_04705 | EQN00207 | protein fimH | Escherichia coli HVH 3 (4-7276001) | 815.5 | 1.10E-241 |
| FimH | 40 | G922_04599 | EQX05415 | protein fimH | Escherichia coli UMEA 3159-1 | 815.5 | 1.10E-241 |
| FimH | 41 | ECKD1_07334 | EIL52294 | type 1 fimbiral adhesin FimH | Escherichia coli KD1 | 815.5 | 1.10E-241 |
| FimH | 42 | WIE_00175 | ELI30713 | fimbrial protein FimH | Escherichia coli KTE113 | 815.5 | 1.10E-241 |
| FimH | 43 | G682_04740 | EQM98472 | protein fimH | Escherichia coli HVH 2 (4-6943160) | 815.5 | 1.10E-241 |
| FimH | 44 | G850_04504 | EQU05506 | protein fimH | Escherichia coli HVH 198 (4-3206106) | 815.5 | 1.10E-241 |
| FimH | 45 | G728_04402 | EQP02773 | protein fimH | Escherichia coli HVH 56 (4-2153033) | 815.5 | 1.10E-241 |
| FimH | 46 | WCY_00465 | ELC32640 | protein fimH | Escherichia coli KTE16 | 815.5 | 1.10E-241 |
| FimH | 47 | G773_04508 | EQQ80597 | protein fimH | Escherichia coli HVH 112 (4-5987253) | 815.5 | 1.10E-241 |
| FimH | 48 | G977_04850 | EQZ23815 | protein fimH | Escherichia coli UMEA 3585-1 | 815.5 | 1.10E-241 |
| FimH | 49 | A13U_00404 | ELH13134 | fimbrial protein FimH | Escherichia coli KTE192 | 815.5 | 1.10E-241 |
| FimH | 50 | G898_04602 | EQW12217 | protein fimH | Escherichia coli UMEA 3014-1 | 815.5 | 1.10E-241 |
| FimH | 51 | G974_00413 | ESK21410 | protein fimH | Escherichia coli UMEA 3426-1 | 815.5 | 1.10E-241 |
| FimH | 52 | G950_04661 | EQY37719 | protein fimH | Escherichia coli UMEA 3230-1 | 815.5 | 1.10E-241 |
| FimH | 53 | WGK_00473 | ELF70112 | fimbrial protein FimH | Escherichia coli KTE45 | 815.5 | 1.10E-241 |
| FimH | 54 | ECOPMV1_04766 | CDH68047 | hypothetical protein | Escherichia coli PMV-1 | 815.5 | 1.10E-241 |
| FimH | 55 | G859_04715 | EQU51491 | protein fimH | Escherichia coli HVH 207 (4-3113221) | 815.5 | 1.10E-241 |
| FimH | 56 | G991_04532 | EQZ84869 | protein fimH | Escherichia coli UMEA 3703-1 | 815.5 | 1.10E-241 |
| FimH | 57 | A1UO_04718 | ELE54295 | protein fimH | Escherichia coli KTE76 | 815.5 | 1.10E-241 |
| FimH | 58 | A1W3_00482 | ELG38953 | fimbrial protein FimH | Escherichia coli KTE84 | 815.5 | 1.10E-241 |
| FimH | 59 | G900_03779 | EQW24836 | protein fimH | Escherichia coli UMEA 3033-1 | 815.5 | 1.10E-241 |
| FimH | 60 | A1U7_00769 | ELE44680 | protein fimH | Escherichia coli KTE67 | 815.5 | 1.10E-241 |
| FimH | 61 | A1SS_00319 | ELE36064 | protein fimH | Escherichia coli KTE60 | 815.5 | 1.10E-241 |
| FimH | 62 | CDK52696 | CDK52696 | mannose-specific adhesin FimH | Escherichia coli IS5 | 815.5 | 1.10E-241 |
| FimH | 63 | A13S_00404 | ELC93709 | protein fimH | Escherichia coli KTE191 | 815.5 | 1.10E-241 |
| FimH | 64 | G892_04597 | EQV75849 | protein fimH | Escherichia coli KOEGE 70 (185a) | 815.5 | 1.10E-241 |
| FimH | 65 | G837_04728 | EQT43961 | protein fimH | Escherichia coli HVH 185 (4-2876639) | 815.5 | 1.10E-241 |
| FimH | 66 | G772_04514 | EQQ70526 | protein fimH | Escherichia coli HVH 111 (4-7039018) | 815.5 | 1.10E-241 |
| FimH | 67 | G785_04660 | EQR38181 | protein fimH | Escherichia coli HVH 125 (4-2634716) | 815.5 | 1.10E-241 |
| FimH | 68 | G975_03705 | ETF33547 | protein fimH | Escherichia coli UMEA 3489-1 | 815.5 | 1.10E-241 |
| FimH | 69 | A199_00092 | ELD92815 | protein fimH | Escherichia coli KTE237 | 815.5 | 1.10E-241 |
| FimH | 70 | G888_04552 | EQV60910 | protein fimH | Escherichia coli KOEGE 58 (171a) | 815.5 | 1.10E-241 |
| FimH | 71 | OQA_21628 | EIA33925 | type 1 fimbiral adhesin FimH | Escherichia coli SCI-07 | 815.5 | 1.10E-241 |
| FimH | 72 | AB14_4668 | KDW70404 | protein fimH | Escherichia coli 1-392-07_S1_C1 | 815.5 | 1.10E-241 |
| FimH | 73 | L913_3471 | ESA25194 | mannose-specific adhesin FimH | Escherichia coli SCD2 | 815.5 | 1.10E-241 |
| FimH | 74 | AB79_5088 | KEM48577 | protein fimH | Escherichia coli 6-175-07_S1_C3 | 815.5 | 1.10E-241 |
| FimH | 75 | A13Y_00296 | ELH19709 | fimbrial protein FimH | Escherichia coli KTE194 | 815.5 | 1.10E-241 |
| FimH | 76 | ERS139229_02185 | CTW91752 | FimH protein | Escherichia coli HVH 41 (4-2677849) | 815.5 | 1.10E-241 |
| FimH | 77 | WGG_04597 | ELF78856 | fimbrial protein FimH | Escherichia coli KTE43 | 815.5 | 1.10E-241 |
| FimH | 78 | WI9_04462 | ELI04434 | fimbrial protein FimH | Escherichia coli KTE106 | 815.5 | 1.10E-241 |
| FimH | 79 | A1UG_04873 | ELE46761 | protein fimH | Escherichia coli KTE72 | 815.5 | 1.10E-241 |
| FimH | 80 | G877_04640 | ERA85988 | protein fimH | Escherichia coli HVH 228 (4-7787030) | 815.5 | 1.10E-241 |
| FimH | 81 | G973_04629 | EQZ11174 | protein fimH | Escherichia coli UMEA 3391-1 | 815.5 | 1.10E-241 |
| FimH | 82 | G910_04292 | EQW69328 | protein fimH | Escherichia coli UMEA 3117-1 | 815.5 | 1.10E-241 |
| FimH | 83 | G948_04673 | EQY34880 | protein fimH | Escherichia coli UMEA 3221-1 | 815.5 | 1.10E-241 |
| FimH | 84 | WCU_04653 | ELC23594 | protein fimH | Escherichia coli KTE15 | 815.5 | 1.10E-241 |
| FimH | 85 | NRG857_21795 | ADR29766 | type 1 fimbiral adhesin FimH | Escherichia coli O83:H1 str. NRG 857C | 815.5 | 1.10E-241 |
| FimH | 86 | WIA_00007 | ELI21490 | fimbrial protein FimH | Escherichia coli KTE109 | 815.5 | 1.10E-241 |
| FimH | 87 | A1W9_04489 | EOV85038 | fimbrial protein FimH | Escherichia coli KTE89 | 815.5 | 1.10E-241 |
| FimH | 88 | WKY_04689 | ELJ47448 | fimbrial protein FimH | Escherichia coli KTE180 | 815.5 | 1.10E-241 |
| FimH | 89 | A13O_00080 | ELC87055 | protein fimH | Escherichia coli KTE189 | 815.5 | 1.10E-241 |
| FimH | 90 | G988_04406 | ESK23472 | protein fimH | Escherichia coli UMEA 3693-1 | 815.5 | 1.10E-241 |
| FimH | 91 | WI5_04538 | ELI01603 | fimbrial protein FimH | Escherichia coli KTE104 | 815.5 | 1.10E-241 |
| FimH | 92 | G983_04262 | EQZ47992 | protein fimH | Escherichia coli UMEA 3656-1 | 815.5 | 1.10E-241 |
| FimH | 93 | G743_01797 | EQP82369 | protein fimH | Escherichia coli HVH 80 (4-2428830) | 815.5 | 1.10E-241 |
| FimH | 94 | G727_04803 | EQO93570 | protein fimH | Escherichia coli HVH 55 (4-2646161) | 815.5 | 1.10E-241 |
| FimH | 95 | G699_03935 | ETF18711 | protein fimH | Escherichia coli HVH 23 (4-6066488) | 815.5 | 1.10E-241 |
| FimH | 96 | A1W5_00063 | ELE85061 | protein fimH | Escherichia coli KTE86 | 815.5 | 1.10E-241 |
| FimH | 97 | AC80_5203 | EYD96380 | protein fimH | Escherichia coli 1-110-08_S4_C1 | 815.5 | 1.10E-241 |
| FimH | 98 | APECO1_2110 | ABJ03827 | type 1 fimbiral adhesin FimH | Escherichia coli O1:K1 / APEC | 815.4 | 1.20E-241 |
| FimH | 99 | HMPREF1604_02604 | ESD40670 | protein FimH | Escherichia coli 908519 | 815.4 | 1.20E-241 |
| FimH | 100 | HMPREF1622_05056 | ESE27383 | protein FimH | Escherichia coli A35218R | 815.4 | 1.20E-241 |
| FimH | 101 | HMPREF9545_02686 | EFU57535 | fimbrial protein | Escherichia coli MS 16-3 | 815.4 | 1.20E-241 |
| FimH | 102 | HMPREF9533_03081 | EGB82096 | fimbrial protein | Escherichia coli MS 60-1 | 815.4 | 1.20E-241 |
| FimH | 103 | ECP_4655 | ABG72591 | FimH protein precursor | Escherichia coli 536 | 815.4 | 1.20E-241 |
| FimH | 104 | HMPREF9532_05240 | EGB74351 | fimbrial protein | Escherichia coli MS 57-2 | 815.4 | 1.20E-241 |
| FimH | 105 | AC15_4772 | KDW27023 | protein fimH | Escherichia coli 2-156-04_S3_C2 | 813 | 6.30E-241 |
| FimH | 106 | UC40_06820 | KJG98289 | fimbrial protein | Escherichia coli | 812.8 | 7.00E-241 |
| FimH | 107 | WE1_00517 | ELF60578 | fimbrial protein FimH | Escherichia coli KTE17 | 812.8 | 7.00E-241 |
| FimH | 108 | WEI_00576 | ELC40118 | protein fimH | Escherichia coli KTE25 | 812.8 | 7.00E-241 |
| FimH | 109 | ECSTEC7V_5033 | EGE61947 | protein fimH | Escherichia coli STEC_7v | 812.8 | 7.00E-241 |
| FimH | 110 | WE3_00338 | ELF68615 | fimbrial protein FimH | Escherichia coli KTE18 | 812.8 | 7.00E-241 |
| FimH | 111 | A179_00610 | ELH78765 | fimbrial protein FimH | Escherichia coli KTE217 | 812.8 | 7.00E-241 |
| FimH | 112 | WK9_04511 | ELI91236 | fimbrial protein FimH | Escherichia coli KTE150 | 812.8 | 7.00E-241 |
| FimH | 113 | G820_04533 | EQS74809 | protein fimH | Escherichia coli HVH 162 (4-5627982) | 812.8 | 7.00E-241 |
| FimH | 114 | ERJG_03169 | EGB60884 | FimH protein | Escherichia coli M863 | 812.8 | 7.00E-241 |
| FimH | 115 | WEE_00340 | ELF77872 | fimbrial protein FimH | Escherichia coli KTE23 | 812.8 | 7.00E-241 |
| FimH | 116 | ECAA86_00023 | EGH38266 | mannose-specific adhesin FimH | Escherichia coli AA86 | 812.8 | 7.00E-241 |
| FimH | 117 | G921_02637 | EQX06252 | protein fimH | Escherichia coli UMEA 3155-1 | 812.8 | 7.00E-241 |
| FimH | 118 | EC12741_5433 | EIG79764 | mannose-binding domain protein FimH | Escherichia coli 1.2741 | 812.8 | 7.00E-241 |
| FimH | 119 | G842_00617 | EQT71821 | protein fimH | Escherichia coli HVH 190 (4-3255514) | 812.8 | 7.00E-241 |
| FimH | 120 | H004_04673 | ERA41269 | protein fimH | Escherichia coli UMEA 4207-1 | 812.8 | 7.10E-241 |
| FimH | 121 | G885_04654 | EQV47265 | protein fimH | Escherichia coli KOEGE 43 (105a) | 812.8 | 7.10E-241 |
| FimH | 122 | G971_04697 | ESK23911 | protein fimH | Escherichia coli UMEA 3342-1 | 812.8 | 7.10E-241 |
| FimH | 123 | G886_04558 | EQV52131 | protein fimH | Escherichia coli KOEGE 44 (106a) | 812.8 | 7.10E-241 |
| FimH | 124 | G889_04784 | EQV61618 | protein fimH | Escherichia coli KOEGE 61 (174a) | 812.8 | 7.10E-241 |
| FimH | 125 | G989_04763 | EQZ71478 | protein fimH | Escherichia coli UMEA 3694-1 | 812.8 | 7.10E-241 |
| FimH | 126 | G987_04605 | EQZ63556 | protein fimH | Escherichia coli UMEA 3687-1 | 812.8 | 7.10E-241 |
| FimH | 127 | G996_04802 | ERA02709 | protein fimH | Escherichia coli UMEA 3821-1 | 812.8 | 7.10E-241 |
| FimH | 128 | G934_04683 | EQX61870 | protein fimH | Escherichia coli UMEA 3185-1 | 812.8 | 7.10E-241 |
| FimH | 129 | A17M_04701 | ELD47225 | protein fimH | Escherichia coli KTE224 | 812.8 | 7.10E-241 |
| FimH | 130 | A31M_04712 | ELF33611 | protein fimH | Escherichia coli KTE169 | 812.8 | 7.10E-241 |
| FimH | 131 | WKO_04581 | ELJ21565 | fimbrial protein FimH | Escherichia coli KTE168 | 812.8 | 7.10E-241 |
| FimH | 132 | ECIG_03251 | EGI12993 | FimH protein | Escherichia coli M605 | 812.8 | 7.30E-241 |
| FimH | 133 | A17A_00746 | ELH90468 | fimbrial protein FimH | Escherichia coli KTE218 | 812.7 | 7.90E-241 |
| FimH | 134 | G947_04732 | EQY26009 | protein fimH | Escherichia coli UMEA 3220-1 | 812.7 | 7.90E-241 |
| FimH | 135 | G712_04776 | EQO31698 | protein fimH | Escherichia coli HVH 37 (4-2773848) | 812.7 | 7.90E-241 |
| FimH | 136 | G818_04698 | ERA80037 | protein fimH | Escherichia coli HVH 160 (4-5695937) | 812.7 | 7.90E-241 |
| FimH | 137 | G770_04974 | EQQ62239 | protein fimH | Escherichia coli HVH 109 (4-6977162) | 812.7 | 7.90E-241 |
| FimH | 138 | AC24_4837 | KEN68263 | protein fimH | Escherichia coli 8-415-05_S3_C2 | 812.7 | 7.90E-241 |
| FimH | 139 | G754_04626 | EQQ19315 | protein fimH | Escherichia coli HVH 92 (4-5930790) | 812.7 | 7.90E-241 |
| FimH | 140 | G955_04667 | EQY63793 | protein fimH | Escherichia coli UMEA 3257-1 | 812.7 | 7.90E-241 |
| FimH | 141 | G707_04588 | EQO10958 | protein fimH | Escherichia coli HVH 31 (4-2602156) | 812.7 | 7.90E-241 |
| FimH | 142 | G824_04718 | EQS91274 | protein fimH | Escherichia coli HVH 169 (4-1075578) | 812.7 | 7.90E-241 |
| FimH | 143 | G982_04762 | ERF50447 | protein fimH | Escherichia coli UMEA 3652-1 | 812.7 | 7.90E-241 |
| FimH | 144 | G764_04666 | EQQ45245 | protein fimH | Escherichia coli HVH 103 (4-5904188) | 812.7 | 7.90E-241 |
| FimH | 145 | G930_04740 | EQX39407 | protein fimH | Escherichia coli UMEA 3175-1 | 812.7 | 7.90E-241 |
| FimH | 146 | WIY_00010 | ELI80471 | fimbrial protein FimH | Escherichia coli KTE137 | 812.7 | 7.90E-241 |
| FimH | 147 | A15S_02513 | ELH63267 | fimbrial protein FimH | Escherichia coli KTE209 | 812.7 | 7.90E-241 |
| FimH | 148 | G779_04917 | EQR10066 | protein fimH | Escherichia coli HVH 117 (4-6857191) | 812.7 | 7.90E-241 |
| FimH | 149 | G782_04542 | EQR23656 | protein fimH | Escherichia coli HVH 120 (4-6978681) | 812.7 | 7.90E-241 |
| FimH | 150 | G751_04754 | EQP98060 | protein fimH | Escherichia coli HVH 89 (4-5885604) | 812.7 | 7.90E-241 |
| FimH | 151 | G703_04526 | EQN89185 | protein fimH | Escherichia coli HVH 27 (4-7449267) | 812.7 | 7.90E-241 |
| FimH | 152 | G757_04771 | EQQ30961 | protein fimH | Escherichia coli HVH 96 (4-5934869) | 812.7 | 7.90E-241 |
| FimH | 153 | G724_04664 | EQO85499 | protein fimH | Escherichia coli HVH 51 (4-2172526) | 812.7 | 7.90E-241 |
| FimH | 154 | AC54_0007 | KEN35984 | protein fimH | Escherichia coli 8-415-05_S3_C3 | 812.7 | 7.90E-241 |
| FimH | 155 | AC79_4762 | KEJ05046 | protein fimH | Escherichia coli 8-415-05_S4_C1 | 812.7 | 7.90E-241 |
| FimH | 156 | G864_04664 | EQU66587 | protein fimH | Escherichia coli HVH 212 (3-9305343) | 812.7 | 7.90E-241 |
| FimH | 157 | G704_04777 | EQO01218 | protein fimH | Escherichia coli HVH 28 (4-0907367) | 812.7 | 7.90E-241 |
| FimH | 158 | G819_04940 | EQS60389 | protein fimH | Escherichia coli HVH 161 (4-3119890) | 812.7 | 7.90E-241 |
| FimH | 159 | AB96_0008 | KEN45784 | protein fimH | Escherichia coli 8-415-05_S3_C1 | 812.7 | 7.90E-241 |
| FimH | 160 | G731_04483 | EQP15096 | protein fimH | Escherichia coli HVH 61 (4-2736020) | 812.7 | 7.90E-241 |
| FimH | 161 | G868_04582 | EQU88623 | protein fimH | Escherichia coli HVH 216 (4-3042952) | 812.7 | 7.90E-241 |
| FimH | 162 | WKA_04534 | ELI96098 | fimbrial protein FimH | Escherichia coli KTE153 | 812.7 | 7.90E-241 |
| FimH | 163 | SK69_04220 | KLX11202 | protein FimH | Escherichia coli | 812.7 | 7.90E-241 |
| FimH | 164 | G993_04639 | EQZ84781 | protein fimH | Escherichia coli UMEA 3707-1 | 812.7 | 7.90E-241 |
| FimH | 165 | G729_04813 | EQP06018 | protein fimH | Escherichia coli HVH 58 (4-2839709) | 812.7 | 7.90E-241 |
| FimH | 166 | AD07_0007 | KEJ16740 | protein fimH | Escherichia coli 8-415-05_S4_C2 | 812.7 | 7.90E-241 |
| FimH | 167 | WCI_04704 | ELF43362 | fimbrial protein FimH | Escherichia coli KTE8 | 812.7 | 7.90E-241 |
| FimH | 168 | AD36_0007 | KEJ35773 | protein fimH | Escherichia coli 8-415-05_S4_C3 | 812.7 | 7.90E-241 |
| FimH | 169 | G687_04691 | EQN26436 | protein fimH | Escherichia coli HVH 7 (4-7315031) | 812.7 | 7.90E-241 |
| FimH | 170 | G756_04706 | EQQ20725 | protein fimH | Escherichia coli HVH 95 (4-6074464) | 812.7 | 7.90E-241 |
| FimH | 171 | G775_04558 | EQQ82681 | protein fimH | Escherichia coli HVH 114 (4-7037740) | 812.7 | 7.90E-241 |
| FimH | 172 | G970_04595 | EQZ06495 | protein fimH | Escherichia coli UMEA 3341-1 | 812.6 | 8.00E-241 |
| FimH | 173 | i02_4917 | AER87432 | FimH protein precursor | Escherichia coli str. 'clone D i2' | 812.6 | 8.20E-241 |
| FimH | 174 | HMPREF9531_01596 | EFJ93321 | fimbrial protein | Escherichia coli MS 45-1 | 812.6 | 8.20E-241 |
| FimH | 175 | HMPREF3041_03035 | KXG93807 | protein FimH | Escherichia coli O6:H1 (strain CFT073 / ATCC 700928 / UPEC) | 812.6 | 8.20E-241 |
| FimH | 176 | HMPREF1603_02700 | ESD37388 | protein FimH | Escherichia coli 907892 | 812.6 | 8.20E-241 |
| FimH | 177 | c5400 | AAN83822 | FimH protein precursor | Escherichia coli CFT073 | 812.6 | 8.20E-241 |
| FimH | 178 | i14_4917 | AER92351 | FimH protein precursor | Escherichia coli str. 'clone D i14' | 812.6 | 8.20E-241 |
| FimH | 179 | HMPREF9544_02307 | EFU52578 | fimbrial protein | Escherichia coli MS 153-1 | 812.6 | 8.20E-241 |
| FimH | 180 | T638_06400 | KKJ21621 | fimbrial protein | Escherichia coli MRSN 10204 | 812.5 | 9.00E-241 |
| FimH | 181 | G903_04604 | EQW36500 | protein fimH | Escherichia coli UMEA 3053-1 | 812.5 | 9.00E-241 |
| FimH | 182 | G714_04554 | EQO38165 | protein fimH | Escherichia coli HVH 39 (4-2679949) | 812.5 | 9.00E-241 |
| FimH | 183 | G924_04725 | EQX14147 | protein fimH | Escherichia coli UMEA 3161-1 | 812.5 | 9.00E-241 |
| FimH | 184 | A15M_00067 | ELD17304 | protein fimH | Escherichia coli KTE206 | 812.5 | 9.00E-241 |
| FimH | 185 | WIU_04550 | ELI61786 | fimbrial protein FimH | Escherichia coli KTE131 | 812.5 | 9.00E-241 |
| FimH | 186 | EC91649_4802 | KKA61881 | mannose-binding domain protein FimH | Escherichia coli 9.1649 | 812.5 | 9.00E-241 |
| FimH | 187 | AF56_03926 | EZQ56163 | protein fimH | Escherichia coli BIDMC 83 | 812.5 | 9.00E-241 |
| FimH | 188 | A15O_00589 | ELH67504 | fimbrial protein FimH | Escherichia coli KTE207 | 812.5 | 9.00E-241 |
| FimH | 189 | G745_02969 | ETF19741 | protein fimH | Escherichia coli HVH 83 (4-2051087) | 812.5 | 9.00E-241 |
| FimH | 190 | H002_04797 | ERA28390 | protein fimH | Escherichia coli UMEA 4075-1 | 812.5 | 9.00E-241 |
| FimH | 191 | G953_04600 | EQY50164 | protein fimH | Escherichia coli UMEA 3244-1 | 812.5 | 9.00E-241 |
| FimH | 192 | G981_04511 | EQZ45613 | protein fimH | Escherichia coli UMEA 3632-1 | 812.5 | 9.00E-241 |
| FimH | 193 | AB16_1136 | KDZ53180 | protein fimH | Escherichia coli 3-073-06_S1_C1 | 812.5 | 9.00E-241 |
| FimH | 194 | EC07798_0009 | EKI46128 | fimH | Escherichia coli 07798 | 812.5 | 9.00E-241 |
| FimH | 195 | G748_04789 | ESP14116 | protein fimH | Escherichia coli HVH 86 (4-7026218) | 812.5 | 9.00E-241 |
| FimH | 196 | ERS139252_00950 | CTX20581 | FimH protein | Escherichia coli | 812.5 | 9.00E-241 |
| FimH | 197 | WIW_04599 | ELI65835 | fimbrial protein FimH | Escherichia coli KTE133 | 812.5 | 9.00E-241 |
| FimH | 198 | G691_04689 | EQN40439 | protein fimH | Escherichia coli HVH 13 (4-7634056) | 812.5 | 9.00E-241 |
| FimH | 199 | G932_04713 | EQX51233 | protein fimH | Escherichia coli UMEA 3178-1 | 812.5 | 9.00E-241 |
| FimH | 200 | G826_04537 | EQS94734 | protein fimH | Escherichia coli HVH 171 (4-3191958) | 812.5 | 9.00E-241 |
| FimH | 201 | G876_04718 | EQV20944 | protein fimH | Escherichia coli HVH 227 (4-2277670) | 812.5 | 9.00E-241 |
| FimH | 202 | EcF11_0633 | EDV68689 | protein FimH | Escherichia coli F11 | 812.5 | 9.00E-241 |
| FimH | 203 | WK5_00007 | ELI92894 | fimbrial protein FimH | Escherichia coli KTE145 | 812.5 | 9.00E-241 |
| FimH | 204 | A175_00083 | ELH86323 | fimbrial protein FimH | Escherichia coli KTE215 | 812.5 | 9.00E-241 |
| FimH | 205 | EcoM_02399 | EFW69891 | mannose-specific adhesin FimH | Escherichia coli WV_060327 | 812.3 | 1.00E-240 |
| FimH | 206 | G920_04335 | EQW97666 | protein fimH | Escherichia coli UMEA 3152-1 | 812.3 | 1.00E-240 |
| FimH | 207 | H003_04426 | ERA40517 | protein fimH | Escherichia coli UMEA 4076-1 | 812.3 | 1.00E-240 |
| FimH | 208 | ERS085395_03365 | CTV09222 | FimH protein | Escherichia coli | 812.3 | 1.00E-240 |
| FimH | 209 | AB22_1075 | KEL52084 | protein fimH | Escherichia coli 6-175-07_S1_C1 | 812.3 | 1.00E-240 |
| FimH | 210 | ECTW07793_4525 | EII94938 | mannose-binding domain protein FimH | Escherichia coli TW07793 | 812.3 | 1.00E-240 |
| FimH | 211 | ECKG_03439 | EGI24020 | FimH protein | Escherichia coli TA206 | 812.3 | 1.10E-240 |
| FimH | 212 | G881_04718 | EQV27997 | protein fimH | Escherichia coli KOEGE 30 (63a) | 812.2 | 1.10E-240 |
| FimH | 213 | G942_04648 | EQY06496 | protein fimH | Escherichia coli UMEA 3208-1 | 812.2 | 1.10E-240 |
| FimH | 214 | G814_04742 | ERA63757 | protein fimH | Escherichia coli HVH 156 (4-3206505) | 812.2 | 1.10E-240 |
| FimH | 215 | G951_04723 | EQY50640 | protein fimH | Escherichia coli UMEA 3233-1 | 812.2 | 1.10E-240 |
| FimH | 216 | G959_04576 | ESK11759 | protein fimH | Escherichia coli UMEA 3290-1 | 812.2 | 1.10E-240 |
| FimH | 217 | G846_04702 | EQT91090 | protein fimH | Escherichia coli HVH 194 (4-2356805) | 812.2 | 1.10E-240 |
| FimH | 218 | G816_04477 | EQS57105 | protein fimH | Escherichia coli HVH 158 (4-3224287) | 812.2 | 1.10E-240 |
| FimH | 219 | G928_04652 | EQX36221 | protein fimH | Escherichia coli UMEA 3173-1 | 812.2 | 1.10E-240 |
| FimH | 220 | WG9_00385 | ELC59627 | protein fimH | Escherichia coli KTE39 | 812.2 | 1.10E-240 |
| FimH | 221 | G957_04753 | EQY71513 | protein fimH | Escherichia coli UMEA 3268-1 | 812.2 | 1.10E-240 |
| FimH | 222 | G969_04783 | EQY95087 | protein fimH | Escherichia coli UMEA 3337-1 | 812.2 | 1.10E-240 |
| FimH | 223 | G980_04563 | EQZ33064 | protein fimH | Escherichia coli UMEA 3617-1 | 812.2 | 1.10E-240 |
| FimH | 224 | G956_04756 | EQY63448 | protein fimH | Escherichia coli UMEA 3264-1 | 812.2 | 1.10E-240 |
| FimH | 225 | G976_04783 | EQZ18336 | protein fimH | Escherichia coli UMEA 3490-1 | 812.2 | 1.10E-240 |
| FimH | 226 | A173_00895 | ELD42549 | protein fimH | Escherichia coli KTE214 | 812.2 | 1.10E-240 |
| FimH | 227 | G713_04628 | EQO38363 | protein fimH | Escherichia coli HVH 38 (4-2774682) | 812.2 | 1.10E-240 |
| FimH | 228 | G907_04452 | EQW50325 | protein fimH | Escherichia coli UMEA 3097-1 | 812.2 | 1.10E-240 |
| FimH | 229 | ECABU_c49570 | ADN49333 | type 1 fimbiral adhesin FimH | Escherichia coli ABU 83972 | 812.1 | 1.20E-240 |
| FimH | 230 | HMPREF0358_1784 | EEJ48487 | fimbrial protein | Escherichia coli 83972 | 812.1 | 1.20E-240 |
| FimH | 231 | LF82_0665 | CAP78801 | Protein fimH | Escherichia coli LF82 | 811.9 | 1.30E-240 |
| FimH | 232 | A1YS_00318 | ELG82374 | fimbrial protein FimH | Escherichia coli KTE141 | 811.9 | 1.30E-240 |
| FimH | 233 | WI1_04350 | ELJ90246 | fimbrial protein FimH | Escherichia coli KTE97 | 811.9 | 1.30E-240 |
| FimH | 234 | A13K_00356 | ELC76557 | protein fimH | Escherichia coli KTE187 | 811.9 | 1.30E-240 |
| FimH | 235 | A1SE_00356 | ELE08299 | protein fimH | Escherichia coli KTE53 | 811.9 | 1.40E-240 |
| FimH | 236 | ERS139266_04444 | CTY68185 | FimH protein | Escherichia coli (GCA_001277595) | 811.6 | 1.70E-240 |
| FimH | 237 | HMPREF9553_04102 | EFJ59828 | fimbrial protein | Escherichia coli MS 200-1 | 810.5 | 3.60E-240 |
| FimH | 238 | G887_04645 | EQV61282 | protein fimH | Escherichia coli KOEGE 56 (169a) | 810.3 | 4.20E-240 |
| FimH | 239 | G856_04406 | EQU34073 | protein fimH | Escherichia coli HVH 204 (4-3112802) | 810.2 | 4.50E-240 |
| FimH | 240 | XB01_16990 | KLD45834 | fimbrial protein | Escherichia coli (GCA_001010195) | 810.1 | 4.60E-240 |
| FimH | 241 | AL530_23775 | KTK77803 | fimbrial protein | Escherichia fergusonii | 810 | 5.10E-240 |
| FimH | 242 | G827_04756 | EQT05654 | protein fimH | Escherichia coli HVH 172 (4-3248542) | 810 | 5.10E-240 |
| FimH | 243 | G911_04808 | EQW72698 | protein fimH | Escherichia coli UMEA 3121-1 | 810 | 5.10E-240 |
| FimH | 244 | G949_04827 | EQY35945 | protein fimH | Escherichia coli UMEA 3222-1 | 810 | 5.10E-240 |
| FimH | 245 | G738_04690 | EQP44607 | protein fimH | Escherichia coli HVH 74 (4-1034782) | 810 | 5.10E-240 |
| FimH | 246 | G721_04524 | EQO77496 | protein fimH | Escherichia coli HVH 46 (4-2758776) | 810 | 5.10E-240 |
| FimH | 247 | G811_04679 | EQS44724 | protein fimH | Escherichia coli HVH 153 (3-9344314) | 810 | 5.10E-240 |
| FimH | 248 | ECNC101_02808 | EFM51613 | type 1 fimbiral adhesin FimH | Escherichia coli NC101 | 809.9 | 5.50E-240 |
| FimH | 249 | G815_04576 | ERA65098 | protein fimH | Escherichia coli HVH 157 (4-3406229) | 809.8 | 5.60E-240 |
| FimH | 250 | G802_04869 | EQS12127 | protein fimH | Escherichia coli HVH 144 (4-4451937) | 809.8 | 5.60E-240 |
| FimH | 251 | G870_04565 | EQU95733 | protein fimH | Escherichia coli HVH 218 (4-4500903) | 809.8 | 5.60E-240 |
| FimH | 252 | G871_04507 | EQV03589 | protein fimH | Escherichia coli HVH 220 (4-5876842) | 809.8 | 5.60E-240 |
| FimH | 253 | G817_04756 | ERA71841 | protein fimH | Escherichia coli HVH 159 (4-5818141) | 809.8 | 5.60E-240 |
| FimH | 254 | G790_04604 | EQR60833 | protein fimH | Escherichia coli HVH 132 (4-6876862) | 809.8 | 5.60E-240 |
| FimH | 255 | G812_04525 | EQS59387 | protein fimH | Escherichia coli HVH 154 (4-5636698) | 809.8 | 5.70E-240 |
| FimH | 256 | G693_04564 | EQN48780 | protein fimH | Escherichia coli HVH 17 (4-7473087) | 809.8 | 5.70E-240 |
| FimH | 257 | G769_04519 | ESP40224 | protein fimH | Escherichia coli HVH 108 (4-6924867) | 809.8 | 5.70E-240 |
| FimH | 258 | BBZ52_23550 | OCS66147 | fimbrial protein | Escherichia coli | 809.8 | 5.70E-240 |
| FimH | 259 | G831_04207 | ETF14368 | protein fimH | Escherichia coli HVH 177 (4-2876612) | 809.8 | 5.70E-240 |
| FimH | 260 | G752_04706 | EQQ09828 | protein fimH | Escherichia coli HVH 90 (4-3191362) | 809.8 | 5.70E-240 |
| FimH | 261 | WCG_02044 | ELF46857 | fimbrial protein FimH | Escherichia coli KTE6 | 809.8 | 5.70E-240 |
| FimH | 262 | G804_04805 | EQS28810 | protein fimH | Escherichia coli HVH 146 (4-3189767) | 809.8 | 5.70E-240 |
| FimH | 263 | A137_00363 | ELC68318 | protein fimH | Escherichia coli KTE178 | 809.8 | 5.70E-240 |
| FimH | 264 | G854_04911 | EQU28360 | protein fimH | Escherichia coli HVH 202 (4-3163997) | 809.8 | 5.70E-240 |
| FimH | 265 | G799_04639 | EQR98465 | protein fimH | Escherichia coli HVH 141 (4-5995973) | 809.8 | 5.70E-240 |
| FimH | 266 | EH65_22685 | KEP01862 | fimbrial protein | Escherichia coli str. UCD_JA17 | 809.8 | 5.80E-240 |
| FimH | 267 | WIM_00022 | ELI48268 | fimbrial protein FimH | Escherichia coli KTE124 | 809.7 | 6.40E-240 |
| FimH | 268 | G740_04507 | EQP64010 | protein fimH | Escherichia coli HVH 77 (4-2605759) | 809.7 | 6.40E-240 |
| FimH | 269 | A15C_00482 | ELD03192 | protein fimH | Escherichia coli KTE201 | 809.7 | 6.40E-240 |
| FimH | 270 | WC5_01844 | EOU45989 | fimbrial protein FimH | Escherichia sp. KTE114 | 809.7 | 6.40E-240 |
| FimH | 271 | BW82_01380 | EYZ78819 | fimbrial protein | Escherichia coli O111:NM str. 04-3211 | 809.7 | 6.40E-240 |
| FimH | 272 | BX76_13255 | EYY67473 | fimbrial protein | Escherichia coli O111:NM str. 2010C-4799 | 809.7 | 6.40E-240 |
| FimH | 273 | EC08BKT77219_5188 | ERE00904 | protein fimH | Escherichia coli 08BKT77219 | 809.7 | 6.40E-240 |
| FimH | 274 | A313_03080 | ELG89867 | fimbrial protein FimH | Escherichia coli KTE147 | 809.7 | 6.40E-240 |
| FimH | 275 | BY53_05145 | EZB18161 | fimbrial protein | Escherichia coli O157:H7 str. F7384 | 809.7 | 6.40E-240 |
| FimH | 276 | ECSE_4593 | BAG80117 | type-1 fimbrial minor subunit FimH | Escherichia coli (strain SE11) | 809.7 | 6.40E-240 |
| FimH | 277 | ECP02994833_4750 | END76837 | protein fimH | Escherichia coli P0299483.3 | 809.7 | 6.40E-240 |
| FimH | 278 | SS323385_5077 | EIQ37827 | protein fimH | Shigella sonnei 3233-85 | 809.7 | 6.40E-240 |
| FimH | 279 | BY41_13805 | EYV93467 | fimbrial protein | Escherichia coli O86:H34 str. 99-3124 | 809.7 | 6.40E-240 |
| FimH | 280 | ECDEC5D_0196 | EHV46618 | minor fimbrial subunit, D-mannose specific adhesin | Escherichia coli DEC5D | 809.7 | 6.40E-240 |
| FimH | 281 | ECDEC14C_5180 | EHX82517 | minor fimbrial subunit, D-mannose specific adhesin | Escherichia coli DEC14C | 809.7 | 6.40E-240 |
| FimH | 282 | ECO55CA74_24670 | AEZ43466 | adhesin | Escherichia coli O55:H7 str. RM12579 | 809.7 | 6.40E-240 |
| FimH | 283 | B231_0105 | ERE45431 | protein fimH | Escherichia coli Tx3800 | 809.7 | 6.40E-240 |
| FimH | 284 | SGB_03682 | EFW54348 | mannose-specific adhesin FimH | Shigella boydii ATCC 9905 | 809.7 | 6.40E-240 |
| FimH | 285 | EC71982_0045 | ELW21826 | protein fimH | Escherichia coli 7.1982 | 809.7 | 6.40E-240 |
| FimH | 286 | ECP02989421_5129 | ENA07463 | protein fimH | Escherichia coli P0298942.1 | 809.7 | 6.40E-240 |
| FimH | 287 | ECDEC5C_5266 | EHV31702 | minor fimbrial subunit, D-mannose specific adhesin | Escherichia coli DEC5C | 809.7 | 6.40E-240 |
| FimH | 288 | EC990678_0101 | EKW96678 | type 1 fimbrial adhesin | Escherichia coli 99.0678 | 809.7 | 6.40E-240 |
| FimH | 289 | ECOK1180_4902 | EFZ61803 | protein fimH | Escherichia coli OK1180 | 809.7 | 6.40E-240 |
| FimH | 290 | BW89_03540 | EYZ63311 | fimbrial protein | Escherichia coli O55:H7 str. 06-3555 | 809.7 | 6.40E-240 |
| FimH | 291 | G908_04271 | EQW62041 | protein fimH | Escherichia coli UMEA 3108-1 | 809.7 | 6.40E-240 |
| FimH | 292 | XF37_26830 | KKF80418 | fimbrial protein | Escherichia coli O157:H7 (GCA_001006425) | 809.7 | 6.40E-240 |
| FimH | 293 | EC950183_0023 | EKW36788 | protein fimH | Escherichia coli 95.0183 | 809.7 | 6.40E-240 |
| FimH | 294 | BY00_02650 | EYY03491 | fimbrial protein | Escherichia coli O121:H19 str. 2011C-3500 | 809.7 | 6.40E-240 |
| FimH | 295 | BX24_08685 | EZE65819 | fimbrial protein | Escherichia coli O91:H21 str. 2009C-4646 | 809.7 | 6.40E-240 |
| FimH | 296 | ECPA34_0037 | EKH20939 | type 1 fimbrial adhesin | Escherichia coli PA34 | 809.7 | 6.40E-240 |
| FimH | 297 | BX62_24885 | EYU80171 | fimbrial protein | Escherichia coli O121:H19 str. 2010C-4254 | 809.7 | 6.40E-240 |
| FimH | 298 | ECP030477714_4798 | ENE64853 | protein fimH | Escherichia coli P0304777.14 | 809.7 | 6.40E-240 |
| FimH | 299 | BY36_24440 | EZF08542 | fimbrial protein | Escherichia coli O157:H7 str. 2011EL-2290 | 809.7 | 6.40E-240 |
| FimH | 300 | ECPA19_4658 | ELV78209 | protein fimH | Escherichia coli PA19 | 809.7 | 6.40E-240 |
| FimH | 301 | BW93_13935 | EYZ46476 | fimbrial protein | Escherichia coli O121:H19 str. 06-3822 | 809.7 | 6.40E-240 |
| FimH | 302 | ECC1470_03169 | EST87062 | adhesin | Escherichia coli ECC-1470 (GCA_000831565) | 809.7 | 6.40E-240 |
| FimH | 303 | EC30301_5114 | EGW77993 | protein fimH | Escherichia coli 3030-1 | 809.7 | 6.40E-240 |
| FimH | 304 | ECH7EC4076_4074 | EDU69751 | protein FimH | Escherichia coli O157:H7 str. EC4076 | 809.7 | 6.40E-240 |
| FimH | 305 | ECO7815_09103 | EFX22751 | adhesin | Escherichia coli O55:H7 str. 3256-97 | 809.7 | 6.40E-240 |
| FimH | 306 | BX50_13370 | EYV16921 | fimbrial protein | Escherichia coli O145:NM str. 2010C-3521 | 809.7 | 6.40E-240 |
| FimH | 307 | AB44_4694 | KDZ57179 | protein fimH | Escherichia coli 3-073-06_S1_C2 | 809.7 | 6.40E-240 |
| FimH | 308 | QYO_0037 | ERC15464 | protein fimH | Escherichia coli B29-1 | 809.7 | 6.40E-240 |
| FimH | 309 | SS17_5473 | AIF96955 | mannose-specific adhesin FimH | Escherichia coli O157:H7 str. SS17 | 809.7 | 6.40E-240 |
| FimH | 310 | G913_04375 | EQW81442 | protein fimH | Escherichia coli UMEA 3124-1 | 809.7 | 6.40E-240 |
| FimH | 311 | EC180200_4709 | ENA60809 | protein fimH | Escherichia coli 180200 | 809.7 | 6.40E-240 |
| FimH | 312 | AB12_4963 | EZK25500 | protein fimH | Escherichia coli 1-182-04_S1_C1 | 809.7 | 6.40E-240 |
| FimH | 313 | J444_4746 | AKP87396 | fimbrial protein | Escherichia coli ACN001 | 809.7 | 6.40E-240 |
| FimH | 314 | BW72_33380 | KDV18465 | fimbrial protein | Escherichia coli O78:H12 str. 00-3279 | 809.7 | 6.40E-240 |
| FimH | 315 | BX69_16245 | EYZ04641 | fimbrial protein | Escherichia coli O111:NM str. 2010C-4592 | 809.7 | 6.40E-240 |
| FimH | 316 | EC2845650_4920 | EMW13247 | protein fimH | Escherichia coli 2845650 | 809.7 | 6.40E-240 |
| FimH | 317 | ECP02994388_4890 | ENC18714 | protein fimH | Escherichia coli P0299438.8 | 809.7 | 6.40E-240 |
| FimH | 318 | EcHS_A4546 | ABV08699 | protein FimH | Escherichia coli HS | 809.7 | 6.40E-240 |
| FimH | 319 | ECTW09195_5731 | EIO86200 | type 1 fimbrial adhesin | Escherichia coli TW09195 | 809.7 | 6.40E-240 |
| FimH | 320 | ECTW09098_5720 | EIO65767 | type 1 fimbrial adhesin | Escherichia coli TW09098 | 809.7 | 6.40E-240 |
| FimH | 321 | EC33884_4999 | EII56449 | mannose-binding domain protein FimH | Escherichia coli 3.3884 | 809.7 | 6.40E-240 |
| FimH | 322 | ECP030477715_4766 | ENE71594 | protein fimH | Escherichia coli P0304777.15 | 809.7 | 6.40E-240 |
| FimH | 323 | BW79_08025 | EZA00181 | fimbrial protein | Escherichia coli O119:H4 str. 03-3458 | 809.7 | 6.40E-240 |
| FimH | 324 | EC2875000_4926 | EMV33041 | protein fimH | Escherichia coli 2875000 | 809.7 | 6.40E-240 |
| FimH | 325 | ERS139235_01099 | CTX12138 | FimH protein | Escherichia coli O55:H7 (strain CB9615 / EPEC) | 809.7 | 6.40E-240 |
| FimH | 326 | EC100869_5467 | EKK62172 | protein fimH | Escherichia coli 10.0869 | 809.7 | 6.40E-240 |
| FimH | 327 | BX54_06945 | EYV01818 | fimbrial protein | Escherichia coli O121:H19 str. 2010C-3840 | 809.7 | 6.40E-240 |
| FimH | 328 | ECDEC13C_5267 | EHX54316 | minor fimbrial subunit, D-mannose specific adhesin | Escherichia coli DEC13C | 809.7 | 6.40E-240 |
| FimH | 329 | ECPA47_5052 | ELV91663 | protein fimH | Escherichia coli PA47 | 809.7 | 6.40E-240 |
| FimH | 330 | QYS_4926 | ERC10681 | protein fimH | Escherichia coli B36-1 | 809.7 | 6.40E-240 |
| FimH | 331 | ECP029894211_4876 | ENB43046 | protein fimH | Escherichia coli P0298942.11 | 809.7 | 6.40E-240 |
| FimH | 332 | ECPA41_5705 | EIO29684 | type 1 fimbrial adhesin | Escherichia coli PA41 | 809.7 | 6.40E-240 |
| FimH | 333 | EC881467_0031 | EKV88787 | protein fimH | Escherichia coli 88.1467 | 809.7 | 6.40E-240 |
| FimH | 334 | ECP03047772_4721 | ENE74113 | protein fimH | Escherichia coli P0304777.2 | 809.7 | 6.40E-240 |
| FimH | 335 | WEQ_04196 | ELF82195 | fimbrial protein FimH | Escherichia coli KTE29 | 809.7 | 6.40E-240 |
| FimH | 336 | BW91_08685 | EYZ42980 | fimbrial protein | Escherichia coli O91:H14 str. 06-3691 | 809.7 | 6.40E-240 |
| FimH | 337 | ECP02994386_4896 | ENC09010 | protein fimH | Escherichia coli P0299438.6 | 809.7 | 6.40E-240 |
| FimH | 338 | BU56_08160 | KDV36600 | fimbrial protein | Escherichia coli O145:H25 str. 07-3858 | 809.7 | 6.40E-240 |
| FimH | 339 | S1G_5296 | ERC48282 | protein fimH | Escherichia coli B95 | 809.7 | 6.40E-240 |
| FimH | 340 | ECTW14313_5573 | EIP03321 | type 1 fimbrial adhesin | Escherichia coli O157:H7 str. TW14313 | 809.7 | 6.40E-240 |
| FimH | 341 | AC07_2909 | KEK76813 | protein fimH | Escherichia coli 3-475-03_S3_C1 | 809.7 | 6.40E-240 |
| FimH | 342 | G2583_5121 | ADD59535 | Adhesin | Escherichia coli O55:H7 str. CB9615 | 809.7 | 6.40E-240 |
| FimH | 343 | ECSTECO31_4923 | EJK93362 | protein fimH | Escherichia coli STEC_O31 | 809.7 | 6.40E-240 |
| FimH | 344 | ECMT8_17731 | EIL75080 | adhesin | Escherichia coli CUMT8 | 809.7 | 6.40E-240 |
| FimH | 345 | ECP030477713_4759 | ENE60389 | protein fimH | Escherichia coli P0304777.13 | 809.7 | 6.40E-240 |
| FimH | 346 | ECTW14301_5469 | EIP05619 | type 1 fimbrial adhesin | Escherichia coli TW14301 | 809.7 | 6.40E-240 |
| FimH | 347 | AC33_0306 | KDU02208 | protein fimH | Escherichia coli 3-267-03_S3_C2 | 809.7 | 6.40E-240 |
| FimH | 348 | BX83_07495 | EYY46560 | fimbrial protein | Escherichia coli O157:H7 str. 2010C-4979C1 | 809.7 | 6.40E-240 |
| FimH | 349 | ECP03047771_4674 | EMW95236 | protein fimH | Escherichia coli P0304777.1 | 809.7 | 6.40E-240 |
| FimH | 350 | ECP03048165_4876 | ENH33708 | protein fimH | Escherichia coli P0304816.5 | 809.7 | 6.40E-240 |
| FimH | 351 | G709_00505 | EQO36903 | protein fimH | Escherichia coli HVH 33 (4-2174936) | 809.7 | 6.40E-240 |
| FimH | 352 | ECRM12581_27345 | AHY73999 | mannose-specific adhesin FimH | Escherichia coli O145:H28 str. RM12581 | 809.7 | 6.40E-240 |
| FimH | 353 | ECH7EC4401_4311 | EDU76264 | protein FimH | Escherichia coli O157:H7 str. EC4401 | 809.7 | 6.40E-240 |
| FimH | 354 | ECEC1856_5611 | EKI92974 | type 1 fimbrial adhesin | Escherichia coli EC1856 | 809.7 | 6.40E-240 |
| FimH | 355 | EC08BKT55439_5140 | ERC53539 | protein fimH | Escherichia coli 08BKT055439 | 809.7 | 6.40E-240 |
| FimH | 356 | ECPA28_0037 | EIO09699 | type 1 fimbrial adhesin | Escherichia coli PA28 | 809.7 | 6.40E-240 |
| FimH | 357 | EC82524_5570 | EKK52354 | protein fimH | Escherichia coli 8.2524 | 809.7 | 6.40E-240 |
| FimH | 358 | ERS009833_03815 | CSQ02930 | protein FimH | Shigella sonnei | 809.7 | 6.40E-240 |
| FimH | 359 | ESNG_01155 | EHO01963 | fimH | Escherichia coli B093 | 809.7 | 6.40E-240 |
| FimH | 360 | ECKD2_14367 | EIL49994 | adhesin | Escherichia coli KD2 | 809.7 | 6.40E-240 |
| FimH | 361 | ECSP_5404 | ACT75083 | minor fimbrial subunit, D-mannose specific adhesin | Escherichia coli O157:H7 str. TW14359 | 809.7 | 6.40E-240 |
| FimH | 362 | S3I_0023 | ERD44407 | protein fimH | Escherichia coli B113 | 809.7 | 6.40E-240 |
| FimH | 363 | PPECC33_04820 | AKK51308 | Adhesin | Escherichia coli PCN033 | 809.7 | 6.40E-240 |
| FimH | 364 | BY59_23330 | EZB48656 | fimbrial protein | Escherichia coli O157:H7 str. K1420 | 809.7 | 6.40E-240 |
| FimH | 365 | BY46_04510 | EZA87036 | fimbrial protein | Escherichia coli O111:H8 str. F6627 | 809.7 | 6.40E-240 |
| FimH | 366 | WCQ_04453 | ELC17026 | protein fimH | Escherichia coli KTE12 | 809.7 | 6.40E-240 |
| FimH | 367 | EC990848_5096 | ELV35378 | protein fimH | Escherichia coli 99.0848 | 809.7 | 6.40E-240 |
| FimH | 368 | ECO9574_13279 | EIL19439 | FimH mannose-binding protein | Escherichia coli O111:H8 str. CVM9574 | 809.7 | 6.40E-240 |
| FimH | 369 | G783_04764 | EQR30152 | protein fimH | Escherichia coli HVH 121 (4-6877826) | 809.7 | 6.40E-240 |
| FimH | 370 | ECDEC3E_0023 | EHU85638 | minor fimbrial subunit, D-mannose specific adhesin | Escherichia coli DEC3E | 809.7 | 6.40E-240 |
| FimH | 371 | EC880221_0102 | EKK87688 | protein fimH | Escherichia coli 88.0221 | 809.7 | 6.40E-240 |
| FimH | 372 | AB08_0277 | KEL72198 | protein fimH | Escherichia coli 5-366-08_S1_C1 | 809.7 | 6.40E-240 |
| FimH | 373 | ECF_01256 | EGD69111 | mannose-specific adhesin FimH | Escherichia coli O157:H7 str. 1125 | 809.7 | 6.40E-240 |
| FimH | 374 | EC990816_5172 | ELV30855 | protein fimH | Escherichia coli 99.0816 | 809.7 | 6.40E-240 |
| FimH | 375 | ECH7EC4206_A4039 | EDZ78348 | protein FimH | Escherichia coli O157:H7 str. EC4206 | 809.7 | 6.40E-240 |
| FimH | 376 | EschWDRAFT_4529 | EFN35882 | FimH mannose-binding domain protein | Escherichia coli W (GCA_000258145) | 809.7 | 6.40E-240 |
| FimH | 377 | BY95_01985 | EZD19470 | fimbrial protein | Escherichia coli O157:H7 str. K6687 | 809.7 | 6.40E-240 |
| FimH | 378 | ECPA8_0036 | ELW12446 | protein fimH | Escherichia coli PA8 | 809.7 | 6.40E-240 |
| FimH | 379 | ECTW07945_0030 | EIO79490 | type 1 fimbrial adhesin | Escherichia coli TW07945 | 809.7 | 6.40E-240 |
| FimH | 380 | BY04_19085 | EYX86415 | fimbrial protein | Escherichia coli O156:H25 str. 2011C-3602 | 809.7 | 6.40E-240 |
| FimH | 381 | BW73_21750 | KDV16232 | fimbrial protein | Escherichia coli O111:NM str. 01-3076 | 809.7 | 6.40E-240 |
| FimH | 382 | ECFRIK920_5845 | EKG95805 | type 1 fimbrial adhesin | Escherichia coli FRIK920 | 809.7 | 6.40E-240 |
| FimH | 383 | EREG_02537 | EGB41881 | FimH protein | Escherichia coli H120 | 809.7 | 6.40E-240 |
| FimH | 384 | BX18_18300 | EZE39069 | fimbrial protein | Escherichia coli O111:NM str. 2009C-4006 | 809.7 | 6.40E-240 |
| FimH | 385 | QYG_5333 | ERC25331 | protein fimH | Escherichia coli B7-1 | 809.7 | 6.40E-240 |
| FimH | 386 | AC97_3726 | KDW42122 | protein fimH | Escherichia coli 2-177-06_S4_C2 | 809.7 | 6.40E-240 |
| FimH | 387 | G654_03742 | EWC57536 | adhesin | Escherichia coli EC096/10 | 809.7 | 6.40E-240 |
| FimH | 388 | BX32_22725 | EYV80101 | fimbrial protein | Escherichia coli O121:H19 str. 2009EL1412 | 809.7 | 6.40E-240 |
| FimH | 389 | O199_0202230 | ETJ70863 | fimbrial protein FimH | Escherichia coli ATCC 35150 | 809.7 | 6.40E-240 |
| FimH | 390 | ECH74115_5826 | ACI38701 | protein FimH | Escherichia coli O157:H7 str. EC4115 | 809.7 | 6.40E-240 |
| FimH | 391 | EC960932_0099 | EKW70478 | protein fimH | Escherichia coli 96.0932 | 809.7 | 6.40E-240 |
| FimH | 392 | ECDEC8A_5260 | EHW03149 | protein fimH | Escherichia coli DEC8A | 809.7 | 6.40E-240 |
| FimH | 393 | BX73_18995 | EYY82537 | fimbrial protein | Escherichia coli O111:NM str. 2010C-4735 | 809.7 | 6.40E-240 |
| FimH | 394 | ECEC4402_5675 | EIP27253 | type 1 fimbrial adhesin | Escherichia coli EC4402 | 809.7 | 6.40E-240 |
| FimH | 395 | BZ01_13095 | EZD50952 | fimbrial protein | Escherichia coli O111:NM str. K6897 | 809.7 | 6.40E-240 |
| FimH | 396 | ECP03048169_4826 | ENF66267 | protein fimH | Escherichia coli P0304816.9 | 809.7 | 6.40E-240 |
| FimH | 397 | BX05_07315 | EZD90141 | fimbrial protein | Escherichia coli O157:NM str. 08-4540 | 809.7 | 6.40E-240 |
| FimH | 398 | BY97_00430 | EZD28888 | fimbrial protein | Escherichia coli O111:NM str. K6723 | 809.7 | 6.40E-240 |
| FimH | 399 | ECFRIK1990_5785 | EIN35131 | type 1 fimbrial adhesin | Escherichia coli FRIK1990 | 809.7 | 6.40E-240 |
| FimH | 400 | ECOT7509_4670 | ERC51134 | protein fimH | Escherichia coli TW07509 | 809.7 | 6.40E-240 |
| FimH | 401 | A15G_01132 | ELH55222 | fimbrial protein FimH | Escherichia coli KTE203 | 809.7 | 6.40E-240 |
| FimH | 402 | ECP03048162_4816 | ENF44287 | protein fimH | Escherichia coli P0304816.2 | 809.7 | 6.40E-240 |
| FimH | 403 | ECSTECMHI813_4807 | EGW99981 | protein fimH | Escherichia coli STEC_MHI813 | 809.7 | 6.40E-240 |
| FimH | 404 | EC2867750_4984 | EMV53834 | protein fimH | Escherichia coli 2867750 | 809.7 | 6.40E-240 |
| FimH | 405 | BY54_16045 | EZB30331 | fimbrial protein | Escherichia coli O157:H7 str. F7410 | 809.7 | 6.40E-240 |
| FimH | 406 | ECH7EC508_2472 | EDU98009 | protein FimH | Escherichia coli O157:H7 str. EC508 | 809.7 | 6.40E-240 |
| FimH | 407 | G968_04306 | ESK01929 | protein fimH | Escherichia coli UMEA 3336-1 | 809.7 | 6.40E-240 |
| FimH | 408 | ECEC4013_0100 | EIP38875 | type 1 fimbrial adhesin | Escherichia coli EC4013 | 809.7 | 6.40E-240 |
| FimH | 409 | ECPA39_5724 | EIO24324 | type 1 fimbrial adhesin | Escherichia coli PA39 | 809.7 | 6.40E-240 |
| FimH | 410 | AC17_2101 | KDW99737 | protein fimH | Escherichia coli 2-210-07_S3_C2 | 809.7 | 6.40E-240 |
| FimH | 411 | BX02_26870 | EYW91329 | fimbrial protein | Escherichia coli O145:NM str. 08-4270 | 809.7 | 6.40E-240 |
| FimH | 412 | ECSTECC16502_0304 | EGW75278 | protein fimH | Escherichia coli STEC_C165-02 | 809.7 | 6.40E-240 |
| FimH | 413 | BX31_00825 | EZE90230 | fimbrial protein | Escherichia coli O121:H19 str. 2009EL1302 | 809.7 | 6.40E-240 |
| FimH | 414 | ECP030477711_4744 | ENE51901 | protein fimH | Escherichia coli P0304777.11 | 809.7 | 6.40E-240 |
| FimH | 415 | BY79_01430 | EZC48730 | fimbrial protein | Escherichia coli O157:H7 str. K4527 | 809.7 | 6.40E-240 |
| FimH | 416 | WK1_04334 | ELI74408 | fimbrial protein FimH | Escherichia coli KTE138 | 809.7 | 6.40E-240 |
| FimH | 417 | BX22_21845 | EZE61438 | fimbrial protein | Escherichia coli O157:H7 str. 2009C-4258 | 809.7 | 6.40E-240 |
| FimH | 418 | BX08_06530 | EZE07541 | fimbrial protein | Escherichia coli O103:H2 str. 2009C-3279 | 809.7 | 6.40E-240 |
| FimH | 419 | A1WA_04483 | ELG38556 | fimbrial protein FimH | Escherichia coli KTE91 | 809.7 | 6.40E-240 |
| FimH | 420 | BY47_07770 | EZA94063 | fimbrial protein | Escherichia coli O121:H19 str. F6714 | 809.7 | 6.40E-240 |
| FimH | 421 | ECMA6_0151 | EKI01286 | type 1 fimbrial adhesin | Escherichia coli MA6 | 809.7 | 6.40E-240 |
| FimH | 422 | EC174750_4760 | EMW90155 | protein fimH | Escherichia coli 174750 | 809.7 | 6.40E-240 |
| FimH | 423 | Q460_23965 | ETI71941 | fimbrial protein FimH | Escherichia coli ATCC BAA-2219 | 809.7 | 6.40E-240 |
| FimH | 424 | ECOK1357_4716 | EFZ67447 | protein fimH | Escherichia coli OK1357 | 809.7 | 6.40E-240 |
| FimH | 425 | BX07_07410 | EZD94713 | fimbrial protein | Escherichia coli O91:H14 str. 2009C-3227 | 809.7 | 6.40E-240 |
| FimH | 426 | G938_04729 | EQX81944 | protein fimH | Escherichia coli UMEA 3200-1 | 809.7 | 6.40E-240 |
| FimH | 427 | AB18_1746 | KDU19962 | protein fimH | Escherichia coli 3-267-03_S1_C1 | 809.7 | 6.40E-240 |
| FimH | 428 | EC09BKT76207_5488 | ERB68162 | protein fimH | Escherichia coli 09BKT076207 | 809.7 | 6.40E-240 |
| FimH | 429 | G797_04422 | EQR89494 | protein fimH | Escherichia coli HVH 139 (4-3192644) | 809.7 | 6.40E-240 |
| FimH | 430 | BX94_04820 | EYY14701 | fimbrial protein | Escherichia coli O121:H19 str. 2011C-3216 | 809.7 | 6.40E-240 |
| FimH | 431 | QYQ_0021 | ERC23466 | protein fimH | Escherichia coli B29-2 | 809.7 | 6.40E-240 |
| FimH | 432 | EC930624_5295 | EIH67817 | mannose-binding domain protein FimH | Escherichia coli 93.0624 | 809.7 | 6.40E-240 |
| FimH | 433 | EC1999001_4739 | EMZ73474 | protein fimH | Escherichia coli 199900.1 | 809.7 | 6.40E-240 |
| FimH | 434 | AD34_3939 | KEL59042 | protein fimH | Escherichia coli 5-172-05_S4_C3 | 809.7 | 6.40E-240 |
| FimH | 435 | BX51_00350 | EYV20059 | fimbrial protein | Escherichia coli O145:NM str. 2010C-3526 | 809.7 | 6.40E-240 |
| FimH | 436 | EC2864350_4666 | ENA89901 | protein fimH | Escherichia coli 2864350 | 809.7 | 6.40E-240 |
| FimH | 437 | BX04_24465 | EZD85348 | fimbrial protein | Escherichia coli O157:H7 str. 08-4529 | 809.7 | 6.40E-240 |
| FimH | 438 | EC2851500_4849 | EMV96314 | protein fimH | Escherichia coli 2851500 | 809.7 | 6.40E-240 |
| FimH | 439 | B230_0108 | ERC91719 | protein fimH | Escherichia coli 14A | 809.7 | 6.40E-240 |
| FimH | 440 | T22_006272 | ENO10383 | adhesin | Escherichia coli O157:H43 str. T22 | 809.7 | 6.40E-240 |
| FimH | 441 | ECP029894215_4834 | ENB55901 | protein fimH | Escherichia coli P0298942.15 | 809.7 | 6.40E-240 |
| FimH | 442 | BY02_03325 | EYY00185 | fimbrial protein | Escherichia coli O121:H19 str. 2011C-3537 | 809.7 | 6.40E-240 |
| FimH | 443 | ECO9570_13023 | EIL26781 | FimH mannose-binding protein | Escherichia coli O111:H8 str. CVM9570 | 809.7 | 6.40E-240 |
| FimH | 444 | BU54_25020 | KDV42700 | fimbrial protein | Escherichia coli O45:H2 str. 2010C-4211 | 809.7 | 6.40E-240 |
| FimH | 445 | BY05_24165 | EYX72817 | fimbrial protein | Escherichia coli O111:NM str. 2011C-3632 | 809.7 | 6.40E-240 |
| FimH | 446 | BX45_00360 | EYV45998 | fimbrial protein | Escherichia coli O145:NM str. 2010C-3510 | 809.7 | 6.40E-240 |
| FimH | 447 | ECP03047993_4952 | END98335 | protein fimH | Escherichia coli P0304799.3 | 809.7 | 6.40E-240 |
| FimH | 448 | BX82_16210 | EYY51281 | fimbrial protein | Escherichia coli O121:H19 str. 2010C-4966 | 809.7 | 6.40E-240 |
| FimH | 449 | WGU_00020 | ELJ87470 | fimbrial protein FimH | Escherichia coli KTE90 | 809.7 | 6.40E-240 |
| FimH | 450 | BY67_04725 | EZB80117 | fimbrial protein | Escherichia coli O157:H7 str. K1927 | 809.7 | 6.40E-240 |
| FimH | 451 | ECEC4439_5641 | EIP35311 | type 1 fimbrial adhesin | Escherichia coli EC4439 | 809.7 | 6.40E-240 |
| FimH | 452 | ECDEC4C_5598 | EHV00183 | minor fimbrial subunit, D-mannose specific adhesin | Escherichia coli DEC4C | 809.7 | 6.40E-240 |
| FimH | 453 | ECO9634_03668 | EJE60404 | FimH mannose-binding protein | Escherichia coli O111:H8 str. CVM9634 | 809.7 | 6.40E-240 |
| FimH | 454 | ECP030477712_4793 | ENE59546 | protein fimH | Escherichia coli P0304777.12 | 809.7 | 6.40E-240 |
| FimH | 455 | A13W_03656 | ELC92568 | protein fimH | Escherichia coli KTE193 | 809.7 | 6.40E-240 |
| FimH | 456 | ECH7EC869_5308 | EDU93133 | protein FimH | Escherichia coli O157:H7 str. EC869 | 809.7 | 6.40E-240 |
| FimH | 457 | ECEC1846_5618 | EKI64798 | type 1 fimbrial adhesin | Escherichia coli EC1846 | 809.7 | 6.40E-240 |
| FimH | 458 | ECPA35_0089 | ELW36471 | protein fimH | Escherichia coli PA35 | 809.7 | 6.40E-240 |
| FimH | 459 | ECDEC5A_5156 | EHV18521 | minor fimbrial subunit, D-mannose specific adhesin | Escherichia coli DEC5A | 809.7 | 6.40E-240 |
| FimH | 460 | ECP03048168_4802 | ENF64187 | protein fimH | Escherichia coli P0304816.8 | 809.7 | 6.40E-240 |
| FimH | 461 | ECDEC9C_5192 | EHW39056 | minor fimbrial subunit, D-mannose specific adhesin | Escherichia coli DEC9C | 809.7 | 6.40E-240 |
| FimH | 462 | BX21_01320 | EZQ34934 | fimbrial protein | Escherichia coli O111:H8 str. 2009C-4126 | 809.7 | 6.40E-240 |
| FimH | 463 | ECSTEC94C_5159 | EGW77900 | protein fimH | Escherichia coli STEC_94C | 809.7 | 6.40E-240 |
| FimH | 464 | BX66_13230 | EYZ24236 | fimbrial protein | Escherichia coli O103:H25 str. 2010C-4529 | 809.7 | 6.40E-240 |
| FimH | 465 | BW74_09320 | EZA29644 | fimbrial protein | Escherichia coli O45:H2 str. 01-3147 | 809.7 | 6.40E-240 |
| FimH | 466 | BU53_15885 | KDV46148 | fimbrial protein | Escherichia coli O91:H21 str. 2009C-3740 | 809.7 | 6.40E-240 |
| FimH | 467 | ECTW06591_5275 | EIO46332 | type 1 fimbrial adhesin | Escherichia coli TW06591 | 809.7 | 6.40E-240 |
| FimH | 468 | BX59_25515 | EYU88411 | fimbrial protein | Escherichia coli O111:NM str. 2010C-4086 | 809.7 | 6.40E-240 |
| FimH | 469 | ECO9602_04388 | EJE61133 | FimH mannose-binding protein | Escherichia coli O111:H8 str. CVM9602 | 809.7 | 6.40E-240 |
| FimH | 470 | QYI_5239 | ERC30082 | protein fimH | Escherichia coli B7-2 | 809.7 | 6.40E-240 |
| FimH | 471 | ECP029894212_4870 | ENB52401 | protein fimH | Escherichia coli P0298942.12 | 809.7 | 6.40E-240 |
| FimH | 472 | BX39_15630 | EZQ25075 | fimbrial protein | Escherichia coli O111:NM str. 2010C-3053 | 809.7 | 6.40E-240 |
| FimH | 473 | ECEC1849_5559 | EKI82696 | type 1 fimbrial adhesin | Escherichia coli EC1849 | 809.7 | 6.40E-240 |
| FimH | 474 | ECP030481613_4836 | ENF35153 | protein fimH | Escherichia coli P0304816.13 | 809.7 | 6.40E-240 |
| FimH | 475 | BX97_01635 | EYY03345 | fimbrial protein | Escherichia coli O111:NM str. 2011C-3362 | 809.7 | 6.40E-240 |
| FimH | 476 | EC40967_5585 | EII33172 | mannose-binding domain protein FimH | Escherichia coli 4.0967 | 809.7 | 6.40E-240 |
| FimH | 477 | ECEC4448_5653 | EIP49951 | type 1 fimbrial adhesin | Escherichia coli EC4448 | 809.7 | 6.40E-240 |
| FimH | 478 | ECFRIK2001_0283 | EKH63188 | type 1 fimbrial adhesin | Escherichia coli FRIK2001 | 809.7 | 6.40E-240 |
| FimH | 479 | BY66_06840 | EZB87929 | fimbrial protein | Escherichia coli O157:H7 str. K1921 | 809.7 | 6.40E-240 |
| FimH | 480 | BX48_09410 | EYV22666 | fimbrial protein | Escherichia coli O145:NM str. 2010C-3517 | 809.7 | 6.40E-240 |
| FimH | 481 | ECTW10119_0290 | EIO86741 | type 1 fimbrial adhesin | Escherichia coli TW10119 | 809.7 | 6.40E-240 |
| FimH | 482 | ECEC1845_5602 | EIP72274 | type 1 fimbrial adhesin | Escherichia coli EC1845 | 809.7 | 6.40E-240 |
| FimH | 483 | EC970246_4011 | EIG92892 | mannose-binding domain protein FimH | Escherichia coli 97.0246 | 809.7 | 6.40E-240 |
| FimH | 484 | AB20_4320 | KDT80121 | protein fimH | Escherichia coli 3-475-03_S1_C1 | 809.7 | 6.40E-240 |
| FimH | 485 | EC50959_5369 | EII10032 | mannose-binding domain protein FimH | Escherichia coli 5.0959 | 809.7 | 6.40E-240 |
| FimH | 486 | BX71_18140 | EYY97815 | fimbrial protein | Escherichia coli O111:NM str. 2010C-4715 | 809.7 | 6.40E-240 |
| FimH | 487 | ECEC4196_5743 | EIO92437 | type 1 fimbrial adhesin | Escherichia coli EC4196 | 809.7 | 6.40E-240 |
| FimH | 488 | G847_04323 | EQT88557 | protein fimH | Escherichia coli HVH 195 (3-7155360) | 809.7 | 6.40E-240 |
| FimH | 489 | ECEC1868_0038 | EKJ36033 | type 1 fimbrial adhesin | Escherichia coli EC1868 | 809.7 | 6.40E-240 |
| FimH | 490 | ECO9450_23124 | EIL00506 | FimH mannose-binding protein | Escherichia coli O103:H2 str. CVM9450 | 809.7 | 6.40E-240 |
| FimH | 491 | A1SG_01146 | ELG10041 | fimbrial protein FimH | Escherichia coli KTE54 | 809.7 | 6.40E-240 |
| FimH | 492 | BX87_04740 | EYY33126 | fimbrial protein | Escherichia coli O121:H19 str. 2010EL1058 | 809.7 | 6.40E-240 |
| FimH | 493 | BX25_24880 | EYV82731 | fimbrial protein | Escherichia coli O121:H19 str. 2009C-4659 | 809.7 | 6.40E-240 |
| FimH | 494 | BW92_08220 | EYZ52240 | fimbrial protein | Escherichia coli O157:H7 str. 06-3745 | 809.7 | 6.40E-240 |
| FimH | 495 | A1S5_00747 | ELG02230 | fimbrial protein FimH | Escherichia coli KTE48 | 809.7 | 6.40E-240 |
| FimH | 496 | BX92_16400 | EYY20975 | fimbrial protein | Escherichia coli O121:H19 str. 2011C-3108 | 809.7 | 6.40E-240 |
| FimH | 497 | BW77_17755 | EZA12178 | fimbrial protein | Escherichia coli O121:H19 str. 03-3227 | 809.7 | 6.40E-240 |
| FimH | 498 | EC2848050_4905 | EMW14377 | protein fimH | Escherichia coli 2848050 | 809.7 | 6.40E-240 |
| FimH | 499 | ECPA40_0109 | EIO32417 | type 1 fimbrial adhesin | Escherichia coli PA40 | 809.7 | 6.40E-240 |
| FimH | 500 | ECJURUA1811_4876 | EMX58836 | protein fimH | Escherichia coli Jurua 18/11 | 809.7 | 6.40E-240 |
| FimH | 501 | ECMP0209801_0007 | END56538 | protein fimH | Escherichia coli MP020980.1 | 809.7 | 6.40E-240 |
| FimH | 502 | ECATCC700728_5062 | ELV63280 | protein fimH | Escherichia coli ATCC 700728 | 809.7 | 6.40E-240 |
| FimH | 503 | BX77_03440 | EYY59382 | fimbrial protein | Escherichia coli O111:NM str. 2010C-4818 | 809.7 | 6.40E-240 |
| FimH | 504 | AE25_04222 | KDG93224 | protein fimH | Escherichia coli UCI 66 | 809.7 | 6.40E-240 |
| FimH | 505 | BY77_00645 | EZC28542 | fimbrial protein | Escherichia coli O157:H7 str. K4405 | 809.7 | 6.40E-240 |
| FimH | 506 | BX58_01330 | EYU87485 | fimbrial protein | Escherichia coli O111:NM str. 2010C-3977 | 809.7 | 6.40E-240 |
| FimH | 507 | ECP02989422_4594 | ENB55930 | protein fimH | Escherichia coli P0298942.2 | 809.7 | 6.40E-240 |
| FimH | 508 | EcE22_2414 | EDV83346 | protein FimH | Escherichia coli E22 | 809.7 | 6.40E-240 |
| FimH | 509 | ECP02989423_5016 | END61544 | protein fimH | Escherichia coli P0298942.3 | 809.7 | 6.40E-240 |
| FimH | 510 | ECP03048161_2221 | EMZ97826 | protein fimH | Escherichia coli P0304816.1 | 809.7 | 6.40E-240 |
| FimH | 511 | BW78_20165 | EYZ94313 | fimbrial protein | Escherichia coli O174:H21 str. 03-3269 | 809.7 | 6.40E-240 |
| FimH | 512 | BX29_09720 | EZE68849 | fimbrial protein | Escherichia coli O45:H2 str. 2009C-4780 | 809.7 | 6.40E-240 |
| FimH | 513 | ECEC1736_5572 | EKI57104 | type 1 fimbrial adhesin | Escherichia coli EC1736 | 809.7 | 6.40E-240 |
| FimH | 514 | ECJURUA2010_4793 | EMX45220 | protein fimH | Escherichia coli Jurua 20/10 | 809.7 | 6.40E-240 |
| FimH | 515 | A15U_00346 | ELD25460 | protein fimH | Escherichia coli KTE210 | 809.7 | 6.40E-240 |
| FimH | 516 | BX43_26730 | EZE91456 | fimbrial protein | Escherichia coli O145:NM str. 2010C-3508 | 809.7 | 6.40E-240 |
| FimH | 517 | BX10_01865 | EZE16036 | fimbrial protein | Escherichia coli O121:H7 str. 2009C-3299 | 809.7 | 6.40E-240 |
| FimH | 518 | EC32608_5496 | EIH54067 | mannose-binding domain protein FimH | Escherichia coli 3.2608 | 809.7 | 6.40E-240 |
| FimH | 519 | S1E_5461 | ERC40362 | protein fimH | Escherichia coli B94 | 809.7 | 6.40E-240 |
| FimH | 520 | EC5905_0093 | EKI02753 | type 1 fimbrial adhesin | Escherichia coli 5905 | 809.7 | 6.40E-240 |
| FimH | 521 | ECDEC9D_5276 | EHW46820 | minor fimbrial subunit, D-mannose specific adhesin | Escherichia coli DEC9D | 809.7 | 6.40E-240 |
| FimH | 522 | EC991805_5009 | ELV61609 | protein fimH | Escherichia coli 99.1805 | 809.7 | 6.40E-240 |
| FimH | 523 | BX44_01800 | EYV38384 | fimbrial protein | Escherichia coli O145:NM str. 2010C-3509 | 809.7 | 6.40E-240 |
| FimH | 524 | BX19_15110 | EZE42773 | fimbrial protein | Escherichia coli O121:H19 str. 2009C-4050 | 809.7 | 6.40E-240 |
| FimH | 525 | ECP02994831_0105 | END74264 | protein fimH | Escherichia coli P0299483.1 | 809.7 | 6.40E-240 |
| FimH | 526 | G776_04662 | EQQ96817 | protein fimH | Escherichia coli HVH 115 (4-4465997) | 809.7 | 6.40E-240 |
| FimH | 527 | BZ03_00020 | EZD63929 | fimbrial protein | Escherichia coli O111:NM str. K6904 | 809.7 | 6.40E-240 |
| FimH | 528 | ECDEC13D_4980 | EHX56685 | minor fimbrial subunit, D-mannose specific adhesin | Escherichia coli DEC13D | 809.7 | 6.40E-240 |
| FimH | 529 | EC990672_5682 | EKW76240 | protein fimH | Escherichia coli 99.0672 | 809.7 | 6.40E-240 |
| FimH | 530 | EC253486_0096 | EGW78214 | protein fimH | Escherichia coli 2534-86 | 809.7 | 6.40E-240 |
| FimH | 531 | S1C_5448 | ERC33955 | protein fimH | Escherichia coli B93 | 809.7 | 6.40E-240 |
| FimH | 532 | ECH7EC4113_5498 | EDU54322 | protein FimH | Escherichia coli O157:H7 str. EC4113 | 809.7 | 6.40E-240 |
| FimH | 533 | EC900039_5467 | EKV91502 | protein fimH | Escherichia coli 90.0039 | 809.7 | 6.40E-240 |
| FimH | 534 | C202_21586 | EMD02565 | adhesin | Escherichia coli O08 | 809.7 | 6.40E-240 |
| FimH | 535 | ECSTECB2F1_4719 | EGW64666 | protein fimH | Escherichia coli STEC_B2F1 | 809.7 | 6.40E-240 |
| FimH | 536 | BY94_01425 | EZD15739 | fimbrial protein | Escherichia coli O157:H7 str. K6676 | 809.7 | 6.40E-240 |
| FimH | 537 | BW96_01420 | EYZ36199 | fimbrial protein | Escherichia coli O157:H7 str. 07-3391 | 809.7 | 6.40E-240 |
| FimH | 538 | AE16_04226 | KDG81436 | protein fimH | Escherichia coli UCI 57 | 809.7 | 6.40E-240 |
| FimH | 539 | EC40522_5701 | EIH80077 | mannose-binding domain protein FimH | Escherichia coli 4.0522 | 809.7 | 6.40E-240 |
| FimH | 540 | ECEC4436_5595 | EIP40241 | type 1 fimbrial adhesin | Escherichia coli EC4436 | 809.7 | 6.40E-240 |
| FimH | 541 | EC960497_4786 | EIH31860 | mannose-binding domain protein FimH | Escherichia coli 96.0497 | 809.7 | 6.40E-240 |
| FimH | 542 | BZ00_24230 | EZD40541 | fimbrial protein | Escherichia coli O111:NM str. K6895 | 809.7 | 6.40E-240 |
| FimH | 543 | AB33_3668 | KDY24734 | protein fimH | Escherichia coli 2-427-07_S1_C2 | 809.7 | 6.40E-240 |
| FimH | 544 | BW76_12920 | EZA07319 | fimbrial protein | Escherichia coli O28ac:NM str. 02-3404 | 809.7 | 6.40E-240 |
| FimH | 545 | ECDEC10E_5191 | EHW84106 | minor fimbrial subunit, D-mannose specific adhesin | Escherichia coli DEC10E | 809.7 | 6.40E-240 |
| FimH | 546 | ECEC1864_0036 | EKJ19846 | type 1 fimbrial adhesin | Escherichia coli EC1864 | 809.7 | 6.40E-240 |
| FimH | 547 | ECDEC4B_5659 | EHU89528 | minor fimbrial subunit, D-mannose specific adhesin | Escherichia coli DEC4B | 809.7 | 6.40E-240 |
| FimH | 548 | BX86_16290 | EYY39724 | fimbrial protein | Escherichia coli O153:H2 str. 2010C-5034 | 809.7 | 6.40E-240 |
| FimH | 549 | ECT128201_5105 | ERE14349 | protein fimH | Escherichia coli T1282_01 | 809.7 | 6.40E-240 |
| FimH | 550 | ECP03048163_4909 | ENH27338 | protein fimH | Escherichia coli P0304816.3 | 809.7 | 6.40E-240 |
| FimH | 551 | ECEC1863_5359 | EIP72947 | type 1 fimbrial adhesin | Escherichia coli EC1863 | 809.7 | 6.40E-240 |
| FimH | 552 | EC2862600_4818 | ENA88886 | protein fimH | Escherichia coli 2862600 | 809.7 | 6.40E-240 |
| FimH | 553 | AB90_4021 | KDY30870 | protein fimH | Escherichia coli 2-427-07_S3_C1 | 809.7 | 6.40E-240 |
| FimH | 554 | BX35_01300 | EZE89467 | fimbrial protein | Escherichia coli O157:H7 str. 2009EL1913 | 809.7 | 6.40E-240 |
| FimH | 555 | ESRG_01607 | EHN87309 | protein fimH | Escherichia coli TA124 | 809.7 | 6.40E-240 |
| FimH | 556 | BGK51_21135 | ODQ12213 | fimbrial protein | Shigella sp. FC569 | 809.7 | 6.40E-240 |
| FimH | 557 | AB64_2979 | KDY91238 | protein fimH | Escherichia coli 2-427-07_S1_C3 | 809.7 | 6.40E-240 |
| FimH | 558 | EC179550_4674 | ENA57800 | protein fimH | Escherichia coli 179550 | 809.7 | 6.40E-240 |
| FimH | 559 | ECK71_06998 | KRR59676 | adhesin | Escherichia coli K71 | 809.7 | 6.40E-240 |
| FimH | 560 | ECMP0209802_0281 | EMX57015 | protein fimH | Escherichia coli MP020980.2 | 809.7 | 6.40E-240 |
| FimH | 561 | BZ02_03760 | EZD57785 | fimbrial protein | Escherichia coli O111:NM str. K6898 | 809.7 | 6.40E-240 |
| FimH | 562 | EC100833_0104 | EKK67962 | type 1 fimbrial adhesin | Escherichia coli 10.0833 | 809.7 | 6.40E-240 |
| FimH | 563 | BX93_15750 | EYY14446 | fimbrial protein | Escherichia coli O111:NM str. 2011C-3170 | 809.7 | 6.40E-240 |
| FimH | 564 | BX70_00925 | EYY91228 | fimbrial protein | Escherichia coli O111:NM str. 2010C-4622 | 809.7 | 6.40E-240 |
| FimH | 565 | L668_08230 | ERA59261 | fimbrial protein FimH | Escherichia coli 95NR1 | 809.7 | 6.40E-240 |
| FimH | 566 | QYU_0036 | ERC32327 | protein fimH | Escherichia coli B36-2 | 809.7 | 6.40E-240 |
| FimH | 567 | ECSTECH18_5459 | EGX01124 | protein fimH | Escherichia coli STEC_H.1.8 | 809.7 | 6.40E-240 |
| FimH | 568 | ECDEC4E_5303 | EHV06163 | minor fimbrial subunit, D-mannose specific adhesin | Escherichia coli DEC4E | 809.7 | 6.40E-240 |
| FimH | 569 | ECP029894214_4795 | ENB49828 | protein fimH | Escherichia coli P0298942.14 | 809.7 | 6.40E-240 |
| FimH | 570 | EC2866750_4940 | EMV69660 | protein fimH | Escherichia coli 2866750 | 809.7 | 6.40E-240 |
| FimH | 571 | AE34_00426 | KDF73177 | protein fimH | Escherichia coli BIDMC 59 | 809.7 | 6.40E-240 |
| FimH | 572 | ECJB195_5468 | EIH87840 | mannose-binding domain protein FimH | Escherichia coli JB1-95 | 809.7 | 6.40E-240 |
| FimH | 573 | ECEC1866_5509 | EKJ20886 | type 1 fimbrial adhesin | Escherichia coli EC1866 | 809.7 | 6.40E-240 |
| FimH | 574 | SSMOSELEY_0093 | EJL19845 | minor fimbrial subunit, D-mannose specific adhesin | Shigella sonnei str. Moseley | 809.7 | 6.40E-240 |
| FimH | 575 | ECEC1848_0102 | EKI91893 | type 1 fimbrial adhesin | Escherichia coli EC1848 | 809.7 | 6.40E-240 |
| FimH | 576 | QYM_0038 | ERC07703 | protein fimH | Escherichia coli B28-2 | 809.7 | 6.40E-240 |
| FimH | 577 | CFSAN001632_03250 | EKU03261 | adhesin | Escherichia coli O111:H8 str. CFSAN001632 | 809.7 | 6.40E-240 |
| FimH | 578 | EC2872800_4875 | EMV42146 | protein fimH | Escherichia coli 2872800 | 809.7 | 6.40E-240 |
| FimH | 579 | EC178200_4303 | ENG78413 | protein fimH | Escherichia coli 178200 | 809.7 | 6.40E-240 |
| FimH | 580 | A1UQ_00301 | ELE66680 | protein fimH | Escherichia coli KTE77 | 809.7 | 6.40E-240 |
| FimH | 581 | EC174900_4760 | EMZ59504 | protein fimH | Escherichia coli 174900 | 809.7 | 6.40E-240 |
| FimH | 582 | ECDEC13B_4652 | EHX55110 | minor fimbrial subunit, D-mannose specific adhesin | Escherichia coli DEC13B | 809.7 | 6.40E-240 |
| FimH | 583 | ECCG_02905 | EFE60478 | mannose-specific adhesin FimH | Escherichia coli B088 | 809.7 | 6.40E-240 |
| FimH | 584 | DC23_14425 | KDM77237 | fimbrial protein | Escherichia coli O145:H28 str. 4865/96 | 809.7 | 6.40E-240 |
| FimH | 585 | BX84_16420 | EYY37880 | fimbrial protein | Escherichia coli O121:H19 str. 2010C-4989 | 809.7 | 6.40E-240 |
| FimH | 586 | BZ05_01435 | EZD73641 | fimbrial protein | Escherichia coli O111:NM str. K6915 | 809.7 | 6.40E-240 |
| FimH | 587 | ECP02994387_4956 | ENC09268 | protein fimH | Escherichia coli P0299438.7 | 809.7 | 6.40E-240 |
| FimH | 588 | BX52_15310 | EYV08542 | fimbrial protein | Escherichia coli O121:H19 str. 2010C-3609 | 809.7 | 6.40E-240 |
| FimH | 589 | ECP030481610_4883 | ENF19852 | protein fimH | Escherichia coli P0304816.10 | 809.7 | 6.40E-240 |
| FimH | 590 | ECBCE001MS16_4696 | EMX83918 | protein fimH | Escherichia coli BCE001_MS16 | 809.7 | 6.40E-240 |
| FimH | 591 | ECP030481612_4838 | ENF26950 | protein fimH | Escherichia coli P0304816.12 | 809.7 | 6.40E-240 |
| FimH | 592 | BY37_09455 | EYW07451 | fimbrial protein | Escherichia coli O157:H7 str. 2011EL-2312 | 809.7 | 6.40E-240 |
| FimH | 593 | BW83_02310 | EYZ86851 | fimbrial protein | Escherichia coli O121:H19 str. 06-3003 | 809.7 | 6.40E-240 |
| FimH | 594 | ECP030481611_4783 | ENF16184 | protein fimH | Escherichia coli P0304816.11 | 809.7 | 6.40E-240 |
| FimH | 595 | ECO111_5176 | BAI38893 | minor component FimH of type 1 fimbriae | Escherichia coli O111:H- str. 11128 | 809.7 | 6.40E-240 |
| FimH | 596 | ECP02989428_4670 | ENB70674 | protein fimH | Escherichia coli P0298942.8 | 809.7 | 6.40E-240 |
| FimH | 597 | ECDEC9A_5641 | EHW28004 | minor fimbrial subunit, D-mannose specific adhesin | Escherichia coli DEC9A | 809.7 | 6.40E-240 |
| FimH | 598 | BU57_33875 | KDV49380 | fimbrial protein | Escherichia coli O121:H19 str. 2011C-3609 | 809.7 | 6.40E-240 |
| FimH | 599 | ECO5905_15048 | EFX28096 | adhesin | Escherichia coli O55:H7 str. USDA 5905 | 809.7 | 6.40E-240 |
| FimH | 600 | S1M_0023 | ERE39718 | protein fimH | Escherichia coli B90 | 809.7 | 6.40E-240 |
| FimH | 601 | ECDG_02704 | EFF03501 | minor fimbrial subunit | Escherichia coli B185 | 809.7 | 6.40E-240 |
| FimH | 602 | ECEC1870_5611 | EKJ36521 | type 1 fimbrial adhesin | Escherichia coli EC1870 | 809.7 | 6.40E-240 |
| FimH | 603 | ECP03018674_4984 | ENA27980 | protein fimH | Escherichia coli P0301867.4 | 809.7 | 6.40E-240 |
| FimH | 604 | EC2735000_4834 | EMZ62461 | protein fimH | Escherichia coli 2735000 | 809.7 | 6.40E-240 |
| FimH | 605 | EC990839_5081 | ELV31211 | protein fimH | Escherichia coli 99.0839 | 809.7 | 6.40E-240 |
| FimH | 606 | AB04_3850 | KDZ93245 | protein fimH | Escherichia coli 2-427-07_S1_C1 | 809.7 | 6.40E-240 |
| FimH | 607 | ECT23400_5284 | ERC74208 | protein fimH | Escherichia coli T234_00 | 809.7 | 6.40E-240 |
| FimH | 608 | EC2788150_4866 | EMW37572 | protein fimH | Escherichia coli 2788150 | 809.7 | 6.40E-240 |
| FimH | 609 | EC900091_0103 | EKW05793 | protein fimH | Escherichia coli 90.0091 | 809.7 | 6.40E-240 |
| FimH | 610 | BZ04_07795 | EZD60323 | fimbrial protein | Escherichia coli O111:NM str. K6908 | 809.7 | 6.40E-240 |
| FimH | 611 | BX65_19270 | EYZ12833 | fimbrial protein | Escherichia coli O103:H2 str. 2010C-4433 | 809.7 | 6.40E-240 |
| FimH | 612 | A1YU_04050 | ELF07189 | protein fimH | Escherichia coli KTE142 | 809.7 | 6.40E-240 |
| FimH | 613 | ECMP0209401_0057 | EMX61206 | protein fimH | Escherichia coli MP020940.1 | 809.7 | 6.40E-240 |
| FimH | 614 | AB40_4853 | KDA65782 | protein fimH | Escherichia coli 1-182-04_S1_C2 | 809.7 | 6.40E-240 |
| FimH | 615 | BY98_04305 | EZD31498 | fimbrial protein | Escherichia coli O111:NM str. K6728 | 809.7 | 6.40E-240 |
| FimH | 616 | G960_04698 | ERB28442 | protein fimH | Escherichia coli UMEA 3292-1 | 809.7 | 6.40E-240 |
| FimH | 617 | BX42_05805 | EYV57486 | fimbrial protein | Escherichia coli O145:NM str. 2010C-3507 | 809.7 | 6.40E-240 |
| FimH | 618 | G902_04702 | EQW25434 | protein fimH | Escherichia coli UMEA 3052-1 | 809.7 | 6.40E-240 |
| FimH | 619 | EC2866550_4965 | EMV66036 | protein fimH | Escherichia coli 2866550 | 809.7 | 6.40E-240 |
| FimH | 620 | BW80_21875 | EZA00592 | fimbrial protein | Escherichia coli O111:NM str. 03-3484 | 809.7 | 6.40E-240 |
| FimH | 621 | A311_00415 | ELG92204 | fimbrial protein FimH | Escherichia coli KTE146 | 809.7 | 6.40E-240 |
| FimH | 622 | ECT184097_5002 | ERC65844 | protein fimH | Escherichia coli T1840_97 | 809.7 | 6.40E-240 |
| FimH | 623 | ECP03047778_4627 | ENF04711 | protein fimH | Escherichia coli P0304777.8 | 809.7 | 6.40E-240 |
| FimH | 624 | AB26_4849 | EZK15596 | protein fimH | Escherichia coli 2-011-08_S1_C2 | 809.7 | 6.40E-240 |
| FimH | 625 | EC180600_4732 | EMW73185 | protein fimH | Escherichia coli 180600 | 809.7 | 6.40E-240 |
| FimH | 626 | AC48_3484 | KDY30417 | protein fimH | Escherichia coli 2-427-07_S3_C3 | 809.7 | 6.40E-240 |
| FimH | 627 | ECDEC11D_5089 | EHX04070 | protein fimH | Escherichia coli DEC11D | 809.7 | 6.40E-240 |
| FimH | 628 | BU64_06045 | KDV62129 | fimbrial protein | Escherichia coli O128:H2 str. 2011C-3317 | 809.7 | 6.40E-240 |
| FimH | 629 | ECO9340_01835 | EIL09194 | FimH mannose-binding protein | Escherichia coli O103:H25 str. CVM9340 | 809.7 | 6.40E-240 |
| FimH | 630 | ECEC1850_0052 | EKJ05482 | type 1 fimbrial adhesin | Escherichia coli EC1850 | 809.7 | 6.40E-240 |
| FimH | 631 | BX81_02415 | EYY46072 | fimbrial protein | Escherichia coli O165:H25 str. 2010C-4874 | 809.7 | 6.40E-240 |
| FimH | 632 | KO11_23225 | AFH19601 | adhesin | Escherichia coli KO11 | 809.7 | 6.40E-240 |
| FimH | 633 | BY32_04020 | EYW27200 | fimbrial protein | Escherichia coli O157:H7 str. 2011EL-2286 | 809.7 | 6.40E-240 |
| FimH | 634 | G861_04239 | EQU61552 | protein fimH | Escherichia coli HVH 209 (4-3062651) | 809.7 | 6.40E-240 |
| FimH | 635 | ECDEC11B_5032 | EHW98407 | minor fimbrial subunit, D-mannose specific adhesin | Escherichia coli DEC11B | 809.7 | 6.40E-240 |
| FimH | 636 | ECPA2_0036 | ELV98826 | protein fimH | Escherichia coli PA2 | 809.7 | 6.40E-240 |
| FimH | 637 | EC2865200_4927 | EMV84898 | protein fimH | Escherichia coli 2865200 | 809.7 | 6.40E-240 |
| FimH | 638 | ECH7EC4196_1019 | EDU34053 | protein FimH | Escherichia coli O157:H7 str. EC4196 | 809.7 | 6.40E-240 |
| FimH | 639 | EC2780750_0007 | EMW53736 | protein fimH | Escherichia coli 2780750 | 809.7 | 6.40E-240 |
| FimH | 640 | L341_0460 | EST02275 | mannose-specific adhesin FimH | Escherichia coli CE418 | 809.7 | 6.40E-240 |
| FimH | 641 | BY38_12440 | EZF05463 | fimbrial protein | Escherichia coli O157:H7 str. 2011EL-2313 | 809.7 | 6.40E-240 |
| FimH | 642 | SS53G_3490 | EFZ51907 | protein fimH | Shigella sonnei 53G | 809.7 | 6.40E-240 |
| FimH | 643 | ECBD561099_5220 | ERC62116 | protein fimH | Escherichia coli Bd5610_99 | 809.7 | 6.40E-240 |
| FimH | 644 | AD12_0008 | EZJ31874 | protein fimH | Escherichia coli 1-392-07_S4_C2 | 809.7 | 6.40E-240 |
| FimH | 645 | EC990814_5088 | ELV14506 | protein fimH | Escherichia coli 99.0814 | 809.7 | 6.40E-240 |
| FimH | 646 | BX91_04165 | EYY27547 | fimbrial protein | Escherichia coli O121:H19 str. 2011C-3072 | 809.7 | 6.40E-240 |
| FimH | 647 | ECP03047779_4738 | ENF07502 | protein fimH | Escherichia coli P0304777.9 | 809.7 | 6.40E-240 |
| FimH | 648 | BX09_24175 | EZD97569 | fimbrial protein | Escherichia coli O145:H28 str. 2009C-3292 | 809.7 | 6.40E-240 |
| FimH | 649 | AB76_1908 | KDT62504 | protein fimH | Escherichia coli 3-267-03_S1_C3 | 809.7 | 6.40E-240 |
| FimH | 650 | ECDEC13A_0092 | EHX52176 | minor fimbrial subunit, D-mannose specific adhesin | Escherichia coli DEC13A | 809.7 | 6.40E-240 |
| FimH | 651 | EC5412_5623 | EKI06083 | type 1 fimbrial adhesin | Escherichia coli 5412 | 809.7 | 6.40E-240 |
| FimH | 652 | ECO103_5101 | BAI33780 | minor component FimH of type 1 fimbriae | Escherichia coli O103:H2 str. 12009 | 809.7 | 6.40E-240 |
| FimH | 653 | ECDEC4D_5340 | EHU99600 | minor fimbrial subunit, D-mannose specific adhesin | Escherichia coli DEC4D | 809.7 | 6.40E-240 |
| FimH | 654 | ECEC1734_0037 | EIP71383 | type 1 fimbrial adhesin | Escherichia coli EC1734 | 809.7 | 6.40E-240 |
| FimH | 655 | ECEC1737_5624 | EKI59045 | type 1 fimbrial adhesin | Escherichia coli EC1737 | 809.7 | 6.40E-240 |
| FimH | 656 | BX47_26910 | EYV24894 | fimbrial protein | Escherichia coli O145:NM str. 2010C-3516 | 809.7 | 6.40E-240 |
| FimH | 657 | S3K_0037 | ERD52951 | protein fimH | Escherichia coli B114 | 809.7 | 6.40E-240 |
| FimH | 658 | BY96_06130 | EZD31898 | fimbrial protein | Escherichia coli O111:NM str. K6722 | 809.7 | 6.40E-240 |
| FimH | 659 | G777_00488 | EQR06844 | protein fimH | Escherichia coli HVH 115 (4-4465989) | 809.7 | 6.40E-240 |
| FimH | 660 | WAY_04401 | EOU27307 | fimbrial protein FimH | Escherichia coli KTE13 | 809.7 | 6.40E-240 |
| FimH | 661 | ECH74042_A0948 | EDZ87200 | protein FimH | Escherichia coli O157:H7 str. EC4042 | 809.7 | 6.40E-240 |
| FimH | 662 | ECEC96038_5574 | EKI03008 | type 1 fimbrial adhesin | Escherichia coli EC96038 | 809.7 | 6.40E-240 |
| FimH | 663 | BY99_01390 | EZD37230 | fimbrial protein | Escherichia coli O111:NM str. K6890 | 809.7 | 6.40E-240 |
| FimH | 664 | BU55_12190 | KDV39764 | fimbrial protein | Escherichia coli O146:H21 str. 2010C-3325 | 809.7 | 6.40E-240 |
| FimH | 665 | ECPA48_4937 | ELV93061 | protein fimH | Escherichia coli PA48 | 809.7 | 6.40E-240 |
| FimH | 666 | ECP02994832_0109 | END84228 | protein fimH | Escherichia coli P0299483.2 | 809.7 | 6.40E-240 |
| FimH | 667 | BX53_00445 | EZE99945 | fimbrial protein | Escherichia coli O121:H19 str. 2010C-3794 | 809.7 | 6.40E-240 |
| FimH | 668 | B232_0156 | ERE42136 | protein fimH | Escherichia coli Tx1686 | 809.7 | 6.40E-240 |
| FimH | 669 | BX49_03980 | EYV18148 | fimbrial protein | Escherichia coli O145:NM str. 2010C-3518 | 809.7 | 6.40E-240 |
| FimH | 670 | BX14_11905 | EZE22330 | fimbrial protein | Escherichia coli O45:H2 str. 2009C-3686 | 809.7 | 6.40E-240 |
| FimH | 671 | BX79_06225 | EYY63245 | fimbrial protein | Escherichia coli O121:H19 str. 2010C-4824 | 809.7 | 6.40E-240 |
| FimH | 672 | BN16_03841 | CCK49700 | minor fimbrial subunit, D-mannose specific adhesin | Escherichia coli chi7122 | 809.7 | 6.40E-240 |
| FimH | 673 | SS52_5686 | AJA29457 | mannose-specific adhesin FimH | Escherichia coli O157:H7 str. SS52 | 809.7 | 6.40E-240 |
| FimH | 674 | ECPA24_5483 | EIN88108 | type 1 fimbrial adhesin | Escherichia coli PA24 | 809.7 | 6.40E-240 |
| FimH | 675 | ECPA25_5452 | EIN89936 | type 1 fimbrial adhesin | Escherichia coli PA25 | 809.7 | 6.40E-240 |
| FimH | 676 | AE17_03600 | KDG84299 | protein fimH | Escherichia coli UCI 58 | 809.7 | 6.40E-240 |
| FimH | 677 | ECSTECDG1313_0208 | EGW98767 | protein fimH | Escherichia coli STEC_DG131-3 | 809.7 | 6.40E-240 |
| FimH | 678 | EC2845350_4893 | EMW26474 | protein fimH | Escherichia coli 2845350 | 809.7 | 6.40E-240 |
| FimH | 679 | EC960939_5686 | EKW43928 | protein fimH | Escherichia coli 96.0939 | 809.7 | 6.40E-240 |
| FimH | 680 | ECDEC9B_5185 | EHW33330 | minor fimbrial subunit, D-mannose specific adhesin | Escherichia coli DEC9B | 809.7 | 6.40E-240 |
| FimH | 681 | A1YO_00359 | ELG73929 | fimbrial protein FimH | Escherichia coli KTE136 | 809.7 | 6.40E-240 |
| FimH | 682 | BX37_23555 | EZQ22840 | fimbrial protein | Escherichia coli O111:H8 str. 2009EL-2169 | 809.7 | 6.40E-240 |
| FimH | 683 | ECEC4203_5692 | EIO87455 | type 1 fimbrial adhesin | Escherichia coli EC4203 | 809.7 | 6.40E-240 |
| FimH | 684 | ECDEC13E_4953 | EHX67565 | minor fimbrial subunit, D-mannose specific adhesin | Escherichia coli DEC13E | 809.7 | 6.40E-240 |
| FimH | 685 | ECoL_02136 | EFW75157 | mannose-specific adhesin FimH | Escherichia coli EC4100B | 809.7 | 6.40E-240 |
| FimH | 686 | BX46_11050 | EYV44995 | fimbrial protein | Escherichia coli O145:NM str. 2010C-3511 | 809.7 | 6.40E-240 |
| FimH | 687 | ECNE1487_0155 | EKH53702 | type 1 fimbrial adhesin | Escherichia coli NE1487 | 809.7 | 6.40E-240 |
| FimH | 688 | ECPA3_5726 | EIN49135 | type 1 fimbrial adhesin | Escherichia coli PA3 | 809.7 | 6.40E-240 |
| FimH | 689 | L342_2264 | ESS94093 | mannose-specific adhesin FimH | Escherichia coli CE516 | 809.7 | 6.40E-240 |
| FimH | 690 | BY35_00460 | EYW13520 | fimbrial protein | Escherichia coli O157:H7 str. 2011EL-2289 | 809.7 | 6.40E-240 |
| FimH | 691 | BY33_00460 | EYW24529 | fimbrial protein | Escherichia coli O157:H7 str. 2011EL-2287 | 809.7 | 6.40E-240 |
| FimH | 692 | EC12264_5388 | EIH21423 | mannose-binding domain protein FimH | Escherichia coli 1.2264 | 809.7 | 6.40E-240 |
| FimH | 693 | L667_10010 | ERE04312 | fimbrial protein FimH | Escherichia coli 95JB1 | 809.7 | 6.40E-240 |
| FimH | 694 | BW87_04340 | EYZ76645 | fimbrial protein | Escherichia coli O145:NM str. 06-3484 | 809.7 | 6.40E-240 |
| FimH | 695 | BY81_11715 | EZC56800 | fimbrial protein | Escherichia coli O121:H19 str. K5269 | 809.7 | 6.40E-240 |
| FimH | 696 | ECDEC8E_5340 | EHW21331 | minor fimbrial subunit, D-mannose specific adhesin | Escherichia coli DEC8E | 809.7 | 6.40E-240 |
| FimH | 697 | ECDEC5B_0026 | EHV37794 | minor fimbrial subunit, D-mannose specific adhesin | Escherichia coli DEC5B | 809.7 | 6.40E-240 |
| FimH | 698 | AD04_4694 | KEL22176 | protein fimH | Escherichia coli 5-172-05_S4_C2 | 809.7 | 6.40E-240 |
| FimH | 699 | ECP03048166_4881 | ENF44667 | protein fimH | Escherichia coli P0304816.6 | 809.7 | 6.40E-240 |
| FimH | 700 | BX60_18765 | EYU74461 | fimbrial protein | Escherichia coli O111:NM str. 2010C-4221 | 809.7 | 6.40E-240 |
| FimH | 701 | EC990815_5065 | ELV23614 | protein fimH | Escherichia coli 99.0815 | 809.7 | 6.40E-240 |
| FimH | 702 | ECP029943811_4905 | ENB91456 | protein fimH | Escherichia coli P0299438.11 | 809.7 | 6.40E-240 |
| FimH | 703 | ERBG_01520 | EGC12416 | FimH protein | Escherichia coli E1167 | 809.7 | 6.40E-240 |
| FimH | 704 | SS322685_0182 | EIQ48973 | protein fimH | Shigella sonnei 3226-85 | 809.7 | 6.40E-240 |
| FimH | 705 | AE49_03677 | KDG17551 | protein fimH | Escherichia coli BIDMC 74 | 809.7 | 6.40E-240 |
| FimH | 706 | L403_04597 | ESM26285 | protein fimH | Escherichia coli BWH 32 | 809.7 | 6.40E-240 |
| FimH | 707 | G840_04556 | EQT56814 | protein fimH | Escherichia coli HVH 188 (4-2356988) | 809.7 | 6.40E-240 |
| FimH | 708 | ECH7EC4486_2038 | EDU82484 | protein FimH | Escherichia coli O157:H7 str. EC4486 | 809.7 | 6.40E-240 |
| FimH | 709 | EC2733950_4776 | END36112 | protein fimH | Escherichia coli 2733950 | 809.7 | 6.40E-240 |
| FimH | 710 | ECP030477710_4704 | ENE40693 | protein fimH | Escherichia coli P0304777.10 | 809.7 | 6.40E-240 |
| FimH | 711 | AB38_4602 | EYE32313 | protein fimH | Escherichia coli 1-110-08_S1_C2 | 809.7 | 6.40E-240 |
| FimH | 712 | ECDEC10F_0180 | EHX02337 | minor fimbrial subunit, D-mannose specific adhesin | Escherichia coli DEC10F | 809.7 | 6.40E-240 |
| FimH | 713 | LI75_01980 | AIT33120 | fimbrial protein | Escherichia coli FAP1 | 809.7 | 6.40E-240 |
| FimH | 714 | ECFRIK1996_5654 | EIN15661 | type 1 fimbrial adhesin | Escherichia coli FRIK1996 | 809.7 | 6.40E-240 |
| FimH | 715 | ECO5101_17266 | EFX08693 | adhesin | Escherichia coli O157:H7 str. G5101 | 809.7 | 6.40E-240 |
| FimH | 716 | EcSMS35_4847 | ACB17846 | protein FimH | Escherichia coli SMS-3-5 | 809.7 | 6.40E-240 |
| FimH | 717 | ECP029894210_4754 | ENB33504 | protein fimH | Escherichia coli P0298942.10 | 809.7 | 6.40E-240 |
| FimH | 718 | BX99_22945 | EZQ39573 | fimbrial protein | Escherichia coli O111:H8 str. 2011C-3453 | 809.7 | 6.40E-240 |
| FimH | 719 | BX20_13945 | EZE49769 | fimbrial protein | Escherichia coli O111:NM str. 2009C-4052 | 809.7 | 6.40E-240 |
| FimH | 720 | Q459_03880 | ETD59161 | fimbrial protein FimH | Escherichia coli ATCC BAA-2215 | 809.7 | 6.40E-240 |
| FimH | 721 | AB71_0120 | EZK00374 | protein fimH | Escherichia coli 1-182-04_S1_C3 | 809.7 | 6.40E-240 |
| FimH | 722 | AB85_5065 | KDV96349 | protein fimH | Escherichia coli 2-156-04_S3_C1 | 809.7 | 6.40E-240 |
| FimH | 723 | ECP03047775_4548 | ENE91965 | protein fimH | Escherichia coli P0304777.5 | 809.7 | 6.40E-240 |
| FimH | 724 | WIC_04871 | ELI18357 | fimbrial protein FimH | Escherichia coli KTE112 | 809.7 | 6.40E-240 |
| FimH | 725 | ECP02989427_4762 | ENB73113 | protein fimH | Escherichia coli P0298942.7 | 809.7 | 6.40E-240 |
| FimH | 726 | ECH7EC4045_A0392 | EDZ83056 | protein FimH | Escherichia coli O157:H7 str. EC4045 | 809.7 | 6.40E-240 |
| FimH | 727 | ECDEC14D_5083 | EHX84450 | minor fimbrial subunit, D-mannose specific adhesin | Escherichia coli DEC14D | 809.7 | 6.40E-240 |
| FimH | 728 | EC179100_4995 | END29361 | protein fimH | Escherichia coli 179100 | 809.7 | 6.40E-240 |
| FimH | 729 | A1YM_01695 | ELG65699 | fimbrial protein FimH | Escherichia coli KTE135 | 809.7 | 6.40E-240 |
| FimH | 730 | ESQG_01453 | EHN86475 | protein fimH | Escherichia coli H494 | 809.7 | 6.40E-240 |
| FimH | 731 | ECBCE034MS14_5200 | EMV15255 | protein fimH | Escherichia coli BCE034_MS-14 | 809.7 | 6.40E-240 |
| FimH | 732 | ECFRIK1999_0022 | EKH43266 | type 1 fimbrial adhesin | Escherichia coli FRIK1999 | 809.7 | 6.40E-240 |
| FimH | 733 | ECEC1862_5620 | EKJ00663 | type 1 fimbrial adhesin | Escherichia coli EC1862 | 809.7 | 6.40E-240 |
| FimH | 734 | EC2853500_4883 | EMV96225 | protein fimH | Escherichia coli 2853500 | 809.7 | 6.40E-240 |
| FimH | 735 | AF33_04555 | KDG50636 | protein fimH | Escherichia coli CHS 77 | 809.7 | 6.40E-240 |
| FimH | 736 | ECOSU61_14721 | EFX32609 | adhesin | Escherichia coli O157:H7 str. LSU-61 | 809.7 | 6.40E-240 |
| FimH | 737 | ECRM13514_5564 | AHG12180 | mannose-specific adhesin FimH | Escherichia coli O145:H28 str. RM13514 | 809.7 | 6.40E-240 |
| FimH | 738 | BW88_24035 | EYZ59759 | fimbrial protein | Escherichia coli O79:H7 str. 06-3501 | 809.7 | 6.40E-240 |
| FimH | 739 | ECP03048164_4865 | ENH27473 | protein fimH | Escherichia coli P0304816.4 | 809.7 | 6.40E-240 |
| FimH | 740 | EC971742_5134 | EKW68844 | protein fimH | Escherichia coli 97.1742 | 809.7 | 6.40E-240 |
| FimH | 741 | ECDEC11C_5357 | EHX05910 | protein fimH | Escherichia coli DEC11C | 809.7 | 6.40E-240 |
| FimH | 742 | ECDEC3F_0095 | EHU97516 | minor fimbrial subunit, D-mannose specific adhesin | Escherichia coli DEC3F | 809.7 | 6.40E-240 |
| FimH | 743 | ECPA9_0035 | EIN68667 | type 1 fimbrial adhesin | Escherichia coli PA9 | 809.7 | 6.40E-240 |
| FimH | 744 | ECIAI1_4536 | CAR01277 | minor component of type 1 fimbriae | Escherichia coli IAI1 | 809.7 | 6.40E-240 |
| FimH | 745 | S1K_0038 | ERE36133 | protein fimH | Escherichia coli B89 | 809.7 | 6.40E-240 |
| FimH | 746 | BY80_11950 | EZC53192 | fimbrial protein | Escherichia coli O121:H19 str. K5198 | 809.7 | 6.40E-240 |
| FimH | 747 | HMPREF0986_00662 | EHP67398 | protein fimH | Escherichia coli 4_1_47FAA | 809.7 | 6.40E-240 |
| FimH | 748 | A13E_01204 | ELH37523 | fimbrial protein FimH | Escherichia coli KTE184 | 809.7 | 6.40E-240 |
| FimH | 749 | APECO78_02740 | AGC85287 | adhesin | Escherichia coli APEC O78 | 809.7 | 6.40E-240 |
| FimH | 750 | ECPA13_4921 | ELV76717 | protein fimH | Escherichia coli PA13 | 809.7 | 6.40E-240 |
| FimH | 751 | EC991762_0036 | ELW27728 | protein fimH | Escherichia coli 99.1762 | 809.7 | 6.40E-240 |
| FimH | 752 | BX67_25075 | EYZ21896 | fimbrial protein | Escherichia coli O145:NM str. 2010C-4557C2 | 809.7 | 6.40E-240 |
| FimH | 753 | G742_04546 | EQP66793 | protein fimH | Escherichia coli HVH 79 (4-2512823) | 809.7 | 6.40E-240 |
| FimH | 754 | ECEC1847_5639 | EKI72727 | type 1 fimbrial adhesin | Escherichia coli EC1847 | 809.7 | 6.40E-240 |
| FimH | 755 | BW71_19760 | EZA20108 | fimbrial protein | Escherichia coli O113:H21 str. 07-4224 | 809.7 | 6.40E-240 |
| FimH | 756 | AB67_5018 | KEJ70461 | protein fimH | Escherichia coli 5-366-08_S1_C3 | 809.7 | 6.40E-240 |
| FimH | 757 | A31K_01757 | ELH09238 | fimbrial protein FimH | Escherichia coli KTE165 | 809.7 | 6.40E-240 |
| FimH | 758 | BX27_21975 | EZE76683 | fimbrial protein | Escherichia coli O121:H19 str. 2009C-4750 | 809.7 | 6.40E-240 |
| FimH | 759 | AC69_4759 | KDX45977 | protein fimH | Escherichia coli 2-177-06_S4_C1 | 809.7 | 6.40E-240 |
| FimH | 760 | EC01288_4726 | EKJ54229 | fimH | Escherichia coli 0.1288 | 809.7 | 6.40E-240 |
| FimH | 761 | BY34_23110 | EYW09887 | fimbrial protein | Escherichia coli O157:H7 str. 2011EL-2288 | 809.7 | 6.40E-240 |
| FimH | 762 | ECP02989429_4460 | ENB71701 | protein fimH | Escherichia coli P0298942.9 | 809.7 | 6.40E-240 |
| FimH | 763 | ECO9389_14688 | EFX13481 | adhesin | Escherichia coli O157:H- str. 493-89 | 809.7 | 6.40E-240 |
| FimH | 764 | BY07_10570 | EYX74606 | fimbrial protein | Escherichia coli O111:NM str. 2011C-3679 | 809.7 | 6.40E-240 |
| FimH | 765 | EC50588_4796 | EIH03433 | mannose-binding domain protein FimH | Escherichia coli 5.0588 | 809.7 | 6.40E-240 |
| FimH | 766 | ECEC1735_5669 | EKI46625 | type 1 fimbrial adhesin | Escherichia coli EC1735 | 809.7 | 6.40E-240 |
| FimH | 767 | LY180_22660 | AGW11321 | fimbrial protein FimH | Escherichia coli LY180 | 809.7 | 6.40E-240 |
| FimH | 768 | ECP03047777_4737 | ENE96913 | protein fimH | Escherichia coli P0304777.7 | 809.7 | 6.40E-240 |
| FimH | 769 | BW70_02140 | EZA37356 | fimbrial protein | Escherichia coli O174:H8 str. 04-3038 | 809.7 | 6.40E-240 |
| FimH | 770 | ECP030481615_4793 | ENF42048 | protein fimH | Escherichia coli P0304816.15 | 809.7 | 6.40E-240 |
| FimH | 771 | ECP03047773_4774 | ENE82532 | protein fimH | Escherichia coli P0304777.3 | 809.7 | 6.40E-240 |
| FimH | 772 | ECP02989424_4903 | END61092 | protein fimH | Escherichia coli P0298942.4 | 809.7 | 6.40E-240 |
| FimH | 773 | EC2720900_0042 | EMX96615 | protein fimH | Escherichia coli 2720900 | 809.7 | 6.40E-240 |
| FimH | 774 | S3G_0037 | ERD37205 | protein fimH | Escherichia coli B112 | 809.7 | 6.40E-240 |
| FimH | 775 | BY03_24995 | EYX90194 | fimbrial protein | Escherichia coli O111:NM str. 2011C-3573 | 809.7 | 6.40E-240 |
| FimH | 776 | ESTG_04081 | EIG42820 | protein fimH | Escherichia coli B799 | 809.7 | 6.40E-240 |
| FimH | 777 | CDK60037 | CDK60037 | mannose-specific adhesin FimH | Escherichia coli IS9 | 809.7 | 6.40E-240 |
| FimH | 778 | ECEC1869_0037 | EKJ47685 | type 1 fimbrial adhesin | Escherichia coli EC1869 | 809.7 | 6.40E-240 |
| FimH | 779 | QYK_0036 | ERC08333 | protein fimH | Escherichia coli B28-1 | 809.7 | 6.40E-240 |
| FimH | 780 | ECP02994383_4888 | ENB93561 | protein fimH | Escherichia coli P0299438.3 | 809.7 | 6.40E-240 |
| FimH | 781 | BY09_01290 | EYX70078 | fimbrial protein | Escherichia coli O157:H7 str. 2011EL-1107 | 809.7 | 6.40E-240 |
| FimH | 782 | ECDEC11E_4993 | EHX14666 | protein fimH | Escherichia coli DEC11E | 809.7 | 6.40E-240 |
| FimH | 783 | BX56_22465 | EYU87674 | fimbrial protein | Escherichia coli O45:H2 str. 2010C-3876 | 809.7 | 6.40E-240 |
| FimH | 784 | Q458_24225 | ETD61297 | fimbrial protein FimH | Escherichia coli ATCC BAA-2209 | 809.7 | 6.40E-240 |
| FimH | 785 | ECO2687_22284 | EFX18258 | adhesin | Escherichia coli O157:H- str. H 2687 | 809.7 | 6.40E-240 |
| FimH | 786 | AC76_0698 | KEL40648 | protein fimH | Escherichia coli 5-172-05_S4_C1 | 809.7 | 6.40E-240 |
| FimH | 787 | ECEC4437_5762 | EIP49010 | type 1 fimbrial adhesin | Escherichia coli EC4437 | 809.7 | 6.40E-240 |
| FimH | 788 | BY82_00140 | EZC62937 | fimbrial protein | Escherichia coli O157:H7 str. K5418 | 809.7 | 6.40E-240 |
| FimH | 789 | EC2756500_4980 | EMW55274 | protein fimH | Escherichia coli 2756500 | 809.7 | 6.40E-240 |
| FimH | 790 | BY45_26395 | EZA89733 | fimbrial protein | Escherichia coli O157:H7 str. F6142 | 809.7 | 6.40E-240 |
| FimH | 791 | A191_02467 | ELD67888 | protein fimH | Escherichia coli KTE233 | 809.7 | 6.40E-240 |
| FimH | 792 | ECDEC14B_5251 | EHX73038 | minor fimbrial subunit, D-mannose specific adhesin | Escherichia coli DEC14B | 809.7 | 6.40E-240 |
| FimH | 793 | BY78_01310 | EZC44001 | fimbrial protein | Escherichia coli O157:H7 str. K4406 | 809.7 | 6.40E-240 |
| FimH | 794 | ECDEC11A_5002 | EHW84764 | minor fimbrial subunit, D-mannose specific adhesin | Escherichia coli DEC11A | 809.7 | 6.40E-240 |
| FimH | 795 | BY08_12080 | EYX87038 | fimbrial protein | Escherichia coli O103:H2 str. 2011C-3750 | 809.7 | 6.40E-240 |
| FimH | 796 | BX72_10245 | EYY89859 | fimbrial protein | Escherichia coli O121:H19 str. 2010C-4732 | 809.7 | 6.40E-240 |
| FimH | 797 | HMPREF1591_03546 | ESA62906 | protein FimH | Escherichia coli 113303 | 809.7 | 6.40E-240 |
| FimH | 798 | BX03_17855 | EYW93581 | fimbrial protein | Escherichia coli O111:NM str. 08-4487 | 809.7 | 6.40E-240 |
| FimH | 799 | EC90111_5738 | EII21409 | mannose-binding domain protein FimH | Escherichia coli 9.0111 | 809.7 | 6.40E-240 |
| FimH | 800 | APT93_26470 | KSX88195 | fimbrial protein | Escherichia coli | 809.7 | 6.40E-240 |
| FimH | 801 | G741_04605 | EQP63493 | protein fimH | Escherichia coli HVH 78 (4-2735946) | 809.7 | 6.40E-240 |
| FimH | 802 | A151_00057 | EOV12099 | fimbrial protein FimH | Escherichia coli KTE195 | 809.7 | 6.40E-240 |
| FimH | 803 | G684_04796 | EQN13467 | protein fimH | Escherichia coli HVH 4 (4-7276109) | 809.7 | 6.40E-240 |
| FimH | 804 | AD15_4353 | KDT42015 | protein fimH | Escherichia coli 3-105-05_S4_C2 | 809.7 | 6.40E-240 |
| FimH | 805 | HMPREF9350_02457 | EFU35801 | protein FimH | Escherichia coli MS 85-1 | 809.6 | 6.60E-240 |
| FimH | 806 | EC970264_4955 | KIO83560 | mannose-binding domain protein FimH | Escherichia coli 97.0264 | 809.6 | 6.60E-240 |
| FimH | 807 | ECW26_02900 | EID69032 | fimbrial protein | Escherichia coli W26 | 809.6 | 6.60E-240 |
| FimH | 808 | HMPREF1592_04020 | ESA74045 | protein FimH | Escherichia coli 907357 | 809.6 | 6.60E-240 |
| FimH | 809 | ACN002_4586 | ALY16044 | type 1 fimbiral adhesin FimH | Escherichia coli | 809.6 | 6.60E-240 |
| FimH | 810 | HMPREF9534_02260 | EFJ81717 | protein FimH | Escherichia coli MS 69-1 | 809.6 | 6.60E-240 |
| FimH | 811 | HMPREF1608_03724 | ESD66831 | protein FimH | Escherichia coli 908525 | 809.6 | 6.60E-240 |
| FimH | 812 | HMPREF1590_02968 | ESC97937 | protein FimH | Escherichia coli 113302 | 809.6 | 6.60E-240 |
| FimH | 813 | HMPREF9535_02852 | EFK73222 | protein FimH | Escherichia coli MS 78-1 | 809.6 | 6.60E-240 |
| FimH | 814 | HMPREF1598_03570 | ESD18261 | protein FimH | Escherichia coli 907710 | 809.6 | 6.60E-240 |
| FimH | 815 | HMPREF9542_04438 | EGB86157 | protein FimH | Escherichia coli MS 117-3 | 809.6 | 6.60E-240 |
| FimH | 816 | HMPREF9346_03630 | EFK44743 | protein FimH | Escherichia coli MS 119-7 | 809.6 | 6.60E-240 |
| FimH | 817 | ECPG_01549 | EGI43222 | protein FimH | Escherichia coli H591 | 809.6 | 6.60E-240 |
| FimH | 818 | CDL30129 | CDL30129 | mannose-specific adhesin FimH | Escherichia coli ISC7 | 809.6 | 6.60E-240 |
| FimH | 819 | SSJG_02837 | EGJ06788 | protein FimH | Escherichia coli D9 | 809.6 | 6.60E-240 |
| FimH | 820 | HMPREF1611_03517 | ESD82377 | protein FimH | Escherichia coli 908573 | 809.6 | 6.60E-240 |
| FimH | 821 | HMPREF1595_03996 | ESD04901 | protein FimH | Escherichia coli 907672 | 809.6 | 6.60E-240 |
| FimH | 822 | HMPREF9347_03730 | EFK67380 | protein FimH | Escherichia coli MS 124-1 | 809.6 | 6.60E-240 |
| FimH | 823 | ECAI27_01330 | EIE57787 | fimbrial protein | Escherichia coli AI27 | 809.6 | 6.60E-240 |
| FimH | 824 | HMPREF1609_05017 | ESD66396 | protein FimH | Escherichia coli 908541 | 809.6 | 6.60E-240 |
| FimH | 825 | HMPREF9536_03313 | EFJ86413 | protein FimH | Escherichia coli MS 84-1 | 809.6 | 6.60E-240 |
| FimH | 826 | HMPREF9348_02800 | EFO57973 | protein FimH | Escherichia coli MS 145-7 | 809.6 | 6.60E-240 |
| FimH | 827 | EL76_4112 | KGM58309 | Protein FimH | Escherichia coli G3/10 | 809.6 | 6.60E-240 |
| FimH | 828 | ECOG_02493 | EGI52684 | FimH protein | Escherichia coli H299 | 809.6 | 6.60E-240 |
| FimH | 829 | HMPREF9548_04471 | EFK00872 | protein FimH | Escherichia coli MS 182-1 | 809.6 | 6.60E-240 |
| FimH | 830 | G905_04660 | EQW45353 | protein fimH | Escherichia coli UMEA 3087-1 | 809.5 | 7.10E-240 |
| FimH | 831 | G992_04410 | EQZ85482 | protein fimH | Escherichia coli UMEA 3705-1 | 809.5 | 7.20E-240 |
| FimH | 832 | A13G_00250 | EOX26806 | fimbrial protein FimH | Escherichia coli KTE185 | 809.5 | 7.30E-240 |
| FimH | 833 | WGW_00016 | ELJ90797 | fimbrial protein FimH | Escherichia coli KTE94 | 809.5 | 7.30E-240 |
| FimH | 834 | OK10_17315 | OAO72647 | fimbrial protein | Escherichia coli (GCA_001651715) | 809.2 | 9.00E-240 |
| FimH | 835 | ERS085436_00952 | CTR19434 | FimH protein | Escherichia coli (GCA_001283985) | 809.2 | 9.10E-240 |
| FimH | 836 | HMPREF9549_02316 | EFJ56285 | fimbrial protein | Escherichia coli MS 185-1 | 809.1 | 9.30E-240 |
| FimH | 837 | G715_04539 | EQO48523 | protein fimH | Escherichia coli HVH 40 (4-1219782) | 809.1 | 9.60E-240 |
| FimH | 838 | AD40_5112 | KEN62238 | protein fimH | Escherichia coli 1-392-07_S4_C3 | 808.9 | 1.10E-239 |
| FimH | 839 | AD31_5188 | KEJ42775 | protein fimH | Escherichia coli 2-427-07_S4_C3 | 808.9 | 1.10E-239 |
| FimH | 840 | AB05_5048 | KEO24647 | protein fimH | Escherichia coli 2-460-02_S1_C1 | 808.9 | 1.10E-239 |
| FimH | 841 | AC84_5022 | KEN94699 | protein fimH | Escherichia coli 1-392-07_S4_C1 | 808.9 | 1.10E-239 |
| FimH | 842 | AC88_5113 | KEJ55387 | protein fimH | Escherichia coli 3-267-03_S4_C1 | 808.9 | 1.10E-239 |
| FimH | 843 | VK74_13505 | AKC13566 | fimbrial protein | Escherichia coli (GCA_001284605) | 808.8 | 1.20E-239 |
| FimH | 844 | G936_04821 | EQX71423 | protein fimH | Escherichia coli UMEA 3193-1 | 808.6 | 1.40E-239 |
| FimH | 845 | A1YC_00310 | EOW41405 | fimbrial protein FimH | Escherichia coli KTE126 | 808.4 | 1.60E-239 |
| FimH | 846 | A1S3_00309 | ELD93561 | protein fimH | Escherichia coli KTE47 | 808.4 | 1.60E-239 |
| FimH | 847 | ERS085340_04025 | CTT77731 | FimH protein | Escherichia coli (GCA_001283825) | 807.9 | 2.20E-239 |
| FimH | 848 | ECP03047774_4808 | ENE90363 | protein fimH | Escherichia coli P0304777.4 | 807.8 | 2.40E-239 |
| FimH | 849 | ECDEC14A_4847 | EHX71040 | protein fimH | Escherichia coli DEC14A | 807.7 | 2.40E-239 |
| FimH | 850 | A1US_00277 | ELG30555 | fimbrial protein FimH | Escherichia coli KTE78 | 807.7 | 2.50E-239 |
| FimH | 851 | ECMG_03848 | EGI29016 | protein FimH | Escherichia coli TA143 | 807.7 | 2.50E-239 |
| FimH | 852 | G926_04504 | EQX26749 | protein fimH | Escherichia coli UMEA 3163-1 | 807.7 | 2.50E-239 |
| FimH | 853 | A1Y1_04532 | ELG45760 | fimbrial protein FimH | Escherichia coli KTE115 | 807.7 | 2.50E-239 |
| FimH | 854 | AE24_04582 | KDG86409 | protein fimH | Escherichia coli UCI 65 | 807.7 | 2.50E-239 |
| FimH | 855 | ERYG_00377 | KNA43568 | FimH protein | Escherichia coli M114 | 807.7 | 2.50E-239 |
| FimH | 856 | G686_04541 | EQN19243 | protein fimH | Escherichia coli HVH 6 (3-8296502) | 807.7 | 2.50E-239 |
| FimH | 857 | A1UU_01771 | ELG33925 | fimbrial protein FimH | Escherichia coli KTE79 | 807.7 | 2.50E-239 |
| FimH | 858 | ERS085429_01865 | CTU96511 | FimH protein | Escherichia coli | 807.7 | 2.50E-239 |
| FimH | 859 | PU31_20230 | KHI41582 | fimbrial protein | Escherichia coli (GCA_000797775) | 807.7 | 2.50E-239 |
| FimH | 860 | EC890511_5607 | EKV70675 | protein fimH | Escherichia coli 89.0511 | 807.1 | 3.80E-239 |
| FimH | 861 | BX89_02505 | EZQ47412 | fimbrial protein | Escherichia coli O157: str. 2010EL-2044 | 807.1 | 3.80E-239 |
| FimH | 862 | QYC_0036 | ERB88995 | protein fimH | Escherichia coli B102 | 807.1 | 3.80E-239 |
| FimH | 863 | ECDEC3D_5459 | EHU68128 | minor fimbrial subunit, D-mannose specific adhesin | Escherichia coli DEC3D | 807.1 | 3.80E-239 |
| FimH | 864 | EC970010_5673 | EKY34643 | protein fimH | Escherichia coli 97.0010 | 807.1 | 3.80E-239 |
| FimH | 865 | S1W_0037 | ERD91797 | protein fimH | Escherichia coli B84 | 807.1 | 3.80E-239 |
| FimH | 866 | ECPA23_5626 | EKH63870 | minor fimbrial subunit/D-mannose specific adhesin | Escherichia coli PA23 | 807.1 | 3.80E-239 |
| FimH | 867 | BY31_07780 | EYW25794 | fimbrial protein | Escherichia coli O157:H7 str. 2011EL-2114 | 807.1 | 3.80E-239 |
| FimH | 868 | BY93_18405 | EZD17501 | fimbrial protein | Escherichia coli O157:H7 str. K6590 | 807.1 | 3.80E-239 |
| FimH | 869 | BY50_12620 | EZB11547 | fimbrial protein | Escherichia coli O157:H7 str. F6751 | 807.1 | 3.80E-239 |
| FimH | 870 | S31_4998 | ERD88452 | protein fimH | Escherichia coli B86 | 807.1 | 3.80E-239 |
| FimH | 871 | BY15_00170 | EYX44011 | fimbrial protein | Escherichia coli O157:H7 str. 2011EL-2094 | 807.1 | 3.80E-239 |
| FimH | 872 | BY24_25810 | EYW64030 | fimbrial protein | Escherichia coli O157:H7 str. 2011EL-2106 | 807.1 | 3.80E-239 |
| FimH | 873 | BY27_03940 | EYW49473 | fimbrial protein | Escherichia coli O157:H7 str. 2011EL-2109 | 807.1 | 3.80E-239 |
| FimH | 874 | BX36_01515 | EYV67666 | fimbrial protein | Escherichia coli O157:H7 str. 2009EL2109 | 807.1 | 3.80E-239 |
| FimH | 875 | S35_0035 | ERD05041 | protein fimH | Escherichia coli B104 | 807.1 | 3.80E-239 |
| FimH | 876 | BY83_24050 | EZC70440 | fimbrial protein | Escherichia coli O157:H7 str. K5448 | 807.1 | 3.80E-239 |
| FimH | 877 | EC960428_0037 | EKW51283 | protein fimH | Escherichia coli 96.0428 | 807.1 | 3.80E-239 |
| FimH | 878 | EC991781_0023 | ELW23889 | protein fimH | Escherichia coli 99.1781 | 807.1 | 3.80E-239 |
| FimH | 879 | ACP61_05365 | KOZ32829 | fimbrial protein | Escherichia coli | 807.1 | 3.80E-239 |
| FimH | 880 | ECTW10246_0284 | EIO63581 | minor fimbrial subunit/D-mannose specific adhesin | Escherichia coli TW10246 | 807.1 | 3.80E-239 |
| FimH | 881 | QYE_0104 | ERB79237 | protein fimH | Escherichia coli B107 | 807.1 | 3.80E-239 |
| FimH | 882 | EC52239_0036 | EKK36724 | protein fimH | Escherichia coli 5.2239 | 807.1 | 3.80E-239 |
| FimH | 883 | ECH7EC4501_2834 | EDU87388 | protein FimH | Escherichia coli O157:H7 str. EC4501 | 807.1 | 3.80E-239 |
| FimH | 884 | EC09BKT24447_0106 | ERE21820 | protein fimH | Escherichia coli 09BKT024447 | 807.1 | 3.80E-239 |
| FimH | 885 | BAB38702 | BAB38702 | minor fimbrial subunit/D-mannose specific adhesin | Escherichia coli O157:H7 str. Sakai | 807.1 | 3.80E-239 |
| FimH | 886 | BX90_01425 | EZQ50264 | fimbrial protein | Escherichia coli O157: str. 2010EL-2045 | 807.1 | 3.80E-239 |
| FimH | 887 | BY70_17570 | EZC05155 | fimbrial protein | Escherichia coli O157:H7 str. K2192 | 807.1 | 3.80E-239 |
| FimH | 888 | EC940618_5456 | EKW08508 | protein fimH | Escherichia coli 94.0618 | 807.1 | 3.80E-239 |
| FimH | 889 | ECCB7326_5682 | EKH96704 | minor fimbrial subunit/D-mannose specific adhesin | Escherichia coli CB7326 | 807.1 | 3.80E-239 |
| FimH | 890 | BX33_22545 | EZE88824 | fimbrial protein | Escherichia coli O157:H7 str. 2009EL1449 | 807.1 | 3.80E-239 |
| FimH | 891 | S37_0037 | ERD35196 | protein fimH | Escherichia coli B109 | 807.1 | 3.80E-239 |
| FimH | 892 | BY14_02825 | EYX44863 | fimbrial protein | Escherichia coli O157:H7 str. 2011EL-2093 | 807.1 | 3.80E-239 |
| FimH | 893 | S1U_0035 | ERD88011 | protein fimH | Escherichia coli B83 | 807.1 | 3.80E-239 |
| FimH | 894 | BY91_01435 | EYV84705 | fimbrial protein | Escherichia coli O157:H7 str. K5806 | 807.1 | 3.80E-239 |
| FimH | 895 | ECoA_01160 | EGD70511 | mannose-specific adhesin FimH | Escherichia coli O157:H7 str. 1044 | 807.1 | 3.80E-239 |
| FimH | 896 | S15_0038 | ERD72620 | protein fimH | Escherichia coli B40-1 | 807.1 | 3.80E-239 |
| FimH | 897 | BY56_10170 | EZB32033 | fimbrial protein | Escherichia coli O157:H7 str. G5303 | 807.1 | 3.80E-239 |
| FimH | 898 | EC970003_0036 | EKW68034 | protein fimH | Escherichia coli 97.0003 | 807.1 | 3.80E-239 |
| FimH | 899 | BY64_13460 | EZB74708 | fimbrial protein | Escherichia coli O157:H7 str. K1796 | 807.1 | 3.80E-239 |
| FimH | 900 | BY16_15940 | EYX32986 | fimbrial protein | Escherichia coli O157:H7 str. 2011EL-2096 | 807.1 | 3.80E-239 |
| FimH | 901 | EC930056_0036 | EKW19046 | protein fimH | Escherichia coli 93.0056 | 807.1 | 3.80E-239 |
| FimH | 902 | Z5918 | AAG59502 | minor fimbrial subunit, D-mannose specific adhesin | Escherichia coli O157:H7 str. EDL933 (GCA_000732965) | 807.1 | 3.80E-239 |
| FimH | 903 | EC34880_5151 | ELW28338 | protein fimH | Escherichia coli 3.4880 | 807.1 | 3.80E-239 |
| FimH | 904 | ECEC4421_5514 | EIP10322 | minor fimbrial subunit/D-mannose specific adhesin | Escherichia coli EC4421 | 807.1 | 3.80E-239 |
| FimH | 905 | ECFRIK1997_0039 | EKH48853 | minor fimbrial subunit/D-mannose specific adhesin | Escherichia coli FRIK1997 | 807.1 | 3.80E-239 |
| FimH | 906 | BY58_12015 | EZB47003 | fimbrial protein | Escherichia coli O157:H7 str. H2498 | 807.1 | 3.80E-239 |
| FimH | 907 | BY30_04770 | EYW34629 | fimbrial protein | Escherichia coli O157:H7 str. 2011EL-2113 | 807.1 | 3.80E-239 |
| FimH | 908 | BY18_15125 | EYX27779 | fimbrial protein | Escherichia coli O157:H7 str. 2011EL-2098 | 807.1 | 3.80E-239 |
| FimH | 909 | QYY_0037 | ERD83209 | protein fimH | Escherichia coli B5-2 | 807.1 | 3.80E-239 |
| FimH | 910 | BY72_11000 | EZC17267 | fimbrial protein | Escherichia coli O157:H7 str. K2581 | 807.1 | 3.80E-239 |
| FimH | 911 | ECPA31_5538 | EIO07006 | minor fimbrial subunit/D-mannose specific adhesin | Escherichia coli PA31 | 807.1 | 3.80E-239 |
| FimH | 912 | BY12_10530 | EYX54530 | fimbrial protein | Escherichia coli O157:H7 str. 2011EL-2091 | 807.1 | 3.80E-239 |
| FimH | 913 | BY88_08920 | EZC92789 | fimbrial protein | Escherichia coli O157:H7 str. K5602 | 807.1 | 3.80E-239 |
| FimH | 914 | ECPA45_0036 | EKH87922 | minor fimbrial subunit/D-mannose specific adhesin | Escherichia coli PA45 | 807.1 | 3.80E-239 |
| FimH | 915 | EC950943_0035 | EKW36598 | protein fimH | Escherichia coli 95.0943 | 807.1 | 3.80E-239 |
| FimH | 916 | BY71_01695 | EZB95424 | fimbrial protein | Escherichia coli O157:H7 str. K2324 | 807.1 | 3.80E-239 |
| FimH | 917 | ECFDA506_0360 | EKH25307 | minor fimbrial subunit/D-mannose specific adhesin | Escherichia coli FDA506 | 807.1 | 3.80E-239 |
| FimH | 918 | B233_0036 | ERD09285 | protein fimH | Escherichia coli 2886-75 | 807.1 | 3.80E-239 |
| FimH | 919 | ECFDA517_5917 | EIN16654 | minor fimbrial subunit/D-mannose specific adhesin | Escherichia coli FDA517 | 807.1 | 3.80E-239 |
| FimH | 920 | EC991793_0025 | ELV62483 | protein fimH | Escherichia coli 99.1793 | 807.1 | 3.80E-239 |
| FimH | 921 | BY75_13275 | EZC30143 | fimbrial protein | Escherichia coli O157:H7 str. K2854 | 807.1 | 3.80E-239 |
| FimH | 922 | S1Q_4942 | ERD45149 | protein fimH | Escherichia coli B17 | 807.1 | 3.80E-239 |
| FimH | 923 | ECPA10_5916 | EIN65436 | minor fimbrial subunit/D-mannose specific adhesin | Escherichia coli PA10 | 807.1 | 3.80E-239 |
| FimH | 924 | EC960107_0035 | EKW74802 | protein fimH | Escherichia coli 96.0107 | 807.1 | 3.80E-239 |
| FimH | 925 | BY52_08995 | EZB20602 | fimbrial protein | Escherichia coli O157:H7 str. F7377 | 807.1 | 3.80E-239 |
| FimH | 926 | EC951288_5384 | EKW22401 | protein fimH | Escherichia coli 95.1288 | 807.1 | 3.80E-239 |
| FimH | 927 | CDCO157_4964 | AFJ32012 | D-mannose specific adhesin | Escherichia coli Xuzhou21 | 807.1 | 3.80E-239 |
| FimH | 928 | EC34870_0036 | EKK37416 | protein fimH | Escherichia coli 3.4870 | 807.1 | 3.80E-239 |
| FimH | 929 | EC930055_5504 | EKW04239 | protein fimH | Escherichia coli 93.0055 | 807.1 | 3.80E-239 |
| FimH | 930 | S11_0102 | ERB91898 | protein fimH | Escherichia coli B26-1 | 807.1 | 3.80E-239 |
| FimH | 931 | ECFRIK523_5610 | EKJ48086 | minor fimbrial subunit/D-mannose specific adhesin | Escherichia coli FRIK523 | 807.1 | 3.80E-239 |
| FimH | 932 | BY89_20770 | EZC86971 | fimbrial protein | Escherichia coli O157:H7 str. K5607 | 807.1 | 3.80E-239 |
| FimH | 933 | BY13_13715 | EYX48116 | fimbrial protein | Escherichia coli O157:H7 str. 2011EL-2092 | 807.1 | 3.80E-239 |
| FimH | 934 | BW95_22310 | EYZ37434 | fimbrial protein | Escherichia coli O157:H7 str. 07-3091 | 807.1 | 3.80E-239 |
| FimH | 935 | BY11_12725 | EYX57425 | fimbrial protein | Escherichia coli O157:H7 str. 2011EL-2090 | 807.1 | 3.80E-239 |
| FimH | 936 | BY74_13525 | EZC25637 | fimbrial protein | Escherichia coli O157:H7 str. K2845 | 807.1 | 3.80E-239 |
| FimH | 937 | EC990713_0036 | EKW96281 | protein fimH | Escherichia coli 99.0713 | 807.1 | 3.80E-239 |
| FimH | 938 | BY19_06015 | EYW85314 | fimbrial protein | Escherichia coli O157:H7 str. 2011EL-2099 | 807.1 | 3.80E-239 |
| FimH | 939 | BW98_01025 | EYX13645 | fimbrial protein | Escherichia coli O157:H7 str. 08-3037 | 807.1 | 3.80E-239 |
| FimH | 940 | BY84_03670 | EZC77680 | fimbrial protein | Escherichia coli O157:H7 str. K5449 | 807.1 | 3.80E-239 |
| FimH | 941 | S1O_5050 | ERD40253 | protein fimH | Escherichia coli B15 | 807.1 | 3.80E-239 |
| FimH | 942 | BY22_04650 | EYW71169 | fimbrial protein | Escherichia coli O157:H7 str. 2011EL-2104 | 807.1 | 3.80E-239 |
| FimH | 943 | ECTT12B_0037 | EKH95588 | minor fimbrial subunit/D-mannose specific adhesin | Escherichia coli TT12B | 807.1 | 3.80E-239 |
| FimH | 944 | BY20_01425 | EYW83347 | fimbrial protein | Escherichia coli O157:H7 str. 2011EL-2101 | 807.1 | 3.80E-239 |
| FimH | 945 | ECNE037_0039 | EKH61569 | minor fimbrial subunit/D-mannose specific adhesin | Escherichia coli NE037 | 807.1 | 3.80E-239 |
| FimH | 946 | ECPA7_0407 | EKH07702 | minor fimbrial subunit/D-mannose specific adhesin | Escherichia coli PA7 | 807.1 | 3.80E-239 |
| FimH | 947 | BY51_15780 | EYV89267 | fimbrial protein | Escherichia coli O157:H7 str. F7350 | 807.1 | 3.80E-239 |
| FimH | 948 | BX01_14875 | EYX02222 | fimbrial protein | Escherichia coli O157:H7 str. 08-4169 | 807.1 | 3.80E-239 |
| FimH | 949 | EC902281_0036 | EKW03266 | protein fimH | Escherichia coli 90.2281 | 807.1 | 3.80E-239 |
| FimH | 950 | BY90_06585 | EZD00249 | fimbrial protein | Escherichia coli O157:H7 str. K5609 | 807.1 | 3.80E-239 |
| FimH | 951 | BZ06_06610 | EZD75286 | fimbrial protein | Escherichia coli O157:H7 str. K7140 | 807.1 | 3.80E-239 |
| FimH | 952 | BY63_06760 | EZB71558 | fimbrial protein | Escherichia coli O157:H7 str. K1795 | 807.1 | 3.80E-239 |
| FimH | 953 | EC950083_5044 | ELW29973 | protein fimH | Escherichia coli 95.0083 | 807.1 | 3.80E-239 |
| FimH | 954 | BY48_01425 | EZB05956 | fimbrial protein | Escherichia coli O157:H7 str. F6749 | 807.1 | 3.80E-239 |
| FimH | 955 | ECDEC3A_5324 | EHU51912 | minor fimbrial subunit, D-mannose specific adhesin | Escherichia coli DEC3A | 807.1 | 3.80E-239 |
| FimH | 956 | BY76_10715 | EZC37627 | fimbrial protein | Escherichia coli O157:H7 str. K4396 | 807.1 | 3.80E-239 |
| FimH | 957 | ECFDA507_5685 | EKH13374 | minor fimbrial subunit/D-mannose specific adhesin | Escherichia coli FDA507 | 807.1 | 3.80E-239 |
| FimH | 958 | BY17_20265 | EYX30678 | fimbrial protein | Escherichia coli O157:H7 str. 2011EL-2097 | 807.1 | 3.80E-239 |
| FimH | 959 | ECFDA504_0038 | EKH36339 | minor fimbrial subunit/D-mannose specific adhesin | Escherichia coli FDA504 | 807.1 | 3.80E-239 |
| FimH | 960 | ECPA22_0101 | EIN94399 | minor fimbrial subunit/D-mannose specific adhesin | Escherichia coli PA22 | 807.1 | 3.80E-239 |
| FimH | 961 | EC991775_4981 | ELV47149 | protein fimH | Escherichia coli 99.1775 | 807.1 | 3.80E-239 |
| FimH | 962 | S1Y_0037 | ERD98704 | protein fimH | Escherichia coli B85 | 807.1 | 3.80E-239 |
| FimH | 963 | Q455_0213300 | ETJ79073 | fimbrial protein FimH | Escherichia coli ATCC BAA-2192 | 807.1 | 3.80E-239 |
| FimH | 964 | EC881042_0036 | EKV85120 | protein fimH | Escherichia coli 88.1042 | 807.1 | 3.80E-239 |
| FimH | 965 | EC970007_5019 | EKW71392 | protein fimH | Escherichia coli 97.0007 | 807.1 | 3.80E-239 |
| FimH | 966 | EC01304_5758 | EKJ55018 | minor fimbrial subunit/D-mannose specific adhesin | Escherichia coli 0.1304 | 807.1 | 3.80E-239 |
| FimH | 967 | EC80586_0037 | EKK61111 | protein fimH | Escherichia coli 8.0586 | 807.1 | 3.80E-239 |
| FimH | 968 | BY86_08115 | EZC83432 | fimbrial protein | Escherichia coli O157:H7 str. K5460 | 807.1 | 3.80E-239 |
| FimH | 969 | ECPA4_0038 | EKH71840 | minor fimbrial subunit/D-mannose specific adhesin | Escherichia coli PA4 | 807.1 | 3.80E-239 |
| FimH | 970 | EC100821_0037 | EKK91205 | protein fimH | Escherichia coli 10.0821 | 807.1 | 3.80E-239 |
| FimH | 971 | EC960109_5705 | EKY34555 | protein fimH | Escherichia coli 96.0109 | 807.1 | 3.80E-239 |
| FimH | 972 | ECEC1738_0102 | EIP63369 | minor fimbrial subunit/D-mannose specific adhesin | Escherichia coli EC1738 | 807.1 | 3.80E-239 |
| FimH | 973 | ASO15_27335 | KRQ04775 | fimbrial protein | Escherichia coli O157:H7 | 807.1 | 3.80E-239 |
| FimH | 974 | ECDEC4F_5262 | EHV16600 | minor fimbrial subunit, D-mannose specific adhesin | Escherichia coli DEC4F | 807.1 | 3.80E-239 |
| FimH | 975 | BY23_05510 | EYW67161 | fimbrial protein | Escherichia coli O157:H7 str. 2011EL-2105 | 807.1 | 3.80E-239 |
| FimH | 976 | ECEC4422_0034 | EIP33963 | minor fimbrial subunit/D-mannose specific adhesin | Escherichia coli EC4422 | 807.1 | 3.80E-239 |
| FimH | 977 | BY85_22385 | EZC64777 | fimbrial protein | Escherichia coli O157:H7 str. K5453 | 807.1 | 3.80E-239 |
| FimH | 978 | ECFDA505_5591 | EIN15798 | minor fimbrial subunit/D-mannose specific adhesin | Escherichia coli FDA505 | 807.1 | 3.80E-239 |
| FimH | 979 | BW99_13265 | EYX12309 | fimbrial protein | Escherichia coli O157:H7 str. 08-3527 | 807.1 | 3.80E-239 |
| FimH | 980 | EC991753_5095 | ELV44357 | protein fimH | Escherichia coli 99.1753 | 807.1 | 3.80E-239 |
| FimH | 981 | ECPA38_5451 | EKI33215 | minor fimbrial subunit/D-mannose specific adhesin | Escherichia coli PA38 | 807.1 | 3.80E-239 |
| FimH | 982 | S3E_0037 | ERD22972 | protein fimH | Escherichia coli B106 | 807.1 | 3.80E-239 |
| FimH | 983 | S17_0038 | ERD68399 | protein fimH | Escherichia coli B40-2 | 807.1 | 3.80E-239 |
| FimH | 984 | ECPA15_0037 | EIN85437 | minor fimbrial subunit/D-mannose specific adhesin | Escherichia coli PA15 | 807.1 | 3.80E-239 |
| FimH | 985 | ECFRIK1985_5883 | EIN32996 | minor fimbrial subunit/D-mannose specific adhesin | Escherichia coli FRIK1985 | 807.1 | 3.80E-239 |
| FimH | 986 | ECoD_01979 | EFW65215 | mannose-specific adhesin FimH | Escherichia coli O157:H7 str. EC1212 | 807.1 | 3.80E-239 |
| FimH | 987 | BY87_00175 | EZC90351 | fimbrial protein | Escherichia coli O157:H7 str. K5467 | 807.1 | 3.80E-239 |
| FimH | 988 | BY28_12290 | EYW43030 | fimbrial protein | Escherichia coli O157:H7 str. 2011EL-2111 | 807.1 | 3.80E-239 |
| FimH | 989 | ECTW11039_0038 | EIO69679 | minor fimbrial subunit/D-mannose specific adhesin | Escherichia coli TW11039 | 807.1 | 3.80E-239 |
| FimH | 990 | BY21_12450 | EYW75539 | fimbrial protein | Escherichia coli O157:H7 str. 2011EL-2103 | 807.1 | 3.80E-239 |
| FimH | 991 | S1I_0037 | ERD08452 | protein fimH | Escherichia coli B103 | 807.1 | 3.80E-239 |
| FimH | 992 | S3C_0038 | ERD20768 | protein fimH | Escherichia coli B105 | 807.1 | 3.80E-239 |
| FimH | 993 | BX34_16715 | EYV70474 | fimbrial protein | Escherichia coli O157:H7 str. 2009EL1705 | 807.1 | 3.80E-239 |
| FimH | 994 | ECPA5_5570 | EIN52288 | minor fimbrial subunit/D-mannose specific adhesin | Escherichia coli PA5 | 807.1 | 3.80E-239 |
| FimH | 995 | S3A_0036 | ERD73562 | protein fimH | Escherichia coli B49-2 | 807.1 | 3.80E-239 |
| FimH | 996 | BY65_09935 | EZB77230 | fimbrial protein | Escherichia coli O157:H7 str. K1845 | 807.1 | 3.80E-239 |
| FimH | 997 | BY26_11440 | EYW49160 | fimbrial protein | Escherichia coli O157:H7 str. 2011EL-2108 | 807.1 | 3.80E-239 |
| FimH | 998 | BW94_20830 | EYZ38289 | fimbrial protein | Escherichia coli O157:H7 str. 06-4039 | 807.1 | 3.80E-239 |
| FimH | 999 | EC960427_5629 | EKW38083 | protein fimH | Escherichia coli 96.0427 | 807.1 | 3.80E-239 |
| FimH | 1000 | ECDEC4A_5335 | EHU86083 | minor fimbrial subunit, D-mannose specific adhesin | Escherichia coli DEC4A | 807.1 | 3.80E-239 |
| FimH | 1001 | ECPA42_0037 | EIO49147 | minor fimbrial subunit/D-mannose specific adhesin | Escherichia coli PA42 | 807.1 | 3.80E-239 |
| FimH | 1002 | ECT92401_0027 | ERC92390 | protein fimH | Escherichia coli T924_01 | 807.1 | 3.80E-239 |
| FimH | 1003 | ECPA49_0036 | EKH83451 | minor fimbrial subunit/D-mannose specific adhesin | Escherichia coli PA49 | 807.1 | 3.80E-239 |
| FimH | 1004 | ECDEC3B_5551 | EHU53050 | minor fimbrial subunit, D-mannose specific adhesin | Escherichia coli DEC3B | 807.1 | 3.80E-239 |
| FimH | 1005 | BY69_11265 | EZB98887 | fimbrial protein | Escherichia coli O157:H7 str. K2191 | 807.1 | 3.80E-239 |
| FimH | 1006 | ECPA14_5744 | EIN69780 | minor fimbrial subunit/D-mannose specific adhesin | Escherichia coli PA14 | 807.1 | 3.80E-239 |
| FimH | 1007 | ECTW09109_0103 | EIO79123 | minor fimbrial subunit/D-mannose specific adhesin | Escherichia coli TW09109 | 807.1 | 3.80E-239 |
| FimH | 1008 | BY25_21430 | EYW57158 | fimbrial protein | Escherichia coli O157:H7 str. 2011EL-2107 | 807.1 | 3.80E-239 |
| FimH | 1009 | S33_0038 | ERD24013 | protein fimH | Escherichia coli B108 | 807.1 | 3.80E-239 |
| FimH | 1010 | BY68_02645 | EZB81713 | fimbrial protein | Escherichia coli O157:H7 str. K2188 | 807.1 | 3.80E-239 |
| FimH | 1011 | S13_0105 | ERC00124 | protein fimH | Escherichia coli B26-2 | 807.1 | 3.80E-239 |
| FimH | 1012 | ECNE098_0036 | EKJ53176 | minor fimbrial subunit/D-mannose specific adhesin | Escherichia coli NE098 | 807.1 | 3.80E-239 |
| FimH | 1013 | BY61_02730 | EZB59816 | fimbrial protein | Escherichia coli O157:H7 str. K1792 | 807.1 | 3.80E-239 |
| FimH | 1014 | BY62_06910 | EZB55863 | fimbrial protein | Escherichia coli O157:H7 str. K1793 | 807.1 | 3.80E-239 |
| FimH | 1015 | EC990670_0037 | ELW46392 | protein fimH | Escherichia coli 99.0670 | 807.1 | 3.80E-239 |
| FimH | 1016 | ECDEC3C_0114 | EHU79284 | minor fimbrial subunit, D-mannose specific adhesin | Escherichia coli DEC3C | 807.1 | 3.80E-239 |
| FimH | 1017 | BY57_07585 | EZB41542 | fimbrial protein | Escherichia coli O157:H7 str. H2495 | 807.1 | 3.80E-239 |
| FimH | 1018 | BY29_17775 | EYW39180 | fimbrial protein | Escherichia coli O157:H7 str. 2011EL-2112 | 807.1 | 3.80E-239 |
| FimH | 1019 | ESCCO14588_1214 | EEC30722 | protein FimH | Escherichia coli O157:H7 str. TW14588 | 807.1 | 3.80E-239 |
| FimH | 1020 | ECPA33_5598 | EIO11138 | minor fimbrial subunit/D-mannose specific adhesin | Escherichia coli PA33 | 807.1 | 3.80E-239 |
| FimH | 1021 | EC60172_0037 | EKK37856 | minor fimbrial subunit/D-mannose specific adhesin | Escherichia coli 6.0172 | 807.1 | 3.80E-239 |
| FimH | 1022 | BY73_25935 | EZC08758 | fimbrial protein | Escherichia coli O157:H7 str. K2622 | 807.1 | 3.80E-239 |
| FimH | 1023 | ECPA11_0035 | ELV75988 | protein fimH | Escherichia coli PA11 | 807.1 | 3.80E-239 |
| FimH | 1024 | BY92_10540 | EZD08950 | fimbrial protein | Escherichia coli O157:H7 str. K5852 | 807.1 | 3.80E-239 |
| FimH | 1025 | BY49_01610 | EZB04385 | fimbrial protein | Escherichia coli O157:H7 str. F6750 | 807.1 | 3.80E-239 |
| FimH | 1026 | EC93001_5709 | EIN33859 | minor fimbrial subunit/D-mannose specific adhesin | Escherichia coli 93-001 | 807.1 | 3.80E-239 |
| FimH | 1027 | ECPA32_5618 | EIO07275 | minor fimbrial subunit/D-mannose specific adhesin | Escherichia coli PA32 | 807.1 | 3.80E-239 |
| FimH | 1028 | AB46_4346 | KDU00185 | protein fimH | Escherichia coli 3-267-03_S1_C2 | 807.1 | 3.80E-239 |
| FimH | 1029 | G805_04508 | EQS28707 | protein fimH | Escherichia coli HVH 147 (4-5893887) | 807.1 | 3.90E-239 |
| FimH | 1030 | ECP03052605_4612 | ENG08612 | protein fimH | Escherichia coli P0305260.5 | 807.1 | 4.00E-239 |
| FimH | 1031 | A1YA_02100 | ELG61916 | fimbrial protein FimH | Escherichia coli KTE123 | 807.1 | 4.00E-239 |
| FimH | 1032 | ECP03052603_4705 | ENF97401 | protein fimH | Escherichia coli P0305260.3 | 807.1 | 4.00E-239 |
| FimH | 1033 | ECP03052609_4676 | ENG27749 | protein fimH | Escherichia coli P0305260.9 | 807.1 | 4.00E-239 |
| FimH | 1034 | AB37_4683 | KEO04670 | protein fimH | Escherichia coli 8-415-05_S1_C2 | 807.1 | 4.00E-239 |
| FimH | 1035 | G739_04737 | EQP57050 | protein fimH | Escherichia coli HVH 76 (4-2538717) | 807.1 | 4.00E-239 |
| FimH | 1036 | G851_04739 | EQU06192 | protein fimH | Escherichia coli HVH 199 (4-5670322) | 807.1 | 4.00E-239 |
| FimH | 1037 | G778_04653 | EQR01028 | protein fimH | Escherichia coli HVH 116 (4-6879942) | 807.1 | 4.00E-239 |
| FimH | 1038 | ECDEC1A_5110 | EHU02571 | minor fimbrial subunit, D-mannose specific adhesin | Escherichia coli DEC1A | 807.1 | 4.00E-239 |
| FimH | 1039 | A31I_00061 | ELF33253 | protein fimH | Escherichia coli KTE162 | 807.1 | 4.00E-239 |
| FimH | 1040 | G710_04750 | EQO25397 | protein fimH | Escherichia coli HVH 35 (4-2962667) | 807.1 | 4.00E-239 |
| FimH | 1041 | A15W_00410 | ELH78507 | fimbrial protein FimH | Escherichia coli KTE211 | 807.1 | 4.00E-239 |
| FimH | 1042 | ESPG_02748 | EHN96209 | fimH | Escherichia coli H397 | 807.1 | 4.00E-239 |
| FimH | 1043 | WG7_00036 | EOV16056 | fimbrial protein FimH | Escherichia coli KTE38 | 807.1 | 4.00E-239 |
| FimH | 1044 | AE40_03897 | KDF94837 | protein fimH | Escherichia coli BIDMC 65 | 807.1 | 4.00E-239 |
| FimH | 1045 | ESAG_03944 | EEH88232 | protein fimH | Escherichia sp. 3_2_53FAA | 807.1 | 4.00E-239 |
| FimH | 1046 | G962_04133 | EQY78990 | protein fimH | Escherichia coli UMEA 3304-1 | 807.1 | 4.00E-239 |
| FimH | 1047 | AC14_4778 | KEN70509 | protein fimH | Escherichia coli 2-052-05_S3_C2 | 807.1 | 4.00E-239 |
| FimH | 1048 | WG5_00026 | EOU99246 | fimbrial protein FimH | Escherichia coli KTE37 | 807.1 | 4.00E-239 |
| FimH | 1049 | A1SQ_00461 | ELG19621 | fimbrial protein FimH | Escherichia coli KTE59 | 807.1 | 4.00E-239 |
| FimH | 1050 | AB84_4598 | KDT14108 | protein fimH | Escherichia coli 2-052-05_S3_C1 | 807.1 | 4.00E-239 |
| FimH | 1051 | AC08_4303 | KEL11550 | protein fimH | Escherichia coli 4-203-08_S3_C1 | 807.1 | 4.00E-239 |
| FimH | 1052 | G706_04689 | EQO09241 | protein fimH | Escherichia coli HVH 30 (4-2661829) | 807.1 | 4.00E-239 |
| FimH | 1053 | G681_04683 | EQN01360 | protein fimH | Escherichia coli HVH 1 (4-6876161) | 807.1 | 4.00E-239 |
| FimH | 1054 | WK7_04603 | ELI90006 | fimbrial protein FimH | Escherichia coli KTE148 | 807.1 | 4.00E-239 |
| FimH | 1055 | OO96_18505 | OAO67084 | fimbrial protein | Escherichia coli O78:H11 (strain H10407 / ETEC) | 807.1 | 4.00E-239 |
| FimH | 1056 | G935_02346 | EQX79715 | protein fimH | Escherichia coli UMEA 3190-1 | 807.1 | 4.00E-239 |
| FimH | 1057 | ECDEC2D_0041 | EHU49215 | minor fimbrial subunit, D-mannose specific adhesin | Escherichia coli DEC2D | 807.1 | 4.00E-239 |
| FimH | 1058 | ECDEC2E_5231 | EHU48717 | minor fimbrial subunit, D-mannose specific adhesin | Escherichia coli DEC2E | 807.1 | 4.00E-239 |
| FimH | 1059 | AC78_4471 | KEN37378 | protein fimH | Escherichia coli 7-233-03_S4_C1 | 807.1 | 4.00E-239 |
| FimH | 1060 | AD45_4266 | KDU65130 | protein fimH | Escherichia coli 4-203-08_S4_C3 | 807.1 | 4.00E-239 |
| FimH | 1061 | A1Y5_00869 | ELG57361 | fimbrial protein FimH | Escherichia coli KTE118 | 807.1 | 4.00E-239 |
| FimH | 1062 | ECDEC1B_5221 | EHU04581 | minor fimbrial subunit, D-mannose specific adhesin | Escherichia coli DEC1B | 807.1 | 4.00E-239 |
| FimH | 1063 | WEA_04461 | ELF87810 | fimbrial protein FimH | Escherichia coli KTE22 | 807.1 | 4.00E-239 |
| FimH | 1064 | WK3_04473 | ELI79077 | fimbrial protein FimH | Escherichia coli KTE139 | 807.1 | 4.00E-239 |
| FimH | 1065 | G694_04559 | EQN58592 | protein fimH | Escherichia coli HVH 18 (4-8589585) | 807.1 | 4.00E-239 |
| FimH | 1066 | G749_05046 | EQP98046 | protein fimH | Escherichia coli HVH 87 (4-5977630) | 807.1 | 4.00E-239 |
| FimH | 1067 | A1SO_00594 | ELE27255 | protein fimH | Escherichia coli KTE58 | 807.1 | 4.00E-239 |
| FimH | 1068 | BW69_19295 | EZA33092 | fimbrial protein | Escherichia coli O103:H11 str. 04-3023 | 807.1 | 4.00E-239 |
| FimH | 1069 | ECDEC1E_0070 | EHU32356 | minor fimbrial subunit, D-mannose specific adhesin | Escherichia coli DEC1E | 807.1 | 4.00E-239 |
| FimH | 1070 | ECDEC2B_0016 | EHU45226 | minor fimbrial subunit, D-mannose specific adhesin | Escherichia coli DEC2B | 807.1 | 4.00E-239 |
| FimH | 1071 | G862_04596 | ERA85087 | protein fimH | Escherichia coli HVH 210 (4-3042480) | 807.1 | 4.00E-239 |
| FimH | 1072 | A1UC_05354 | EOV68218 | fimbrial protein FimH | Escherichia coli KTE70 | 807.1 | 4.00E-239 |
| FimH | 1073 | A1UK_04970 | EOV83215 | fimbrial protein FimH | Escherichia coli KTE74 | 807.1 | 4.00E-239 |
| FimH | 1074 | AB93_4328 | KEL47608 | protein fimH | Escherichia coli 5-172-05_S3_C1 | 807.1 | 4.00E-239 |
| FimH | 1075 | ECP030526012_4651 | ENF80717 | protein fimH | Escherichia coli P0305260.12 | 807.1 | 4.00E-239 |
| FimH | 1076 | G780_04724 | EQR11768 | protein fimH | Escherichia coli HVH 118 (4-7345399) | 807.1 | 4.00E-239 |
| FimH | 1077 | G863_04701 | EQU65347 | protein fimH | Escherichia coli HVH 211 (4-3041891) | 807.1 | 4.00E-239 |
| FimH | 1078 | EC236275_1850 | EFR17176 | protein fimH | Escherichia coli 2362-75 | 807.1 | 4.00E-239 |
| FimH | 1079 | G722_04498 | EQO79521 | protein fimH | Escherichia coli HVH 48 (4-2658593) | 807.1 | 4.00E-239 |
| FimH | 1080 | A1UA_05229 | EOV58208 | fimbrial protein FimH | Escherichia coli KTE69 | 807.1 | 4.00E-239 |
| FimH | 1081 | ECP03052607_4698 | ENG10883 | protein fimH | Escherichia coli P0305260.7 | 807.1 | 4.00E-239 |
| FimH | 1082 | ECP03052601_4496 | EMZ89820 | protein fimH | Escherichia coli P0305260.1 | 807.1 | 4.00E-239 |
| FimH | 1083 | A133_00466 | ELH30423 | fimbrial protein FimH | Escherichia coli KTE173 | 807.1 | 4.00E-239 |
| FimH | 1084 | ECP030526015_4649 | ENF93188 | protein fimH | Escherichia coli P0305260.15 | 807.1 | 4.00E-239 |
| FimH | 1085 | A1U3_04663 | ELG21220 | fimbrial protein FimH | Escherichia coli KTE65 | 807.1 | 4.00E-239 |
| FimH | 1086 | AB09_4607 | KEN18962 | protein fimH | Escherichia coli 8-415-05_S1_C1 | 807.1 | 4.00E-239 |
| FimH | 1087 | ECDEC2A_0098 | EHU33789 | protein fimH | Escherichia coli DEC2A | 807.1 | 4.00E-239 |
| FimH | 1088 | ECP03052606_4649 | ENG10371 | protein fimH | Escherichia coli P0305260.6 | 807.1 | 4.00E-239 |
| FimH | 1089 | A177_00301 | ELD45798 | protein fimH | Escherichia coli KTE216 | 807.1 | 4.00E-239 |
| FimH | 1090 | ECP02994382_4574 | ENA00252 | protein fimH | Escherichia coli P0299438.2 | 807.1 | 4.00E-239 |
| FimH | 1091 | A1UY_00580 | ELE75351 | protein fimH | Escherichia coli KTE81 | 807.1 | 4.00E-239 |
| FimH | 1092 | A135_00506 | ELH31332 | fimbrial protein FimH | Escherichia coli KTE175 | 807.1 | 4.00E-239 |
| FimH | 1093 | AC06_4045 | KDT63317 | protein fimH | Escherichia coli 3-373-03_S3_C1 | 807.1 | 4.00E-239 |
| FimH | 1094 | AD19_4384 | KEL06090 | protein fimH | Escherichia coli 4-203-08_S4_C2 | 807.1 | 4.00E-239 |
| FimH | 1095 | A1YW_00075 | ELF22960 | protein fimH | Escherichia coli KTE143 | 807.1 | 4.00E-239 |
| FimH | 1096 | ETEC_4632 | CBJ04141 | minor component of type 1 fimbriae | Escherichia coli ETEC H10407 | 807.1 | 4.00E-239 |
| FimH | 1097 | ECDEC1C_5251 | EHU02454 | minor fimbrial subunit, D-mannose specific adhesin | Escherichia coli DEC1C | 807.1 | 4.00E-239 |
| FimH | 1098 | G844_04755 | EQT77258 | protein fimH | Escherichia coli HVH 192 (4-3054470) | 807.1 | 4.00E-239 |
| FimH | 1099 | AC42_4720 | KDV77042 | protein fimH | Escherichia coli 2-052-05_S3_C3 | 807.1 | 4.00E-239 |
| FimH | 1100 | AD35_4423 | KEN49906 | protein fimH | Escherichia coli 7-233-03_S4_C3 | 807.1 | 4.00E-239 |
| FimH | 1101 | ECDEC2C_5328 | EHU34985 | minor fimbrial subunit, D-mannose specific adhesin | Escherichia coli DEC2C | 807.1 | 4.00E-239 |
| FimH | 1102 | ECP03052608_4696 | ENG20642 | protein fimH | Escherichia coli P0305260.8 | 807.1 | 4.00E-239 |
| FimH | 1103 | ECSF_4261 | BAI57801 | minor fimbrial subunit FimH | Escherichia coli SE15 | 807.1 | 4.00E-239 |
| FimH | 1104 | EC5411_15302 | EIL63543 | adhesin | Escherichia coli 541-1 | 807.1 | 4.00E-239 |
| FimH | 1105 | APT99_17445 | KSY18432 | fimbrial protein | Escherichia coli (GCA_001463535) | 807.1 | 4.00E-239 |
| FimH | 1106 | AC66_3049 | KDT04856 | protein fimH | Escherichia coli 2-011-08_S4_C1 | 807 | 4.10E-239 |
| FimH | 1107 | A1WO_01359 | EOW11766 | fimbrial protein FimH | Escherichia coli KTE102 | 807 | 4.10E-239 |
| FimH | 1108 | AD26_4642 | KDX37699 | protein fimH | Escherichia coli 2-156-04_S4_C3 | 807 | 4.10E-239 |
| FimH | 1109 | AC98_4757 | KDX53979 | protein fimH | Escherichia coli 2-210-07_S4_C2 | 807 | 4.10E-239 |
| FimH | 1110 | AC71_4568 | KEM85270 | protein fimH | Escherichia coli 2-222-05_S4_C1 | 807 | 4.10E-239 |
| FimH | 1111 | AD28_4472 | KDX61189 | protein fimH | Escherichia coli 2-210-07_S4_C3 | 807 | 4.10E-239 |
| FimH | 1112 | AD24_4595 | KDT12174 | protein fimH | Escherichia coli 2-011-08_S4_C3 | 807 | 4.10E-239 |
| FimH | 1113 | AB60_4826 | KDV96363 | protein fimH | Escherichia coli 2-156-04_S1_C3 | 807 | 4.10E-239 |
| FimH | 1114 | AC68_4699 | KDW12852 | protein fimH | Escherichia coli 2-156-04_S4_C1 | 807 | 4.10E-239 |
| FimH | 1115 | AC64_4806 | KEM79687 | protein fimH | Escherichia coli 6-537-08_S3_C3 | 807 | 4.10E-239 |
| FimH | 1116 | AC39_4867 | KEN13311 | protein fimH | Escherichia coli 6-537-08_S3_C2 | 807 | 4.10E-239 |
| FimH | 1117 | AC11_4761 | KEM71262 | protein fimH | Escherichia coli 6-537-08_S3_C1 | 807 | 4.10E-239 |
| FimH | 1118 | ERS139222_00714 | CTV92880 | FimH protein | Escherichia coli (GCA_001283605) | 807 | 4.10E-239 |
| FimH | 1119 | ESOG_00106 | EHO03068 | fimH | Escherichia coli E101 | 807 | 4.10E-239 |
| FimH | 1120 | AD21_4748 | KEM25292 | protein fimH | Escherichia coli 6-319-05_S4_C2 | 807 | 4.10E-239 |
| FimH | 1121 | AD47_4836 | KEM54981 | protein fimH | Escherichia coli 6-319-05_S4_C3 | 807 | 4.10E-239 |
| FimH | 1122 | HMPREF9349_04258 | EGU95831 | protein FimH | Escherichia coli MS 79-10 | 807 | 4.10E-239 |
| FimH | 1123 | HMPREF9345_03561 | EFK49949 | protein FimH | Escherichia coli MS 107-1 | 807 | 4.10E-239 |
| FimH | 1124 | ERLG_03383 | EGB51161 | FimH protein | Escherichia coli H263 | 807 | 4.10E-239 |
| FimH | 1125 | HMPREF9539_03151 | EFU46323 | protein FimH | Escherichia coli MS 110-3 | 807 | 4.10E-239 |
| FimH | 1126 | HMPREF1594_01816 | ESC98953 | protein FimH | Escherichia coli 907446 | 807 | 4.10E-239 |
| FimH | 1127 | G734_04761 | EQP29551 | protein fimH | Escherichia coli HVH 68 (4-0888028) | 807 | 4.20E-239 |
| FimH | 1128 | G855_04539 | EQU29471 | protein fimH | Escherichia coli HVH 203 (4-3126218) | 807 | 4.20E-239 |
| FimH | 1129 | DR76_2431 | AIL14233 | protein fimH | Escherichia coli ATCC 25922 | 807 | 4.20E-239 |
| FimH | 1130 | HMPREF1615_00172 | ESE12290 | protein FimH | Escherichia coli 908632 | 807 | 4.20E-239 |
| FimH | 1131 | AB34_5291 | KEO35355 | protein fimH | Escherichia coli 2-460-02_S1_C2 | 807 | 4.20E-239 |
| FimH | 1132 | A1W7_00274 | ELE93788 | protein fimH | Escherichia coli KTE87 | 807 | 4.20E-239 |
| FimH | 1133 | ECHM605_02290 | EIL82349 | adhesin | Escherichia coli HM605 | 807 | 4.20E-239 |
| FimH | 1134 | HMPREF1614_04610 | ESD94182 | protein FimH | Escherichia coli 908624 | 807 | 4.20E-239 |
| FimH | 1135 | L475_04781 | ESL31881 | protein fimH | Escherichia coli BIDMC 38 | 807 | 4.20E-239 |
| FimH | 1136 | EC958_0051 | CDN85268 | type 1 fimbriae adhesin FimH | Escherichia coli O25b:H4-ST131 | 807 | 4.20E-239 |
| FimH | 1137 | L456_04794 | ETX87707 | protein fimH | Escherichia coli BIDMC 20B | 807 | 4.20E-239 |
| FimH | 1138 | A17Q_04743 | EOX03377 | fimbrial protein FimH | Escherichia coli KTE226 | 807 | 4.20E-239 |
| FimH | 1139 | BFF50_22075 | ODG79008 | fimbrial protein | Shigella sp. FC2928 | 807 | 4.20E-239 |
| FimH | 1140 | G838_04314 | EQT51896 | protein fimH | Escherichia coli HVH 186 (4-3405044) | 807 | 4.20E-239 |
| FimH | 1141 | HMPREF1593_03574 | ESC95011 | protein FimH | Escherichia coli 907391 | 807 | 4.20E-239 |
| FimH | 1142 | AL500_20675 | KTK65102 | fimbrial protein | Escherichia coli | 807 | 4.20E-239 |
| FimH | 1143 | G941_04674 | EQX95578 | protein fimH | Escherichia coli UMEA 3206-1 | 807 | 4.20E-239 |
| FimH | 1144 | G685_00713 | EQN23180 | protein fimH | Escherichia coli HVH 5 (4-7148410) | 807 | 4.20E-239 |
| FimH | 1145 | HMPREF1601_01780 | ESA90733 | protein FimH | Escherichia coli 907779 | 807 | 4.20E-239 |
| FimH | 1146 | G927_04631 | EQX28043 | protein fimH | Escherichia coli UMEA 3172-1 | 807 | 4.20E-239 |
| FimH | 1147 | AE33_04528 | KDF70482 | protein fimH | Escherichia coli BIDMC 58 | 807 | 4.20E-239 |
| FimH | 1148 | ECOLIN_24025 | AID81493 | fimbrial protein | Escherichia coli Nissle 1917 | 807 | 4.20E-239 |
| FimH | 1149 | G800_04599 | EQS11028 | protein fimH | Escherichia coli HVH 142 (4-5627451) | 807 | 4.20E-239 |
| FimH | 1150 | A171_04113 | ELD28735 | protein fimH | Escherichia coli KTE213 | 807 | 4.20E-239 |
| FimH | 1151 | A1SY_00611 | ELG19920 | fimbrial protein FimH | Escherichia coli KTE63 | 807 | 4.20E-239 |
| FimH | 1152 | A1S7_00582 | ELE01015 | protein fimH | Escherichia coli KTE49 | 807 | 4.20E-239 |
| FimH | 1153 | AE51_04134 | KDG24977 | protein fimH | Escherichia coli BIDMC 76 | 807 | 4.20E-239 |
| FimH | 1154 | AB65_5221 | KEJ36645 | protein fimH | Escherichia coli 2-460-02_S1_C3 | 807 | 4.20E-239 |
| FimH | 1155 | V412_07075 | ETE33664 | fimbrial protein FimH | Escherichia coli LAU-EC7 | 807 | 4.20E-239 |
| FimH | 1156 | AE38_04465 | KDF79917 | protein fimH | Escherichia coli BIDMC 63 | 807 | 4.20E-239 |
| FimH | 1157 | HMPREF1621_01425 | ESE36131 | protein FimH | Escherichia coli A25922R | 807 | 4.20E-239 |
| FimH | 1158 | CDK86545 | CDK86545 | mannose-specific adhesin FimH | Escherichia coli IS29 | 807 | 4.20E-239 |
| FimH | 1159 | AE45_04386 | KDF95946 | protein fimH | Escherichia coli BIDMC 70 | 807 | 4.20E-239 |
| FimH | 1160 | G696_04616 | EQN58018 | protein fimH | Escherichia coli HVH 20 (4-5865042) | 807 | 4.20E-239 |
| FimH | 1161 | WKE_00010 | ELJ18359 | fimbrial protein FimH | Escherichia coli KTE160 | 807 | 4.20E-239 |
| FimH | 1162 | AE39_03816 | KDF87825 | protein fimH | Escherichia coli BIDMC 64 | 807 | 4.20E-239 |
| FimH | 1163 | G853_04731 | EQU18642 | protein fimH | Escherichia coli HVH 201 (4-4459431) | 807 | 4.20E-239 |
| FimH | 1164 | HMPREF1597_03586 | ESD18459 | protein FimH | Escherichia coli 907701 | 807 | 4.20E-239 |
| FimH | 1165 | L411_05114 | ERO93607 | protein fimH | Escherichia coli BWH 24 | 807 | 4.20E-239 |
| FimH | 1166 | G702_04606 | EQN87826 | protein fimH | Escherichia coli HVH 26 (4-5703913) | 807 | 4.20E-239 |
| FimH | 1167 | G945_04483 | EQY17072 | protein fimH | Escherichia coli UMEA 3216-1 | 807 | 4.20E-239 |
| FimH | 1168 | G857_04829 | EQU41950 | protein fimH | Escherichia coli HVH 205 (4-3094677) | 807 | 4.20E-239 |
| FimH | 1169 | G940_04732 | EQX94618 | protein fimH | Escherichia coli UMEA 3203-1 | 807 | 4.20E-239 |
| FimH | 1170 | CDL05895 | CDL05895 | mannose-specific adhesin FimH | Escherichia coli IS35 | 807 | 4.20E-239 |
| FimH | 1171 | L455_09105 | ETX90602 | protein fimH | Escherichia coli BIDMC 20A | 807 | 4.20E-239 |
| FimH | 1172 | AE50_04506 | KDG21488 | protein fimH | Escherichia coli BIDMC 75 | 807 | 4.20E-239 |
| FimH | 1173 | AF25_04782 | KDG48321 | protein fimH | Escherichia coli CHS 69 | 807 | 4.20E-239 |
| FimH | 1174 | G990_04485 | EQZ72258 | protein fimH | Escherichia coli UMEA 3702-1 | 807 | 4.20E-239 |
| FimH | 1175 | G807_04428 | EQS33142 | protein fimH | Escherichia coli HVH 149 (4-4451880) | 807 | 4.20E-239 |
| FimH | 1176 | G746_04716 | EQP84604 | protein fimH | Escherichia coli HVH 84 (4-1021478) | 807 | 4.20E-239 |
| FimH | 1177 | WKM_04413 | ELJ20338 | fimbrial protein FimH | Escherichia coli KTE167 | 807 | 4.20E-239 |
| FimH | 1178 | WEM_01820 | EOU79649 | fimbrial protein FimH | Escherichia coli KTE27 | 807 | 4.20E-239 |
| FimH | 1179 | CR63_21725 | KKJ99660 | fimbrial protein | Escherichia coli NB8 | 807 | 4.20E-239 |
| FimH | 1180 | G692_04621 | EQN42732 | protein fimH | Escherichia coli HVH 16 (4-7649002) | 807 | 4.20E-239 |
| FimH | 1181 | L404_04797 | ETY47965 | protein fimH | Escherichia coli BWH 34 | 807 | 4.20E-239 |
| FimH | 1182 | A17E_04406 | ELD45373 | protein fimH | Escherichia coli KTE220 | 807 | 4.20E-239 |
| FimH | 1183 | WGY_04666 | ELJ77909 | fimbrial protein FimH | Escherichia coli KTE95 | 807 | 4.20E-239 |
| FimH | 1184 | ECNA114_4565 | AEG39414 | Mannose-specific adhesin FimH | Escherichia coli NA114 | 807 | 4.20E-239 |
| FimH | 1185 | A1YQ_00366 | ELG76802 | fimbrial protein FimH | Escherichia coli KTE140 | 807 | 4.20E-239 |
| FimH | 1186 | AD01_4591 | KDY41275 | protein fimH | Escherichia coli 2-427-07_S4_C2 | 807 | 4.20E-239 |
| FimH | 1187 | A1SW_00449 | ELE36897 | protein fimH | Escherichia coli KTE62 | 807 | 4.20E-239 |
| FimH | 1188 | HMPREF1618_04157 | ESE15050 | protein FimH | Escherichia coli 908691 | 807 | 4.20E-239 |
| FimH | 1189 | HMPREF1596_02541 | ESD11602 | protein FimH | Escherichia coli 907700 | 807 | 4.20E-239 |
| FimH | 1190 | A17K_00426 | ELH94126 | fimbrial protein FimH | Escherichia coli KTE223 | 807 | 4.20E-239 |
| FimH | 1191 | AF24_03976 | KDG47799 | protein fimH | Escherichia coli CHS 68 | 807 | 4.20E-239 |
| FimH | 1192 | G708_04667 | EQO17953 | protein fimH | Escherichia coli HVH 32 (4-3773988) | 807 | 4.20E-239 |
| FimH | 1193 | AE54_03984 | KDG42472 | protein fimH | Escherichia coli BIDMC 79 | 807 | 4.20E-239 |
| FimH | 1194 | G984_04779 | EQZ48780 | protein fimH | Escherichia coli UMEA 3662-1 | 807 | 4.20E-239 |
| FimH | 1195 | AE53_02599 | KDG37319 | protein fimH | Escherichia coli BIDMC 78 | 807 | 4.20E-239 |
| FimH | 1196 | P423_24570 | AGY86963 | fimbrial protein FimH | Escherichia coli JJ1886 | 807 | 4.20E-239 |
| FimH | 1197 | AE37_04316 | KDF81708 | protein fimH | Escherichia coli BIDMC 62 | 807 | 4.20E-239 |
| FimH | 1198 | WKQ_00009 | ELJ45711 | fimbrial protein FimH | Escherichia coli KTE174 | 807 | 4.20E-239 |
| FimH | 1199 | AC95_4864 | KDV78006 | protein fimH | Escherichia coli 2-052-05_S4_C2 | 807 | 4.20E-239 |
| FimH | 1200 | AE10_04574 | KDG67515 | protein fimH | Escherichia coli UCI 51 | 807 | 4.20E-239 |
| FimH | 1201 | V411_26220 | ETE09721 | fimbrial protein FimH | Escherichia coli LAU-EC6 | 807 | 4.20E-239 |
| FimH | 1202 | G763_00444 | EQQ43930 | protein fimH | Escherichia coli HVH 102 (4-6906788) | 807 | 4.20E-239 |
| FimH | 1203 | G830_04544 | EQT17813 | protein fimH | Escherichia coli HVH 176 (4-3428664) | 807 | 4.20E-239 |
| FimH | 1204 | HMPREF1607_02893 | ESD56763 | protein FimH | Escherichia coli 908524 | 807 | 4.20E-239 |
| FimH | 1205 | HMPREF1620_02072 | ESA95880 | protein FimH | Escherichia coli 909945-2 | 807 | 4.20E-239 |
| FimH | 1206 | WEO_04656 | ELC45414 | protein fimH | Escherichia coli KTE28 | 806.9 | 4.50E-239 |
| FimH | 1207 | A1UI_04675 | EOV73210 | fimbrial protein FimH | Escherichia coli KTE73 | 806.8 | 4.60E-239 |
| FimH | 1208 | G931_04480 | EQX50470 | protein fimH | Escherichia coli UMEA 3176-1 | 806.8 | 4.60E-239 |
| FimH | 1209 | ECP030186711_4988 | ENC87495 | protein fimH | Escherichia coli P0301867.11 | 806.8 | 4.70E-239 |
| FimH | 1210 | AB52_4911 | KEN41180 | protein fimH | Escherichia coli 6-537-08_S1_C2 | 806.8 | 4.70E-239 |
| FimH | 1211 | ECP03018672_4957 | ENA40933 | protein fimH | Escherichia coli P0301867.2 | 806.8 | 4.70E-239 |
| FimH | 1212 | A17G_00032 | EOV45864 | fimbrial protein FimH | Escherichia coli KTE221 | 806.8 | 4.70E-239 |
| FimH | 1213 | AB81_4986 | KEN49312 | protein fimH | Escherichia coli 6-537-08_S1_C3 | 806.8 | 4.70E-239 |
| FimH | 1214 | BX68_04290 | EYZ00084 | fimbrial protein | Escherichia coli O177:NM str. 2010C-4558 | 806.8 | 4.70E-239 |
| FimH | 1215 | ECP03018678_4738 | ENC85876 | protein fimH | Escherichia coli P0301867.8 | 806.8 | 4.70E-239 |
| FimH | 1216 | AB24_5056 | KEM36352 | protein fimH | Escherichia coli 6-537-08_S1_C1 | 806.8 | 4.70E-239 |
| FimH | 1217 | G897_04349 | EQW07891 | protein fimH | Escherichia coli KOEGE 131 (358a) | 806.8 | 4.70E-239 |
| FimH | 1218 | EC2719100_0062 | EMX91825 | protein fimH | Escherichia coli 2719100 | 806.8 | 4.70E-239 |
| FimH | 1219 | ECP03018677_5008 | ENH04215 | protein fimH | Escherichia coli P0301867.7 | 806.8 | 4.70E-239 |
| FimH | 1220 | WKG_04668 | ELJ07784 | fimbrial protein FimH | Escherichia coli KTE163 | 806.8 | 4.70E-239 |
| FimH | 1221 | EC178900_4795 | ENA54430 | protein fimH | Escherichia coli 178900 | 806.8 | 4.70E-239 |
| FimH | 1222 | ECP030186713_5112 | END86274 | protein fimH | Escherichia coli P0301867.13 | 806.8 | 4.70E-239 |
| FimH | 1223 | AB86_4914 | KDW11735 | protein fimH | Escherichia coli 2-177-06_S3_C1 | 806.8 | 4.70E-239 |
| FimH | 1224 | AM264_18845 | KQJ03372 | fimbrial protein | Escherichia coli | 806.8 | 4.70E-239 |
| FimH | 1225 | ECP03018671_5299 | EMX15073 | protein fimH | Escherichia coli P0301867.1 | 806.8 | 4.70E-239 |
| FimH | 1226 | ECP03018673_4961 | ENG93191 | protein fimH | Escherichia coli P0301867.3 | 806.8 | 4.70E-239 |
| FimH | 1227 | G994_04618 | EQZ94027 | protein fimH | Escherichia coli UMEA 3718-1 | 806.8 | 4.80E-239 |
| FimH | 1228 | ECDEC5E_5467 | EHV41955 | protein fimH | Escherichia coli DEC5E | 806.7 | 5.10E-239 |
| FimH | 1229 | G823_04748 | EQS83509 | protein fimH | Escherichia coli HVH 167 (4-6073565) | 806.7 | 5.10E-239 |
| FimH | 1230 | ECP02999171_0051 | ENA10868 | protein fimH | Escherichia coli P0299917.1 | 806.7 | 5.10E-239 |
| FimH | 1231 | G852_04873 | EQU18344 | protein fimH | Escherichia coli HVH 200 (4-4449924) | 806.7 | 5.10E-239 |
| FimH | 1232 | G788_04813 | EQR53849 | protein fimH | Escherichia coli HVH 128 (4-7030436) | 806.7 | 5.10E-239 |
| FimH | 1233 | AC34_4566 | KDU07658 | protein fimH | Escherichia coli 3-373-03_S3_C2 | 806.7 | 5.10E-239 |
| FimH | 1234 | HQ24_22230 | AIF39564 | fimbrial protein | Escherichia coli KLY | 806.7 | 5.10E-239 |
| FimH | 1235 | P810_03286 | ETY55492 | protein fimH | Escherichia coli BIDMC 49a | 806.7 | 5.10E-239 |
| FimH | 1236 | A31C_00501 | ELH03250 | fimbrial protein FimH | Escherichia coli KTE158 | 806.7 | 5.10E-239 |
| FimH | 1237 | AD13_5000 | KDZ36770 | protein fimH | Escherichia coli 3-020-07_S4_C2 | 806.7 | 5.10E-239 |
| FimH | 1238 | A139_04430 | ELC68649 | protein fimH | Escherichia coli KTE181 | 806.7 | 5.10E-239 |
| FimH | 1239 | ECDEC15C_5068 | EHY01777 | minor fimbrial subunit, D-mannose specific adhesin | Escherichia coli DEC15C | 806.7 | 5.10E-239 |
| FimH | 1240 | EC3006_4961 | EKI32774 | protein fimH | Escherichia coli 3006 | 806.7 | 5.10E-239 |
| FimH | 1241 | A1U1_04600 | EOV52261 | fimbrial protein FimH | Escherichia coli KTE64 | 806.7 | 5.10E-239 |
| FimH | 1242 | A319_00334 | EOW64293 | fimbrial protein FimH | Escherichia coli KTE155 | 806.7 | 5.10E-239 |
| FimH | 1243 | A13A_04996 | EOW90162 | fimbrial protein FimH | Escherichia coli KTE182 | 806.7 | 5.10E-239 |
| FimH | 1244 | G789_04716 | EQR56318 | protein fimH | Escherichia coli HVH 130 (4-7036876) | 806.7 | 5.10E-239 |
| FimH | 1245 | G698_04639 | EQN74782 | protein fimH | Escherichia coli HVH 22 (4-2258986) | 806.7 | 5.10E-239 |
| FimH | 1246 | A15Q_04928 | ELD16703 | protein fimH | Escherichia coli KTE208 | 806.7 | 5.10E-239 |
| FimH | 1247 | WCA_00525 | ELC04746 | protein fimH | Escherichia coli KTE2 | 806.7 | 5.10E-239 |
| FimH | 1248 | A17O_01134 | EOX13535 | fimbrial protein FimH | Escherichia coli KTE225 | 806.7 | 5.10E-239 |
| FimH | 1249 | G695_04688 | EQN65041 | protein fimH | Escherichia coli HVH 19 (4-7154984) | 806.7 | 5.10E-239 |
| FimH | 1250 | A193_00585 | ELD74373 | protein fimH | Escherichia coli KTE234 | 806.7 | 5.10E-239 |
| FimH | 1251 | IAM_13784 | EGV47105 | minor component of type 1 fimbriae | Escherichia coli XH001 | 806.7 | 5.10E-239 |
| FimH | 1252 | WAU_00559 | EOU41311 | fimbrial protein FimH | Escherichia coli KTE3 | 806.7 | 5.10E-239 |
| FimH | 1253 | A31A_00664 | ELF23255 | protein fimH | Escherichia coli KTE156 | 806.7 | 5.10E-239 |
| FimH | 1254 | AC59_4546 | KDT71107 | protein fimH | Escherichia coli 3-373-03_S3_C3 | 806.7 | 5.10E-239 |
| FimH | 1255 | ECDEC15A_5306 | EHX92022 | minor fimbrial subunit, D-mannose specific adhesin | Escherichia coli DEC15A | 806.7 | 5.10E-239 |
| FimH | 1256 | G771_04832 | EQQ61855 | protein fimH | Escherichia coli HVH 110 (4-6978754) | 806.7 | 5.10E-239 |
| FimH | 1257 | L444_09140 | ETY20387 | protein fimH | Escherichia coli BIDMC 15 | 806.7 | 5.10E-239 |
| FimH | 1258 | A155_00589 | ELH54113 | fimbrial protein FimH | Escherichia coli KTE197 | 806.7 | 5.10E-239 |
| FimH | 1259 | G765_05048 | EQQ45654 | protein fimH | Escherichia coli HVH 104 (4-6977960) | 806.7 | 5.10E-239 |
| FimH | 1260 | WIS_04724 | ELI51554 | fimbrial protein FimH | Escherichia coli KTE129 | 806.7 | 5.10E-239 |
| FimH | 1261 | WEK_00147 | ELC51178 | protein fimH | Escherichia coli KTE26 | 806.7 | 5.10E-239 |
| FimH | 1262 | AC72_4734 | KDY08103 | protein fimH | Escherichia coli 2-316-03_S4_C1 | 806.7 | 5.10E-239 |
| FimH | 1263 | A1WK_00583 | EOW12362 | fimbrial protein FimH | Escherichia coli KTE100 | 806.7 | 5.10E-239 |
| FimH | 1264 | G798_04759 | EQR98241 | protein fimH | Escherichia coli HVH 140 (4-5894387) | 806.7 | 5.10E-239 |
| FimH | 1265 | L453_09027 | ETX96663 | protein fimH | Escherichia coli BIDMC 19B | 806.7 | 5.10E-239 |
| FimH | 1266 | A1SA_00495 | ELE06263 | protein fimH | Escherichia coli KTE51 | 806.7 | 5.10E-239 |
| FimH | 1267 | P804_03288 | ETX76952 | protein fimH | Escherichia coli BIDMC 43b | 806.7 | 5.10E-239 |
| FimH | 1268 | WI7_04517 | ELI02050 | fimbrial protein FimH | Escherichia coli KTE105 | 806.7 | 5.10E-239 |
| FimH | 1269 | WC9_04686 | EOU44759 | fimbrial protein FimH | Escherichia coli KTE231 | 806.7 | 5.10E-239 |
| FimH | 1270 | A15A_00195 | EOV27219 | fimbrial protein FimH | Escherichia coli KTE200 | 806.7 | 5.10E-239 |
| FimH | 1271 | P811_03277 | ETY52643 | protein fimH | Escherichia coli BIDMC 49b | 806.7 | 5.10E-239 |
| FimH | 1272 | G929_04679 | EQX46307 | protein fimH | Escherichia coli UMEA 3174-1 | 806.7 | 5.10E-239 |
| FimH | 1273 | G689_04593 | EQN36956 | protein fimH | Escherichia coli HVH 10 (4-6832164) | 806.7 | 5.10E-239 |
| FimH | 1274 | A17I_01816 | EOV39918 | fimbrial protein FimH | Escherichia coli KTE222 | 806.7 | 5.10E-239 |
| FimH | 1275 | G821_04866 | EQS73510 | protein fimH | Escherichia coli HVH 163 (4-4697553) | 806.7 | 5.10E-239 |
| FimH | 1276 | BWG_4018 | ACR63775 | minor component of type 1 fimbriae | Escherichia coli BW2952 | 806.7 | 5.10E-239 |
| FimH | 1277 | L428_08946 | ETY35378 | protein fimH | Escherichia coli BIDMC 2B | 806.7 | 5.10E-239 |
| FimH | 1278 | BW25113_4320 | AIN34594 | minor component of type 1 fimbriae | Escherichia coli BW25113 | 806.7 | 5.10E-239 |
| FimH | 1279 | G860_04859 | EQU56247 | protein fimH | Escherichia coli HVH 208 (4-3112292) | 806.7 | 5.10E-239 |
| FimH | 1280 | G747_04454 | EQP87843 | protein fimH | Escherichia coli HVH 85 (4-0792144) | 806.7 | 5.10E-239 |
| FimH | 1281 | b4320 | AAC77276 | minor component of type 1 fimbriae | Escherichia coli str. K-12 substr. MG1655 (GCA_000801205) | 806.7 | 5.10E-239 |
| FimH | 1282 | G865_04979 | EQU75561 | protein fimH | Escherichia coli HVH 213 (4-3042928) | 806.7 | 5.10E-239 |
| FimH | 1283 | G735_04628 | EQP30902 | protein fimH | Escherichia coli HVH 69 (4-2837072) | 806.7 | 5.10E-239 |
| FimH | 1284 | L474_04418 | ESL31368 | protein fimH | Escherichia coli BIDMC 37 | 806.7 | 5.10E-239 |
| FimH | 1285 | G895_04780 | EQV95302 | protein fimH | Escherichia coli KOEGE 77 (202a) | 806.7 | 5.10E-239 |
| FimH | 1286 | AB69_4958 | EYE31282 | protein fimH | Escherichia coli 1-110-08_S1_C3 | 806.7 | 5.10E-239 |
| FimH | 1287 | A17U_03576 | ELD56445 | protein fimH | Escherichia coli KTE228 | 806.7 | 5.10E-239 |
| FimH | 1288 | B185_025475 | ELL39109 | type 1 fimbrial adhesin FimH | Escherichia coli J96 | 806.7 | 5.10E-239 |
| FimH | 1289 | IAE_10094 | EGU27035 | minor component of type 1 fimbriae | Escherichia coli XH140A | 806.7 | 5.10E-239 |
| FimH | 1290 | L452_05240 | ETY08706 | protein fimH | Escherichia coli BIDMC 19A | 806.7 | 5.10E-239 |
| FimH | 1291 | G813_04744 | ERA63177 | protein fimH | Escherichia coli HVH 155 (4-4509048) | 806.7 | 5.10E-239 |
| FimH | 1292 | AE52_03246 | KDG33750 | protein fimH | Escherichia coli BIDMC 77 | 806.7 | 5.10E-239 |
| FimH | 1293 | G883_04510 | EQV39960 | protein fimH | Escherichia coli KOEGE 33 (68a) | 806.7 | 5.10E-239 |
| FimH | 1294 | G891_04596 | EQV75294 | protein fimH | Escherichia coli KOEGE 68 (182a) | 806.7 | 5.10E-239 |
| FimH | 1295 | WAS_00529 | EOX00059 | fimbrial protein FimH | Escherichia coli KTE1 | 806.7 | 5.10E-239 |
| FimH | 1296 | EC180050_0304 | EMW90798 | protein fimH | Escherichia coli 180050 | 806.7 | 5.10E-239 |
| FimH | 1297 | G784_04618 | EQR29810 | protein fimH | Escherichia coli HVH 122 (4-6851606) | 806.7 | 5.10E-239 |
| FimH | 1298 | BAE78313 | BAE78313 | minor component of type 1 fimbriae | Escherichia coli str. K-12 substr. W3110 | 806.7 | 5.10E-239 |
| FimH | 1299 | P803_04627 | ETX82102 | protein fimH | Escherichia coli BIDMC 43a | 806.7 | 5.10E-239 |
| FimH | 1300 | EH66_19670 | KEO95250 | fimbrial protein | Escherichia coli HVH 70 (4-2963531) | 806.7 | 5.10E-239 |
| FimH | 1301 | G732_04727 | EQP20030 | protein fimH | Escherichia coli HVH 63 (4-2542528) | 806.7 | 5.10E-239 |
| FimH | 1302 | SU67_18595 | KIO39383 | fimbrial protein | Escherichia coli O139:H28 str. E24377A (GCA_000832005) | 806.7 | 5.10E-239 |
| FimH | 1303 | G882_04590 | EQV36558 | protein fimH | Escherichia coli KOEGE 32 (66a) | 806.7 | 5.10E-239 |
| FimH | 1304 | G809_04596 | EQS43764 | protein fimH | Escherichia coli HVH 151 (4-5755573) | 806.7 | 5.10E-239 |
| FimH | 1305 | G792_04765 | EQR70507 | protein fimH | Escherichia coli HVH 134 (4-6073441) | 806.7 | 5.10E-239 |
| FimH | 1306 | AF55_03226 | EZQ66580 | protein fimH | Escherichia coli BIDMC 82 | 806.7 | 5.10E-239 |
| FimH | 1307 | G874_04779 | EQV16310 | protein fimH | Escherichia coli HVH 223 (4-2976528) | 806.7 | 5.10E-239 |
| FimH | 1308 | A15I_04654 | ELD00678 | protein fimH | Escherichia coli KTE204 | 806.7 | 5.10E-239 |
| FimH | 1309 | WIQ_04635 | ELI49498 | fimbrial protein FimH | Escherichia coli KTE128 | 806.7 | 5.10E-239 |
| FimH | 1310 | ECVR50_4786 | AKA93478 | protein FimH | Escherichia coli VR50 | 806.7 | 5.10E-239 |
| FimH | 1311 | G774_04777 | EQQ82421 | protein fimH | Escherichia coli HVH 113 (4-7535473) | 806.7 | 5.10E-239 |
| FimH | 1312 | ECDEC15B_5187 | EHX98954 | minor fimbrial subunit, D-mannose specific adhesin | Escherichia coli DEC15B | 806.7 | 5.10E-239 |
| FimH | 1313 | AB10_4755 | EYE31743 | protein fimH | Escherichia coli 1-110-08_S1_C1 | 806.7 | 5.10E-239 |
| FimH | 1314 | A1YE_00640 | EOW43068 | fimbrial protein FimH | Escherichia coli KTE127 | 806.7 | 5.10E-239 |
| FimH | 1315 | A1UW_04765 | ELE66185 | protein fimH | Escherichia coli KTE80 | 806.7 | 5.10E-239 |
| FimH | 1316 | N840_4403 | AGX36214 | minor component of type 1 fimbriae | synthetic Escherichia coli C321.deltaA | 806.7 | 5.10E-239 |
| FimH | 1317 | ECGG_03269 | EFF02905 | fimH | Escherichia coli FVEC1412 | 806.7 | 5.10E-239 |
| FimH | 1318 | A195_04382 | ELD74185 | protein fimH | Escherichia coli KTE235 | 806.7 | 5.10E-239 |
| FimH | 1319 | ECP029970676_5112 | ENC26701 | protein fimH | Escherichia coli P02997067.6 | 806.7 | 5.10E-239 |
| FimH | 1320 | A317_02449 | ELG97935 | fimbrial protein FimH | Escherichia coli KTE154 | 806.7 | 5.10E-239 |
| FimH | 1321 | G736_04975 | EQP42919 | protein fimH | Escherichia coli HVH 70 (4-2963531) | 806.7 | 5.10E-239 |
| FimH | 1322 | A31O_00468 | EOW72650 | fimbrial protein FimH | Escherichia coli KTE170 | 806.7 | 5.10E-239 |
| FimH | 1323 | A13Q_00339 | ELH29756 | fimbrial protein FimH | Escherichia coli KTE190 | 806.7 | 5.10E-239 |
| FimH | 1324 | AD14_3408 | KDZ73666 | protein fimH | Escherichia coli 3-073-06_S4_C2 | 806.7 | 5.10E-239 |
| FimH | 1325 | G894_04457 | EQV93961 | protein fimH | Escherichia coli KOEGE 73 (195a) | 806.7 | 5.10E-239 |
| FimH | 1326 | G915_04344 | EQW93093 | protein fimH | Escherichia coli UMEA 3140-1 | 806.7 | 5.10E-239 |
| FimH | 1327 | AD06_0008 | KEN16711 | protein fimH | Escherichia coli 7-233-03_S4_C2 | 806.7 | 5.10E-239 |
| FimH | 1328 | G806_01565 | ESP34418 | protein fimH | Escherichia coli HVH 148 (4-3192490) | 806.7 | 5.10E-239 |
| FimH | 1329 | AD30_4491 | KDY15238 | protein fimH | Escherichia coli 2-316-03_S4_C3 | 806.7 | 5.10E-239 |
| FimH | 1330 | WKI_00032 | ELJ30943 | fimbrial protein FimH | Escherichia coli KTE166 | 806.7 | 5.10E-239 |
| FimH | 1331 | G967_04582 | EQY93840 | protein fimH | Escherichia coli UMEA 3329-1 | 806.7 | 5.10E-239 |
| FimH | 1332 | L454_04740 | ERO99460 | protein fimH | Escherichia coli BIDMC 19C | 806.7 | 5.10E-239 |
| FimH | 1333 | G835_04897 | EQT33067 | protein fimH | Escherichia coli HVH 183 (4-3205932) | 806.7 | 5.10E-239 |
| FimH | 1334 | L447_04574 | ETY13672 | protein fimH | Escherichia coli BIDMC 17B | 806.7 | 5.10E-239 |
| FimH | 1335 | BN896_4018 | CDJ74537 | minor component of type 1 fimbriae | Escherichia coli str. K-12 substr. MC4100 | 806.7 | 5.10E-239 |
| FimH | 1336 | G873_04544 | EQV07680 | protein fimH | Escherichia coli HVH 222 (4-2977443) | 806.7 | 5.10E-239 |
| FimH | 1337 | ECDEC15D_5004 | EHY09589 | minor fimbrial subunit, D-mannose specific adhesin | Escherichia coli DEC15D | 806.7 | 5.10E-239 |
| FimH | 1338 | EC990741_4993 | EIH11211 | mannose-binding domain protein FimH | Escherichia coli 97.0259 | 806.7 | 5.10E-239 |
| FimH | 1339 | AB99_4817 | EZJ86796 | protein fimH | Escherichia coli 1-182-04_S3_C1 | 806.7 | 5.10E-239 |
| FimH | 1340 | PCN061_4505 | AKM37936 | minor component of type 1 fimbriae | Escherichia coli PCN061 | 806.7 | 5.10E-239 |
| FimH | 1341 | G828_04754 | EQT06495 | protein fimH | Escherichia coli HVH 173 (3-9175482) | 806.7 | 5.10E-239 |
| FimH | 1342 | AF44_03476 | KDG67739 | protein fimH | Escherichia coli MGH 58 | 806.7 | 5.10E-239 |
| FimH | 1343 | ECCZ_01840 | EST65314 | minor component of type 1 fimbriae | Escherichia coli ECC-Z | 806.7 | 5.10E-239 |
| FimH | 1344 | WKW_04674 | ELJ46791 | fimbrial protein FimH | Escherichia coli KTE179 | 806.7 | 5.10E-239 |
| FimH | 1345 | L408_00433 | ETY51036 | protein fimH | Escherichia coli BWH 40 | 806.7 | 5.10E-239 |
| FimH | 1346 | G808_04469 | EQS44529 | protein fimH | Escherichia coli HVH 150 (4-3258106) | 806.7 | 5.10E-239 |
| FimH | 1347 | G793_04691 | EQR73364 | protein fimH | Escherichia coli HVH 135 (4-4449320) | 806.7 | 5.10E-239 |
| FimH | 1348 | L670_08839 | KGL70395 | type 1 fimbrial adhesin FimH | Escherichia coli NCTC 50110 | 806.7 | 5.10E-239 |
| FimH | 1349 | L432_08804 | ETY61284 | protein fimH | Escherichia coli BIDMC 6 | 806.7 | 5.10E-239 |
| FimH | 1350 | G939_03661 | EQX93230 | protein fimH | Escherichia coli UMEA 3201-1 | 806.7 | 5.10E-239 |
| FimH | 1351 | A19A_00286 | EOX16873 | fimbrial protein FimH | Escherichia coli KTE240 | 806.7 | 5.10E-239 |
| FimH | 1352 | A1YK_00096 | EOW60890 | fimbrial protein FimH | Escherichia coli KTE134 | 806.7 | 5.10E-239 |
| FimH | 1353 | L446_09028 | ETY13465 | protein fimH | Escherichia coli BIDMC 17A | 806.7 | 5.10E-239 |
| FimH | 1354 | A1SK_02385 | ELE13880 | protein fimH | Escherichia coli KTE56 | 806.7 | 5.10E-239 |
| FimH | 1355 | ERKG_02918 | EGB46468 | FimH protein | Escherichia coli H252 | 806.7 | 5.10E-239 |
| FimH | 1356 | A1W1_04843 | ELE76388 | protein fimH | Escherichia coli KTE83 | 806.7 | 5.10E-239 |
| FimH | 1357 | G791_04738 | EQR69087 | protein fimH | Escherichia coli HVH 133 (4-4466519) | 806.7 | 5.10E-239 |
| FimH | 1358 | WGI_00375 | ELC65340 | protein fimH | Escherichia coli KTE44 | 806.7 | 5.10E-239 |
| FimH | 1359 | G875_04715 | EQV20530 | protein fimH | Escherichia coli HVH 225 (4-1273116) | 806.7 | 5.10E-239 |
| FimH | 1360 | AD41_2617 | KDZ50083 | protein fimH | Escherichia coli 3-020-07_S4_C3 | 806.7 | 5.10E-239 |
| FimH | 1361 | A15K_04825 | ELD04937 | protein fimH | Escherichia coli KTE205 | 806.7 | 5.10E-239 |
| FimH | 1362 | G958_04802 | ERB24218 | protein fimH | Escherichia coli UMEA 3271-1 | 806.7 | 5.10E-239 |
| FimH | 1363 | G944_04809 | EQY08479 | protein fimH | Escherichia coli UMEA 3215-1 | 806.7 | 5.10E-239 |
| FimH | 1364 | G737_04820 | EQP45593 | protein fimH | Escherichia coli HVH 73 (4-2393174) | 806.7 | 5.10E-239 |
| FimH | 1365 | G832_04608 | ESP27353 | protein fimH | Escherichia coli HVH 178 (4-3189163) | 806.7 | 5.10E-239 |
| FimH | 1366 | G890_04975 | EQV77930 | protein fimH | Escherichia coli KOEGE 62 (175a) | 806.7 | 5.10E-239 |
| FimH | 1367 | WKU_04624 | ELJ37079 | fimbrial protein FimH | Escherichia coli KTE177 | 806.7 | 5.10E-239 |
| FimH | 1368 | G730_04646 | EQP09554 | protein fimH | Escherichia coli HVH 59 (4-1119338) | 806.7 | 5.10E-239 |
| FimH | 1369 | SH05_03260 | AKD59517 | fimbrial protein | Escherichia coli (strain K12) | 806.7 | 5.10E-239 |
| FimH | 1370 | EC5761_17109 | EIL65020 | minor component of type 1 fimbriae | Escherichia coli 576-1 | 806.7 | 5.10E-239 |
| FimH | 1371 | G867_04852 | EQU82042 | protein fimH | Escherichia coli HVH 215 (4-3008371) | 806.7 | 5.10E-239 |
| FimH | 1372 | G750_04680 | EQP98733 | protein fimH | Escherichia coli HVH 88 (4-5854636) | 806.7 | 5.10E-239 |
| FimH | 1373 | A17W_03408 | ELH96325 | fimbrial protein FimH | Escherichia coli KTE229 | 806.7 | 5.10E-239 |
| FimH | 1374 | G733_04585 | EQP27811 | protein fimH | Escherichia coli HVH 65 (4-2262045) | 806.7 | 5.10E-239 |
| FimH | 1375 | G952_04896 | EQY50240 | protein fimH | Escherichia coli UMEA 3240-1 | 806.7 | 5.10E-239 |
| FimH | 1376 | G803_04923 | EQS19596 | protein fimH | Escherichia coli HVH 145 (4-5672112) | 806.7 | 5.10E-239 |
| FimH | 1377 | ECDH1ME8569_4178 | BAJ46034 | type 1 fimbrial adhesin FimH | Escherichia coli DH1 | 806.7 | 5.10E-239 |
| FimH | 1378 | G434_03631 | EOW71237 | fimbrial protein FimH | Escherichia sp. KTE172 | 806.7 | 5.10E-239 |
| FimH | 1379 | ESSG_00223 | EIG50733 | protein fimH | Escherichia coli H730 | 806.7 | 5.10E-239 |
| FimH | 1380 | G768_04920 | EQQ62547 | protein fimH | Escherichia coli HVH 107 (4-5860571) | 806.7 | 5.10E-239 |
| FimH | 1381 | L436_08809 | ETY23979 | protein fimH | Escherichia coli BIDMC 9 | 806.7 | 5.10E-239 |
| FimH | 1382 | A1Y3_00662 | ELF04244 | protein fimH | Escherichia coli KTE116 | 806.7 | 5.10E-239 |
| FimH | 1383 | G705_04742 | EQO01888 | protein fimH | Escherichia coli HVH 29 (4-3418073) | 806.7 | 5.10E-239 |
| FimH | 1384 | WG3_00198 | EOU96445 | fimbrial protein FimH | Escherichia coli KTE36 | 806.7 | 5.10E-239 |
| FimH | 1385 | G858_04775 | EQU46082 | protein fimH | Escherichia coli HVH 206 (4-3128229) | 806.7 | 5.10E-239 |
| FimH | 1386 | G767_04795 | EQQ54014 | protein fimH | Escherichia coli HVH 106 (4-6881831) | 806.7 | 5.10E-239 |
| FimH | 1387 | G972_04700 | EQZ08508 | protein fimH | Escherichia coli UMEA 3355-1 | 806.7 | 5.10E-239 |
| FimH | 1388 | G906_05088 | EQW54031 | protein fimH | Escherichia coli UMEA 3088-1 | 806.7 | 5.10E-239 |
| FimH | 1389 | WCC_00161 | ELC03557 | protein fimH | Escherichia coli KTE4 | 806.7 | 5.10E-239 |
| FimH | 1390 | WGO_04705 | ELJ63711 | fimbrial protein FimH | Escherichia coli KTE85 | 806.7 | 5.10E-239 |
| FimH | 1391 | G833_04634 | EQT22138 | protein fimH | Escherichia coli HVH 180 (4-3051617) | 806.7 | 5.10E-239 |
| FimH | 1392 | ECUMN_4927 | CAR16038 | minor component of type 1 fimbriae | Escherichia coli UMN026 | 806.7 | 5.10E-239 |
| FimH | 1393 | G781_04800 | EQR15626 | protein fimH | Escherichia coli HVH 119 (4-6879578) | 806.7 | 5.10E-239 |
| FimH | 1394 | WGM_04859 | ELJ61291 | fimbrial protein FimH | Escherichia coli KTE82 | 806.7 | 5.10E-239 |
| FimH | 1395 | ECFG_03462 | EFI22339 | mannose-specific adhesin FimH | Escherichia coli FVEC1302 | 806.7 | 5.10E-239 |
| FimH | 1396 | L429_09119 | ETY29990 | protein fimH | Escherichia coli BIDMC 3 | 806.7 | 5.10E-239 |
| FimH | 1397 | G901_04570 | EQW26676 | protein fimH | Escherichia coli UMEA 3041-1 | 806.7 | 5.10E-239 |
| FimH | 1398 | G787_04672 | EQR49492 | protein fimH | Escherichia coli HVH 127 (4-7303629) | 806.7 | 5.10E-239 |
| FimH | 1399 | AC56_4856 | EZJ65532 | protein fimH | Escherichia coli 1-182-04_S3_C3 | 806.7 | 5.10E-239 |
| FimH | 1400 | WIK_04764 | ELI36508 | fimbrial protein FimH | Escherichia coli KTE122 | 806.7 | 5.10E-239 |
| FimH | 1401 | G841_04600 | EQT69921 | protein fimH | Escherichia coli HVH 189 (4-3220125) | 806.7 | 5.10E-239 |
| FimH | 1402 | G690_04431 | ESP12982 | protein fimH | Escherichia coli HVH 12 (4-7653042) | 806.7 | 5.10E-239 |
| FimH | 1403 | AC85_5376 | KEJ53310 | protein fimH | Escherichia coli 3-020-07_S4_C1 | 806.7 | 5.10E-239 |
| FimH | 1404 | G720_04964 | EQO74526 | protein fimH | Escherichia coli HVH 45 (4-3129918) | 806.7 | 5.10E-239 |
| FimH | 1405 | A1U9_04994 | EOV54786 | fimbrial protein FimH | Escherichia coli KTE68 | 806.7 | 5.10E-239 |
| FimH | 1406 | G744_00128 | EQP94294 | protein fimH | Escherichia coli HVH 82 (4-2209276) | 806.7 | 5.10E-239 |
| FimH | 1407 | ECDEC15E_0008 | EHY21326 | minor fimbrial subunit, D-mannose specific adhesin | Escherichia coli DEC15E | 806.7 | 5.10E-239 |
| FimH | 1408 | AA102_13895 | AKH24937 | fimbrial protein | Escherichia coli | 806.7 | 5.10E-239 |
| FimH | 1409 | BX16_16425 | EZE34085 | fimbrial protein | Escherichia coli O91:NM str. 2009C-3745 | 806.7 | 5.10E-239 |
| FimH | 1410 | EC970259_5207 | EIH46478 | mannose-binding domain protein FimH | Escherichia coli 99.0741 | 806.7 | 5.10E-239 |
| FimH | 1411 | ECEG_02933 | EFF14738 | conserved hypothetical protein | Escherichia coli B354 | 806.7 | 5.10E-239 |
| FimH | 1412 | ERS009869_03906 | CSR34683 | protein FimH | Shigella sonnei (GCA_001259515) | 806.7 | 5.10E-239 |
| FimH | 1413 | EC1303_c45120 | AJF59125 | minor component of type 1 fimbriae | Escherichia coli 1303 | 806.7 | 5.10E-239 |
| FimH | 1414 | AB70_4854 | EZK04728 | protein fimH | Escherichia coli 1-176-05_S1_C3 | 806.7 | 5.10E-239 |
| FimH | 1415 | EC2846750_4882 | EMZ59739 | protein fimH | Escherichia coli 2846750 | 806.7 | 5.10E-239 |
| FimH | 1416 | AB49_4567 | KEK90907 | protein fimH | Escherichia coli 4-203-08_S1_C2 | 806.7 | 5.10E-239 |
| FimH | 1417 | G965_04428 | EQY93504 | protein fimH | Escherichia coli UMEA 3318-1 | 806.7 | 5.10E-239 |
| FimH | 1418 | BY42_11180 | EYV92423 | fimbrial protein | Escherichia coli O6:H16 str. 99-3165 | 806.7 | 5.10E-239 |
| FimH | 1419 | AB11_4753 | EYD78656 | protein fimH | Escherichia coli 1-176-05_S1_C1 | 806.7 | 5.10E-239 |
| FimH | 1420 | ECAD30_15070 | EKJ83601 | protein FimH | Escherichia coli AD30 | 806.7 | 5.10E-239 |
| FimH | 1421 | AD25_4679 | KDV76744 | protein fimH | Escherichia coli 2-052-05_S4_C3 | 806.7 | 5.10E-239 |
| FimH | 1422 | BY44_19150 | EZA74596 | fimbrial protein | Escherichia coli O6:H16 str. F5656C1 | 806.7 | 5.10E-239 |
| FimH | 1423 | JO86_10500 | KGT31353 | fimbrial protein | Escherichia coli | 806.7 | 5.10E-239 |
| FimH | 1424 | N444_08280 | ETS27571 | fimbrial protein FimH | Escherichia coli O6:H16:CFA/II str. B2C | 806.7 | 5.10E-239 |
| FimH | 1425 | AC16_3149 | KDX44291 | protein fimH | Escherichia coli 2-177-06_S3_C2 | 806.7 | 5.10E-239 |
| FimH | 1426 | Ec53638_1471 | EDU63570 | protein FimH | Escherichia coli 53638 | 806.7 | 5.10E-239 |
| FimH | 1427 | ERCG_03416 | EGB31606 | FimH protein | Escherichia coli E1520 | 806.7 | 5.10E-239 |
| FimH | 1428 | EcolC_3743 | ACA79352 | FimH mannose-binding domain protein | Escherichia coli ATCC 8739 | 806.7 | 5.10E-239 |
| FimH | 1429 | AC44_0030 | KEO17101 | protein fimH | Escherichia coli 2-177-06_S3_C3 | 806.7 | 5.10E-239 |
| FimH | 1430 | AB78_2320 | KEK99695 | protein fimH | Escherichia coli 4-203-08_S1_C3 | 806.7 | 5.10E-239 |
| FimH | 1431 | ECDEC7B_4720 | EHV88116 | minor fimbrial subunit, D-mannose specific adhesin | Escherichia coli DEC7B | 806.7 | 5.10E-239 |
| FimH | 1432 | EC2785200_4685 | EMW30266 | protein fimH | Escherichia coli 2785200 | 806.7 | 5.10E-239 |
| FimH | 1433 | AB21_4534 | KDU59961 | protein fimH | Escherichia coli 4-203-08_S1_C1 | 806.7 | 5.10E-239 |
| FimH | 1434 | AB39_4833 | EZK13514 | protein fimH | Escherichia coli 1-176-05_S1_C2 | 806.7 | 5.10E-239 |
| FimH | 1435 | ECP03019043_4928 | END86741 | protein fimH | Escherichia coli P0301904.3 | 806.7 | 5.10E-239 |
| FimH | 1436 | EC2872000_5047 | EMV50833 | protein fimH | Escherichia coli 2872000 | 806.7 | 5.10E-239 |
| FimH | 1437 | PU63_21160 | KHH15668 | fimbrial protein | Escherichia coli (GCA_000819245) | 806.7 | 5.10E-239 |
| FimH | 1438 | WR06_21020 | KNG06100 | fimbrial protein | Escherichia coli (GCA_001191455) | 806.7 | 5.20E-239 |
| FimH | 1439 | AGA24_12040 | KNY55666 | fimbrial protein | Escherichia coli (GCA_001262865) | 806.7 | 5.20E-239 |
| FimH | 1440 | ECMP0210174_4905 | EMU86024 | protein fimH | Escherichia coli MP021017.4 | 806.7 | 5.20E-239 |
| FimH | 1441 | EC2861200_0006 | EMV91360 | protein fimH | Escherichia coli 2861200 | 806.7 | 5.20E-239 |
| FimH | 1442 | ECMP02101711_0005 | EMV14654 | protein fimH | Escherichia coli MP021017.11 | 806.7 | 5.20E-239 |
| FimH | 1443 | ECMP0210175_4934 | EMU75172 | protein fimH | Escherichia coli MP021017.5 | 806.7 | 5.20E-239 |
| FimH | 1444 | ECMP0210171_0016 | EMX43716 | protein fimH | Escherichia coli MP021017.1 | 806.7 | 5.20E-239 |
| FimH | 1445 | ECBCE008MS01_4601 | ENB05000 | protein fimH | Escherichia coli BCE008_MS-01 | 806.7 | 5.20E-239 |
| FimH | 1446 | ECMP0210173_0005 | EMV01222 | protein fimH | Escherichia coli MP021017.3 | 806.7 | 5.20E-239 |
| FimH | 1447 | ECMP02101710_0005 | EMV10833 | protein fimH | Escherichia coli MP021017.10 | 806.7 | 5.20E-239 |
| FimH | 1448 | ECMP0210176_0005 | EMU87150 | protein fimH | Escherichia coli MP021017.6 | 806.7 | 5.20E-239 |
| FimH | 1449 | ECBCE008MS13_0009 | ENA19793 | protein fimH | Escherichia coli BCE008_MS-13 | 806.7 | 5.20E-239 |
| FimH | 1450 | ECMP0210172_0005 | EMV02744 | protein fimH | Escherichia coli MP021017.2 | 806.7 | 5.20E-239 |
| FimH | 1451 | ECMP02101712_4605 | EMV11314 | protein fimH | Escherichia coli MP021017.12 | 806.7 | 5.20E-239 |
| FimH | 1452 | HMPREF1606_00958 | ESD61053 | protein FimH | Escherichia coli 908522 | 806.6 | 5.30E-239 |
| FimH | 1453 | HMPREF1599_04100 | ESA83512 | protein FimH | Escherichia coli 907713 | 806.6 | 5.30E-239 |
| FimH | 1454 | HMPREF1605_05322 | ESD45670 | protein FimH | Escherichia coli 908521 | 806.6 | 5.30E-239 |
| FimH | 1455 | PGC_10140 | CDP75633 | Putative uncharacterized protein | Escherichia coli D6-117.29 | 806.6 | 5.30E-239 |
| FimH | 1456 | HMPREF1600_02983 | ESD25683 | protein FimH | Escherichia coli 907715 | 806.6 | 5.30E-239 |
| FimH | 1457 | HMPREF9552_00195 | EFJ76111 | protein FimH | Escherichia coli MS 198-1 | 806.6 | 5.30E-239 |
| FimH | 1458 | HMPREF9530_03164 | EFK20218 | protein FimH | Escherichia coli MS 21-1 | 806.6 | 5.30E-239 |
| FimH | 1459 | HMPREF9543_03381 | EFK89801 | protein FimH | Escherichia coli MS 146-1 | 806.6 | 5.30E-239 |
| FimH | 1460 | HMPREF1612_01612 | ESD92073 | protein FimH | Escherichia coli 908585 | 806.6 | 5.30E-239 |
| FimH | 1461 | HMPREF1613_01493 | ESD92712 | protein FimH | Escherichia coli 908616 | 806.6 | 5.30E-239 |
| FimH | 1462 | OQE_35130 | EIE35473 | fimbrial protein | Escherichia coli J53 | 806.6 | 5.30E-239 |
| FimH | 1463 | AC789_1c47760 | AJE58925 | type 1 fimbiral adhesin FimH | Escherichia coli | 806.6 | 5.30E-239 |
| FimH | 1464 | HMPREF9540_00357 | EFJ99534 | protein FimH | Escherichia coli MS 115-1 | 806.6 | 5.30E-239 |
| FimH | 1465 | HMPREF1617_00768 | ESE21364 | protein FimH | Escherichia coli 908675 | 806.6 | 5.30E-239 |
| FimH | 1466 | PGA_01100 | CDU33087 | Type 1 fimbrial adhesin FimH | Escherichia coli D6-113.11 | 806.6 | 5.30E-239 |
| FimH | 1467 | HMPREF1589_00465 | ESA76437 | protein FimH | Escherichia coli 113290 | 806.6 | 5.30E-239 |
| FimH | 1468 | G893_00526 | EQV94164 | protein fimH | Escherichia coli KOEGE 71 (186a) | 806.5 | 5.80E-239 |
| FimH | 1469 | A364_00235 | EMD15425 | adhesin | Escherichia coli SEPT362 | 806.5 | 5.80E-239 |
| FimH | 1470 | ESCG_01105 | EEH72447 | protein fimH | Escherichia sp. 1_1_43 | 806.5 | 6.00E-239 |
| FimH | 1471 | AM269_17245 | KQJ28042 | fimbrial protein | Escherichia coli (GCA_001420045) | 806.4 | 6.40E-239 |
| FimH | 1472 | G759_04775 | ESJ98611 | protein fimH | Escherichia coli HVH 98 (4-5799287) | 806.4 | 6.40E-239 |
| FimH | 1473 | RT53_26060 | KHO55993 | fimbrial protein | Escherichia coli (GCA_000806195) | 806.4 | 6.40E-239 |
| FimH | 1474 | ECDEC9E_5686 | EHW48437 | minor fimbrial subunit, D-mannose specific adhesin | Escherichia coli DEC9E | 806.4 | 6.50E-239 |
| FimH | 1475 | ACU58_24715 | KPO16429 | fimbrial protein | Escherichia coli (GCA_001309535) | 806.4 | 6.50E-239 |
| FimH | 1476 | ERS009798_03310 | CSP66563 | protein FimH | Shigella sonnei (GCA_001255415) | 806.1 | 7.70E-239 |
| FimH | 1477 | ERS139207_02570 | CTW11049 | FimH protein | Escherichia coli (GCA_001285965) | 806.1 | 7.70E-239 |
| FimH | 1478 | AC35_2637 | KEK87144 | protein fimH | Escherichia coli 3-475-03_S3_C2 | 806.1 | 7.70E-239 |
| FimH | 1479 | AML35_18530 | KYS90557 | fimbrial protein | Escherichia coli | 806.1 | 7.80E-239 |
| FimH | 1480 | A1UM_00146 | ELE59502 | protein fimH | Escherichia coli KTE75 | 805.9 | 9.10E-239 |
| FimH | 1481 | ECNG_01389 | EGI42635 | protein FimH | Escherichia coli TA280 | 805.8 | 9.40E-239 |
| FimH | 1482 | ACP64_12835 | KOZ37757 | fimbrial protein | Escherichia coli (GCA_001281925) | 805.8 | 9.70E-239 |
| FimH | 1483 | EC09BKT78844_5722 | ELV15331 | protein fimH | Escherichia coli 09BKT078844 | 805.8 | 9.70E-239 |
| FimH | 1484 | HMPREF9550_01119 | EFK26741 | fimbrial protein | Escherichia coli MS 187-1 | 805.7 | 1.00E-238 |
| FimH | 1485 | EC96154_4688 | EIH98117 | mannose-binding domain protein FimH | Escherichia coli 96.154 | 805.4 | 1.20E-238 |
| FimH | 1486 | ECIAI39_4793 | CAR20890 | minor component of type 1 fimbriae | Escherichia coli IAI39 | 805.4 | 1.20E-238 |
| FimH | 1487 | ECC69171_18570 | KIG25314 | fimbrial protein | Escherichia coli C691-71 (14b) | 805.4 | 1.20E-238 |
| FimH | 1488 | LJ08_2847 | KGI48507 | mannose-specific adhesin FimH | Escherichia coli O7:K1 (strain IAI39 / ExPEC) | 805.4 | 1.20E-238 |
| FimH | 1489 | G725_04899 | EQO90981 | protein fimH | Escherichia coli HVH 53 (4-0631051) | 805.4 | 1.20E-238 |
| FimH | 1490 | CE10_5064 | AEQ15726 | minor component of type 1 fimbriae | Escherichia coli O7:K1 str. CE10 | 805.4 | 1.20E-238 |
| FimH | 1491 | H000_04153 | ERA29476 | protein fimH | Escherichia coli UMEA 3899-1 | 805.4 | 1.20E-238 |
| FimH | 1492 | G839_03857 | EQT55706 | protein fimH | Escherichia coli HVH 187 (4-4471660) | 805.4 | 1.20E-238 |
| FimH | 1493 | G801_04501 | EQS09440 | protein fimH | Escherichia coli HVH 143 (4-5674999) | 805.3 | 1.40E-238 |
| FimH | 1494 | G912_04732 | EQW76254 | protein fimH | Escherichia coli UMEA 3122-1 | 805.2 | 1.40E-238 |
| FimH | 1495 | M13_15221 | EST67439 | adhesin | Escherichia coli P4-96 | 805.1 | 1.60E-238 |
| FimH | 1496 | MOI_17809 | EST67516 | adhesin | Escherichia coli P4-NR | 805.1 | 1.60E-238 |
| FimH | 1497 | EC2730350_4622 | ENA73805 | protein fimH | Escherichia coli 2730350 | 805.1 | 1.60E-238 |
| FimH | 1498 | G697_04687 | EQN69717 | protein fimH | Escherichia coli HVH 21 (4-4517873) | 805.1 | 1.60E-238 |
| FimH | 1499 | EC2850750_4921 | EMW00356 | protein fimH | Escherichia coli 2850750 | 804.9 | 1.80E-238 |
| FimH | 1500 | ECBCE002MS12_4678 | EMV29092 | protein fimH | Escherichia coli BCE002_MS12 | 804.9 | 1.80E-238 |
| FimH | 1501 | EC2866450_4936 | EMV66497 | protein fimH | Escherichia coli 2866450 | 804.9 | 1.80E-238 |
| FimH | 1502 | ECP03048167_4883 | ENF58808 | protein fimH | Escherichia coli P0304816.7 | 804.9 | 1.80E-238 |
| FimH | 1503 | ECP02994385_4982 | ENC05268 | protein fimH | Escherichia coli P0299438.5 | 804.9 | 1.80E-238 |
| FimH | 1504 | ECP030481614_4753 | ENF29362 | protein fimH | Escherichia coli P0304816.14 | 804.9 | 1.80E-238 |
| FimH | 1505 | EC2850400_4939 | EMW13088 | protein fimH | Escherichia coli 2850400 | 804.9 | 1.80E-238 |
| FimH | 1506 | EC2875150_4902 | ENB05267 | protein fimH | Escherichia coli 2875150 | 804.9 | 1.80E-238 |
| FimH | 1507 | WES_00531 | EOU82579 | fimbrial protein FimH | Escherichia sp. KTE31 | 804.6 | 2.20E-238 |
| FimH | 1508 | WEW_02704 | EOQ54950 | fimbrial protein FimH | Escherichia coli KTE33 | 804.6 | 2.20E-238 |
| FimH | 1509 | L912_3621 | ESA31439 | mannose-specific adhesin FimH | Escherichia coli SCD1 | 804.6 | 2.30E-238 |
| FimH | 1510 | EC54115_03927 | EIL57997 | adhesin | Escherichia coli 541-15 | 804.6 | 2.30E-238 |
| FimH | 1511 | AC96_4810 | KDX22361 | protein fimH | Escherichia coli 2-156-04_S4_C2 | 804.6 | 2.30E-238 |
| FimH | 1512 | ECP02989426_4832 | ENB55874 | protein fimH | Escherichia coli P0298942.6 | 804.5 | 2.30E-238 |
| FimH | 1513 | E2348C_4627 | CAS12175 | minor component of type 1 fimbriae | Escherichia coli O127:H6 str. E2348/69 | 804.5 | 2.30E-238 |
| FimH | 1514 | ECARS42123_4870 | EKI21526 | protein fimH | Escherichia coli ARS4.2123 | 804.5 | 2.30E-238 |
| FimH | 1515 | EC3003_4859 | EII87794 | mannose-binding domain protein FimH | Escherichia coli 3003 | 804.5 | 2.30E-238 |
| FimH | 1516 | ECRN5871_1615 | EFZ75108 | protein fimH | Escherichia coli RN587/1 | 804.5 | 2.30E-238 |
| FimH | 1517 | ERS085359_04026 | CTT65755 | FimH protein | Escherichia coli (GCA_001284505) | 804.4 | 2.50E-238 |
| FimH | 1518 | ERS139241_04127 | CTY46076 | FimH protein | Escherichia coli (GCA_001285545) | 804.4 | 2.50E-238 |
| FimH | 1519 | HMPREF1623_01291 | ESE25603 | protein FimH | Escherichia coli 910096-2 | 804.4 | 2.60E-238 |
| FimH | 1520 | EH64_09835 | KEP03989 | fimbrial protein | Escherichia coli str. UCD_JA23 | 804.4 | 2.60E-238 |
| FimH | 1521 | A1WS_00275 | EOW30043 | fimbrial protein FimH | Escherichia coli KTE107 | 804.4 | 2.60E-238 |
| FimH | 1522 | ACU57_13795 | KPO11109 | fimbrial protein | Escherichia coli | 804.4 | 2.60E-238 |
| FimH | 1523 | ECDEC8B_5526 | EHW02907 | minor fimbrial subunit, D-mannose specific adhesin | Escherichia coli DEC8B | 804.2 | 3.00E-238 |
| FimH | 1524 | SB521682_5172 | EGI88523 | protein fimH | Shigella boydii 5216-82 | 804 | 3.30E-238 |
| FimH | 1525 | SF660363_4840 | EJL09927 | minor fimbrial subunit, D-mannose specific adhesin | Shigella flexneri 6603-63 | 803.9 | 3.70E-238 |
| FimH | 1526 | SFK272_0227 | EGK30686 | protein fimH | Shigella flexneri K-272 | 803.9 | 3.70E-238 |
| FimH | 1527 | SFyv_6099 | AIL43523 | fimbrial protein | Shigella flexneri Shi06HN006 | 803.9 | 3.70E-238 |
| FimH | 1528 | SFy_6031 | AIL38587 | fimbrial protein | Shigella flexneri 2003036 | 803.9 | 3.70E-238 |
| FimH | 1529 | SFK671_3950 | EGJ82505 | protein fimH | Shigella flexneri K-671 | 803.9 | 3.70E-238 |
| FimH | 1530 | SFK304_5232 | EGK31466 | protein fimH | Shigella flexneri K-304 | 803.9 | 3.70E-238 |
| FimH | 1531 | SF5M90T_4114 | EID64417 | FimH protein | Shigella flexneri 5a str. M90T | 803.9 | 3.70E-238 |
| FimH | 1532 | SF4200 | AAN45621 | minor fimbrial subunit, D-mannose specific adhesin | Shigella flexneri 2a str. 301 | 803.9 | 3.70E-238 |
| FimH | 1533 | ERS574920_03333 | CEP58472 | protein FimH | Shigella flexneri 2a | 803.9 | 3.70E-238 |
| FimH | 1534 | SF285071_4093 | EIQ05202 | protein fimH | Shigella flexneri 2850-71 | 803.9 | 3.70E-238 |
| FimH | 1535 | SFK227_5262 | EGK31550 | protein fimH | Shigella flexneri K-227 | 803.9 | 3.70E-238 |
| FimH | 1536 | SF274771_5010 | EGJ80869 | protein fimH | Shigella flexneri 2747-71 | 803.9 | 3.70E-238 |
| FimH | 1537 | SF123566_7369 | EIQ52932 | protein fimH | Shigella flexneri 1235-66 | 803.9 | 3.70E-238 |
| FimH | 1538 | AD871_23965 | AMN65281 | fimbrial protein | Shigella flexneri 4c | 803.9 | 3.70E-238 |
| FimH | 1539 | SFJ1713_5001 | EGM58759 | minor fimbrial subunit, D-mannose specific adhesin | Shigella flexneri SFJ17B | 803.9 | 3.70E-238 |
| FimH | 1540 | SF2A_22785 | AKK56597 | fimbrial protein FimH | Shigella flexneri G1663 | 803.9 | 3.70E-238 |
| FimH | 1541 | SFxv_4583 | ADA76499 | minor fimbrial subunit, D-mannose specific adhesin | Shigella flexneri 2002017 | 803.9 | 3.70E-238 |
| FimH | 1542 | SFK404_5368 | EIQ19695 | protein fimH | Shigella flexneri K-404 | 803.9 | 3.70E-238 |
| FimH | 1543 | NCTC1_04566 | CDX09629 | protein FimH,hypothetical protein,fimbrial chaperone protein,P pilus assembly protein, pilin FimA,FimH, mannose binding | Shigella flexneri | 803.9 | 3.70E-238 |
| FimH | 1544 | SFK218_5442 | EGK16168 | protein fimH | Shigella flexneri K-218 | 803.9 | 3.70E-238 |
| FimH | 1545 | S4456 | AAP19406 | minor fimbrial subunit, D-mannose specific adhesin | Shigella flexneri 2a str. 2457T (GCA_000183785) | 803.9 | 3.70E-238 |
| FimH | 1546 | SF293071_3881 | EGJ94936 | minor fimbrial subunit, D-mannose specific adhesin | Shigella flexneri 2930-71 | 803.9 | 3.70E-238 |
| FimH | 1547 | SFV_4206 | ABF06190 | FimH protein precursor | Shigella flexneri 5 str. 8401 | 803.9 | 3.70E-238 |
| FimH | 1548 | ECDEC12A_0039 | EHX37059 | protein fimH | Escherichia coli DEC12A | 803.8 | 3.80E-238 |
| FimH | 1549 | EC2016001_0334 | ENA25934 | protein fimH | Escherichia coli 201600.1 | 803.8 | 3.80E-238 |
| FimH | 1550 | ECP029943810_4845 | ENB84232 | protein fimH | Escherichia coli P0299438.10 | 803.8 | 3.80E-238 |
| FimH | 1551 | AC73_3879 | KDY40591 | protein fimH | Escherichia coli 2-427-07_S4_C1 | 803.8 | 3.80E-238 |
| FimH | 1552 | ECTX1999_5182 | EGX18422 | protein fimH | Escherichia coli TX1999 | 803.8 | 3.80E-238 |
| FimH | 1553 | ECDEC12D_5616 | EHX38160 | minor fimbrial subunit, D-mannose specific adhesin | Escherichia coli DEC12D | 803.8 | 3.80E-238 |
| FimH | 1554 | ECDEC7D_0097 | EHV96586 | minor fimbrial subunit, D-mannose specific adhesin | Escherichia coli DEC7D | 803.8 | 3.80E-238 |
| FimH | 1555 | ERDG_03715 | EGB35879 | FimH protein | Escherichia coli E482 | 803.8 | 3.80E-238 |
| FimH | 1556 | ECE128010_0502 | EFZ49149 | protein fimH | Escherichia coli E128010 | 803.8 | 3.80E-238 |
| FimH | 1557 | ECEPECA12_5175 | EIQ57958 | protein fimH | Escherichia coli EPECa12 | 803.8 | 3.80E-238 |
| FimH | 1558 | WGE_00633 | ELF78995 | fimbrial protein FimH | Escherichia coli KTE42 | 803.8 | 3.80E-238 |
| FimH | 1559 | ECDEC12C_5350 | EHX24090 | protein fimH | Escherichia coli DEC12C | 803.8 | 3.80E-238 |
| FimH | 1560 | ECG581_0043 | EGX13870 | protein fimH | Escherichia coli G58-1 | 803.8 | 3.80E-238 |
| FimH | 1561 | EC2762100_5083 | EMW48607 | protein fimH | Escherichia coli 2762100 | 803.8 | 3.80E-238 |
| FimH | 1562 | C4893_41820 | EMR92145 | minor component of type 1 fimbriae | Escherichia coli ONT:H33 str. C48/93 | 803.8 | 3.80E-238 |
| FimH | 1563 | ECDEC7E_4983 | EHV94344 | protein fimH | Escherichia coli DEC7E | 803.8 | 3.80E-238 |
| FimH | 1564 | AB54_4733 | KDS94766 | protein fimH | Escherichia coli 2-011-08_S1_C3 | 803.8 | 3.80E-238 |
| FimH | 1565 | ECEPECC34262_0042 | EIQ72997 | minor fimbrial subunit, D-mannose specific adhesin | Escherichia coli EPEC C342-62 | 803.8 | 3.80E-238 |
| FimH | 1566 | BY43_15420 | EZA75352 | fimbrial protein | Escherichia coli O25:NM str. E2539C1 | 803.8 | 3.80E-238 |
| FimH | 1567 | ECLT68_3974 | EFZ56981 | protein fimH | Escherichia coli LT-68 | 803.8 | 3.80E-238 |
| FimH | 1568 | EcB171_2735 | EDX31708 | protein FimH | Escherichia coli B171 | 803.8 | 3.80E-238 |
| FimH | 1569 | ECDEC12B_0039 | EHX37426 | minor fimbrial subunit, D-mannose specific adhesin | Escherichia coli DEC12B | 803.8 | 3.80E-238 |
| FimH | 1570 | AB87_4321 | KDX53005 | protein fimH | Escherichia coli 2-210-07_S3_C1 | 803.8 | 3.80E-238 |
| FimH | 1571 | ECDEC12E_0031 | EHX54712 | minor fimbrial subunit, D-mannose specific adhesin | Escherichia coli DEC12E | 803.8 | 3.80E-238 |
| FimH | 1572 | ERS085440_03840 | CTS97902 | FimH protein | Escherichia coli | 803.8 | 3.80E-238 |
| FimH | 1573 | EC2731150_4990 | EMW70557 | protein fimH | Escherichia coli 2731150 | 803.8 | 3.80E-238 |
| FimH | 1574 | G943_04915 | EQY08757 | protein fimH | Escherichia coli UMEA 3212-1 | 803.8 | 3.80E-238 |
| FimH | 1575 | ECDEC7C_0032 | EHV91095 | minor fimbrial subunit, D-mannose specific adhesin | Escherichia coli DEC7C | 803.8 | 3.80E-238 |
| FimH | 1576 | ECB41_4817 | EIJ02085 | mannose-binding domain protein FimH | Escherichia coli B41 | 803.8 | 3.80E-238 |
| FimH | 1577 | ECDEC7A_0007 | EHV81993 | protein fimH | Escherichia coli DEC7A | 803.8 | 3.80E-238 |
| FimH | 1578 | ECMP0215612_0303 | EMX33703 | protein fimH | Escherichia coli MP021561.2 | 803.8 | 3.80E-238 |
| FimH | 1579 | AD43_5029 | KDT47670 | protein fimH | Escherichia coli 3-105-05_S4_C3 | 803.8 | 3.80E-238 |
| FimH | 1580 | A9D65_17980 | ODA87761 | fimbrial protein | Escherichia coli | 803.8 | 3.90E-238 |
| FimH | 1581 | EC75_04621 | EIL70476 | minor component of type 1 fimbriae | Escherichia coli 75 | 803.8 | 3.90E-238 |
| FimH | 1582 | ECA727_15835 | EST77918 | minor component of type 1 fimbriae | Escherichia coli ECA-727 | 803.8 | 3.90E-238 |
| FimH | 1583 | G796_04501 | EQR88654 | protein fimH | Escherichia coli HVH 138 (4-6066704) | 803.7 | 4.00E-238 |
| FimH | 1584 | GR02_21125 | KFH97616 | fimbrial protein | Escherichia coli str. CS01 | 803.7 | 4.10E-238 |
| FimH | 1585 | SbBS512_E4794 | ACD10421 | protein FimH | Shigella boydii CDC 3083-94 | 803.7 | 4.10E-238 |
| FimH | 1586 | WC3_00373 | EOU56828 | fimbrial protein FimH | Escherichia coli KTE35 | 803.7 | 4.20E-238 |
| FimH | 1587 | WEY_00377 | EOV00583 | fimbrial protein FimH | Escherichia coli KTE34 | 803.7 | 4.20E-238 |
| FimH | 1588 | C827_04192 | EMZ38871 | protein fimH | Escherichia coli SWW33 | 803.5 | 4.70E-238 |
| FimH | 1589 | ECP03052604_4701 | ENF98077 | protein fimH | Escherichia coli P0305260.4 | 803.5 | 4.80E-238 |
| FimH | 1590 | ECP030526013_4714 | ENF84123 | protein fimH | Escherichia coli P0305260.13 | 803.5 | 4.80E-238 |
| FimH | 1591 | ECP02994389_4613 | ENC26274 | protein fimH | Escherichia coli P0299438.9 | 803.5 | 4.80E-238 |
| FimH | 1592 | PU70_19140 | KIG36021 | fimbrial protein | Escherichia coli (GCA_000819285) | 803.5 | 4.90E-238 |
| FimH | 1593 | WE9_00125 | ELC44846 | protein fimH | Escherichia coli KTE21 | 803.4 | 5.00E-238 |
| FimH | 1594 | G966_05074 | ESK25397 | protein fimH | Escherichia coli UMEA 3323-1 | 803.4 | 5.00E-238 |
| FimH | 1595 | WE7_00095 | EOU74399 | fimbrial protein FimH | Escherichia coli KTE20 | 803.4 | 5.00E-238 |
| FimH | 1596 | G701_04778 | EQN87718 | protein fimH | Escherichia coli HVH 25 (4-5851939) | 803.4 | 5.00E-238 |
| FimH | 1597 | SM09_00666 | KME75886 | protein FimH | Escherichia coli HVH 25 (4-5851939) | 803.4 | 5.00E-238 |
| FimH | 1598 | ECP03018675_5034 | ENG96467 | protein fimH | Escherichia coli P0301867.5 | 803.4 | 5.10E-238 |
| FimH | 1599 | ECP030529310_4625 | ENG25609 | protein fimH | Escherichia coli p0305293.10 | 803.4 | 5.10E-238 |
| FimH | 1600 | BY40_21420 | EZA68174 | fimbrial protein | Escherichia coli O157:H16 str. 98-3133 | 803.4 | 5.10E-238 |
| FimH | 1601 | AB51_4715 | KEM08626 | protein fimH | Escherichia coli 6-319-05_S1_C2 | 803.4 | 5.10E-238 |
| FimH | 1602 | ECP03052938_4722 | ENG67734 | protein fimH | Escherichia coli p0305293.8 | 803.4 | 5.10E-238 |
| FimH | 1603 | ECMP02155211_4666 | EMU56365 | protein fimH | Escherichia coli MP021552.11 | 803.4 | 5.10E-238 |
| FimH | 1604 | G918_04492 | ERB12101 | protein fimH | Escherichia coli UMEA 3150-1 | 803.4 | 5.10E-238 |
| FimH | 1605 | EC178850_4564 | ENG83835 | protein fimH | Escherichia coli 178850 | 803.4 | 5.10E-238 |
| FimH | 1606 | EC2747800_4713 | EMW70275 | protein fimH | Escherichia coli 2747800 | 803.4 | 5.10E-238 |
| FimH | 1607 | ECSTECS1191_0262 | EGX21686 | protein fimH | Escherichia coli STEC_S1191 | 803.4 | 5.10E-238 |
| FimH | 1608 | ECP030529314_4861 | ENE04575 | protein fimH | Escherichia coli p0305293.14 | 803.4 | 5.10E-238 |
| FimH | 1609 | ECP030529313_4584 | END30671 | protein fimH | Escherichia coli p0305293.13 | 803.4 | 5.10E-238 |
| FimH | 1610 | ECTW15901_4880 | EKI14225 | type 1 fimbiral adhesin FimH | Escherichia coli TW15901 | 803.4 | 5.10E-238 |
| FimH | 1611 | EC2729250_4760 | ENA47401 | protein fimH | Escherichia coli 2729250 | 803.4 | 5.10E-238 |
| FimH | 1612 | AB91_5819 | KDY38684 | protein fimH | Escherichia coli 2-460-02_S3_C1 | 803.4 | 5.10E-238 |
| FimH | 1613 | ECDEC6D_5119 | EHV65437 | protein fimH | Escherichia coli DEC6D | 803.4 | 5.10E-238 |
| FimH | 1614 | EC80566_4602 | EKK38130 | type 1 fimbiral adhesin FimH | Escherichia coli 8.0566 | 803.4 | 5.10E-238 |
| FimH | 1615 | ECP030529312_4700 | ENG39262 | protein fimH | Escherichia coli p0305293.12 | 803.4 | 5.10E-238 |
| FimH | 1616 | A1SU_00026 | EOV57285 | fimbrial protein FimH | Escherichia coli KTE61 | 803.4 | 5.10E-238 |
| FimH | 1617 | AC20_5410 | KDY54912 | protein fimH | Escherichia coli 2-460-02_S3_C2 | 803.4 | 5.10E-238 |
| FimH | 1618 | EC23916_5650 | EII45433 | mannose-binding domain protein FimH | Escherichia coli 2.3916 | 803.4 | 5.10E-238 |
| FimH | 1619 | AC49_4181 | KDY58429 | protein fimH | Escherichia coli 2-460-02_S3_C3 | 803.4 | 5.10E-238 |
| FimH | 1620 | ECMP0215527_5005 | EMU56338 | protein fimH | Escherichia coli MP021552.7 | 803.4 | 5.10E-238 |
| FimH | 1621 | ECTW00353_4787 | EKI21424 | type 1 fimbiral adhesin FimH | Escherichia coli TW00353 | 803.4 | 5.10E-238 |
| FimH | 1622 | L340_3487 | EPH48406 | mannose-specific adhesin FimH | Escherichia coli E2265 | 803.4 | 5.10E-238 |
| FimH | 1623 | EC80569_4582 | EKK39125 | protein fimH | Escherichia coli 8.0569 | 803.4 | 5.10E-238 |
| FimH | 1624 | EC2860650_4706 | ENA88526 | protein fimH | Escherichia coli 2860650 | 803.4 | 5.10E-238 |
| FimH | 1625 | ECMP0215528_5217 | EMX32756 | protein fimH | Escherichia coli MP021552.8 | 803.4 | 5.10E-238 |
| FimH | 1626 | ECP03052936_4611 | ENH50966 | protein fimH | Escherichia coli p0305293.6 | 803.4 | 5.10E-238 |
| FimH | 1627 | UWO_18530 | EIF16578 | FimH mannose-binding domain-containing protein | Escherichia coli O32:H37 str. P4 | 803.4 | 5.10E-238 |
| FimH | 1628 | ECDEC6C_5141 | EHV52521 | protein fimH | Escherichia coli DEC6C | 803.4 | 5.10E-238 |
| FimH | 1629 | ECMP02155212_0271 | EMU72107 | protein fimH | Escherichia coli MP021552.12 | 803.4 | 5.10E-238 |
| FimH | 1630 | AE12_04077 | KDG73961 | protein fimH | Escherichia coli UCI 53 | 803.4 | 5.10E-238 |
| FimH | 1631 | ECP03052939_4590 | ENG74318 | protein fimH | Escherichia coli p0305293.9 | 803.4 | 5.10E-238 |
| FimH | 1632 | ECENVIRA811_0036 | EMX78086 | protein fimH | Escherichia coli Envira 8/11 | 803.4 | 5.10E-238 |
| FimH | 1633 | ECDEC6E_5026 | EHV68515 | minor fimbrial subunit, D-mannose specific adhesin | Escherichia coli DEC6E | 803.4 | 5.10E-238 |
| FimH | 1634 | A1WY_00582 | ELF02998 | protein fimH | Escherichia coli KTE111 | 803.4 | 5.10E-238 |
| FimH | 1635 | UMNF18_5384 | AEJ59812 | protein fimH | Escherichia coli UMNF18 | 803.4 | 5.10E-238 |
| FimH | 1636 | ERS139242_04276 | CTY23145 | FimH protein | Escherichia coli | 803.4 | 5.10E-238 |
| FimH | 1637 | EC32303_4958 | EII75258 | mannose-binding domain protein FimH | Escherichia coli 3.2303 | 803.4 | 5.10E-238 |
| FimH | 1638 | ECP030529311_4821 | ENG35155 | protein fimH | Escherichia coli p0305293.11 | 803.4 | 5.10E-238 |
| FimH | 1639 | G964_04344 | EQY81876 | protein fimH | Escherichia coli UMEA 3317-1 | 803.4 | 5.10E-238 |
| FimH | 1640 | ECP03052934_4743 | ENG57697 | protein fimH | Escherichia coli p0305293.4 | 803.4 | 5.10E-238 |
| FimH | 1641 | EC2860050_4803 | EMV87440 | protein fimH | Escherichia coli 2860050 | 803.4 | 5.10E-238 |
| FimH | 1642 | AC70_1389 | KDW89292 | protein fimH | Escherichia coli 2-210-07_S4_C1 | 803.4 | 5.10E-238 |
| FimH | 1643 | ECP03052931_5082 | EMZ79607 | protein fimH | Escherichia coli p0305293.1 | 803.4 | 5.10E-238 |
| FimH | 1644 | AB23_4758 | KEM96528 | protein fimH | Escherichia coli 6-319-05_S1_C1 | 803.4 | 5.10E-238 |
| FimH | 1645 | EC2874_20605 | KRR54445 | FimH mannose-binding domain-containing protein | Escherichia coli VL2874 | 803.4 | 5.10E-238 |
| FimH | 1646 | AB80_4894 | KEM21709 | protein fimH | Escherichia coli 6-319-05_S1_C3 | 803.4 | 5.10E-238 |
| FimH | 1647 | ECP030529315_4765 | ENG48112 | protein fimH | Escherichia coli p0305293.15 | 803.4 | 5.10E-238 |
| FimH | 1648 | G711_04702 | ESP06160 | protein fimH | Escherichia coli HVH 36 (4-5675286) | 803.4 | 5.10E-238 |
| FimH | 1649 | ECP03052937_4741 | ENH48657 | protein fimH | Escherichia coli p0305293.7 | 803.4 | 5.10E-238 |
| FimH | 1650 | ECENVIRA101_0030 | EMX76340 | protein fimH | Escherichia coli Envira 10/1 | 803.4 | 5.10E-238 |
| FimH | 1651 | EC2866350_4681 | ENB03995 | protein fimH | Escherichia coli 2866350 | 803.4 | 5.10E-238 |
| FimH | 1652 | AB45_3864 | KDZ79935 | protein fimH | Escherichia coli 3-105-05_S1_C2 | 803.4 | 5.10E-238 |
| FimH | 1653 | A31E_04161 | EOW56832 | fimbrial protein FimH | Escherichia sp. KTE159 | 803.4 | 5.20E-238 |
| FimH | 1654 | G995_04748 | ERA00101 | protein fimH | Escherichia coli UMEA 3805-1 | 803.4 | 5.20E-238 |
| FimH | 1655 | HMPREF1602_05054 | ESD31657 | protein FimH | Escherichia coli 907889 | 803.4 | 5.20E-238 |
| FimH | 1656 | HMPREF1610_03979 | ESD66059 | protein FimH | Escherichia coli 908555 | 803.3 | 5.30E-238 |
| FimH | 1657 | EL77_3581 | KGM63414 | Protein fimH | Escherichia coli str. G4/9 | 803.3 | 5.30E-238 |
| FimH | 1658 | A1U5_00247 | ELE46156 | protein fimH | Escherichia coli KTE66 | 803.3 | 5.40E-238 |
| FimH | 1659 | HMPREF9551_00610 | EFI90316 | protein FimH | Escherichia coli MS 196-1 | 803.1 | 6.20E-238 |
| FimH | 1660 | A1YG_00375 | EOW53824 | fimbrial protein FimH | Escherichia coli KTE130 | 803.1 | 6.20E-238 |
| FimH | 1661 | A1YI_00413 | EOW53771 | fimbrial protein FimH | Escherichia coli KTE132 | 803.1 | 6.20E-238 |
| FimH | 1662 | AB88_5076 | KEN84376 | protein fimH | Escherichia coli 2-222-05_S3_C1 | 803.1 | 6.20E-238 |
| FimH | 1663 | G979_04824 | EQZ33245 | protein fimH | Escherichia coli UMEA 3609-1 | 803.1 | 6.20E-238 |
| FimH | 1664 | ECK5_20360 | CCP96088 | mannose-specific adhesin FimH | Escherichia coli O10:K5(L):H4 str. ATCC 23506 | 803.1 | 6.20E-238 |
| FimH | 1665 | G978_04654 | EQZ28308 | protein fimH | Escherichia coli UMEA 3592-1 | 803.1 | 6.20E-238 |
| FimH | 1666 | AC18_4889 | KEN93633 | protein fimH | Escherichia coli 2-222-05_S3_C2 | 803.1 | 6.20E-238 |
| FimH | 1667 | AC46_3692 | KDX84853 | protein fimH | Escherichia coli 2-222-05_S3_C3 | 803.1 | 6.20E-238 |
| FimH | 1668 | G925_04672 | EQX22129 | protein fimH | Escherichia coli (strain UMEA 3162-1) | 803.1 | 6.30E-238 |
| FimH | 1669 | AD09_4740 | KDA87321 | protein fimH | Escherichia coli 1-176-05_S4_C2 | 803.1 | 6.30E-238 |
| FimH | 1670 | ERS372666_04536 | CUK16573 | fimbrial chaperone protein | Achromobacter sp. ATCC35328 | 803.1 | 6.30E-238 |
| FimH | 1671 | G866_03450 | ETF28072 | protein fimH | Escherichia coli HVH 214 (4-3062198) | 803.1 | 6.30E-238 |
| FimH | 1672 | G825_04867 | EQS96614 | protein fimH | Escherichia coli HVH 170 (4-3026949) | 803.1 | 6.30E-238 |
| FimH | 1673 | G937_04526 | EQX79095 | protein fimH | Escherichia coli UMEA 3199-1 | 803.1 | 6.30E-238 |
| FimH | 1674 | G753_04360 | EQQ13363 | protein fimH | Escherichia coli HVH 91 (4-4638751) | 803.1 | 6.30E-238 |
| FimH | 1675 | AD00_4495 | KDY14306 | protein fimH | Escherichia coli 2-316-03_S4_C2 | 803.1 | 6.30E-238 |
[truncated: 33,166 more chars]
