# Supplementary material for: Functional role of the type 1 pilus rod structure in mediating host-pathogen interactions
Source: eLife. 2018 Jan 18;7:e31662. doi: 10.7554/eLife.31662 (PMC5798934; doi:10.7554/eLife.31662)
Supplement: Supplementary file 5. [file elife-31662-supp5.docx]

| UTI89 Codon Position | AA Identity^a^ | *dS* | *dN* | *dN*/*dS*^b^ | Normalized *dN*-*dS* | dS (when dN=dS) | Log(L) | LRT | p-value^c^ |
| --- | --- | --- | --- | --- | --- | --- | --- | --- | --- |
| 1 | 49.18 | 0.063 | 1.639 | 25.906 | 1.092 | 1.193 | -48.304 | 2.683 | 0.101 |
| 2 | 51.04 | 2.355 | 1.548 | 0.657 | -0.559 | 1.818 | -67.600 | 0.619 | 0.431 |
| 3 | 85.83 | 1.038 | 0.000 | 0.000 | -0.719 | 0.264 | -8.200 | 2.600 | 0.107 |
| 4 | 85.83 | 0.000 | 0.000 | Undefined | 0.000 | 0.000 | -4.393 | 0.000 | 1.000 |
| 5 | 100.00 | 1.026 | 0.000 | 0.000 | -0.711 | 0.224 | -17.754 | 6.074 | 0.014 |
| 6 | 100.00 | 0.000 | 0.000 | Undefined | 0.000 | 0.000 | 0.000 | 0.000 | 1.000 |
| 7 | 99.89 | 1.659 | 0.307 | 0.185 | -0.936 | 0.738 | -47.275 | 4.549 | 0.033 |
| 8 | 100.00 | 0.000 | 0.000 | Undefined | 0.000 | 0.000 | 0.000 | 0.000 | 1.000 |
| 9 | 99.95 | 1.120 | 0.211 | 0.188 | -0.630 | 0.538 | -28.763 | 2.456 | 0.117 |
| 10 | 100.00 | 0.000 | 0.000 | Undefined | 0.000 | 0.000 | 0.000 | 0.000 | 1.000 |
| 11 | 99.89 | 0.748 | 0.169 | 0.226 | -0.401 | 0.373 | -27.923 | 1.598 | 0.206 |
| 12 | 98.47 | 0.750 | 0.305 | 0.407 | -0.308 | 0.434 | -28.926 | 0.770 | 0.380 |
| 13 | 99.84 | 0.000 | 0.153 | Infinite | 0.106 | 0.099 | -13.033 | 0.859 | 0.354 |
| 14 | 100.00 | 0.524 | 0.000 | 0.000 | -0.363 | 0.098 | -10.881 | 3.314 | 0.069 |
| 15 | 100.00 | 0.000 | 0.000 | Undefined | 0.000 | 0.000 | 0.000 | 0.000 | 1.000 |
| 16 | 100.00 | 1.023 | 0.000 | 0.000 | -0.709 | 0.298 | -18.065 | 4.918 | 0.027 |
| 17 | 100.00 | 1.435 | 0.000 | 0.000 | -0.994 | 0.232 | -13.093 | 7.244 | 0.007 |
| 18 | 99.67 | 0.349 | 0.149 | 0.426 | -0.139 | 0.208 | -14.583 | 0.351 | 0.554 |
| 19 | 100.00 | 0.000 | 0.000 | Undefined | 0.000 | 0.000 | 0.000 | 0.000 | 1.000 |
| 20 | 100.00 | 1.758 | 0.000 | 0.000 | -1.218 | 0.467 | -26.330 | 13.041 | 0.000 |
| 21 | 100.00 | 0.962 | 0.000 | 0.000 | -0.666 | 0.418 | -25.648 | 6.597 | 0.010 |
| 22 | 100.00 | 0.000 | 0.000 | Undefined | 0.000 | 0.000 | 0.000 | 0.000 | 1.000 |
| 23 | 100.00 | 1.902 | 0.000 | 0.000 | -1.317 | 0.589 | -22.233 | 11.502 | 0.001 |
| 24 | 99.84 | 2.663 | 0.186 | 0.070 | -1.716 | 1.090 | -44.447 | 10.719 | 0.001 |
| 25 | 100.00 | 0.000 | 0.000 | Undefined | 0.000 | 0.000 | 0.000 | 0.000 | 1.000 |
| 26 | 100.00 | 0.000 | 0.000 | Undefined | 0.000 | 0.000 | 0.000 | 0.000 | 1.000 |
| 27 | 99.73 | 2.328 | 0.375 | 0.161 | -1.353 | 1.094 | -49.520 | 6.361 | 0.012 |
| 28 | 100.00 | 0.231 | 0.000 | 0.000 | -0.160 | 0.110 | -8.401 | 1.486 | 0.223 |
| 29 | 98.91 | 0.000 | 0.163 | Infinite | 0.113 | 0.111 | -12.740 | 0.765 | 0.382 |
| 30 | 99.51 | 0.356 | 0.153 | 0.429 | -0.141 | 0.213 | -19.701 | 0.345 | 0.557 |
| 31 | 100.00 | 0.000 | 0.000 | Undefined | 0.000 | 0.000 | 0.000 | 0.000 | 1.000 |
| 32 | 100.00 | 0.000 | 0.000 | Undefined | 0.000 | 0.000 | 0.000 | 0.000 | 1.000 |
| 33 | 99.45 | 0.703 | 0.287 | 0.409 | -0.288 | 0.446 | -34.211 | 0.971 | 0.324 |
| 34 | 99.95 | 0.000 | 0.152 | Infinite | 0.106 | 0.106 | -11.034 | 0.722 | 0.396 |
| 35 | 99.95 | 0.000 | 0.182 | Infinite | 0.126 | 0.151 | -13.483 | 0.381 | 0.537 |
| 36 | 99.95 | 5.523 | 0.154 | 0.028 | -3.719 | 1.592 | -64.866 | 26.659 | 0.000 |
| 37 | 100.00 | 5.968 | 0.000 | 0.000 | -4.135 | 2.022 | -62.762 | 29.334 | 0.000 |
| 38 | 100.00 | 0.902 | 0.000 | 0.000 | -0.625 | 0.151 | -9.561 | 3.555 | 0.059 |
| 39 | 99.95 | 0.364 | 0.152 | 0.418 | -0.147 | 0.215 | -18.645 | 0.365 | 0.546 |
| 40 | 99.95 | 0.000 | 0.196 | Infinite | 0.136 | 0.126 | -12.829 | 0.889 | 0.346 |
| 41 | 100.00 | 0.468 | 0.000 | 0.000 | -0.325 | 0.177 | -14.348 | 3.873 | 0.049 |
| 42 | 100.00 | 2.194 | 0.000 | 0.000 | -1.520 | 0.738 | -28.474 | 12.786 | 0.000 |
| 43 | 96.66 | 3.962 | 1.117 | 0.282 | -1.970 | 1.930 | -75.585 | 4.501 | 0.034 |
| 44 | 100.00 | 1.057 | 0.000 | 0.000 | -0.733 | 0.343 | -21.097 | 6.714 | 0.010 |
| 45 | 45.35 | 1.856 | 1.558 | 0.840 | -0.206 | 1.639 | -62.743 | 0.063 | 0.801 |
| 46 | 99.84 | 3.736 | 0.532 | 0.143 | -2.219 | 1.042 | -37.672 | 5.923 | 0.015 |
| 47 | 33.92 | 1.737 | 1.948 | 1.121 | 0.146 | 1.897 | -69.047 | 0.028 | 0.867 |
| 48 | 100.00 | 0.587 | 0.000 | 0.000 | -0.407 | 0.249 | -14.333 | 3.420 | 0.064 |
| 49 | 100.00 | 1.565 | 0.000 | 0.000 | -1.084 | 0.586 | -28.212 | 9.789 | 0.002 |
| 50 | 100.00 | 0.228 | 0.000 | 0.000 | -0.158 | 0.088 | -11.113 | 1.908 | 0.167 |
| 51 | 100.00 | 0.716 | 0.000 | 0.000 | -0.496 | 0.198 | -12.678 | 5.088 | 0.024 |
| 52 | 100.00 | 0.000 | 0.000 | Undefined | 0.000 | 0.000 | 0.000 | 0.000 | 1.000 |
| 53 | 100.00 | 2.502 | 0.000 | 0.000 | -1.733 | 0.758 | -26.816 | 13.594 | 0.000 |
| 54 | 99.29 | 0.490 | 0.152 | 0.311 | -0.234 | 0.282 | -21.021 | 0.972 | 0.324 |
| 55 | 99.95 | 0.000 | 0.210 | Infinite | 0.146 | 0.131 | -11.593 | 0.940 | 0.332 |
| 56 | 100.00 | 0.000 | 0.000 | Undefined | 0.000 | 0.000 | 0.000 | 0.000 | 1.000 |
| 57 | 100.00 | 0.287 | 0.000 | 0.000 | -0.199 | 0.088 | -11.240 | 2.349 | 0.125 |
| 58 | 100.00 | 0.000 | 0.000 | Undefined | 0.000 | 0.000 | 0.000 | 0.000 | 1.000 |
| 59 | 100.00 | 4.015 | 0.000 | 0.000 | -2.782 | 0.572 | -21.855 | 15.131 | 0.000 |
| 60 | 100.00 | 0.336 | 0.000 | 0.000 | -0.233 | 0.113 | -13.037 | 2.186 | 0.139 |
| 61 | 100.00 | 0.497 | 0.000 | 0.000 | -0.344 | 0.101 | -8.123 | 3.156 | 0.076 |
| 62 | 100.00 | 0.548 | 0.000 | 0.000 | -0.380 | 0.124 | -8.273 | 2.947 | 0.086 |
| 63 | 100.00 | 0.961 | 0.000 | 0.000 | -0.665 | 0.334 | -17.194 | 6.239 | 0.012 |
| 64 | 99.89 | 0.494 | 0.498 | 1.009 | 0.003 | 0.497 | -25.053 | 0.000 | 0.994 |
| 65 | 100.00 | 0.997 | 0.000 | 0.000 | -0.691 | 0.363 | -22.701 | 8.013 | 0.005 |
| 66 | 40.54 | 0.000 | 1.742 | Infinite | 1.207 | 1.275 | -50.432 | 6.736 | 0.009 |
| 67 | 99.95 | 0.000 | 0.152 | Infinite | 0.105 | 0.106 | -10.863 | 0.721 | 0.396 |
| 68 | 100.00 | 0.873 | 0.000 | 0.000 | -0.605 | 0.340 | -19.302 | 5.632 | 0.018 |
| 69 | 48.30 | 2.378 | 0.805 | 0.339 | -1.089 | 1.300 | -62.067 | 3.135 | 0.077 |
| 70 | 99.78 | 0.000 | 0.117 | Infinite | 0.081 | 0.100 | -10.329 | 0.324 | 0.569 |
| 71 | 100.00 | 2.329 | 0.000 | 0.000 | -1.613 | 0.901 | -39.485 | 14.642 | 0.000 |
| 72 | 99.89 | 0.732 | 0.375 | 0.511 | -0.248 | 0.495 | -29.302 | 0.431 | 0.512 |
| 73 | 93.11 | 0.000 | 0.296 | Infinite | 0.205 | 0.209 | -13.110 | 1.388 | 0.239 |
| 74 | 100.00 | 0.000 | 0.000 | Undefined | 0.000 | 0.000 | 0.000 | 0.000 | 1.000 |
| 75 | 100.00 | 0.997 | 0.000 | 0.000 | -0.690 | 0.266 | -18.025 | 7.809 | 0.005 |
| 76 | 99.84 | 1.451 | 0.475 | 0.327 | -0.676 | 0.750 | -42.442 | 1.302 | 0.254 |
| 77 | 100.00 | 1.610 | 0.000 | 0.000 | -1.115 | 0.548 | -21.591 | 8.426 | 0.004 |
| 78 | 100.00 | 2.981 | 0.000 | 0.000 | -2.065 | 0.750 | -31.191 | 18.929 | 0.000 |
| 79 | 89.55 | 0.995 | 0.760 | 0.764 | -0.163 | 0.846 | -38.418 | 0.120 | 0.729 |
| 80 | 100.00 | 0.000 | 0.000 | Undefined | 0.000 | 0.000 | 0.000 | 0.000 | 1.000 |
| 81 | 99.78 | 0.807 | 0.161 | 0.199 | -0.447 | 0.344 | -16.019 | 1.836 | 0.175 |
| 82 | 67.94 | 2.253 | 1.833 | 0.814 | -0.291 | 1.964 | -68.527 | 0.118 | 0.731 |
| 83 | 39.55 | 1.328 | 2.858 | 2.152 | 1.060 | 2.438 | -91.627 | 1.439 | 0.230 |
| 84 | 86.27 | 0.000 | 0.630 | Infinite | 0.436 | 0.479 | -26.950 | 2.189 | 0.139 |
| 85 | 79.27 | 1.395 | 0.632 | 0.453 | -0.529 | 0.877 | -38.898 | 1.213 | 0.271 |
| 86 | 54.70 | 2.053 | 1.088 | 0.530 | -0.668 | 1.318 | -59.849 | 0.806 | 0.369 |
| 87 | 99.95 | 1.561 | 0.152 | 0.098 | -0.976 | 0.617 | -38.208 | 6.431 | 0.011 |
| 88 | 100.00 | 1.761 | 0.000 | 0.000 | -1.220 | 0.568 | -27.461 | 11.210 | 0.001 |
| 89 | 99.95 | 0.362 | 0.186 | 0.514 | -0.122 | 0.246 | -15.404 | 0.215 | 0.643 |
| 90 | 100.00 | 1.745 | 0.000 | 0.000 | -1.209 | 0.571 | -29.423 | 11.064 | 0.001 |
| 91 | 99.73 | 1.866 | 0.180 | 0.096 | -1.168 | 0.451 | -22.969 | 3.755 | 0.053 |
| 92 | 100.00 | 0.000 | 0.000 | Undefined | 0.000 | 0.000 | 0.000 | 0.000 | 1.000 |
| 93 | 100.00 | 2.664 | 0.000 | 0.000 | -1.845 | 0.837 | -38.287 | 15.569 | 0.000 |
| 94 | 99.95 | 0.356 | 0.186 | 0.522 | -0.118 | 0.244 | -20.268 | 0.206 | 0.650 |
| 95 | 99.73 | 2.793 | 0.378 | 0.135 | -1.674 | 1.084 | -43.630 | 7.000 | 0.008 |
| 96 | 99.95 | 1.156 | 0.211 | 0.182 | -0.655 | 0.543 | -27.518 | 2.564 | 0.109 |
| 97 | 99.89 | 1.257 | 0.138 | 0.110 | -0.776 | 0.477 | -33.071 | 5.127 | 0.024 |
| 98 | 100.00 | 0.000 | 0.000 | Undefined | 0.000 | 0.000 | 0.000 | 0.000 | 1.000 |
| 99 | 99.95 | 0.301 | 0.144 | 0.478 | -0.109 | 0.195 | -16.761 | 0.265 | 0.607 |
| 100 | 93.44 | 3.100 | 0.647 | 0.209 | -1.699 | 1.243 | -63.405 | 7.075 | 0.008 |
| 101 | 100.00 | 0.000 | 0.000 | Undefined | 0.000 | 0.000 | 0.000 | 0.000 | 1.000 |
| 102 | 100.00 | 1.515 | 0.000 | 0.000 | -1.049 | 0.538 | -21.654 | 8.137 | 0.004 |
| 103 | 100.00 | 0.507 | 0.000 | 0.000 | -0.352 | 0.117 | -10.753 | 2.931 | 0.087 |
| 104 | 99.95 | 4.058 | 0.180 | 0.044 | -2.686 | 0.760 | -32.143 | 10.069 | 0.002 |
| 105 | 100.00 | 2.024 | 0.000 | 0.000 | -1.402 | 0.581 | -34.746 | 16.994 | 0.000 |
| 106 | 100.00 | 1.062 | 0.000 | 0.000 | -0.736 | 0.343 | -23.299 | 6.755 | 0.009 |
| 107 | 99.95 | 0.292 | 0.154 | 0.527 | -0.096 | 0.202 | -16.447 | 0.200 | 0.655 |
| 108 | 89.22 | 0.000 | 0.506 | Infinite | 0.351 | 0.404 | -29.254 | 1.786 | 0.181 |
| 109 | 99.95 | 1.875 | 0.137 | 0.073 | -1.204 | 0.605 | -35.536 | 8.274 | 0.004 |
| 110 | 100.00 | 6.311 | 0.000 | 0.000 | -4.372 | 2.000 | -61.316 | 31.635 | 0.000 |
| 111 | 63.02 | 0.000 | 1.959 | Infinite | 1.357 | 1.313 | -46.972 | 8.616 | 0.003 |
| 112 | 86.27 | 1.071 | 1.117 | 1.042 | 0.031 | 1.105 | -47.609 | 0.003 | 0.960 |
| 113 | 99.95 | 1.004 | 0.163 | 0.162 | -0.582 | 0.439 | -25.758 | 2.954 | 0.086 |
| 114 | 63.35 | 0.971 | 1.512 | 1.556 | 0.374 | 1.370 | -55.605 | 0.341 | 0.559 |
| 115 | 100.00 | 0.660 | 0.000 | 0.000 | -0.457 | 0.218 | -15.465 | 4.421 | 0.036 |
| 116 | 100.00 | 2.610 | 0.000 | 0.000 | -1.808 | 0.767 | -28.083 | 16.959 | 0.000 |
| 117 | 100.00 | 2.377 | 0.000 | 0.000 | -1.646 | 0.792 | -39.861 | 12.731 | 0.000 |
| 118 | 99.95 | 0.000 | 0.181 | Infinite | 0.125 | 0.131 | -8.948 | 0.635 | 0.426 |
| 119 | 89.33 | 0.000 | 0.591 | Infinite | 0.410 | 0.381 | -29.730 | 3.465 | 0.063 |
| 120 | 100.00 | 0.839 | 0.000 | 0.000 | -0.581 | 0.182 | -12.807 | 6.039 | 0.014 |
| 121 | 100.00 | 1.000 | 0.000 | 0.000 | -0.693 | 0.208 | -12.444 | 6.190 | 0.013 |
| 122 | 42.40 | 0.638 | 2.456 | 3.849 | 1.259 | 1.743 | -72.544 | 3.788 | 0.052 |
| 123 | 62.69 | 0.000 | 0.878 | Infinite | 0.609 | 0.714 | -40.843 | 2.468 | 0.116 |
| 124 | 99.95 | 1.193 | 0.000 | 0.000 | -0.827 | 0.376 | -24.671 | 9.209 | 0.002 |
| 125 | 99.89 | 0.697 | 0.137 | 0.197 | -0.388 | 0.345 | -24.931 | 2.348 | 0.125 |
| 126 | 100.00 | 1.323 | 0.000 | 0.000 | -0.917 | 0.436 | -26.864 | 8.823 | 0.003 |
| 127 | 99.95 | 0.521 | 0.123 | 0.235 | -0.276 | 0.198 | -17.269 | 0.955 | 0.328 |
| 128 | 99.95 | 0.884 | 0.123 | 0.139 | -0.528 | 0.346 | -23.107 | 3.503 | 0.061 |
| 129 | 100.00 | 2.102 | 0.000 | 0.000 | -1.456 | 0.769 | -28.146 | 11.775 | 0.001 |
| 130 | 99.84 | 1.233 | 0.137 | 0.111 | -0.760 | 0.528 | -31.159 | 5.666 | 0.017 |
| 131 | 100.00 | 0.280 | 0.000 | 0.000 | -0.194 | 0.085 | -9.904 | 2.376 | 0.123 |
| 132 | 99.56 | 0.446 | 0.414 | 0.928 | -0.022 | 0.426 | -33.992 | 0.007 | 0.935 |
| 133 | 100.00 | 0.000 | 0.000 | Undefined | 0.000 | 0.000 | 0.000 | 0.000 | 1.000 |
| 134 | 100.00 | 0.000 | 0.000 | Undefined | 0.000 | 0.000 | 0.000 | 0.000 | 1.000 |
| 135 | 99.95 | 0.274 | 0.117 | 0.426 | -0.109 | 0.164 | -19.965 | 0.351 | 0.554 |
| 136 | 99.84 | 0.000 | 0.175 | Infinite | 0.121 | 0.144 | -12.891 | 0.381 | 0.537 |
| 137 | 100.00 | 0.492 | 0.000 | 0.000 | -0.341 | 0.130 | -9.276 | 2.655 | 0.103 |
| 138 | 99.95 | 0.000 | 0.188 | Infinite | 0.130 | 0.121 | -12.074 | 0.888 | 0.346 |
| 139 | 100.00 | 0.499 | 0.000 | 0.000 | -0.346 | 0.119 | -10.979 | 2.833 | 0.092 |
| 140 | 58.97 | 0.000 | 0.884 | Infinite | 0.613 | 0.683 | -38.165 | 3.121 | 0.077 |
| 141 | 99.95 | 0.271 | 0.178 | 0.657 | -0.064 | 0.215 | -18.032 | 0.087 | 0.768 |
| 142 | 56.40 | 0.808 | 0.512 | 0.633 | -0.206 | 0.607 | -40.078 | 0.342 | 0.559 |
| 143 | 99.51 | 1.676 | 0.617 | 0.368 | -0.734 | 1.015 | -50.010 | 1.884 | 0.170 |
| 144 | 52.08 | 1.148 | 1.891 | 1.646 | 0.514 | 1.609 | -60.516 | 0.583 | 0.445 |
| 145 | 100.00 | 0.276 | 0.000 | 0.000 | -0.191 | 0.108 | -10.155 | 1.875 | 0.171 |
| 146 | 99.89 | 0.000 | 0.137 | Infinite | 0.095 | 0.084 | -11.525 | 0.977 | 0.323 |
| 147 | 99.62 | 11.326 | 0.337 | 0.030 | -7.613 | 2.667 | -81.398 | 40.731 | 0.000 |
| 148 | 100.00 | 5.532 | 0.000 | 0.000 | -3.832 | 1.860 | -46.874 | 29.087 | 0.000 |
| 149 | 99.95 | 5.407 | 0.178 | 0.033 | -3.622 | 1.837 | -68.581 | 23.781 | 0.000 |
| 150 | 100.00 | 0.000 | 0.000 | Undefined | 0.000 | 0.000 | 0.000 | 0.000 | 1.000 |
| 151 | 100.00 | 0.527 | 0.000 | 0.000 | -0.365 | 0.099 | -8.505 | 3.327 | 0.068 |
| 152 | 99.95 | 0.486 | 0.178 | 0.366 | -0.213 | 0.261 | -17.902 | 0.482 | 0.488 |
| 153 | 100.00 | 0.000 | 0.000 | Undefined | 0.000 | 0.000 | 0.000 | 0.000 | 1.000 |
| 154 | 100.00 | 1.333 | 0.000 | 0.000 | -0.924 | 0.381 | -18.178 | 7.466 | 0.006 |
| 155 | 99.95 | 0.000 | 0.137 | Infinite | 0.095 | 0.084 | -11.524 | 0.977 | 0.323 |
| 156 | 100.00 | 0.000 | 0.000 | Undefined | 0.000 | 0.000 | 0.000 | 0.000 | 1.000 |
| 157 | 100.00 | 0.849 | 0.000 | 0.000 | -0.588 | 0.112 | -10.636 | 4.027 | 0.045 |
| 158 | 100.00 | 0.000 | 0.000 | Undefined | 0.000 | 0.000 | 0.000 | 0.000 | 1.000 |
| 159 | 99.95 | 0.000 | 0.175 | Infinite | 0.121 | 0.144 | -12.900 | 0.381 | 0.537 |
| 160 | 100.00 | 0.000 | 0.000 | Undefined | 0.000 | 0.000 | 0.000 | 0.000 | 1.000 |
| 161 | 100.00 | 0.000 | 0.000 | Undefined | 0.000 | 0.000 | 0.000 | 0.000 | 1.000 |

^a^Amino acid position in alignment of 1,828 mature FimA sequences in Table S3

^b^Values of “Undefined” indicate codons where *dS* and *dN* = 0, preventing estimation of selection pressure (p-value = 1). Values of “Infinite” indicate codons where *dS* = 0 and *dN* >0

^c^p-values <0.1 are considered significant and are indicated in red text
