# Supplementary material for: Functional role of the type 1 pilus rod structure in mediating host-pathogen interactions
Source: eLife. 2018 Jan 18;7:e31662. doi: 10.7554/eLife.31662 (PMC5798934; doi:10.7554/eLife.31662)
Supplement: Supplementary file 6. [file elife-31662-supp6.docx]

| **Strain** | **Patvhotype** | **Clade** | **BioProject (NCBI ID)** | **FimA Carriage** |
| --- | --- | --- | --- | --- |
| 536 | UPEC | B2 | PRJNA16235 | Yes |
| 11128 | EHEC | B1 | PRJDA32513 | Yes |
| 11368 | EHEC | B1 | PRJDA32509 | Yes |
| 12009 | EHEC | B1 | PRJDA32511 | Yes |
| 55989 | EAEC | B1 | PRJNA33413 |  |
| 2009EL-2050 | EHEC | B1 | PRJNA81097 |  |
| 2009EL-2071 | EHEC | B1 | PRJNA81099 |  |
| 2011C-3493 | EHEC | B1 | PRJNA81095 |  |
| ABU 83972 | ABU | B2 | PRJNA38725 |  |
| APEC O1 | APEC | B2 | PRJNA16718 | Yes |
| APEC O78 | APEC | B1 | PRJNA184588 | Yes |
| ATCC 8739 | Commensal | A | PRJNA18083 |  |
| BL21(DE3) | Lab Strain | A | PRJNA20713 | Yes |
| BW2952 | Lab Strain | A | PRJNA33775 | Yes |
| CB9615 | EPEC | E | PRJNA42729 | Yes |
| CE10 | NMEC | D | PRJNA63597 | Yes |
| CFT073 | UPEC | B2 | PRJNA313 | Yes |
| clone D i14 | UPEC | B2 | PRJNA52023 | Yes |
| clone D i2 | UPEC | B2 | PRJNA52021 | Yes |
| DH1 | Lab Strain | A | PRJDA52077 | Yes |
| DH10B | Lab Strain | A | PRJNA20079 |  |
| E2348/69 | EPEC | B2 | PRJEA32571 | Yes |
| E24377A | ETEC | B1 | PRJNA13960 |  |
| EC4115 | EHEC | E | PRJNA27739 | Yes |
| ED1a | Commensal | B2 | PRJNA33409 | Yes |
| EDL933 | EHEC | E | PRJNA259 | Yes |
| H10407 | ETEC | A | PRJEA42749 | Yes |
| HS | Commensal | A | PRJNA13959 | Yes |
| IAI39 | UPEC | D | PRJNA33411 | Yes |
| IHE3034 | NMEC | B2 | PRJNA43693 | Yes |
| K12 MG1655 | Commensal | A | PRJNA40075 | Yes |
| LF82 | AIEC | B2 | PRJNA33825 | Yes |
| NRG 857C | AIEC | B2 | PRJNA41221 | Yes |
| REL606 | Lab Strain | A | PRJNA18281 | Yes |
| RM12579 | EPEC | E | PRJNA68245 | Yes |
| S88 | Commensal | B2 | PRJNA33375 | Yes |
| SE11 | Commensal | B1 | PRJNA18057 | Yes |
| SE15 | Commensal | B2 | PRJDA19053 | Yes |
| SMS-3-5 | Environmental | D | PRJNA19469 | Yes |
| TW14359 | EHEC | E | PRJNA30045 | Yes |
| UM146 | AIEC | B2 | PRJNA50883 | Yes |
| UMNK88 | ETEC | A | PRJNA42137 |  |
| UTI89 | UPEC | B2 | PRJNA16259 | Yes |
| W | Lab Strain | B1 | PRJNA48011 | Yes |
| W3110 | Lab Strain | A | PRJNA16351 | Yes |
| Xuzhou21 | EHEC | E | PRJNA45823 | Yes |
| 2.1a | UPEC | A | PRJNA269984 | Yes |
| 2.2r | UPEC | D | PRJNA269984 | Yes |
| 5.1a | UPEC | B1 | PRJNA269984 |  |
| 5.3r | UPEC | B2 | PRJNA269984 | Yes |
| 9.1a | UPEC | D | PRJNA269984 | Yes |
| 9.2p | UPEC | B1 | PRJNA269984 | Yes |
| 9.3r | UPEC | B1 | PRJNA269984 | Yes |
| 11.1a | UPEC | A | PRJNA269984 | Yes |
| 12.1a | UPEC | B2 | PRJNA269984 | Yes |
| 17.1a | UPEC | B2 | PRJNA269984 | Yes |
| 20.1a | UPEC | B2 | PRJNA269984 | Yes |
| 21.1a | UPEC | B2 | PRJNA269984 | Yes |
| 26.1a | UPEC | B2 | PRJNA269984 | Yes |
| 31.1a | UPEC | B1 | PRJNA269984 | Yes |
| 31.3r | UPEC | B2 | PRJNA269984 | Yes |
| 34.1a | UPEC | B2 | PRJNA269984 | Yes |
| 35.1a | UPEC | B2 | PRJNA269984 | Yes |
| 41.1a | UPEC | B2 | PRJNA269984 | Yes |
| 41.4p | UPEC | B1 | PRJNA269984 | Yes |
| 56.1a | UPEC | B1 | PRJNA269984 | Yes |
| 56.3r | UPEC | B2 | PRJNA269984 | Yes |
