# Supplementary material for: Functional role of the type 1 pilus rod structure in mediating host-pathogen interactions
Source: eLife. 2018 Jan 18;7:e31662. doi: 10.7554/eLife.31662 (PMC5798934; doi:10.7554/eLife.31662)
Supplement: Supplementary file 7. [file elife-31662-supp7.docx]

| Position^a^ | AA Conservation | *dN*/*dS*^b^ | p-value^c^ | FimA_BW25113_^d^ | HA in PBS^e^ | HA in Mannose^e^ | Clumping Phenotype | Weaker Uncoiling | FimA_UTI89_^f^ | Ha in PBS^e^ | HA in Mannose^e^ | IBC Formation Defect | Bladder Colonization Defect | Gut Colonization Defect |
| --- | --- | --- | --- | --- | --- | --- | --- | --- | --- | --- | --- | --- | --- | --- |
| - | - | - | - | WT | 6 | 1 |  |  | WT | 8 | 0 |  |  |  |
| 5 | 99.89 | 0 | 0.014 | V5R | 6 | 1 | Yes |  |  |  |  |  |  |  |
| 22 | 99.84 | 0.07 | 0.001 | A22R | 6 | 1 |  | Yes | A22R | 8 | 0 | Yes | Yes | Yes |
| 25 | 99.73 | 0.161 | 0.012 | A25R | 3 | 1 |  |  |  |  |  |  |  |  |
| 32 | 99.95 | Infinite | 0.396 | V32R | 1 | 1 |  |  |  |  |  |  |  |  |
| 45 | 33.92 | 1.121 | 0.867 | E45R | 6 | 4 | Yes |  |  |  |  |  |  |  |
| 62 | 99.89 | 1.009 | 0.994 | D62R | 5 | 3 | Slight |  | D62R | 7 | 1 |  | Yes | Yes |
| 65 | 99.95 | Infinite | 0.396 | V65R | 1 | 1 |  |  |  |  |  |  |  |  |
| 85 | 99.95 | 0.098 | 0.011 | V85R | 1 | 1 |  |  |  |  |  |  |  |  |
| 92 | 99.95 | 0.522 | 0.650 | A92R | 7 | 1 |  | Yes |  |  |  |  |  |  |
| 114 | 100 | 0 | 0.000 | D114R | 6 | 3 | Slight | Yes | D114R | 8 | 3 | Yes | Yes | Yes |
| 121 | 62.69 | Infinite | 0.116 | E121R | 7 | 4 | Yes |  |  |  |  |  |  |  |
| 132 | 100 | Undefined | 1.000 | P132R | 6 | 1 |  | Yes | P134R | 8 | 0 | Slight |  |  |
| 142 | 52.08 | 1.646 | 0.445 | A142R | 5 | 4 | Yes |  |  |  |  |  |  |  |
| 145 | 99.62 | 0.03 | 0.000 | P145R | 0 | 0 |  |  |  |  |  |  |  |  |
| 155 | 100 | 0 | 0.045 | K155E | 6 | 1 |  | Yes |  |  |  |  |  |  |

^a^Codon positions are based on the global alignment shown in Figure 3a, which is the same as FimA_BW25113_.

^b^*dN*/*dS* ratios of "Infinite" indicate that dS = 0, while ratios of "Undefined" indicate that both dN and dS values = 0, thus preventing analysis. dN/dS >1 indicate adaptive selection and dN/dS <1 indicate purifying selection

­^c^p-values <0.1 are considered significant and are indicated in red text.

^d^The indicated mutations were made in the plasmid pTRC99a- FimA_BW25113_ and electroporated into UTI89∆LIR, *fimA*- for *in vitro* analysis. Vector control (pTRC99a with no FimA) displayed an HA titer of 2 in PBS and 1 in Mannose

^e^“PBS” indicates phosphate buffered saline and “Mannose” indicates PBS with 2% w/v methyl-α-D-mannopyranoside, an inhibitor of type 1 pilus binding. Values represent the last well in a 1:2 serial dilution with a visible hemagglutination (HA) phenotype. Each value shown the median average of 3 biological replicates each with 2 technical replicates. Values with a defect in HA are indicated in red.

^f^The indicated mutations in the mature protein were made in the chromosomal copy of the *fimA* gene in UTI89 for analysis *in vivo*. Not all mutations tested in plasmids were tested in the corresponding *in vivo* analyses.
